# siRNAs with decreased off-target effect facilitate the identification of essential genes in cancer cells

**DOI:** 10.18632/oncotarget.4269

**Published:** 2015-05-25

**Authors:** Chunyan Li, Zhenzhen Liu, Fang Yang, Wensheng Liu, Di Wang, Encheng Dong, Yu Wang, Chung-I Wu, Xuemei Lu

**Affiliations:** ^1^ CAS Key Laboratory of Genomic and Precision Medicine, Beijing Institute of Genomics, Chinese Academy of Sciences, Chaoyang District, Beijing, P. R. China; ^2^ University of Chinese Academy of Sciences, Shijingshan District, Beijing, P. R. China

**Keywords:** essential gene, siRNA, off-target, cell viability, transcriptome

## Abstract

Since the essential genes are crucial to the proliferation and survival of cancer cells, the interference of these genes is promising to be an option for cancer therapy to overcome heterogeneity. However, the essential genes are highly overestimated by RNA interference (RNAi) screenings, which is mainly caused by the pervasive off-target effect of small interference RNA (siRNA) and short hairpin RNA (shRNA). In the present study, we designed Match-Mismatch paired siRNAs to discriminate the on-target effect from off-target effect of siRNAs on cell viability. Only one of the 7 potential essential genes was validated as essential to cell viability, which demonstrates the high false positive rate in RNAi screenings. We modified the siRNA by introducing random nucleotides (N) into the guide strand to mitigate the off-target effect, without significantly compromising the on-target effect. The whole transcriptome profile analysis of cells transfected with siRNAs with or without Nindicates that siRNA-dN (with Ns on both the 2^nd^ and the 18^th^ bases of the guide strand) weakens the off-target effect by decreasing the unintended targets. The optimized siRNAs can be applied in the characterization of essential genes in cancer cells.

## INTRODUCTION

Genome-wide sequencing has revolutionized our understanding of cancer genetics, and hundreds to thousands of mutations have been characterized in each cancer type [1-4]. However, it remains difficult to determine the key mutations, which are essential to the progression of cancer cells, from hundreds of genes or functional domains alerted by somatic mutations. Essential genes are those that are indispensable for a certain organism under a certain condition [5]. Identification of essential genes for cell survival and proliferation of cancer cells is an efficient way to identify candidates for drug development and cancer therapy [6, 7]. High-throughput RNA interfering (RNAi) screenings are effective tools that are ubiquitously used to characterize essential genes [8, 9]. Small interference RNA (siRNA) and short hairpin RNA (shRNA) are central players in RNAi. However, the low validation rate and the lack of overlap between different screenings demonstrate high false positive rate in RNAi screening.

Luo and colleagues systematically identified cell essential genes in 12 cancer cell lines using 45,000 shRNAs targeting ∼9,500 human genes. Although 530 genes were supposed to be essential genes (of the top 5%) in at least 5 cell lines, only 2 essential genes were shared by two uncorrelated cell lines [10]. A pooled total of 8203 distinct shRNAs targeting 2924 genes were independently screened in 3 cancer cell lines (DLD-1, HCT 116, and HCC 1954), and only 25 genes were characterized as essential genes among these three cancer cell lines. Only 44 essential genes were shared by the two colorectal cancer cell lines (DLD-1 and HCT 116) (the number of essential genes for DLD-1 and HCT 116 is 88 and 151, respectively) [11]. Since the false positive rate of discovering essential genes will decrease with increase of cell line types in RNAi screening, Cheung et al. and Marcotte et al. assessed the essentiality of 11,194 genes in 102 human cancer cell lines and ∼16,000 genes in 72 human cancer cell lines, respectively [6, 12]. However, among the genes that were characterized to be essential in >50% of the cell lines in each study, no more than 60% of the essential genes were shared by these two screenings [6]. The divergence between different screenings demonstrates the high false positive rate in RNAi screenings. The dramatic overestimation on the essential genes is significantly caused by the off-target effects that result from the unintended gene interactions in different cell type context [13, 14].

Two main factors lead to the off-target effect of siRNAs. First, the unintended incorporation of the passenger strand (the strand of the duplex, that is not the guide strand) into the RISC (RNA-induced silencing complex) leads to the off-targets, which could be circumvented by appropriate thermodynamic asymmetry or by the 5′-phosphorylation on the guide strand and the 2′-O-methyl modification on the passenger strand [15-17]. The unintended targets remain widespread even when the guide strand is loaded into the RISC [18]. Second, a large proportion of off-targets showed 3′ UTR sequence complementarity to the siRNAs, especially to the 5′ end of the guide strand (miRNA-like off-target) [19, 20]. The siRNAs with low seed complement frequencies (SCFs) were experimentally validated to have minimal off-targets [21]. The modification of the seed region (2-8nt of the guide strand), such as 2′-O-methyl ribosyl substitution at position 2 of the guide strand, will enhance the specificity of siRNAs and reduce the off-target effect as well as dramatically increase the cost of siRNAs [22]. Alternatively, pooling siRNAs that target different regions of the target mRNA can diminish the off-target effect [23]. However, the high complexity of the pool makes the number of potentially problematic siRNAs be high. It is better to characterize the siRNAs with good performance and use the pool of these siRNAs for the further study.

In the present study, we developed a combined methodology for discriminating the siRNA on-target effect from the off-target effect using the paired Match-Mismatch siRNA strategy and decreasing the off-target effects by introducing random nucleotides (N) into the guide strand of siRNA. Previous studies reported that the nucleotide(s) replacement at the position 9 to 11 of the siRNA guide strand may result in the loss of most or all on-target effects [24-26]. To avoid the introduction of new off-targets by nucleotide replacement, we developed optimal negative controls (named as Mismatch) for siRNAs by replacing only one of the nucleotides at the position of 9 to 11 of the guide strand. The Mismatch siRNAs with nearly no on-target effect were used in this study. The effects caused by the ‘Mismatch’ siRNAs with minimal knockdown efficiency on the gene, therefore, are off-target. To minimize the off-target effects of siRNAs, we designed siRNAs avoiding complementary to the 3′ UTR of genes, screened siRNAs with low SCFs, and introduced N (random nucleotides) into the 2^nd^ and the 18^th^ bases of the guide strand to eliminate the off-target effect of siRNAs.

## RESULTS

### Match-Mismatch siRNA assays discriminate the siRNA target effect from the off-target effect

Dozens of genes, which were previously investigated to be generally essential for cancer cell viability [6, 7, 11, 27-32], were used as targets for paired Match-Mismatch siRNAs design. The Mismatch siRNAs were designed to have a nucleotide mismatch on the guide strand (as described in Materials and Methods). Using quantitative Real-Time PCR (qRT-PCR) to detect the target gene expression, we screened multiple paired siRNAs to assure that Match siRNAs had an effect on targets and Mismatch siRNAs could be used as optimal negative controls. The criteria were: (i) knock-down efficiency of Match should be greater than 60% compared to the blank control; (ii) knock-down efficiency of Match should be 50% higher than Mismatch.

Off-target effects increase along with the elevation of the siRNA concentration, and are not negligible when the concentration of siRNA is up to 20 nM [18]. Additionally, a high concentration of siRNA leads to toxicity by the competition of RISC with miRNAs *in vivo* [14]. To determine the optimal siRNA concentration without severe toxicity, a gradient of siRNA negative control (NC, without known targets in the transcriptome) was transfected into HCT 116 and the cell viability was analyzed (Figure S1). The results demonstrated that up to 10 nM, siRNA could lead to cell toxicity; therefore, a lower concentration of 5 nM was chosen in this study.

**Figure 1:**
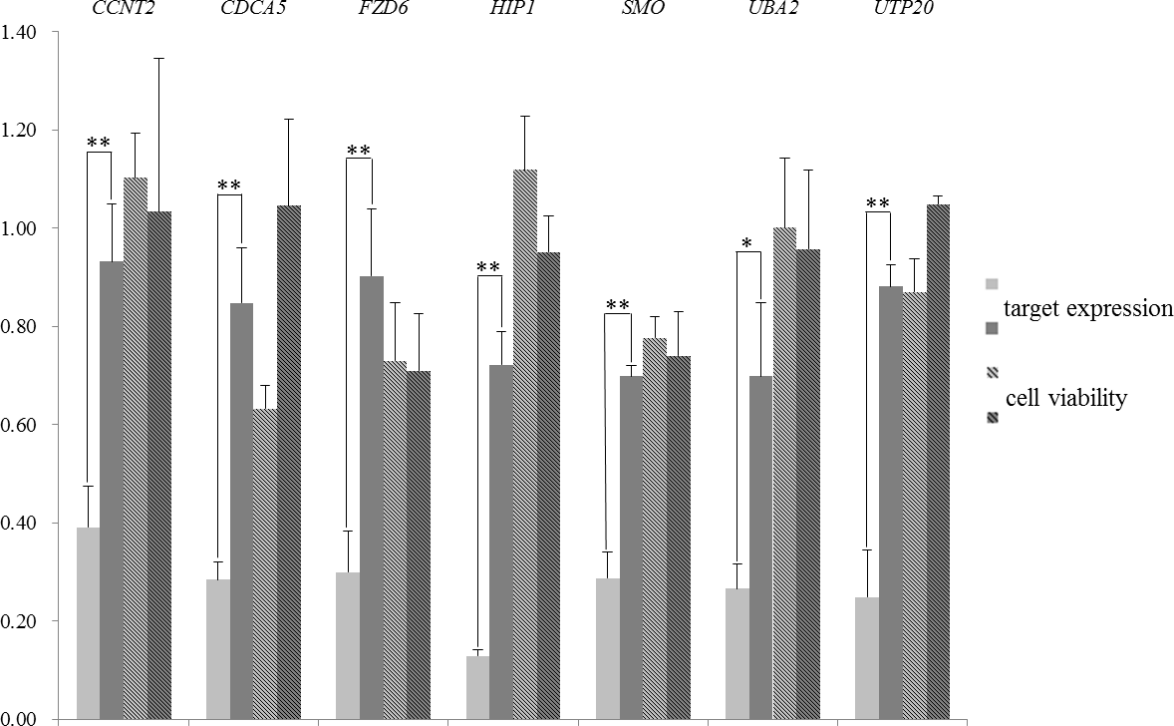
Gene expression of siRNA targets and cell viability after the transfection of Match (light gray bars) or Mismatch (dark gray bars) siRNAs in HCT 116 The target genes of siRNA oligos are labeled at the top of the graph. The y-axis denotes the relative expression level of siRNA target genes (bars without lines) and the cell viability (bars with lines), which were measured against the blank control cells by qRT-PCR and cell viability analysis, respectively (described in Materials and Methods). For the differential expression between cells transfected with Match and Mismatch siRNA oligos, statistically significant *P* values for the Student *t* test are marked by ** (*P* < 0.01), and * (*P* < 0.05) above the bars. Error bars represent the standard deviation.

After the filtering, 7 pairs of siRNAs remained (the siRNA sequences are listed in Table S1). Both the target expression level and cell viability were examined in HCT 116 after the siRNA transfection. The qRT-PCR results confirmed that the Match siRNAs had an acceptable knock-down efficiency, while the knock-down efficiency of Mismatch siRNAs was significantly lower than that of the Match (Figure 1; * for *P* < 0.05, and ** for *P* < 0.01). However, only 1 (*CDCA5*) of the 7 targets was likely to affect the cell viability in HCT 116. The cell viability dropped to below 0.80 when the *CDCA5* was targeted by Match siRNA, while the Mismatch siRNA did not have any effect on the cell viability (approximately 1.0). On the contrary, although the Mismatch siRNA had no effect on the expression of *FZD6*, the cell viability dropped to below 0.80. Therefore, the siRNA targeting *FZD6* exerted a strong off-target effect. Only *CDCA5*, one of the 7 genes that we screened, was validated as an essential gene, and the validation rate was lower than 15%. To delineate the genuine essential genes in cells, optimization of the shRNAs or siRNAs is necessary.

### The on-target effect of siRNAs is not significantly affected by the introduction of N

According to the general discrimination profile depicted by Huang et al., mismatches at the 5′ or 3′ end of the guide strand have a minor effect on the down-regulation of targets [25]. The introduction of N into these regions may have a negligible effect on the knock-down efficiency, and minimize the off-target effect as well. In the present study, 2 types of modified siRNAs (sN and dN) were designed through introducing N (an even mixture of A, T, G, and C) to the nucleotides on the guide strand (Table 1). The 0N represents the siRNA without the introduction of N. The sN is the siRNA with N on the 2^nd^ nucleotide of the guide strand. To further increase the complexity of pooling siRNAs and to dilute the off-target effect, the siRNA-dN was designed with N on both the 2^nd^ and the 18^th^ nucleotides of the guide strand.

**Table 1:** Design of siRNAs with N

siRNA type	sequences of the guide strand (5′-3′)	
	Match	Mismatch
−0N	ACCGUAUGAAG	ACCGUAUGAAA
	UACUUGGCdTdT	UACUUGGCdTdT
−sN	ANCGUAUGAAG	ANCGUAUGAAA
	UACUUGGCdTdT	UACUUGGCdTdT
−dN	ANCGUAUGAAG	ANCGUAUGAAA
	UACUUGNCdTdT	UACUUGNCdTdT

**Figure 2:**
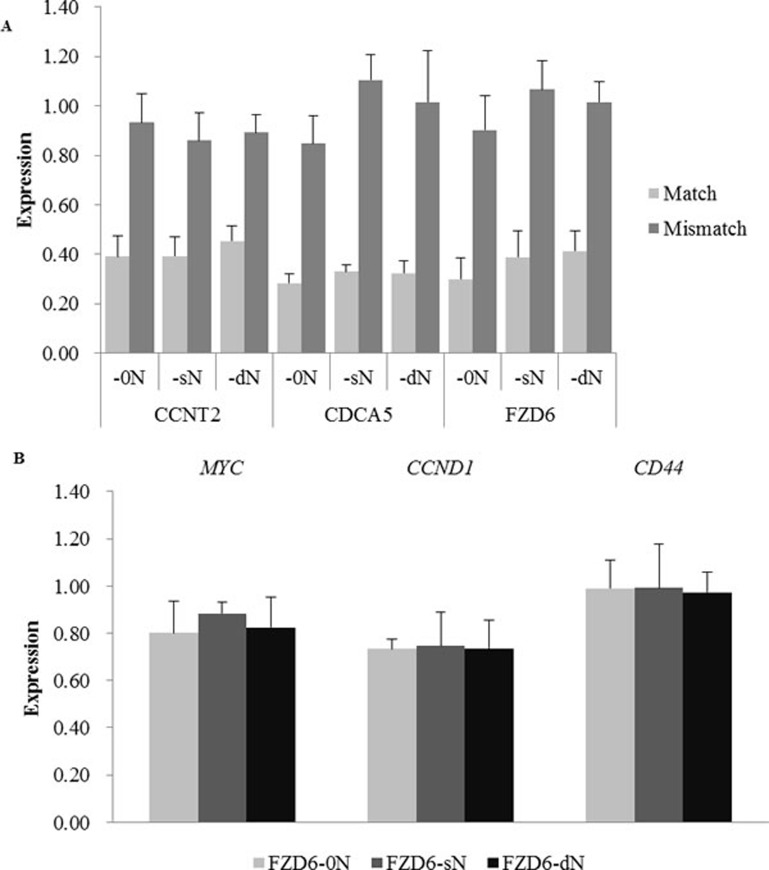
The expression of siRNA target genes and *FZD6* downstream genes after the transfection of siRNAs with N (−sN and -dN) or without N (−0N) **A.** The relative expression levels of three target genes after the transfection of Match (light gray bars) and Mismatch (dark gray bars) siRNAs with or without N were measured by qRT-PCR. The target genes and the type of siRNAs are labeled under the x-axis. **B.** The expression of 3 downstream targets (*MYC*, *CCND1*, and *CD44*) of *FZD6* in the Wnt pathway were examined by qRT-PCR, 24 hours after the transfection of siRNAs with or without N. Bars in light gray, dark gray, and black represent the cells transfected with Match siRNA without N (labeled as FZD6-0N), Match siRNA with single N on the 2^nd^ position of the guide strand (labeled as FZD6-sN), and Match siRNA with double Ns on the 2^nd^ and the 18^th^ position of the guide strand (labeled as FZD6-dN), respectively. Error bars represent the standard deviation.

To confirm that the introduction of N did not affect the knock-down efficiency of the on-targets, 3 (*CCNT2*, *CDCA5*, and *FZD6*) of the 7 previously analyzed targets were examined using 3 pairs of siRNAs (−0N, -sN, and -dN) for each target (Figure 2A). For all 3 targets, the introduction of N (either sN or dN) did not significantly weaken the on-target effects of Match siRNAs (pairwise Student *t* test, *P* > 0.05, *P* values are listed in Table S2), and the Mismatch siRNAs had little effect on the targets.

The slight difference between knockdown efficiencies of the siRNAs with N and without N could have cascade effects on the downstream genes of siRNA targets, leading to the functional changes of the targeting pathway and consequently, the related cell phenotypes. *FZD6* encodes 7-transmembrane domain proteins, which are receptors for Wnt signaling [33]. According to the “Wnt/beta-Catenin Signaling” annotation in Cell Signaling Technology, we examined the expression of the direct downstream genes regulated by *FZD6* in Wnt signaling pathway (http://www.cellsignal.com/contents/science/cst-pathways/science-pathways). According to the expression of six downstream genes that was detected by RNA-sequencing after the transfection of FZD6-0N or FZD6-sN/dN in the cell line, their expression levels in FZD6-sN/dN transfection did not significantly differ from that in FZD6-0N transfection (Table S4). Three (*MYC*, *CCND1* and *CD44*) of the six downstream targets were reported to be transcriptionally regulated by the Wnt signaling in the colon carcinoma cells from which the HCT 116 (the cell line we used in this study) was derived [34-36]. The expression level of these three downstream genes with FPRK (fragments per kilobase of exon model per million mapped reads) > 20 in all 4 samples was further validated by qRT-PCR (Figure 2B and Table S4). The expression levels of these three downstream targets in the transfections of siRNAs with N and without N were not different from each other, which was consistent with the RNA-Seq results (Figure S2 and Table S4). The *P* values of analysis of variance (ANOVA) were 0.667, 0.989, and 0.983 for *MYC*, *CCND1* and *CD44*, respectively (Table S4). The results above indicated that the introduction of N did not affect the on-target effect.

### The introduction of N into siRNAs minimizes the number of the unintended targets and weakens the off-target effect

**Figure 3:**
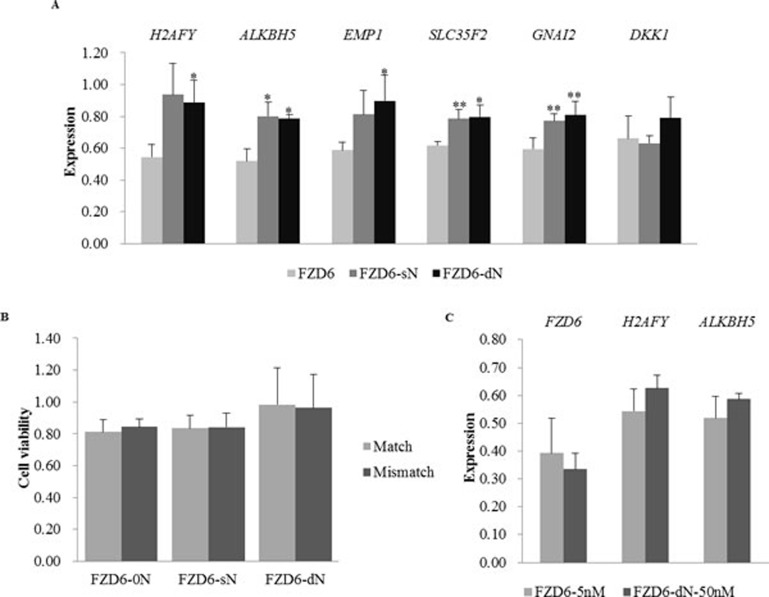
The effects of siRNAs with or without N on the gene expression of *FZD6* and six unintended targets and on cell viability **A.** The expression of 6 unintended target genes after the transfection of FZD6 siRNAs with or without N. The bars in different colors represent the cells transfected with siRNAs with or without N, as described in Figure 2B. The off-target effects on the 6 unintended genes’ expression were significantly reduced (*, *P* < 0.05; **, *P* < 0.01) by introducing N to the siRNAs. **B.** The cell viability after the transfection of Match (light gray) and Mismatch (dark gray) siRNAs with N (FZD6-sN and FZD6-dN) or without N (FZD6-0N). The drop in cell viability (y-axis) is rescued by the introduction of N into the siRNA oligos (from ∼0.80 to ∼1.00). **C.** The expression of *FZD6* and 2 unintended targets after the transfection of FZD6-0N (5 nM; light gray) or FZD6-dN (50 nM; dark gray). Error bars represent the standard deviation.

Because the Match siRNA targeting *FZD6* had severe off-target effect (Figure 1), whole genome expression profiles of cells with or without the transfection of *FZD6* siRNAs were delineated by RNA-Seq to elucidate the genome wide off-targets (the statistics of the RNA-Seq data is shown in Table S3). The transcriptome of cells transfected with Match siRNA without N (FZD6-0N) had the largest differences from the transcriptome of the blank control cells, compared to those of sN and dN transfected cells (Figure S3). Compared to the blank control, the numbers of the significantly differentially expressed genes in the cells transfected with FZD6-0N, FZD6-sN, and FZD6-dN were 189, 168, and 97, respectively (fold change >1.5 and *P* < 0.01; Figure S4). The smallest number of genes was affected by FZD6-dN at the transcriptional level. Among the 189 genes, 6 genes with FPKM > 20 in all of the 3 samples were randomly selected to validate their expression levels using qRT-PCR. The expression of these 6 genes in FZD6-sN and FZD6-dN transfected cells was more closed to their expression in 0C than that in the FZD6-0N transfection, which was consistent with the RNA-seq data (Figure 3A and Table S5). It indicates that the unintended effects on the genes’ expression were significantly reduced (*, *P* < 0.05; **, *P* < 0.01) by introducing N to the siRNAs.

The function of the differentially expressed genes in cells after the siRNA transfection was analyzed by DAVID (http://david.abcc.ncifcrf.gov/). GO (Gene Ontology) terms with *P* < 0.01 are listed in Table 2. In the cells transfected with siRNA FZD6, the genes were enriched in GO terms related to cell death and apoptosis. The expression variation of these genes may cause the off-target effects on cell viability. Among the 189 genes revealed in FZD6 transfection, 16 genes were annotated to be involved in cell-death. Compared to the blank control, 10 and 5 of the 16 genes were differentially expressed in cells transfected with sN and dN siRNA oligos, respectively, and the difference was even smaller in the FZD6-dN transfected cells than the FZD6-sN transfected cells (Table S6). It suggests that the off-target effect of FZD6-0N on cell viability is largely resulted from the unintended genes, which is reduced by introducing Ns to the siRNA oligos (Figure 3B).

**Table 2:** Enriched GO terms (*P* < 0.01) by DAVID functional annotation of the differentially expressed genes after the transfection of siRNAs targeting *FZD6*

GO term	number of genes	*P* value
**after the transfection of FZD6-0N**		
cell death	15	2.0E-3
death	15	2.2E-3
apoptosis	13	3.6E-3
programmed cell death	13	4.0E-3
NADP metabolic process	3	7.3E-3
**after the transfection of FZD6-sN**		
positive regulation of anti-apoptosis	4	8.3E-4
regulation of anti-apoptosis	4	1.7E-3
induction of apoptosis by intracellular signals	4	4.6E-3
response to organic substance	12	4.9E-3
response to organic cyclic substance	5	6.8E-3
cofactor metabolic process	6	7.3E-3
response to inorganic substance	6	8.9E-3
**after the transfection of FZD6-dN**		
response to organic substance	8	6.3E-3

To examine whether the off-target effect of siRNA-dN was increased with the enhancement of the on-target effect by increasing the concentration of siRNA oligos, the expression of *FZD6* and 2 potential off-target genes (*H2AFY* and *ALKBH5*) was examined by qRT-PCR. Although the knock-down efficiency of *FZD6* was slightly stronger after increasing the concentration of the dN siRNA oligo, the off-target effect was still weaker than that exerted by the unmodified siRNA (Figure 3C). The increase of dN siRNA concentration enhances on-target effect rather than off-target effect.

## DISCUSSION

Large pools of shRNAs have been used for highly parallel multiplex screening for essential genes in human cell lines [6, 7, 11]. The validation of the essential genes from the screening of human mammary cells revealed that cell viability could drop to below 50% even when the targets have no obvious depletion after the shRNA transfection (the Figure 2B in [7]). The number of essential genes characterized in previous RNAi screenings is listed in Table S7. Although 4 or even more siRNAs/shRNAs were designed for each gene, the essential gene enrichment analysis was frequently done based on the 1-2 siRNAs/shRNAs with strongest effect [6, 12]. Not all siRNAs/shRNAs are effective, whereas the estimation based on 1-2 siRNAs/shRNAs is insufficient to deplete the off-target effect. These studies disclosed that most of the false positive candidates were caused by off-targets, which were prevalent in RNAi screenings. The result in this study shows that the paired Match-Mismatch siRNAs can be applied to discriminate the on-target and off-target effects of the candidate essential genes effectively.

The pooling strategy has been used to diminish the off-target effect of siRNAs [23]. Several siRNAs with a high efficiency of on-target effects have to be screened out, which will elevate the cost. Based on the pooling strategy, introducing N (an even mixture of A, T, G, and C) into the guide strand may be an alternative to dilute the off-target effect. In this study, we designed 2 types of modified siRNAs (sN and dN) to eliminate the off-target effect. The results of transcriptome analysis and cell viability measurement in cancer cell line demonstrate that siRNAs with dN are a promising and easy approach to characterize essential genes by minimizing the off-target effect. The limitation of this strategy is that to minimize the false positive rate in siRNA screenings for essential genes in cancer cell lines, only siRNAs with strong on-target effect should be used in the introduction of dN to minimize the off-target effect.

Although a limited proportion of genes were essential for each cancer cell line, the essential genes were divergent among different cancer types, even among subtypes [6, 7, 11, 12]. The high level of complexity in gene interaction networks maintains cellular homeostasis, fitness, and survival in both normal and cancer cells [37-39]. Additionally, drug-resistance and relapse are inevitable in single-target therapy because of the heterogeneity found within tumors. Predictably, the large-scale screenings of functional important genes will be required in gene network characterizations and in the development of individualized cancer therapy. To do a large set of RNAi screening in as many cancer cell lines as possible is labor-intensive and makes the ranking algorithms for essential genes even harder. It is convenient to knock-down multiple essential genes at the same time using combined siRNAs with dN, developed herein.

The single nucleotide variations (SNVs) are divergent among different cancer types and different cancer patients, even among the cancer cells within the tumor [40]. The number of SNVs varies from hundreds to millions in exome [41]. The somatic SNVs in the coding region of tumors can be used in the siRNA screening to discriminate cancer cells from normal cells, since tumor-specific siRNAs are mismatched and hence may not effectively bind to the gene region with a single nucleotide difference in normal cells [25, 26]. Based on the somatic SNVs detected in tumors, the optimized Match-siRNAs in this study can be utilized in developing personalized cancer therapy by specifically targeting essential genes in cancer cells and eliminating the toxicity of siRNAs in normal cells in the precision medicine.

## MATERIALS AND METHODS

### siRNA design and synthesis

To minimize the off-target effect of siRNAs, each oligo targets less than 2 genes in which more than 12 consecutive basepairs are complementary to the guide strand of the siRNA oligos. The 3′UTR is avoided for the target region of siRNAs. The siRNAs that are completely complementary to the targets are named ‘Match’, while the siRNAs with a non-complementary nucleotide to the targets are named ‘Mismatch’. The Mismatch siRNAs are designed according to the previously mentioned rule [25]. The sN and dN oligos are 2 types of modified siRNAs: the sN is the siRNA with a random nucleotide (N) on the 2^nd^ base of the guide strand, and dN is the siRNA with Ns on both the 2^nd^ and the 18^th^ bases of the guide strand. All of the siRNAs were synthesized by Guangzhou RiboBio Co., Ltd.

### Cell culture

The HCT 116 cell line was obtained from the cell bank of Shanghai Institutes for Biological Sciences, CAS. The cells were cultured in McCOY's 5A (Sigma) plus 10% FBS (Hyclone) with penicillin (100 U/ml) plus streptomycin (100 μg/ml) (Sigma) at 37°C with 5% CO_2_ in a humidified incubator (Thermo Scientific), and were expanded on a 6-cm dish until the cells were seeded on 96/24-well plates. The cells were counted using a hemocytometer.

### siRNA transfection

Cells were seeded into the wells one day before transfection. For cell viability analysis, 2×10^3^ HCT 116 cells in 50 μl of medium without antibiotics were seeded in each of the internal 60 wells, and 200 μl of PBS were added to each of the outside 36 wells of the 96-well plate. For knock-down efficiency detection, 3×10^4^ HCT 116 cells in 400 μl of medium without antibiotics were seeded in each well of the 24-well plates.

The transfection was mediated by Lipofectamine™ 2000 (Invitrogen) according to the manufacturer's instructions. The medium was replaced by 200 μl or 500 μl of fresh medium without antibiotics for 96-well and 24-well plates separately, 6 hours after transfection. The final concentration of siRNA was 5 nM unless noted otherwise.

### Cell viability analysis

Three days after transfection, cell viability was measured using the CellTiter 96® AQueous One Solution Cell Proliferation Assay (Promega). A volume of 20 μl CellTiter 96® AQueous One Solution Reagent was pipetted into each well of the 96-well plates containing samples. After incubation at 37°C for 2 hours, the absorbance at 490 nm was recorded. More than 3 independent experiments were carried out for each treatment, and triplicates were conducted in the same plate. The cell viability was the ratio of the absorbance at 490 nm divided by the blank control (0C), which was transfected with the transfection reagents without siRNA oligo.

### qRT-PCR (quantitative real-time PCR)

qRT-PCR was carried out 24 hours after the siRNA transfection to quantitate the on-target and the off-target effects. Total RNA was extracted using RNA prep Pure Cell/Bacteria Kit (Tiangen). A total of 1 μg of extracted total RNA from each sample was reverse-transcribed by Reverse Transcription System (Promega) according to the manufacturer's protocol. qRT-PCR experiments were performed using Maxima SYBR Green/ROX qPCR Master Mix (Thermo) in triplicate 20 μl reactions according to manufacturer's protocol on an AB 7500. Thermal cycling was organized in 2 steps: an initial denaturation step of 10 min at 95°C, followed by 40 repeated cycles of 95°C for 10 sec and 60°C for 30 sec. Melting curves were obtained by increasing the temperature from 60°C to 95°C with a plate reading every 0.2°C. The housekeeping gene *GAPDH* was used as an endogenous control. Primer sequences of candidate genes were designed on the basis of sequence data obtained from the NCBI database (http://www.ncbi.nlm.nih/org). The expression level of each sample was normalized according to its *GAPDH* content. The ΔΔCt method was used to measure the expression level of each candidate. ΔCt = Ct (target)− Ct (*GAPDH*). ΔΔCt = ΔCt (sample) –ΔCt (0C). The fold change of each gene was calculated by the equation 2^−ΔΔCt^, which represented each gene's relative expression level against the blank control cells (0C) [42].

### RNA-Seq library construction

Total RNA was extracted using a RNA prep Pure Cell/Bacteria Kit (Tiangen). Three milligrams of total RNA from each of the 3 independent experiments was mixed for the whole transcriptome library construction. The library preparation was performed according to the TruSeq™ RNA Sample Preparation Guide standard protocol. Sequencing was conducted on a Hi-Seq 2000 (Illumina).

### Transcriptome sequencing, mapping and expression analysis

Sequencing reads of 101 bp in length were aligned to the reference genome (GRCh37.73/hg19, downloaded from Ensembl database, ftp.ensembl.org) with the Tophat alignment software tools [43]. The uniquely aligned reads were used for calculation by Cufflinks software and the gene expression levels were normalized using the FPKM (fragments per kilobase of exon model per million mapped reads) [44]. The expression level of each gene is listed in Table S5. The results of differentially expressed genes were analyzed and visualized by the Bioconductor function “CummeRbund” in the R language [45]. Gene expression RNA-Seq data have been deposited in the Gene Expression Omnibus (GEO) archive with the accession number of GSE62968.

### Statistical analysis

Data are expressed as the mean ± standard deviation from at least 3 independent experiments performed in at least duplicate. The differences were considered significantly different at *P* < 0.05 (denoted by *) and *P* < 0.01 (denoted by **) using a double-sided Student *t* test.

## SUPPLEMENTARY MATERIAL FIGURES AND TABLES


