# Supplementary material for: siRNAs with decreased off-target effect facilitate the identification of essential genes in cancer cells
Source: Oncotarget. 2015 May 25;6(25):21603–13. doi: 10.18632/oncotarget.4269 (PMC4673289; doi:10.18632/oncotarget.4269)
Supplement: Supplementary file 1 [file oncotarget-06-21603-s001.pdf]

siRNAs with decreased off-target effect facilitate the identification of essential genes in cancer cells

Supplementary Material

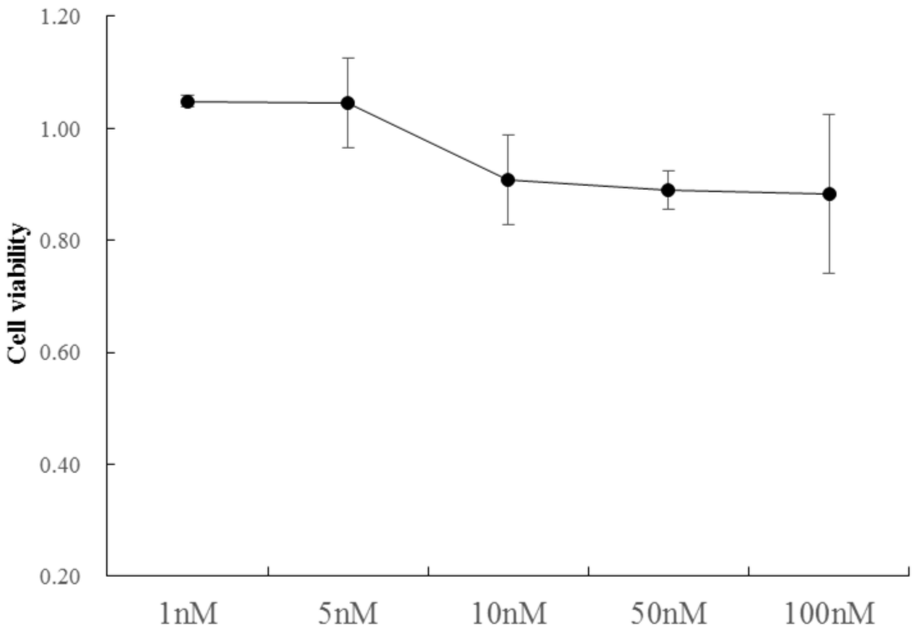

Figure S1: The cell viability after the transfection of NC siRNA with the concentration gradient from 1 nM to 100 nM, compared to the blank control. Error bars represent the standard deviation.

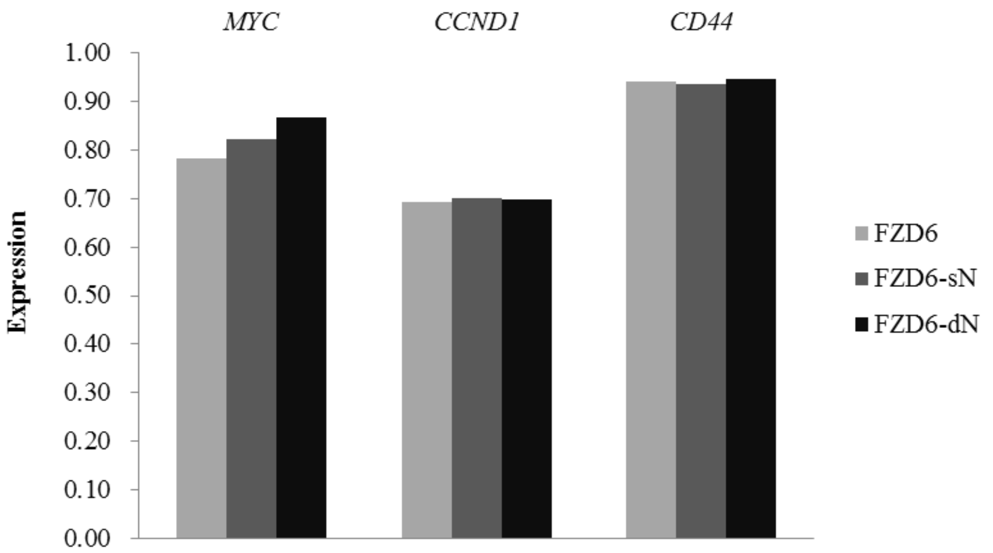

Figure S2: The expression of three downstream genes of *FZD6* after the transfection of siRNAs with N (FZD6-sN and FZD6-dN) or without N (FZD6-0N) in RNA-Seq data. The y-axis denotes the expression ratio of the three downstream targets (*MYC*, *CCND1*, and *CD44*), which was measured against the blank control and determined by RNA-Seq data. The bars in different colors represent the cells transfected with siRNAs with or without N, as described in Figure 2B.

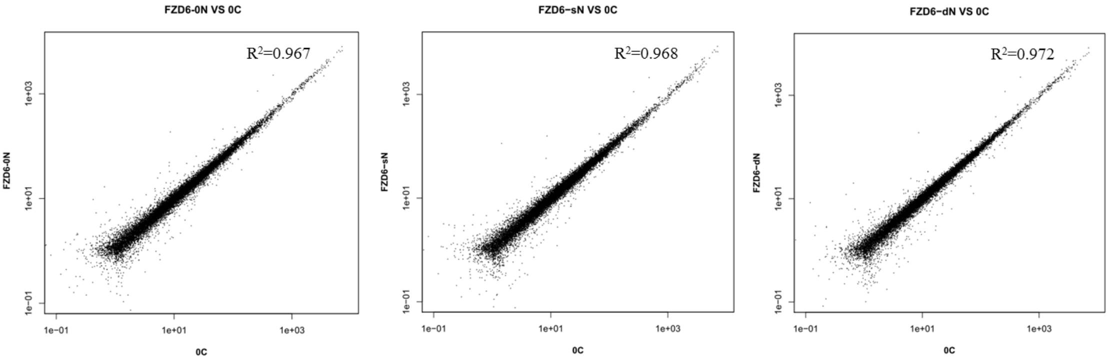

Figure S3: The pairwise correlation coefficient of transcriptome profiles in the cells transfected with siRNA (FZD6-0N, FZD6-sN and FZD6-dN) and the cells transfected with the blank control (0C).

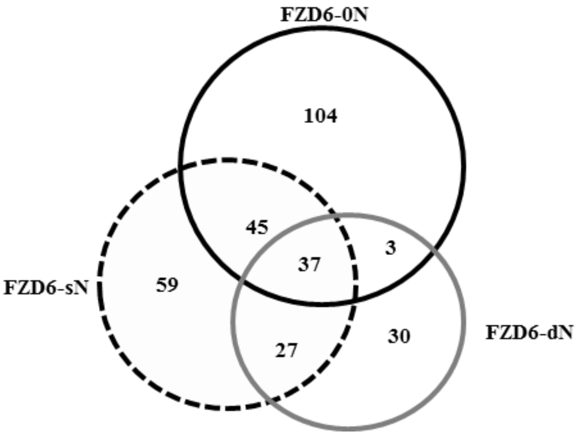

Figure S4: The numbers of genes that are significantly differentially expressed (fold change > 1.50 and $P < 0.01$) in the cells transfected with siRNAs (FZD6-0N, FZD6-sN and FZD6-dN) against the cells transfected with 0C are shown in the 3 circles. The numbers of shared genes are labeled in the overlapped areas.

Table S1: The sequences of siRNA oligos that used in this study

	guide strand
UBA2-Match	UAGUAUCCAGAUCAAUCAGdTdT
UBA2-Mismatch	UAGUAUCCACAUCAAUCAGdTdT
UTP20-Match	UCACUUAAACUCAUAUCUCdTdT
UTP20-Mismatch	UCACUUAAAGUCAUAUCUCdTdT
CCNT2-Match	ACCGUAUGAAGUACUUGGCdTdT
CCNT2-Mismatch	ACCGUAUGAAAUACUUGGCdTdT
CDCA5-Match	AAUGAUGAUCUCAACUCUGdTdT
CDCA5-Mismatch	AAUGAUGAUCACAACUCUGdTdT
FZD6-Match	UCCUAGGUAAUCAUGGCUAdTdT
FZD6-Mismatch	UCCUAGGUACUCAUGGCUAdTdT
HIP1-Match	UAUAGUCGCUCAAUUAAGUdTdT
HIP1-Mismatch	UAUAGUCGCUGAAUUAAGUdTdT
SMO-Match	UGAAGAAGUCGUAGAAGUGdTdT
SMO-Mismatch	UGAAGAAGACGUAGAAGUGdTdT
CCNT2-sN-Match	ANCGUAUGAAGUACUUGGCdTdT
CCNT2-sN-Mismatch	ANCGUAUGAAAUACUUGGCdTdT
CCNT2-dN-Match	ANCGUAUGAAGUACUUGNCdTdT
CCNT2-dN-Mismatch	ANCGUAUGAAAUACUUGNCdTdT
CDCA5-sN-Match	ANUGAUGAUCUCAACUCUGdTdT
CDCA5-sN-Mismatch	ANUGAUGAUCACAACUCUGdTdT
CDCA5-dN-Match	ANUGAUGAUCUCAACUCNGdTdT
CDCA5-dN-Mismatch	ANUGAUGAUCACAACUCNGdTdT
FZD6-sN-Match	UNCUAGGUAAUCAUGGCUAdTdT
FZD6-sN-Mismatch	UNCUAGGUACUCAUGGCUAdTdT
FZD6-dN-Match	UNCUAGGUAAUCAUGGCNAdTdT
FZD6-dN-Mismatch	UNCUAGGUACUCAUGGCNAdTdT
HIP1-dN-Match	UNUAGUCGCUCAAUUAANUdTdT
HIP1-dN-Mismatch	UNUAGUCGCUGAAUUAANUdTdT
SMO-dN-Match	UNAAGAAGUCGUAGAAGNGdTdT
SMO-dN-Mismatch	UNAAGAAGACGUAGAAGNGdTdT

Table S2: Statistical analysis of target expression (on-target effect) between siRNAs with N and without N

Target	Comparison*	*P* values	
		Match	Mismatch
CCNT2	sN vs. 0N	0.987	0.404
	dN vs. 0N	0.279	0.582
CDCA5	sN vs. 0N	0.161	0.043
	dN vs. 0N	0.309	0.307
FZD6	sN vs. 0N	0.322	0.187
	dN vs. 0N	0.173	0.301

*The expression comparisons of target genes after the transfection of sN, dN or 0N siRNAs.

Table S3: Statistical information of the RNA-Seq data

		reads filtered		reads mapped		reads uniquely mapped	
	total reads #	#	Ratio	#	Ratio	#	Ratio
0C	56,090,057	56,083,898	99.99%	51,915,633	92.56%	42,258,499	75.35%
FZD6-0N	88,918,569	88,910,612	99.99%	82,117,531	92.35%	66,684,078	75.00%
FZD6-sN	125,301,731	125,289,139	99.99%	118,698,764	94.73%	95,898,189	76.54%
FZD6-dN	85,540,868	85,533,699	99.99%	81,779,183	95.60%	65,847,778	76.98%

Table S4: The expression level of the targets downstream of Wnt signaling

Gene Name	RNA-Seq (FPKM)			qRT-PCR (fold changes of three independent experiments)*									
	FZD6-0N	FZD6-sN	FZD6-dN	FZD6-0N/0C			FZD6-sN/0C			FZD6-dN/0C			*P* values of ANOVA
				1	2	3	1	2	3	1	2	3	
MYC	142.578	149.444	157.735	0.9431	0.6751	0.7864	0.9371	0.8440	0.8700	0.9747	0.7499	0.7490	0.6673
CCND1	325.373	329.545	327.788	0.7815	0.7122	0.7074	0.9090	0.6901	0.6375	0.8715	0.6626	0.6682	0.9890
TCF7	18.1034	17.4489	16.2										
PPARD	16.8029	16.373	15.5707										
AXIN2	10.9983	8.31831	8.45874										
CD44	210.646	209.645	211.509	0.9150	1.1281	0.9257	0.9292	1.2013	0.8484	1.0076	1.0383	0.8752	0.9832

* each gene's relative expression level against the blank control cells (0C).

Table S5: The expression of each Ensembl gene

Gene Name	FPKM			
	0C	FZD6-0N	FZD6-sN	FZD6-dN
TSPAN6	20.8709	20.8929	20.2119	22.3483
DPM1	62.3228	52.8707	50.6733	56.4642
SCYL3	3.33476	3.45317	3.43001	3.75131
C1orf112	7.85242	6.20479	6.44831	7.73788
FUCA2	59.162	53.4728	57.7635	58.3624
GCLC	52.3525	42.0892	38.8611	39.1362
NFYA	18.0514	14.6665	15.2744	15.7784
STPG1	4.94994	5.01043	4.49879	4.54282
NIPAL3	4.88837	5.15252	5.03482	4.5389
LAS1L	44.4284	38.7797	39.255	40.4692
ENPP4	5.0528	4.61091	5.12006	5.15596
SEMA3F	7.02601	7.78098	8.46628	7.43938
ANKIB1	7.96319	9.85644	9.698	9.62516
CYP51A1	35.2435	28.2359	28.2566	32.1302
KRIT1	10.8985	12.1178	13.6583	12.7849
RAD52	14.1204	13.0296	14.206	13.5996
BAD	45.301	36.7784	41.6357	39.7343
LAP3	54.6894	54.5769	53.6399	55.8955
CD99	22.8522	24.9672	26.1636	28.0137
HS3ST1	67.1098	54.621	62.895	54.9188
WNT16	31.8864	36.9105	38.5409	41.02
HECW1	2.02142	3.08153	2.82914	3.41007
MAD1L1	24.148	22.4024	23.3306	19.7692
LASP1	85.4596	86.3095	90.535	91.4912
SNX11	19.5423	16.5903	18.0731	18.1932
M6PR	165.983	146.455	159.047	154.109

KLHL13	2.63317	2.2658	2.16611	3.00398
CYP26B1	7.72323	7.33568	9.05208	7.47056
ICA1	4.32414	6.69401	6.58773	6.94766
DBNDD1	34.9231	38.3	38.9627	36.4235
ALS2	7.84169	7.9792	7.80418	8.50353
CFLAR	12.8231	13.5204	14.0813	15.3616
TFPI	4.69836	4.69885	5.53952	5.7351
NDUFAF7	9.96375	10.6877	10.8831	11.4219
RBM5	41.0632	33.0233	37.4103	35.7034
SLC7A2	4.52954	3.99052	3.93113	4.44514
ARF5	208.867	228.811	232.863	226.625
POLDIP2	54.6165	48.6584	50.1968	48.3748
PLXND1	20.1233	21.6235	23.91	21.892
AK2	153.58	133.475	128.792	142.617
FKBP4	254.744	212.853	200.122	214.077
KDM1A	134.769	117.203	118.718	118.894
RBM6	24.2416	22.081	21.8219	22.5562
CAMKK1	3.72807	3.55444	4.53651	4.51163
RECQL	12.902	12.1615	12.0754	12.9532
CCDC132	7.24941	6.41884	7.53648	7.5088
ARHGAP33	4.64695	5.5178	5.25478	5.14953
NDUFAB1	282.485	263.186	268.005	272.724
ZMYND10	1.16711	1.13255	1.2818	1.09331
SLC25A13	9.11901	9.01284	10.4521	10.2031
ST7	9.79188	8.03215	7.97184	8.37556
CDC27	79.2607	74.8481	75.3086	84.2236
HCCS	32.7648	29.75	28.6113	30.9881
DVL2	36.5929	38.172	38.8498	36.1169
PRSS22	3.70823	4.465	5.63218	4.07614

UPF1	46.0099	41.0387	44.6707	43.1077
SKAP2	6.72414	5.42449	6.00336	6.60465
SLC25A5	641.418	516.82	625.586	640.034
CCDC109B	18.9591	19.2489	20.814	19.5456
HOXA11	5.65112	4.49592	4.20301	4.6836
POLR2J	55.1632	52.6687	52.3451	52.3136
DHX33	21.8052	19.2967	19.7564	20.896
LIG3	22.9415	20.7868	23.6873	22.9912
RPAP3	28.805	23.3062	22.8568	28.7711
ACSM3	2.14358	2.5522	1.78556	2.24067
AC004381.6	3.65254	3.79871	4.08384	4.545
CIAPIN1	98.9439	77.0126	84.8792	97.0932
SPPL2B	21.3375	25.5569	27.0751	23.3062
FAM214B	3.44367	3.10384	4.6742	4.26474
PRKAR2B	6.53378	5.37707	5.1111	5.81596
MSL3	5.80005	5.80319	5.37837	6.16772
CREBBP	12.8701	10.2752	11.1263	11.7743
GCFC2	11.7817	10.5896	11.4027	12.7291
WDR54	28.53	28.7098	27.6273	25.8926
KMT2E	7.9033	6.84223	7.12846	6.39355
RHBDD2	23.1605	25.4107	20.7707	21.7609
SOX8	3.14757	3.13219	3.54902	3.42056
IBTK	10.3322	11.2912	10.9698	11.151
ZNF195	14.5301	16.3863	15.0218	15.4759
MYCBP2	16.7556	14.2876	15.7991	15.5864
FBXL3	21.0936	19.7126	20.8381	18.9217
PDK2	43.5057	37.4896	44.8241	39.6128
ITGA3	119.12	132.391	141.059	139.06
ZFX	5.28246	4.04344	3.87852	4.3461

LAMP2	23.5733	23.0561	21.199	22.8926
GGNBP2	15.4017	14.4572	13.1287	15.9512
ITGA2B	1.42435	1.38488	1.42549	1.41602
GDE1	34.9446	32.5851	35.7544	36.5884
C19orf60	57.8505	72.6934	75.2718	62.9429
CRLF1	34.6973	29.9269	35.1159	36.1814
OSBPL7	5.30942	5.9822	6.66321	6.07903
TMEM98	31.1824	31.2628	25.1925	25.3385
MAP3K14	9.64088	6.77818	7.63089	7.31602
SYNRG	6.89098	6.66678	6.96749	8.31003
TMEM132A	52.3526	62.0695	70.7113	59.8224
AP2B1	57.4624	71.8474	62.9739	59.7039
ZNF263	23.2656	21.036	20.4322	20.8178
SPATA20	21.238	26.6579	28.8519	25.7274
TNFRSF12A	268.979	208.469	319.584	301.722
MAP3K9	3.44534	2.3911	3.09221	3.17464
RALA	22.5014	22.41	21.1976	23.7834
BAIAP2L1	21.9855	21.8347	21.0757	21.8455
JHDM1D	1.3636	1.69931	1.75097	1.45896
ETV1	4.39393	5.03608	5.577	4.76406
AGK	20.1744	15.5643	16.435	17.5154
ALDH3B1	6.95113	9.52211	12.4405	10.9674
PHTF2	13.4301	10.8322	11.527	13.2033
FARP2	19.8152	15.5298	14.3991	16.1389
USH1C	3.99939	6.18498	6.21709	6.95737
GGCT	46.2203	45.6561	42.9397	48.4652
DBF4	35.9781	31.9827	29.6208	36.4565
TBXA2R	3.10563	2.22815	2.75783	2.28017
IFRD1	26.8954	27.5012	22.0908	23.1348

COX10	15.2675	15.3806	16.2831	15.9415
GTF2IRD1	8.55971	9.04589	8.13247	7.67395
PAF1	48.822	43.4909	44.9974	46.2637
VPS41	20.5277	21.4073	23.3909	21.3034
ARHGAP44	2.28858	2.31816	2.51442	2.57792
ELAC2	118.174	102.137	119.128	109.843
ARSD	2.18527	3.32354	3.61712	2.75365
PNPLA4	10.3567	13.5721	12.8309	15.5204
ADIPOR2	38.7154	33.8573	36.6995	39.2113
CDKL3	1.66239	2.04924	2.1725	2.68952
MARK4	11.6309	10.406	13.3509	11.2287
PROM1	12.0152	12.2588	10.8127	12.4892
CCDC124	178.031	166.972	171.687	161.366
PAFAH1B1	58.9628	46.0273	48.2858	48.5544
KIAA0100	61.939	52.761	58.6118	57.6373
GAS7	1.17045	0.997303	1.2403	1.00871
TRAPPC6A	4.66781	9.66304	8.96825	7.72469
ST7L	5.29662	6.11035	5.97059	7.34113
PAX6	1.65211	1.39682	1.52173	1.63898
RPUSD1	100.815	77.711	89.5476	85.2066
RHBDF1	6.61075	6.88062	8.67287	7.56779
LUC7L	23.4394	20.052	21.9577	23.1401
CACNA2D2	3.46081	3.92954	4.02057	3.95213
BAIAP3	5.31123	5.42281	7.37108	4.87429
TSR3	79.6459	66.4524	70.3902	66.9346
PIGQ	6.28408	8.81246	7.72882	7.14725
CRAMP1L	10.0263	8.85378	9.04368	8.93376
TEAD3	12.044	10.7232	13.873	13.0124
DNAJC11	48.7716	45.0165	47.0617	48.5362

MYLIP	3.11922	2.55188	2.51487	2.46054
E2F2	7.8159	7.94802	7.96762	7.67015
PSMB1	387.211	364.148	361.399	376.187
JARID2	5.18894	5.17822	5.39432	5.58267
CDK11A	31.5169	29.5409	31.1539	30.6171
NADK	73.1493	60.2648	62.5255	56.1286
CYTH3	11.3629	8.44386	9.60912	9.16738
ADAM22	2.56666	2.6774	2.71483	2.63222
SYPL1	69.4787	49.7295	47.6749	53.6303
CYB561	49.8924	40.3679	48.9754	46.7736
SPAG9	34.2591	29.6964	28.3731	29.5653
CELSR3	1.4715	2.09993	2.56943	1.9356
PLEKHG6	17.8903	18.6125	19.5515	16.0525
SS18L2	28.0016	25.0875	20.9697	27.7674
MPND	15.2074	18.1953	19.2785	16.6802
MGST1	148.637	111.246	100.061	131.251
CRY1	8.86541	7.66499	6.83923	7.71119
NFIX	11.0867	9.58287	10.0828	9.97462
ST3GAL1	6.16191	5.77009	5.81044	6.78613
MMP25	0.65459	0.868626	1.06715	0.990873
IL32	4.99347	4.86913	5.22127	5.43416
PKD1	46.2708	48.8013	53.7011	43.8927
MAPK8IP2	5.3046	8.26673	9.26363	7.72182
MED24	65.0344	66.2417	63.6467	54.1468
RHOBTB2	5.34673	6.15594	6.42321	6.25982
HEATR5B	3.00808	2.84334	3.13738	3.33903
SEC62	35.3965	27.2158	32.0485	36.5889
RPS20	2071.91	1937.86	1655.74	1862.63
CSDE1	216.797	206.262	195.281	197.707

UBE3C	45.6733	43.826	41.0471	45.0811
REV3L	5.74483	7.19492	7.05538	7.1095
FAM76A	3.31846	2.63586	2.6022	3.14597
TRAF3IP3	2.2268	1.80533	1.95569	1.96086
POMT2	29.7903	27.9705	32.1068	29.5289
VTA1	54.2152	53.1143	54.8788	57.0663
MLXIPL	4.57946	5.89479	5.5276	4.71026
BAZ1B	21.6782	17.8161	16.9644	18.0473
RANBP9	34.1888	30.7287	33.6903	33.3323
SPRTN	9.479	7.23038	7.87547	8.18723
METTL13	40.7139	33.4995	35.1064	37.0399
DYRK4	21.2511	23.5585	19.1477	23.1279
ZNF207	136.515	121.303	118.852	122.201
UQCRC1	314.743	310.509	329.658	307.458
STARD3NL	29.0883	27.0656	27.1409	29.6636
CD9	168.505	162.097	165.506	172.22
NCAPD2	84.8218	75.8232	74.4973	75.0834
GIPR	0.814125	1.09511	3.36894	3.34397
PHF7	2.54441	2.03704	2.6468	2.47108
SEMA3G	1.16733	1.70766	1.43764	1.21386
NISCH	21.3619	23.4627	23.3749	21.4525
FUZ	3.17033	3.79245	3.27022	3.59213
IDS	9.37317	9.76967	11.0404	10.5111
PRSS3	52.01	48.6139	47.1937	46.7138
ZNF200	5.06874	5.03221	4.84748	4.98938
LRRC23	2.52435	2.92541	2.61508	2.34687
HFE	7.1689	5.85021	5.83753	5.71899
SCMH1	15.1221	12.9515	14.4508	13.1114
FYN	6.29538	6.68687	5.70296	4.16654

HIVEP2	1.14835	1.55247	0.997638	1.081
TCEB3	17.9608	13.604	15.3833	16.0274
LYPLA2	118.348	111.387	128.607	122.583
CLCN6	2.16853	2.21975	2.58245	2.53924
MRC2	2.44537	3.41901	2.68943	2.15796
NME2	1069.52	1075.26	993.035	1082.48
TSPAN9	10.456	10.5178	11.2469	11.8425
BTBD7	7.39268	6.51012	6.73074	7.82156
APBA3	17.7499	16.5361	17.1252	16.5727
MKS1	19.783	18.2407	17.7116	17.8732
ABHD5	14.1574	10.0789	13.9577	15.3995
AKAP8L	44.6257	40.1753	43.8905	40.653
MBTD1	8.00883	8.89737	8.68198	9.53062
UTP18	83.6008	70.1082	67.9313	72.9573
RNF216	20.6527	18.412	18.4735	19.5056
TTC19	31.3105	29.5696	29.9551	32.9442
PTBP1	438.308	319.546	291.195	294.448
DPF1	7.15209	4.95539	6.87076	5.29566
SYT7	11.7188	13.1529	13.9945	12.585
LARS2	17.8457	15.4773	17.2283	16.894
PIK3C2A	8.21447	8.08938	7.65407	8.60874
PLAUR	29.2312	25.6582	27.0795	25.3921
ANLN	56.4123	45.4283	46.132	52.5823
WIZ	17.5963	14.9635	14.6905	13.7697
RABGAP1	6.41129	6.55716	7.08481	8.0364
QPCTL	9.22851	9.37675	8.17933	8.7547
PPP5C	91.4704	83.1442	90.9452	90.2525
CEP68	7.06696	6.93726	6.40712	6.74429
MAP4K3	10.6625	7.62705	8.30104	8.48265

TMEM159	16.8893	16.2411	15.8293	17.3462
BRCA1	19.1075	16.4049	16.8302	18.1672
ERCC1	94.7363	95.2755	95.0998	89.5428
CD22	1.85638	2.68112	2.1995	1.84138
SEMA3B	2.3186	3.9326	4.00507	3.01792
MBTPS2	8.66319	8.15854	8.72877	8.94212
PRICKLE3	7.84033	7.62226	9.01476	8.77404
EXTL3	36.4924	29.8336	37.4644	34.9059
ELOVL5	42.0678	32.1247	28.5542	34.2955
KDM5D	0.971549	0.855488	1.10776	0.981819
CALCOCO1	7.62704	11.8113	9.76921	9.08765
UBR7	25.8845	19.8721	20.0109	23.197
MAP4K5	11.6249	10.9827	11.8795	12.4215
PSMC4	167.217	141.31	153.01	144.602
MAN2B2	9.91939	9.85996	11.2108	10.4029
SLC25A39	314.508	315.302	318.277	295.928
MVP	33.2012	45.3053	48.2135	41.5641
NUB1	20.6966	19.7207	19.4038	22.3048
PGM3	26.8301	25.6787	27.8511	33.6229
RWDD2A	2.25018	2.39207	2.47923	2.31121
CLK1	17.4068	15.2693	14.1304	16.2867
POLR3B	6.37361	6.24601	6.47006	6.83704
ANGEL1	11.7714	8.36251	12.1067	11.1602
RNF14	27.0402	24.8425	24.4708	26.1871
DNASE1L1	3.62514	3.46794	4.361	2.80898
DDX11	53.3268	43.8133	47.8573	47.7932
HEBP1	27.067	24.5771	27.6839	26.8483
GPRC5A	131.941	114.808	133.18	139.136
MAMLD1	1.07062	1.16553	1.385	1.3359

TACC3	81.3704	65.6035	72.5808	68.2487
UFL1	7.43492	7.48795	7.60112	8.3371
POLA2	34.0048	31.7684	34.3074	38.8727
ZC3H3	15.3518	14.0667	15.4842	14.0877
CAPN1	136.761	140.308	160.068	148.576
MDH1	241.156	250.896	256.107	247.313
SLC30A9	27.9953	28.7884	27.6556	31.3441
MTMR11	2.01344	2.06772	1.77041	1.9341
COX15	48.9498	49.6992	49.0645	51.6701
CCDC88C	5.58495	5.77683	5.22197	5.42785
YAF2	13.2681	12.0541	10.8771	9.85685
ZMYND11	12.7442	14.0362	13.041	13.0441
DPEP1	1.66644	1.79461	2.51906	1.67594
BID	64.6535	62.4198	66.3935	59.9354
MATR3	189.848	153.62	174.733	178.676
XYLT2	17.1347	18.4623	18.339	16.9341
RGPD5	5.16476	8.28894	7.4928	8.67797
NUDCD3	44.0813	31.0853	40.1048	39.3481
ISL1	0.794285	0.908499	0.774227	1.01371
CHDH	5.64087	5.72921	7.31251	5.97065
GLT8D1	35.4132	35.2947	33.4557	36.2939
ATP2C1	45.7053	44.2854	45.6223	47.2288
SRCIN1	2.21758	2.15387	2.32752	2.05239
SLC38A5	58.6944	48.119	50.8316	47.6168
RALBP1	28.5178	26.8658	27.0964	27.7165
RUFY3	9.06757	7.40731	8.22471	7.70411
WWTR1	21.181	16.2517	17.3768	18.7494
AGPS	27.5614	22.6495	21.9749	25.06
CXorf56	23.1021	18.7284	18.1335	18.5574

TTC27	15.2159	13.2092	13.4181	14.0137
PHLDB1	10.7155	12.1611	12.3118	10.078
CYP24A1	96.6664	69.4058	85.9939	93.1568
PRDM11	4.08008	4.09159	4.15888	3.7687
ZRANB1	12.085	9.70795	10.2253	12.3517
NCDN	21.6921	20.6635	24.4393	22.8224
GPR124	1.25405	2.0286	2.02141	1.78545
ZFP64	6.44664	6.6208	7.25427	7.31539
MNAT1	40.7066	36.2808	34.2118	38.2801
SAMD4A	4.07205	2.88253	3.04912	3.38835
RUNX3	14.7289	13.6282	14.5898	14.6771
MRE11A	4.24305	4.6885	4.0203	4.42394
PLEKHB1	6.95825	7.91699	8.65054	7.63392
SERPINB1	15.7157	22.8184	24.5529	26.5948
SPAST	5.05934	4.23211	4.74876	5.27991
OSBPL5	13.1906	18.3034	20.4562	16.0531
AQR	15.8988	15.9071	15.1091	15.4827
CPS1	7.95456	9.23615	8.82919	7.98977
FHL1	7.27709	6.26492	6.80385	7.2599
RTFDC1	62.3376	61.3823	59.6735	61.1053
NLRP2	6.29218	5.7253	5.00416	4.62641
SLC45A4	8.64171	8.29453	9.70341	9.7414
RNF10	115.877	115.519	125.031	111.084
ZNF839	7.58883	7.65843	7.96392	7.52921
ZDHHC6	60.2441	64.2711	57.8052	67.7911
GRAMD1B	1.0398	1.16384	1.14112	0.991487
RNH1	92.6469	77.2726	90.6539	84.2002
NDUFS1	43.0585	46.0593	48.3364	49.1048
RB1CC1	10.0453	9.55612	9.67791	9.55098

ERP44	6.72348	5.7118	5.74796	6.13235
ALAS1	55.6293	44.7625	52.1911	49.3439
AKAP11	4.54111	4.89797	4.9381	5.42149
GLRX2	25.8069	24.0355	21.5516	24.391
SNAPC1	8.02342	7.06589	6.34859	7.49414
DERA	23.7066	28.0721	26.6382	30.6126
STRAP	212.092	182.19	184.379	200.613
ABCC2	9.31538	11.6118	12.4023	11.7858
DEF6	22.0541	22.1099	24.5145	23.5122
PLEKHO1	3.09898	2.70423	2.77164	2.71926
GCLM	49.7694	35.4992	34.3892	37.4991
UBR2	5.71153	6.15114	6.14182	6.15317
EHD2	29.5276	32.9244	34.7312	32.7189
DEPDC1	22.512	19.2141	16.5744	20.9559
CCDC28A	4.56276	5.11844	4.03673	4.52485
RRAGD	5.69718	5.04189	5.28219	5.92404
HSF2	16.3582	14.0524	15.2005	15.4937
PHF20	6.71732	6.24731	4.9574	4.2141
HSD17B6	1.92693	1.57861	2.09437	1.94923
NR1H3	15.0499	15.8479	15.5673	14.993
TYMP	19.7779	22.3995	22.9618	20.0931
NCAPH2	75.4247	66.8684	70.3558	63.5282
TOMM34	61.4149	40.6879	52.2319	54.6786
SEC63	29.3645	28.1565	23.5117	27.2492
KPNA6	18.6286	17.4969	17.4008	18.3588
VIM	1.75467	1.59395	1.30346	1.08458
RTEL1-TNFRSF6B	4.97641	4.72814	5.95011	4.46521
FAS	8.25155	14.2423	11.0549	10.5335
RNASET2	18.7512	17.6874	18.9012	19.3296

CD44	223.618	210.646	209.645	211.509
KCNG1	12.755	22.6545	18.9819	16.6
AGPAT4	3.91414	3.77473	4.66142	4.43563
BTN3A1	1.07599	1.6226	1.30252	1.61493
MIPEP	19.4945	16.594	19.0542	20.0288
PRKCH	6.34726	7.85935	7.47385	7.29859
IFNGR1	13.9687	13.8311	12.203	12.6876
B4GALT7	17.5503	20.3419	21.0157	19.2366
SH2D2A	1.76937	1.89231	2.10112	1.74662
VRK2	15.4396	15.1618	14.0433	15.4051
VEZT	18.2971	18.8056	18.5358	18.8585
BRD9	30.0094	28.8171	31.2778	29.192
SNX1	46.6919	43.7332	55.2015	52.6112
TBPL1	35.0814	27.9392	31.5862	38.1368
ARNTL2	8.69896	7.67657	6.34774	7.10235
BCLAF1	98.9785	81.0189	87.7163	99.3864
SLC39A9	36.1712	29.8925	30.9159	35.4184
TFB1M	21.1728	20.0923	18.0971	20.1594
RABEP1	18.9774	16.2608	17.9776	19.6044
HMGB3	44.3855	41.2774	37.1818	43.3204
NUP160	30.6191	30.3993	29.3056	30.3328
BAK1	28.9304	18.7089	24.1184	24.2164
GRN	73.2847	97.8118	92.7361	77.999
FAM13B	3.52183	3.03377	2.95539	2.93593
CENPQ	9.40427	5.51986	6.06481	8.20159
SARS	91.2738	112.043	88.5501	87.6005
RANBP3	70.7813	56.4902	63.0782	63.7219
ARID4A	2.51221	1.70745	1.52794	1.31043
TSSC1	29.2096	29.7785	33.0749	31.6777

PNPLA6	46.6541	50.5685	50.857	44.3947
IFT88	5.27091	5.44054	5.76834	5.8976
ALG1	56.0801	46.376	46.3458	42.0909
ZCCHC8	10.668	10.4508	9.03521	9.6403
ABCF2	89.6477	82.6588	81.2109	81.2162
CHPF2	9.4462	9.02633	10.2186	9.12559
FUT8	13.0086	12.3812	14.3003	13.7712
UBA6	20.6643	17.2657	20.3592	20.1152
ATP6V0A1	20.1541	22.8848	25.0627	22.308
PIAS1	12.5057	9.48149	10.0648	11.1561
SLC4A7	10.9964	10.0977	11.24	11.1394
UHRF1	75.0066	57.4996	64.0494	67.497
MAP2K3	92.6011	66.3879	83.6372	83.7799
TMSB10	1901.79	1891.55	1760.66	1997.71
ASTE1	4.73031	2.94997	3.3261	3.52814
RNF19A	8.47097	10.0907	12.1362	11.8607
PEX3	20.6436	16.846	19.4838	21.7489
GABARAPL2	63.3083	64.0786	60.9623	66.7278
SH3YL1	9.70185	11.3616	10.5496	9.51225
FAM136A	84.1833	62.7363	51.4041	56.2502
VCL	37.3941	36.498	37.0699	36.8767
DEPDC1B	24.0553	21.5765	19.6195	23.044
DAPK2	1.2782	1.9025	1.49698	1.59836
NSMAF	20.3965	20.9204	21.3826	22.326
ADSS	29.3016	30.4004	30.0657	34.1827
TIMP2	32.1455	33.4905	34.4054	35.7749
RFC1	45.3387	29.4574	34.2437	41.3346
TBC1D23	6.34595	7.04909	6.95204	7.57452
CUL3	47.925	47.1299	43.8054	52.1609

MYOM2	1.13457	3.1247	1.89132	1.47201
ZZZ3	17.4125	15.521	15.3349	17.4408
USP2	2.78099	2.40356	2.26346	2.56472
TUBG2	7.92629	8.56503	10.0405	8.97261
RPL26L1	127.34	113.38	111.103	110.479
NSUN2	86.1136	74.8826	81.9562	86.3605
FBXO42	12.0272	11.4158	11.6145	11.3625
MFAP3	9.14209	7.905	7.61227	8.11791
MRI1	9.19956	7.48989	8.04807	6.85682
METTL1	63.8047	57.144	60.4602	59.4541
AGA	3.9225	4.3995	4.02658	4.55643
PI4K2B	12.1381	13.3018	12.4645	13.5038
BOD1L1	4.96484	4.97422	5.9974	5.63402
MAT2B	35.6055	33.5035	35.316	38.5821
EDC4	94.9018	90.4102	90.5911	86.0713
TRIO	21.0529	16.7537	17.7408	17.7918
VCAN	27.0057	20.7425	24.0056	23.6022
CLEC16A	8.6214	8.76977	10.3667	9.36356
CDH1	22.3531	16.4536	25.8404	22.4918
SKIV2L2	69.5349	54.2972	52.5581	62.0987
ZFYVE16	9.26315	10.6928	10.6915	11.0749
FAM65A	35.6133	26.8568	21.9121	22.6636
RAI14	27.8824	27.328	22.5382	23.2205
PNKP	21.2172	24.4098	23.4974	22.4788
PHLPP2	6.48973	5.31228	5.42599	5.8887
SPDL1	22.2261	15.8105	16.6954	17.7484
STAU2	13.5374	14.8348	13.8182	14.4118
PQLC2	8.79044	10.8619	11.1919	9.91274
CTNS	8.16684	8.78039	8.27884	8.41964

RTN4R	15.384	13.8454	15.5578	13.4492
PHF23	44.0013	43.9649	40.6067	44.6439
INPP4A	5.60956	6.15395	6.18126	6.45853
PSMA4	347.384	299.086	304.238	331.163
LSG1	35.7654	31.04	35.6168	35.2286
PARP3	5.67008	8.32151	9.54203	7.76072
THAP3	14.6629	13.8859	14.0439	12.7189
TDP1	15.4168	12.7192	13.1242	13.6555
AIFM2	29.1122	28.0657	28.8408	27.5048
SPATA7	1.92687	2.0479	1.90958	2.38383
MED17	52.8708	51.0455	47.8768	52.9517
RETSAT	23.3993	28.393	25.9206	24.349
CAPG	108.672	122.109	119.376	116.888
AP2S1	251.842	248.337	271.903	264.001
DCUN1D1	18.3955	16.5077	16.4953	18.8751
PHF15	10.8864	9.53136	9.65988	10.6925
ZIC2	18.9	17.3362	18.7632	18.7935
TRIT1	20.6343	17.4639	18.0636	21.9409
CUL7	9.14056	10.1	11.9309	10.6172
CTNNA1	157.569	152.921	156.694	157.847
PHKA2	10.5764	11.3543	12.9469	11.8429
CNTLN	1.46568	1.73425	1.41144	1.59834
HSPA5	246.135	237.744	251.715	252.622
DSG2	39.9262	40.2952	39.9799	42.5024
GEMIN8	9.56449	9.5245	8.48999	8.17262
OFD1	4.19425	3.72267	3.8906	3.75461
WDR37	3.71485	4.34589	4.29131	4.07803
YTHDC2	9.7145	9.09106	9.11527	10.0471
CTPS2	5.13131	4.88871	5.23767	5.61028

ATP6V1H	32.2265	22.4552	26.5687	24.4472
POLR2B	84.5578	70.219	74.1326	86.3914
FAM214A	0.864387	1.2137	1.00942	0.77811
ARAP2	2.48258	1.8044	1.62023	2.36978
TPR	49.1437	46.7601	48.1541	48.6066
CP	0.934546	1.24774	0.945842	1.1722
KIAA0556	6.87077	6.43775	6.9952	5.73068
DTNBP1	17.0053	16.9016	16.438	16.9708
C12orf4	8.30684	6.05297	6.39958	7.42848
SCML1	3.74629	3.56933	3.83683	4.20198
WWC3	5.07475	4.95657	3.95782	4.66119
MAP4	64.5047	61.446	63.921	64.1739
GOPC	11.5613	11.1626	11.5536	12.6415
USP28	9.98336	9.18134	10.7072	10.1306
TSPAN17	39.6618	33.2467	36.1141	35.9373
NOP16	113.262	89.3124	94.113	94.5334
CC2D2A	6.88451	7.86903	6.70985	7.63032
RRM2B	17.5245	25.7467	17.9981	22.1558
ZNF800	7.74046	6.7428	7.10042	7.90241
SNX29	1.19825	1.0202	1.24915	1.13162
MRPS10	39.4855	41.5236	38.3493	44.9433
RSF1	11.9207	9.87591	8.3709	8.61546
VPS13D	5.96709	6.60188	6.80944	7.11533
FAM120A	60.1498	58.658	59.9283	56.9133
R3HDM1	30.5194	27.0994	28.7287	29.6556
COL9A2	2.00919	2.10377	2.03145	2.04639
KITLG	198.255	193.476	175.196	205.339
ERCC8	11.5629	10.2845	9.4373	11.0972
H6PD	1.61165	3.10262	3.45004	2.97489

VAMP3	62.449	59.2393	55.0842	54.7572
PER3	1.09211	1.40296	1.00487	1.20302
UTS2	1.43296	2.20638	1.90653	2.16209
EPN3	4.11437	7.5044	7.77821	6.75548
LTBP1	1.8252	1.60087	1.65756	1.68704
RCN1	77.0913	82.2098	68.6764	78.0608
RFC2	45.9015	37.7204	36.4889	37.2203
ARID1B	7.61321	7.04598	7.60707	7.26174
CLPTM1L	233.465	182.249	197.43	195.435
NEDD4L	23.0747	18.3607	18.5449	21.575
HEXB	90.3729	84.4142	85.6604	85.2823
PTCD2	6.81009	7.96307	8.08312	7.97437
JKAMP	59.338	48.995	44.7326	49.2057
ARHGEF5	8.80295	8.25042	8.00463	8.47191
NFE2L3	25.9115	22.5477	23.8739	27.0079
MCUR1	25.1594	20.9081	20.0525	22.0825
LIMA1	39.6346	39.603	42.5283	45.3077
LETMD1	40.5926	37.2672	41.5301	39.3145
LAMC3	2.71508	3.53058	3.40544	3.32182
MAPK9	28.365	21.5751	20.8508	23.224
BCAR1	26.275	25.3416	30.771	26.4174
FAM160A2	14.1249	13.877	15.003	14.857
HERPUD1	50.5044	39.9117	45.5764	44.9201
HOMER3	30.7264	30.5134	31.2369	29.9009
RAD51	31.1257	23.5553	25.8348	25.2781
POLQ	6.73903	4.6822	4.35806	4.74614
PIK3CB	20.5751	19.3037	21.1378	22.2012
CYBA	230.929	237.896	257.085	232.592
THOC3	81.8822	66.1108	62.4575	61.0905

HEBP2	33.1678	33.8001	32.9781	33.4696
MPHOSPH9	14.4075	12.9786	12.3157	14.4672
PLEKHA5	4.43679	4.62314	4.52157	4.633
PRSS8	16.9015	24.46	25.1528	21.4203
SIKE1	38.3446	34.2205	33.7027	36.7077
RRP12	36.1716	32.3234	33.0767	32.3309
FNIP2	1.68631	1.7865	1.24884	1.44683
MSMO1	19.4954	15.6239	13.4194	17.0828
TTC17	17.0478	16.1651	16.3056	15.2911
FOXN3	4.71322	4.58552	4.1139	3.82504
AKR7A2	61.6697	65.1898	65.4762	61.4836
MRTO4	119.352	92.1024	102.007	98.3166
USE1	23.3649	23.3688	23.9128	23.6035
LAMA3	1.36758	1.7696	1.73226	2.01392
AP5M1	9.80971	12.4523	11.8339	11.7957
ANAPC4	17.3915	15.5427	17.0026	18.5627
KCNQ1	1.95532	1.43691	1.76738	1.61464
TRAPPC3	85.0428	68.8335	74.2691	82.433
THRAP3	69.1914	61.7772	63.2669	74.3175
PHPT1	91.2231	112.387	109.922	104.143
ENTPD2	8.42696	8.51418	9.82763	8.8836
ARID4B	4.70988	4.99298	4.96545	6.59366
OPN3	1.6935	1.87476	2.17712	1.80454
SDCCAG8	36.0462	27.6902	26.2072	28.1416
HHAT	0.772593	1.9345	1.68027	1.25442
KIF1B	7.98862	7.44117	7.3997	7.76626
FOXC1	11.8567	10.882	11.587	12.255
TBC1D22A	11.0008	11.3571	12.1978	11.9123
SYNE2	4.32842	4.18153	4.20648	4.17808

PLEKHH1	4.64346	7.24473	8.3333	6.89291
ATP9A	4.06516	4.57293	5.01973	5.21662
FAM168A	8.46432	7.66689	8.91809	8.70481
RELT	9.17026	6.97483	7.24847	7.3011
NOP58	123.922	95.517	95.3941	105.525
SZRD1	104.772	93.0124	91.7333	93.549
KCNH2	6.52045	5.54662	6.85159	6.12705
CUL1	34.8729	29.4454	30.5575	33.9721
FAM114A2	10.2503	10.4354	8.93482	9.95844
CYFIP2	21.4719	33.2216	31.3653	28.3508
TAB2	19.5491	17.5609	19.1747	20.4604
GINM1	13.9591	14.9112	14.0818	14.9021
EIF2AK2	16.5799	14.8932	12.9806	14.8323
USP36	53.6712	46.96	48.5551	45.8853
KMT2C	3.05862	3.64052	3.19478	3.26858
MCOLN3	4.66333	4.51926	4.82601	4.93847
PUM2	33.9392	26.1988	28.3044	31.2672
MRPL43	130.825	128.11	130.768	129.455
ITIH4	1.12238	0.71562	0.81755	0.479123
C4orf27	31.7194	26.3982	25.8724	26.9794
ZFR	47.7708	43.4692	41.8992	44.8273
ZNF280C	3.94352	3.55734	3.94574	4.07742
RC3H2	13.8058	13.3156	10.5613	12.0663
PCGF2	38.3963	32.9828	35.7595	34.7143
IL17RB	7.85647	5.59242	5.82053	6.59455
TRAF3IP2	5.75029	5.99273	6.27697	6.54781
DCBLD2	169.59	154.92	168.913	181.607
SOAT1	20.5084	18.9767	19.0459	21.1016
PKP2	11.5213	8.53045	9.27129	9.80053

GDI2	101.101	87.5957	84.8694	91.5248
ATG5	17.3619	17.623	15.7139	18.785
TMCC3	1.74215	1.73849	1.646	1.79403
PITHD1	61.0068	44.8728	47.6823	48.0498
MTA3	18.0653	18.4363	17.6098	18.145
USP13	9.15828	7.5519	8.32602	9.73251
ATP11B	11.8444	10.3041	12.1672	13.4971
LAMC2	7.60721	7.27386	7.88175	7.6688
CDK14	2.5444	2.25046	2.2086	2.60278
SEC61A1	157.068	151.074	161.758	150.185
PPP1R12A	27.021	22.9947	22.9873	23.4607
CROCC	11.0928	11.4339	11.7846	10.6685
POLR3E	48.3647	41.3689	40.167	39.7047
ATP2B4	8.786	8.16054	7.32743	8.74812
ZC3H11A	31.7844	30.6258	30.8261	32.8718
RIOK2	41.163	33.8294	33.9222	38.6226
YIPF1	11.1968	12.9802	12.3586	12.844
NDC1	47.1778	36.0241	37.1137	43.7277
DGKG	4.57545	5.88369	5.54556	5.72296
FLYWCH1	28.9734	33.6461	35.6182	30.3147
UNKL	15.7851	14.6262	15.0676	16.9162
TBXAS1	1.03237	1.06558	0.981559	1.13014
PARP12	8.23324	8.82539	10.1069	10.8704
ALDH18A1	56.0766	43.9167	48.7	51.3169
TARBP1	3.16522	3.32756	3.21035	3.40877
PET112	21.5771	20.0743	19.7112	20.1565
CDK17	6.67375	7.93304	6.99607	8.18354
DNAJC25	20.6286	17.2511	16.5583	19.3296
SLC2A3	6.55127	4.65802	4.96014	5.79258

PSD	6.54779	7.01805	7.98285	7.09118
CTDP1	16.2977	15.298	16.6195	15.6779
YBX3	233.005	288.637	276.429	252.63
STYK1	1.97616	2.08532	2.36505	2.63238
WNK1	22.7942	20.8593	21.8261	20.625
CCAR1	62.2672	57.0335	56.1324	53.2473
OGFR	51.101	44.7243	47.1448	45.948
PIGV	6.25667	9.03013	9.06727	8.32809
PTPRU	17.6085	19.2543	21.7519	19.5449
SNRNP40	100.055	84.3408	84.912	90.201
QSER1	14.2067	11.3294	12.7758	11.6207
MPC1	15.3734	13.6396	15.1541	17.2017
ACAA1	38.7169	37.1618	37.9703	34.1655
HDAC7	16.5603	13.0063	14.1167	13.492
LZTS1	1.58979	1.5749	1.56258	1.65697
SPAG4	0.822082	1.44113	1.21157	1.0533
NCKAP1	68.6628	52.5958	40.8883	44.3277
MRPS35	56.084	48.831	47.0013	53.1897
SFSWAP	26.3684	21.5507	24.3608	22.9422
TNK2	14.2012	13.123	17.9587	14.3475
MON2	6.65998	8.28764	8.18776	7.29236
CDH3	46.2054	37.7494	52.8382	48.504
GPBP1	31.149	29.3222	25.7259	28.8737
DGAT2	1.87438	3.04337	3.29127	2.3983
CS	178.703	160.218	180.401	177.085
LTK	2.17564	3.15528	3.23709	3.10144
MRPS24	177.228	160.356	152.576	153.92
ELMO2	9.7894	10.4671	10.4851	9.16942
WAPAL	35.9357	34.5002	36.6203	32.489

VMP1	118.866	106.014	95.6114	103.41
APPBP2	12.8747	11.5899	10.5696	11.3176
POLD1	76.8969	67.9692	69.3342	63.1846
EIF4B	239.893	222.768	239.504	244.957
SLC6A16	0.925599	1.37376	1.44879	1.39049
GLTSCR1	3.49144	3.55788	3.26206	2.8916
SPHK2	9.89351	12.2265	13.6115	10.3711
RPL18	1577.76	1574.4	1503.12	1508.85
CA11	15.5518	15.1963	15.5586	14.4882
ISOC2	43.5162	42.0307	43.906	42.467
U2AF2	265.9	240.693	253.984	252.032
EPN1	66.9964	64.9277	75.2751	65.1567
MED29	20.1148	19.3575	20.7327	17.1128
AHRR	5.89467	5.95412	6.66778	5.87145
ZNF275	2.25435	2.06809	1.66695	1.75113
MTMR1	6.8596	5.63509	6.10063	6.33736
GPC1	101.893	104.518	116.375	106.875
ADCK1	4.37467	5.8314	6.24092	5.55619
HAGH	22.4412	25.1987	29.1387	24.9236
RNF4	41.8651	38.1518	39.8086	40.3383
CASP8	17.9905	17.566	18.6746	21.5598
LIMCH1	2.01513	1.89398	1.7795	1.49805
ASUN	50.907	42.7387	42.8522	47.5072
TM7SF3	58.9229	55.0303	71.7431	66.9708
SPA17	10.2705	8.9447	7.61959	8.65874
ST3GAL6	5.09948	4.24539	4.06645	5.21385
ATP2C2	2.53161	3.10259	3.36362	3.63437
NGFR	2.09214	2.52429	2.58949	2.25215
CDON	3.72944	3.45291	3.95762	4.37049

TAF2	33.2189	28.3441	29.9659	30.3519
HIPK2	6.52159	6.81472	6.96358	7.45201
TNPO3	35.2259	28.0238	29.845	31.2581
MEF2BNB-MEF2B	9.2966	9.4328	8.84471	8.55362
RFXANK	34.1424	40.3789	43.6038	38.8244
TMEM161A	60.9203	62.2772	65.4228	56.0806
LPAR2	26.3475	26.1849	27.1348	24.3596
CTSA	76.1453	81.8024	78.445	74.7131
SUGP2	21.7262	20.2788	19.0335	20.4475
SLC12A2	16.7318	14.2262	16.3225	16.763
SNX24	6.73688	7.19873	6.27411	7.37124
CNN2	205.183	144.017	187.993	182.139
ABCA7	32.1001	32.224	36.7034	31.0073
DDX20	12.9045	12.4681	12.2388	13.4047
BTBD1	59.7883	56.6577	61.8429	63.7612
FAR2	3.49592	3.73653	3.74695	3.91187
SBNO2	22.4357	23.8299	26.3059	22.3971
PMS1	12.0135	13.998	12.3468	13.7388
HMG20B	77.8957	81.1323	82.6481	76.0946
TAF11	24.7687	18.7573	16.3139	18.0118
ANKS1A	8.30076	7.56559	8.48695	8.31963
AP3D1	80.23	79.7591	81.3337	77.4962
ZNF76	17.8689	20.7456	22.5755	19.0552
SLC9A3R2	33.2591	33.331	32.4006	30.9071
NTHL1	37.8777	34.631	37.2157	33.9577
UHRF1BP1	6.06127	5.79529	5.53602	5.853
GNAI3	57.4769	52.9009	50.97	57.8152
IPO5	121.476	111.809	115.92	122.167
OAT	112.799	102.505	100.322	109.1

WDR3	64.7761	54.7018	54.5697	59.7466
PKN2	11.9147	13.5765	13.8982	15.249
WDR18	109.097	113.519	114.587	102.605
TRAM2	8.91403	8.33271	8.52304	9.34176
NTN1	2.45191	2.62949	2.86564	2.60229
MCM10	22.1401	14.6564	15.6041	18.3416
DGKA	25.3965	34.184	32.5751	26.5645
ERBB3	9.34216	8.61741	7.38475	7.01082
ANKRD44	3.09181	3.13671	1.70882	2.13444
KARS	176.19	153.332	158.5	165.707
ADAT1	23.4479	25.5955	21.3428	22.8104
PDIA5	22.8474	19.0231	22.5579	22.6443
TBC1D22B	5.85502	5.68344	5.471	5.47003
NDUFB4	196.929	176.57	178.005	179.978
SPEN	18.3092	18.8901	18.4399	17.4073
ZC3H15	68.1313	53.899	51.6165	57.8318
MAP2K4	24.8943	21.7381	21.3867	23.197
TMEM206	24.7627	21.2195	21.309	21.7236
SLK	12.4708	10.7069	10.9579	12.7194
CYB5R4	13.6131	14.2531	13.6571	14.5332
GSTO2	84.7747	86.2826	94.4017	100.027
SEC61A2	5.6746	4.96442	5.26976	6.30846
TLE2	15.6133	16.3466	18.6928	18.1431
ASB1	19.0557	16.7579	19.4798	18.4112
FAM107B	12.5185	14.9361	11.198	11.4347
ME1	38.9094	33.9009	31.5077	36.3197
TBC1D1	49.3325	42.1375	40.9209	45.888
CDK13	7.42401	6.7789	6.99005	7.63561
MTHFD2	274.536	151.893	145.294	171.974

SLC9A7	5.0388	3.90677	4.68886	4.15978
FOXJ2	7.01652	6.53268	7.08449	7.1765
YBX1	915.042	842.055	844.776	876.339
PDE4A	17.2388	14.6758	16.8212	16.3011
PPP2R5A	17.818	15.9896	16.3517	17.2927
ELAVL1	80.9389	69.6124	72.7293	78.2088
DIP2B	10.8125	9.70203	10.7176	11.1084
SMARCD1	91.4734	86.0273	93.4042	90.8651
KDM4A	10.0221	11.0559	11.5434	11.4096
NFYC	43.9817	42.3585	50.273	44.5936
SLC9A3	1.04373	1.66576	2.06737	1.57166
NGEF	2.4032	4.35445	4.16729	4.79741
ASPM	6.35493	5.81581	5.40585	5.67322
ELOVL1	123.675	111.373	126.503	121.579
ZNRD1	12.8942	11.2757	9.60404	8.42371
ZBTB11	6.76803	6.14854	6.42528	6.65461
ATXN3	13.1103	13.7339	15.2852	14.2023
GOLGA5	10.1134	11.1196	10.6389	11.4005
FGFR2	5.69273	4.41398	3.74807	4.19013
LRRC40	9.00062	7.57929	8.76359	10.0361
ISOC1	39.5144	34.3148	35.5157	37.866
EML1	7.45954	4.99121	5.44633	4.78965
TRMT11	19.0982	19.7688	19.0465	20.2617
THUMPD1	16.0278	16.7222	15.1718	16.3523
MSANTD3	31.838	19.4654	21.8766	24.9026
KIF26A	5.96547	4.95809	5.88131	5.37791
ATG2B	1.50735	2.39745	1.90279	2.16006
ARFGEF1	8.90455	10.4228	9.19903	10.8261
ZFAT	2.25242	2.42709	2.47213	2.27375

MTFR1	24.1656	19.9219	21.0371	24.9181
FECH	23.7887	31.2939	31.3263	31.4577
MYO9A	3.52651	2.88826	3.08134	2.715
DDX3Y	3.12394	3.13217	3.36714	3.3654
PFKP	159.527	136.383	138.022	141.381
IDI1	30.788	19.6806	21.3478	23.8868
SP100	16.0765	18.3508	19.8815	20.5179
KLF6	25.3692	22.3199	23.5595	24.845
PPAP2A	8.83599	9.67985	8.97516	9.74534
NEO1	12.4773	16.4687	17.9789	18.0093
TRAM1	88.5761	78.6787	78.4387	84.2593
PHKA1	3.53134	3.38192	3.53121	3.77201
TNFRSF1A	73.9121	56.3384	65.7695	66.6161
CACNB1	4.01304	3.51962	3.68957	3.31969
EVI5	5.17975	4.36296	4.10232	5.13824
STOML1	3.63073	3.38631	3.99572	3.42205
PKM	1143.78	1065.49	1099.65	1123.72
DHX29	12.6099	11.7183	10.8574	12.6557
DNTTIP2	42.3736	32.3776	31.694	37.7962
METTL22	10.8382	14.7778	17.1607	13.7599
TP53BP1	17.2229	19.195	19.4699	17.8817
RRP15	27.3867	19.229	21.6267	24.1156
RHOA	398.307	341.118	337.06	350.84
DHX8	23.058	23.8892	24.5772	28.9881
PMS2P4	3.15426	3.7178	3.04304	3.28649
PRKCZ	19.3711	21.572	21.1682	20.7766
IARS2	67.8159	66.2678	69.6675	73.9363
SYT1	7.68748	8.15109	7.60052	8.48869
NAV3	4.00971	4.29937	3.60806	4.1535

IDH3G	39.1669	40.3202	38.2336	38.8168
ROGDI	13.2872	12.9539	14.0325	13.7752
ROCK1	11.3042	10.3771	11.4733	12.8535
CBFB	87.2085	75.0065	74.4919	80.567
PDK3	3.49547	3.23872	3.17111	3.44845
HYAL2	29.9049	26.9095	28.4263	24.5799
HDAC4	3.65052	4.58963	4.0805	3.30536
RASSF1	23.0982	19.2623	20.9596	20.3934
FGFR3	13.9702	13.8809	16.7493	14.0634
IFI35	5.92027	8.02563	8.88815	7.38255
HEATR6	16.3263	16.6751	15.9386	15.2594
COASY	66.4502	63.5322	64.5259	67.1723
PLEKHH3	20.5547	21.033	21.6378	19.9318
MEF2A	8.56586	7.00778	7.47638	7.75628
OTUD5	11.692	11.2141	12.2307	12.1828
TFE3	3.20999	3.10899	3.73342	3.45511
TBC1D25	2.99545	2.69338	2.93502	2.63219
ACSL4	18.2406	18.2047	19.0488	19.2115
INPP5A	12.2344	9.62119	10.0874	11.0588
GPKOW	7.80394	6.92464	6.93872	7.18069
GRIPAP1	7.18064	8.39445	7.98945	7.67595
FTSJ1	29.2895	24.9008	26.6092	25.7483
PRR11	29.4952	27.985	24.9419	26.8256
REEP1	3.22503	3.00343	2.64379	3.15282
ATP11A	16.2268	19.0806	21.9941	19.3201
POLR1A	19.2395	19.8874	23.5147	20.604
LAPTM4A	81.9316	82.0106	84.0909	96.2564
TTC7A	14.2439	12.5312	12.5041	12.5802
IP6K2	48.1677	55.0228	53.1371	50.1208

SRBD1	10.2488	8.23356	8.6628	9.58332
CYFIP1	61.3139	60.4362	63.8471	58.9429
KIF2A	14.5813	12.576	11.1973	13.4674
RASGRP2	5.30452	4.95004	6.04905	5.50656
PSME4	35.4328	30.5992	33.8681	35.1785
IFT80	7.71648	8.14229	6.83996	8.05251
SIRT2	9.92176	8.01437	8.69017	8.80533
ERLEC1	16.1544	16.5269	17.2328	19.2185
PPP2R5B	4.75532	4.46622	5.16266	4.74246
PITX1	33.6709	25.7147	30.5681	28.7783
NUP133	25.6145	22.4972	23.1678	24.1202
NUCKS1	96.8684	84.7333	85.7363	90.2399
VPS35	55.9449	60.1822	58.0909	62.7953
DNAJA2	55.4652	46.3584	49.526	51.9546
BCL3	9.99048	11.0562	13.5322	11.0167
KCNAB2	7.98617	8.70179	9.75626	9.02551
GAL	166.955	140.58	134.207	142.883
FUNDC1	17.1003	14.6772	14.4235	15.5599
MAOB	16.7386	18.7026	18.3578	18.7345
TGFBR3	2.34756	2.00182	2.22731	2.46687
HES2	5.59327	9.16864	7.7221	7.48488
ATP1B3	252.91	201.629	178.117	216.544
NEDD4	16.8174	17.5658	14.2281	17.7934
PIGB	5.16197	4.82014	4.65209	5.06615
MAPK6	33.0798	31.5308	25.9539	30.6721
GNB5	16.7934	16.3066	15.6684	14.874
RAB27A	4.85808	3.17989	4.1088	4.28327
CECR5	72.5414	63.7776	69.9851	69.8195
UFD1L	173.61	144.581	136.951	150.262

LRP6	12.4795	10.283	12.2255	11.4929
PHRF1	23.0177	20.3411	21.5643	20.5482
IKBKAP	26.4909	24.5079	25.7839	26.5951
NUCB2	23.3903	20.7866	21.1501	22.6836
PFN2	181.007	171.541	156.618	171.508
PTPN3	19.1204	20.2854	19.5489	20.2623
SPTB	1.03598	1.26944	1.34533	1.41465
SLC44A1	14.0638	12.9292	13.6048	13.9405
TMEM260	3.15208	4.25301	3.63579	4.21254
SMG6	14.4404	13.827	15.6935	14.7745
EXOC5	22.9383	24.5718	22.97	24.0279
CLTCL1	1.38294	1.81933	2.05119	1.27302
FSTL3	22.8363	21.6772	25.7252	24.3739
DGCR2	22.7363	26.4292	28.5853	26.5325
RNF126	125.078	111.737	117.683	110.923
MNT	4.77034	4.29918	5.84873	5.03376
ZXDC	8.05226	7.83537	8.02644	7.53983
JMJD6	55.9265	36.4127	41.1337	42.0497
POLB	29.1132	28.9691	24.515	30.3381
WIPI1	7.79417	9.48875	11.378	9.36677
GBA2	25.5848	25.3291	30.58	27.7701
NDST1	16.4818	16.3641	18.1598	15.4224
ASNS	47.9538	133.806	83.7199	74.94
AP3M2	14.0598	10.3567	10.0209	11.9757
ST6GALNAC2	21.3369	20.9329	22.0669	22.4453
PABPC1	3635.05	3418.14	3568.21	3291.74
C16orf80	67.1617	52.0414	64.3998	60.4922
CSNK2A2	46.7528	33.1665	32.337	34.5166
PTPN21	3.20688	3.05139	4.0065	4.54355

EIF2B3	18.3556	15.3893	17.2706	17.7442
TCOF1	69.3513	56.9135	56.0304	53.7625
CDC42	181.615	170.331	166.53	189.451
OSBPL3	16.8193	15.226	15.3883	15.9904
RAD18	12.0818	8.81376	9.36773	10.3208
ATP2B1	19.8319	19.2595	18.1376	19.9135
NCK2	13.3877	15.6689	14.6329	14.0701
MAP4K4	35.4167	36.8701	37.5204	37.5769
MGAT4A	1.59152	1.30826	1.13582	1.81345
RPL31	2180.12	2159.86	1965.5	2184.43
WDR1	282.63	271.674	292.481	272.567
SNX13	11.8528	11.7743	10.1371	11.6924
ARHGAP10	12.1648	10.4198	10.6872	11.2863
RPS6KA2	1.58972	1.82925	1.84392	1.60772
ING3	8.69819	8.07224	8.16518	8.63699
VASH1	4.24431	3.82662	4.33824	5.06455
LMCD1	1.67077	1.4018	1.21382	1.44373
WBSCR22	125.869	131.156	125.762	129.262
SEL1L	8.53863	9.5253	8.89825	9.23979
TRIP13	53.9106	45.0313	45.003	47.5171
ATP6AP1	26.1378	25.0377	26.9064	25.4277
TCF3	191.402	175.341	183.351	182.401
TRIB2	5.07107	6.43297	6.12895	6.28474
DAZAP1	234.197	223.429	259.187	240.338
MBD3	52.3163	47.0672	52.4512	47.3457
HLTF	15.0606	14.1381	14.8275	16.3568
FAM50A	19.0913	16.5288	17.5629	17.5296
FAM3A	7.16762	7.95455	9.51639	8.42382
CPSF1	63.5232	60.7504	59.0774	50.9207

CYBRD1	7.20355	7.10796	7.00022	7.33864
PDCD2	119.805	109.279	108.172	119.347
RDH11	42.1871	34.1953	35.7377	39.2169
PRKACA	40.4988	44.854	44.1089	44.3856
LPHN1	32.864	31.2272	35.8396	32.1337
ACTN1	63.887	55.5899	60.3172	62.0694
ZFYVE26	3.92673	3.80669	4.08218	4.29332
EPN2	26.3945	25.6101	25.6367	33.3682
PTPN18	13.2626	13.1022	15.115	13.1582
LIMS2	1.99825	1.92129	1.81553	1.48226
SPEG	15.0679	17.2303	17.9629	16.3145
LNX1	1.68698	1.5498	1.45977	1.52373
ALDH3A2	43.7708	47.9315	49.7127	46.1386
TFRC	128.028	113.865	105.996	116.121
SREBF1	81.6357	93.4571	89.6256	78.0548
AFF4	5.10779	5.12757	5.12403	5.57249
UBE2D1	11.3418	11.0261	11.2988	13.1332
MPP5	5.98799	4.76372	5.05055	5.66877
SMC1A	25.3656	17.0527	21.1203	21.5959
HSD17B10	62.7888	63.4984	65.1469	60.5763
MARK2	22.0774	16.9196	18.985	18.2792
HMMR	24.4998	21.7629	19.632	23.0599
TRHDE	1.15187	0.68094	0.54705	0.701615
P4HA2	19.1468	13.5354	13.9965	14.9781
NFATC3	9.92495	6.97639	8.42534	8.96411
TRNT1	11.6971	12.0846	10.501	12.5865
ACADVL	265.741	296.262	289.103	277.985
STK10	11.7167	10.7952	12.3219	11.1858
FBXW11	22.5772	17.6571	16.3896	18.5769

ACAP1	2.69523	3.07565	3.27038	4.04079
DERL2	46.997	45.7839	43.2941	49.8076
NDE1	32.3139	24.7876	26.688	27.41
MRVI1	1.49834	2.43101	2.28644	2.05525
TMEM38A	7.76887	7.41095	6.95122	7.16071
AP1M1	46.8881	52.1367	51.2635	49.617
PVR	52.6485	44.9784	49.2321	47.078
IKBKG	9.928	10.6881	11.6886	12.002
XRCC1	28.0432	24.2172	24.6366	24.3175
SCARB1	98.2232	84.1499	93.9705	85.9159
MCM2	107.58	99.8693	101.838	96.202
PANX2	2.31108	3.06864	2.73207	2.34464
RP3-402G11.5	15.8867	17.1442	19.636	16.9714
ALPK1	5.08266	3.79531	4.68317	4.20361
LLGL2	64.8137	74.4759	78.639	68.6383
PDE8A	12.652	13.05	14.1068	13.9561
CLCN4	1.45455	1.46245	1.56327	1.84782
NLE1	58.149	51.0837	52.6587	46.4112
SDHA	95.8933	93.1658	91.5308	87.4441
SMARCE1	118.137	107.814	109.821	118.422
GSDMB	4.06415	2.97779	2.6399	2.66669
KDM5A	8.01756	8.60005	7.50735	7.09925
ADAM11	3.08536	3.68076	3.96116	3.34028
PPP2R3A	1.56595	1.5415	1.61625	1.67906
FERMT2	15.4982	11.828	11.3561	11.7865
IGF2BP2	46.8176	50.3562	45.6307	46.5399
MAP3K13	12.6791	11.0182	9.79379	11.8978
ST6GAL1	5.92752	6.96181	5.70521	6.22043
PICALM	49.0084	46.3751	53.9808	53.2508

NSF	34.3527	31.6277	30.4813	31.7395
GLI2	3.02363	1.92266	2.06181	2.00661
CLASP1	9.05917	7.45711	7.50738	8.31555
MRPS34	210.607	182.218	182.984	194.901
NOTCH3	4.26231	4.94812	4.89791	5.33088
CLNS1A	193.567	109.44	153.556	168.416
PPP2R2C	17.176	16.3842	16.6628	17.1791
TEAD2	16.4184	18.3556	17.5725	18.1545
EED	24.4584	18.522	19.3107	22.2392
TSG101	100.773	87.5622	98.6175	106.487
C17orf85	21.3131	23.1776	23.7918	24.8779
ATP2A3	4.67364	5.9241	8.61155	6.87929
CA12	1.25702	1.20348	1.69843	1.22407
MGLL	6.98308	4.25002	5.16026	4.39933
NTN4	1.00387	1.09445	1.33748	1.04119
BCS1L	66.0884	53.9705	56.6515	54.8113
NUAK1	3.43896	3.31328	2.83122	2.79044
DPP8	12.2484	8.49776	8.9672	9.38954
SLC24A1	5.00257	5.02214	4.49283	3.95546
ZNF532	5.80141	7.13342	5.35726	5.00328
LMAN1	20.5669	18.9058	16.8323	19.8545
PTPLAD1	57.1523	45.5361	45.8352	50.2108
ZZEF1	5.43417	5.26951	5.91015	6.10218
ENO1	1885.37	1752.23	1780.14	1755.23
C19orf10	132.27	118.612	122.945	127.111
ANO8	4.30149	5.95328	6.69966	5.58272
TUBE1	7.46508	8.17668	6.63795	7.61798
ARHGEF10L	4.16171	5.21458	5.51657	4.86101
KCNQ2	50.3018	49.9191	52.1725	50.8569

ACTR6	17.5916	18.1596	16.8604	20.1218
TIPIN	30.7495	21.645	22.9488	25.2585
SRI	109.944	99.491	102.199	106.488
EIF4G3	14.6904	12.482	14.0493	13.8969
NUP37	63.9924	59.8636	55.8937	65.6365
SEMA3A	1.23859	1.31107	1.58823	1.81639
GTSE1	23.3853	19.3807	18.6977	20.1109
SEMA3C	5.15356	5.59777	5.66866	5.80696
TTC38	15.8192	15.3132	14.868	14.4413
ACAT1	75.0457	75.429	70.4922	73.6301
GRAMD4	7.64444	10.1966	9.289	9.60309
CELSR1	5.95396	7.21884	7.38051	6.56268
ZNF638	30.544	29.588	30.5728	31.7136
SLC25A40	9.49463	9.13279	9.07588	11.0753
TIMM21	35.6491	24.9028	23.5846	30.3155
RASAL2	3.43758	3.0072	3.33938	3.29063
VPS9D1	3.26743	3.28387	3.3705	3.12428
ZNF37A	5.73512	5.44842	4.89377	5.50525
MARK3	47.1287	42.4999	43.0866	45.7734
SLC25A3	752.702	759.636	730.619	729.285
FNDC3B	7.8603	7.16453	7.42922	8.51578
FOSL2	20.3291	16.5471	17.3643	16.9872
FRYL	8.0241	8.65081	7.95616	8.62482
TMEM131	14.5743	18.5604	19.2451	19.6472
FSCN1	195.716	199.354	194.015	181.066
ACTB	3468.92	2199.46	2485.74	2369.88
MOCOS	4.61771	5.01015	5.10513	4.677
PLD1	0.935387	1.28499	1.42069	2.05435
WDR62	11.9258	10.3125	10.9683	10.0005

DLG1	23.7721	21.8674	22.426	24.7672
RAB7A	254.667	196.054	205.711	208.029
BCAP29	43.4976	30.1448	29.5326	28.2907
SEC31B	4.4549	5.91141	5.35263	5.6545
SART3	41.6221	35.3286	37.96	35.3961
PAX2	1.5955	1.83454	1.84427	1.70029
EXOSC7	81.4193	65.184	69.7829	71.3855
KIFAP3	5.31541	4.90201	4.65504	5.41358
MKRN2	25.7971	23.6431	23.6474	23.8043
MCM6	96.7764	79.7885	85.5533	87.5744
REXO2	102.352	89.7992	86.6757	99.0574
RBM7	22.4447	21.2553	20.4724	22.2833
RBMS2	10.6812	9.71807	11.5383	11.0071
BAZ2A	31.1553	24.7756	29.5087	28.6959
PTPN23	16.4967	16.4741	17.801	15.818
MLH1	4.5773	5.00186	4.98458	3.1364
UNG	77.3359	71.2791	77.4911	76.9064
FMO4	0.827971	1.23002	1.62796	1.44381
KLHL20	9.6982	7.61827	9.0825	8.68572
SLC46A1	4.3572	4.32203	4.79964	2.82789
SPAG5	40.8763	33.2601	33.5058	34.8701
ANKRD13A	11.6127	9.30505	10.4317	9.76802
TPD52	68.3756	57.6263	53.7031	62.1012
ACACB	2.36937	1.73579	1.63701	1.63345
TRAF4	101.262	99.5267	109.311	99.1585
PAG1	1.15428	0.331828	0.484794	0.386414
GPATCH1	3.91219	3.51845	4.08577	3.46331
ICAM3	11.54	10.66	12.2249	12.9365
NT5C2	40.9577	42.4305	37.3789	41.0655

MCAM	20.6147	15.5701	20.754	17.1543
GPC4	5.45274	5.14506	5.55894	5.44827
MBNL3	1.12307	1.52355	1.14396	1.25966
CAMSAP3	9.24689	8.28729	9.7233	9.38183
RAP1GAP	11.1768	10.6155	10.7664	10.3083
XAB2	63.3047	61.2121	64.3336	59.7705
ARHGEF1	60.2195	56.9773	63.8914	57.8193
STXBP2	57.2152	56.8161	70.7338	58.0465
MAP2K7	32.4427	30.6073	29.5418	24.5934
DGKD	14.592	12.9077	13.0688	12.8755
CTTNBP2	2.29857	1.6454	2.06916	1.57253
TOP2B	25.5263	23.9747	23.0871	25.4142
TM9SF3	38.5596	23.7354	25.8731	31.0386
NFKB2	34.3092	37.1533	41.0192	36.0877
UBE2T	84.3617	66.6677	64.1477	72.9445
PPP1R12B	1.31233	1.53246	1.52393	1.59475
DNAJC10	21.7892	22.0372	20.4512	23.7349
GTF3C1	57.3935	63.9676	67.338	65.2421
IL4R	14.6547	13.0164	14.3114	13.459
USP33	16.901	18.0227	18.5068	19.4822
SNRPA	186.293	176.228	181.867	180.281
EXOSC5	51.7085	49.8497	49.5616	46.053
DYNC1I2	25.204	22.2713	23.8834	26.1064
LRCH4	15.4114	14.8574	16.5169	14.9022
FAM76B	6.86413	8.39674	7.37224	7.82844
SIRT6	23.1708	20.9103	22.4552	19.6846
POLD3	10.3399	8.35064	9.2579	9.41708
CAPZB	134.117	133.612	145.832	135.633
GPR137B	7.60882	8.28507	9.1012	9.21975

PHF17	9.23912	8.54033	8.39911	8.81756
SLC25A43	9.09906	8.73183	8.07679	8.19674
UBE2A	49.9928	46.8578	43.6955	47.2535
FGFR1	33.4675	27.0874	29.6805	32.0392
GTF2I	33.5353	34.5126	39.1078	31.7449
FBLN1	10.388	10.7512	11.6323	9.93557
MAP2	0.676999	0.819782	1.01133	1.0455
PIAS2	12.3478	12.9814	13.0818	13.3627
ARAF	26.2876	21.0887	24.3591	22.3238
MCCC1	3.79731	6.8233	6.71743	4.40111
NEBL	3.50701	4.95058	4.18181	4.00427
ACER3	11.2999	7.4837	6.79307	9.47962
UBE2K	80.3777	75.0384	65.9113	84.0079
PIK3C3	24.4022	27.812	27.0425	26.2418
N4BP2	1.23344	1.26899	0.997115	1.18107
C12orf5	16.7044	21.6265	17.8237	19.8064
TULP3	13.5236	11.9606	11.903	12.991
SYNJ2	10.2756	8.45057	8.81935	9.2543
PPP2R5C	53.1344	48.4418	48.7211	51.1777
PMS2P1	25.8548	23.4806	21.7721	25.2735
GNB1	243.195	245.721	250.243	247.358
HOXA9	77.364	63.8764	71.0802	73.1829
MLLT10	24.5821	23.4414	21.7907	24.0447
NRD1	76.5665	71.1897	75.8814	71.3547
VDAC3	137.839	105.044	116.442	124.941
PCM1	26.7121	25.612	23.6115	29.1008
TNRC6C	3.98176	3.70519	4.2049	3.55642
CBFA2T2	2.3692	1.96453	2.30109	2.14794
ITCH	20.8561	13.2489	13.3669	16.7323

TP53INP2	1.68951	1.78135	2.22583	2.01278
SDF4	113.489	98.4263	104.797	100.179
TP73	7.21545	7.0684	7.48473	7.65529
TOLLIP	22.6885	23.4265	28.1494	24.9081
UBE2D4	7.87272	7.13194	7.94551	8.0636
THOC1	11.2951	10.4898	10.0021	10.4839
FKBP7	1.97469	2.51579	1.84707	2.76999
OSBPL6	3.74857	4.30608	3.69516	3.94218
SLC1A3	10.6547	8.41969	9.51129	8.37441
XRCC5	361.505	304.528	298.303	350.444
SP140	1.56527	0.53816	0.522317	1.48355
MKNK1	17.8694	16.0017	17.9744	18.1316
TNS1	1.61794	1.73574	1.59048	1.58809
REXO1	49.712	48.6704	54.1683	44.6354
SAR1A	79.0695	65.7447	71.2957	73.5941
CDC14A	1.42254	1.41728	1.32872	1.58742
SENP1	7.62548	5.88107	5.71077	6.22569
CIC	14.9422	17.0269	17.2267	14.9467
LIPE	12.7748	9.87125	9.53416	8.586
FDFT1	68.9706	58.5652	60.2	64.005
PAFAH1B3	107.845	105.367	117.22	98.6196
OPHN1	1.2044	0.980729	1.17906	1.40442
KIF22	133.595	113.33	110.162	111.049
LRRC16A	6.07427	5.70966	6.44412	6.34473
PGM1	17.4359	18.8064	20.4721	20.6303
DDX1	92.8504	79.6754	84.6212	91.0794
DNM2	67.8277	68.108	70.1339	65.7958
EPB41L2	28.5862	26.0738	23.31	27.0748
STX7	14.6794	13.9317	15.95	16.5078

RABL2B	8.823	9.94067	9.30709	8.79911
KEAP1	116.108	96.2012	98.6444	101.626
PTPRH	2.48359	2.81697	3.17716	2.98776
SLC35C2	25.1686	26.2366	28.3408	27.693
CRYBG3	4.51285	3.93592	4.31335	4.51424
RFX3	2.12365	1.49847	1.52713	1.53876
RIF1	8.10875	7.09123	7.28142	7.71213
RAB21	29.2482	21.3645	18.8784	21.3299
SMARCA2	1.8276	1.98509	2.96197	2.36621
SESN1	5.75527	8.13099	6.83	6.92578
MID2	1.98836	1.60463	1.51734	1.59453
SRCAP	19.2748	18.1109	19.7179	19.0815
KIAA0020	33.0661	27.8706	24.8045	30.4852
CNOT4	6.38896	5.85453	5.8471	6.45506
PSEN1	12.7909	13.0073	12.8995	12.9134
CPOX	189.47	193.382	230.066	216.258
CLDND1	191.122	182.481	185.961	184.529
MOK	6.09779	4.02782	4.6893	5.18687
HSP90AA1	1553.88	1191.15	1154.63	1308.25
RBL1	5.12049	4.13072	4.36421	4.65666
DLGAP4	15.352	14.4733	15.9551	14.2959
IGSF9B	1.5665	1.80677	1.73042	1.53887
CROCCP3	1.74847	1.77977	0.727223	0.45485
NDC80	25.7047	21.6795	19.8647	22.505
AP4E1	3.91314	3.32086	3.54841	3.903
RSBN1	5.86218	5.17865	5.34607	6.17591
MAGI3	2.39758	2.3042	2.47536	2.89701
TCF7	20.0576	18.1034	17.4489	16.2
OSTM1	9.00544	10.6314	7.50459	6.74362

PCNP	80.4428	79.5382	70.4264	78.7347
EXD2	7.39236	6.83738	7.60712	7.90864
ARG2	5.29767	5.63299	5.3567	5.06569
MEF2C	2.97814	2.40603	2.72629	2.50794
UBA5	46.4682	37.3863	40.5431	39.8944
STK17B	3.53403	3.34063	3.10421	3.45243
CDC14B	7.72752	8.57325	8.02256	8.93369
ZNF510	1.24672	1.20842	1.03514	0.984302
JMJD4	13.6131	13.4152	14.0751	12.8728
DUSP12	29.9896	24.6946	24.0082	24.3564
AACS	7.11803	6.96482	7.50059	7.2591
KIAA0141	31.5097	32.4793	36.3372	34.3839
CADPS2	3.31606	2.52503	2.61802	2.90694
HSPB11	31.4002	32.1634	28.3235	33.7181
PHLPP1	4.3271	2.90464	3.73002	4.41577
ATP8B1	4.23075	2.61705	2.87442	3.05592
SMARCD3	14.0563	13.9471	15.9255	14.2368
WDR70	23.1622	23.0628	20.4968	24.2286
STRADB	13.1055	17.6698	17.0618	18.6979
BZW1	292.068	243.876	222.711	238.047
C1QTNF3	0.844526	1.10527	1.30578	0.678555
ME2	38.8888	34.341	32.3577	39.266
C5orf22	13.1999	10.1843	10.7943	10.4817
CCNT2	8.88388	7.60369	7.95288	8.31259
FAM135A	5.8524	4.29714	5.78267	5.70345
COBLL1	1.8044	1.32071	1.10148	1.71664
DLG3	14.8995	15.9277	15.3346	16.3838
TRAF5	3.02676	2.89625	3.24655	3.02579
MRPL22	62.2852	55.854	55.528	63.6384

GEMIN5	27.3172	25.0268	25.1375	26.3418
NFE2L1	96.1582	98.4663	98.9408	94.6418
GSK3B	17.3751	16.0438	18.0099	18.5905
ITGB5	98.3728	86.5292	90.3091	89.7422
ERC1	7.16388	4.9743	5.20047	5.4767
XPO1	99.1697	92.9852	90.9183	100.492
RNF13	24.0279	25.9969	18.1779	20.6804
PALB2	8.95748	7.90635	8.73756	8.54332
DOPEY1	1.11429	1.57427	1.41739	1.51914
LYRM2	17.1855	15.4403	14.5336	17.312
BCKDHB	2.52677	3.25889	3.12818	3.19792
KAT6A	6.45794	5.08629	5.57062	5.94594
ZCCHC6	5.10111	3.8414	3.96798	4.80768
ULK2	4.82572	3.4056	3.64286	4.27161
GRHL2	5.86833	6.82769	4.5697	4.5469
TNPO1	69.7232	48.2503	57.9596	63.2983
PLOD1	137.481	126.954	127.673	122.685
P2RX5	12.773	12.9336	14.2122	13.1518
ITGAE	36.0395	33.6552	31.9621	37.0598
DIS3	25.9212	24.3974	24.3617	29.0776
PIBF1	8.27188	6.4249	7.16476	6.68408
TDRD3	5.37819	4.4719	4.832	5.7128
NUFIP1	9.93752	8.40809	8.62184	9.44545
PDS5B	7.68099	6.21956	6.20086	6.70615
OXCT1	22.2737	23.2373	21.9745	27.2004
RRAGB	5.70372	7.30058	6.74979	5.87363
CYLD	3.12261	2.49104	2.97145	3.0971
SLC27A5	39.7375	32.3884	29.8167	35.8572
ZNF324	6.34055	4.42897	5.1897	4.9898

ZNF416	1.82761	1.57844	1.40328	1.57046
ZNF586	1.74248	1.42881	1.57864	1.5545
ZNF446	3.40555	3.1829	3.30001	3.06957
ZNF8	2.42505	2.66943	2.40642	2.11
ZNF264	7.78414	5.9076	7.26378	7.47685
RPS5	2163.3	2155.56	2185.04	2105.22
FAT1	269.31	308.24	297.611	281.131
YTHDC1	26.2947	23.2677	21.9863	22.3062
CHMP2B	29.4855	22.2241	24.575	25.7029
SMAP2	10.1239	8.47197	11.2896	11.0358
PPIE	78.6757	64.6666	66.5087	68.8804
ZMPSTE24	40.3109	35.4717	31.0288	34.6005
STARD7	110.136	101.339	105.134	105.853
NOA1	19.9189	19.295	20.8447	20.0225
REST	6.03783	5.01983	4.74073	5.54298
SSH1	19.1638	22.3969	22.1333	22.3484
GSTP1	1029.91	1081.46	1083.74	1034.08
APLP2	158.87	157.173	163.369	167.552
KIAA1467	6.6839	4.31396	6.84483	6.7389
WBP11	53.2957	44.7414	46.8647	52.2258
EIF3I	401.714	345.633	361.134	369.635
NKAIN1	0.706306	1.18131	0.951786	1.01658
COL16A1	1.69353	1.62912	1.55403	1.89393
TXLNA	18.0105	16.0847	14.4439	14.762
NCOA1	3.21733	3.24395	3.1459	3.48607
AGBL5	20.3461	21.6653	21.0672	22.0528
KIF3C	1.35178	1.86656	2.01621	1.84326
RAB10	88.1155	70.4048	66.5301	74.576
HADHA	49.0485	56.0525	54.2733	52.5087

MAPRE3	6.55076	6.89309	6.94828	6.89998
CAD	67.1283	68.4977	70.444	61.7003
CD59	95.4038	69.1886	71.8402	77.6089
CD82	7.35392	8.81016	10.7029	9.71432
BCORL1	6.61279	7.28036	7.55417	7.11113
ATRX	4.92882	4.49714	3.93214	5.08842
TAF9	74.8887	71.757	68.8398	71.1915
MYNN	7.60874	6.21375	6.68737	7.7709
SCAMP1	14.1407	12.9101	13.6723	14.8691
PREP	35.4788	33.6957	36.6877	36.5207
HACE1	3.82781	4.79721	4.38178	4.7536
SEH1L	62.4444	49.17	53.3634	57.258
WDR47	4.21811	3.47667	3.77123	4.10634
WDFY1	20.1379	16.9949	17.8686	17.0149
OVGP1	1.1273	0.95632	0.779184	0.820193
SLC25A24	14.014	14.5402	15.545	17.3183
MAP3K4	13.0834	10.4035	10.1286	12.0893
IGSF9	1.93874	2.37725	2.3824	2.13271
ABCB1	1.78348	1.16126	1.78162	0.912107
ZNF213	5.89805	6.7148	6.33632	5.34568
AKR1B1	52.1584	49.5456	47.7051	48.5279
CPNE3	47.7716	42.3549	45.9334	48.9991
RRN3	39.3474	34.5791	35.8737	39.3311
CTTN	83.3137	86.2185	91.1774	84.9798
MTIF2	30.2244	28.0087	26.45	29.4497
DDHD2	32.7145	28.7728	36.6098	37.5229
TTC39A	9.41034	11.8819	10.5282	11.1805
EPS15	10.2054	11.3881	9.12849	9.9182
ORC1	20.7397	14.2085	15.7977	16.2882

MGST2	10.853	10.3371	9.65936	11.3025
CHERP	45.5923	41.9122	43.7408	39.5985
ATG16L1	21.2562	19.6785	21.7248	21.6899
USP40	14.0474	16.9035	16.3017	15.5169
POMGNT1	44.0056	45.1823	49.8654	48.2416
RAD54L	20.8071	13.9999	16.7692	17.8258
MAST2	22.5706	18.8832	20.8656	20.518
DNAJA1	249.995	170.098	177.683	206.281
B4GALT1	19.089	17.8326	18.6523	18.8746
CHMP5	55.1114	46.128	50.2171	52.1
NFX1	18.0467	16.7179	17.1818	17.9868
DIMT1	47.2037	40.1975	41.1394	45.137
IPO11	27.6632	24.9223	22.2807	23.9543
EIF2AK1	74.3615	79.4753	67.197	77.2988
EPDR1	12.0675	10.3224	7.7437	7.9708
SNX10	11.8638	11.7767	11.4751	12.4663
SEPHS1	85.8673	72.6561	75.1763	77.5122
MRPL28	61.0042	59.3255	59.199	58.6395
ITPKC	5.3036	5.69387	5.38719	5.70105
RBM22	33.6183	29.0783	29.8001	30.9635
TMED2	196.589	175.478	177.887	183.917
ERO1LB	1.24651	1.07282	1.15177	1.24248
ZFAND6	33.4903	29.2266	33.5562	35.5451
TXLNG	14.0955	12.2424	11.5244	13.5133
LAT2	1.87272	2.10272	1.74514	1.52921
HUWE1	40.2592	43.6848	42.4671	42.2037
ZW10	15.4447	14.0993	13.7135	15.8773
ALG9	7.76745	7.45743	6.80915	6.30247
ACOX3	7.41492	9.22556	8.17844	7.79275

MTMR2	22.9307	21.1287	20.3574	22.6096
PPP1R15A	18.7838	18.9858	18.5372	17.9385
HSD17B14	1.5649	1.79012	2.00655	2.32202
TRIP6	102.504	94.1286	96.774	92.4446
ACHE	2.34886	0.967441	2.58036	1.0744
FTL	2686.88	2461.19	2083.74	2279.81
SRRT	153.412	129.725	135.156	129.244
BAX	168.936	202.518	180.492	194.351
NLK	7.38392	5.95029	6.56246	6.58412
PIGS	40.0871	36.6899	44.3296	41.339
ADAMTS2	1.10322	1.64596	1.523	1.36235
ATXN7L3	46.4424	44.8451	45.2693	48.7039
PGS1	30.8272	27.0033	28.0709	29.182
PSMC5	232.214	170.125	187.747	196.74
UIMC1	12.8873	13.4704	11.8309	13.0842
LPCAT2	8.73242	6.81189	6.30936	8.1404
OGFOD1	29.2476	27.8085	27.2563	29.6818
SH3BP2	17.5807	17.9313	18.5892	17.2004
NOP14	44.493	39.5184	38.7305	43.0622
ADD1	56.2767	58.7865	53.1409	54.1723
L2HGDH	7.31138	5.5515	7.14225	7.26913
C14orf166	154.048	145.271	134.62	141.25
GMCL1	10.5655	9.53583	9.78381	9.75068
SF3B2	167.532	153.47	150.986	155.514
KLHL42	10.2171	10.6893	10.5825	9.95193
GNAS	436.629	445.364	451.142	437.151
DNM1L	32.3825	30.9061	31.1712	32.5412
ERGIC2	62.4176	64.5481	64.1753	69.4241
TFAP2C	1.69099	1.52307	2.14066	1.84555

AURKA	67.0032	54.1861	50.8236	58.1344
PIR	26.7378	29.1829	22.3052	26.2533
AAMDC	7.61583	8.17759	8.99134	7.79878
RFX2	7.30635	7.04	7.07183	7.28631
METTL2A	29.9347	22.9317	25.7377	28.1463
SULT2B1	1.39909	1.04869	1.01427	1.33139
ALG6	12.5447	10.9734	10.5572	12.4205
CNOT3	7.25575	5.89147	6.18831	5.10939
PTPN4	6.36378	7.11085	7.76022	7.97789
DDX18	98.4642	78.4573	77.0085	88.3841
KHSRP	386.055	359.216	366.997	370.549
GNA11	52.1678	44.5187	49.906	48.3231
ASAP3	4.75496	5.01289	5.30582	5.17633
EDEM2	12.4209	12.203	14.5854	14.4801
DNMT3B	9.30954	5.22347	5.31228	5.50368
TPX2	73.0469	35.193	45.6465	54.2852
FER1L4	0.787666	1.33015	1.06387	1.428
PDRG1	33.1758	28.9499	28.0984	30.5503
EPB41L1	25.1796	21.5676	24.2704	24.2215
DOCK9	29.8712	27.128	29.968	31.2369
ANKRD10	42.1487	48.3735	44.1545	46.7728
TGDS	16.2187	14.9818	13.852	15.4851
C3orf18	3.66691	3.94267	4.30943	4.3983
COQ9	52.9802	48.9181	47.4407	48.9487
TMEM40	6.12324	6.2529	7.46753	8.13169
KIF9	13.2449	10.647	11.5851	11.9661
CRLS1	47.9189	42.743	45.7282	48.7353
PPP1R13B	5.98791	4.8895	5.60528	4.82638
ATRN	6.78033	7.26098	7.84748	7.88249

SMOX	15.9851	22.6073	22.9698	22.9342
FKBP1A	308.781	331.85	341.105	327.153
NSFL1C	72.5965	66.4722	63.0661	66.0581
SLC4A11	11.509	11.3477	12.0252	10.147
C20orf194	1.0224	1.53374	1.59632	1.51916
ZNF343	5.676	5.17909	5.28085	5.36147
EBF4	0.983096	1.35853	1.68286	1.61628
MAVS	23.8398	23.0115	24.4186	22.2134
LZTS3	2.26853	2.13964	2.74975	2.42735
XRN2	61.0554	54.1303	53.4121	58.3099
PLK1S1	3.02739	4.42708	2.96192	2.69287
DYNLL1	284.068	270.474	233.559	265.207
TESC	18.3682	17.7844	17.2241	15.2806
SNX5	137.133	127.162	127.619	136.478
RPL6	1779.51	1695.71	1626.65	1787.54
MAPKAPK5	31.6753	30.0112	32.5025	31.1997
ESF1	15.5429	11.8748	11.9967	14.4647
RBBP9	5.05596	6.63675	6.42295	7.07802
ANAPC5	79.4288	68.4923	74.4922	74.1314
SLC23A2	6.12811	4.01621	4.54227	4.6397
SLC24A6	14.9102	16.7383	16.9485	17.1603
TMEM230	65.188	60.7902	51.5385	63.4144
KDM2B	16.2321	10.2658	10.4423	9.72465
LHX5	1.61579	2.02491	1.99011	1.92771
TASP1	6.40943	5.32709	6.05572	6.08695
GCN1L1	81.3663	79.7741	84.3755	81.8547
RPLP0	3881.34	3976.43	3892.78	3897
PXN	148.665	114.397	124.182	129.688
KIF16B	1.43222	1.16835	1.10459	1.18628

TRMT6	14.4651	12.6931	12.5153	13.7191
PEBP1	245.172	232.884	232.673	231.046
BRAP	11.6224	10.9589	10.7693	10.5277
ERP29	104.211	110.821	92.4706	92.6461
FUS	355.767	332.118	319.78	360.751
IGBP1	30.0554	21.8711	22.4777	25.1297
FXYD5	78.324	70.1389	78.189	80.6509
ZNF302	4.08774	4.0896	4.21846	4.20301
GRAMD1A	38.5211	39.8444	38.2888	35.4838
FXYD3	4.59844	4.89555	5.40023	5.85766
CDIP1	6.56537	6.67523	6.78128	6.41031
CMTM1	6.52554	3.33257	4.42901	4.37857
GANAB	217.405	222.339	230.473	216.757
GMIP	6.25158	7.41324	8.36384	7.86794
RBM41	4.59857	3.90687	3.62926	3.75677
BIRC5	92.0558	58.1208	59.9891	69.4607
MLF2	313.299	294.341	318.016	316.745
OTUB2	2.36174	3.58182	3.2947	3.3406
DDX24	81.4222	65.7132	72.326	75.8119
ZBTB25	18.2912	18.4914	16.7934	18.3614
NECAP1	23.8837	22.8601	25.0601	25.1754
ARHGAP4	18.5802	20.2999	21.0428	16.6295
DHX32	19.0096	22.858	23.0302	22.9187
RCOR1	10.7597	7.40166	8.61734	9.67587
GPATCH2L	12.1516	9.7193	8.63799	11.1175
LTBP4	98.8566	104.55	118.277	98.5567
BLVRB	89.4127	102.743	108.656	101.57
SLC9A1	11.8807	9.97743	8.38069	8.85629
SPTLC1	51.98	47.2412	48.5982	51.1373

PAPOLA	106.18	94.6078	87.858	101.399
CCNK	26.563	28.635	26.6549	29.02
PCBP4	31.4206	34.5822	34.9952	33.0092
YPEL3	1.44617	2.56519	2.19922	2.56425
MRPS33	70.6793	70.0203	66.8782	71.8502
NDUFB2	228.702	203.466	192.316	207.675
NUDC	168.162	122.045	132.866	141.267
MAEA	66.3478	63.5543	70.893	66.2044
ICAM1	1.95773	3.03247	3.26721	2.73961
STRN4	69.5316	64.8262	72.1279	60.361
MUL1	23.4113	22.2243	23.1175	23.4766
TFAP4	28.6312	26.5029	26.8309	25.4461
PDCD7	18.8192	13.7959	17.0666	18.2603
SPG21	49.0898	47.3458	49.5226	54.4838
DNAJB11	106.637	87.0766	85.6688	97.825
CHRD	2.99918	2.94763	3.15483	2.91619
FLT3LG	1.03823	1.22842	1.31397	1.51194
RAB11FIP3	7.94313	7.69543	7.26453	8.01231
GNPTG	18.5043	18.4346	18.8283	18.5182
ZNF268	4.71934	4.01498	4.85047	4.90328
GOLGA3	15.4592	13.6561	14.7804	13.6257
PABPC4	343.548	253.795	206.84	208.201
CERS4	17.1491	19.9474	20.3428	17.8402
MCOLN1	16.4813	14.4285	16.4402	14.0185
USP48	29.3534	28.5077	29.8411	31.7044
EFNB1	11.6373	12.936	13.1456	11.7466
PDPR	10.318	6.92764	6.47169	6.88261
AARS	73.0915	101.655	90.2871	80.5001
GLG1	56.092	67.9035	70.5793	66.2653

KIF4A	18.3681	14.7895	15.5845	16.0467
TNRC6A	28.142	25.4785	24.8844	23.1246
PLEKHG2	17.704	15.0559	12.3677	11.0792
NAT14	39.9653	41.2117	39.2019	40.4934
PITPNM2	3.81256	4.32893	4.76473	3.99551
EXOC1	7.28193	6.96787	8.18952	7.25794
RBM27	15.0768	14.9415	15.4663	14.62
OSBPL8	15.2364	12.1008	14.1738	15.5146
DTX2	15.2364	15.823	18.4034	16.291
PUS7	21.153	18.5743	18.2476	19.5174
LAMB1	10.5093	14.0754	13.0341	10.3953
DLD	72.0921	61.7473	61.8299	72.7664
WDR7	3.40957	3.71983	3.71907	4.48771
TXNL1	76.7704	72.2864	67.2676	74.8939
ABCC6	0.740156	1.21575	0.979654	1.22325
CMTM6	33.0752	31.0499	32.0742	35.2062
ITGA6	28.2042	33.1446	28.361	32.2643
MLTK	13.8016	12.002	12.4164	13.5104
FH	52.0531	46.4701	46.4846	51.1155
SEL1L3	27.682	31.6737	26.3636	27.6444
CDV3	108.073	93.6113	90.2819	103.964
ALKBH5	59.607	27.5147	39.3571	41.0089
NLRP1	0.49556	1.15932	0.907432	0.740416
ACKR6	0.854784	1.17768	1.21903	1.17009
SPAG7	37.413	34.1578	35.8045	34.7521
ORC6	40.5056	33.0365	31.5365	35.6916
ZFHX4	1.20427	1.37478	1.32593	1.36011
ZC3HC1	24.4937	18.6401	21.8213	23.7443
RGS17	1.06505	0.93685	0.987618	1.19072

TMEM101	38.1367	36.7502	38.9706	35.9288
PSME1	68.528	80.4034	77.1721	78.5738
PPP2R3C	11.027	9.99225	7.86718	11.0933
HAUS4	27.6415	32.1589	28.6303	29.3401
SLC7A8	1.16472	1.71131	1.54978	1.67392
OSGEP	20.5282	17.6027	18.9115	17.1709
SLC22A17	2.44509	3.27276	3.33123	3.10611
RNF31	60.8494	51.9816	57.7673	50.646
SCFD1	46.577	38.8099	37.7776	48.0811
G2E3	23.0535	16.3143	14.5794	17.5533
HECTD1	67.4426	61.4076	68.3182	64.4199
HNRNPC	826.293	666.361	643.034	735.198
SUPT16H	109.706	98.4286	96.9236	102.15
TOX4	29.6271	25.4392	28.2141	31.388
GEMIN2	28.4628	27.3224	24.3488	28.8425
TGM1	3.0734	2.95525	3.26052	2.38085
TINF2	22.0615	24.0945	24.6819	21.9335
SEMA6A	2.30421	2.43014	2.12505	2.41311
TRPM7	9.76953	11.1921	11.2808	10.8605
TYRO3	26.8424	24.2209	25.6229	22.1458
WDR76	22.3535	18.4979	18.4827	21.3689
SNAP23	26.9477	27.9326	26.2657	28.368
PHGDH	44.3949	90.6884	61.4196	54.1454
COL9A3	13.7036	16.8942	14.2371	14.3465
EZR	649.987	571.553	594.317	600.998
MYL6	870.53	829.741	804.619	825.016
AGO1	9.23487	8.37253	7.73409	8.49296
CLSPN	21.2295	13.2506	15.6877	16.2098
RFFL	25.3115	25.6364	24.8125	27.9169

UNC13D	5.67458	6.68386	5.87823	5.58615
MFSD11	164.975	146.546	174.215	152.044
DPYSL2	7.20112	7.20608	6.8049	7.64701
GPATCH2	4.42014	3.3354	3.3983	4.00132
NUP50	43.4081	32.3793	35.4147	38.6962
CDC45	31.8138	28.0969	28.221	27.5915
COMT	79.6566	85.3027	88.5367	80.8057
XXbac-B461K10.4	3.11188	2.95726	2.84212	2.57006
ECHDC1	83.612	79.2047	76.173	78.6167
LRRFIP2	18.927	16.2672	17.7839	18.8554
SEC22C	27.927	22.3226	21.1728	24.7258
XYLB	4.68	5.25494	5.007	5.47744
HDAC6	14.0328	14.1702	15.7683	14.2319
GABRP	2.03473	2.11809	1.9857	3.25781
CDC6	68.4237	50.8222	51.7731	63.2339
UPRT	3.61143	2.01462	3.16969	3.44468
CDC23	28.3071	18.9675	22.1949	23.3127
AAAS	51.5663	48.8159	49.7741	48.9323
CBX5	13.8934	15.5067	13.8915	15.2087
SUCO	7.04984	7.69234	8.06159	8.85088
MSH2	32.1844	27.8183	26.5661	32.0032
MAP3K1	1.78401	2.16634	2.08689	2.13368
DHPS	76.1388	73.4783	75.6143	72.9085
HOOK2	31.8557	28.73	27.8426	21.928
ARCN1	50.8451	46.1263	44.8294	42.7588
EPB41L4B	7.08998	6.22606	5.75283	6.62857
TMEM38B	18.6627	15.902	18.3743	19.6807
PSMD5	36.5833	35.9891	37.5267	43.7996
PTGS1	1.29569	1.368	1.36295	1.38497

NUP188	83.3205	73.7659	77.3321	71.4492
CRAT	9.43012	11.3903	12.2939	10.9605
NANS	36.4776	31.5434	31.3069	32.6186
TBC1D2	8.93368	9.1765	10.5422	9.28406
DFNB31	3.82172	4.28765	4.4188	3.97584
CWF19L1	34.603	30.2718	27.8834	32.1702
SEMA4G	7.69368	6.5066	8.4317	7.18796
BTAF1	13.4123	12.895	10.9983	10.9746
IKZF5	6.68688	6.20245	5.58674	6.01628
BAMBI	5.92649	7.23111	6.48226	6.60996
IL11	0.970698	1.02144	1.01861	1.25929
WAC	48.4803	43.2262	40.6358	42.6626
CREM	20.9935	23.9021	20.9442	19.482
NUBP2	88.5973	85.7625	93.9222	83.9857
HIVEP1	1.92874	1.5469	1.93315	1.56213
FKBP5	24.8522	23.381	25.0966	25.7754
SRPK1	51.5146	39.7944	42.6776	45.1038
BRPF3	8.72647	8.31337	7.81938	7.98808
MRPS18A	72.0062	69.4822	73.3076	70.5746
TMEM14A	47.1905	38.3922	37.6987	44.2134
EFHC1	1.49959	1.24437	1.21631	1.05103
RPS18	580.388	582.242	634.349	672.427
BAG6	23.8676	25.5056	27.335	29.6721
VARS	19.3491	20.7518	21.4912	21.7848
HSP90AB1	1870.12	1722.52	1690.24	1736.32
CDC5L	12.1852	10.8525	10.0725	12.0975
ITPR3	32.7288	28.9354	29.6315	29.0002
ZNF184	3.40711	2.94078	2.76238	3.0593
DSP	19.0684	20.8407	19.6504	20.513

SIRT1	10.3584	8.51309	8.33671	10.3657
HNRNPH3	210.713	140.603	149.195	168.104
IFT74	5.99082	6.71914	5.77762	5.8765
JAK2	1.32908	1.44749	1.82819	1.9654
ABL1	21.9292	23.0705	23.3208	22.7821
ACOT7	146.434	140.97	138.212	134.72
SH3GLB1	25.1127	22.2396	22.5534	23.1314
CDC7	15.7036	14.7736	14.0177	15.7787
PCSK5	1.84107	2.49537	2.10778	2.15169
SCD	93.3908	67.84	61.6394	74.4198
TMED1	35.1902	32.642	35.0993	31.3208
ABLIM1	38.394	39.8394	38.1791	42.2671
ERMP1	14.5696	14.5062	15.4276	15.7959
RAB18	52.5033	48.1496	46.1084	52.1487
NRP1	1.24463	0.541276	0.738592	0.692969
HSD17B7P2	1.28866	1.20019	1.29222	1.33503
TSPAN15	10.4139	8.46846	10.2003	9.55342
H2AFY2	24.9867	28.9473	26.9847	26.8283
FAM21A	5.0492	4.50506	3.78581	4.39209
MAST3	3.00718	3.21374	3.73507	3.46756
MZF1	7.14378	7.86726	8.22107	6.45237
OCEL1	19.2558	17.0425	15.9774	15.3321
MYO9B	31.0716	28.6891	33.87	31.2399
KCNK6	7.11463	6.66154	7.5494	7.4814
PSMD8	282.608	252.752	262.802	260.892
FBXL19	32.9501	19.0391	20.5919	21.6663
HSD3B7	2.70336	2.43073	3.01904	2.53588
SETD1A	10.2482	9.28308	9.75451	9.08611
BCL7C	52.413	45.8765	46.0836	49.5857

MAGEB2	113.078	117.643	115.611	124.714
EFNA2	5.76483	5.50126	5.8258	6.15843
CIRBP	145.548	149.708	160.497	172.547
ATP5D	367.862	342.446	354.775	327.74
C19orf26	10.9342	11.2199	12.0164	10.7155
HNRNPM	515.435	421.843	456.287	425.469
2-Mar	15.1458	16.7031	17.333	15.476
NDUFB7	162.617	152.472	160.433	155.937
TECR	155.212	140.424	145.928	136.415
TIMM13	201.562	179.746	177.506	184.931
CDC34	132.869	122.476	125.527	116.01
MTAP	113.964	92.2315	83.7798	100.34
MISP	29.3973	21.3183	27.5408	27.6315
CEP170B	20.3756	19.8563	22.2516	20.148
POLR2E	262.899	248.279	263.42	257.231
POLRMT	112.128	97.5554	102.375	92.311
HCN2	1.62702	2.16343	2.52619	1.99642
RASSF7	32.4265	28.8608	32.9263	27.9282
GADD45B	21.2862	17.4628	15.7325	16.2058
PALM	7.42172	9.96768	10.9178	8.79033
MKNK2	79.3954	105.98	99.847	85.6979
ARVCF	12.7907	16.9449	18.878	13.587
TRMT2A	65.9276	60.6662	65.9916	61.6629
RANBP1	370.138	326.618	318.248	331.414
ZDHHC8	41.5312	36.3618	40.2973	36.1251
KLHL22	6.26082	5.5132	7.41753	6.67297
MED15	28.3552	25.8378	29.4847	26.6335
SNAP29	23.1685	21.1739	22.3663	22.5553
CRKL	34.4837	27.3422	26.4063	29.0834

LZTR1	39.8121	44.9837	40.7788	35.5972
MMP11	1.53135	2.49117	3.14201	2.9556
SMARCB1	84.3697	79.5397	83.2412	79.3186
BCL2L13	18.6722	25.143	22.719	24.1373
DDTL	8.29131	8.7551	9.81453	8.42503
DDT	208.733	207.407	227.575	222.367
GSTT2	9.52858	9.61076	10.6293	10.2134
CABIN1	20.0043	18.9198	19.4774	17.5291
TBC1D10A	8.80088	8.30762	7.9839	7.56184
SUSD2	4.47086	4.83408	5.52477	4.94556
SF3A1	112.246	85.1201	99.0111	97.6669
RNF215	8.70488	9.8907	10.9645	9.84031
SEC14L2	11.7589	12.8941	12.7685	12.5474
SPECC1L	9.40417	7.91439	7.79696	7.95415
PPIL2	22.0215	20.589	23.061	23.0652
YPEL1	1.41008	1.40469	1.27463	1.29403
SNRPD3	231.324	196.664	197.107	197.407
PES1	155.388	136.766	139.187	136.355
MAPK1	59.052	48.1878	54.5913	61.2932
GGT1	10.6306	13.2269	13.8674	11.4075
PRODH	1.54815	2.29725	2.49794	2.00834
PPM1F	15.3765	15.2446	17.687	17.1754
SLC35E4	5.13756	4.33943	4.99159	4.54667
TOP3B	18.331	17.2777	17.8949	17.0479
DGCR14	13.2318	10.9406	12.2907	12.4807
CRYBB2P1	10.0574	8.66872	8.8268	9.37694
MFNG	1.33871	1.1849	1.18802	1.25209
CARD10	22.0554	18.0719	19.8919	19.0289
LRP5L	2.38286	2.10606	2.3052	1.88128

SLC25A1	42.4681	39.5629	43.738	41.6584
ADRBK2	3.02395	2.69736	2.5935	3.17257
GGA1	29.7152	32.1516	32.6818	31.0582
HIRA	23.4004	20.5035	21.6522	21.536
SH3BP1	33.9406	27.0848	33.045	29.46
LGALS1	615.557	586.618	645.628	615.717
HPS4	17.4811	16.4809	17.4567	17.5523
SRRD	38.2444	30.637	30.3826	32.6364
PATZ1	14.743	15.7079	16.9691	16.6474
TRIOBP	62.6185	79.9941	81.5591	71.5571
TFIP11	33.6656	30.8987	33.3823	31.944
GCAT	58.8353	56.96	60.2065	54.045
ANKRD54	23.5468	16.9117	19.1712	16.996
EIF3L	556.59	589.989	536.849	579.955
NHP2L1	434.208	364.864	374.752	379.124
MICALL1	14.6211	16.4191	17.3053	16.4329
POLR2F	138.381	121.031	126.013	124.087
CCDC134	4.44064	4.5751	3.82025	4.27686
DEPDC5	12.347	11.4579	11.6376	14.1255
PICK1	19.6158	19.9048	21.6513	20.4477
CENPM	50.6278	39.6016	42.2716	38.7138
3-Sep	5.29895	4.22018	4.62645	4.74692
TPTEP1	7.98741	6.93611	9.84486	12.4069
KDELR3	22.666	23.4425	23.7027	25.2788
DDX17	61.6294	58.1107	59.4245	62.9175
TCF20	8.62854	7.54823	7.42329	7.8209
HSCB	12.6768	12.6192	12.0646	12.7943
CBY1	16.5216	17.8837	17.3485	18.3806
TOMM22	195.195	168.577	155.245	172.331

XBP1	42.8612	46.0613	41.4989	42.3362
RTCB	51.9796	49.1976	48.5503	50.2204
JOSD1	36.9008	32.6599	36.4903	36.4363
FBXO7	54.1865	48.3341	51.3547	51.6347
GTPBP1	10.4798	10.2787	11.1667	9.69282
POLDIP3	50.5318	43.2875	42.3927	43.4772
RAB36	4.46935	4.10624	3.91768	3.92404
PPP6R2	28.1022	31.7747	33.0692	29.6445
SBF1	35.5791	38.9228	40.9436	34.9415
SUN2	26.1467	26.0857	28.4994	23.6768
CYB5R3	109.654	126.637	129.674	119.697
DNAL4	9.58115	11.3939	10.0787	10.2572
LMF2	27.662	29.698	31.1651	28.3537
RHBDD3	21.3605	20.5106	22.5282	19.7841
PACSIN2	25.4471	23.4906	25.0062	24.6628
TTLL1	1.12896	2.30438	1.99109	1.89187
AP1B1	42.1243	41.0503	44.3933	40.5604
HMGXB4	12.4213	11.345	10.7666	12.2545
TOM1	10.3823	10.501	10.7956	9.4064
CHKB	17.0899	19.3073	20.4133	16.4426
BIK	21.0961	26.2223	26.1586	26.1401
HMOX1	77.4747	59.2863	46.819	51.4297
MCAT	44.0836	39.0788	42.7523	40.8108
THOC5	43.7651	43.024	44.3359	43.0738
MCM5	236.712	232.73	230.228	213.191
ARSA	2.78007	4.47779	4.73114	3.34761
TSPO	155.983	168.851	181.348	159.935
TTLL12	91.6962	86.3012	98.6504	94.5465
CBX7	5.87892	7.46176	8.0261	6.8328

PDGFB	12.7741	7.70013	9.43731	9.63124
RPL3	1742.29	1880.61	1886.24	1785.19
ZMAT5	19.1599	19.371	19.6381	17.4218
RBFOX2	36.7962	30.3446	34.9842	37.4119
SYNGR1	14.0812	13.8358	16.0532	13.629
TAB1	14.1049	12.578	13.2763	12.5564
ASCC2	87.1425	58.0537	61.5757	69.1131
MTMR3	8.18067	8.56025	8.4686	8.04625
SMCR7L	31.6436	27.4299	30.5602	30.6551
PNPLA5	1.93604	1.61893	1.75378	1.68603
PNPLA3	1.11361	1.57026	1.78424	1.60759
MYH9	83.5861	85.8897	80.1587	87.7527
SAMM50	35.4262	36.9684	36.3974	38.1168
TXN2	134.958	122.586	135.746	130.488
FOXRED2	8.52764	8.7659	8.38415	8.22081
EIF3D	265.697	250.049	265.998	261.964
TNRC6B	3.7095	3.13843	2.5949	2.73421
SGSM3	38.5159	39.8185	42.6904	37.3232
IFT27	10.3448	12.5736	11.4986	10.9241
KIAA0930	52.5752	50.7763	51.8962	51.9311
SLC25A17	19.1808	15.7174	16.0713	17.9061
FAM118A	15.8717	18.0186	15.5608	17.5183
KCTD17	17.2963	18.4039	20.4253	19.8791
ST13	267.047	228.24	225.647	252.697
RBX1	39.2654	33.4539	33.2868	38.2663
EP300	8.00088	7.6647	7.63342	7.60028
L3MBTL2	20.4093	18.3378	19.3564	19.08
RANGAP1	106.93	91.1454	95.0131	93.4752
ZC3H7B	28.0649	24.5401	25.9364	25.8456

PHF5A	181.674	143.911	146.297	159.072
ACO2	50.2766	57.9803	61.5408	55.2969
POLR3H	59.2266	47.1652	52.1484	50.6318
TRMU	51.4861	40.0309	41.8517	43.4139
PMM1	19.8779	18.7506	20.2833	19.7433
DESI1	30.396	32.2259	30.6806	32.118
CERK	24.9371	28.8345	28.8124	28.1919
BRD1	10.8227	10.6788	10.8339	10.2845
ZBED4	10.2971	10.4683	10.8833	10.7097
HDAC10	23.2382	22.3373	26.4241	21.4938
ABHD4	7.23163	7.43164	8.3971	7.95818
KHNYN	13.2733	15.2886	16.8048	14.84
FKBP3	46.0914	46.3584	41.0097	46.4591
SDR39U1	42.838	42.5421	46.9375	40.0225
RBM23	35.0268	31.7987	33.7931	34.45
PRMT5	398.155	336.771	344.759	340.632
COCH	4.56681	3.73829	4.5451	4.05922
AP4S1	3.60939	3.58475	2.87787	3.89967
POLE2	35.7263	23.7523	22.6202	28.1321
METTL21D	5.85691	5.31072	5.51834	5.78655
SOS2	2.01621	2.19664	1.76311	2.33588
CDKL1	4.78964	4.52736	4.66591	2.97909
NIN	12.3425	11.6912	11.7966	12.2369
PYGL	104.273	107.415	114.09	111.078
TRIM9	1.26184	1.31504	1.34882	1.3074
PSMC6	85.3716	65.9776	68.6975	81.2575
GNPNAT1	32.0421	27.2434	31.0381	28.9496
DDHD1	9.95366	9.17126	10.1749	11.0163
CDKN3	78.0003	58.9372	52.7684	60.6628

CNIH	210.69	177.91	172.025	172.634
CGRRF1	10.0789	9.71383	8.33285	9.23835
ATP6V1D	52.6227	36.2587	35.2056	41.6181
PLEK2	51.0868	44.7869	44.8326	50.0525
PIGH	20.7907	17.2673	18.9559	20.0794
PSMA3	298.015	253.781	252.476	260.137
VTI1B	72.0247	71.033	70.2846	81.4049
TIMM9	35.652	30.081	29.5824	33.7802
GSTZ1	15.1965	22.1172	23.1978	21.4548
KIAA0586	6.58769	5.96108	5.75984	6.28241
TMED8	7.56809	6.787	6.78331	7.77028
AHSA1	406.797	338.494	323.731	344.512
DAAM1	4.73455	4.19746	3.0908	3.57947
SPTLC2	16.9239	17.1031	19.3658	20.0343
RIN3	10.0835	11.0868	11.8441	11.0072
LGMN	30.9529	28.3414	28.9494	27.0008
ALKBH1	13.8057	12.9801	12.919	14.9044
SNW1	68.1847	52.0587	56.5184	61.6528
ITPK1	59.835	50.9349	56.0834	51.1302
DHRS7	35.1519	34.9786	36.0623	35.7616
PPM1A	17.0933	17.7059	17.9039	18.2444
SIX4	6.04182	4.84211	5.1683	5.27246
CEP128	3.93845	2.44514	2.7244	3.22666
ERH	449.335	401.186	387.501	440.649
HIF1A	53.0746	41.4799	36.5473	39.2306
KIAA0247	2.89945	3.13869	2.63347	2.62022
SRSF5	184.622	161.343	158.239	168.545
EIF5	271.673	206.999	189.47	207.547
DICER1	16.0747	14.1317	12.8664	13.7383

ZFYVE21	28.268	31.5818	28.9894	27.9315
MTHFD1	76.8959	65.0321	69.2787	72.7514
ZC3H14	37.7608	34.0221	33.7597	37.9368
TELO2	50.826	49.1702	51.2069	45.4881
PCNX	11.8642	14.7497	13.859	16.1744
GSKIP	14.5449	16.2913	15.4142	17.1374
VRK1	33.9117	26.7029	28.8718	31.0673
PSMC1	155.424	107.254	116.294	133
PAPLN	1.27848	1.76625	1.59624	1.61228
RPS6KA5	4.72543	4.4851	3.16753	3.37238
SMEK1	41.9461	40.1275	41.5165	43.7784
C14orf93	3.2276	4.08971	4.17629	3.40947
PSMB5	356.32	305.241	324.188	313.681
YY1	60.8953	51.7136	52.1768	55.7419
ACIN1	87.8121	74.329	77.5171	76.6189
CCNB1IP1	44.4095	42.6136	38.7454	40.3582
TRIP11	5.15239	4.50422	3.78179	4.45648
APEX1	227.332	191.068	186.563	196.572
PABPN1	129.643	119.949	119.109	121.515
ARHGAP5	14.3397	14.0069	13.5761	15.3252
CINP	53.2985	47.8519	50.8604	48.863
DHRS2	9.03565	8.24856	6.35209	6.6018
SRP54	34.9731	32.2513	33.7982	37.7221
CHD8	24.0528	22.7593	21.0243	20.4637
PCK2	8.35829	32.8425	16.3147	11.0831
KIAA0391	26.8036	20.403	22.9665	24.4815
DCAF11	20.9658	23.8202	25.7966	24.8597
PSMA6	225.379	195.772	180.838	199.959
NFKBIA	18.6745	20.3206	17.2593	19.6688

EMC9	29.5695	27.6377	27.0761	25.979
PSME2	101.071	111.582	111.539	114.203
BRMS1L	4.77784	4.54311	5.51267	4.89046
REC8	1.59131	1.84927	1.59024	1.29347
TM9SF1	19.0453	17.9385	19.4686	18.4749
SEC23A	42.8663	50.933	49.3704	52.4084
GMPR2	94.1723	93.5754	90.7101	92.119
PNN	55.8871	43.0937	42.2555	45.2951
RABGGTA	29.2307	29.0859	31.9338	28.4522
NFATC4	1.15618	0.935067	1.43561	0.678186
PLTP	1.88636	2.57851	2.20649	1.82123
PCIF1	11.3648	13.8274	14.7796	13.2196
GSS	48.2521	57.361	58.2158	55.358
TRPC4AP	40.1219	40.2632	41.0528	39.009
PYGB	38.7302	43.3176	44.4042	38.8789
ABHD12	20.4952	20.4404	21.0464	19.6019
PROCR	59.8986	59.4385	61.7197	60.6647
GINS1	21.4736	12.9239	15.8197	18.6133
NINL	1.55341	2.36927	2.0599	1.6696
CD40	14.485	12.4786	12.9147	13.0159
UQCC	51.7051	48.5504	53.8159	48.5744
ZMYND8	5.27531	6.05464	5.66049	5.76414
IFT52	26.7834	23.161	23.2277	22.0063
MYBL2	74.7823	63.8475	63.2714	63.6509
HNF4A	1.95496	2.25641	2.11566	2.05589
NDRG3	20.1225	19.8487	20.253	20.7876
SLA2	1.83305	1.60571	1.36053	1.66109
C20orf24	133.167	117.579	116.793	123.439
PABPC1L	17.3872	27.9207	25.8521	20.5931

STK4	10.8964	10.877	10.6978	11.3803
ADNP	25.5617	24.4527	22.6565	24.3559
PFDN4	77.6241	65.7034	56.9402	62.4132
CSTF1	36.1372	31.3918	34.5693	33.9091
BMP7	8.0904	7.63908	6.42093	6.63845
RAE1	75.3587	65.691	65.6167	69.2324
TPD52L2	153.293	132.154	137.031	140.395
DNAJC5	26.1551	20.5295	22.6668	22.9974
NELFCD	75.9977	76.0809	75.9898	77.3652
CTSZ	92.2646	82.6721	87.7657	87.7734
PRPF6	43.1018	41.7825	43.4848	41.1525
SLMO2	114.986	97.8061	104.065	106.707
MTG2	48.2976	41.1561	38.6085	38.1531
PSMA7	453.045	404.633	416.973	407.832
SLCO4A1	44.6332	40.8037	40.6387	41.8429
NTSR1	68.916	100.979	104.494	93.1912
MRGBP	55.602	46.605	48.1933	50.673
TCFL5	9.33584	8.43196	8.26869	9.29982
DIDO1	15.6642	13.6745	14.0659	14.8147
GID8	37.3149	29.3933	31.6028	32.7321
SLC17A9	17.6589	14.3916	13.919	14.0203
ARFGAP1	56.8352	52.6609	62.0191	53.8815
EEF1A2	252.936	280.951	306.29	286.83
PTK6	1.83195	1.88881	2.44145	2.27796
GMEB2	7.7213	7.17836	7.59539	7.48207
C20orf27	127.545	114.731	121.887	119.469
CDC25B	94.3673	102.356	114.255	102.34
RNF24	2.02306	1.24784	1.60588	1.57003
ARFRP1	22.4769	23.3314	23.1632	22.7958

NDUFAF5	21.7632	17.5334	18.1244	18.6801
TRIB3	29.9677	48.7206	34.8451	29.2881
RASSF2	1.70739	1.35628	1.21724	1.3108
CSNK2A1	94.6908	84.8526	84.0546	95.44
SLC52A3	4.77346	6.49582	6.59638	6.37466
CDS2	25.913	27.0649	29.3169	26.7606
HM13	139.07	138.051	134.638	133.971
SNPH	2.0138	2.02564	2.03779	1.78067
SEC23B	71.8957	64.565	67.3774	68.0813
FERMT1	20.1533	16.6184	16.3506	17.6772
MYL9	20.2238	19.4793	18.168	18.1625
TM9SF4	27.7508	26.4678	27.5706	27.0394
CRNKL1	14.0066	12.7831	12.1709	13.2941
POFUT1	28.2298	25.7657	26.3735	24.8573
SAMHD1	9.21455	8.07475	8.07497	8.62715
KIF3B	15.9986	10.9627	10.3232	11.649
NOP56	622.321	506.047	490.011	512.767
MANBAL	57.2325	48.1173	49.8156	53.1767
IDH3B	130.509	125.46	130.124	122.705
MAPRE1	81.5166	78.9036	77.1694	87.1206
JAG1	7.39854	9.45893	9.73032	9.32572
CDK5RAP1	55.909	46.1306	47.4968	50.6494
SNTA1	8.83261	9.53696	9.93089	9.69337
TTI1	21.0799	18.0716	19.7034	18.8337
E2F1	23.5538	23.1291	25.3737	25.1993
RPRD1B	13.1866	12.3754	12.8949	13.0543
PXMP4	4.8555	4.75145	4.78406	5.10826
CHMP4B	55.4328	52.0998	58.014	55.8747
CST3	111.819	132.853	136.956	118.58

ACTR5	14.3531	14.7035	15.1441	14.76
AHCY	284.568	282.063	300.151	292.83
FAM83D	54.4432	45.7164	45.2859	47.5759
DHX35	11.312	10.4312	10.3348	10.2571
DNTTIP1	45.1366	43.1849	38.2274	39.6878
PIGU	28.0816	20.969	24.6632	25.2293
ACOT8	10.2069	10.988	11.8235	10.8739
APMAP	80.9832	75.4327	78.0582	83.5361
ZNF516	3.20606	4.9257	4.78138	5.17525
ADNP2	19.8487	16.3183	17.227	18.6782
RBFA	37.5748	33.2638	33.7213	34.6386
USP14	37.6749	29.3056	28.7675	32.92
VAPA	154.538	142.62	143.811	147.838
METTL4	6.02273	4.70619	3.55391	4.43281
LPIN2	3.08328	2.39039	2.79524	3.13141
SMCHD1	15.0777	14.1216	14.2074	15.4505
MYL12A	268.575	278.71	251.504	265.671
CEP76	13.5626	10.0707	11.058	10.7109
CEP192	12.044	11.8559	10.8803	10.7458
RNMT	15.6893	15.6316	15.3927	15.8063
SMAD7	5.38278	4.87526	3.65918	4.43169
RNF125	2.7114	2.60609	2.39015	2.62733
ANKRD12	1.8309	1.74586	1.79373	2.03304
POLI	6.1356	8.11692	7.94965	7.84393
MIB1	6.65731	5.89862	7.26277	8.02329
RBBP8	39.6164	37.4712	38.0602	40.6865
RIOK3	14.7104	15.5677	14.5076	16.6835
CSTF2	34.3319	27.5797	27.5316	27.7187
PSMD10	43.6473	38.1183	39.3649	43.9106

ATG4A	5.3434	2.36127	3.14215	4.16404
TBL1X	9.7076	10.0661	10.3954	10.1371
GPR143	0.993845	1.60622	1.30764	1.12046
PGRMC1	67.3532	55.5923	51.9529	56.9
POLA1	10.274	9.29658	8.86634	9.53197
MID1	17.1847	14.4332	17.6133	17.7614
NKAP	10.5204	9.91705	9.37571	9.93853
NXT2	8.52003	5.23599	6.04727	6.67887
RP3-324O17.4	12.0961	9.9299	9.56849	10.4509
ALG13	34.1838	31.0063	30.9682	30.8754
PRPS2	46.7012	37.6778	37.2614	40.7466
MOSPD1	33.8003	17.8183	24.1602	29.4941
AMMECR1	6.0326	4.93549	4.93547	6.00328
WDR13	16.5328	16.1705	19.2052	16.3298
SUV39H1	7.28892	5.91284	6.31402	6.301
SRPX	11.9928	10.8624	10.5254	10.1552
XIAP	12.4315	9.7184	12.3447	12.7807
STAG2	22.214	17.735	17.8773	19.8118
ATP11C	8.04737	7.3281	9.17405	9.247
ABCD1	1.2504	1.52165	1.66179	1.6431
CCDC22	3.00312	3.48984	3.36099	2.93108
SYP	0.283555	0.911176	1.01002	0.699524
PLP2	66.6564	57.6898	60.9033	59.2801
PLS3	23.8154	21.8301	22.5886	23.6412
NAA10	72.9411	63.1496	64.5451	62.2129
ELF4	13.3593	10.1145	11.8065	12.6377
SMARCA1	10.8045	7.96689	7.25498	9.05149
ASB9	1.30447	2.02318	1.46433	1.54942
RBBP7	103.51	87.5374	90.6203	100.722

SLC25A14	9.91139	8.21474	8.39448	9.31228
FMR1	5.75595	6.45137	5.78643	6.56022
PIM2	5.85545	6.17681	6.18302	6.02366
SCML2	3.70642	4.24685	4.34033	4.67789
SLC35A2	17.5138	14.5405	16.4526	16.6773
PQBP1	37.0697	29.5414	33.7042	31.2367
EMD	56.2266	53.3112	57.0153	59.5874
TAZ	8.34623	9.66477	9.4387	9.15968
PGK1	300.324	284.479	277.367	282.702
MAGT1	10.506	9.19394	9.43726	10.5325
SMS	134.983	117.621	124.907	131.336
UBL4A	37.7193	28.6303	31.2235	30.4376
CD99L2	1.53143	1.92249	1.95185	1.84946
EEA1	2.55473	1.70801	2.59236	2.76614
RP2	1.67345	1.68268	1.71028	1.86674
PHF16	2.24054	2.12158	2.36131	2.56514
CDK16	72.1497	73.7615	79.398	74.3087
USP11	22.8947	28.2082	29.3112	28.1907
HTATSF1	39.2018	34.0309	33.9916	39.527
TIMP1	219.67	208.707	221.268	210.754
FGD1	2.58712	2.27077	2.28805	2.38505
PIN4	15.6875	14.3858	15.3709	19.9634
PORCN	4.33866	4.26871	4.4637	4.81775
MAGED2	22.3192	28.5392	27.6728	28.2533
RBM3	148.17	135.532	130.182	139.038
SRPX2	1.42848	1.82952	2.03534	1.98843
SYTL4	2.4269	4.20351	3.45559	3.76447
CENPI	7.19606	5.74749	5.35376	7.04418
PBDC1	9.2814	8.03304	8.41075	9.23488

GLA	21.6641	17.1327	16.6471	17.1796
ARMCX3	3.84508	3.87866	3.43698	3.64282
NDFIP2	34.7757	30.9958	31.4355	34.8479
FNDC3A	8.73359	11.1163	10.0477	10.6269
CDADC1	6.05456	5.27034	4.26814	7.22019
CAB39L	2.24802	1.72654	1.24814	1.81668
KLF5	27.5022	21.1945	27.6265	27.7645
STK24	68.3986	58.4796	57.6926	62.3102
DNAJC3	10.1421	8.58741	9.27236	10.1754
UGGT2	4.5373	5.49275	4.9806	5.18732
ARHGEF7	14.1125	14.4988	15.0867	15.5573
FGF9	0.778204	1.40424	1.12607	1.38879
PARP4	19.2785	22.6082	21.3668	22.2909
SUPT20H	21.4265	23.1579	23.8169	23.7821
MRPS31	17.8013	15.7144	16.6045	17.8082
SLC25A15	19.0617	21.8639	23.7147	22.0491
KPNA3	34.0371	27.66	27.7521	30.8434
RGCC	2.8066	2.99828	2.60709	2.48188
VWA8	5.57324	6.34117	6.15607	6.63998
KATNAL1	7.60428	5.01312	5.13104	6.04452
INTS6	13.7652	11.627	12.1371	14.4789
DHRS12	1.21728	1.46236	1.60993	1.79885
TSC22D1	118.287	144.89	140.966	144.143
CLN5	15.9728	14.7433	13.5295	15.056
MSLN	17.371	20.7754	19.8317	18.2283
MGRN1	19.5541	22.9433	23.7322	22.0714
ZNF629	7.22148	7.89286	8.15268	7.95559
TRADD	11.608	12.4917	13.8648	13.0081
HSF4	11.8611	12.171	11.6318	10.6899

CORO1A	3.88959	3.7359	3.44799	2.86875
MAPK3	40.5595	39.7801	44.0833	42.0535
GDPD3	2.94739	1.12681	1.38159	1.28107
ELMO3	12.2524	14.3347	16.3382	14.523
PHKB	13.876	17.0314	16.9621	17.4382
LYRM1	14.3132	22.4935	18.0817	18.3429
NUTF2	160.15	160.476	159.858	164.712
NUP93	119.452	91.8692	97.6936	90.4659
CENPT	21.7068	20.1939	21.4759	18.8812
TSNAXIP1	1.54417	1.52325	1.92331	1.64357
NFAT5	6.12531	6.53662	7.53759	7.24431
LONP2	19.0561	18.3432	19.6805	19.7921
N4BP1	13.9198	14.3188	16.666	18.4957
ARL2BP	30.4994	28.9273	32.4477	35.3139
PLLP	2.01283	2.36182	2.85359	1.71496
DHODH	17.5774	14.3115	14.8025	15.6504
CTCF	24.3381	20.9958	21.4361	22.6474
ACD	54.8766	50.9648	51.5244	46.4854
POLR2C	102.379	93.1538	91.808	94.181
PARD6A	2.90725	4.12011	4.26376	3.85862
ZNF821	2.68368	1.74356	2.27667	1.76866
MMP15	20.2504	21.9483	24.9266	22.3254
USB1	76.4545	65.7212	73.7445	70.389
CYB5B	477.027	459.478	449.894	478.481
CCDC113	2.63717	3.37441	3.04059	3.38299
PRSS54	1.78743	1.86376	1.78314	1.70359
NME3	78.4657	82.0315	81.8207	80.4059
NDRG4	6.18179	7.2635	7.25285	7.91004
PSMD7	117.492	97.2888	98.6023	104.585

SETD6	23.137	22.6543	23.033	22.1806
SLC38A7	6.20009	7.25459	6.88227	5.94134
VAC14	26.694	24.1104	24.8576	22.7301
HAS3	47.7416	43.9082	51.6057	51.058
TANGO6	12.9258	10.1016	10.8569	12.0644
COG4	24.2884	22.3578	24.9708	22.5092
SLC7A6OS	23.2711	16.3648	17.4114	19.1364
SLC7A6	53.4714	38.8419	39.3559	38.0297
PLA2G15	7.40093	4.96338	6.1074	5.63294
ESRP2	32.3991	30.7469	32.6628	31.2827
FA2H	1.4596	1.55574	1.90283	1.61033
WDR59	21.4294	21.1849	24.3116	23.9497
MON1B	12.7386	12.8537	14.7516	13.0798
CMC2	128.608	105.36	102.781	111.807
AXIN1	10.5409	10.2491	11.0117	9.59336
HCFC1R1	93.9679	81.2501	82.1802	85.044
NPRL3	24.0365	22.1615	23.1472	22.4695
MLYCD	4.79185	5.72245	6.43345	5.59463
MPG	23.2771	23.4719	27.8221	27.6356
HSDL1	26.329	27.4311	26.8131	25.7729
TAF1C	42.2807	39.3215	43.6756	36.296
NAGPA	15.5431	15.71	14.3845	12.013
COTL1	264.485	229.043	246.021	253.192
USP10	112.331	102.739	104.217	110.145
CRISPLD2	1.29088	1.35502	1.64267	1.68727
TSC2	24.0629	24.2109	25.9529	22.8219
ZNF500	6.3325	6.78773	6.48231	6.37982
NME4	55.2022	58.389	59.4047	58.2016
ABCC1	29.9376	32.4119	33.9153	32.5501

NOMO3	70.6279	65.6992	75.9115	73.332
LMF1	2.90034	3.40667	3.55148	3.17409
FOXF1	1.50816	1.837	1.99115	1.90766
NARFL	24.0917	24.6283	27.7217	25.1803
MTHFSD	11.3727	12.5427	13.8259	11.6792
CLCN7	42.49	44.8909	43.1951	40.1246
HAGHL	27.2919	28.2335	29.7963	25.8099
FAM173A	50.5178	46.9887	52.2317	46.7834
SLC7A5	341.923	241.326	244.046	239.464
METRN	139.326	143.953	155.487	141.548
FBXO31	26.1959	26.9682	29.2186	27.4902
STUB1	86.7914	72.0644	80.2291	69.8142
RHBDL1	4.08585	4.10412	4.56108	3.73409
NUBP1	19.9472	18.0072	17.8296	19.0575
UBE2I	136.471	116.51	112.378	121.913
CRYM	1.80531	2.53578	2.23968	2.21946
EEF2K	9.00829	8.3602	9.04854	8.67766
CAPN15	26.3991	24.1172	28.4037	23.6739
PIEZO1	93.3808	94.8785	100.57	82.1984
GSPT1	88.0104	82.9237	81.4055	91.3575
ZNF174	5.2429	4.56428	4.60699	4.9557
CLUAP1	12.8568	13.0084	12.5457	14.9594
UBFD1	42.4359	51.2792	50.2265	49.4434
EARS2	37.1248	35.3094	27.2364	30.087
TCEB2	442.243	430.74	442.799	433.99
GGA2	58.989	55.1773	57.581	52.825
CPPED1	5.28224	5.52831	5.78922	5.91501
USP31	8.79148	7.60185	8.38404	8.47804
HMOX2	60.5969	55.8444	60.5589	61.6699

DNAJA3	64.9482	56.3956	62.7691	61.9363
BFAR	36.8953	35.8173	34.2311	36.5151
RRN3P2	1.98301	1.81441	1.56871	1.97893
RBL2	5.35372	5.47758	6.7082	6.13997
XYLT1	0.773779	1.16769	0.889052	0.838298
RPGRIP1L	4.02273	3.43038	3.17996	3.6159
MAZ	221.67	187.729	200.72	185.665
STX4	12.7384	12.5491	14.5509	13.986
CDIPT	61.4522	56.3046	61.0964	60.7039
BCKDK	44.9914	45.4142	49.9145	44.5277
KAT8	26.5476	24.8705	23.2706	23.9404
NOMO1	70.2321	66.908	74.6567	68.4884
SYT17	3.36564	2.89093	3.11775	2.75893
TMC5	4.61457	4.44556	4.61008	3.85864
CCP110	10.2711	7.05403	6.78525	8.43827
C16orf62	11.2825	9.73016	10.7424	11.0451
RNF40	84.9585	71.1158	77.9876	78.0685
KNOP1	10.5423	9.21824	10.343	10.5108
AAGAB	52.6714	43.3077	49.5563	53.7121
IQCH	1.58564	1.66863	1.71568	1.59974
LACTB	6.22042	5.91485	6.02598	6.78052
CORO2B	2.15883	2.12138	2.11636	2.16015
CSK	66.0259	59.1742	65.9632	58.7068
HERC1	9.94421	10.2539	11.3249	11.1964
TRIP4	12.0832	11.8836	11.0671	12.9015
MTFMT	24.5648	25.4361	26.5424	25.8232
ACSBG1	7.36627	6.40919	7.13133	7.66612
RAB11A	197.489	176.637	167.607	192.652
CTSH	15.398	17.8885	18.2923	17.8214

TTC23	3.23955	3.06263	3.18762	3.89258
CD276	58.3082	64.1403	69.7257	64.4704
FAH	8.60195	12.0143	11.49	11.1854
KIAA1199	1.52796	1.78073	1.9887	1.549
RPAP1	20.1578	15.6521	18.7052	17.5519
EHD4	32.3058	28.1438	31.9393	31.2921
TMEM87A	20.3235	20.0368	18.0411	21.0419
ZNF106	8.13917	6.13488	6.56005	7.14528
CEP152	3.27278	2.62401	2.65171	2.78668
DTWD1	23.7611	24.2982	19.0058	24.0113
GABPB1	18.7508	15.6085	14.8251	14.3855
TJP1	28.8709	28.8913	23.6485	26.348
DMXL2	4.7267	5.58489	5.47589	7.00177
DNAJC17	16.0875	15.1635	17.3462	14.1474
EIF3J	75.1162	62.6201	55.7114	60.0621
SPG11	11.0666	12.3726	12.9377	11.4959
RHOV	3.08527	2.29241	2.40763	2.47
VPS18	13.413	13.1227	14.4944	14.1572
OIP5	28.3956	25.4802	23.5122	22.9157
SLC30A4	1.09338	0.847729	0.871675	1.10056
BLOC1S6	21.6491	21.77	19.9722	22.6163
SGK3	5.57085	4.28093	4.74791	5.35124
PDGFRL	3.32848	3.25312	3.21493	3.50459
CSPP1	11.0318	8.14957	9.76656	9.79885
ZDHHC2	27.9514	24.5204	25.5979	23.0663
BRF2	12.2713	11.2679	11.2228	10.9883
TRIM35	18.0578	12.4871	13.858	11.8423
ZFAND1	68.9897	57.6001	55.7395	60.4481
CA2	22.1104	19.8449	19.3623	20.1423

FZD3	4.08954	3.84022	3.7994	3.78669
INTS9	13.3249	13.0478	12.8709	12.7515
RIPK2	21.1285	20.8998	21.4473	22.8195
NBN	15.8163	10.853	9.62152	11.634
DECR1	28.3465	35.5473	35.4228	38.1914
CALB1	1.21121	0.811821	1.32718	1.64268
IMPAD1	29.4823	23.2835	24.1442	26.8213
LAPTM4B	302.965	253.947	236.487	248.123
UBE2W	19.5489	19.4378	18.953	20.1372
POP1	38.5947	25.7887	27.5841	29.3845
NIPAL2	2.74795	3.33249	4.17648	2.45115
IKBKB	15.2056	16.5982	18.4474	19.1197
PLAT	4.63764	3.99564	4.08891	4.04873
JPH1	7.81913	6.66726	7.76123	8.07587
STK3	15.1583	11.5346	13.5744	13.6577
GDAP1	11.8289	8.12671	9.25344	11.0706
RAB2A	68.9732	59.8193	65.4056	74.889
EIF3E	500.623	461.811	434.732	484.44
EMC2	42.0487	35.0219	34.0795	33.1422
ESRP1	28.3955	29.6571	28.8516	30.6535
NDRG1	35.8081	45.1135	44.4594	36.5229
ZC2HC1A	4.68293	5.12768	4.59315	6.04803
ARMC1	31.9971	27.5198	28.2394	29.7013
SPAG1	6.8455	3.39399	3.80877	4.54938
CHRAC1	51.8971	44.1781	41.6051	47.2664
SNX16	3.24233	2.3897	2.57098	3.02767
UBR5	39.057	39.5069	38.7815	43.7646
GSDMD	43.4715	47.1228	49.4503	45.5607
TSTA3	120.104	111.135	109.408	109.171

PYCRL	16.3028	20.4413	19.7968	17.9902
EEF1D	1043.16	977.138	970.222	946.349
SQLE	33.2785	24.3622	23.9442	27.0862
SH2D4A	16.9653	15.0644	15.2972	16.3677
INTS10	71.3499	67.8921	65.1992	73.6816
ERI1	28.4503	22.9025	25.2609	28.8138
SLC39A14	53.5564	37.2835	40.9957	46.1741
MTMR9	3.53327	3.2093	3.64311	3.81278
LEPROTL1	46.9163	43.3545	43.5531	49.0333
DCTN6	42.7464	40.0493	40.5357	44.2765
R3HCC1	34.6043	28.3405	31.6558	31.9403
GSR	132.221	118.09	125.278	129.557
TNFRSF10A	27.3114	29.6465	30.2622	29.2927
UBXN8	15.8269	15.1867	16.6102	14.8224
PPP2CB	99.3747	89.3325	90.7838	98.3014
ERICH1	10.0149	9.76355	10.4394	10.2195
ARHGEF10	19.756	22.2586	23.7137	19.751
KLHDC4	48.4149	47.588	47.3266	47.8237
MCM4	380.395	239.936	274.074	293.065
KCTD9	27.8144	24.0817	23.3984	25.0362
ASAH1	44.9589	46.6624	47.5153	49.8318
BNIP3L	9.52258	10.7446	8.99418	10.2539
MAN2B1	14.969	17.9444	18.3759	17.1363
KCNN4	30.6719	34.6839	37.5493	36.0447
NUCB1	39.1948	42.2835	42.3613	38.345
DHDH	4.15137	3.94288	4.06844	3.73261
GYS1	26.4415	28.2869	30.2712	30.2867
MAP4K1	5.01761	4.26297	4.28627	5.11909
CGB2	2.10676	2.38068	2.42928	1.91063

ECH1	47.0613	49.5356	52.037	42.9032
HNRNPL	430.75	351.366	342.562	393.076
NFKBIB	55.7132	41.5101	48.9841	46.5361
LHB	0.537441	1.19183	1.22235	1.13568
TUBB4A	14.3716	17.0631	17.0983	17.1356
SARS2	39.5054	42.5522	46.0161	38.3365
SNRNP70	194.871	165.693	178.553	176.076
CLPTM1	81.0812	75.6428	80.409	72.9285
RELB	9.97199	10.7014	11.6907	11.1615
CLASRP	31.776	32.0932	30.9006	30.857
LIN7B	3.94821	3.31943	3.76331	3.82169
PPP1R37	15.1457	16.0062	16.2943	15.1695
PIH1D1	64.319	65.6157	64.3591	64.2632
ARHGEF18	13.9349	13.9854	13.8741	13.9488
PPP1R13L	22.1712	20.597	21.0428	22.7437
PEX11G	1.09216	2.32541	3.01509	1.90495
ERCC2	32.4893	37.3431	35.4709	32.7539
DOT1L	18.2083	11.8299	17.0901	14.1014
PLEKHJ1	109.962	105.255	114.096	107.656
RNASEH2A	70.4336	65.926	68.1293	68.4161
KLC3	12.7426	11.6813	12.4394	11.5594
CD37	1.37002	1.75339	1.359	1.56569
SF3A2	100.126	92.9996	92.4118	83.0245
AMH	10.7561	8.61193	9.55475	7.68493
OAZ1	993.492	932.457	924.376	891.301
TRMT1	110.532	96.2371	100.975	92.4626
STX10	127.832	113.644	125.478	112.944
DMPK	13.6621	12.4086	12.3867	13.551
TBC1D17	9.74361	12.9251	13.4613	12.2165

TLE6	2.69533	3.66137	3.94651	3.3924
CCDC130	10.5756	11.8378	11.7624	10.2807
PTOV1	104.91	104.768	108.89	96.7099
AES	359.961	391.347	399.279	394.589
SGTA	117.874	102.821	109.568	109.389
MED25	36.5796	39.1648	41.3688	34.7521
SNAPC2	31.76	35.3076	28.3536	27.9181
C19orf53	217.144	189.794	187.381	188.318
TIMM44	82.3597	90.8581	93.7314	83.9533
CCDC61	2.79765	3.01063	3.46637	2.53711
IL27RA	13.1852	12.2871	13.0034	12.7802
ASF1B	66.104	56.3849	60.5445	61.8789
TNNT1	124.593	124.166	133.78	138.062
VRK3	22.2105	21.7276	20.0707	20.377
FAM32A	68.9193	61.4468	62.8585	65.3517
PPP6R1	59.3393	59.9485	64.057	58.4616
C19orf44	5.45348	3.75054	4.1918	3.30591
MED26	7.12454	5.8846	5.90687	5.96621
OLFM2	4.79463	13.9713	16.6703	12.5735
AKAP8	24.4404	19.8304	21.6517	19.1252
ILVBL	47.0865	45.6078	47.9418	41.4898
SYDE1	7.34139	8.10316	7.40532	6.26612
POP4	89.7623	77.8801	78.6492	79.6561
CCNE1	40.2105	40.5791	44.1779	46.3547
URI1	33.3526	24.7151	27.2775	30.3516
PDCD5	186.654	169.084	158.654	181.666
ANKRD27	20.5449	16.7455	18.6254	19.5861
RPS16	2155.67	2125.98	2024.17	2046.86
TIMM50	228.636	189.191	207.924	222.024

FBL	694.485	630.882	632.479	619.068
DYRK1B	2.5041	2.97397	2.70581	2.73055
CNTD2	4.29406	4.90462	5.03721	4.81942
GPI	370.639	358.403	361.907	352.042
AKT2	68.6569	76.5215	88.4514	74.1859
PLD3	72.1919	86.1844	85.2959	77.7571
PRX	1.14262	1.2736	1.25252	1.20911
PIAS4	51.2428	53.3807	57.4004	60.6003
NUMBL	18.8631	21.0533	22.1329	21.8135
CCDC94	28.7062	24.435	25.5127	24.5098
SHD	1.22735	1.00541	1.07444	0.688092
TBCB	117.433	112.296	115.725	110.773
FSD1	16.8313	13.5359	13.5581	15.0174
POLR2I	128.901	120.963	129.941	122.046
SLC1A5	162.219	206.146	170.552	158.256
PRKD2	31.6757	23.9154	26.4853	26.6912
TJP3	6.02071	7.81722	9.258	7.49458
APLP1	20.0866	18.3758	17.5504	18.5751
CACTIN	27.8372	23.1833	26.3648	23.5483
CCDC9	14.4913	13.6771	13.0394	13.8296
HNRNPUL1	90.5725	80.0516	80.9895	85.1891
FZR1	46.3176	41.6181	42.6894	43.9104
BBC3	8.71079	19.624	15.3027	10.9965
TGFB1	40.0135	35.1616	38.1155	35.7592
DENND3	4.48133	4.39545	5.37531	5.36115
ATP5SL	59.2741	53.1471	50.2522	52.0422
PLIN3	137.829	137.877	157.592	142.78
MYH14	8.93264	11.2401	11.67	10.664
MRPL4	128.558	120.378	126.564	114.729

RPS19	2433.72	2563.09	2309.32	2542.42
GLTSCR2	183.954	210.538	187.738	186.719
ICAM5	12.6524	13.5024	15.3791	13.4295
ETFB	123.512	108.606	117.271	115.06
CD33	1.94738	2.42299	2.48973	2.33091
BABAM1	93.7644	98.4177	92.2137	91.444
TYK2	49.1635	49.0099	50.0544	44.4387
CDC37	281.132	261.021	258.432	251.604
NAPA	59.2171	56.1341	61.8269	56.3948
RABAC1	46.9991	55.5782	56.7905	50.9531
ATP1A3	11.3708	11.6286	13.1542	12.7674
MEIS3	10.1295	14.7469	14.3603	13.2479
PTPRS	20.8496	21.7304	24.1252	22.0337
CNFN	7.94891	9.67979	9.43736	8.93614
MEGF8	6.22947	9.1685	9.80004	9.42858
KDELR1	220.761	174.061	195.92	189.131
CYTH2	18.1219	20.733	18.4707	16.6888
GRWD1	70.8135	57.6653	63.6432	64.3654
GRIN2D	8.34131	7.92295	9.79389	8.99415
CLEC11A	4.26134	4.87668	4.68833	4.52309
CARD8	7.97626	7.55667	6.52375	6.12649
LIG1	61.3487	62.7276	54.2613	60.6476
ZNF175	2.19938	2.33717	2.65904	2.28626
PLA2G4C	0.615611	1.01903	0.687046	0.497957
RAB3D	5.32743	6.61844	6.41452	6.01726
DBP	6.48001	8.28401	8.22412	7.66397
TMEM205	45.4394	47.1259	48.3727	46.5503
DKFZP761J1410	16.2575	14.9983	18.285	17.7337
BCAT2	46.4484	52.7925	58.4785	52.2979

MIER2	11.9099	12.5091	12.457	11.6009
PLEKHA4	6.1327	5.26971	6.24064	5.09475
PPP2R1A	321.917	317.219	321.72	293.184
TNPO2	50.5531	48.9559	50.3034	45.4395
WDR83OS	192.41	192.809	193.05	191.33
GCDH	17.9092	16.8823	18.3531	16.7536
DNASE2	17.4988	21.2017	19.7	19.4359
MAST1	10.2469	10.742	12.3634	11.8451
LENG1	1.06751	1.0984	0.845537	0.673606
PRPF31	13.8099	12.7052	11.4944	9.55367
RPL18A	1947.34	2134.19	2119.55	1933.29
ARRDC2	17.8423	17.0857	18.4033	17.4797
PIK3R2	16.4349	13.9314	16.7163	14.93
RAB3A	7.53119	6.50056	8.14005	6.00655
ISYNA1	117.883	130.153	130.401	115.255
ELL	8.81369	7.45852	7.91357	7.5311
CRTC1	3.72575	4.44565	4.9126	4.66022
KMT2B	9.78582	10.8719	10.6168	10.2114
COPE	223.746	213.605	217.346	190.438
DDX49	79.8846	77.7221	89.8585	86.9047
ETV2	1.07976	1.66249	1.93474	1.21491
ARMC6	51.7398	42.7038	46.5476	47.7757
TMEM147	211.989	182.076	199.482	183.919
TMEM59L	7.48288	8.56464	8.1702	6.78996
USF2	84.1329	79.5047	80.8159	73.9762
LSR	189.765	191.001	199.638	181.636
KXD1	106.204	93.111	98.4736	105.813
FKBP8	140.73	146.984	156.172	137.485
SUGP1	30.4873	32.3102	30.4446	29.8467

ZNF14	2.07481	1.91244	1.77237	1.77966
SCN1B	5.77592	5.61414	5.13003	4.70772
PBX4	5.04198	4.4622	5.38643	4.43542
ERF	63.8431	54.3467	57.2512	59.3548
GSK3A	65.8531	61.0878	65.6521	60.5883
ATP13A1	38.9328	42.0574	43.0153	41.0636
ZNF574	14.0474	13.0447	14.0783	12.7522
SIPA1L3	16.7976	17.6106	18.129	19.2947
ZNF85	1.05598	0.817496	1.00298	1.36659
ETHE1	51.9517	56.098	59.5032	55.4893
CADM4	27.9101	25.7284	30.8507	25.4982
SMG9	38.968	37.0904	41.7966	34.3467
AVL9	20.852	19.3736	18.9942	21.8971
RUNDC3B	0.879402	0.727959	0.885435	1.17285
GTPBP10	18.8936	18.9036	17.2973	20.3162
RASA4	1.83262	1.91731	2.15175	1.60549
CDK6	13.9463	11.9363	10.3303	11.8775
PMPCB	61.4912	59.9062	60.0236	61.8871
DNAJC2	34.0944	27.4547	27.2904	28.6184
BET1	11.7392	9.80375	8.15779	10.3434
NAMPT	70.8994	57.6667	59.7958	66.4452
TWISTNB	18.0577	11.8098	11.5858	12.9222
PON3	1.3609	1.375	1.20088	1.07004
PON2	30.0672	32.5434	29.425	30.8165
HBP1	5.90848	4.69989	4.7047	4.36855
DUS4L	6.22349	6.00554	6.1303	6.11639
SP4	2.49596	2.23699	2.67723	2.24276
WDR91	4.63936	5.11188	5.76744	5.96093
CBLL1	21.6307	20.3269	20.8464	22.797

MTPN	73.1838	59.3979	65.5256	69.2377
MPP6	14.4738	10.915	12.4203	14.6991
DFNA5	3.53225	4.6869	4.53522	4.51126
ZC3HAV1	9.29296	10.3476	10.009	10.5687
TTC26	6.34509	4.17308	4.0135	4.59287
OGDH	24.8869	22.7342	27.7413	27.0467
ADAP1	18.4222	15.5628	20.4708	17.7786
H2AFV	160.002	145.681	142.198	143.531
CAV2	216.942	190.484	193.788	188.085
CAV1	299.532	244.032	268.602	291.769
MET	53.9791	56.6651	58.6231	61.8311
RNF32	2.31074	2.6688	2.60157	3.20917
LMBR1	22.1211	23.4657	19.7289	23.2148
HOXA1	1.26841	1.80143	1.72906	1.61483
DNAJB6	74.8828	62.9547	62.7848	70.9946
HOXA3	5.11047	3.77115	3.17074	3.81356
LFNG	6.6662	6.90813	7.33899	6.78088
HOXA5	5.25969	5.52183	4.83999	6.08032
HOXA6	4.29522	4.04459	3.89106	2.82294
BRAT1	40.9413	41.3095	43.303	38.8672
IQCE	10.0217	8.26593	8.19464	8.1847
TSPAN12	3.55471	3.03104	3.04866	3.94067
SSBP1	171.276	146.417	144.538	156.823
HOXA13	1.62247	1.12716	1.32962	1.57626
HIBADH	15.2067	9.19271	14.5582	15.7899
TAX1BP1	31.2594	29.3329	30.3856	33.2916
CPVL	9.09473	10.319	10.0701	11.4595
CHN2	1.29908	0.935343	0.742934	1.09837
GRB10	8.84793	10.3812	7.68765	8.38356

ABHD11	28.6721	25.3304	26.5695	24.017
COBL	6.26615	6.84237	6.09773	6.9116
FKBP14	3.9875	2.90703	2.94796	3.03572
PLEKHA8	5.53794	4.72955	4.42027	4.57525
STX1A	8.19476	6.42636	6.35784	5.79426
NOD1	2.58286	2.12736	2.07395	2.14666
GARS	157.745	212.608	183.353	173.93
EPHB6	2.18339	2.86634	2.73184	2.24785
FAM188B	2.67638	2.72719	2.8833	2.78238
NSUN5P2	12.3471	12.0673	11.0491	10.8515
CASP2	23.7541	19.9029	16.3498	18.4528
CHCHD2	478.927	389.138	399.571	401.681
HSPB1	569.647	491.331	486.698	478.322
PDAP1	187.775	179.748	176.273	183.804
BUD31	72.6739	73.0238	82.2131	72.7341
PTCD1	16.2632	15.3653	16.1422	15.7185
ZKSCAN1	5.51232	6.70446	6.18316	6.07978
EIF3B	247.143	220.226	217.753	234.794
SNX8	66.5388	64.1029	68.1165	61.8581
NUDT1	60.6224	51.7968	51.1812	55.231
TAF6	37.6922	39.338	39.8353	37.8365
WASL	10.2406	9.57152	9.93835	10.4726
AIMP2	34.9231	39.3375	37.9853	39.7285
TFR2	6.54177	5.69689	6.14873	5.67259
MOSPD3	17.9739	22.1323	23.8307	21.4014
PCOLCE	4.24346	3.46329	4.97687	5.07459
RBM28	23.3568	18.0703	20.3836	20.9692
USP42	5.0712	5.00648	5.04657	5.12138
IMPDH1	93.4473	79.5415	88.041	81.7157

AGFG2	9.80943	8.23422	9.6511	9.50406
LSM5	141.458	124.878	116.647	143.792
SERPINE1	13.992	17.6282	12.632	13.4584
AP1S1	149.628	146.711	155.571	158.365
C1GALT1	14.7811	13.7869	13.5586	14.0675
PLOD3	53.9935	54.1921	55.4452	51.1755
RPA3	66.8366	66.8157	55.8881	65.6667
ZNHIT1	41.8263	43.7182	43.6638	45.8708
CLDN15	5.87926	6.89319	6.15537	5.37021
GLCCI1	1.38335	0.620179	0.704515	0.768181
PHF14	3.67254	2.67403	3.19196	3.85087
NRF1	16.7398	13.1123	13.5471	13.8518
TMEM106B	17.2682	15.284	15.2418	16.548
EZH2	34.7719	28.1064	27.6465	29.9086
CEP41	3.10312	3.81966	2.65394	2.77089
ZNF862	1.14196	1.42111	1.59954	1.86476
MEST	28.6935	28.6219	27.4917	25.5667
ANKMY2	12.74	13.6326	13.9106	15.0053
TSPAN13	13.3964	12.4438	11.3865	12.7642
AHR	7.52946	6.64257	7.07799	7.19452
CHCHD3	71.6862	60.8552	58.96	57.6168
PSMA2	216.274	213.121	204.108	227.554
MRPL32	62.4186	50.0114	53.5329	58.938
COA1	71.1887	66.6203	67.3998	69.5692
BLVRA	22.234	22.0419	24.4886	26.5079
URGCP	13.525	12.7794	14.426	13.1202
TMEM248	47.9902	43.7185	45.7998	46.1142
STAG3L4	11.7055	10.0394	11.3121	11.1928
RHEB	101.807	84.2862	81.5103	93.4025

PRKAG2	22.6849	18.2473	21.2611	21.6697
POLD2	284.475	271.433	270.562	265.158
BCL7B	29.0544	25.4299	25.9555	24.1029
YKT6	83.0313	73.4388	78.4608	79.7151
TBL2	18.6476	17.6668	16.3733	14.2409
CLIP2	7.02486	7.19994	6.90449	6.24895
EIF4H	165.357	137.017	134.612	141.374
LIMK1	18.3871	16.2367	20.4739	18.8285
SLC1A1	2.76671	3.34129	3.35536	3.89535
LHX2	8.63807	7.329	8.52722	8.54089
FKTN	4.2012	2.37338	2.79853	3.24441
FSD1L	1.93354	1.43246	1.70813	1.66641
CNTNAP3	2.42662	3.01986	2.87064	2.72427
SPIN1	24.6997	25.0137	24.1968	26.9413
NMRK1	2.79085	2.96857	3.41278	2.87785
TMEM245	15.7461	14.0595	14.5888	15.3426
MEGF9	2.39298	1.52862	1.81781	2.18669
TRIM14	29.7157	25.2483	25.059	25.2317
CORO2A	2.60635	2.71465	3.00121	3.1522
TGFBR1	8.69129	8.3067	8.77923	9.09566
SEC61B	171.141	120.429	126.732	139.392
LHX6	2.04117	1.57932	1.59414	1.7717
PTGR1	28.2407	29.7706	27.503	30.0836
SUSD1	3.58701	4.25121	4.70137	4.31043
AKNA	3.643	3.83357	3.43023	3.53884
DNM1	0.890933	1.29832	1.20951	1.25692
AK1	59.366	59.5243	64.7257	66.9303
CDC37L1	10.3151	10.2094	10.4873	9.98397
PLGRKT	13.7289	15.6094	14.3121	16.9378

TBC1D13	10.1871	10.2099	11.1171	10.7609
KIAA1432	9.84771	10.4381	10.8291	10.4684
KDM4C	5.62038	4.33675	4.72905	5.9248
KANK1	5.06156	6.01072	5.84621	5.45888
NCS1	20.6871	14.3989	15.3306	15.2599
TESK1	12.8192	13.872	13.8746	13.1971
FUBP3	84.7443	66.1692	78.2923	68.2834
CREB3	25.8681	22.863	25.1903	25.0455
RGP1	11.5156	11.8439	11.6764	12.1951
DDX58	1.73263	1.99353	2.11605	2.10394
EDF1	276.623	285.353	266.763	262.969
GLIS3	0.856414	0.895179	1.04021	0.847365
BAG1	38.672	36.7482	45.3898	40.1942
RAPGEF1	20.4635	17.779	21.7055	22.078
NPDC1	51.6191	59.7903	67.8235	58.4832
SETX	14.401	13.6736	14.1201	14.5805
ABCA2	34.7614	34.6624	40.4617	30.1581
SHB	19.7445	15.8025	17.375	16.5976
UBE2R2	18.4874	19.0378	18.3775	20.4344
ABHD17B	9.1892	8.7199	9.42167	9.83295
EXOSC3	117.199	101.612	99.7015	116.815
ZFAND5	50.2336	42.4793	43.159	48.2444
DVL1	83.5022	70.9753	82.1632	74.8502
PDLIM1	52.3646	49.9624	50.1807	52.5423
CCNJ	11.6025	13.1278	12.076	12.1675
GATA3	1.35357	1.18074	1.22177	1.19857
HPS1	46.4451	39.6437	42.1532	41.9533
PHYH	6.00477	7.91217	6.92984	7.11766
RASSF4	1.00676	1.08258	1.25471	1.03634

DNMBP	20.5319	15.167	15.9733	17.8065
RAB11FIP2	7.79976	8.14763	7.65468	5.17956
ERLIN1	45.8027	41.8566	40.8447	45.2045
EIF3A	131.586	112.877	120.46	126.104
TRDMT1	5.01838	3.86886	4.03006	3.90304
DDX50	14.9496	14.8793	15.0899	16.7731
MAPK8	34.6298	33.3363	32.6063	34.788
SEC23IP	58.2622	54.1149	53.2809	60.2621
ATE1	14.5511	13.5663	12.0368	13.468
NSMCE4A	57.9714	50.3464	48.5428	52.0975
PLEKHA1	15.8928	14.8431	16.2169	16.3432
PALD1	24.7131	26.5177	28.3465	26.2112
UNC5B	6.45226	7.84825	7.50385	7.05567
C10orf54	21.3493	17.782	25.2424	21.682
MICU1	18.5593	15.3386	17.6181	18.7229
PPP3CB	19.7979	18.0608	16.8804	18.4784
CCSER2	6.51519	5.83302	5.94489	7.09038
BMPR1A	13.719	13.8828	12.971	13.8017
MINPP1	15.7314	15.1141	14.568	14.7384
ACTA2	1.85149	6.74113	3.72487	2.96498
LIPA	12.3133	15.7066	14.6505	15.6592
C10orf2	44.5306	38.8243	39.0119	40.0382
LZTS2	47.0087	44.4919	48.6281	44.8936
SFXN3	18.0447	16.3267	17.6346	17.3824
KAZALD1	2.82181	2.95331	3.66228	3.51597
FBXW4	12.9489	19.6912	18.2518	17.7999
NPM3	538.204	509.486	537.021	489.275
TNKS2	7.98341	8.72064	8.89257	9.18861
PITX3	2.20413	2.05521	2.12054	1.66123

GBF1	23.8762	24.6677	26.07	24.6094
ARHGAP21	15.6004	11.827	11.1743	10.8336
FBXL15	16.808	19.2874	22.0911	18.247
CUEDC2	82.4931	85.002	85.2447	82.7491
SUFU	8.13864	8.07422	7.85939	8.02885
ANKRD26	1.28768	1.10831	1.38953	1.25664
ACBD5	9.00681	9.38631	10.536	10.9556
LHPP	14.3074	17.3523	16.8161	16.7125
LARP4B	43.6951	36.9389	40.415	40.0833
GTPBP4	91.9587	75.0703	82.9615	84.8755
C10orf137	21.6672	22.4714	21.9459	24.4395
BCCIP	139.171	108.033	97.7549	117.589
MTPAP	17.4794	18.2844	16.6238	18.2188
NEURL	1.50588	1.26274	1.61469	1.54612
SH3PXD2A	2.28931	1.82486	2.01431	1.7972
PITRM1	32.8722	28.5792	29.7565	30.5491
OBFC1	17.224	20.6424	21.9119	21.1699
MAP3K8	1.79964	1.26538	1.39933	1.358
DKK1	361.615	224.211	246.604	284.829
GLRX3	66.5781	55.6475	50.8095	63.2968
FAM208B	25.621	22.7299	22.2779	25.8669
XPNPEP1	104.078	84.7863	107.068	104.506
SMC3	23.1197	18.6766	18.8533	21.6605
SHOC2	12.8868	12.6811	11.906	12.7857
TFAM	36.1646	28.6087	30.0591	35.2345
CCDC6	19.6248	18.4857	19.1684	20.0253
CUL2	26.6354	22.2249	20.4396	23.5728
CCNY	39.5576	44.1796	47.4435	44.1957
UBE2S	598.471	464.381	484.736	487.023

RPL28	1477.13	1540.2	1557.92	1553.38
ZMIZ1	7.86408	7.98009	8.26439	8.4274
DNAJC12	1.42532	1.51242	1.31974	1.76345
PPIF	147.857	109.079	111.856	114.657
PBLD	1.05921	0.952753	0.68501	1.0639
TSPAN14	57.1088	54.6838	59.2009	56.2728
TBC1D12	2.67614	2.07817	2.74996	2.80842
KRT23	3.23397	4.18058	4.56753	4.07555
NUFIP2	22.4639	17.5083	18.2814	24.4672
GIT1	39.8457	38.8817	40.9934	39.4318
TADA2A	13.5383	14.7078	13.5535	14.255
AATF	70.9991	65.8297	71.0903	73.186
DHRS11	9.59244	7.2569	8.42179	8.11698
ZNHIT3	23.0947	21.7353	17.8698	20.7619
MLLT6	21.4507	22.8394	24.5189	23.0176
PSMB3	586.006	560.23	541.627	544.567
CWC25	10.7452	8.68112	9.5589	9.89582
RPL19	2151.98	2133.12	2106.45	2118.48
FBXL20	1.87259	2.58625	2.80222	2.44139
RUNDC3A	0.561299	1.26074	1.65982	0.997858
UBTF	56.8364	48.2782	49.509	51.1428
PSMD3	195.939	174.272	176.409	178.953
CASC3	32.7399	30.0994	30.5036	30.0988
RAPGEFL1	2.93346	3.44262	3.56908	3.22472
RNF43	18.2521	21.3977	19.9412	20.1277
WNT3	1.74995	1.68755	1.80213	1.67502
RAD51C	98.1421	80.6893	71.0225	82.9781
4-Sep	1.23058	2.17418	2.20792	1.74746
MTMR4	24.2935	23.6484	22.5539	23.9026

TRIM37	28.1732	27.8224	24.8436	27.5642
DHX40	27.0647	26.8293	22.6557	25.5454
TUBD1	14.1368	10.4919	10.3605	11.7082
KPNB1	390.626	321.379	325.411	350.458
GOSR2	55.7925	46.82	51.2233	52.0863
PNPO	35.9866	38.4936	33.5822	34.8885
RPS6KB1	30.3773	20.5357	22.3057	24.5943
TRIM16L	18.2038	15.5719	13.3583	13.1617
ZNF29P	0	0	1.79462	0
CDK5RAP3	58.5892	67.2212	70.9603	64.0117
CBX1	104.959	99.4278	96.4079	99.7293
RECQL5	19.8559	29.7552	24.9033	23.4369
PIGL	20.3142	17.1971	17.7881	16.9434
GALK1	32.1538	34.1133	37.3703	30.98
INTS2	10.6649	10.4433	11.2782	11.8607
CAMTA2	23.0966	20.0953	30.655	24.965
MED13	14.9238	15.4539	14.2417	16.4254
HOXB6	13.6273	14.7886	14.9084	13.304
ENO3	55.0797	51.0912	53.7826	51.2642
PFN1	1497.07	1305.73	1376.66	1376.53
RNF167	100.79	92.7211	93.656	103.274
SLC25A11	74.6107	74.6777	81.861	80.4161
RAI1	9.65234	9.25833	9.58885	10.2095
NUP88	121.425	99.8472	103.86	110.048
C1QBP	614.663	579.674	563.744	576.315
BLMH	51.93	39.0774	41.7459	39.881
CPD	19.2541	20.5874	18.8159	21.0874
GOSR1	19.5389	16.055	19.5207	20.2524
CCDC47	80.7903	54.2611	62.4706	66.1456

MED31	22.1916	23.1611	20.4071	25.6695
DRG2	31.5841	29.8896	31.0783	27.2617
FTSJ3	67.806	59.2988	61.4644	58.244
AKAP10	12.061	11.7152	9.68754	10.8982
ALDH3A1	0.818784	1.15817	1.00305	0.746939
SMARCD2	109.73	104.107	103.691	103.589
SYNGR2	196.381	187.717	209.009	189.862
B9D1	15.2257	19.8377	17.3042	15.0381
UTP6	63.297	53.3452	54.4656	70.8473
DDX5	252.74	240.182	212.969	235.6
C17orf75	15.4365	15.3143	13.7638	14.5397
CYTH1	11.8485	11.3387	12.4191	11.9141
PSMD11	110.765	103.252	97.0032	112.97
LGALS3BP	197.566	245.793	241.523	217.958
PEX12	4.63104	4.04494	4.69426	4.8522
HNF1B	1.76551	1.80884	1.95275	1.94726
KRT32	0.959313	1.29672	1.70939	1.39353
KAT2A	23.9391	20.2061	20.4414	19.2276
RAB5C	159.553	153.35	171.545	157.415
NAGLU	9.92734	13.7046	14.34	12.2239
HSD17B1	0.949884	1.38467	1.39355	1.2064
MLX	53.3925	49.6622	48.8639	51.1591
CNTNAP1	2.00625	2.24205	2.24696	2.00463
EZH1	6.5745	7.77161	7.7561	6.81805
DLX4	3.93677	4.8422	4.61011	4.83876
PPP1R9B	56.946	48.9367	53.8977	51.5737
COL1A1	5.32286	4.74655	6.10979	5.30376
PTGES3L-AARSD1	2.67397	1.31179	1.178	1.9179
MRPL27	170.654	144.972	150.156	153.738

VAT1	80.2003	77.2967	81.6466	76.9955
LRRC59	243.292	195.172	200.916	213.406
HDAC5	18.984	19.2486	19.4807	15.7759
ABCC3	7.87743	15.4996	15.7774	13.7858
LUC7L3	99.6903	80.1075	83.3228	83.2831
MPP2	6.83293	5.60928	6.58079	6.51418
SMURF2	33.5898	31.1467	28.801	31.0012
DUSP3	18.7449	17.5138	19.4894	19.2455
EFTUD2	150.21	146.12	144.831	148.43
SLC16A6	1.03154	1.36132	1.35323	1.5348
PRKAR1A	130.903	121.762	133.702	139.707
YWHAE	685.566	612.011	610.274	658.742
MMD	8.23552	8.32829	7.27119	7.29332
RANGRF	35.1379	30.0524	29.283	31.8627
DPH1	26.2503	26.2897	29.0554	26.4664
DHRS7B	16.3119	16.8004	15.1183	15.1782
WSB1	22.6056	24.926	25.5864	27.3661
SLC9A3R1	79.0148	72.7539	73.8837	77.4063
MYH3	0.99321	1.04028	0.742315	1.00274
NAT9	42.2889	42.4663	43.7439	42.0235
TMEM104	13.2272	12.2103	12.2811	12.0904
TNFAIP1	10.7494	9.9605	12.847	11.8422
IFT20	7.92688	7.93229	7.21136	7.50575
TMEM97	31.9496	18.8744	18.91	21.3723
CDR2L	23.8483	20.6682	21.8674	20.6278
PMP22	36.792	28.5414	28.1689	27.3781
UNC119	39.0219	35.0538	36.5872	34.9989
ALDOC	8.14952	8.01808	7.63107	8.22997
SUPT6H	29.7149	23.6049	22.4873	23.3314

PHF12	16.127	19.1891	19.6138	19.4976
TMEM33	67.2635	53.6527	56.2108	60.2053
SLAIN2	37.4694	24.0805	26.6012	28.6546
OCIAD1	122.559	122.294	114.958	129.415
DCUN1D4	19.0636	20.5922	17.0062	20.4688
USP46	12.4819	14.4976	12.7035	13.9985
CHIC2	6.15524	5.95439	5.8352	6.13473
NMU	116.012	86.891	86.2116	86.7197
KIAA1211	7.57546	5.01728	5.65216	6.60593
LAMTOR3	20.6281	14.6038	15.0772	17.0564
NFKB1	16.8303	14.1163	16.1497	16.0855
AREG	124.683	141.071	133.511	125.884
MANBA	5.54573	6.93334	7.34933	6.92281
UBE2D3	281.83	241.94	245.451	277.671
ELF2	9.05626	8.8339	8.20607	8.81987
NDUFC1	182.352	178.952	170.156	187.063
TBC1D9	3.01811	3.08149	3.24602	2.94808
ZNF330	45.1096	37.6067	40.7709	40.0208
INPP4B	3.08058	3.13755	3.49837	3.46935
KLHL2	18.5203	17.3602	17.387	18.7871
CPE	16.6897	20.1467	17.2998	17.1799
RPL34	1424.79	1515.97	1419.72	1561.11
WFS1	13.1604	15.0144	16.5158	14.5512
ANXA10	6.38428	5.87579	5.95443	6.46625
GRPEL1	92.5925	71.5586	71.444	76.5478
GAR1	53.4663	42.7945	42.5481	52.3376
FRG1	9.17453	8.24152	7.39148	8.74687
CLCN3	26.3272	28.1487	27.7147	27.9116
AADAT	9.44773	8.82001	8.87531	9.90539

GALNT7	10.3875	12.7121	13.1774	13.0719
DHX15	130.628	111.085	110.114	116.521
SEPSECS	3.52308	4.18832	3.91149	3.91113
TRIM2	2.87323	3.65761	3.69669	3.89093
SLC2A9	1.20158	1.52949	1.31396	1.2224
FBXW7	10.5018	8.5166	7.82051	7.66273
NEIL3	10.9495	9.42505	8.79651	10.1386
TBC1D19	5.65721	6.93867	8.98304	8.9854
WHSC1	57.1132	44.724	46.4129	47.6419
SH3D19	207.351	221.328	187.344	209.069
STIM2	27.3345	24.2096	24.7264	26.7383
MFSD10	82.4327	78.367	85.2317	75.1101
RAPGEF2	8.64274	9.67581	9.13205	8.4518
SNX25	7.38322	3.92438	5.17274	6.19008
UFSP2	26.3128	24.842	22.8095	26.4687
KLF3	7.78812	9.89665	9.54596	9.30507
KLHL5	7.84028	5.83868	8.59193	8.09667
FAM149A	1.66314	2.27446	2.31517	1.92341
NCAPG	43.4842	33.3223	33.4858	39.7356
UGDH	39.5711	30.674	32.7663	35.5473
HTATIP2	45.2484	46.1629	41.9834	46.1262
CTSC	44.6634	38.3248	38.9981	44.185
CCDC34	15.5793	11.8256	10.7266	13.2289
ELP4	13.9193	13.3941	11.9248	11.8056
ZNF259	49.3422	39.4165	45.6689	44.8265
MTCH2	236.439	211.481	223.593	227.288
FNBP4	20.8697	21.6693	19.9107	19.9017
SC5D	31.1402	19.8689	23.9843	19.8077
C11orf63	1.26674	1.232	1.14925	1.33387

HSPA8	2458.97	1776.6	1962.3	2137.97
VWA5A	1.21765	1.20619	0.95817	1.23115
DNAJC4	14.3074	16.5904	17.5975	16.2734
SIAE	11.0071	12.021	10.4648	10.3687
SNX15	7.95074	8.18624	7.03312	7.16313
DTX4	2.35991	2.17873	2.25115	2.53126
ATG2A	4.12306	5.16303	5.12122	4.35529
EHD1	48.8573	46.1397	57.1097	48.2818
OSBP	18.9307	17.4643	18.2412	20.5654
UNC93B1	38.3034	41.6287	43.4556	39.0978
PUS3	7.40809	6.03769	6.82283	7.49976
DCPS	27.2108	22.3567	21.723	20.9589
SUV420H1	15.3607	13.9675	12.3273	14.1976
FOXRED1	27.9992	24.9722	27.6889	26.8682
PPP6R3	44.0121	33.9604	36.795	38.7311
ST3GAL4	12.4764	10.4837	10.5334	10.0218
CPT1A	31.104	30.5745	28.2408	28.1368
CCND1	469.947	325.373	329.545	327.788
CCDC86	77.1241	56.0561	60.6826	62.2107
PRPF19	270.948	210.167	209.693	215.384
TMEM109	53.8108	45.6799	44.81	45.7215
HPX	1.29131	0.302825	0.544446	2.10989
TRIM3	9.11563	10.2257	10.7036	10.8541
CHORDC1	140.669	100.898	94.2459	113.276
FOLR1	1.61994	1.84198	1.87977	2.16456
ANAPC15	76.8055	71.6247	71.645	70.0211
PANX1	14.1714	13.6007	14.1005	14.5909
ARHGEF17	8.1691	7.83775	9.0966	8.08886
CEP164	6.83157	5.84213	6.25118	5.32345

RNF141	69.4511	40.063	47.8503	57.3291
EIF4G2	1001.11	996.561	824.068	949.846
GALNT18	14.1447	12.5497	14.5732	14.0396
BIRC2	19.2756	17.5665	19.0149	18.3891
UBE4A	8.32189	5.00105	5.02128	5.77414
DDX6	26.2565	24.4759	21.7124	24.6901
CBL	7.60708	5.70592	5.36705	6.15434
PVRL1	14.4318	14.7895	14.5712	12.9437
HIPK3	7.67375	9.36034	9.74416	9.81473
FBXO3	19.0742	16.2816	17.0538	18.7053
PDHX	18.3451	17.3413	16.7342	19.3995
COMMD9	36.9973	28.4331	29.1035	30.1144
ACCS	2.16703	4.42574	4.36105	3.28955
MDK	256.456	287.01	264.245	259.506
AMBRA1	7.1742	8.19697	8.82988	9.10552
MADD	13.0494	13.9563	18.6697	17.6538
PTPMT1	111.675	92.9288	96.1293	90.6533
NAA40	9.34205	10.8642	11.9197	11.0619
CARS	32.7145	50.8885	39.3494	37.6904
SLC22A18	9.39272	14.3605	15.6993	14.4599
CD81	308.752	281.691	295.325	274.496
SLC35F2	51.4294	31.943	42.061	43.507
ELMOD1	1.46616	1.6134	1.4266	1.41727
C11orf58	164.037	140.292	144.492	132.12
PITPNM1	29.1163	25.9211	33.6142	28.2831
RPS13	1612.25	1646.27	1462.71	1582.53
AIP	32.2483	29.5042	30.5594	28.8829
NUP98	73.9147	69.8852	68.7482	73.6034
NDUFS8	170.383	184.087	181.307	172.791

TCIRG1	45.6896	46.1669	47.8823	41.7508
CHKA	26.366	19.5192	18.7283	20.7375
HPS5	11.977	13.6523	11.6134	12.3049
GTF2H1	23.6524	23.1119	21.4429	23.6503
PSMD9	58.6487	54.0907	57.9603	57.8248
LEPREL2	9.88315	10.0794	10.0545	9.11824
PPFIBP1	30.4438	36.5004	41.552	38.2167
PRPF40B	5.53373	7.1928	7.76342	6.6249
PRDM4	19.4812	17.8015	17.2141	17.1162
COQ5	26.9559	26.1511	24.8236	25.6018
CORO1C	86.2282	57.6731	70.7456	70.9464
ASIC1	8.23119	10.0886	10.7871	10.0404
CAPRIN2	5.20421	6.11114	5.94702	6.10765
KCTD10	24.7515	22.8348	24.3888	21.4127
SLC11A2	17.3389	15.6185	19.12	19.0789
MLEC	70.5927	55.4708	55.1199	61.0185
MVK	15.3493	13.3987	12.8093	13.3729
CSRNP2	7.54121	8.18056	8.12864	8.14494
CAMKK2	16.0803	16.0896	16.1008	15.9261
ATP5B	1250.11	1194.92	1233.35	1276.36
PTGES3	375.52	313.689	290.66	343.792
BCL7A	6.89731	6.08482	7.28905	7.29931
RSRC2	32.9015	27.6496	26.8784	30.413
CYP27B1	5.13027	4.02983	5.1913	3.90429
KRT18	889.881	712.304	768.308	766.036
TENC1	7.54341	9.0725	10.5245	9.08138
GLI1	2.40705	0.847443	1.4687	1.74195
PPM1H	2.68094	1.83926	2.10695	2.67365
METAP2	151.364	118.318	129.936	129.921

LTA4H	64.7711	68.9429	66.9881	68.0661
ELK3	25.8135	24.9241	24.5258	25.758
MAGOHB	48.7916	40.7741	35.6091	42.7816
ITFG2	14.6077	13.7927	10.67	11.4001
FOXM1	68.7111	58.9589	54.018	57.4603
PRR4	2.34871	1.79173	2.00917	1.2622
ARPC3	148.91	125.298	113.2	134.494
GPN3	51.342	46.3166	47.1382	49.242
VPS29	80.8415	74.5212	71.0864	83.0982
MYL2	3.22416	3.21532	3.24951	3.1988
RAD51AP1	21.2654	17.5791	17.2249	18.1765
SH2B3	6.68474	7.10249	6.68071	6.57347
AKAP3	0.90317	1.49891	1.54745	1.35344
DUSP16	3.05678	3.1901	3.55861	3.52398
CREBL2	5.22658	6.07978	5.91175	5.7688
ACAD10	6.44469	7.02976	6.99024	7.04439
ALDH2	10.0014	15.4354	13.7641	12.9882
CDKN1B	12.3052	11.4587	10.4353	10.3611
GPRC5D	0.975391	1.07792	1.01471	0.850917
NAA25	19.8504	14.2267	15.4256	16.7107
SCNN1A	16.5241	16.3206	20.9471	17.9585
LTBR	87.7809	76.3609	78.9713	77.6832
OGFOD2	26.6653	24.0208	23.3224	23.1499
CDK2AP1	241.796	199.131	191.283	202.474
OAS3	11.6695	11.6817	12.6238	10.8374
RASAL1	6.46796	6.37783	7.43424	6.32786
ARHGDIB	2.3768	1.96792	1.80308	2.64982
GTF2H3	43.9072	39.079	39.8659	44.3729
EIF2B1	37.4382	32.0561	36.2686	37.5399

DDX55	28.6846	21.0943	21.8231	24.9996
SLC38A1	41.7731	39.7665	37.9684	40.9334
C12orf49	27.2144	21.671	23.9897	22.7883
VDR	5.96912	3.79547	4.88847	5.14723
RFC5	68.3021	47.7878	42.2923	52.0953
STX2	12.7438	11.4312	10.2868	12.3539
COPZ1	157.796	148.508	146.114	158.142
TBC1D30	13.4008	11.7003	12.9943	12.6656
CAND1	83.2897	72.9867	78.7311	87.2688
RAB5B	11.1911	13.1592	13.8033	13.4317
MDM1	5.07159	4.21064	3.4582	4.51908
NUP107	59.9856	51.5876	50.4055	58.1812
CNOT2	40.1531	36.0916	36.584	39.4242
TIMELESS	26.3823	26.4739	27.3568	28.5365
CPSF6	81.2077	61.5243	65.2174	66.686
KRR1	103.896	77.943	76.6884	97.0096
MRPL51	265.038	227.571	216.397	245.188
GAPDH	4188.37	4202.63	4299.05	4225.23
NOP2	87.7167	78.3524	80.6331	76.303
CHD4	241.972	217.025	233.086	213.92
UHRF1BP1L	8.12811	9.03898	7.44305	7.74056
COPS7A	81.5722	53.9956	74.5589	73.9792
ING4	4.06939	6.14125	5.80574	5.50007
GNB3	2.19812	1.56743	1.73304	1.60987
CDCA3	34.9184	28.9882	25.5517	25.8853
CHPT1	12.9663	11.63	11.9604	12.5849
USP5	43.7238	45.5462	42.9456	41.7542
TPI1	686.423	629.562	605.268	549.244
GNPTAB	14.7857	13.5357	14.3969	15.3314

SPSB2	4.99466	6.06265	6.51795	5.12808
ENO2	12.8827	11.0737	11.2227	10.2837
ATN1	31.3686	28.9064	28.4009	25.2233
C12orf57	131.795	111.743	106.418	99.0334
PTPN6	9.10125	5.77563	7.76965	6.95144
LPCAT3	21.6648	17.823	20.2928	20.6908
NT5DC3	6.32131	4.8333	4.83422	5.4252
SLCO1B3	3.56468	3.1046	3.03327	3.26575
SUDS3	14.5239	12.0822	13.7061	14.5601
GOLT1B	20.3353	19.3894	16.065	19.1186
LDHB	1178.28	1076.99	1085.58	1172.99
PRKAB1	9.98765	13.4387	12.6113	12.7396
CMAS	20.4569	21.5929	22.2941	23.9175
HCFC2	2.07901	1.70357	1.99331	2.39268
C2CD5	7.43305	7.37989	7.19699	6.85039
RAB35	65.8747	56.1241	58.5563	61.2412
PHC1	7.19219	6.53608	7.61226	7.09289
COX6A1	536.052	525.898	551.049	539.493
AL021546.6	3.79218	2.34322	2.81255	2.70197
RIC8B	5.09851	5.12431	4.53781	4.98593
SRSF9	253.873	223.956	219.052	248.989
RP11-22B23.1	5.4787	4.98231	5.16449	5.02961
FGFR1OP2	15.8887	13.6766	14.3829	15.7097
COL12A1	26.7773	25.2851	25.6046	21.3773
TDP2	25.6671	23.8773	21.7677	26.0757
DSE	11.3913	10.2266	10.3225	10.6517
RWDD1	27.5707	24.6552	24.5314	28.1271
TMEM14C	62.6628	67.1598	67.5186	67.7868
PAK1IP1	33.2962	25.6619	25.1098	29.6513

SMIM8	2.95427	3.01118	2.97357	3.56559
CEP85L	1.36778	0.76348	1.15483	0.970245
ASF1A	14.9967	15.6839	13.2179	14.9641
MCM9	5.05948	4.67734	5.08644	4.96276
RNGTT	10.7892	10.2338	10.3106	11.4792
MAN1A1	8.87837	13.0246	13.5881	12.6391
SERINC1	17.5611	18.0769	17.6115	18.3193
HDDC2	64.4452	54.1168	52.9322	58.7728
TPD52L1	43.575	44.3848	44.4438	46.7256
HINT3	6.7759	7.54522	6.88666	7.39557
NCOA7	2.96641	2.76769	3.05207	2.28815
SASH1	1.6592	1.56622	1.4025	1.5433
UST	2.48617	2.209	2.44593	2.14695
ULBP1	1.37835	2.72792	1.9977	1.86558
FBXO5	22.8455	16.7689	16.9333	19.9146
MTRF1L	23.8695	20.6101	20.1935	20.3729
PPARD	15.0029	16.8029	16.373	15.5707
FANCE	15.1932	13.059	14.016	14.2352
MAPK14	18.0944	16.2803	17.3835	18.4229
KCTD20	14.3559	10.8176	9.67422	11.0661
STK38	11.3944	11.2839	12.0215	13.1481
SRSF3	196.104	157.996	152.275	182.815
SOD2	72.984	61.9649	69.9155	65.8056
MRPL18	107.558	96.9878	97.0283	108.708
MCM3	137.107	118.411	125.801	130.526
RNF8	18.7556	18.4184	17.9334	18.9368
MDGA1	2.02979	1.52525	1.26525	2.05266
ICK	3.19014	3.00619	2.56817	2.83024
FBXO9	49.3554	45.0645	52.1864	52.6063

CD83	6.53774	6.15417	6.89029	6.85567
MDN1	20.0622	18.8189	18.5991	18.1008
SAYSD1	6.24469	6.00093	5.80206	6.24442
RBM24	7.72412	5.83467	6.83279	7.03358
CAP2	2.94754	3.20023	4.52642	4.76905
ZNF451	10.8705	8.78936	9.47558	9.84079
BAG2	42.3763	33.3298	33.8362	38.7468
RAB23	7.31858	5.82573	6.18289	6.52427
GPR63	2.20139	2.48348	2.80463	2.80349
FBXL4	4.47806	5.05841	5.44685	5.47401
CCNC	114.354	77.7829	68.3155	82.4149
E2F3	13.7988	9.26805	9.87555	10.8064
PTP4A1	219.353	239.793	226.505	275.113
ASCC3	21.5379	20.5141	21.8221	21.4212
MED23	7.06994	7.69792	7.83849	7.89483
WASF1	12.6791	14.6173	14.2047	13.0672
GPLD1	6.85461	8.36024	7.54793	7.34749
ALDH5A1	3.127	3.44839	3.031	3.05953
AIM1	1.87695	1.9387	1.84101	2.32407
ACOT13	15.0478	15.6677	16.5917	18.7523
RPS12	2482.97	2562.24	2465.22	2515.57
C6orf62	59.583	58.6328	59.9193	64.9007
GMNN	53.5799	43.4372	42.6084	46.9376
SOBP	1.08123	0.87456	1.10544	1.26834
SNX3	132.208	113.691	108.177	119.15
HBS1L	66.994	64.9084	58.5244	61.6849
TRIM38	2.77652	3.2183	2.99331	3.04934
PEX7	4.2716	4.92846	5.18387	5.29668
ZBTB24	5.48302	4.71902	4.73286	4.98367

FIG4	5.26231	6.62117	5.77999	7.27527
PERP	40.8238	45.3953	45.2045	46.6589
SLC16A10	4.14702	3.56267	3.09637	4.08344
HECA	1.74815	1.76309	1.66218	1.74337
GPR126	15.5844	13.7034	14.3245	15.1916
PHACTR2	5.91451	5.31052	6.15762	5.56609
EPM2A	1.36278	1.60903	1.46995	1.1142
SLC39A7	27.6895	25.6267	22.7788	20.7284
HSD17B8	1.13393	1.50853	1.52585	1.4469
TAPBP	4.85106	4.84897	5.99302	5.71224
PHF1	17.7137	13.9486	15.6374	14.3308
CUTA	98.8238	92.2517	86.3854	84.9135
QKI	12.7707	13.1935	13.1815	11.6862
MDFI	31.0486	26.0325	31.5462	28.8108
TFEB	3.02023	2.20069	3.20165	2.7366
CCND3	48.2034	33.065	34.7311	39.5598
BYSL	76.7392	52.8854	55.883	55.7746
FAM120B	8.07183	11.4045	9.22549	9.48487
TBP	19.5533	16.9722	17.5599	18.0789
GUCA1B	1.82554	1.5388	1.4834	1.46839
GLTSCR1L	3.1018	3.76744	3.17526	3.26443
PPP2R5D	46.2773	43.7291	43.4163	45.5305
MRPL2	132.186	131.297	133.999	131.041
PTK7	45.3721	48.7464	51.0557	45.8751
SRF	40.2167	33.8787	34.7171	33.8425
CUL9	10.039	14.9094	14.49	10.4616
DNPH1	117.158	133.008	130.533	116.144
DUSP22	9.51984	10.9902	11.2909	10.292
EXOC2	11.5392	12.6798	13.3322	13.3424

COX7A2	583.862	601.149	551.419	568.303
TMEM30A	40.7867	42.4511	35.9086	43.7655
GMDS	13.1332	13.813	15.8374	15.0157
SENP6	20.8591	21.7545	19.8747	21.565
VEGFA	25.727	26.1059	28.3615	23.7737
PRPF4B	27.7104	25.7639	26.7601	26.6091
TTK	20.3315	16.4046	15.9184	20.2757
SLC29A1	86.7553	67.2427	73.0572	73.1255
BTN2A1	6.58759	5.21052	6.12765	6.33872
FAM46A	5.33634	4.19519	4.5006	4.41071
FBRSL1	133.428	136.245	148.187	135.382
ENPP5	1.18841	2.45642	2.47212	2.41339
PRSS16	4.90542	5.02702	5.43284	5.17126
TBX18	4.40161	3.93448	4.12336	3.75495
ERBB2IP	19.0648	15.0971	16.2065	21.0774
HARS2	32.6832	31.2327	32.5829	32.7998
NUDT12	6.62378	7.69975	7.37958	8.53508
CEP72	9.29593	7.43431	7.86375	7.07019
MAN2A1	7.50531	8.41145	8.03642	8.92974
PAPD7	18.9743	15.2932	17.7296	18.4251
HMGCS1	22.9599	18.7824	19.1282	22.4952
DAP	39.0731	41.8366	42.1257	41.9431
NME5	2.75001	2.79105	2.12494	2.20918
BRD8	22.9559	24.4881	26.1521	22.7889
KIF20A	38.2178	34.1252	30.1854	33.3849
MRPS30	69.3924	65.1012	64.3608	67.1954
HSPA9	405.353	388.895	376.383	407.238
MRPS27	100.802	94.3372	95.3708	93.075
PFDN1	63.4346	63.5295	64.3635	62.7944

HBEGF	5.00056	3.05292	3.62315	3.91902
APBB3	7.09197	8.41258	9.47148	7.24325
TMCO6	14.8929	13.9269	15.0721	12.7182
IK	64.9276	58.8859	62.0323	63.4804
HMGCR	37.8481	32.7692	34.2241	37.528
COL4A3BP	9.39162	6.71044	8.23261	8.19542
FAF2	33.2186	28.7367	26.7961	30.5483
CLK4	3.47123	2.98403	3.37825	3.4739
RNF130	28.5985	29.977	27.7137	31.5068
THG1L	41.778	37.794	39.2338	40.952
ARSB	3.3208	3.36761	3.42622	3.72067
CLINT1	60.1533	50.7389	57.2414	52.3637
CNOT6	20.9398	23.0556	20.4607	21.8208
TTC1	51.1421	46.7856	42.4653	49.0226
MSH3	1.00725	1.04556	0.881816	0.959176
CCNG1	59.3677	87.6087	72.067	72.0921
POLR3G	37.0097	28.3046	30.0471	35.3598
DROSHA	42.9666	38.6124	34.5473	34.6069
LMNB1	139.852	132.455	128.171	121.672
ARRDC3	7.99629	8.77716	7.75311	9.43279
GOLPH3	36.8092	31.9873	32.4419	35.4635
SUB1	211.175	173.674	161.998	181.245
FAM172A	8.20362	6.81662	8.98311	7.72598
SLC27A6	3.94829	3.69033	3.87292	4.12573
TARS	104.474	111.761	97.9899	99.12
IRX4	10.5646	11.2982	11.441	10.2272
LNPEP	1.67453	1.57048	1.73618	1.82548
RAD1	26.224	23.3728	22.6745	24.0919
BRIX1	86.4223	71.6961	65.9008	77.9047

SLC12A7	16.4622	14.9922	16.7368	16.1434
RAD50	19.4055	16.1816	18.0836	19.2797
GNPDA1	60.8196	53.3421	53.2118	52.4876
SKP1	314.651	291.74	258.422	290.246
NUP155	34.4805	28.4144	26.347	29.654
PPP2CA	254.804	202.678	217.599	214.156
NR3C1	2.55339	2.57498	2.74528	3.00677
C5orf15	25.1696	21.1577	24.9744	26.0435
PPWD1	14.1532	12.4764	11.9418	14.5177
LIFR	2.35974	2.4693	2.75094	2.48618
TRIM23	5.28613	4.91959	5.3456	5.57648
TRAPPC13	9.39296	10.9537	10.9552	11.0669
SEC24A	8.70435	7.27484	8.746	8.98992
TXNDC15	19.5299	16.6104	19.0486	18.529
TTC33	6.55907	4.80502	4.87895	5.3841
RARS	91.3271	81.2119	83.3406	90.9482
WWC1	50.0628	43.9189	49.3188	53.4254
H2AFY	171.868	73.9369	123.975	134.249
TCERG1	46.1847	35.6717	42.2821	41.9407
DPYSL3	12.8201	11.1075	11.2213	10.9605
SMAD5	22.3384	17.6748	18.2458	19.2982
CSNK1A1	151.359	142.931	128.303	142.448
HMGXB3	30.3433	24.6772	28.7766	25.837
ERGIC1	44.5835	42.9384	44.5248	42.8948
ATP6V0E1	142.708	143.943	140.631	151.13
BNIP1	16.892	14.4326	15.2973	16.5538
STC2	27.1768	36.1782	27.0764	27.9958
CPEB4	3.73053	4.35017	4.011	4.42119
DBN1	139.393	95.678	116.031	98.859

ZNF346	8.39098	7.92597	7.62189	7.65429
UNC5A	2.90188	4.97385	5.61092	4.23045
EHHADH	2.58155	3.29248	2.69104	3.05355
SMC4	55.6311	46.889	46.7854	51.6431
SELK	47.7642	40.6441	42.7188	43.9034
ACTR8	16.2055	12.7656	14.4662	17.2938
TBCCD1	12.3554	11.8123	12.6917	13.1714
TIMMDC1	76.0614	80.4822	76.8096	82.3779
CRBN	15.8456	16.1043	16.6705	14.5751
BCL6	2.86221	3.49915	2.93498	2.99629
ARL6	2.54129	2.29418	1.95744	2.28819
NPHP3	2.19268	2.29313	2.33342	1.84727
AMOTL2	32.8192	19.0836	22.9484	20.384
NIT2	33.3137	37.6556	35.4174	38.5837
FAM162A	62.1855	56.7659	56.3069	66.8609
OGG1	13.5315	13.1655	14.7476	12.6152
KPNA1	35.3468	30.17	31.3011	34.6125
PCCB	38.0975	40.7892	46.0872	43.5062
UBE3A	22.5835	19.3606	19.1268	21.686
ARMC8	42.5243	41.9985	52.9341	43.4819
CEP70	13.5708	11.5146	12.1952	13.3839
RBP1	104.002	105.607	96.1464	97.5655
SLC25A36	21.6788	20.3906	19.9605	20.852
RNF7	79.5348	78.9765	75.6972	76.1884
TFDP2	16.6051	16.0699	14.6335	17.0001
XRN1	4.15618	4.605	4.91364	4.41838
KAT2B	1.01166	0.624915	0.930457	0.768044
PDCD10	41.1168	40.0626	34.9136	41.9601
PFKFB4	5.79817	5.00419	5.84303	5.22811

COL7A1	2.28846	2.7324	3.75769	3.0053
PRKAR2A	22.7335	18.8303	20.4347	22.0556
HES1	10.1992	10.1166	9.00129	9.18146
USP4	14.1758	13.3529	12.2803	13.0655
ACAP2	8.99931	7.10043	7.31167	7.88764
ECT2	43.9767	37.3003	35.7466	40.8073
GNAI2	63.4127	37.5845	51.2965	50.4025
TFG	81.2261	75.7707	77.4431	77.3155
HYAL1	0.5997	0.842767	1.11171	0.943754
TUSC2	30.5735	27.8461	27.4396	24.1492
NPRL2	27.4852	22.2263	25.7217	25.6044
RPL24	1142.49	1086.93	949.174	1170.63
CYB561D2	18.38	17.6102	20.3995	18.4894
FXR1	123.763	114.426	112.226	115.52
CBLB	1.23834	1.50083	1.42187	1.62591
BBX	9.27313	10.544	8.87764	10.356
IFT57	31.2633	27.0065	29.1031	32.769
IQCG	4.06054	4.48152	4.24146	4.88143
GBE1	13.7185	14.1537	14.4555	15.3086
UMPS	79.6322	60.4796	63.1036	69.6527
NCBP2	55.3124	54.2623	50.1299	59.3209
SNX4	16.6791	12.3146	10.4876	11.1146
C3orf52	11.4858	9.43704	9.17995	9.48938
SLC41A3	21.079	22.1673	23.0716	21.4078
PLXNA1	22.0659	21.5363	23.7774	20.6814
ATP6V1A	20.0351	10.3584	14.2102	16.2182
ABTB1	4.09609	5.41011	5.46845	4.1169
PODXL2	1.08821	1.8335	1.6077	1.50452
CSPG5	1.65595	1.49654	1.99647	2.17843

KLHL18	25.6628	19.5683	23.9303	22.9685
SCAP	32.6001	34.6982	37.9371	33.4686
KIAA1257	1.11029	1.26951	1.3264	1.26084
MRPL3	188.916	153.762	150.702	168.873
PLSCR4	0.567375	0.578287	1.03752	0.92994
HEMK1	9.32713	8.82567	10.2828	9.22409
CISH	2.91648	2.63999	3.21106	3.04801
MAPKAPK3	90.2387	71.2627	82.4856	83.3814
ACVR2B	1.92305	2.01254	1.86044	1.80449
WDR48	9.95362	10.3844	8.76829	10.5825
COMMD2	23.568	20.7316	19.3519	20.3809
GORASP1	20.016	19.4003	21.9597	20.3155
RRP9	94.566	77.386	79.0533	78.1522
ABCC5	6.06758	5.27695	5.25448	5.57521
ABHD14B	36.6166	51.6523	46.8491	43.3522
EIF1B	33.1117	25.8876	26.3655	29.2213
ABHD14A-ACY1	0.653298	0.993562	1.1156	0.649438
ARHGEF26	4.99907	2.97811	3.3995	3.59532
KLHL24	1.21089	2.27254	1.54149	1.05792
VIPR1	3.84223	4.43234	4.81463	4.51036
DNAH1	0.653117	1.02484	0.927035	0.820386
SSR3	129.099	108.231	100.295	110.656
ZBTB47	2.64044	2.05474	2.45318	2.40154
TNNC1	4.48247	3.79997	4.5321	4.34187
NKTR	7.4789	8.03543	8.10691	7.55454
CLCN2	13.2016	10.9298	12.1887	10.1637
FOXP1	0.774584	1.16401	0.844104	0.872988
EIF4G1	340.052	251.033	296.862	270.779
SPCS1	146.06	127.373	124.832	136.564

NEK4	19.2024	14.5485	16.942	16.8501
SLC4A3	7.52633	7.86192	8.65874	7.82599
INO80D	1.15077	1.1607	1.02783	1.0895
EEF1B2	917.641	871.793	823.235	890.501
DGUOK	97.2455	87.8287	81.6615	89.8218
MOB1A	50.6201	47.8937	46.7704	51.8732
KANSL3	16.8484	15.4042	17.4067	15.3367
LMAN2L	16.1434	13.8981	13.7523	14.8361
RTKN	45.8632	44.4627	48.8149	45.6206
TTL	21.896	16.8341	16.1943	18.4335
PIKFYVE	3.26719	3.37565	3.5694	3.68054
KCNIP3	1.01985	1.29038	1.34178	0.941453
FAHD2A	15.103	15.9539	14.6327	14.3605
NCL	1045.53	800.475	881.358	935.772
ACTR1B	15.2893	16.5884	17.7356	16.5242
SLC35F5	4.03818	5.62744	3.79057	4.90066
ACTR3	150.362	132.319	133.414	139.254
STEAP3	20.3246	21.7644	22.0262	21.1077
EPB41L5	8.12406	7.89173	8.44098	8.42664
TFCP2L1	2.49614	2.24924	2.37544	2.5081
SF3B14	141.247	133.654	134.729	147.193
TP53I3	7.32774	20.0549	16.0028	13.8411
DNAJC27	0.889506	1.01376	0.933312	0.933095
STAM2	7.60952	6.88732	7.2304	8.13473
GPD2	29.3465	23.6262	23.624	27.4753
CENPA	30.7521	23.4896	24.7082	24.0502
ACVR1	7.34898	7.80447	9.08477	9.01829
TANC1	3.7474	4.20144	4.18643	3.99997
SLC30A3	12.2584	10.9863	11.3675	11.3287

MPV17	43.4751	40.9318	45.5684	45.4318
GTF3C2	53.2077	45.0703	45.6878	46.3893
EIF2B4	76.8605	60.1744	73.1655	67.8623
NRBP1	63.8464	75.0862	74.1175	71.054
FNDC4	1.35178	2.00637	1.69587	1.65471
PSMD14	121.383	108.506	105.938	119.864
SNX17	68.8658	65.2539	71.6554	67.9023
GPR75-ASB3	9.05768	7.82469	7.79855	8.39033
PPM1G	140.1	114.645	117.715	118.394
REEP6	29.7806	32.5864	33.9222	30.8687
PCSK4	1.10627	1.22894	1.46006	1.5158
IFIH1	0.784221	1.11901	1.0965	1.10475
RPS15	1627.32	1421.55	1436.2	1361.48
GCA	10.774	10.4448	8.94675	9.24617
INO80B	13.6571	16.3215	15.5164	15.1647
MOGS	40.7234	37.8704	37.9194	37.9601
TTC31	10.4468	10.9892	10.8215	10.9759
NDUFS7	91.288	84.0345	89.6154	80.2591
PCGF1	56.0312	42.8832	46.2498	43.8804
GRB14	2.85087	2.71853	2.46125	2.4113
CLIP4	11.8555	12.7207	11.8515	11.5042
SPTBN1	19.7923	19.7195	19.4637	19.8929
AUP1	141.505	124.845	135.427	130.427
RTN4	193.714	176.199	170.241	195.947
HTRA2	30.2753	24.9679	20.2486	20.0386
LOXL3	4.88783	4.72038	3.67935	3.64996
DOK1	16.4216	14.546	16.4948	15.8063
GALNT3	2.61877	3.20095	3.49661	3.69306
POLE4	39.707	37.3883	34.9185	38.4103

CCDC88A	4.41694	4.03633	3.65649	3.70599
EVA1A	1.73006	2.06496	2.35607	1.87193
MRPL19	65.9539	55.0616	53.9418	61.4278
LANCL1	26.014	16.3009	20.837	21.8016
WDR75	56.5581	45.4693	45.8181	51.4273
FANCL	22.734	21.0586	19.9499	22.0647
FN1	4.68493	4.9775	3.73912	3.30224
STAT1	28.3055	27.924	30.4788	30.5388
GLS	41.1899	38.5923	36.5331	38.877
PAPOLG	5.3096	6.50655	5.52015	6.35513
PECR	8.8735	9.96361	9.47645	9.07181
UNC50	29.5364	26.4032	27.0319	26.9008
IGFBP2	130.735	133.354	138.001	130.028
ELMOD3	8.34756	7.59701	6.50937	6.14994
USP34	30.7972	25.6346	25.4984	27.2254
CCT4	318.055	277.986	259.276	300.305
GGCX	23.4407	22.508	24.4772	25.5336
EHBP1	18.0236	17.2009	19.6671	18.1085
OTX1	3.1399	2.86475	3.20735	2.88043
TXNDC9	14.7211	12.0748	11.4943	13.7743
COQ10B	15.4731	14.1448	14.167	14.9495
SF3B1	188.676	171.977	164.141	184.598
ST3GAL5	4.71658	4.9452	6.69702	6.08317
PDCL3	25.7358	25.4614	24.6204	26.5256
MOB4	26.27	19.3661	23.3654	24.3446
HSPE1	938.905	707.468	667.605	746.923
KDM3A	8.92994	8.27343	8.98271	8.08263
PLCD4	2.28475	1.64472	1.85489	1.8015
CHMP3	39.0411	39.2279	38.9761	40.49

ZNF142	13.81	11.2295	12.8659	12.2589
IL1R1	2.86186	2.60654	2.71622	2.93727
WNT6	0.861035	1.04268	1.06241	0.852243
FHL2	61.0622	66.1683	65.6183	64.3746
MLPH	36.1821	32.4394	39.4203	38.0755
CNPPD1	24.8357	24.9398	32.7107	31.1461
UXS1	37.4586	38.8874	30.6301	28.1327
ABCB6	19.0047	22.4188	19.0755	18.1271
STK16	30.3147	26.6344	27.1516	26.6332
HDLBP	242.649	238.729	236.507	238.148
PPP1R7	56.9191	48.2895	53.8341	54.5417
PASK	11.4669	9.1289	10.2044	10.0368
STK25	138.999	136.15	144.265	133.989
ID2	142.545	110.338	113.831	130.063
TAF1B	13.3529	11.4363	11.5666	12.5551
HPCAL1	63.7193	50.6659	62.0775	58.1126
ODC1	403.609	264.522	316.935	350.219
BIRC6	17.9171	19.302	18.3521	18.1301
NOL10	26.2345	21.3854	22.2941	22.8566
PLEKHB2	69.4036	51.3869	56.7277	60.2938
GORASP2	77.9092	66.1024	70.9809	71.6431
STRN	6.58407	5.20791	5.00594	5.27457
CEBPZ	20.4356	16.4468	16.68	19.0196
PRKD3	5.54539	3.98456	4.37362	4.99543
DCAF17	5.68624	6.49067	6.05277	6.80035
RAB3GAP1	16.9955	17.3134	15.8774	16.0855
SLC25A12	11.3069	9.39254	11.615	13.0481
RMDN2	1.02025	1.20564	1.41472	1.3253
DLX2	2.31071	1.80284	2.1765	2.25798

DARS	83.6242	72.6599	66.1422	66.8381
SRSF7	217.566	148.265	163.582	186.161
SDC1	36.2761	36.2765	39.9832	36.8811
SLC1A4	1.53178	2.96438	1.55558	1.49978
SOS1	6.60568	6.17858	5.33685	6.00654
ORC2	18.6822	18.86	17.1871	18.5075
COX7A2L	74.0158	62.7067	63.4475	68.5841
PNO1	51.0323	42.0353	41.5777	42.9301
ORC4	24.3697	21.5517	22.0474	24.6735
RND3	13.9531	11.549	11.3356	11.6123
ATF2	7.34385	8.20272	6.42411	7.11837
THADA	12.4128	13.5718	15.5772	14.7341
AAK1	2.78675	2.42174	3.23274	3.21487
TRAK2	6.31931	6.85217	6.91909	7.25814
C2orf42	6.06233	7.03934	6.86854	5.73453
TIA1	42.2423	40.6408	39.7614	42.8124
PCYOX1	18.0102	13.3064	13.341	13.8866
KISS1R	4.64538	4.70271	4.86154	4.4548
EPAS1	10.6087	8.93818	10.5661	11.2868
ARID3A	13.0982	14.1618	16.1192	13.2051
SUMO1	98.8159	105.308	88.3539	103.407
NFE2L2	70.2668	67.279	63.512	68.5244
MSH6	26.3197	20.8874	21.8289	24.9609
PLEKHA3	7.74843	8.38569	8.72855	9.08621
SPR	73.5034	68.1469	72.8498	71.2481
FARSB	77.9482	68.0774	69.8339	79.74
ALMS1	9.41347	6.88891	6.68414	7.95258
BCL9	4.58252	5.02042	4.68959	5.04828
DHCR24	81.4866	56.7087	64.9256	69.6796

DNAJC16	9.82606	5.6035	7.51962	8.57371
MARK1	2.51391	1.8693	2.19492	2.70662
MORN1	2.25995	3.4078	3.67214	3.68739
CACYBP	392.538	304.353	285.585	389.012
SCP2	38.6153	40.7393	38.0717	43.7047
RALGPS2	5.93312	6.61424	6.32511	9.01274
CEP104	23.3812	25.0767	26.0589	24.9654
FAM20B	11.8025	10.6005	11.0064	11.6103
TCEANC2	2.37851	2.25961	2.28834	2.64305
TMEM59	42.5818	47.2235	42.2479	47.2642
LRRC42	54.7748	50.3114	53.5567	55.5864
WRAP73	59.0063	54.0707	54.8751	50.2112
MRPL37	118.912	103.456	103.31	107.66
ICMT	66.0932	45.6477	42.7769	47.7577
RPL22	723.982	677.287	611.448	742.539
CHD5	2.91154	2.10738	2.09148	1.93853
QSOX1	39.4132	36.4193	37.3626	37.7417
STXBP3	12.1786	10.6558	12.3065	12.9655
PHF13	13.42	10.4402	11.3142	12.8798
ERRFI1	142.144	150.881	150.606	157.516
PARK7	442.089	406.354	414.131	438.95
AMPD2	47.9884	43.2813	47.0611	44.2089
SRSF4	48.6545	57.8504	54.0335	50.4105
MECR	28.6358	33.8874	30.3552	29.107
KCNC4	2.50352	2.84081	4.08996	3.14108
EDEM3	3.65862	3.34055	3.8321	3.59566
WDR77	134.478	124.297	136.298	121.251
ATP5F1	440.108	390.862	408.097	426.277
RAP1A	31.0199	28.5706	27.5429	30.497

HDAC1	177.521	181.28	193.665	185.569
CAPZA1	114.017	109.067	106.58	125.098
S100PBP	12.376	12.6086	13.4266	14.6318
RNF19B	14.3336	14.0659	13.0704	12.8774
SCAMP3	44.3473	33.9264	34.7487	34.142
TRIM62	3.36964	2.61248	3.50649	2.85156
ASH1L	5.66565	5.98758	7.07651	5.96175
SFPQ	225.352	196.36	202.207	206.63
RHOU	1.81558	2.26935	1.44966	1.98611
GON4L	15.3394	13.6228	14.4489	14.2122
ARHGEF2	16.7878	22.2431	23.216	19.985
LAMTOR2	62.6913	66.8511	63.7372	62.2601
MEF2D	17.3116	16.6805	20.3096	18.6152
DOCK7	8.81288	7.63328	8.04612	8.56606
SRM	198.792	195.69	212.973	187.456
FBXO2	51.0735	62.5673	56.6263	51.0693
FBXO6	26.1559	22.2873	25.6615	24.7833
C1orf21	5.46896	4.25532	3.5465	4.91953
MAD2L2	91.3195	83.7872	83.894	85.4271
DNAJC6	3.37301	3.3507	3.26506	3.31666
LEPR	1.7222	1.50018	1.51076	1.98666
IVNS1ABP	31.4714	22.7523	25.4312	26.7432
KIAA2013	60.5217	51.2627	56.7029	53.8259
MFN2	66.4203	57.0464	55.8424	58.1239
MIIP	30.9393	29.2078	29.0482	27.4242
SMG7	24.5597	21.1926	22.6616	24.6017
SLC35D1	3.6621	3.66201	3.90948	4.23393
PLA2G4A	4.44933	3.4578	3.87172	4.3941
GADD45A	30.3244	42.031	36.5323	31.645

WLS	22.8956	24.0623	23.0748	25.2388
PRDM2	3.0469	2.8746	2.79592	2.97734
RGS2	14.3827	10.1684	14.1781	15.5081
TROVE2	21.3525	17.1058	18.6066	21.1588
UCHL5	47.064	45.1738	40.6437	47.8284
BCAS2	43.0552	39.81	36.9077	42.3534
SRSF11	91.6333	81.084	79.3231	82.8529
CTH	3.6592	6.00606	5.23668	5.10068
PLEKHM2	28.3688	27.6024	29.5254	26.5637
CRYZ	28.249	24.8524	26.8333	30.3801
PHTF1	7.18331	6.03973	6.07116	6.21722
ZBTB17	20.7495	20.3523	23.1823	17.8904
CD58	10.7402	11.9017	12.6002	12.3195
TFAP2E	5.3174	4.46788	4.8066	4.82131
TTF2	19.978	19.5265	20.1843	23.3009
KIF21B	1.11622	1.24542	1.15704	1.04082
TMEM9	63.0178	56.0062	60.4276	54.0095
ADPRHL2	32.6021	27.3939	29.5002	28.567
MAP7D1	48.807	42.6828	45.8124	44.2163
WARS2	3.68673	3.84007	3.55905	3.52278
OSCP1	3.63299	2.34715	2.76738	2.34177
MRPS15	114.433	104.971	97.4827	110.123
EXOC8	3.92631	3.5238	3.64742	4.1191
GNPAT	28.2679	30.8499	29.9348	31.0581
TSNAX	27.3789	23.3061	24.2581	25.7209
C1orf109	36.6643	31.8122	34.0729	36.2649
RRAGC	8.84447	5.27821	6.8465	8.57855
TBCE	27.421	26.1052	23.0599	27.7451
LGALS8	13.1053	14.5725	14.021	15.5294

HPCAL4	1.93497	2.14861	2.29855	2.28451
MTR	4.72841	3.46293	4.48027	4.91533
BMP8B	3.79257	3.81045	4.03385	3.9429
MYCL	2.3699	2.70971	2.56246	2.5658
SIPA1L2	2.89461	3.52616	3.49576	3.46906
RLF	5.42835	4.81566	4.61028	5.14053
ZNF684	2.47546	2.51749	2.14811	2.48742
KCNQ4	2.12723	2.1907	2.60672	2.26673
RIMS3	1.65681	1.85869	2.06029	1.73868
ETV3	7.78758	7.10882	7.54861	8.01953
ACADM	30.2311	28.0608	23.0387	28.7756
SDHB	182.873	185.866	189.619	183.809
MFAP2	75.9198	81.9609	80.8691	73.9469
RPF1	82.7904	70.1399	68.1763	76.9947
KDM5B	5.6461	6.88409	7.58899	5.62329
UAP1	41.9127	34.7959	35.7967	35.6392
CTBS	6.21589	7.4293	7.26078	6.98109
KLHL12	18.9691	18.9605	18.7109	19.3903
SSX2IP	21.0983	18.1556	18.2241	20.5893
ZNHIT6	12.7916	9.91101	9.37116	11.0664
RBBP5	13.4681	11.2422	10.8396	11.7689
PINK1-AS	6.44389	5.83842	7.39851	7.21082
GPR89A	5.52329	4.74421	4.19053	4.79721
CDK18	5.2031	8.0427	10.0826	8.98397
RAB7L1	11.0159	7.76639	10.8732	9.70109
ECE1	32.3621	39.3853	37.6043	35.4039
HMGCL	12.9296	16.2734	16.2365	14.0295
GALE	79.8022	95.7325	109.695	99.168
ID3	93.8708	91.9738	92.5941	95.31

CR2	1.21126	1.25151	1.1284	1.11248
CD46	44.2818	45.2236	41.0749	44.3284
PRPF3	15.0291	13.1322	12.6798	11.991
APH1A	61.4879	51.3659	51.2992	52.1492
LEPRE1	20.0098	24.7998	24.6026	19.8225
SLC2A1	200.319	212.056	212.637	196.389
EBNA1BP2	156.67	124.691	132.424	143.583
CDC20	461.732	354.473	370.012	378.939
ARTN	23.815	23.6037	27.5282	27.6891
IPO13	14.056	15.831	15.8385	14.9751
ATP6V0B	196.371	166.518	170.127	155.951
B4GALT2	83.3913	86.1913	93.0531	89.0892
ERI3	84.1279	80.7757	83.2732	74.8061
AKR1A1	122.182	131.962	125.548	119.23
PRDX1	1595.39	1508.47	1371.51	1525.15
TSPAN1	2.82404	3.78537	4.12233	3.56397
BLZF1	10.0381	9.94292	10.1334	11.5547
SLC19A2	9.73702	7.16423	7.73117	8.47729
FAAH	10.457	11.0123	11.5114	11.5433
NSUN4	21.9131	17.9275	18.5806	20.3694
TMED5	19.3626	15.5159	15.7305	17.6206
DR1	47.1284	33.325	33.8053	40.0055
CNN3	128.615	99.2708	91.5199	93.1924
PRRC2C	52.0463	45.7872	43.2702	47.4089
F3	89.6198	48.6314	62.3982	69.2893
ABCD3	30.852	25.7873	26.0746	30.5865
VAMP4	2.18063	2.54147	2.72741	3.3843
DPH5	33.7772	30.0343	30.5705	31.7798
PTBP2	5.96772	5.72006	10.2274	10.6998

PRDX6	247.807	234.545	223.979	240.532
DARS2	22.3103	19.8533	20.3906	22.8146
IRF6	8.34371	7.82815	7.93331	7.57423
DIEXF	6.00094	5.38211	5.37773	5.84815
RCAN3	3.84468	4.16508	4.77511	4.49999
SYF2	25.9958	23.5933	23.9752	24.9592
C1orf63	19.654	17.5301	14.4109	15.6106
SLC35A3	7.36245	7.04067	6.67673	8.54282
RCOR3	10.7551	11.9357	11.832	11.3866
STMN1	245.128	189.771	193.254	217.082
MTFR1L	22.8678	24.3317	25.1601	25.1087
NEK2	29.7774	22.9876	20.3641	21.6372
RPS6KA1	34.2619	33.7672	35.8398	32.9257
DHDDS	25.0765	21.9585	24.5562	26.5816
NENF	71.8113	74.0678	68.5417	69.6792
NSL1	24.38	17.9948	15.4805	19.2718
ARID1A	20.2965	19.5984	20.6504	20.9323
CENPF	28.6462	29.8911	26.0173	26.4579
RPA2	71.2465	70.3358	65.5197	71.2794
PPP1R8	69.1278	57.3622	58.5904	57.8315
STX12	10.6847	9.16743	9.26356	9.50596
2-Mar	7.22274	6.07952	5.4141	6.15531
OSBPL9	23.5411	28.4021	27.6211	26.2875
TXNDC12	76.1108	76.4872	67.301	76.6885
ESYT2	32.4294	31.5633	31.8855	33.3497
CD3EAP	12.9768	8.67114	9.10275	10.9934
MESDC2	18.9304	17.4945	17.1455	18.8012
RCN2	77.3457	76.3208	77.2436	81.2345
CHRNB4	0.759621	1.01218	0.922861	1.16265

CTSD	71.5157	88.8243	91.1565	82.5627
STAG1	12.5173	10.3246	10.4426	11.5095
STK11	78.4624	79.1722	78.0504	70.5408
KMT2A	4.998	5.23044	4.68501	4.80178
TREH	0.857422	1.18874	0.872236	0.748891
IFT46	5.85791	5.45543	5.02965	4.88517
KPTN	10.7728	14.0385	10.7932	11.5347
RPS25	459.87	482.465	413.235	437.274
KIF14	6.20625	5.60502	5.45571	6.06065
DDX59	6.83344	6.03954	6.24561	6.08646
CAMSAP2	8.12227	7.08838	6.93798	8.14737
ATF6	7.90708	8.36966	7.79986	7.67854
MREG	9.66942	9.60344	8.87544	9.50332
FASTKD2	27.6912	24.0904	25.8805	28.1223
CREB1	23.6415	22.0873	21.0238	22.1881
KLF7	1.86433	1.64794	1.71504	1.4013
B4GALT6	3.80861	4.41234	6.09764	6.73654
SPCS2	128.245	103.64	74.8214	99.3045
USP35	1.70299	1.87898	2.01037	1.85795
CASP8AP2	3.58891	3.25698	3.30334	3.90782
HMGN3	46.8817	33.8461	38.5495	45.9783
UBE3D	6.70605	3.87618	4.98494	4.68205
ANKRD13C	8.96273	9.1609	9.62541	9.86316
PHF3	8.57377	8.19617	7.87334	8.59748
PLAGL1	13.1678	11.3686	12.7312	13.6576
FBXO30	5.20628	3.60753	3.40362	3.85087
TNFAIP3	1.12975	0.864202	1.10187	1.28819
AKAP7	1.17374	1.22544	1.02344	0.994012
MYB	1.32014	1.15627	1.4762	1.69195

SGK1	1.74094	1.49693	1.76896	1.61633
RNF146	7.39349	7.96148	7.21379	7.85898
CTGF	3.40167	2.41303	1.99005	2.34206
PMFBP1	13.5173	12.6542	13.4367	12.9753
FBXL5	12.7641	11.8252	12.6705	13.0351
MED28	38.8381	46.8525	41.2371	41.7646
SLC16A7	1.34514	1.04227	1.19552	1.70592
TMEM5	16.8435	14.8059	13.854	15.8956
ZNF430	4.17926	3.39443	2.94781	3.50834
VAMP8	44.6917	45.6361	49.3531	53.1301
DCLRE1B	7.6234	6.28986	6.52411	7.03473
MYL12B	419.969	405.243	399.119	426.933
FOXO3	9.17031	9.64614	8.22047	8.55562
ARMC2	2.24001	1.97375	1.20747	1.7231
RPN2	165.255	157.584	175.429	161.739
TGIF2	7.90077	6.73653	6.70677	5.90413
PKD2	3.11424	3.25322	3.53217	3.46496
ABCG2	1.02384	1.06648	0.930461	1.09268
SPP1	4.62235	1.7643	1.11863	1.10929
FAM47E-STBD1	3.29806	2.89939	3.04703	2.86865
CCNI	90.7719	96.9881	97.5473	96.8004
MFSD1	15.9975	12.011	11.7793	14.1145
RAB3GAP2	9.92472	7.68075	7.46479	7.09272
FAM86A	30.7491	30.8147	33.4722	30.1278
PPL	4.99631	6.40697	7.49277	6.86788
UBN1	24.8707	22.0835	24.1265	23.3845
KLF12	0.704373	1.00822	1.15549	1.08152
UCHL3	29.7754	26.3597	29.4465	31.5598
HS1BP3	9.88076	10.5377	10.5377	10.9354

C2orf43	14.3357	13.4006	12.619	13.32
WDR35	3.36201	3.469	3.05665	3.40485
ELL2	7.82238	8.14066	6.7689	7.84864
CYP20A1	2.71515	3.20422	2.59001	2.99615
NDUFB3	125.343	117.845	104.476	126.092
GTF3C3	33.9167	30.0186	30.6989	30.5472
SATB2	2.72618	2.78811	3.03041	2.94689
UBE2B	75.07	65.6586	63.2118	70.1225
GDA	29.0158	22.858	22.8092	27.5133
TJP2	9.47374	8.29717	10.2409	9.24863
ITGB1BP1	73.9904	65.1188	59.7203	68.9569
CPSF3	48.0753	45.9295	45.1268	48.4123
SENP5	16.3105	13.6684	12.7531	13.732
CCDC92	1.39107	2.91134	2.97498	2.39819
C1orf198	15.2655	17.8373	16.7167	15.6174
HEATR1	18.2214	18.6369	18.2437	20.1181
PTBP3	22.2454	22.6589	21.9641	25.2584
RAD23B	276.094	177.201	184.954	180.027
FKBP15	7.66309	6.52453	8.0179	6.67343
CTNNAL1	60.3733	54.5904	54.6693	64.187
FAM206A	12.1109	11.1915	8.6484	9.56399
WDR34	118.03	106.07	115.551	109.106
SET	444.739	334.618	310.867	366.895
PPP2R4	115.701	105.201	101.452	103.413
GLE1	19.5122	17.2292	19.022	20.0559
RAB14	29.8646	26.1198	25.9351	29.0203
CNTRL	1.81438	1.67674	1.57055	1.5995
TRIM32	11.6368	12.1335	13.6072	13.3719
FBXW2	31.536	20.8285	22.3455	25.0475

PHF19	65.8376	50.2458	54.3081	51.5569
NEK6	22.9046	21.6814	22.1228	21.9069
BSPRY	10.7331	10.0638	11.6806	10.7896
PPP6C	57.9106	53.8742	49.2329	56.815
NDUFA8	85.9768	81.6505	80.2953	85.47
HDHD3	11.641	13.5334	14.1938	12.8674
RBM18	21.6169	19.7252	19.8561	22.9511
HSDL2	24.6744	26.8226	26.8996	27.0706
MAPKAP1	77.0166	75.7752	76.8739	77.2862
INVS	5.68322	6.12961	5.79675	5.85977
GALNT12	5.05215	5.90097	6.56869	6.44316
DENND1A	12.3624	15.6569	14.5461	16.3955
ALG2	23.2201	17.8285	15.333	17.6823
KDSR	38.401	36.4108	38.5729	36.3979
VPS4B	27.2803	24.3104	25.8854	29.8543
C19orf25	50.1035	52.7813	58.614	52.6349
ZBTB45	12.1573	13.841	14.2488	12.3019
YLPM1	13.0035	13.2681	15.0133	14.6219
DCAF4	7.83405	7.78659	8.17247	8.37359
FCF1	57.1473	49.8359	47.1564	53.0609
PGF	5.72492	5.50407	5.04441	4.90751
IFI27L2	45.6182	45.6953	48.4594	47.0237
CCDC176	1.00215	1.18101	1.17365	1.16913
NEK9	14.5022	15.8513	12.6787	13.1427
ACYP1	24.8914	18.9374	21.1459	26.0117
IFT43	18.027	21.0618	19.2739	19.1819
NPC2	110.793	112.678	114.937	121.3
DNAL1	8.59361	6.22266	6.88399	6.83491
IRF2BPL	19.1517	18.6885	20.0889	18.9818

LTBP2	0.914853	1.29708	0.918361	0.753669
AREL1	22.6222	15.7207	19.8213	21.5308
MLH3	7.20629	7.0469	8.25795	7.47202
TTLL5	8.38207	7.76601	8.51099	8.23683
ABCD4	25.7334	28.515	26.7697	26.004
DLST	67.5894	53.0681	57.2768	57.1139
PPP4R4	1.39369	1.63279	2.23653	1.77016
SLIRP	371.548	337.014	282.526	349.043
RBM25	38.7448	30.597	29.1706	35.3741
ALDH6A1	3.0883	3.38186	3.32755	3.51137
EIF2B2	44.295	31.8653	34.445	36.307
NRDE2	8.57548	7.32372	7.10608	7.63717
COQ6	20.8216	20.1196	22.8921	24.4058
ZNF410	28.4519	26.0322	24.0948	26.6035
RHOQ	16.1109	17.364	14.4597	17.6309
SUPT7L	14.4858	14.7406	13.6796	14.2287
KLHL29	4.68302	4.12093	5.36855	5.23424
DNMT3A	3.68647	4.74418	3.92692	3.30222
TMEM214	48.5484	44.7751	48.2129	41.0132
ATAD2B	1.61877	2.22753	2.15816	1.81437
FKBP1B	6.49535	7.51961	8.45388	7.79102
ATL2	39.8265	30.8258	33.7129	32.5113
YPEL5	5.89827	5.80151	6.19046	6.62447
FAM98A	73.8167	59.7702	67.9587	68.1701
YIPF4	14.7919	16.9758	16.9603	16.8561
AFTPH	9.87154	10.0502	9.86591	10.941
LGALSL	14.211	11.2456	10.641	12.5036
CRIPT	6.51276	7.38743	6.27862	6.93191
EPCAM	196.944	163.787	154.922	178.394

SLC17A5	13.59	11.0841	11.9991	12.8429
OGFRL1	4.41997	3.66416	3.76804	3.88655
FAM178A	10.0081	8.42311	8.58593	9.66607
IDE	23.7275	22.0464	21.6794	23.8061
ELOVL3	4.60852	4.09168	4.14305	4.31611
GPAM	11.3831	11.8726	11.2098	12.2844
CUTC	61.1123	47.5391	47.3104	56.5067
PYROXD2	2.62264	3.50634	3.76614	3.56839
MXI1	12.2196	15.139	13.7773	14.0316
SMNDC1	46.3274	35.6338	36.0512	42.4692
C10orf88	12.4081	10.7568	11.1126	13.3866
HELLS	25.4308	20.4804	20.2975	22.8152
TCTN3	36.76	32.3899	32.3158	33.7232
FAM45A	27.3891	29.2513	27.8648	26.7114
AVPI1	14.8331	13.6734	15.7286	14.803
WDR11	17.9491	21.2175	19.552	20.3663
C10orf76	13.7596	14.0566	15.3037	16.9251
KCNIP2	1.2953	1.53973	1.62711	1.34819
GOT1	75.6614	93.0124	90.8356	82.206
C10orf95	2.89923	2.97304	3.0312	2.82207
GNA13	20.4762	18.1514	17.6471	20.4969
HOXB8	49.5974	46.7223	60.126	57.6751
KANSL1	13.4937	13.6503	14.9259	14.0207
HOXB5	4.56906	5.57833	6.37579	5.70681
HOXB3	5.20395	4.66826	5.46169	6.38683
DUSP1	5.73602	5.12146	5.2229	5.43819
PANK3	21.0753	18.0638	17.8011	19.3287
MSX2	11.4269	11.7644	10.7258	10.9229
RCL1	26.8072	23.7153	22.6332	23.8134

CAAP1	14.5697	16.0546	16.4357	15.3446
NUP43	28.6866	25.1023	26.5954	26.4129
MTHFD1L	60.7778	70.8384	66.3783	67.4633
LRP11	8.49203	11.4549	11.5165	11.1234
PCMT1	84.4526	65.6868	71.9497	74.7928
PLEKHG1	0.992794	1.12645	0.997045	0.878113
CYSTM1	63.9908	62.9398	66.6321	67.8455
WDR55	17.547	14.2731	16.8031	16.2231
ARAP3	5.15303	5.53568	5.819	5.39568
MRPS14	21.8293	21.6096	21.1375	23.3597
CENPL	8.77872	7.7111	6.88854	8.32983
TNFSF18	1.00873	1.21823	1.9256	1.78635
GORAB	5.27166	3.77144	3.77504	4.12097
ACAT2	25.7071	17.4643	19.8787	20.8598
TCP1	482.837	391.954	397.063	406.965
SNX19	12.315	10.915	12.0721	11.3405
MSANTD2	3.1172	3.17842	3.10908	3.05228
PDZD11	36.013	32.5961	30.5805	31.0544
SLC10A7	2.71133	2.24698	2.42288	2.70408
NUDCD1	45.3422	36.8809	35.5538	40.975
ENY2	165.982	145.911	134.24	167.682
MASTL	10.1452	6.95162	6.92868	8.06517
KIAA1217	0.879235	1.67138	1.40585	1.18601
EPC1	10.6323	10.0661	11.5122	9.447
CCDC77	8.61742	6.90633	5.85588	6.33927
TAF12	41.7936	38.0441	43.4257	45.296
ENOX1	2.9381	2.45646	2.59313	2.51199
MTRF1	5.94416	4.67391	5.1137	5.54713
DNAJC15	13.718	11.4034	11.6467	10.6064

PROSER1	20.5042	19.8291	19.5737	18.4872
UFM1	20.451	19.3647	18.1838	21.0545
WBP4	13.3213	12.4153	10.8255	13.509
ELF1	11.3871	12.157	11.1969	12.3006
HSPH1	120.227	91.2147	87.0001	108.904
KBTBD7	2.73922	2.53473	2.52873	2.46936
ALG5	29.1962	24.3931	21.3373	23.4534
EXOSC8	95.0637	86.7427	78.1432	87.064
ETF1	123.717	111.107	105.869	128.869
FAM53C	17.0994	12.7724	14.3917	14.0263
SIL1	18.4891	20.7891	24.9717	24.0074
PAIP2	70.2942	73.6756	65.4389	69.6493
KDM3B	23.1339	23.7509	25.1849	24.4415
EGR1	19.4776	16.6216	20.8398	21.7565
SERP1	93.4952	77.3763	88.3618	84.6806
PLS1	15.7988	15.6235	14.867	16.1799
ZFP30	1.34194	1.15891	0.947056	1.20865
NR2C1	15.7493	14.0408	14.684	15.0879
UTP20	8.18252	7.42742	6.82991	7.35722
TMPO	93.4869	66.6401	71.6807	80.696
ARL1	52.2486	50.3259	46.7796	53.372
GLT8D2	2.13081	2.35492	2.34145	2.34818
MTERFD3	4.08575	3.61661	3.43589	3.47767
SOCS2	15.3287	9.53295	11.9187	13.1993
NFYB	25.3672	23.9331	23.1851	24.7518
CCDC53	7.09167	8.59271	8.71604	10.535
APAF1	2.43816	2.89841	2.5115	2.46259
DUSP4	29.9252	33.2538	33.4985	31.5509
CLU	331.21	318.741	312.934	318.777

TNFRSF10B	45.1098	63.1577	51.9191	49.0925
SORBS3	90.318	71.878	77.9752	80.4786
PTK2B	2.1977	2.76211	3.06933	3.00966
PPP3CC	9.82264	6.6487	6.80732	7.55559
PDLIM2	37.6952	27.1768	32.9808	33.6086
EPHX2	7.51949	10.5254	8.58931	8.42917
RNF170	7.06114	4.48548	9.42382	8.91525
UBIAD1	14.7688	13.3512	14.0817	14.1397
TARDBP	120.504	94.0522	94.9515	103.977
ZNF706	131.265	114.359	104.333	115.06
LYPLA1	90.503	81.9756	84.5419	86.8434
COPS5	93.2834	72.2036	77.7722	84.6378
RDH10	24.7785	15.0112	21.5665	21.1617
AKAP1	69.8814	52.9406	60.7562	61.0786
COIL	19.3738	18.2475	18.6533	19.9904
TRIM25	44.4912	34.9883	35.5097	40.2408
SCPEP1	23.353	23.9419	24.8205	23.797
SPOP	52.1081	48.586	52.2115	53.0093
SLC35B1	125.22	107.689	104.511	113.957
DYNLL2	141.14	99.6762	121.52	130.45
FAM117A	5.06156	6.28998	6.06363	5.79811
NCAPH	46.9368	33.9286	36.6701	37.8436
LRAT	1.45196	1.24087	1.14493	1.12019
KIAA0922	6.54046	7.96189	8.50818	8.37733
MND1	26.6059	22.3704	21.4684	23.4526
TRIM6	1.53007	1.22701	1.39559	1.44116
PAPD5	9.22825	8.3126	8.90141	9.96839
ADCY7	20.8501	13.8802	17.1407	16.522
CEP89	7.91774	6.89807	6.69868	7.83168

ECHDC2	12.0344	13.1657	13.1424	12.1898
PYROXD1	7.47964	8.62122	8.27465	9.51442
PSPC1	42.3773	40.6652	41.7494	44.0503
ZNF211	1.95197	2.95782	2.46529	2.88504
RNF2	23.9867	18.8395	21.6529	23.1392
TRMT1L	12.8103	13.8146	13.9725	14.5065
SEC22A	12.1514	11.6465	13.1229	13.289
CSTA	4.81173	10.756	5.82499	5.39512
B4GALT4	9.50056	6.62466	7.54831	7.67174
NAA50	128.094	90.5523	109.299	115.965
KIF18A	14.4266	13.043	13.1775	14.5579
DESI2	29.7639	21.2554	21.3451	23.3835
MAPK8IP1	6.14864	7.44852	7.69084	8.00043
CRY2	4.61084	5.30352	4.73084	4.4078
PEX16	33.8588	30.9113	36.1592	32.6736
DEPDC7	5.90097	4.83898	4.48665	5.06084
CAT	20.8906	21.7112	23.4991	22.8365
PILRB	44.3399	48.8892	45.9782	42.3401
ZMYM2	7.59941	9.02865	7.58687	10.765
GJA3	3.00309	3.4283	3.63805	3.77466
TBC1D15	15.688	14.1824	12.5601	14.7424
BAI2	6.50292	7.17379	8.16625	8.99908
ZCCHC17	28.0252	27.5179	28.2819	26.4849
KHDRBS1	132.715	127.311	132.946	131.192
TMEM39B	14.9194	13.4993	14.0274	13.6969
RNF115	9.72052	9.09307	8.68382	9.27432
POLR3GL	7.15391	8.47842	8.28281	7.82398
ZNF639	32.4902	28.1496	27.8826	30.7272
PIK3CA	3.9961	3.60349	3.89491	4.35496

PDS5A	38.4934	34.3686	34.9908	38.0282
LIAS	16.7549	13.0274	14.4502	14.5245
TMEM54	89.1866	101.108	91.8499	85.1431
ZSCAN20	1.01951	1.09233	1.11676	1.0356
LRIF1	9.92962	10.182	9.74042	10.7146
CLCC1	17.7174	16.3204	14.3819	21.3026
GPSM2	26.701	24.8963	22.5575	25.954
GTDC1	6.91993	4.4492	4.84183	5.4644
ZRANB3	2.16862	2.93567	2.56123	3.01856
ACVR2A	3.70169	3.22806	3.43374	3.54256
POLK	3.96314	3.93706	3.4833	4.32728
RPL21	2410.5	2261.18	2139.38	2188.72
MTIF3	16.5051	18.1206	18.7183	20.4523
GTF3A	88.553	86.8567	83.2811	93.4913
RASL11A	9.78252	7.79353	7.81325	8.69444
UBL3	4.70818	4.15509	4.59446	4.91881
FYTTD1	33.6809	28.3926	29.0448	33.0612
MTERFD2	26.5702	22.2484	23.9688	24.7332
OCRL	9.11673	8.31616	8.85349	9.53596
MRPS2	114.182	104.056	110.921	102.004
KIAA1191	50.7258	40.5923	41.0796	42.9141
COPA	46.4405	40.1379	41.122	41.6195
RBBP6	29.4727	23.6757	25.416	26.5296
ZC3H7A	20.4467	16.0098	15.3028	18.7714
SERAC1	4.56142	3.53049	3.71445	3.93164
ANXA11	136.933	131.231	143.485	157.26
FAM35A	51.2889	42.2477	44.7574	46.0973
FAM213A	1.45414	1.51232	1.32304	1.39842
ZNF205	18.2609	14.4843	14.8376	13.628

NAA60	24.198	24.3448	27.903	23.4707
RPL5	1252.5	1246.67	1197.46	1245.47
ODF2L	6.04227	6.24022	5.96391	6.1072
SPATA1	0.903482	1.59207	0.901124	0.855224
TRMT13	5.1659	4.45857	4.69819	4.78933
RWDD3	9.84476	9.07004	7.79584	9.32863
ZNF644	16.6604	16.5818	15.6748	16.9492
CCDC18	17.0785	13.2069	11.7078	13.2001
RPAP2	10.2204	10.8467	9.56181	12.0016
PQLC1	38.0158	33.5141	35.4091	33.8909
NBPF14	6.9587	6.36507	5.90302	5.8412
BBS9	2.33821	3.18309	2.68035	2.63961
PMS2	10.8424	11.1221	11.8505	12.1044
ZMIZ2	24.5788	28.0985	31.0267	27.506
7-Sep	65.95	56.4137	51.7046	60.4307
EEPD1	2.32459	2.9345	3.74589	3.51791
KLHL7	15.3377	15.0137	15.8457	14.9701
HERPUD2	7.66452	8.55169	8.12677	8.32419
CBX3	214.518	183.534	173.406	201.055
HNRNPA2B1	1013.85	800.71	773.808	890.012
WIPF3	3.25152	3.68857	3.56361	3.73484
FAM126A	6.89349	4.77684	5.10298	5.67041
HOXA7	13.6182	8.47913	6.0904	5.77045
FKBP9	29.8934	30.5409	33.4789	32.5057
NT5C3A	23.1551	19.9764	20.161	22.2402
ARL4A	24.3279	22.7729	20.2332	23.9267
CCZ1	31.9669	30.3806	28.6696	33.5837
POLM	6.4697	8.04896	8.3258	7.13621
FTSJ2	41.2958	37.665	39.67	40.1431

TWIST1	1.07338	0.951399	0.865733	0.862856
SMU1	58.6525	54.0661	51.3085	55.2277
GLIPR2	11.5816	10.0367	8.96365	9.72063
SLC25A51	16.6704	15.6261	16.472	17.6176
CLTA	153.638	141.35	149.867	148.696
RECK	1.51353	1.74419	1.56899	1.74787
ACO1	19.7978	20.5027	20.0065	20.2974
DCAF10	8.92858	8.64473	8.35228	8.77249
TRIM24	7.86438	6.16357	7.26079	7.22837
C7orf49	33.3428	30.1862	30.9344	30.0673
CALD1	47.0013	56.3012	49.1592	49.9284
PLAU	27.8143	23.5235	25.7968	26.1745
CHST3	7.88058	4.80859	5.87748	5.88191
CISD1	57.2469	51.0914	51.468	57.7336
ECD	18.1499	15.7569	14.9984	16.4154
P4HA1	11.1653	8.2832	8.15764	9.16983
SLC25A16	6.03991	5.6811	6.29669	6.2484
ZWINT	123.619	90.7649	84.6409	93.6963
VPS26A	28.8966	27.0483	26.9891	28.5409
RBM19	12.0712	10.6689	12.4331	10.6744
CIT	7.82575	7.23833	6.80186	7.26701
IFT81	3.17859	2.9491	2.45659	2.86201
ACADS	7.43958	9.92513	10.2803	8.77402
DDX54	128.576	114.293	119.629	121.748
MED13L	29.4854	29.6341	27.4794	28.9676
CDKN2C	4.60713	3.66332	4.28853	4.92923
RNF11	56.1598	59.5831	57.2645	58.9137
RASSF8	2.44142	1.55286	2.2953	2.59427
ITPR2	0.677688	0.816196	0.822743	1.13882

CCDC91	8.51653	7.127	9.06621	8.42164
WWP1	27.4405	25.9476	24.7325	27.5959
ACOT9	42.9519	39.4403	40.199	43.556
PRDX4	173.039	148.956	141.731	152.272
DDX39A	155.83	114.825	123.71	121.348
PKN1	84.9028	95.6599	98.5732	91.7255
C19orf43	172.784	158.851	159.403	165.498
CD97	31.1368	34.6716	37.4663	32.675
WDR83	18.1898	14.6185	17.162	16.6337
GIPC1	93.8363	87.6658	98.5604	93.5094
SPRYD7	11.9873	10.5188	11.2089	11.5984
EBPL	73.4738	67.7648	61.1106	66.6658
ATP7B	1.75513	1.9481	2.52578	2.39108
ZC3H13	12.8251	12.7503	13.8644	13.7734
GUCY1B2	0.649684	0.45144	1.06451	0.432766
NLN	33.774	32.2759	31.0645	33.9324
CENPK	13.3738	12.039	10.8411	11.6386
OPTN	7.34228	6.58773	6.94028	7.37994
ATF1	31.1022	25.9619	23.7404	25.3564
TSFM	49.2574	48.598	46.2394	49.3637
MMP19	0.673646	1.41824	0.896402	0.979993
PFDN5	408.749	388.232	366.73	370.913
SPATS2	61.5359	58.0073	61.9907	59.7487
ORMDL2	34.7153	35.0495	35.9206	33.6968
NR4A1	23.1117	17.6225	19.6675	19.4797
HOXC13	2.65776	2.63016	2.54923	2.81871
CDK2	116.905	94.5087	97.7488	108.442
LRP1	1.01382	1.98109	1.20016	1.11942
C12orf44	69.67	60.2338	67.6683	64.5356

IKZF4	2.71603	1.50168	2.25125	2.03188
SMUG1	41.2606	47.0124	49.0167	44.359
TUBA1B	2531.86	1694.97	1896.83	1987.83
METTL21B	2.5297	2.38659	3.32534	2.92526
KBTBD4	13.5556	11.0021	11.1167	11.0848
SARDH	0.882673	1.6276	1.49602	1.36299
ATPAF1	46.8734	48.9123	43.5137	43.9206
STIL	21.0082	16.6765	15.4476	17.683
HJURP	49.2598	28.2229	35.9567	36.3367
AMD1	63.7141	54.5438	54.7563	61.6068
NDUFAF4	68.3671	59.8833	55.208	65.4104
USP45	7.20935	7.34853	7.91679	8.3083
MORF4L2	151.798	117.931	118.273	134.071
FAM199X	6.36608	6.22869	6.16298	6.8611
RAB9A	14.3078	12.409	12.6877	13.3927
METTL8	17.2129	17.2706	15.4295	17.5077
TTC21B	4.03976	4.65275	4.25946	4.55257
NMI	2.55176	3.00267	2.7567	3.14789
BAZ2B	2.76136	2.4748	3.01331	3.96349
SLC36A1	4.77581	5.75901	5.13923	6.05309
LPGAT1	14.9063	11.2546	10.1081	11.0312
BATF3	6.68496	6.63333	6.56561	7.3441
RAP2C	14.9272	12.2161	13.7946	13.9578
EXOSC9	77.0869	60.7803	59.5922	70.9905
PLA2G12A	74.2578	52.5068	53.2522	55.25
B9D2	7.8689	5.66404	5.78487	5.88437
ADCK4	16.1734	19.8155	22.1875	18.9642
PFKFB2	6.14143	5.56271	6.17495	6.65875
RAB38	7.97641	8.84029	9.18466	8.8592

AGO2	43.6758	30.4161	37.2339	33.8659
MXD4	5.15167	8.51489	9.26345	7.39992
PMS2P5	3.92984	3.90571	4.92995	4.71565
CKS2	299.405	221.17	208.328	234.893
ACSL3	51.4998	39.3425	36.6594	46.8162
CHPF	204.955	235.409	253.145	216.024
DNPEP	103.541	108.681	105.621	104.111
OBSL1	42.7213	40.8446	39.7757	37.5164
SLC12A4	18.2811	22.3086	24.5664	21.2719
ENKD1	13.0697	13.8943	13.1736	12.4403
FAM210B	5.48988	9.66417	9.01384	8.2112
SNX21	5.64598	7.51209	7.18445	6.47507
SLPI	0.877758	1.5015	1.39215	1.17942
WFDC3	2.1511	2.63998	2.02029	1.91506
TTPAL	6.26371	6.01451	4.71373	5.51074
PREX1	1.54071	1.87782	1.99328	1.83664
SDC4	63.0579	52.5967	62.2294	65.5136
NCOA3	5.05053	5.60679	5.78491	6.1061
PIGT	52.2536	55.1529	64.2919	60.0046
NCOA5	18.011	18.6877	18.5361	18.8378
VAPB	30.1071	27.3074	26.7558	28.1846
PARD6B	5.16956	2.96648	3.38986	3.75213
ATP5E	229.724	181.061	168.004	204.113
CHD6	1.9296	2.3643	2.23793	2.19572
PLCG1	23.1076	25.8181	28.6359	24.5446
TOX2	13.795	12.6563	13.4107	14.295
SRSF6	150.017	129.325	128.578	147.363
ARFGEF2	7.05827	7.37979	6.89307	7.97225
ZNFX1	12.2388	14.0018	13.814	13.7533

CSE1L	148.572	123.84	121.106	140.733
TMEM189-UBE2V1	1.57883	4.55464	4.90898	0.979734
RAB22A	5.11666	2.37708	2.36353	2.93082
STAU1	45.4132	38.025	38.3938	41.5817
SNAI1	9.33765	7.62982	8.01335	7.95351
MOCS3	8.87501	9.32871	9.73563	9.56019
STX16	16.9731	15.037	15.4105	16.6517
PPP4R1L	2.82425	2.96153	2.53561	3.08039
RNF114	34.3964	32.4579	32.2497	33.8585
DDX27	25.5244	25.0364	25.6861	24.3779
BCAS4	22.6691	22.1483	21.536	20.7281
MTRR	31.3033	28.3731	26.7636	29.3018
FASTKD3	17.1668	12.2473	13.0326	14.7418
PEPD	34.7694	34.6174	35.2369	35.8593
IQSEC2	2.07611	2.85378	3.21067	2.92292
VAMP7	24.229	20.0991	18.3744	21.6779
STAMBP	27.2273	25.1285	25.8346	26.2862
NAGK	25.1532	24.7873	29.8334	30.1983
MCEE	3.18807	3.87539	3.87389	3.67334
PAIP2B	0.841702	1.11588	0.904765	1.01334
SNRNP27	32.1816	28.1306	26.8409	30.5998
MPHOSPH10	19.4216	17.3413	15.9157	18.0642
USP22	86.598	84.9401	77.279	80.737
ZNF576	14.359	12.8417	12.7712	12.9724
ZNF45	4.8975	4.26558	3.98562	4.84825
LYPD3	2.26294	2.61406	2.59072	2.57085
USP9X	12.9592	13.1926	11.8531	12.8925
TRERF1	2.87588	2.70647	2.8457	2.87897
PACSIN1	1.44086	1.57566	1.63523	1.65916

BTN2A2	5.62939	4.31215	4.44343	4.36511
SIRT5	6.33969	7.10429	7.31979	7.69207
MRS2	33.6776	33.6863	31.1837	32.2172
WRNIP1	72.0193	66.1076	70.0731	68.4141
RRP36	79.7999	66.2175	65.2798	68.8415
BTN2A3P	0.993106	1.08467	0.821119	0.758784
SNRPC	313.717	296.246	290.002	291.948
SERPINB6	33.6688	37.6593	36.5096	38.0741
XPO5	89.7624	85.1971	104.697	88.7866
ABCC10	9.56032	11.1406	14.2975	11.7252
PEX6	8.62086	8.22673	9.27399	8.54935
NQO2	68.6344	62.1663	57.6526	56.9854
PRICKLE4	2.60729	2.56474	2.40737	2.01447
OARD1	15.5897	16.5307	15.6085	18.5473
AARS2	7.94588	7.4846	7.89181	7.48843
RPS10	1701	1749.86	1734.91	1738.52
MOCS1	5.07451	5.43268	6.41396	5.76517
HIST1H2BJ	1.13055	1.11923	0.675232	0.550304
MED20	17.3973	15.7277	16.4574	17.379
TBCC	15.2676	14.9036	14.7053	14.7885
MAD2L1BP	36.6527	31.1971	33.1057	32.3518
KLHDC3	45.4834	43.6145	42.1894	42.4739
MEA1	78.3624	70.6574	66.3057	67.2535
CDKN1A	107.488	229.121	170.128	145.187
SOX4	16.2843	23.2221	21.3444	21.3091
GLO1	154.546	141.835	134.69	151.107
RREB1	4.15977	4.70769	4.9841	4.5329
SSR1	75.3003	74.2822	72.3338	75.0122
RIOK1	34.1997	20.4967	24.6984	24.807

SLC35B3	7.03967	6.76444	5.92619	6.41127
RPP40	23.6423	20.4633	19.6715	22.681
ATXN1	1.71345	1.89812	1.70279	1.63117
NUP153	24.8989	19.8269	20.311	22.9712
DEK	53.7462	44.4811	40.4331	49.0698
EEF1E1	122.195	94.1966	91.008	99.0597
LRRFIP1	30.6991	24.2229	23.3837	24.7453
RAB17	15.7067	18.5803	19.6414	19.1597
EREG	133.712	148.497	162.168	162.919
MYRF	16.8974	17.8037	18.9697	17.7781
AHNAK	19.6391	20.9575	20.2423	19.944
EMC3	25.4424	18.9329	19.5169	22.6024
SH3TC1	1.21314	1.72188	2.19971	2.2488
CNOT1	148.737	132.889	140.712	137.48
BBS2	22.4189	25.2774	26.8889	28.8764
MT2A	1004.72	1203.28	1300.08	1164.48
C16orf70	24.8413	23.9014	22.8855	22.0861
GOT2	95.519	89.1498	88.7093	92.9376
DOK4	6.91187	5.84893	6.75709	6.61175
CLYBL	5.17697	7.52762	6.41406	6.22743
TMTC4	6.12056	8.33995	8.67001	7.72952
RAP2A	9.28835	8.58291	7.72154	8.66562
ABCC4	11.0274	9.96637	9.83243	11.1922
EFNB2	9.65901	7.06142	7.97449	8.65277
TM9SF2	53.7574	47.2729	47.6347	52.0102
C17orf53	6.72351	6.03845	6.2966	6.58426
IRF1	8.82996	9.00331	9.64029	8.81211
UPF3B	11.1452	9.64034	9.70034	10.865
RNF113A	4.69906	4.13764	4.51628	4.39843

6-Sep	4.99119	5.64859	6.33972	6.68272
NDUFA1	124.675	113.584	112.31	124.68
ATP5S	3.36862	3.77116	4.17719	4.06975
BMP4	101.47	110.418	133.081	124.217
FAM193A	5.72352	5.35923	5.54366	5.35371
GRK4	3.67181	3.43256	3.93367	4.03668
SOX9	22.2126	27.6759	25.4721	25.3309
SLC25A35	1.08086	1.47072	1.72327	1.68089
MRPS7	139.783	140.421	141.837	137.85
GGA3	29.3923	30.9373	32.6254	31.5214
ARMC7	30.1459	27.0878	28.1812	28.8447
NUP85	168.425	131.074	126.137	139.141
SLC25A19	55.5768	38.3154	41.9835	44.4481
MIF4GD	16.3806	18.1291	18.3365	17.7138
NT5C	99.5988	100.809	107.859	97.5131
MSTO1	41.331	44.0059	42.9993	42.4361
C1orf61	7.22834	7.14947	6.7549	6.22068
TTF1	9.30982	9.00304	8.71059	11.7085
GTF3C4	14.8948	11.952	11.9751	13.1472
DDX31	17.5285	16.0281	16.3564	16.1898
PPP1R12C	36.1243	40.6855	43.9923	38.1649
MBOAT7	11.7432	13.8214	12.5082	10.4317
SRMS	2.46664	2.75855	2.93783	2.67777
SLC2A4RG	117.047	125.207	125.304	113.527
PPDPF	257.022	283.919	264.984	230.337
CHCHD5	49.9768	51.6056	53.0193	49.8319
PAX8	4.89406	5.87787	5.69015	5.4084
INSIG2	10.8568	7.90857	8.41562	8.94304
POLR1B	48.01	38.5196	40.065	43.2647

CCDC93	7.74965	7.32337	7.47481	8.89788
PSD4	3.01953	3.53516	4.33731	3.81215
SLC25A23	34.6928	37.5072	42.2835	37.8878
PSPN	1.56009	0.847852	0.698293	0.892479
GTF2F1	77.5386	54.4485	59.0157	60.5843
ALKBH7	27.5598	35.0554	38.2156	34.3066
CLPP	124.262	112.298	125.457	113.435
TNFSF9	20.309	25.0977	24.0013	21.4585
THOC2	22.0716	19.0811	19.5091	22.3257
MED1	22.2521	20.6164	20.7918	22.7186
RPL23	1832.11	1743.12	1676	1863.69
ATG4C	9.963	9.83459	8.31101	9.05635
SH2D3A	43.3718	35.146	40.4848	39.5505
TRIP10	71.1919	73.808	66.8467	66.6461
GPR108	39.2004	51.5377	50.2346	41.0577
OPA3	6.58283	7.28024	8.05015	7.73774
SNRPD2	1402.78	1347	1295.86	1275.34
RTN2	7.178	11.3493	11.2434	9.01175
EML2	52.8833	65.5018	80.1743	68.6482
VASP	85.3132	84.8937	104.661	99.6768
SYMPK	68.8715	74.1877	69.624	71.4344
GPCPD1	6.74848	8.72903	8.56402	8.84076
PANK2	16.8338	18.2859	16.3042	18.0744
GZF1	10.1897	10.7498	11.3706	10.6525
NAPB	2.52835	2.42239	3.16912	3.00774
CENPB	36.8436	35.8539	38.1174	35.8466
PSMF1	140.213	131.012	132.851	146.072
DTD1	39.722	38.3072	36.3066	38.9785
RBCK1	44.1804	58.1931	50.114	45.3628

TMX4	3.06502	2.54249	2.9677	3.62873
STK35	16.2222	13.1979	15.6606	15.7261
SNRPB	483.316	418.026	435.999	439.43
AP5S1	7.01667	7.47775	8.04253	7.60729
RRBP1	41.0752	40.9243	42.9652	40.8798
ZNF133	2.85083	3.86174	3.74628	3.62199
MKKS	23.4981	23.516	21.21	23.379
BFSP1	2.35399	3.70995	3.20906	3.25052
DSTN	137.97	125.912	119.894	130.79
SNRPB2	83.7504	72.347	68.8079	82.4936
MGME1	30.4388	26.3416	24.4175	27.2751
TBC1D20	12.1932	10.3439	10.698	10.4749
ITPA	70.8385	72.9336	71.7517	70.0179
MCM8	13.2565	10.5078	11.6229	13.1469
TMEM74B	5.47852	6.50558	6.40195	5.73144
FAM110A	13.1512	12.6377	14.1223	13.2774
MRPS26	102.957	89.0027	92.9774	88.5211
NCLN	120.824	95.6345	111.879	104.353
CITED1	3.35838	2.72263	2.63634	3.45806
HNRNPR	141.704	112.151	106.349	119.138
ZNF436	2.75502	2.84207	2.73342	2.86167
MAX	39.8728	35.1492	37.712	36.6408
CHURC1-FNTB	20.4671	18.5295	19.6834	20.5669
ARMCX5	5.42426	4.60829	5.85226	5.13588
MMP24	1.71231	1.67441	1.69002	1.4815
NECAB3	28.8839	30.3718	30.377	27.0597
ID1	296.05	312.124	312.708	279.812
RALY	109.367	100.537	110.934	102.796
DYNLRB1	120.754	125.383	128.975	119.387

EIF2S2	74.6655	74.2973	69.0112	70.5593
ERGIC3	167.591	172.146	168.978	165.342
ROMO1	281.746	271.269	249.11	278.264
CEP250	9.4486	10.2619	9.40787	8.7404
PLAGL2	11.8079	10.1115	11.508	11.2063
MMP24-AS1	24.121	27.7419	26.395	26.5216
KDM5C	11.5227	11.6694	10.9797	11.1244
AMOT	1.25324	1.14924	1.04217	0.972097
TMEM115	20.8574	19.2259	20.1163	19.3467
PSMB2	116.572	112.38	112.238	115.609
AGO3	7.82945	6.35388	8.07725	8.02289
UROD	77.2393	70.4729	73.4004	73.5604
ST3GAL3	0.813282	1.8258	1.50387	1.48191
TMEM53	6.15312	7.00192	6.99391	7.46692
HECTD3	15.5959	16.2197	17.696	16.4814
KLC1	57.0434	61.1262	79.3185	67.6572
XRCC3	28.6968	21.6835	25.8074	22.8648
TUBGCP3	30.333	26.5516	28.981	30.2543
MCF2L	3.09431	2.93614	3.38666	3.40505
PCID2	55.2841	48.4623	48.4443	51.6609
LRFN3	13.3723	14.1989	15.0684	13.786
IGFLR1	6.28773	8.67577	8.48269	7.21748
CAPNS1	581.351	559.242	364.233	376.175
PDCD2L	35.7609	29.8457	31.5487	33.789
RBM42	70.2624	62.6078	65.9552	62.1627
UBA2	87.4381	66.4379	62.8352	67.6411
HCST	0.920096	1.00361	0.921059	0.695358
COX6B1	487.35	453.515	434.638	431.774
THRA	9.08553	9.62305	9.72908	9.13056

NR1D1	15.538	15.7867	15.3771	14.8025
FRMD8	20.9924	19.2902	20.8119	20.1756
PRDX5	253.797	266.323	280.977	269.404
BCL2L12	125.373	121.63	118.254	119.088
IRF3	74.4648	69.3787	75.833	69.4779
PRMT1	419.025	399.365	414.937	384.539
RRAS	32.355	30.8276	33.4919	32.6752
PRRG2	3.24863	4.15166	4.21866	3.84284
SCAF1	33.7084	33.1938	35.9372	33.2528
PRR12	7.41955	6.62366	6.65693	6.74741
ASL	34.3053	42.993	35.5121	33.9687
SBDS	53.8639	40.4537	41.7353	45.6305
WNK4	1.15237	1.18119	0.882502	1.2191
BECN1	48.8155	53.0477	52.0077	51.7587
TRAP1	261.966	254.203	272.652	246.399
GLIS2	6.19348	5.00173	5.475	5.24362
NSRP1	14.7795	11.9664	11.5763	13.2663
DNAJC8	77.1536	70.35	63.1992	70.3194
AHDC1	6.73274	6.24677	7.70692	7.00827
IFI6	8.01787	9.10853	7.54999	7.89831
ZNF384	41.64	35.5898	40.865	39.3789
EMG1	49.6404	41.0255	40.8025	43.1164
UXT	113.487	108.956	103.326	111.454
ELK1	34.4856	33.4117	36.137	33.1946
TIMM17B	13.7612	16.0522	16.4161	15.1404
PCNXL4	20.0199	21.0567	21.2753	20.4725
ATG14	4.79087	5.1623	4.31264	4.63058
KTN1	42.6853	38.8775	35.2581	40.1911
SIX1	2.99605	2.22093	2.66184	2.84403

DLGAP5	31.2099	27.3023	23.7488	27.9868
L3HYPDH	6.48624	7.37899	5.89622	7.04851
HSPA2	2.77053	1.94697	2.33208	2.80811
ZBTB1	7.26699	7.15567	6.76398	7.37204
TRMT5	12.7943	11.5083	11.7464	12.9185
SGPP1	10.2053	10.7225	11.2417	12.4418
PLEKHG3	19.1896	15.3501	15.0614	18.1902
RHOT1	18.025	15.6917	16.9568	17.9685
WDR60	2.36703	2.75752	3.33163	2.32326
AIF1L	19.3156	18.922	19.2996	20.3139
FAM78A	1.11831	1.38341	1.44943	1.20212
NUP214	18.1692	13.4437	12.743	14.817
SLC10A3	9.55158	8.38329	9.0062	8.57273
MAP2K2	115.072	118.173	120.987	118.942
HNRNPH2	21.6034	20.1149	21.4901	22.054
TIMM8A	8.01371	7.13921	7.40596	8.8196
ZC4H2	3.96046	4.64534	5.14015	5.26997
CANX	451.904	397.454	385.929	425.84
CPSF3L	114.751	101.044	106.631	102.786
IPPK	20.0718	15.54	18.5986	18.8855
ZNF484	1.75842	1.53876	1.67137	1.69198
FGD3	2.48052	1.67213	3.10493	3.08695
HIVEP3	2.71224	2.34626	3.23347	3.1646
PPCS	34.7462	34.495	33.8242	36.2411
COX7C	1113.66	1114.4	1005.52	1143.3
TRAF2	20.5735	17.8705	19.3523	16.9541
ABHD8	1.51274	2.01356	1.8985	1.81194
HELB	1.01019	1.14517	1.1657	0.967076
RAP1B	75.2404	67.8982	63.3848	71.3475

RAB3IP	13.8131	11.1284	10.9495	11.3893
PTPRB	2.29357	2.0464	2.42834	2.80042
DYRK2	12.7337	10.6388	11.6863	10.4039
YEATS4	19.3455	19.0858	20.5906	21.0677
LRRC61	8.22918	9.19716	9.92281	8.13971
IDUA	2.78594	6.70237	8.10539	5.79486
FGFRL1	50.8588	45.2512	54.0938	48.1645
TMEM175	12.0815	14.0389	17.5002	16.6261
AUNIP	13.6671	11.0711	11.1876	12.5277
PIN1	75.451	70.302	71.051	70.3127
FBXL12	13.8392	14.2206	15.1684	13.2201
EMC1	73.627	56.8972	66.6687	74.1253
UBR4	49.4009	49.5496	49.2582	49.6751
HP1BP3	95.9037	91.4757	87.5385	89.4098
EMR2	2.85304	2.25678	2.29635	2.20341
SIN3B	22.0326	22.0582	23.4581	21.1898
SLC35E1	39.6061	34.7411	38.3791	35.0847
EPS15L1	17.0854	14.9911	16.2	15.9335
KLF2	33.3537	35.8678	35.2973	29.4258
UQCR11	256.319	250.929	242.739	254.676
GFER	70.9849	63.9183	62.8847	60.6797
SYNGR3	3.80882	3.32679	3.06781	3.03333
PKMYT1	79.6068	67.4166	71.4734	68.0604
WDR24	7.1226	5.83449	6.11206	5.90929
FBXL16	38.189	37.8827	60.0717	39.7528
CHTF18	50.7871	45.2977	52.2273	41.65
MACF1	40.5693	46.9163	44.7792	48.9405
SMARCA4	61.9475	67.2711	62.4104	62.7862
KDM4B	6.04729	8.5441	8.34281	7.29038

TICAM1	12.9309	12.9182	14.3376	13.7312
GAN	3.47837	2.2576	2.20091	2.75282
METTL25	1.72132	2.28662	1.61961	2.79256
IL17B	0.992334	1.49948	1.31656	1.11916
EMC6	106.961	98.7483	100.441	93.0591
METTL16	23.0964	19.3405	17.1495	19.6717
TUBA4A	84.5179	52.4213	58.5015	63.1695
AAMP	184.333	158.683	187.324	169.7
PNKD	48.6086	46.7996	46.9899	45.3169
RNF6	22.8886	24.2263	23.1508	23.5948
ECHS1	44.5909	49.3452	48.2466	46.6056
AKAP9	5.87894	6.9869	7.17232	5.19164
SHFM1	231.626	219.961	230.293	258.583
HIP1	2.96266	3.45203	3.28187	3.30363
PTPN12	35.641	36.4217	44.8547	34.1982
POR	33.0802	34.1266	35.6357	29.4236
STYXL1	16.4271	18.1253	18.2392	19.1236
GNAI1	100.652	90.2983	100.26	93.7641
PMS2P3	1.78362	1.69604	1.56129	1.75685
PEX1	5.8836	5.80929	6.35454	6.51828
MTERF	7.9923	8.42021	7.89278	9.87769
SGCE	10.3704	8.6577	8.01983	8.20344
RBM48	8.05258	7.11095	7.53624	9.38391
CASD1	1.71534	1.75399	2.25474	2.16625
ZNF780B	2.83448	2.91003	2.35296	2.7882
LRFN1	11.2406	10.5607	13.231	11.3861
ZFP36	20.5437	17.9134	22.0193	18.3921
SRD5A3	10.2562	12.3002	11.6123	12.2746
SPINK2	1.19654	0.982212	1.23105	1.22907

PAICS	274.335	228.77	218.901	245.289
PPAT	42.5439	37.7958	38.795	41.897
TUBGCP6	14.0025	14.2014	14.2149	12.8883
ADM2	1.09526	2.59309	2.16638	1.57055
DGCR6L	45.5987	38.8441	45.5476	44.566
DGCR8	21.0754	19.5972	21.3986	18.6163
ASPHD2	3.0097	2.66952	2.74257	2.75537
VPREB3	1.06673	1.59008	1.42548	1.14411
SDF2L1	148.698	103.53	129.511	126.795
YWHAH	145.819	124.859	122.957	131.677
GNAZ	2.04418	3.35219	3.92328	3.4583
ADORA2A	3.65309	2.51452	2.44025	3.22064
ATF4	369.004	462.291	384.691	363.6
A4GALT	12.0935	11.201	13.239	11.8467
CDC42EP1	88.9648	92.5533	89.9799	86.3187
TPST2	10.0597	8.71638	9.58356	9.06477
MPST	43.9971	44.6431	48.4708	42.3194
TST	9.17262	12.5471	12.9977	11.9479
APOL2	5.84058	6.66655	6.79661	6.49441
RAC2	31.6929	31.8485	34.4312	33.7027
LIF	24.0731	24.2303	26.153	23.2586
C22orf23	1.2683	2.05809	1.95938	1.90104
APOBEC3F	3.33581	3.76336	3.69958	3.43233
RIBC2	2.79821	1.81915	1.93122	2.27757
EMC4	102.807	93.6786	92.2501	95.9842
SPECC1	10.6652	10.54	9.15762	10.4106
CPA4	36.9195	19.1422	30.3725	34.246
DOCK4	0.904081	0.790727	0.730474	1.34548
POT1	17.1353	11.313	11.77	12.8307

ATP6V1F	175.169	164.357	178.553	179.686
NAA38	68.2968	54.644	48.6175	53.9429
CDHR3	2.95123	3.73902	4.2272	3.97295
PRKRIP1	6.28912	6.49675	6.00846	6.27728
VGF	14.9206	27.5188	30.0231	22.4742
PODXL	14.8801	14.5862	16.3463	15.3833
STRIP2	5.21464	4.11269	4.34699	4.39152
RABL5	28.5885	25.2443	21.8312	22.8913
MKLN1	15.9681	18.3796	18.4327	19.7192
DNAJB9	5.56002	4.51738	4.03049	5.65837
FLNC	19.4908	18.5007	19.5691	18.6604
CALU	92.618	68.0222	68.7351	79.8387
CCDC136	2.51478	1.87141	2.11887	2.30598
SMO	10.1931	9.07544	10.7422	9.70741
IRF5	7.32457	7.38024	7.92031	7.52055
KLHDC10	10.0993	10.8424	7.90482	9.75135
NDUFA5	48.5146	47.8437	48.0014	52.1688
MRPS12	140.804	119.6	134.009	129.063
MYO1B	24.2962	22.1622	22.7927	24.6331
HOXD3	0.740012	1.1298	1.04771	0.862961
MTX2	60.5842	55.5117	55.9395	58.188
CHN1	3.09028	2.39036	2.62212	3.19715
GAD1	3.82984	4.29306	4.34165	4.09148
EIF2S2P4	10.8777	12.2024	10.3578	10.8913
OSGEPL1	7.1727	6.611	5.58509	6.83593
ORMDL1	26.9489	26.5525	23.8558	26.5796
HAT1	70.1552	57.5745	57.3032	66.9829
HERC2	41.973	41.7991	40.4087	44.9171
PSMG2	34.559	23.5945	26.4155	27.604

TWSG1	20.3819	17.5116	16.5755	18.2992
ARHGAP22	5.27284	4.93347	5.71702	5.40232
EIF2AK4	18.8185	17.7391	18.2055	18.9747
MYO5C	7.30623	8.62466	9.27298	9.21915
TMOD2	1.0373	1.04549	0.951067	0.96609
ELL3	11.7815	8.05763	9.33337	8.57621
C15orf57	13.7771	12.6491	13.3655	14.0502
INO80	29.7786	24.467	27.8511	23.8072
NARG2	29.1382	25.2591	23.3176	27.2179
FAM63B	2.06366	1.86851	1.94933	2.11211
IVD	37.7675	36.2302	34.6314	32.5164
KNSTRN	80.6713	57.4884	63.3873	67.7971
DUT	122.357	116.534	116.404	119.366
CHAC1	5.80653	30.783	12.299	9.46207
CLN6	53.2082	47.8866	50.8886	48.5998
ARPP19	75.0759	66.2541	59.0602	68.1227
VPS13C	3.20386	3.13971	3.42374	3.80134
CALML4	6.81662	6.08924	5.97057	5.40866
THAP10	1.78886	2.11719	1.48622	1.28051
LOXL1	7.10541	7.16806	8.3431	8.4403
ANAPC13	50.1691	43.1076	44.0398	48.684
MBD4	11.5427	10.8107	11.2454	11.4595
COPB1	57.1366	55.1311	53.0692	55.7838
PSMA1	308.422	294.468	294.018	312.868
SUMF2	91.679	109.203	112.616	111.25
PALLD	64.371	57.4835	54.497	58.1128
SPCS3	47.3037	40.1615	39.1438	41.837
SERGEF	17.407	15.846	15.8941	13.7982
E2F8	11.4181	8.30398	8.7269	9.37714

DCTD	66.3643	57.2774	61.9365	65.8915
SOX15	1.13812	1.06748	0.988847	0.977747
FAM64A	41.1141	36.5171	33.1664	34.4293
RPAIN	46.0978	44.005	42.9178	43.0067
PLD2	17.1691	21.586	20.9383	18.3053
CD68	4.48757	3.60287	4.07673	4.6653
TXNDC17	289.905	272.853	263.681	302.465
FXR2	38.4532	36.957	38.2307	36.2008
KIF1C	92.1318	90.1414	95.4121	90.3333
MPDU1	101.6	88.9119	92.8877	92.8864
MRM1	7.74106	6.39726	6.25851	5.99711
PHF20L1	17.1156	13.0364	11.66	14.5127
LRRC6	2.62718	2.39175	1.64753	1.73124
CCNT1	12.3635	8.83037	9.5623	10.7201
PUS7L	4.50757	4.47204	4.16622	4.36891
KRI1	26.4132	24.4484	24.8587	25.2748
ILF3	180.185	182.788	178.054	179.346
SLC44A2	72.8718	82.1465	84.9493	82.9343
AP1M2	57.4939	56.4601	66.6584	71.6726
CDKN2D	10.9972	8.75795	9.36831	8.76222
MTUS1	23.6561	24.9352	25.0381	29.2571
KLK10	9.85943	10.0743	12.6998	10.925
KLK8	68.2698	69.9194	67.0021	67.3826
NGDN	60.0894	46.6498	49.4014	51.3518
RAB2B	7.93449	5.10957	4.64775	5.84017
BCL2L2	16.4249	14.1237	14.8342	16.0649
AJUBA	34.1348	27.3696	28.6968	28.489
DTD2	16.3401	16.2267	16.2056	16.8595
PARP2	53.2028	39.0261	40.8186	44.8319

HEATR5A	10.5839	10.8978	12.4224	12.2143
FOXA1	0.741513	0.899812	1.03122	0.853497
SNX6	39.2491	40.8452	37.3276	44.815
EAPP	13.4553	16.5515	14.6776	15.2152
EGLN3	3.42029	2.67668	3.10496	3.66409
MIS18BP1	11.9347	10.8048	9.87302	11.0185
RNASE1	0.97708	0.99355	1.21304	0.983235
NEDD8	259.351	230.908	244.437	259.517
DAD1	210.205	189.366	183.712	198.795
TEP1	6.8747	8.84992	9.06559	7.81924
EPB41L4A	0.724362	1.28809	1.23312	1.53559
REEP5	116.055	100.645	109.55	108.001
ITFG1	23.0199	21.6104	22.6715	25.1093
QRICH2	2.47654	2.85062	2.75299	2.62583
SEC14L1	19.429	21.6235	22.0529	22.0808
RHBDF2	16.7359	21.5051	22.2223	19.3552
MAP7D3	7.47923	6.93448	6.71069	7.59994
ASH2L	25.307	20.4995	21.5569	23.2454
TTI2	16.2302	15.7396	15.3076	16.7388
CHRNA10	2.44083	1.66982	1.63418	1.47774
CDKN1C	3.92754	5.00315	4.7957	4.805
SGOL1	10.7329	8.2707	7.14759	8.08167
RPS4Y1	49.8321	52.0719	52.6493	52.9686
CDH15	1.06941	1.07257	1.23296	1.01671
KLF16	59.7625	49.1387	59.9522	51.901
TMEM8A	35.1262	30.2248	33.8992	30.0331
DOHH	47.0569	40.6524	47.4511	39.9951
MAU2	15.031	17.9388	17.5318	16.2761
ABHD17A	60.4866	55.3749	66.2311	56.2994

SYT5	1.04326	0.919217	1.13162	0.766392
TNNI3	16.3558	18.5343	22.0386	21.1031
CBFA2T3	6.80703	7.18619	7.88411	8.18051
GAMT	71.1252	78.3501	77.7476	70.662
HDHD1	17.6397	17.9446	19.274	18.7863
C6orf70	17.9352	18.1509	19.9623	18.0035
PHF10	23.4445	19.8587	19.4857	21.5596
EFCAB4B	6.45161	5.76105	6.24645	6.41011
STARD8	2.38771	2.70428	2.32439	2.29159
FAM155B	7.14065	6.78319	6.68302	6.69525
SAT1	70.7128	86.0092	75.2609	75.7671
GNL3L	15.3924	16.2229	17.8604	14.7774
SH3BP4	20.9796	22.2802	23.1675	21.6166
MOSPD2	2.54065	2.43758	1.97259	2.67726
DOCK6	15.9986	21.9907	22.7997	18.5921
ECSIT	31.8839	33.0372	34.5084	31.8718
LDLR	25.3522	21.7131	23.9933	28.2421
ELOF1	89.6427	69.5631	74.6267	80.7992
PRKCSH	185.816	181.142	185.585	179.283
CDC16	34.0308	32.3287	33.0952	33.344
THEM6	12.1404	13.4697	13.6843	12.2725
PVRL2	53.136	52.3519	57.0035	51.8231
TOMM40	232.487	192.519	196.998	199.609
APOC1	1.22716	1.35925	0.698238	1.02611
XPO7	28.7504	26.3226	26.0164	24.3701
FAM98C	26.0017	29.1814	29.7756	27.7467
SAFB2	22.4044	21.9408	22.9059	20.1512
RPL36	2662.56	2492.73	2212.03	2223.74
ATP8B3	5.57479	6.26908	5.21618	5.51495

GTPBP3	52.795	36.5107	41.6437	39.2342
SLC27A1	5.47843	6.4469	7.27601	5.90153
NSUN5	37.7566	35.6431	34.3543	33.7668
COLGALT1	89.6885	86.0263	91.3028	84.8551
DDA1	60.0925	59.6081	59.4369	61.2704
MRPL34	125.482	128.251	131.024	127.227
PGLS	25.963	40.9843	38.4972	31.9372
LSM7	230.417	204.728	183.955	191.34
TULP4	3.36494	2.17815	2.52787	2.66628
SNX9	17.4247	16.5068	17.2621	18.5713
RTN4IP1	7.95756	7.11866	6.99245	7.39547
QRSL1	12.5138	10.7326	11.1913	12.7574
C6orf203	3.30786	4.41487	4.01411	4.3539
RSPH3	5.97581	5.05877	5.50168	5.6703
MLLT1	19.4222	17.7229	17.6962	17.175
MLLT4	11.927	10.5551	11.5725	11.6322
ACTN4	432.76	405.075	327.498	317.361
STK33	5.38656	6.08345	5.56444	6.53188
NDUFA10	127.677	127.262	127.975	133.368
ARPC1B	321.302	307.412	278.09	258.02
ZSWIM6	1.89529	1.51811	2.00799	1.9256
FCHO1	23.1994	22.7214	24.6847	21.1168
UNC13A	10.0734	12.3668	13.0023	11.5437
MAP1S	37.9788	32.8648	37.2476	35.8469
SCO2	50.0065	44.3189	48.8187	45.2535
PXDN	5.33329	5.94476	6.05051	5.02671
SSBP4	89.6838	92.0213	97.9644	94.0748
GDF15	67.503	212.278	173.7	131.76
PGPEP1	5.30032	6.50201	7.40679	7.06083

LSM4	333.46	303.795	275.66	268.522
JUND	74.2397	104.283	104.276	80.961
TRPM4	10.5199	12.1445	13.1348	11.116
SULT4A1	2.72973	2.57627	2.60308	2.56159
ZNF557	3.33208	2.8622	3.38364	3.47587
CRB3	20.7188	22.1852	25.5337	23.8863
OLFM1	1.29669	1.97802	2.55368	1.99367
CAMSAP1	15.7498	13.9667	14.3203	14.7198
UBAC1	36.8186	34.9705	36.1131	36.9882
ZBTB46	3.087	3.00482	3.47573	3.23968
HELZ2	7.50941	8.11285	9.33757	9.34078
SAMD10	1.87113	2.25601	2.98215	2.65204
H19	5.78671	3.65637	6.78786	5.39102
ATXN10	138.666	157.347	163.628	160.531
TUBGCP2	24.9776	27.351	27.5815	24.312
PAK4	42.1298	41.1268	42.3527	40.2091
MNX1	4.5114	3.41786	3.44189	3.27421
ZNF337	4.77337	5.98969	5.65097	5.50967
CEP85	22.5442	21.0907	22.3071	21.3254
TAF4	9.82349	7.48718	7.50367	8.20116
LAMA5	105.568	103.223	118.739	96.2605
OSBPL2	6.0182	6.10578	6.07492	6.36258
ADRM1	233.86	199.643	200.328	201.614
ASS1	27.6631	40.0876	34.1393	34.3974
EXOSC2	66.9679	51.4337	53.0386	54.4012
POMT1	9.36212	10.8769	10.2602	9.74023
UCK1	17.4984	16.2779	17.2675	16.7184
FIBCD1	19.5346	18.6005	17.9701	19.3934
PRRC2B	42.1662	47.2851	46.3311	44.8979

CHMP2A	80.1607	82.6781	90.7231	85.6256
UBE2M	211.774	165.123	171.444	167.486
TRIM28	1479.84	1250.92	1394.18	1243.75
C16orf13	72.3712	68.5098	66.4943	62.095
YIPF2	51.2432	46.9373	49.2153	48.6235
ATG4D	37.1379	30.8242	32.7433	32.9897
EIF2S3	108.065	110.283	103.94	104.535
TMEM160	66.9949	66.389	70.8546	66.4412
ZC3H4	10.6597	10.4012	10.5981	10.72
NPAS1	0.818706	1.75036	1.68341	1.80348
MAP3K10	4.03931	5.7969	5.654	5.46925
ARHGEF16	15.7565	16.1443	20.977	18.5718
LRRC47	42.4397	40.2277	41.4696	41.0136
SESN2	6.62282	11.0307	7.57451	6.65382
SMPDL3B	7.08412	9.38415	8.14095	7.54429
ATPIF1	186.131	187.117	171.88	171.273
MED18	26.9223	23.7832	26.2419	27.5737
THEMIS2	5.31236	4.81404	5.52058	5.60667
CLIP1	11.0789	11.2733	11.7684	10.6931
HIP1R	29.7435	32.2114	32.6453	30.2957
ZNF317	21.1548	18.8801	18.9489	19.4836
PPAN	81.7717	63.5208	74.287	74.837
EIF3G	214.876	198.352	213.636	202.112
ANGPTL6	1.22957	1.18463	1.49899	1.36562
C19orf66	3.33088	4.10915	4.77837	4.06511
DNMT1	106.421	101.929	97.3081	107.568
DKC1	74.364	56.3737	71.1323	68.6425
PLXNA3	6.72208	8.65046	9.94363	8.69196
DUSP9	5.48086	5.06401	5.17954	4.51687

MPP1	3.04175	2.80242	2.98608	3.12237
ZNF236	2.36444	2.53623	2.39621	2.08188
LRP3	38.1819	35.1073	39.8789	35.6723
C12orf65	15.7758	17.5262	16.3542	18.5012
NOL11	116.249	97.5374	94.778	103.609
UBE4B	17.6549	18.9596	18.5766	22.4768
CASZ1	1.18821	0.960907	1.10082	0.908334
HABP4	9.52996	9.82123	9.44515	9.90848
SLC35D2	18.9405	22.1928	22.3837	21.9307
PRRG1	2.64039	2.55064	2.69246	3.06746
UBA1	65.9746	67.7202	69.2254	67.0603
POLN	0.713511	1.22456	0.968198	1.01548
TXLNG2P	1.71271	1.44924	1.47562	1.21619
PPIL4	16.6884	13.141	14.4432	16.9855
ULBP2	9.37789	7.51114	8.2416	7.63101
AKAP12	261.71	261.168	261.072	275.549
SYNE1	0.982016	0.989641	1.34652	1.12386
ULBP3	8.3492	9.19417	8.70532	9.25272
LATS1	4.79595	4.46894	4.46451	4.92563
EPS8L1	11.6612	13.0765	14.748	12.7157
AAR2	26.483	24.2839	25.2758	24.645
RBM39	169.528	145.229	139.184	135.863
ZNF341	1.96963	2.09129	2.31485	1.97573
GGT7	2.72707	3.41237	3.73856	3.12617
ACSS2	19.9136	16.5612	16.8717	13.8077
ARHGEF9	1.24938	0.992403	1.44459	1.42521
C1QL1	1.87511	1.71899	1.9466	1.68468
GFAP	0.663842	1.23721	0.290126	0.449737
ATP6V1E1	85.1685	68.8321	63.0952	73.3365

ZNF227	7.06167	6.88226	6.41321	6.34916
ZNF428	33.8159	36.1464	37.0486	34.134
COX4I1	905.796	873.638	862.713	869.708
EMC8	144.731	123.511	133.656	118.496
GSE1	14.4783	14.6527	16.41	16.5931
RP11-178L8.4	2.5281	3.51025	4.26625	2.98501
GINS2	38.6727	32.9402	30.4992	34.5627
CHMP1A	130.098	113.919	125.895	119.09
SH3BGRL	12.7375	15.6786	14.2287	16.039
COX7B	142.5	132.028	126.875	132.66
F12	32.8274	28.7392	32.8032	29.9535
PRR7	24.7786	25.6105	30.9223	25.6864
NFATC1	6.52065	6.33301	6.59926	6.73159
CAP1	204.45	168.42	166.446	178.774
PPT1	64.6339	62.8621	58.2198	56.7825
RAB11FIP4	5.7941	4.26814	6.06544	5.39219
RLIM	7.50792	7.12174	7.14366	7.63927
ABCB7	10.743	10.9986	11.0776	11.6679
TRAF3	16.1921	13.265	15.6897	16.5248
HAUS8	23.9276	21.666	21.4867	24.4774
MRPS25	46.4884	44.6406	45.1583	43.9274
SH3BP5	13.1313	12.7999	13.0577	13.1395
HACL1	9.44575	7.85435	7.4462	8.46884
TBC1D5	9.54261	9.48191	9.01141	10.5684
CAPN7	12.1129	11.7259	10.5088	13.6237
RFTN1	3.30943	2.36555	2.82229	2.41147
ZFYVE20	5.94066	5.56657	5.4252	5.96041
SLC6A6	14.0253	14.2319	13.5908	16.1009
KCNC3	1.50702	1.86797	1.81158	1.66901

NAPSA	0.72099	0.959595	1.03414	0.910168
NR1H2	19.1984	24.1563	25.9309	21.5334
KIF3A	4.41318	4.57829	4.62562	4.31465
MGAT1	52.2732	46.5663	42.4498	42.0632
GFPT2	4.8989	3.22898	2.91271	3.23104
TUBG1	131.797	111.235	120.266	119.159
PSME3	166.032	126.604	129.382	139.31
RPL27	1761.59	1712.17	1528.79	1785.09
PSMC3IP	31.0185	23.0978	23.4642	25.5044
AOC3	2.17481	1.25794	1.47656	1.37786
ACLY	66.0241	59.692	57.8097	54.8155
VPS25	69.478	71.4621	70.6925	69.8979
AOC2	2.33304	2.23033	2.02589	1.99626
RP11-798G7.5	1.41163	2.05076	1.93088	2.01621
NDUFA2	72.5402	77.4353	73.1868	71.249
ANKHD1	29.1858	29.8592	32.2488	29.5448
DIAPH1	101.87	93.3783	96.5246	90.0637
NDFIP1	30.2436	27.0859	35.071	29.0477
UBE2D2	74.0318	58.3032	57.0692	66.5269
EXOC4	10.9912	10.6909	10.0484	9.66004
ACAP3	18.4249	19.1875	24.1707	18.2919
C1orf159	14.6171	13.9886	14.9114	12.5349
ANO1	36.0374	32.8432	35.4332	35.1366
PPFIA1	24.4052	20.6246	20.4826	20.1041
KREMEN2	8.62051	9.85945	11.3053	8.67803
THOC6	56.0361	49.8606	48.9676	51.9345
TRAF7	130.325	132.307	141.689	135.991
NINJ1	32.2167	40.094	37.2325	33.6374
NPHP4	4.77321	4.97553	5.26679	4.51059

MAP1B	0.95657	1.65575	1.28331	1.3059
IL13RA1	10.2617	10.8147	10.3258	11.4024
WDR44	5.09541	3.67552	4.58687	5.15954
ZCCHC9	22.9271	21.9322	21.4626	24.6216
TNS4	22.0671	20.8881	24.6609	24.1655
TOP2A	31.9787	29.9352	27.5198	30.3842
STARD3	47.8159	45.6637	52.8886	46.4888
RARA	18.4146	16.6658	18.0998	16.8782
KHDRBS3	28.7745	23.3118	27.3117	32.0323
CHD1L	15.6955	15.5785	16.0243	14.7419
PEX11B	8.18439	8.76805	9.62807	8.96007
PIAS3	7.41685	4.66272	6.44007	6.06579
PRKAB2	2.94756	4.90729	3.75569	3.70508
RBM8A	79.6665	70.9227	67.6294	70.913
CLUHP3	4.25407	4.5881	4.4566	4.33258
PDHA1	233.442	222.6	232.535	228.259
MCCC2	20.3185	23.8537	22.7063	24.4228
ZNF304	2.8374	2.82992	2.76068	2.75483
ZSCAN5A	9.78048	6.761	7.00733	6.85324
VIMP	53.5668	44.1333	41.7258	43.6386
CHSY1	15.8082	13.9997	14.723	16.3352
SNRPA1	88.382	71.6104	67.6272	75.3704
LLGL1	20.2215	20.0838	22.3081	20.768
THAP1	10.0613	9.22965	8.16566	8.69852
RHPN2	20.3329	7.98247	8.68487	10.1536
C19orf12	9.0363	11.4757	12.2971	12.5616
C19orf40	3.19275	3.40766	2.97208	2.88271
ACTR10	37.5226	40.7841	39.1627	45.3931
ABHD12B	2.75916	1.86819	3.29989	3.61863

GCH1	10.1252	9.24285	9.94898	10.1177
LGALS3	68.7689	84.585	89.1734	84.5328
DNAJB1	151.061	102.511	102.866	114.965
ZSWIM4	0.590344	1.49614	1.59789	1.20458
FBXW9	10.5206	11.5186	11.7355	10.8065
RFX1	5.34239	7.15252	8.18151	9.45043
DCAF15	48.0509	43.5369	46.4913	41.6973
CC2D1A	17.238	18.577	20.9953	18.0928
RTBDN	5.60333	6.68479	6.99	7.46511
TRIM21	7.27293	6.81849	7.76545	8.31479
SPATA6	1.40072	1.43596	1.49321	1.51489
LRRC41	22.2784	22.8517	24.4092	23.651
ACACA	39.5541	32.1688	32.715	32.7767
DHX30	80.7009	70.8915	74.9468	73.6151
RAF1	42.2661	43.7648	45.3897	45.8805
PPARG	20.4852	20.9305	23.6196	23.5133
NUP210	25.9483	31.479	31.7445	28.9639
HSD17B7	9.39075	8.84496	9.0623	8.76792
ENOSF1	34.3938	38.7865	32.8457	40.1657
LINC00470	1.68566	2.04605	1.67454	2.90647
EMILIN2	18.0609	18.72	20.175	18.607
SLX1A	23.2589	28.554	30.3678	28.5762
ARFIP2	58.4149	52.8528	58.4746	56.6298
TRIM5	6.13374	5.89074	5.86511	6.47309
TRIM22	0.604308	1.63903	1.66804	1.4152
RRP8	11.2757	10.6187	10.8561	12.6059
TIMM10B	26.1448	25.0445	24.8009	25.888
EFR3A	26.1772	31.1643	31.8585	32.5176
HHLA1	3.6756	2.99417	4.01047	4.73757

PTCD3	72.0237	67.0245	63.4837	66.6561
IMMT	59.9429	57.6521	57.5538	60.475
MRPL35	63.0078	49.6669	46.9245	54.5214
ILKAP	38.568	33.1797	34.2215	35.1725
PER2	3.00415	3.30546	3.08605	3.10526
RAMP1	30.804	28.0612	33.512	30.7835
SCLY	26.4531	23.4653	22.6449	20.8925
PTPRE	4.51754	3.96647	3.89436	3.70011
RAN	1085.53	941.791	966.482	998.838
PRKAA1	19.88	17.1984	16.8487	20.1974
RAP1GAP2	21.3153	23.0807	23.496	22.1704
CLUH	141.528	133.941	141.037	136.288
INPP5K	15.996	14.1265	16.0916	15.9335
MYBBP1A	66.0925	62.5613	60.7439	61.1193
RPA1	141.205	116.179	117.49	112.032
SERPINF1	1.26506	1.22151	1.09081	1.23669
UBE2G1	44.5529	38.0507	38.7109	40.9063
EEFSEC	10.9367	10.881	12.1328	11.1704
TBC1D14	39.9639	37.1207	36.1852	39.1431
TMEM128	18.8432	20.021	18.1244	19.1569
COQ3	25.5298	22.6674	21.5598	25.0518
PNISR	24.8524	23.002	22.8218	24.1047
POPDC3	11.2638	10.4705	8.77497	10.3587
SEC61G	190.039	177.324	173.995	196.348
LANCL2	14.3414	12.8769	13.2079	13.3869
FIGNL1	13.6749	13.861	13.0838	14.3779
GRSF1	143.774	126.725	126.497	134.801
ANKRD17	25.9286	25.946	27.2111	27.9334
UTP3	31.9222	25.4325	27.681	30.9535

ITGB4	97.3792	133.416	149.087	128.18
WBP2	68.7649	77.207	79.1715	75.2974
H3F3B	1431.37	1263.12	1204.21	1297.71
UNK	21.0551	18.3544	21.5938	22.5582
TRIM47	54.2619	53.4418	58.0516	55.9195
ZRANB2	32.8279	27.8598	26.1046	30.7354
EIF5A	1414.32	1274.81	1234.86	1264.77
KDM6B	6.21672	9.28871	7.50357	9.84522
GPS2	169.732	145.927	152.116	150.294
DLG4	4.56156	4.39229	5.055	4.47134
HRSP12	34.3777	35.5031	32.124	34.3402
VPS13B	4.0247	4.5397	4.33711	4.39283
MATN2	0.924398	1.66989	1.27348	1.23179
REEP2	3.05633	3.17989	3.0899	3.46448
PCBD2	6.04185	7.92036	6.03875	6.47432
SDF2	41.6977	43.2402	47.8633	44.5351
FLOT2	36.5333	41.8606	39.9825	35.1771
ERAL1	60.8822	54.9455	60.7106	56.1614
PRMT7	36.5109	31.3899	31.6295	34.2744
NIP7	90.4173	64.9564	66.4683	78.2669
TERF2	51.1121	48.8579	49.8832	49.2754
VPS4A	78.099	70.8693	74.3991	75.9548
MTSS1L	31.7589	28.8489	30.6683	29.5264
ANKEF1	7.23224	7.41035	6.73982	7.90248
PCED1A	9.84266	12.5427	14.6242	11.5745
BTBD3	9.11076	10.2272	9.20134	9.58829
PCNA	302.508	248.149	223.452	253.737
NXT1	35.7632	27.5736	29.3807	29.2752
POLR3F	15.29	12.6796	15.2557	14.5011

PTPRA	22.2108	21.8089	22.7445	22.9329
DAP3	147.7	127.433	128.29	138.22
RHBG	2.64682	2.22315	1.86277	1.83878
KIAA0907	21.3866	18.8599	18.336	18.8862
NES	3.8654	5.20695	5.36035	5.58374
ARHGEF11	10.0912	9.48496	11.2302	10.2947
RAB25	73.9115	68.9525	86.7263	81.0365
DCAF8	41.3071	38.0177	50.5617	46.0334
IGHMBP2	11.3211	10.1283	11.1843	9.74591
MTL5	8.05012	10.594	10.3005	9.5045
MMACHC	7.55592	6.56439	7.26441	7.73483
DPH2	66.7384	61.1345	65.4037	61.3346
TOE1	23.7237	18.0658	18.9938	19.116
NASP	171.232	139.965	145.146	155.745
MUTYH	23.436	27.6576	26.8029	24.794
CTNNBL1	44.9305	40.5956	40.609	42.4898
LPIN3	4.80217	5.00197	6.07334	4.8008
RBM38	42.1374	43.2393	45.6585	38.7988
OSER1	18.9862	19.8585	18.9223	18.9754
SERINC3	31.2157	32.2232	35.0798	32.1624
PPP1R3D	1.17088	1.28492	1.32537	1.29576
AP3B1	24.8456	25.1781	24.7277	24.0941
ZBED3	2.79912	3.97463	2.94004	2.86823
INADL	7.07961	5.62299	6.42158	7.36853
FBXO44	23.8478	24.405	27.7082	23.587
RSG1	4.47072	4.83429	4.51018	4.66459
CASP9	5.87092	5.17494	6.08613	5.85497
DCTN4	26.0397	27.9215	27.4545	29.2907
ZMYM5	3.52483	3.69907	3.04376	3.47961

USPL1	9.01041	8.61531	8.57182	9.45503
XPO4	11.8784	10.5611	9.81525	11.6965
POMP	115.25	105.263	93.0545	117.122
CDK8	21.2576	18.8108	18.8535	20.3046
HMGB1P5	189.36	145.47	140.996	173.121
WASF3	1.14741	1.17725	1.43826	1.43699
MYH10	13.7955	15.6637	14.3087	14.2402
PEMT	55.4362	53.1306	59.9799	48.1563
SCO1	36.8461	34.4745	37.5392	39.5798
MPRIP	140.687	123.944	139.601	140.709
PIK3C2B	3.36844	3.67948	5.02814	4.24119
DSTYK	2.43882	2.16662	2.33957	2.36048
SLC41A1	15.6043	14.5175	13.4147	14.6468
TMCC2	1.64956	1.22543	1.65242	1.33017
COG6	7.71183	6.81561	6.4396	6.45461
RFXAP	1.86818	1.48808	1.62331	1.76879
TPT1	1553.74	1635.32	1601.75	1661.87
KIAA1704	9.38166	8.51054	7.13221	8.56768
RFC3	31.6061	24.8472	25.767	29.1628
STARD13	1.31722	0.97766	0.912908	0.968345
MORC4	11.6282	13.5083	12.1686	12.5875
BEX2	24.4231	31.8795	28.9957	26.0643
RNF128	3.60043	3.32208	3.21539	3.33919
TBC1D8B	2.82141	3.00883	3.00263	3.27765
TCEAL4	16.9617	16.2238	15.0645	16.9344
FAM104A	45.4617	39.3749	40.8418	44.0078
SLC39A11	15.6713	16.4942	17.1945	18.2884
EPHB2	4.83593	6.15521	7.16538	6.78991
SRRM1	56.2118	41.3101	44.249	50.4182

BTBD2	41.4593	53.408	59.5682	50.1907
SUV420H2	6.27563	5.91319	6.54112	5.27354
ZNF414	12.2236	11.7058	15.146	13.2573
HSPBP1	67.8239	65.0409	70.4755	66.3909
CSNK1G2	84.8766	72.9123	82.2291	77.2036
ANKRD32	8.45701	6.17986	6.02116	7.1179
CNDP2	61.4136	60.9749	67.2251	65.5767
MACROD1	40.4422	50.9827	47.6634	47.0815
WDR74	112.575	93.5619	97.03	95.2806
RTN3	50.3025	50.435	47.209	50.1402
FOPNL	51.3502	46.2483	42.0018	49.4029
MED10	32.2802	26.4788	28.8284	29.3593
MORC2	19.5097	16.6164	17.4434	17.1287
GSTT2B	8.7962	9.12512	8.76734	9.07062
SLC2A11	3.75848	5.04514	5.12439	4.76231
C1QTNF6	2.83806	4.19183	4.46897	3.85563
FAM83F	14.5692	14.4759	15.1373	13.526
SEC14L4	4.51406	5.844	5.89636	5.64235
ZDHHC8P1	0.485315	0.601138	0.657625	1.05062
ADCK2	9.97007	7.87237	8.44634	8.45574
MKRN1	36.667	40.3641	42.8411	41.3638
AGAP3	67.1637	64.3683	70.5148	66.9622
ZNF767	3.00129	2.81328	2.51327	2.37069
ACTR3B	12.1707	11.191	12.5659	13.536
BTG1	8.24506	11.3787	8.46557	6.8772
C12orf29	27.839	22.7589	21.3444	22.8191
ATP13A3	77.9897	52.5099	57.258	65.3616
TMEM254	10.1911	9.84307	9.80631	11.3272
KRAS	12.083	9.22377	10.4851	11.8172

IPO8	17.9903	13.9348	16.0657	16.9066
LARS	63.9906	70.6437	64.5614	63.0265
IMPA1	31.952	27.0892	29.2662	34.4103
LRRCC1	4.21504	3.85874	3.92313	4.86816
E2F5	6.56974	8.80834	7.7349	7.7836
CCDC59	49.5598	44.4815	41.053	47.3044
SWAP70	9.22359	8.50068	8.21585	8.83373
ARNTL	6.85999	6.57134	7.35129	7.57115
AMPD3	11.8967	12.7702	14.149	12.4135
MICALCL	1.59658	1.21455	1.24059	1.16151
SBF2	11.4186	15.154	16.4889	16.9577
MICAL2	11.2365	10.1275	11.2062	11.2655
RRAS2	23.5526	22.9238	20.8693	23.4361
HSD17B4	163.338	177.412	181.63	177.048
ZFC3H1	11.9265	12.0103	10.1386	11.0552
TMEM66	58.9045	63.595	62.9959	65.0701
RNF122	2.2083	2.28885	2.50273	2.18635
DPF2	48.5651	48.0935	45.8982	50.2988
MEN1	42.2684	41.1874	41.4425	42.1885
C14orf1	12.4601	12.1035	12.5583	12.7743
C14orf159	14.3983	18.6972	18.8586	18.464
NUMB	14.0341	14.8805	15.3653	15.2475
COX16	49.8886	46.2669	43.1653	49.6298
MED6	40.1789	36.405	35.2049	40.4384
EIF2S1	285.258	218.625	210.756	237.432
LOXL2	5.53034	6.92481	7.19693	5.76749
ELP3	28.5886	31.6869	31.2552	31.6112
CTIF	3.80741	4.45983	5.33144	4.04385
MBD2	142.796	96.3738	93.6773	100.078

IER3IP1	50.5109	44.4098	40.4205	45.7645
MRPS36	53.7171	52.8337	59.0285	65.854
CCNB1	359.761	271.372	262.111	288.331
CDK7	41.1727	34.7583	32.4881	34.4213
IRAK2	1.20637	0.855946	0.902732	0.917394
CAMK1	20.5527	18.2818	19.9691	20.1866
THUMPD3	64.2527	53.3072	58.7849	61.4767
VHL	27.4365	27.2485	28.8988	28.7004
BHLHE40	7.02868	6.17462	5.42593	5.9739
ARL8B	39.3312	30.4296	24.1639	27.8166
EDEM1	4.68075	3.73728	3.23995	3.91433
MEIS2	6.74895	5.87157	5.57611	6.07401
DPH6	16.175	16.3957	16.6696	17.2555
KATNBL1	19.6288	20.4967	20.5115	20.5115
EMC7	42.0465	41.3385	40.5083	43.3546
PRPF38B	21.8498	18.0899	18.456	19.2201
PSRC1	29.2689	29.0866	26.9327	27.182
SORT1	5.70585	4.75249	4.42006	5.28249
PTGFRN	25.1673	26.3782	25.5545	29.554
LAMTOR5	165.65	168.741	165.783	179.204
NOTCH2	12.3586	12.5517	12.3733	12.8624
TRIM45	1.11501	1.13106	1.10176	1.05251
CEPT1	24.8752	20.1876	19.2234	20.8453
CD101	1.26587	0.812847	0.889676	1.00837
AP4B1	9.12148	9.55369	10.6638	8.92109
NAPG	14.1889	13.2231	14.535	15.7659
SPIRE1	6.99981	5.73931	6.13423	6.89589
PPHLN1	38.2446	36.6528	34.4482	38.7553
FKBP11	6.21176	4.67432	6.12689	6.20468

ARF3	83.6743	91.71	97.6177	99.5618
TMEM106C	147.328	91.734	93.6445	94.7198
SLC38A2	167.512	168.915	155.783	175.995
PLEKHA8P1	3.90069	4.20768	3.83083	4.29695
YWHAQ	312.408	300.32	290.74	308.189
KIDINS220	6.53386	6.30684	6.25405	6.92694
GRHL1	3.10812	2.69772	2.75501	3.40607
ROCK2	13.9056	11.388	10.4848	11.8805
LPIN1	2.47502	2.01208	2.89085	2.89523
IAH1	21.1308	25.6304	25.3903	21.9924
LDHA	992.572	738.888	694.749	804.481
IL6ST	8.41435	6.06537	4.88273	6.09298
NAV1	4.61589	3.92938	4.58018	4.40735
CDC73	12.3271	10.4828	10.4245	11.7059
TIMM17A	133.496	102.665	106.729	118.123
RPS15A	1810.68	1857.44	1676.64	1778.02
NARS	113.534	128.279	116.111	124.309
KIAA1468	9.53367	8.37507	10.7655	10.5998
FBXO18	22.4553	24.1778	24.4709	23.0431
RBM17	111.035	87.4403	88.3963	91.8998
ANKRD16	5.762	4.90143	5.2181	4.43851
ECHDC3	6.31361	7.72375	8.15518	8.26589
IL15RA	6.24214	6.71547	7.33769	6.91226
CCNH	52.7284	51.8358	56.2156	54.4988
TMEM241	13.2504	10.57	11.5019	11.9957
KCTD1	5.99121	7.29722	8.23016	7.44048
CABLES1	4.48558	5.02843	4.22562	4.32207
EMP1	56.1085	30.1301	45.4381	46.2493
LRP4	3.99333	3.2005	3.29181	3.74279

DDB2	17.9593	25.2064	20.5364	20.2372
ACP2	16.7264	16.9475	20.4411	17.4448
FAM127A	35.3499	40.3692	48.2804	45.683
RBMX2	16.4602	13.5426	13.8754	13.723
MST4	16.2195	13.9865	13.8389	15.3857
PUM1	24.2864	25.0781	23.8631	23.4336
YARS	112.851	119.227	104.744	109.37
PHC2	68.6935	65.4199	65.6466	64.0537
CDCA8	52.4253	42.084	43.8442	46.3457
GNL2	89.1774	61.7512	65.5142	73.2391
AGO4	2.47378	3.28895	3.02794	2.04037
HOOK1	7.56607	9.11176	9.66104	9.76078
BTF3L4	44.9273	48.2641	38.3096	47.2797
ZCCHC11	19.3764	18.871	19.2757	21.774
PRPF38A	46.0903	45.7382	44.3656	45.6227
DSC2	5.93234	6.58738	6.82542	7.26427
RNF138	52.6365	40.4725	34.4254	39.8128
ELP2	58.4856	62.0881	56.0918	57.0108
DSC3	2.12669	2.87043	2.33678	2.91987
DTNA	2.3896	2.77897	2.92766	2.69938
FHOD3	1.74657	1.66584	1.7007	1.60192
TPGS2	69.5562	72.1945	78.8385	80.4082
DAGLA	1.60336	1.66884	1.91692	1.67583
SLC43A3	26.6497	22.1273	22.7628	23.7261
TIMM10	112.648	91.5624	95.9635	93.6648
DHX34	23.3347	20.7768	22.3485	19.3363
FADS2	6.80692	7.70463	6.35214	6.38261
TMEM258	210.234	191.875	172.062	188.393
TMEM165	44.1949	48.0671	49.1597	51.0275

CLOCK	12.3792	8.46305	9.83816	9.87282
GGACT	2.3457	3.26622	3.63051	3.56159
UBAC2	44.6523	46.0999	46.9808	46.7235
ARGLU1	45.7395	43.6401	44.3552	44.2986
BIVM	6.00805	5.49637	5.92052	5.65765
ERCC5	5.08538	5.34995	4.96321	5.02614
TPP2	14.2389	13.5216	12.7633	14.5725
KDELC1	4.19806	3.86347	3.23398	3.84729
CARS2	40.9408	34.8382	37.7621	37.8809
ARHGAP32	4.16367	3.64381	4.08609	4.24567
STT3A	195.779	140.237	150.615	155.91
ETS1	3.37269	2.68444	3.26878	4.35626
SLC37A2	2.0491	2.62656	3.19598	2.82003
TMED7	75.573	64.4706	79.7847	75.4187
APC	4.78606	2.71309	3.10248	3.13645
NREP	2.23989	2.52719	2.50314	2.34153
WDR36	19.3109	15.9655	15.8305	17.9501
OSTF1	12.4998	10.1473	11.6124	13.6828
RFK	38.2846	34.3512	37.8005	38.5851
UBQLN1	144.867	106.081	122.632	126.206
NAA35	18.4213	18.7342	19.0734	19.7397
C9orf40	8.87946	7.67213	8.86438	9.28458
ANXA1	251.771	275.949	279.621	278.05
CTSL	56.6175	49.6471	47.1004	51.8947
TMEM2	10.0057	12.8374	12.5463	13.9687
AGTPBP1	4.04493	4.63037	4.73591	5.13569
GOLM1	58.2085	50.5202	51.1276	51.4583
PSAT1	74.7161	89.6015	45.2061	53.7334
ISCA1	26.628	22.612	19.6922	21.9768

ADAM19	17.8927	22.7046	24.2764	22.597
CCNJL	2.81756	2.55197	2.57671	2.52519
TAOK3	4.58039	4.66742	4.84847	6.05206
USP30	7.13071	5.83873	6.31588	5.30685
MSI1	9.5226	6.90532	8.25613	8.60726
FBXO21	26.5898	19.3407	23.123	20.2288
TBX3	2.41054	2.81183	2.93749	3.0874
OASL	0.837306	0.878108	0.907395	1.12957
RNFT2	6.63543	8.21673	7.70481	7.27089
P2RX4	5.76674	7.69564	10.0248	7.92884
CCDC64	10.3464	11.8097	15.278	12.2145
TRAFD1	14.0895	13.1048	14.5734	15.5726
DMTF1	23.8178	25.9784	20.6616	23.0398
TMEM243	17.2345	12.3093	13.0337	13.074
CCDC146	1.00034	1.34406	1.40286	1.50728
TMEM60	9.51027	9.06168	9.6536	11.9441
POM121C	19.7049	16.5974	17.275	16.5501
PNPLA8	4.98113	5.19496	4.55503	4.74144
HILPDA	16.3918	13.2586	11.6125	11.9717
RINT1	14.9815	13.484	13.7404	15.8528
SRPK2	17.1456	16.9816	16.1344	17.6067
TES	66.0025	77.7202	72.9275	72.9088
MDFIC	4.55775	3.08567	2.86341	3.97525
MTO1	26.5474	27.9089	27.1233	22.7793
ANKRD6	3.88779	4.05958	3.47045	4.39831
KHDC1	8.60345	7.85916	8.04059	7.67774
KIAA1009	1.28015	1.02172	0.871302	1.12487
SYNCRIP	164.747	134.698	127.744	148.489
SNX14	42.8135	43.3011	40.9775	45.8402

NT5E	31.3751	30.4491	31.1064	31.6558
MRAP2	1.23295	1.3183	1.14422	1.26663
AKIRIN2	23.8616	25.0709	25.5946	24.7614
ORC3	26.0277	26.5289	25.5545	28.744
MAP3K7	14.3084	15.8477	15.3136	17.0719
LMO2	1.11888	1.14896	1.13692	1.09511
PHF21A	4.40794	5.46006	5.00993	5.4149
NAT10	51.8519	48.0092	48.595	51.4106
EHF	12.1038	10.9249	11.1081	12.0431
PRRG4	2.14151	1.47128	1.6647	1.84597
CAPRIN1	189.277	172.466	167.426	169.459
ATP5G2	391.907	385.271	398.259	378.24
DNAJC14	26.6519	19.9147	26.018	28.2939
CD63	281.049	243.534	236.559	265.949
PRPH	1.62704	1.94002	1.68824	1.69853
GDF11	3.94723	3.86599	3.70726	3.5278
ITGA7	3.96394	3.99954	4.11367	3.96256
RDH5	1.6252	1.74019	1.05614	1.5541
AGAP2	5.38092	6.63955	7.46157	6.72012
BLOC1S1	80.5401	83.3424	80.3886	75.596
CDK4	381.635	334.6	354.651	352.369
TROAP	68.4115	54.4705	46.8954	51.405
TSPAN31	10.6835	11.0266	11.3388	11.1263
B4GALNT1	18.501	20.5112	19.1772	17.7226
TFCP2	24.178	22.8799	23.1821	25.6606
COQ10A	5.04121	4.69675	4.99533	5.25848
PAN2	14.6181	20.0001	18.8917	17.713
ESPL1	21.5729	18.747	17.4288	16.2131
KRT7	38.4166	38.4401	38.9941	38.074

ZC3H10	4.22366	3.59607	3.9502	3.80661
HNRNPA1	1320.37	1102.17	1020.16	1129.03
ACVR1B	11.845	12.7036	10.4451	10.1531
OS9	46.0669	50.1346	50.7215	48.4656
KCNH3	16.532	17.6589	18.5808	15.3526
LTV1	51.5241	42.3429	43.7095	48.3374
MAP7	10.1881	8.8092	8.76837	9.03462
CD164	86.9939	74.9342	79.5808	84.4299
LACE1	1.87878	1.95	1.3272	1.68994
NHSL1	3.1419	3.5074	3.30224	3.26574
AHI1	9.49323	8.90324	6.9319	7.95862
PKIB	6.81243	6.48507	6.92737	7.95834
SMPD2	13.2722	11.2655	12.1191	11.8143
MICAL1	8.27967	9.35246	9.70533	9.63342
REPS1	33.3958	23.8574	25.5716	32.2879
TEC	2.07864	2.04013	1.81126	1.73585
PRADC1	12.7135	8.87246	11.1862	11.2819
SEMA4F	7.30406	5.35222	7.02149	6.42084
CCT7	616.724	572.248	563.963	638.218
RAB11FIP5	14.2468	14.1994	14.1532	14.6628
SMYD5	36.372	36.289	35.2979	37.629
CCDC142	4.42945	5.76659	5.52127	4.97903
EMX1	0.783916	1.842	2.11539	1.62562
KCNMB4	1.91747	1.7781	1.94349	2.62477
USP15	38.3258	27.7588	28.4198	29.7738
GNS	20.4009	21.4363	22.8551	23.024
CPM	3.88138	4.14302	3.18784	3.37604
MDM2	76.6567	67.8422	56.8844	65.0174
KLHL36	22.8604	22.4894	25.0777	24.4651

MPHOSPH6	48.4093	43.2293	42.5618	49.9335
KIAA0513	3.24816	2.54148	3.15457	2.92026
DYNC1LI2	37.2043	40.1919	35.5509	37.9709
FBXL8	4.04189	5.85903	6.13906	4.99185
FHOD1	35.4739	31.239	37.8455	31.9593
CCDC102A	1.77023	1.97521	2.21442	1.92319
SLC9A5	4.51514	3.62721	3.61236	3.65783
ZNF670	4.49998	4.06194	3.90807	4.04995
PCNXL2	5.2705	5.08855	5.73575	6.30001
KCNK1	17.7144	14.1865	16.0386	16.8832
URB2	14.9463	12.2173	13.4219	14.4049
EGLN1	15.9918	15.1765	16.6452	18.5738
COG2	14.6344	15.3902	15.0189	16.544
ABCB10	10.5572	11.6477	10.9381	12.4223
NTPCR	23.2139	28.3312	27.4122	27.1879
TAF5L	18.3346	11.7838	10.7716	11.5428
GLUL	96.4257	92.8549	90.5875	93.8488
STX6	12.8616	13.1856	12.5937	12.5656
RNASEL	1.05226	1.27047	1.01371	1.16481
DHX9	196.933	164.883	170.397	185.767
CEP350	4.55142	4.13682	4.66778	5.72097
FAM129A	0.60038	1.44218	0.826745	0.880909
PIGC	11.3869	12.3795	11.9768	11.2993
ACBD6	34.2455	28.6	29.7778	31.5661
LAMC1	28.7691	28.1778	27.6861	28.3344
RC3H1	2.98274	4.07122	2.97689	3.76377
SP110	4.08952	3.74742	4.58346	4.4883
MRPL44	35.9772	31.1597	31.0162	34.5116
TTLL4	32.3816	26.3041	30.8791	27.8146

USP37	5.07085	4.30899	4.06824	5.00469
ITM2C	78.4716	81.8612	91.2399	82.7834
SERPINE2	51.213	32.2266	44.8482	43.1003
DNAJB2	21.0187	17.2932	21.0013	18.9136
TMBIM1	63.7154	59.1744	59.1629	59.2809
EIF4E2	65.1323	60.7921	60.916	68.5932
ARMC9	9.40826	8.49838	9.2624	7.66532
CAB39	26.8882	23.5433	23.8861	26.749
COX5B	258.75	264.135	259.101	257.837
REV1	15.294	13.7578	13.6514	15.2434
TSGA10	4.06207	2.56716	2.58377	4.40748
MFSD9	9.00294	8.47152	7.26208	7.74577
TMEM127	17.2517	18.1383	20.263	18.7411
TGFBRAP1	9.63958	8.50089	8.33875	8.22187
GCC2	3.37768	4.48082	4.14936	4.37556
MRPS9	35.593	34.8114	33.9454	34.6347
C2orf49	9.27354	9.1445	8.76669	9.4383
ANKRD36	1.91907	1.94769	1.89028	2.20096
EPC2	7.25615	6.82656	7.12402	7.60012
ARHGEF4	11.1715	10.7803	11.6578	10.3684
ISCU	78.8675	57.5845	47.4881	46.6391
SCYL2	13.7422	14.9139	14.5541	13.8602
CKAP4	32.3091	28.169	28.9919	30.1848
APPL2	8.62786	11.7483	6.35929	7.26253
PWP1	69.3557	53.8625	55.393	55.7102
DRAM1	9.27095	10.9516	9.77538	9.1396
KIAA1033	11.8683	9.27245	8.24209	11.4345
SLC41A2	3.2548	3.69338	3.27948	5.0487
FLNB	43.3241	48.9891	46.416	41.5567

NEK3	10.5451	9.11666	9.50778	10.3969
VPS36	21.8661	23.5276	22.5565	24.6527
RNASEH2B	23.571	22.4135	20.8377	23.6731
CKAP2	25.2162	22.4183	20.857	23.3618
TBC1D4	13.312	10.7725	11.7145	11.725
BORA	23.656	16.9465	15.8269	18.5001
LRCH1	5.22109	4.43229	5.20144	5.60268
SUCLA2	31.2655	27.3072	26.0976	30.3299
RCBTB1	10.9607	9.49559	9.92192	10.3062
MED4	36.0512	32.1991	31.9974	33.5262
PHF11	3.96848	4.64805	3.66616	3.71699
RPL13AP25	2.87718	2.74042	1.29457	1.08997
COG3	15.1696	13.2052	14.0115	16.246
LMO7	3.67778	3.61609	3.34983	3.87318
ITM2B	79.2931	81.137	79.2027	79.955
SPRY2	3.05291	2.61532	2.853	3.18697
NUDT15	32.1441	28.8701	26.2487	30.6473
SETDB2	1.256	1.14857	1.27712	1.64615
SCRN1	36.1047	30.7797	33.6709	35.0355
C7orf25	1.37961	0.959584	1.20777	1.62633
TNS3	2.51004	3.32342	3.1677	2.93722
CHST12	9.66003	10.1497	11.5757	10.1219
IGF2BP3	26.5483	25.3996	19.3235	23.1359
GPNMB	1.19761	1.52273	1.21293	1.12961
RAPGEF5	2.9667	2.81448	2.77428	3.22217
RAC1	233.281	196.542	193.596	209.623
KDELR2	115.125	113.446	100.47	112.197
NUPL2	37.5406	30.0658	31.1829	37.4125
ZDHHC4	42.265	42.2763	41.8218	42.1938

BZW2	112.256	84.2121	105.618	109.483
TBRG4	101.727	86.5647	94.4574	94.9411
DDX56	155.698	134.815	144.71	133.047
HUS1	12.3671	7.57882	8.17914	9.84595
DBNL	43.1441	32.8557	39.441	37.7593
CCM2	24.015	26.2805	28.3127	24.8407
TTYH3	16.5346	21.0404	22.2846	18.6528
CIDEB	13.1723	11.6218	13.7148	14.1855
TTC5	14.4454	10.394	11.8131	12.4018
NKX2-8	5.00494	5.89122	6.15497	5.45131
MTHFS	27.7259	25.6143	25.4722	27.2447
ADAMTS7	1.81089	3.36616	4.22558	2.93407
ABHD17C	24.1568	19.7056	19.1279	19.9482
IREB2	15.69	18.3463	16.9888	20.3109
CIB2	15.9782	15.9136	17.4086	17.7438
CALCOCO2	47.3071	62.0128	51.8568	54.7523
RSAD1	41.7378	44.9765	47.9737	45.1334
NMT1	163.49	140.822	132.707	138.596
SRSF1	311.633	212.46	207.384	230.872
VEZF1	31.0487	27.4963	27.0602	29.3749
TACO1	41.8736	34.0443	35.5961	35.355
TEX2	11.452	10.775	12.0402	11.0763
DCAF7	55.9993	53.2373	55.9533	55.5786
LIMD2	4.47322	6.39184	6.53466	4.96859
BRIP1	4.84284	4.20088	4.39083	4.77208
KAT7	18.5188	16.3555	17.3676	18.5287
ACTL6A	109.678	76.5958	65.7475	96.5242
NDUFB5	141.562	161.245	152.516	166.088
MRPL47	73.5354	62.2906	58.7263	68.072

TRA2B	264.343	191.684	198.69	210.035
7-Mar	30.6373	29.352	32.7214	31.8924
GALNT5	5.17478	4.51389	5.6539	6.21025
TANK	27.7854	27.0185	26.5944	31.2532
SKIL	2.44365	1.52595	1.24743	1.49727
EPRS	96.1134	100.207	88.7477	95.4177
VPS45	11.0092	10.5521	8.59348	9.52991
KCTD3	41.3318	33.3172	37.1085	34.2218
RPS6KC1	5.38125	5.74732	6.58386	6.156
RASSF5	1.61417	1.66485	1.69406	1.78035
CBWD2	24.2492	22.4375	21.1913	21.8842
SMPD4	83.1603	73.313	75.2515	71.2596
WDR33	25.8296	20.2426	22.2216	25.7671
CCDC115	12.8676	10.0445	11.632	11.7576
SAP130	11.8305	10.7388	11.3903	11.3107
BIN1	27.3608	27.1109	30.7407	28.2183
IMP4	189.182	155.197	177.083	167.625
HS6ST1	25.2087	22.4637	25.2971	24.3848
UGGT1	16.2967	14.2439	15.8	17.563
STAM	11.112	10.9834	10.4637	10.552
ABI1	19.6543	17.8641	18.1623	18.3842
YME1L1	84.7087	77.6892	75.2478	81.6889
DNAJC1	16.6703	13.2549	13.0029	15.6069
NIPSNAP3A	5.76046	5.1611	5.21305	5.5751
LRRC8A	13.2246	12.8108	14.3373	12.552
CDK9	74.2486	77.8287	84.2972	70.8176
TXN	507.531	482.234	416.732	471.567
ODF2	32.5049	26.6261	26.1575	26.91
KIAA0368	48.0096	44.8717	46.1886	42.9596

TOR1B	11.4232	8.59505	11.0382	10.6373
C9orf78	59.567	45.8104	50.3099	53.5335
SMC2	9.68042	7.8542	7.64843	8.7959
KLF4	9.97428	12.9123	12.9066	12.2551
TOR1A	23.783	20.9209	21.0781	22.6618
RALGPS1	1.15086	2.22223	2.38416	2.49225
FAM129B	117.801	114.575	130.542	117.775
ST6GALNAC4	37.4081	33.3358	39.3333	33.2656
TMOD1	1.21084	1.16057	1.17144	1.29453
DAB2IP	9.93136	7.48199	8.74642	8.22947
STXBP1	12.8093	11.7667	12.0923	13.0267
SLC2A8	15.1927	17.5131	16.547	15.3272
CDK5RAP2	16.6327	17.1873	15.0337	15.2187
SLC31A2	1.34552	0.98478	0.985631	0.967882
SLC31A1	17.8039	18.1268	17.5924	19.1029
ZNF189	3.65732	2.96214	3.45709	3.55431
STX17	4.14201	4.08738	3.92825	4.45823
PRPF4	52.5421	40.4292	42.9858	47.1809
FPGS	58.9986	52.6013	61.9436	53.8837
USP20	5.88811	7.36175	8.3538	7.1566
KIF12	1.29481	1.43721	1.65627	1.71427
ATP6V1G1	91.2546	73.8352	67.9785	78.1413
TEX10	35.2109	31.5535	32.2604	36.4234
GARNL3	2.81676	3.03454	3.77235	2.77235
MRPL50	21.7458	19.5205	18.4335	20.7075
DPM2	51.9215	49.9832	50.2093	50.5608
TSTD2	6.57224	6.83446	7.60238	6.92671
PSMB7	315.524	286.957	273.723	293.014
C9orf156	8.3491	5.25316	5.19659	6.51237

RABEPK	53.0071	47.3077	49.9807	49.4692
GOLGA1	7.23802	7.05202	7.67932	7.18543
XPA	7.45884	6.1175	6.47709	6.07567
NCBP1	25.453	18.9441	18.064	21.1041
ANP32B	235.229	194.748	202.404	191.015
PDCL	14.9884	11.2276	11.1489	10.8508
RPL35	2411.84	2268.42	2122.1	2130.37
CTSV	12.7039	10.853	10.5806	12.4948
LMX1B	5.00314	4.06983	4.90257	4.90984
ARPC5L	73.5104	59.7612	62.4181	65.528
DSCC1	34.8626	23.7822	26.5583	26.8155
DERL1	68.9848	43.5569	48.6183	56.865
MYC	181.844	142.578	149.444	157.735
NOV	0.930412	1.18649	1.2523	1.08
C9orf123	43.9272	46.3193	51.3386	45.7932
RANBP6	6.77609	6.62688	5.75039	6.56871
POLR1E	67.5756	49.1443	52.0936	58.083
PLAA	44.881	39.2099	39.1944	42.4713
IL11RA	3.40048	2.90288	3.34187	2.95433
UBAP2	24.0873	20.7511	20.7722	19.4237
APTX	41.1043	39.9562	39.349	40.6217
RNF38	6.70068	7.68275	8.11084	7.54563
TLN1	95.0892	95.89	106.589	95.1426
DNAJB5	2.9802	2.94682	2.97366	3.24547
SPAG8	1.89616	1.68696	1.76546	1.58377
DCTN3	47.3983	47.062	46.9128	44.1159
CD72	3.84102	3.71182	3.67954	3.18584
TMEM8B	2.33427	2.90467	3.14233	3.07103
GRHPR	81.0106	85.9146	87.9612	85.123

ALDH1B1	36.3986	30.3383	33.8888	35.577
HINT2	29.912	37.6142	32.1252	30.5446
ARHGEF39	18.2896	17.3793	15.9137	18.3927
DENND4C	5.44901	5.60298	5.62888	5.32798
RPS6	1701.82	1584.63	1574.26	1716.84
CNPY3	39.9325	37.6735	38.2163	38.0343
FOXP4	16.057	17.0371	18.0841	17.3065
PPIL1	50.1346	47.7951	49.8146	54.0165
KLC4	3.91968	3.77395	4.32556	3.90957
KIF13A	7.08055	7.71239	7.55681	7.49577
ZSCAN9	5.96283	6.13302	5.91245	6.35422
PIM1	7.36749	7.73981	6.96784	6.7094
GMPR	13.6089	15.2618	16.1636	15.6491
FTSJD2	14.3797	15.1846	16.3104	16.0252
TFAP2A	5.1965	5.98187	5.68518	4.61598
YIPF3	66.8756	71.7514	70.8685	70.4434
TMEM14B	87.8332	87.2275	86.4433	93.7082
TMEM63B	20.9795	27.7406	29.1206	25.5928
FRS3	1.90656	2.43667	2.44695	2.26373
TJAP1	17.6726	20.9651	19.626	17.8236
SLC22A23	5.11131	4.67657	4.80398	4.91015
TUBB2A	52.5507	48.4608	45.512	44.3529
LRRC1	9.59884	9.07212	10.7641	10.2386
BPHL	6.06887	7.50755	7.61374	8.29036
RIPK1	9.11202	7.83372	8.06804	8.02557
TUBB2B	10.6619	9.55901	9.66527	8.84123
MNF1	107.299	77.9154	97.6556	105.753
HMGA1	1534.52	1306	1464.01	1470.49
TCF19	3.03013	2.28865	1.99304	1.67353

FLOT1	14.8966	14.2296	11.474	9.85199
TRIM26	1.61187	1.50811	1.66588	1.75335
RANP1	1.05393	0.889392	0.834057	0.986569
IER3	87.6601	86.7501	71.8248	66.7521
DDR1	29.4771	29.9221	26.7153	25.3189
MDC1	8.18585	8.93012	5.53256	5.06058
PGBD1	2.01713	1.77588	1.74459	1.75006
ATAT1	1.96864	2.84015	2.15268	1.92585
TPMT	7.66907	7.04073	5.97228	7.05701
RNF144B	1.0512	0.944917	1.13156	0.987073
HLA-F	1.14411	1.16389	1.2217	1.20728
NRM	3.06858	2.83638	2.37764	1.89395
MTCH1	120.715	96.3111	113.296	110.308
VARS2	4.95991	5.80627	4.42203	4.55772
TAF8	9.02488	8.38122	8.06893	8.27216
FAM8A1	5.27204	5.77066	5.76488	6.19798
C6orf52	3.89259	3.98721	3.65251	4.43484
FGFBP1	5.85507	5.56168	6.3323	6.32657
FCHSD2	4.68228	3.22137	4.30756	4.66951
ARRB1	11.7807	12.8819	14.2867	13.4831
PRKRIR	35.6854	31.6236	31.3588	34.5425
ANKRD42	3.80169	3.80174	3.84112	3.92852
IL18BP	2.52934	1.95902	1.91734	1.99796
NUMA1	41.6672	40.1131	41.7894	38.9319
CCDC90B	24.1962	31.4922	30.0989	30.2901
SYTL2	2.36543	3.15199	3.31447	3.35814
RAB30	1.80448	1.75594	1.73982	1.43837
CREBZF	16.2344	15.4983	15.754	16.2288
PRCP	36.1472	38.3202	39.7084	39.6266

NARS2	23.0267	17.6923	19.4549	22.1942
RNF121	21.0004	14.4942	18.7546	18.7533
MRPL15	81.8122	70.5621	71.7708	81.6108
TTPA	0.577456	1.11866	1.14641	0.563754
GGH	38.2735	35.2512	34.1882	35.2626
TGS1	13.9162	12.4086	12.4086	13.6603
SDCBP	46.5283	41.3382	32.9787	41.0211
NEK1	3.31173	3.092	2.91399	3.30382
DDX60	2.43741	2.42027	2.56489	2.56487
SORL1	4.04482	4.32407	4.53127	4.46322
BUD13	17.0755	14.5266	15.3171	16.7549
DCUN1D5	74.3592	67.1812	67.6166	67.8041
YAP1	35.0829	29.1246	30.0952	32.7429
SLC37A4	24.7922	24.2398	22.6167	20.5551
RDX	42.8148	41.3024	40.3295	46.3993
PPP2R1B	17.2629	17.3371	16.8614	17.9679
FDX1	13.2685	10.1102	10.0978	11.4728
C11orf1	26.4077	29.3531	25.5336	28.3765
ALKBH8	6.95653	5.27782	5.32566	5.48959
MAP2K5	4.62014	5.33471	5.57629	5.74892
SQRDL	6.57422	6.85607	7.35455	7.10693
CTDSPL2	18.8361	16.8455	16.9255	18.8979
SLTM	70.1398	62.5695	62.6406	64.5126
THBS1	10.5074	5.97192	8.73706	6.96705
MAPKBP1	5.54112	5.64237	5.54932	4.59824
NUSAP1	98.9918	75.3474	76.0265	86.5242
NDUFAF1	14.9786	14.3626	14.3144	16.1797
KIF23	56.2856	47.786	48.4582	49.0845
CASC5	9.5737	8.03382	7.33782	8.70556

HAUS2	25.546	19.2463	17.7834	22.6692
RTF1	13.6527	13.9793	11.7671	12.9844
PARP6	25.4238	29.8853	29.3404	29.9045
RPLP1	2740.36	2652.79	2443.54	2277.66
PAQR5	13.2716	12.847	13.2733	12.7067
LRRC49	2.56709	2.88126	2.23756	2.2894
TUBGCP4	19.7044	16.78	17.0779	17.6031
RMDN3	21.7922	9.58972	10.8845	10.8328
ITPKA	8.91412	8.03799	8.86733	7.57427
UACA	9.72758	10.212	11.1935	9.39617
SMAD6	2.54732	3.01415	3.05237	3.8013
PLCB2	1.80004	1.89214	1.66943	1.76827
TMEM62	4.74269	5.24868	4.81801	5.14288
PAK6	11.2847	10.5502	10.099	11.3938
ADAM10	42.2944	38.5917	45.4298	42.223
DUOX1	3.90368	5.66688	7.08917	5.83771
ZNF280D	6.34282	8.37585	6.76936	9.05748
RSL24D1	96.7756	83.5089	79.417	89.1492
GCOM1	1.72877	1.41168	3.44688	3.2827
GCHFR	2.14575	2.92105	2.70624	2.12279
BCAR3	17.5108	13.0919	14.3598	15.1056
TTLL7	5.13899	4.35346	4.21379	4.52578
FNBP1L	9.47131	10.9421	10.7026	11.0382
CCBL2	7.69857	6.67976	5.47753	6.68645
GTF2B	29.0088	26.6437	28.8244	30.0769
RABGGTB	130.092	106.773	100.011	117.191
ARHGAP29	64.323	57.5852	60.6574	67.6276
RP11-122C9.1	86.6343	86.6768	78.2118	95.6063
DBT	4.92857	4.85279	4.34408	4.9068

RTCA	23.96	19.9359	21.9653	23.6171
IFT172	6.05949	5.95561	5.63132	5.30852
EPT1	19.2209	15.1126	15.5033	18.0853
CGREF1	2.21473	2.6359	2.54401	2.44617
HADHB	38.003	44.0181	42.7557	41.9135
KHK	5.97247	4.22569	4.74898	5.55263
ADCY3	93.0593	68.1309	70.3737	68.8019
PPM1B	18.0534	13.3026	13.6851	16.181
PNPT1	25.728	20.8759	20.531	23.8406
DYNC2LI1	8.37987	9.4308	8.33173	10.2603
SMEK2	14.0052	13.5563	14.0063	15.0248
THUMPD2	12.4833	9.89082	11.1426	10.7646
RAB1A	131.169	121.995	124.473	129.315
ACTR2	79.9453	84.159	85.5884	96.5668
PREB	93.6678	77.4919	89.2168	90.0559
SLC5A6	61.2537	53.0213	53.7673	53.4163
PREPL	12.6553	10.4583	13.1323	14.9179
FBXO11	22.5085	24.3	21.006	23.1014
ATRAID	115.184	139.141	121.918	119.268
CENPO	22.6887	17.7408	16.8232	17.2363
LRPPRC	88.35	93.7594	95.16	93.8357
DTNB	10.7454	9.50486	10.7352	9.24596
ACTR1A	71.3718	72.9069	74.7243	75.3185
TMEM180	38.0924	38.2688	41.7404	39.9535
MYOF	44.1291	48.2606	50.1617	51.6829
LOXL4	0.793621	1.46525	1.27424	1.17444
STAMBPL1	7.26116	6.18124	6.14689	6.63013
ATAD1	31.9296	29.9574	28.5007	32.326
KIF11	15.8636	13.0472	12.2673	14.5248

TACC2	12.166	13.6778	13.2635	14.5112
DUSP5	78.3647	69.4067	82.6874	83.6348
CALHM2	18.9745	22.3909	22.6688	20.3827
ARL3	8.75298	8.48987	8.1903	9.12314
CEP55	44.9673	35.3427	35.2927	40.393
KIF20B	8.16393	6.55645	6.27268	7.00077
ENTPD1	2.61603	2.35962	2.37027	2.54728
EXOC6	21.497	15.4126	19.587	13.9378
DBR1	19.3663	14.3525	15.897	17.4052
DNAJC13	10.5929	10.7515	9.6619	8.58181
GPR87	1.45069	2.54265	2.09111	2.18412
ANXA7	104.468	95.354	97.3362	100.943
FAM149B1	4.8485	5.84082	4.80931	5.32872
NCOA4	23.766	22.6469	22.4409	22.4813
TIMM23	110.824	90.4868	89.5578	92.6228
ASCC1	20.2247	19.0613	20.3757	22.6605
ZNF365	0.992944	0.952266	0.904249	1.14884
ADAMTS14	3.19579	5.54726	5.80675	4.87783
RPS24	2189.47	2067.74	1879.05	2232.52
DNA2	7.12042	7.77651	6.52683	7.34107
ATIC	131.132	125.188	126.353	128.609
SMARCAL1	7.68849	7.73303	8.38117	7.87651
BARD1	19.2069	9.33225	21.5446	13.497
ASNSD1	26.5845	21.792	22.5622	25.9151
METTL5	91.186	79.7226	86.4891	82.8177
SSB	71.9971	67.7077	62.083	76.9594
NAB1	11.6742	11.3471	13.8993	14.9181
PPIG	27.8205	21.144	23.8064	25.0596
FASTKD1	14.3842	14.4639	13.4552	15.2945

MDH1B	1.7781	1.78053	1.42325	1.59047
IDH1	26.5972	25.2169	28.5084	29.8697
OLA1	193.171	181.608	167.713	193.406
CIR1	5.44252	4.88057	4.89051	4.96725
SSFA2	663.891	588.547	622.702	632.092
FAM117B	1.16487	1.59039	1.52242	1.27171
WDR12	67.7608	65.1377	65.8397	72.5948
ABI2	34.476	33.1102	28.0407	29.9077
ITGAV	11.6395	11.9248	13.8229	13.283
SLC35A5	11.6948	7.31999	10.1762	11.8756
DIRC2	6.37711	7.73893	7.5871	7.30707
SENP7	1.67534	1.89897	1.75508	1.6793
COX17	90.3718	57.6097	69.4533	74.4245
PARP9	2.39644	2.57685	2.36468	2.46957
MNS1	1.09577	1.05162	1.04579	1.24246
USP8	17.3114	16.1509	12.3282	13.918
SECISBP2L	4.49035	4.83928	4.66608	4.76623
TMOD3	27.0269	24.343	21.1462	23.6287
SPPL2A	23.4026	23.9298	21.1164	24.4444
GLCE	10.02	10.2761	10.6577	11.1433
SHF	1.17222	1.05222	1.09905	0.983003
APH1B	0.924404	1.53384	1.88702	1.45423
VWA9	40.5358	39.0819	38.7799	40.9008
PARP16	5.17405	3.9288	4.62771	4.69839
PPCDC	26.1678	19.9695	24.4095	22.7501
SEMA7A	25.0961	19.3841	23.9168	23.2539
UBL7	52.3995	53.2015	55.6476	51.459
FAM13A	4.91551	5.05492	4.68498	5.16572
HERC3	59.2732	58.9784	56.3648	58.2244

HERC6	2.26316	2.47454	1.96598	1.87504
HERC5	2.61578	2.46075	2.24119	2.60561
C4orf21	4.75587	3.60064	3.66333	4.15347
AP1AR	25.1398	30.1342	28.9299	28.5592
COPS4	38.7811	32.8187	34.6103	36.2808
HNRNPD	437.044	358.747	387.499	398.462
SEC31A	42.1029	40.0574	39.0893	35.3874
AGPAT9	30.8316	16.7343	16.3408	19.2811
FGF2	1.60449	1.11075	1.02088	1.21042
BBS7	20.1898	15.4149	13.9111	17.1953
KIAA1109	5.33168	7.0273	6.50467	6.33589
RAP1GDS1	32.8788	25.5926	24.1656	28.114
LARP1B	13.9622	11.4693	12.036	12.4133
PDE5A	1.32136	0.79416	0.984185	1.03928
PRDM5	1.16102	1.226	1.75131	1.23502
NAAA	16.937	15.5642	16.0236	15.184
NUP54	44.7694	37.678	34.688	41.3702
BMP2K	9.79628	8.59992	7.82311	8.43013
G3BP2	90.5085	84.3805	83.2375	88.9943
11-Sep	46.2315	43.5446	41.939	45.7109
FRAS1	10.0946	12.0556	11.7035	11.624
SCARB2	21.2491	21.4299	20.7795	21.9818
CCNG2	1.71224	3.12108	2.03528	1.95455
CNOT6L	5.73858	4.49298	6.07644	5.46984
USO1	45.184	45.309	44.7791	53.1748
SHROOM3	6.68675	6.04872	7.43328	7.11242
ANXA3	190.061	174.507	166.791	190.258
PPA2	71.8388	70.3373	64.7386	65.2601
CENPE	4.67526	4.28534	3.5847	4.20583

GSTCD	9.12936	8.36973	7.82307	8.59732
INTS12	14.6498	12.0066	12.6164	13.7547
ENPEP	1.0571	1.78539	1.41859	1.59338
CASP6	17.3053	13.8204	11.7376	11.7786
HADH	21.0625	19.6544	18.806	19.0812
PAPSS1	37.8495	36.6624	37.7419	37.7987
SEC24B	15.2361	13.2619	14.3917	14.9726
PPP3CA	38.5089	42.1147	43.0062	42.6832
SLC39A8	14.8172	9.51984	9.46854	10.6199
MAPK8IP3	21.7574	20.4409	21.1113	18.3973
RGS3	15.074	14.0472	15.0576	12.4191
GUCD1	43.2523	40.8891	41.4735	43.2228
RNF185	10.3609	9.76997	10.5434	10.6687
B4GALNT3	4.65034	5.43476	6.04697	5.70318
ETV6	2.08297	2.06104	2.14523	2.2926
GABARAPL1	12.7906	16.1599	15.9327	15.6886
KIF21A	13.7178	13.3338	14.3726	14.645
CPNE8	7.5666	5.16243	6.14606	7.01149
YARS2	41.8433	39.3641	34.3863	38.4699
FGD4	1.40213	1.8721	1.51537	1.65499
ALG10	4.59974	4.92806	4.7102	5.00765
FAM60A	88.5651	84.42	74.7982	97.8703
AEBP2	16.0662	14.5502	13.9077	16.0674
ETNK1	24.0928	22.2005	23.7115	25.5895
ZCRB1	59.1241	57.2518	53.4532	53.6404
TMEM117	2.76475	2.202	2.13703	2.28906
PRICKLE1	1.0932	1.51896	0.977714	0.921058
C1RL	3.33642	4.34946	6.86108	4.61212
NDUFA9	143.89	135.27	133.328	140.343

CLSTN3	9.84097	11.8545	12.6104	12.2964
KLRG1	0.586708	0.913381	1.27203	1.35873
VAMP1	3.39343	3.29503	3.15738	4.00649
RBP5	1.2963	1.06641	1.40511	1.05345
PEX5	26.5166	24.4995	24.808	24.6971
SCAF11	29.9686	28.0894	25.8584	26.9458
LLPH	73.8362	56.8656	57.1447	69.3163
RPL14P1	2.12137	2.18751	1.80699	2.31576
LRIG3	3.02659	2.6448	2.99995	3.13392
9-Mar	8.97582	10.6076	12.2034	10.7724
PHLDA1	61.2149	66.6196	70.8176	74.5141
TMEM19	18.5661	14.6521	14.7409	17.5407
DUSP6	65.5146	73.8101	82.7919	71.992
POC1B	6.13278	6.52732	6.84552	7.48526
TMTC3	6.78603	5.67503	6.25299	6.82537
SNRPF	133.784	110.556	109.932	124.577
AMDHD1	5.03264	5.47856	4.96726	5.26683
NEDD1	21.5841	18.4282	16.7781	20.2746
SYCP3	1.40821	0.794232	0.904948	1.02215
GAS2L3	6.68942	5.25763	4.8287	5.4248
SLC15A4	13.0364	12.2529	13.5897	13.7653
TDG	18.5466	10.5938	12.2306	13.8344
C12orf52	20.0788	18.6793	19.8047	20.3215
SDSL	16.48	23.0389	23.4434	21.3624
MMAB	12.4579	14.0564	11.4188	13.1892
GLTP	25.1558	19.7129	25.1143	26.7168
GIT2	10.0771	9.26349	9.24321	9.5424
TCHP	12.9539	10.0846	9.82014	9.97509
FAM222A	3.61349	2.99828	3.6512	3.36185

NUPL1	46.417	33.7495	33.6359	37.5124
MTMR6	8.20745	6.91089	7.00836	7.75329
SLC7A1	27.0772	32.1566	29.3691	28.3741
PDX1	1.42019	1.11928	1.15577	1.0838
LNX2	5.52904	5.01817	5.47528	5.63906
SUOX	10.2765	10.6106	12.5704	10.6165
TARBP2	42.5672	39.9983	38.2047	38.3348
NABP2	44.8472	43.3057	41.3429	45.1903
SMARCC2	26.8275	27.8326	30.412	31.5596
BRCA2	5.17486	5.63658	4.51734	4.94843
KANSL2	27.2055	27.4712	24.4188	25.9893
CERS5	26.8596	27.8357	26.5616	31.6464
MAP3K12	7.63836	8.23784	7.63996	6.78991
GALNT6	8.63674	7.12859	6.44545	7.34964
CSAD	4.2257	5.26793	5.64142	4.0238
LMBR1L	16.2207	21.1463	21.3344	19.5465
C12orf10	88.5572	74.6529	76.7822	78.4248
ESYT1	54.8302	51.3383	53.6102	49.7116
TMBIM6	318.595	316.76	298.875	310.672
ANKRD52	26.5235	18.2098	15.3496	16.4183
ZNF740	13.237	14.0191	13.4125	14.1137
WDFY2	2.5342	3.12885	2.78177	2.76039
HNRNPA1L2	13.6311	11.8522	11.0375	12.0553
ESD	122.942	117.553	109.645	125.466
RB1	14.8139	15.3001	13.5877	15.7287
SBNO1	13.9833	10.7872	11.2485	12.6289
SETD1B	5.72755	5.19823	5.49053	5.08881
VPS33A	15.0602	19.0912	18.4001	20.2849
VPS37B	36.0449	38.3151	39.2898	36.9996

RHOF	79.7224	67.2916	75.3706	71.6965
DENR	49.7517	45.6973	41.2951	48.1982
DIAPH3	8.59376	7.33926	6.78481	7.67377
RBM26	22.5661	19.909	20.3548	22.3123
MBNL2	14.6681	15.5072	13.8707	15.5654
ZIC5	5.25294	4.50094	4.87837	5.46753
ABHD13	4.04486	3.70527	4.02998	4.21643
RAB20	9.0412	7.83584	8.36625	9.06271
GRTP1	12.2043	14.3773	15.0086	14.6643
CUL4A	51.2888	50.5421	50.0528	50.4058
CDH24	9.89327	8.81548	9.7264	8.59045
TMX1	33.9706	28.4673	27.379	33.156
FRMD6	4.26335	3.87838	4.78914	5.68625
SLC38A6	3.47643	3.38162	3.03671	4.49984
NAA30	10.1588	8.92409	9.00545	10.3406
ADAM21	1.49808	1.40924	1.43589	1.50827
DCAF5	9.12523	8.49641	9.51979	8.66305
RAB15	4.16531	3.96428	3.85669	4.26024
WDR89	13.0765	11.8247	11.7394	13.0114
EFCAB11	11.8136	13.6554	10.5887	12.1824
PTGR2	7.66971	8.33446	6.89129	7.91913
JDP2	5.03469	11.6578	7.92737	7.2541
FBLN5	0.94757	1.16885	1.10163	1.16757
C14orf79	6.48209	8.11466	7.4286	7.40831
WARS	113.741	122.268	101.04	110.764
WDR20	12.4828	11.4831	11.3429	11.1065
NIPA2	56.9866	49.5468	48.1706	50.944
HERC2P2	13.6135	13.9515	14.5493	14.0986
SLC12A6	3.19752	2.08157	2.79432	3.0279

MFAP1	25.7708	22.8419	24.4921	25.8368
TCF12	26.0946	23.4412	23.557	29.5266
SORD	69.5757	70.9619	73.749	78.9334
SERF2	705.87	693.816	675.668	656.586
ZSCAN29	8.32893	8.71628	8.75954	8.34243
LYSMD2	12.6597	10.581	10.2489	9.87847
SLC27A2	7.67932	7.19405	8.27382	8.80577
BNIP2	28.878	29.2168	26.8608	29.5589
GTF2A2	177.755	161.48	144.858	177.806
SRP14	275.717	278.697	282.695	301.251
BAHD1	14.2925	11.795	11.29	11.0734
DISP2	2.37554	4.78864	4.62828	4.71318
CDAN1	9.85733	10.0891	9.59221	10.6562
TLE3	28.3332	28.8787	27.3367	31.1463
ANP32A	195.429	162.718	163.624	178.216
COMMD4	133.814	118.165	120.886	114.202
UBE2Q2	4.80131	5.70885	5.21827	5.8393
ETFA	139.772	131.753	131.68	139.764
HMG20A	17.5127	17.4004	11.9084	12.027
SCAPER	2.19499	2.56573	1.56437	2.10863
TSPAN3	147.302	144.894	143.441	142.72
WDR61	70.6226	68.0746	66.5295	75.5828
NCOA2	4.10668	4.01457	5.38628	5.41715
NEIL1	6.26	9.49295	9.94939	8.1505
MAN2C1	29.031	35.8413	38.014	34.6356
DNAJA4	34.403	29.5588	28.776	30.9023
MESDC1	24.6596	27.5008	32.4234	27.6754
TPM1	12.5222	11.1175	11.1506	11.3412
IGF1R	1.99409	1.56823	2.64364	3.22449

ARRDC4	2.07697	2.04988	2.72075	2.64581
PIF1	12.5255	14.664	11.5743	11.7772
USP3	27.499	25.2572	24.9614	26.0582
BBS4	9.23223	13.2389	11.7112	11.3746
PML	8.60438	10.1643	10.2093	8.43866
CYP1A1	1.95293	2.03793	3.48223	3.02103
ADAMTS17	2.65292	1.47922	2.19437	2.37759
LINS	9.57738	7.67893	7.52087	8.72929
ULK3	26.5301	28.684	30.9508	28.9993
PCSK6	8.02345	7.31233	6.78293	8.69007
SCAMP2	46.76	46.9739	52.155	47.8413
HAPLN3	6.55085	5.45591	6.07119	5.3931
POLG	48.9105	46.9668	50.0088	48.9951
FANCI	48.3425	41.9518	39.2384	45.855
ABHD2	9.63466	8.47677	9.7015	9.24592
TICRR	9.69163	7.66136	7.99131	7.75826
DET1	2.03394	2.9171	2.99542	2.69546
MFGE8	27.0213	30.5275	30.0863	28.4463
ZNF710	3.30369	3.5486	3.57193	3.99483
UNC45A	51.5292	48.882	50.4104	45.8267
MCTP2	2.19332	2.26931	2.54822	2.71258
FURIN	21.3326	22.5416	24.9049	22.6926
IQGAP1	38.978	35.4938	35.864	38.5368
CRTC3	9.40091	7.90532	8.37167	8.68813
EFTUD1	5.87522	5.97636	6.01967	6.59319
SEC11A	160.018	160.086	147.482	173.418
GLYR1	26.0289	27.6194	27.9414	26.3152
PMM2	32.5615	21.678	27.8798	30.115
SLC5A2	0.590454	0.522009	1.2009	1.6468

TGFB1I1	5.89461	6.65225	6.88759	6.10285
C16orf58	25.4626	31.8014	30.4374	31.1291
ARMC5	8.65765	7.98943	8.791	7.39215
PARN	32.061	29.9098	26.2778	31.7153
FTO	15.553	13.4281	13.6001	14.8165
UQCRC2	198.202	176.501	195.913	187.443
CDR2	19.4261	15.6612	16.0644	17.2192
ARHGAP17	20.7212	19.7582	20.7452	20.259
NKD1	2.94708	2.27542	2.33193	2.72008
DHX38	40.3724	42.286	43.7441	36.9385
TXNL4B	14.2227	12.3345	10.5767	12.3522
MARVELD3	12.0209	12.006	12.5355	12.3526
ZFHX3	1.00112	1.29726	1.0292	1.11437
CLEC18B	0.532617	1.04297	0.672327	0.712181
CPNE2	21.4589	27.4391	27.9171	26.1872
NLRC5	2.24925	2.28823	2.6743	2.44408
KATNB1	14.7152	13.4432	12.4795	12.5879
KIFC3	14.4933	12.4064	12.9388	11.4646
NUDT7	1.88417	1.88429	2.04852	2.17504
GCSH	236.673	219.453	205.351	233.974
CMTM3	17.6042	18.0813	17.2665	16.655
NOL3	28.8639	30.5264	31.3323	29.2205
MAP1LC3B	28.0705	24.6699	25.1984	27.5663
MBTPS1	49.7535	56.1023	57.1473	58.1176
ZCCHC14	11.6434	11.6275	11.0478	11.3214
TLDC1	17.8835	18.6818	21.0844	19.2438
OSGIN1	22.5324	18.8615	18.7733	19.7269
RHOT2	58.877	57.0483	68.0302	52.1714
ZSCAN32	13.0113	12.2542	12.5961	14.2675

RPS2	6610.43	6400.78	6662.53	6320.87
NDUFB10	290.261	249.131	258.411	245.035
PDPK1	19.8778	13.2146	15.3095	15.0882
DEF8	146.843	124.892	128.307	126.714
TCF25	236.425	230.903	224.239	222.805
GALNS	12.1801	13.271	15.6368	13.3667
GAS8	14.703	12.4726	16.7824	15.1491
MED9	11.4496	10.1718	9.89605	10.0507
NCOR1	10.2829	13.3308	12.7289	13.3148
COPS3	115.569	94.8456	90.9451	99.1665
GID4	4.77286	2.96787	3.00861	3.01033
KSR1	5.62915	5.98993	4.74256	4.37236
CIRH1A	93.6466	68.6889	71.9959	76.891
RANBP10	8.42989	8.20526	8.19438	8.2983
CTRL	11.1517	14.3086	12.5599	12.3622
GFOD2	13.3188	11.5939	12.1332	12.1728
NOB1	121.44	108.639	103.687	108.455
PRPSAP2	25.6409	26.9724	26.384	29.4351
MYO19	54.6843	50.2884	47.7376	44.6208
DDX52	18.4816	17.0148	16.5406	18.0365
PCTP	8.97562	11.1963	11.3105	10.7891
TOM1L1	29.6068	34.5937	32.1323	33.2258
C17orf80	18.1561	15.5881	17.112	17.7598
TOB1	30.4626	30.0405	28.7151	28.7712
VPS53	20.5632	20.34	18.6185	20.1131
SGSM2	21.8685	19.737	24.0253	20.7909
NPEPPS	65.9585	68.4826	74.9093	73.3737
SKAP1	6.06192	6.0434	5.86991	5.96958
SCRN2	23.0937	29.8187	30.1032	27.7684

SSH2	5.95182	6.55879	6.33112	7.02273
RHBDL3	2.41918	2.19481	2.44127	2.4805
ARSG	2.53917	4.194	4.03911	3.86431
G6PC3	71.1356	87.7736	84.6134	80.2107
CLTC	173.599	164.224	165.887	172.81
BCAS3	4.07107	6.24241	6.32153	5.93725
PTRH2	96.3545	82.7837	83.9023	90.8355
SS18	96.0609	86.4621	105.78	90.6949
TAF4B	7.45071	6.06697	6.7519	6.73637
AFG3L2	51.3213	44.3447	46.0245	55.8367
SLMO1	5.92088	5.33541	5.33793	5.14931
IMPA2	15.2615	16.6465	16.4854	17.859
GNAL	2.67766	2.77342	2.26832	2.19773
SLC39A6	81.8083	81.5653	79.8222	82.5683
RPRD1A	38.5789	31.7866	32.4713	35.4011
C18orf21	20.8076	18.3657	16.5264	18.3134
GALNT1	32.6559	26.5608	27.2394	30.7476
GAREM	1.87766	1.70284	1.44691	1.56712
ESCO1	6.03378	6.15663	6.10679	6.89895
OSBPL1A	9.58323	8.43702	9.15263	9.14667
GATA6	2.23816	2.39736	2.75105	2.4262
GREB1L	6.12505	5.75286	6.65805	6.57657
C18orf8	28.6882	26.4674	24.0273	25.5391
PELP1	47.237	43.6237	46.9516	43.7086
NPC1	26.0173	26.1825	26.4906	27.2983
ARRB2	33.3812	30.1185	30.4795	29.9656
WRAP53	34.7715	24.7939	26.2315	26.7979
MINK1	27.1291	29.2196	33.8519	31.6948
SAT2	29.3555	29.3472	28.7433	28.1254

TP53	30.203	42.9246	42.9626	41.3703
CCDC40	1.84016	2.44429	1.98043	2.17126
ARHGDIA	732.69	564.402	629.15	561.057
TMC6	30.5614	34.6314	40.4518	34.2306
SLC16A3	447.99	370.276	411.229	399.422
TTYH2	12.0521	11.3222	11.9533	11.3451
RAB40B	12.0537	11.7751	12.6092	11.1929
EIF4A3	219.681	172.842	186.365	195.767
CSNK1D	169.632	123.393	148.01	148.327
ANAPC11	197.23	201.445	210.512	190.043
TBCD	77.4951	76.138	77.9581	70.8932
FN3KRP	33.3464	33.6948	33.424	36.0327
NARF	39.0416	34.3518	37.4774	33.2462
RPTOR	24.6956	23.0314	24.1642	23.555
FOXK2	53.0577	42.4013	44.3018	46.4777
TRIM65	18.7467	20.1192	21.125	19.7691
CBX8	10.0824	10.5822	10.7207	12.2086
SECTM1	8.67756	7.75275	7.78607	7.73343
AZI1	14.8165	14.8352	13.6124	12.5419
WDR45B	105.406	96.251	102.581	105.238
CBX4	22.2219	20.3571	19.8176	18.6074
RNF165	1.08088	0.960378	0.851623	0.870171
DYM	19.1623	19.776	19.5706	18.7345
ELAC1	7.22948	8.58119	7.41956	7.38426
MBD1	27.994	25.9384	28.3975	28.2285
SMAD4	38.8444	39.8283	35.5331	37.8668
ZCCHC2	12.4925	12.7653	13.2031	11.9207
PMAIP1	39.8592	43.4099	41.421	42.4375
LEPREL4	11.4342	13.6499	13.5612	11.6957

NT5C3B	68.117	65.4107	63.3765	63.0994
FAM134C	12.5879	12.9111	12.3631	12.2032
PIP4K2B	20.3662	19.1621	21.7229	19.3927
ERBB2	37.9573	28.0579	27.8637	28.5399
GRB7	7.16467	8.29741	8.36475	8.7245
MIEN1	81.4511	76.9307	78.4048	83.226
FKBP10	22.8891	25.1353	23.4038	21.3465
TXNL4A	214.789	204.15	209.044	202.005
hsa-mir-1199	2.80963	2.65665	2.22914	2.50176
SAMD1	45.3286	36.3077	40.0796	41.3585
BRD4	8.73865	8.87971	10.0388	10.3258
SLC39A3	51.8844	50.4865	49.6011	45.8906
NFIC	26.5584	21.3962	26.2965	24.1596
TPGS1	16.106	21.5111	21.1043	17.7017
PPAP2C	136.565	123.954	127.116	120.303
PRDM15	3.94954	3.5282	3.19968	2.91721
PFKL	47.5938	57.2314	62.0185	56.5853
FEM1A	2.31711	2.45105	2.98591	2.21395
VAV1	8.37534	9.51576	9.4042	8.44323
MVB12A	55.4273	63.2566	65.4471	62.4103
SH3GL1	69.5745	64.0937	64.3352	62.3116
DUS3L	65.9374	54.2475	59.0902	54.3557
DPP9	50.8667	43.3357	54.4065	48.238
CCDC97	16.1199	16.1852	15.4543	14.4559
TMEM91	1.28727	1.04267	1.26106	1.43121
ZFP14	0.948967	0.932685	0.823305	1.04445
SIRT3	10.8272	9.87378	9.44598	9.92681
IFITM3	475.235	444.032	517.54	470.025
ATHL1	10.4	13.3642	16.8616	12.3604

COL6A1	31.6292	39.911	38.2026	33.3329
IFNAR1	10.8654	9.25172	9.11277	10.4094
SOD1	409.609	373.419	345.044	344.389
COL6A2	28.2721	35.2484	35.4019	29.5253
SIK1	8.12159	4.88035	6.07377	6.2352
TRPM2	31.6906	26.2216	30.4313	27.5063
SCYL1	74.4121	58.458	63.4584	62.519
TMEM50B	7.88567	8.00265	9.24358	9.36863
APP	166.055	157.093	175.914	157.52
DOPEY2	2.23149	2.97198	2.44247	2.77231
URB1	13.9809	13.7925	14.9849	14.4201
AKT1	106.099	102.422	105.798	104.792
EMP3	191.597	147.653	156.203	163.99
SAE1	184.722	175.948	171.207	186.549
LMTK3	3.77202	3.83332	4.11603	4.02442
GEMIN7	33.5981	32.3835	33.3316	31.263
CBLC	14.05	16.4795	15.7928	14.7026
WTIP	11.007	9.83045	11.0663	10.7936
RNPEPL1	22.2836	24.5448	30.261	25.2249
CAPN10	18.8288	19.1167	19.8221	18.8776
ERVK3-1	37.1279	31.4168	31.4841	32.3052
ZNF787	34.7924	30.2719	32.024	30.6747
C19orf52	18.072	21.0691	20.9363	18.8945
FBN3	1.15465	0.632955	1.31233	1.47469
CARM1	89.9877	93.1953	97.0043	89.7638
EVI5L	6.69638	6.90788	8.00009	6.65291
PSMB6	389.814	340.697	354.823	351.943
ZNF473	6.73681	5.5258	5.8943	6.92319
FAM71E1	5.82613	9.2981	7.8814	7.60515

RPS11	3001.1	3018.95	2948.81	2946.8
PTH2	3.66497	3.21916	5.46326	3.32936
RPL13A	1688.44	1790.36	1668.76	1770.61
CTU1	16.7507	13.9264	15.532	15.0514
NOSIP	149.765	130.809	132.059	126.091
ZNF614	1.51547	1.32607	1.35921	1.38843
RERE	8.32247	8.6143	9.75944	9.17846
MMEL1	26.2312	22.3078	29.2778	25.885
C1orf222	2.76761	1.6681	2.60631	2.03038
PRDM16	1.41616	1.20564	1.11447	1.39842
EPHA2	93.4688	93.3748	102.805	93.1406
ARHGEF19	31.1336	29.0366	32.7734	30.8356
EFHD2	86.2003	72.839	85.9431	84.1019
PEX14	31.6722	28.0166	28.9573	28.65
PGD	129.772	124.156	115.525	123.557
MYOM3	0.843741	1.21618	1.28853	1.24418
SH3BGRL3	108.404	120.523	134.088	128.334
CNKSR1	11.0906	13.0563	12.8553	11.8669
RPL11	1658.98	1575.13	1462.86	1622.35
IL22RA1	1.27192	1.05955	1.2758	1.18512
ZNF593	13.1388	11.9484	13.0783	12.2929
C1orf216	11.6252	12.0912	13.1219	12.1373
KIAA0319L	10.8915	13.6531	14.4371	14.2345
EVA1B	27.8609	29.3284	31.9364	27.2817
PLK4	26.5356	21.4821	22.8997	21.7439
MAP3K6	16.3294	16.5455	16.7063	17.7693
GPN2	32.9546	31.4217	33.7812	31.9443
SYTL1	9.83215	21.6695	21.1934	15.6529
WDTC1	8.98553	10.1481	10.6219	9.93888

NBPF3	6.26998	5.42435	5.68774	5.229
HSPG2	4.90604	8.20364	8.33967	7.66301
ITGB3BP	34.4238	32.9439	28.1242	32.1258
SERBP1	312.211	259.78	261.791	297.368
BCL10	6.16901	4.92531	4.91773	5.45589
CYR61	21.7233	12.3736	12.3274	13.8834
PRKACB	35.8168	25.005	27.1921	34.5936
PIGK	16.0172	14.0893	13.574	14.6875
TINAGL1	61.7127	59.0225	64.2712	60.5744
ADC	2.76358	2.90643	3.14525	2.48894
RPS8	1486.68	1539.76	1391.49	1492.75
KIF2C	54.8558	47.601	44.8052	47.0236
PTPRF	70.1942	64.0145	64.9621	62.6616
MOB3C	2.08235	2.15104	2.51224	2.32728
LMO4	16.5774	18.2346	16.8448	16.5447
MTF2	23.9488	18.2264	19.1157	21.1409
SLC44A3	4.34612	4.25229	4.72474	5.82313
IGSF3	6.77968	6.47136	7.39608	7.08884
ZNF697	2.26777	2.74709	2.34653	2.46059
CTTNBP2NL	2.65525	2.77813	2.51964	2.75644
STRIP1	11.6596	12.6535	12.4973	12.81
PSMA5	230.803	231.98	219.651	217.088
CELSR2	4.74575	5.31564	5.8478	5.13006
GPR161	6.70925	6.41242	7.09065	6.30986
ALDH9A1	29.1539	31.2947	29.2291	29.253
ATP1B1	28.8654	25.0932	27.3635	29.6086
TIPRL	26.5505	25.0409	25.0738	27.9563
NME7	32.4271	26.8459	24.5894	32.001
POGK	19.147	15.1658	14.5863	16.4323

MPC2	39.7755	30.9374	31.8519	34.2515
CREG1	37.551	37.6505	35.0139	36.3905
DCAF6	32.8809	30.9952	29.4817	29.7276
TBX19	0.777377	0.890168	1.1639	0.946659
UCK2	79.2689	64.4799	67.9984	69.6561
TMCO1	80.5647	65.8279	65.4534	75.3779
POU2F1	2.59982	2.40929	2.4056	2.61643
MGST3	80.8818	78.7795	76.0782	81.6181
RFWD2	20.4472	17.8191	18.0285	19.668
UFC1	58.5498	53.357	55.0125	57.0794
PPOX	13.4374	15.4042	15.1191	15.4164
NUF2	18.2712	13.1509	13.555	14.8269
RGS5	1.85064	1.71842	1.33477	1.85585
SDHC	160.111	156.178	162.265	171.279
PFDN2	191.574	153.396	153.142	154.621
NR1I3	4.20877	3.82905	3.71946	3.34063
USP21	10.5205	13.2558	13.0541	13.6565
PRCC	76.0018	65.6228	67.0484	67.3222
RRNAD1	9.85027	12.5327	13.0707	11.5186
MRPL24	115.501	114.67	113.409	113.942
PIGM	2.91058	3.18053	3.8788	3.89188
ISG20L2	42.5206	29.8718	30.6661	35.0322
CRABP2	141.075	125.815	120.186	123.044
HDGF	243.86	203.954	211.005	216.183
ABL2	5.553	4.52681	4.81698	4.94738
XPR1	11.4469	13.6251	14.3751	14.5121
RGS16	3.68521	3.41767	3.24998	3.27523
TOR1AIP1	22.8493	17.9109	19.2517	20.0662
LYPLAL1	39.5288	40.0823	36.5592	40.4373

PRUNE	8.48016	9.94774	9.54346	10.2073
TUFT1	5.15055	4.08432	4.56311	4.75463
SF3B4	58.1407	48.0398	48.9974	50.3278
ECM1	1.11098	1.40638	1.47681	1.40825
ZNF687	9.27004	10.6833	11.7135	10.4613
TARS2	9.21179	13.3944	15.5811	14.8341
CGN	7.87695	6.16422	7.50383	7.78224
SNX27	10.4653	7.33579	8.11978	9.31426
SETDB1	16.6743	15.9763	17.1363	14.9086
MCL1	60.9801	44.054	47.2804	49.3526
RFX5	12.3513	14.0487	15.1198	13.5127
PI4KB	30.3805	26.1226	29.7401	29.2484
PIP5K1A	59.6489	51.1629	62.2812	54.4537
ANP32E	55.6382	38.7688	35.8137	43.0313
ANXA9	1.02871	0.703185	0.811531	0.69736
CERS2	185.316	162.405	137.522	128.306
ENSA	106.237	85.3365	89.3034	91.1818
AC027612.6	10.1168	10.5441	9.50042	9.46276
SEMA6C	2.19845	3.59631	3.18865	2.8826
MRPL9	135.588	115.503	116.371	125.019
ARNT	10.6575	10.1666	11.1956	11.4025
POGZ	13.3192	16.7498	17.4269	16.35
OAZ3	6.05553	6.73375	6.15719	5.99498
GOLPH3L	10.8766	10.0253	9.83173	11.0512
GABPB2	3.69017	4.281	3.49359	3.72124
IKBKE	1.36246	1.98818	2.009	2.10431
DTL	31.8597	27.6923	28.8771	27.0173
DYRK3	1.83119	1.93608	1.64552	1.42268
EIF2D	26.1847	28.0685	27.3168	26.5301

INTS7	16.0973	14.0278	14.5361	15.2607
VASH2	1.55186	1.84314	1.7505	1.78587
TAF1A	16.068	13.4761	14.4183	15.8984
SMYD2	17.8975	17.9318	17.7686	18.672
TP53BP2	14.4354	12.4695	13.0815	14.4282
ATP8B2	13.4217	12.434	16.7445	15.1404
ADAM15	97.7475	88.1683	96.7756	91.426
JTB	140.574	135.493	133.992	131.998
RAB13	47.9187	50.4247	49.4451	51.3194
TPM3	307.651	261.408	253.853	287.232
SNAPIN	32.0374	20.6897	27.1918	28.664
SLC27A3	3.6947	3.2599	2.55363	2.73461
UBAP2L	105.639	90.8308	106.024	99.6337
SLC39A1	52.1242	34.1066	43.7684	43.0658
HAX1	87.7682	76.5479	65.5692	73.9815
CREB3L4	5.95818	5.12534	5.2504	5.14912
EFNA3	18.5665	14.3314	17.2201	15.4171
C1orf43	174.861	176.68	176.806	170.63
GATAD2B	3.63184	3.92267	3.5383	3.76447
ILF2	361.99	307.121	300.661	321.28
RIT1	3.45941	2.67886	2.37134	2.69434
INTS3	11.932	13.0867	13.2614	12.4476
HCN3	1.84937	1.47269	1.85901	1.56321
C1orf131	13.7294	12.7427	13.3167	16.3986
GALNT2	22.8006	26.2864	27.5727	26.1787
TTC13	3.33587	3.61324	4.06223	3.3866
LYST	1.50595	1.05329	1.18643	1.12169
MLK4	8.26569	8.10254	8.07128	8.63931
CEP170	14.6884	12.1453	13.5169	14.1698

ACP1	124.686	122.184	116.94	124.087
SNAP47	26.8835	24.874	26.3209	24.3434
SRP9	170.171	167.351	157.837	181.983
NVL	14.7387	10.7434	11.7571	12.0864
SDE2	16.1974	12.2113	12.5092	14.7351
DEGS1	35.8579	30.4138	29.593	34.0211
FBXO28	19.8547	19.3917	18.8656	18.9415
ARF1	357.216	314.966	297.447	301.898
CNIH4	44.8561	40.5968	37.1133	41.2173
ITPKB	1.47378	1.70481	1.65447	1.52473
GUK1	211.499	192.045	217.961	190.758
CDC42BPA	12.7938	13.3443	13.3915	14.3057
CNIH3	0.664959	0.939872	0.850219	1.25197
C1orf35	25.2289	20.9786	23.5296	23.8008
MBOAT2	10.0536	9.60972	9.49793	9.69536
PARP1	156.503	144.515	158.174	156.516
PSEN2	14.5322	15.4753	15.9836	14.8448
PYCR2	100.076	102.228	103.901	98.4652
LBR	46.6296	55.6255	49.2185	57.1414
WNT9A	2.67942	2.89256	3.64151	3.14447
EPHX1	28.5186	18.4923	16.605	19.1644
SOX13	10.1562	9.85291	11.9675	10.386
ETNK2	14.4798	14.6794	15.279	14.7944
PPFIA4	1.31695	1.4203	1.16943	1.09161
PLEKHA6	2.22875	3.81953	2.73285	3.22667
ARL8A	20.2443	18.3826	19.8256	18.2055
OSR1	1.34195	1.33306	1.53549	1.31376
PDIA6	119.229	105.312	111.466	119.207
RHOB	53.5305	57.8003	48.831	45.1163

HNRNPLL	33.0395	33.9833	35.5906	34.7867
GALM	5.30649	5.42686	5.66869	5.19111
CAMKMT	4.82788	5.418	6.29761	6.65191
EML4	21.1821	20.7906	21.1674	21.3424
CALM2	387.286	294.303	294.499	334.853
CHAC2	10.8204	9.56963	9.2233	11.0397
RPS27A	1589.85	1495.44	1383.04	1477.19
WDPCP	1.69595	1.56234	1.69825	1.85172
VPS54	6.3337	7.19066	6.80781	7.64535
ASXL2	4.82649	4.55947	4.28011	4.64444
ETAA1	3.64138	3.63309	3.28153	3.73753
SNRPG	455.913	374.176	330.638	402.775
CIAO1	67.8595	52.9623	57.9445	56.0451
ZNF514	1.86535	2.46549	2.27993	2.32927
SNRNP200	131.631	144.284	149.133	137.483
MRPS5	55.9791	37.1122	46.7788	49.1051
TPRKB	50.2863	45.8872	40.6649	48.1124
EXOC6B	9.16521	8.68955	8.93643	9.07784
SFXN5	5.79977	6.73874	6.37293	7.63958
TEX261	31.8286	18.2222	19.1412	19.9182
DUSP11	15.0443	14.3011	13.4778	14.2752
NPHP1	5.02851	3.47951	3.48519	4.09012
MALL	97.7378	108.518	113.062	117.851
RALB	31.1684	31.0971	35.1187	35.0088
TMEM177	40.0542	35.129	38.0761	36.8985
NT5DC4	2.66649	1.16814	1.16826	3.28467
RABL2A	5.62159	5.75552	5.31277	4.99379
SLC20A1	59.8828	53.592	55.3401	55.5142
ZC3H8	7.50406	9.1894	8.09175	7.47539

LIPT1	4.3878	4.08672	3.68782	3.92861
FAHD2B	11.7063	13.5421	11.7209	12.0738
LYG1	2.11309	2.88546	2.99991	2.19949
UBXN4	25.1369	27.9812	25.8059	26.5067
SPOPL	14.0957	11.9399	11.2514	13.9984
POLR2D	42.3093	35.7244	33.4767	38.1039
AMMECR1L	12.8905	10.8726	12.1657	12.5996
PKP4	25.2265	23.1235	25.2247	25.0096
SCRN3	4.9012	5.54336	5.24111	6.40735
KIAA1715	9.57841	6.01297	6.3585	8.12459
ZNF385B	3.7561	4.45921	4.40815	4.11818
CDCA7	35.3693	32.4181	31.1933	33.4981
DLX1	1.4367	1.0621	1.45935	1.43098
UBR3	9.6409	8.92811	8.63841	10.0325
PHOSPHO2	1.34555	1.3628	0.997609	1.06625
GULP1	22.2445	21.8697	21.3782	25.5792
FAM171B	4.47228	3.36507	3.62412	4.10469
HSPD1	1047.06	831.335	835.987	935.978
CCDC150	6.37396	6.33516	6.12869	5.43889
METTL21A	22.0828	21.7178	21.2785	22.9367
NBEAL1	1.15578	1.66795	1.56999	1.8619
KANSL1L	1.81161	1.95344	1.86055	2.11463
SUMF1	12.6642	15.5023	15.8459	14.3847
RHBDD1	17.0526	19.3739	17.5014	17.7214
HES6	53.9002	43.6192	47.1149	41.491
ANKMY1	8.68991	7.94121	8.60388	7.20342
COPS7B	42.0806	37.3402	38.3878	36.2788
DIS3L2	17.2587	18.5787	21.1485	18.8737
FANCD2	32.3155	31.1312	31.7887	30.5734

TAMM41	7.57017	6.75821	6.87993	7.27328
VGLL4	11.8949	13.7767	13.8968	13.8637
RAB5A	19.8774	15.8085	16.2146	17.2788
FAM134A	46.3117	42.972	51.6688	48.5069
CTDSP1	39.9622	43.5701	42.7675	40.194
RQCD1	54.6942	51.7972	51.9114	55.8169
STK11IP	18.0945	19.8735	18.8536	19.4055
GMPPA	66.0139	60.2907	74.0487	66.5259
EAF1	10.5239	10.9623	11.3838	11.7488
DYNC1LI1	40.6561	33.8853	34.215	37.0038
OSBPL10	11.6702	9.38159	10.7741	11.1612
GTDC2	22.6114	19.7262	22.1281	21.0856
CSRNP1	6.78974	5.25826	6.12386	6.09873
SLC25A38	26.7021	18.956	20.7922	21.6829
GOLGA4	10.2373	8.32183	9.43398	10.0209
CTDSPL	25.0361	21.4048	22.8729	22.8566
IQSEC1	7.3274	7.17641	7.20957	7.10116
RPL32	1779.7	1729.04	1614.12	1731.71
PTPRG	7.28408	8.07656	7.83944	7.94094
IL17RD	1.52464	2.86105	2.71006	2.24113
SHQ1	18.2951	14.5174	15.304	16.4636
SLC25A26	15.3828	15.8818	14.9671	17.0871
UBA3	24.8719	25.6737	26.1491	28.4318
ARL6IP5	27.4848	26.0276	25.996	27.1066
TMF1	11.8005	7.23519	8.22012	9.49477
LRIG1	1.78869	1.63272	1.74033	1.65687
RP11-977G19.10	39.619	38.0911	37.4846	38.9923
LIMD1	9.66078	11.21	10.0691	10.0114
NFKBIZ	1.12329	0.893253	1.39606	0.978735

NXPE3	3.83644	2.85284	3.10011	3.49844
MYH15	2.6148	2.44061	2.42184	2.48611
PHLDB2	50.4916	47.2559	53.9576	56.2715
ABHD10	145.784	140.58	143.582	139.643
RABL3	15.4879	13.2717	13.018	15.1975
ADPRH	1.02506	1.07782	1.07096	0.951364
ATG3	57.1067	43.2968	47.9363	50.5966
BOC	1.23711	1.25865	1.17378	1.49519
SRPRB	109.198	74.1922	84.5597	86.4559
MED12L	1.05705	0.85155	1.13393	0.923259
EIF2A	127.05	114.135	113.557	131.644
OSBPL11	7.92426	5.25252	5.23975	5.93533
TRPC1	1.49088	1.2535	1.41609	2.03854
NCEH1	8.80133	6.33926	6.84589	7.34453
FAM86B2	1.41284	2.4014	2.54305	2.66247
LPP	2.03067	2.27927	2.33678	2.28635
TMEM44	6.58326	8.00292	7.83454	6.62636
KIAA0226	9.21986	8.3367	10.1198	10.1511
AMT	2.06512	2.34872	2.04011	1.56788
TCTA	8.19642	8.94666	9.34848	8.70875
NICN1	1.73467	2.45497	3.72148	2.77357
VPRBP	11.2039	12.8328	9.53274	9.81228
MANF	83.8675	70.7127	70.7477	71.2551
CCDC39	2.8424	2.17065	2.0893	1.82492
EAF2	3.59789	2.49051	2.85481	3.37155
TM4SF19	1.61069	1.72003	1.84521	2.07135
EIF2B5	41.805	38.7359	48.4851	46.8266
ECE2	12.9591	13.3596	14.0671	12.9116
VWA5B2	1.61659	2.40803	2.26675	2.10373

DGKQ	10.7426	12.2803	13.9562	12.3151
FIP1L1	48.184	44.334	44.6616	42.8546
SLC26A1	0.227937	2.14686	2.95242	2.87904
LYAR	53.9007	43.1285	42.5787	50.7192
CENPC	4.57879	3.60311	3.4082	3.7427
CORIN	1.04008	0.720922	0.942487	0.693596
ATP10D	1.85088	1.47225	1.84595	2.79112
OCIAD2	105.538	123.457	111.467	141.884
SLC10A4	9.83579	8.6528	9.7401	9.32721
SCD5	11.2	7.20511	8.22	9.64195
ENOPH1	64.923	53.1692	57.8939	59.5981
TRMT10A	5.79117	4.84522	5.19004	6.15577
KLHL8	16.1884	9.46729	9.94121	11.4769
PYURF	8.11741	7.55498	8.78857	8.53429
TBCK	5.13477	5.83089	5.86437	5.46744
CAMK2D	15.7121	19.3653	22.1235	22.6458
CISD2	33.304	32.0058	31.437	35.0295
DDIT4L	1.34574	1.63611	1.53002	1.78259
TIFA	15.7735	13.4257	15.4656	16.7664
SPATA5	3.91972	3.03469	3.06914	3.53694
CCNA2	73.099	54.2634	53.9609	59.8086
METTL14	10.7572	10.5813	9.69765	13.2337
USP53	5.70773	5.31184	4.34374	4.27478
SETD7	25.1091	22.2057	23.6751	25.436
NAF1	19.7607	16.3794	17.4915	17.6946
RPS3A	1471.8	1630.97	1582.96	1739.37
PDGFC	5.9687	4.94815	5.4715	5.5438
CBR4	9.57392	10.5559	9.96763	10.6919
CYP4V2	2.07951	2.69372	2.79325	2.86643

NDUFS6	86.7149	81.5492	76.2015	86.2552
6-Mar	67.0931	66.0666	64.4184	61.5692
NKD2	15.3066	14.6974	15.1391	14.3417
ADAMTS16	0.21596	0.324486	1.34912	0.74414
SRD5A1	22.4522	17.3019	19.4395	18.953
MYO10	13.2065	11.9565	12.857	12.5629
FAM105A	4.58402	4.91768	3.49607	3.35419
RPL37	1829.6	1726.07	1788.12	1859.88
SKP2	34.5068	27.9755	28.8831	30.5557
OSMR	12.6882	12.925	12.7293	13.4007
PLK2	80.6471	95.5588	87.557	85.5608
PIK3R1	3.0512	2.12915	2.3627	2.06052
LHFPL2	6.0453	4.76988	5.41078	6.21482
SSBP2	1.73943	1.64107	1.75286	1.84534
RASA1	7.66672	8.59402	8.50228	9.41485
GIN1	3.88458	3.16391	3.02166	2.81588
PPIP5K2	9.93892	9.20946	8.40133	11.5123
PAM	112.173	121.651	136.538	134.859
BDP1	3.60306	2.56245	3.01798	2.63679
GTF2H2	20.1311	19.3941	16.928	18.7427
SLC30A5	61.9221	51.3634	61.3689	57.2478
BTF3	705.352	608.184	528.851	583.373
FBXL17	2.98604	3.53439	4.69621	3.99413
TNFAIP8	6.08832	6.56276	6.3763	7.02514
FEM1C	7.7933	7.63067	7.34493	8.35474
COMMD10	22.4055	21.713	20.5729	24.2082
ATG12	163.909	140.732	151.076	168.579
YIPF5	23.8412	21.763	22.1764	22.4638
ARHGAP26	10.9377	10.6735	12.3435	11.392

SLC25A48	3.4467	3.10564	2.66179	2.9147
DDX46	72.8061	68.2444	62.1531	67.3387
RNF145	40.7537	20.6486	22.3083	24.2312
FBXO38	13.8506	11.9556	14.1494	13.8107
PCYOX1L	9.10399	11.8914	12.5842	12.2349
TNIP1	20.7961	20.077	19.5387	20.2002
G3BP1	133.485	121.464	120.489	125.446
N4BP3	2.74512	2.16163	2.73125	2.63452
NHP2	273.889	253.361	254.707	257.134
RMND5B	18.6126	20.1079	22.2767	19.7444
BOD1	47.689	39.7927	36.0743	43.8744
TBC1D7	14.4395	14.6884	14.5168	15.2717
FARS2	7.68538	8.02464	8.18648	8.51876
GFOD1	1.55953	1.13105	1.25215	1.28562
CDKAL1	4.32799	4.56743	3.97394	4.11048
ZMAT2	54.428	46.0416	42.8555	49.0984
TRIM7	23.3304	22.8108	22.7768	23.67
TRIM41	23.7756	21.3934	23.6864	24.2636
HIGD2A	105.363	99.2842	93.5686	98.2143
FAM193B	19.3559	22.3202	20.4072	20.831
PLA2G7	1.36797	1.90469	1.81965	1.87116
TNFRSF21	34.4135	22.7606	26.9759	27.8616
RNF44	11.878	15.1731	16.505	13.6796
MUT	4.93044	4.82361	4.84188	5.16019
DOK3	5.6967	4.33888	4.27392	4.04921
ABT1	23.3021	20.236	17.6478	17.3396
PPP1R18	4.1684	3.62885	3.09507	2.63581
PRIM2	17.6129	15.7125	14.6914	17.1059
RPL7L1	85.0319	69.3227	70.8362	83.9227

NFKBIE	13.4766	12.6551	14.399	12.8861
TPBG	12.845	13.5697	14.4167	13.0905
IRAK1BP1	1.08812	1.36482	1.1858	1.22882
PHIP	4.51408	4.3959	4.12816	4.63959
MMS22L	16.0252	12.4233	11.2995	13.7716
FAXC	2.26725	2.20308	2.58017	2.68362
PNRC1	2.10094	5.13125	4.14467	3.81018
PM20D2	17.3206	16.6411	16.576	18.1108
RARS2	19.7842	15.3683	16.493	18.714
TBC1D32	2.62663	1.73473	1.78497	1.86055
RNF217	7.62989	5.17663	5.87867	4.58326
ARHGAP18	25.7755	26.7571	27.6056	32.309
ABRACL	46.2908	43.7469	44.1542	50.3074
SLC18B1	13.5334	13.7577	13.5227	13.6626
MTFR2	16.3379	9.8914	11.6365	12.0741
SHPRH	2.23031	2.54676	2.20917	2.57564
AIG1	26.9022	27.5695	26.4187	27.5514
DYNLT1	191.986	196.52	183.735	194.026
TIAM2	2.45956	2.58918	2.64636	2.26292
TMEM181	20.7367	19.4707	20.009	20.6845
WTAP	56.81	52.5621	48.8293	54.7692
ZMYM4	14.4793	16.251	16.0988	15.4775
C6orf211	17.3182	17.3942	17.4518	19.2982
VWDE	2.22716	2.89062	2.81847	2.92102
GNA12	24.3494	16.8267	17.6564	18.9369
C7orf50	42.5807	46.4719	44.8547	44.7201
WASH2P	10.0876	10.3155	10.6335	9.49879
CCZ1B	61.6419	64.3433	65.212	62.6203
C7orf26	18.4569	16.2453	18.292	18.1591

RBAK	5.36698	4.50748	4.64978	4.72643
EGFR	9.79584	11.0521	10.3226	10.843
CDCA5	72.4966	53.9534	57.0507	59.7042
PURB	4.68117	4.46222	4.68358	4.82018
AC004453.8	1.48216	1.25193	0.499688	0.234886
SRCRB4D	1.07881	0.905963	1.20763	1.02757
MDH2	226.686	227.971	228.767	209.298
POMZP3	12.2513	12.2937	11.7428	11.2337
GBAS	31.9698	28.7526	35.9027	33.9426
CCT6A	449.974	360.701	370.964	380.674
PSPH	28.6091	31.5476	24.0421	24.3105
ZNF92	4.05219	3.48907	3.56024	3.96044
ATXN7L1	2.94501	2.35896	2.44613	2.46783
TMEM168	9.75419	9.89082	10.5091	10.9846
C7orf43	15.9147	15.4694	16.899	15.9733
SLC12A9	16.1888	16.406	20.8433	18.2207
GIGYF1	9.87108	11.64	11.0073	9.75058
TRIM4	9.12206	9.43563	9.24147	9.52568
MEPCE	37.4909	33.5713	31.4102	32.6015
TMEM209	18.0264	17.1656	16.4007	17.3171
ZC3HAV1L	4.70465	4.91588	4.05871	4.33395
TMEM140	6.1988	5.65899	5.92634	5.86868
TLK2	40.4765	39.9252	39.6265	37.8179
EPHA1	10.4564	11.3684	12.8757	11.9315
NOM1	19.2716	16.9441	16.9223	16.6994
NCAPG2	49.4996	36.8359	37.9355	42.2289
SHROOM2	1.16603	1.53511	1.70407	1.79941
C7orf55-LUC7L2	34.7582	31.3395	31.7507	34.9182
SH3KBP1	27.5136	30.1933	31.2169	31.4835

CASK	13.456	11.5559	10.9433	12.885
KDM6A	6.88361	5.97008	5.80416	6.09878
MSN	81.1048	37.365	34.2047	42.2617
CCNB3	1.00825	1.35007	1.09167	1.19919
HDAC8	5.48998	5.76375	5.78726	6.45899
CHST7	7.59978	5.85798	7.65815	6.67764
KRBOX4	8.74383	7.30725	7.457	7.08674
NDUFB11	33.7562	35.4347	34.9993	34.1209
ZNF41	3.07049	2.84709	2.8896	2.98433
ZMYM3	9.48126	10.6842	11.9347	11.5523
TAF1	14.4923	11.9237	11.7072	12.3899
NONO	537.894	505.764	507.073	557.712
CCDC120	2.34294	2.73911	2.96648	2.95071
EBP	61.2778	45.8569	48.188	48.2572
OGT	25.0148	30.0608	30.359	26.5777
SNX12	29.8203	28.3345	33.2045	31.4403
ITGB1BP2	1.04208	0.702823	0.794986	0.906643
ZNF711	2.0595	1.94601	1.86171	2.09289
DIAPH2	1.55477	1.41006	1.36865	1.64748
PRPS1	72.6741	56.7213	56.6517	64.0656
CXorf57	3.89657	4.33086	3.07391	4.09631
RBMX	220.877	173.713	173.323	185.149
MCPH1	9.83518	7.73921	8.85529	9.2087
MFHAS1	15.7256	14.6405	14.5424	15.1884
FBXO25	6.83629	6.53184	7.0533	6.80543
FAM58A	28.7744	19.7682	22.9639	21.0811
NSDHL	11.0098	9.39188	9.94454	9.83614
ZNF185	5.08445	5.61092	6.4858	5.49266
CETN2	7.68785	7.71561	5.83562	7.02652

GABRQ	3.10235	2.1398	2.28899	2.8317
RPL10	1312.6	1283.05	1197.78	1299.89
CSGALNACT1	1.06516	0.86573	0.858682	0.990678
ATP6V1B2	40.7183	42.0243	41.8021	42.0908
CCDC25	27.5074	26.6911	29.4834	29.8746
HMBOX1	5.11706	4.84749	5.0456	4.28711
BIN3	19.2683	18.2884	20.5152	20.1478
SLC25A37	34.1728	27.7943	31.5697	33.3947
CHMP7	39.23	32.323	37.2962	36.3189
DOCK5	12.8884	12.1829	13.6777	15.4679
PROSC	62.1272	35.9158	38.8055	41.8552
ERLIN2	11.8646	11.4546	11.7712	12.7463
RGS20	3.98603	3.67602	3.76505	4.14625
TACC1	11.445	11.0103	10.7608	12.4512
GOLGA7	29.9828	28.1454	28.2774	30.4588
PPAPDC1B	18.0587	17.2925	15.8427	16.639
GINS4	15.3497	15.6255	16.5635	16.2457
WHSC1L1	12.5508	11.2687	10.6047	11.8306
MRPS28	83.8392	80.5755	80.0811	85.5887
LACTB2	27.1349	25.7571	25.5002	29.731
TERF1	23.0872	19.49	20.4544	22.7895
RPL7	2568.01	2519.91	2382.33	2630.69
SYBU	11.4574	9.38363	10.3523	11.3682
MTDH	93.633	73.0035	67.9487	78.2111
LRP12	5.10612	3.34748	3.38891	5.31594
EBAG9	19.8241	18.5256	18.2787	17.9175
POLR2K	78.4067	67.7119	62.0372	78.0342
MAL2	81.7006	83.2269	81.5938	91.4335
EIF3H	278.727	274.454	263.311	265.489

UTP23	34.3676	26.2776	29.7084	28.361
NDUFB9	473.819	475.517	444.329	472.95
TATDN1	80.3554	75.6098	63.1743	80.1925
ZNF7	23.5042	21.9729	20.9928	21.3503
ARHGAP39	9.34165	8.76927	9.92843	9.17566
SLC39A4	62.2733	64.7245	64.2774	55.3761
NAPRT1	129.306	140.475	150.852	129.69
AK3	20.2699	22.372	24.0624	24.5204
UHRF2	10.1998	9.22184	10.4058	10.2992
NFIB	1.47446	1.01906	1.08002	1.48529
PLIN2	19.921	17.5184	17.014	17.7055
HAUS6	13.998	12.3363	11.3239	13.1394
CDKN2B	2.88763	1.41141	1.53285	1.72005
CDKN2A	65.0882	62.6319	73.6565	64.444
C9orf72	4.12801	3.65768	4.03545	4.86997
ZCCHC7	16.9362	13.1412	12.6174	14.3377
FBXO10	2.83351	3.70657	2.82505	3.37685
SIGMAR1	114.513	115.815	116.913	110.561
CBWD5	43.79	31.8303	33.6375	35.2981
CEP78	19.3271	17.156	18.2972	19.4315
IDNK	0.670447	1.18527	1.13595	1.34196
AUH	11.6732	13.913	13.397	13.5381
HIATL1	45.6932	34.0064	35.5445	40.3483
C9orf3	10.0855	10.0527	8.86142	9.31127
ZNF462	2.4976	2.30066	2.46271	2.55606
INIP	14.4035	11.0838	11.3784	10.899
UGCG	21.3719	13.3472	14.4577	17.301
SNX30	4.74976	5.17815	5.31941	5.70835
STOM	17.9976	15.6705	14.8084	15.5972

GSN	20.9162	27.473	27.9787	26.1258
MRRF	62.8272	50.8128	52.1006	58.9094
NR6A1	2.1134	2.65791	2.24336	2.18941
ALAD	6.09298	6.59223	6.17265	6.61534
ASTN2	3.96971	5.25192	5.69577	4.91294
WDR31	0.945718	1.13802	1.09399	1.13422
POLE3	93.627	75.2376	75.1333	81.4496
SURF4	81.3641	70.2146	66.0168	65.7194
SURF1	31.2477	36.313	32.0273	29.2011
SURF2	43.3416	35.6928	32.9913	32.6755
SURF6	20.5603	18.5857	19.383	17.8319
MED22	8.13887	8.50387	9.03691	8.46464
REXO4	18.7506	14.2373	14.7414	14.6203
RPL7A	1039.9	1056.18	991.595	974.65
GTF3C5	65.4384	53.8487	57.7676	57.4887
ASB6	23.2333	19.2832	20.8969	19.9072
PTGES2	165.148	159.971	174.215	157.188
NTMT1	132.592	105.455	108.915	106.164
CIZ1	83.7638	78.6082	80.5114	77.7125
SLC25A25	9.68446	7.77385	8.3511	8.21494
SH3GLB2	42.5054	38.1221	43.8139	39.3041
FAM73B	11.9386	10.6883	12.766	12.1745
PTGES	11.538	10.5532	12.0626	12.9694
LCN2	1.41416	0.843852	1.11846	0.952271
LRSAM1	11.6782	13.5543	14.9757	13.3659
GPR107	10.4499	11.8506	12.1837	9.95456
C9orf142	95.6624	91.1929	96.5357	91.4934
IDI2	1.8189	1.54202	1.66842	1.70346
INPP5E	10.1875	10.0569	10.3704	9.89104

SEC16A	16.7806	19.0208	17.8047	17.4709
DPH7	39.324	34.1567	38.6375	36.5326
NOTCH1	8.05231	8.48907	8.76618	7.43112
CACNA1B	1.19689	1.37393	1.33375	1.56458
NACC2	15.7045	16.2349	15.6988	15.4255
PROSER2	48.8478	40.5295	43.6518	41.3929
USP6NL	9.72081	10.0834	10.9781	11.1539
COMMD3	34.6492	37.1781	35.5295	34.2808
MSRB2	7.72599	12.5045	12.2804	11.1476
PDSS1	21.6483	19.7886	20.1109	20.9805
FAM171A1	27.3674	24.4473	26.1018	26.9684
FAM188A	11.6281	9.94709	11.7108	12.0584
RSU1	23.1481	23.0667	22.1329	24.3079
PARD3	14.5311	15.2163	14.1447	14.7309
ZEB1	0.695314	1.04444	1.1033	0.811509
NRBF2	12.9111	9.95972	9.97304	11.3918
POLR3A	10.7027	10.2864	10.9864	10.7315
HERC4	10.1597	8.78552	9.51616	10.3462
C10orf11	0.89273	1.22538	1.07048	1.43661
CAMK2G	16.201	15.7016	16.1095	15.2643
ADIRF	381.834	308.548	303.626	293.661
GLUD1	84.3673	65.6631	66.3012	71.9113
HTR7	14.6016	14.3139	15.1363	15.4826
RPP30	52.5921	45.6546	43.0535	46.6676
FRA10AC1	19.9173	19.7085	18.1043	22.0049
ADD3	10.4708	9.06097	10.7435	11.3282
DNAJB12	14.2669	12.0986	12.4464	13.1416
EIF4EBP2	14.6904	16.7556	15.2774	16.1795
TCF7L2	51.4614	59.6962	60.2913	55.6327

MKI67	46.2257	39.1674	37.6827	41.614
FUOM	18.6206	18.6786	17.5163	17.2887
LRRC27	1.29255	1.40629	1.89655	1.48768
MTG1	12.5791	13.5821	15.5211	14.5524
PAOX	1.01783	1.35868	1.74674	1.56759
GSTO1	156.632	152.439	152.153	164.16
TAF5	7.31855	5.70208	5.93018	6.22764
PPRC1	59.9693	51.5371	54.7727	53.1908
ITPRIP	6.93504	6.05655	6.14839	6.16404
CNNM2	6.74406	6.16344	5.89349	5.9552
PDCD11	57.5029	53.0895	55.6342	53.5786
RGS10	63.746	52.7964	49.1255	54.4926
BTBD10	16.22	16.3876	17.0446	18.1209
ADM	46.3688	40.1056	38.6364	38.5858
LIN7C	12.5121	10.5798	11.3404	12.5791
IMMP1L	36.4553	33.8765	29.9442	28.2345
PGAP2	32.597	32.4725	36.3977	34.4969
TUT1	22.4652	24.5036	22.336	25.1932
ZNF215	4.94404	3.70404	3.9737	4.3327
HSD17B12	43.9657	39.2824	38.3607	40.5295
APIP	28.5265	26.6027	26.6734	27.367
DGKZ	60.8872	57.0637	59.4866	58.6803
EIF3M	381.872	354.389	342.357	379.877
TNKS1BP1	15.7626	18.9162	17.7123	17.8206
SSRP1	222.745	192.649	188.822	190.061
SLC43A1	2.04791	4.38147	3.60841	2.81084
PTPRJ	6.28747	6.38642	7.11694	6.4589
C11orf49	17.9588	15.7218	15.9346	14.4755
ARFGAP2	50.9724	52.685	54.4142	52.5214

CELF1	42.9101	40.6341	42.4691	45.8239
C11orf73	36.285	36.7133	35.3701	37.3614
ENDOD1	18.4415	18.8415	18.9575	19.8739
CCDC82	6.43824	5.72722	7.00566	7.77776
KLHL35	1.18985	1.58993	1.958	1.47711
SERPINH1	114.061	116.463	111.607	104.164
CAPN5	2.26286	1.90494	2.45053	2.01892
INTS4	20.9945	19.6393	18.6575	21.1768
PAK1	33.2915	34.7588	35.6499	38.8296
RPS3	2762.37	2854.23	2837.65	2971.11
ZC3H12C	2.16648	1.57044	1.5597	1.72231
TTC12	4.676	4.63039	4.38617	4.08546
C11orf52	0.198971	0.160224	1.27679	0.0834025
NPAT	5.32603	3.82787	3.96614	4.69524
ATM	6.4772	6.35347	5.30219	6.77484
AASDHPPT	33.5401	26.2236	24.7323	27.4828
GLB1L2	24.7422	25.8808	26.0675	25.0694
SLX4IP	1.51408	1.32317	1.39974	1.37514
LAMTOR1	114.254	115.252	129.127	114.473
ST14	82.444	64.3553	70.0042	69.2892
HYOU1	53.6208	40.9267	47.2411	47.725
CSRP2BP	20.0654	17.4442	17.7734	19.9108
DAK	67.9002	79.7968	84.1213	74.1197
MTA2	64.1377	63.9131	62.6129	62.1341
TMEM138	81.2865	58.4373	70.1884	72.5862
FADS1	14.6403	16.8943	15.5717	16.4544
ROM1	15.282	16.376	19.3334	16.5399
EML3	42.5563	39.6962	47.0753	40.851
INCENP	41.2221	32.8721	34.7794	37.4016

FRG1B	6.88251	7.24347	7.12315	7.85945
CPSF7	56.0905	52.0819	55.4343	55.7194
B3GAT3	38.4169	37.1493	41.554	38.0479
EI24	197.558	187.32	172.333	183.769
CCDC15	1.02043	0.786181	0.515983	0.660567
CHEK1	37.5629	37.2625	36.5412	43.491
ESAM	6.33413	4.81001	5.21042	4.96723
MPZL2	6.58372	5.83657	7.06862	7.56253
SIDT2	11.2094	11.2377	11.5236	11.1746
TMEM25	7.63392	6.44555	6.78829	5.49658
TAGLN	5.83871	5.29388	4.81373	5.27054
COMMD7	9.78024	10.2849	9.78896	10.3987
DSN1	19.9353	15.7158	13.5471	16.0424
SOGA1	4.03563	4.06065	4.09885	4.00162
CDH22	0.546801	1.06873	1.10368	0.912674
LSM14B	30.9015	27.2468	29.7615	29.6283
YTHDF1	31.5991	28.4577	29.1654	30.8064
CABLES2	5.93615	5.64652	4.99658	5.35517
ORAOV1	132.393	86.888	82.1183	89.4823
TRPT1	20.1753	24.3274	24.4008	23.4202
NUDT22	31.0453	32.1571	38.1259	34.3008
FERMT3	2.96036	1.87454	1.75766	1.45351
PLCB3	46.2818	44.1727	44.8457	43.5902
MRPL49	90.9307	80.9361	80.7278	84.5973
CDC42EP2	12.565	12.6252	13.8871	12.6952
FAU	1679.28	1537.44	1511.99	1535.96
TM7SF2	8.67854	13.9267	13.5944	10.6136
VPS51	106.202	109.753	118.325	109.329
PPP4C	166.022	145.899	163.192	155.775

ALDOA	1944.79	1847.43	1890.87	1785.87
DOC2A	10.5677	11.3183	10.9688	10.4553
HIRIP3	16.0911	13.2213	14.1126	14.6751
TAOK2	25.9712	22.8766	25.9928	24.2637
TMEM219	36.8428	40.7061	40.6528	37.7368
HMGA2	64.8473	86.55	79.0599	75.1791
MKX	11.6463	13.4401	12.715	12.2587
MPP7	19.8782	20.731	18.9545	20.4615
ITGB1	173.458	176.146	183.532	193.354
CTF1	0.524444	0.98656	1.17108	1.21238
CWC15	14.2856	13.8437	12.9083	12.8146
ARID5B	0.763769	1.2496	1.12178	1.03237
DCUN1D2	10.0451	9.6094	8.15613	8.19673
TMCO3	28.9515	30.0075	28.5568	28.34
TMEM218	23.1405	24.2343	23.4453	25.5806
TIRAP	5.80363	4.38974	4.74438	5.00447
N6AMT2	8.87839	8.13209	7.40602	8.90817
LATS2	4.98104	4.12618	4.27203	5.43816
SAP18	310.055	259.378	240.686	296.24
KIAA1328	3.02864	2.59114	2.28541	2.2988
FAM124A	1.53142	1.45401	1.62474	1.58174
CTAGE5	10.6797	11.0241	10.9084	11.6363
LYPD1	0.961296	1.14797	1.18488	1.20931
LYPD6B	3.22475	2.99811	3.2413	3.08854
PDCD4	15.4635	29.2167	23.4829	23.6101
GPM6A	0.402879	1.22888	0.594833	0.44425
PRSS23	6.93083	6.23969	6.70651	6.90199
MTMR12	13.1371	10.5962	12.2719	12.7059
PPP1R1C	12.6982	15.8074	13.2445	14.251

CCT5	652.931	518.109	523.27	542.982
FAM173B	10.863	9.3421	9.97915	9.92474
DOCK1	12.082	13.1379	13.2286	13.331
DIXDC1	1.42292	1.84525	1.57592	1.75834
DLAT	32.6988	26.8601	26.3985	28.7007
C11orf57	19.378	17.1386	15.3646	17.7248
TIMM8B	95.3497	83.5995	78.1834	86.7679
IL18	38.3335	39.3058	42.3262	46.2475
PTS	39.541	37.1496	33.9146	37.8976
PIP4K2A	36.8945	31.5922	35.8152	39.5475
FOXO1	2.46337	3.9728	2.68866	2.93823
CRIM1	24.4356	25.0203	21.8595	19.2326
SEC24D	10.0631	8.54428	9.80966	10.0788
ABCB9	5.27599	5.98403	6.10936	6.16389
RILPL2	7.23889	5.90355	5.48431	5.94949
DHX37	33.7472	26.4054	30.1894	29.2431
UBC	1130.22	1011.03	1050.14	1013.39
ITPR1	2.00966	1.6836	1.56549	1.1951
PRSS53	1.14177	0.878114	0.80069	1.13039
SLC7A11	7.31334	9.84306	7.47343	7.85108
CCRN4L	18.8719	13.3732	13.4684	15.0006
DCP1B	7.24656	9.01798	8.83955	7.6925
NGLY1	17.4384	19.2148	20.8519	21.0467
OXSM	16.0534	17.0946	16.9964	17.1011
UEVLD	9.02038	9.53172	8.91562	9.61789
C12orf45	27.9571	21.5642	19.9689	22.9286
C12orf23	11.88	13.1042	13.8974	15.2598
BTBD11	2.03563	1.80494	1.60931	2.02605
UBE3B	8.17201	9.04392	9.59852	10.1649

ANK3	1.61189	1.35753	1.38382	2.09632
IPMK	2.69248	2.18646	2.4771	2.9565
PLBD2	13.5163	15.4647	15.5925	14.1522
DLG5	29.6926	30.3211	28.4836	27.034
SLC2A13	1.85999	1.48784	1.55362	1.62295
GXYLT1	6.28499	7.15836	6.58658	7.14099
TWF1	89.778	83.0693	68.9676	78.0099
DIP2C	9.38767	9.90689	10.3164	9.5942
EIF4E	197.515	137.592	150.116	162.809
MAGI1	6.64403	8.346	9.60739	9.28602
TEX30	23.9032	20.1536	19.6417	23.4187
CSNK1G3	15.2697	15.6747	14.4517	16.224
SRFBP1	4.1056	3.05046	3.2528	4.07625
FAM177A1	38.0232	36.8302	36.1572	38.3456
MBIP	9.52474	9.79267	9.56748	10.357
MIPOL1	3.48942	2.96556	2.99561	3.05836
EXT2	33.0962	30.7637	31.5527	32.5917
TMEM18	34.7925	30.9498	28.8225	31.7695
KCTD14	13.3786	12.8801	10.0932	10.5935
NDUFC2	226.683	195.872	183.088	219.685
NUBPL	5.62483	6.03795	5.71212	5.86506
NEK7	11.474	8.09054	7.83727	8.42621
FER	6.22317	5.29006	5.1366	5.27864
VIPAS39	8.77917	9.61112	8.1039	8.76948
ANKRD50	4.26362	3.58262	3.51558	3.98106
UPF2	11.8017	10.6763	10.9225	11.9583
CDC123	135.151	117.561	108.928	122.19
SCLT1	5.57429	3.73457	3.99783	4.59564
CCDC3	9.01026	9.48995	8.80026	9.17925

C4orf33	2.99091	2.94247	2.37977	2.91158
EPS8	37.3429	32.3031	36.5315	36.2056
ACAD8	14.4799	16.2653	17.7922	16.5369
THYN1	21.8045	23.448	22.1679	21.9718
VPS26B	18.7235	16.7879	18.9543	18.2102
NCAPD3	32.0702	28.3526	29.682	32.0518
VTI1A	22.5842	26.108	25.6346	26.8689
QDPR	35.1119	36.9811	38.5642	39.125
FAM160B1	8.26451	9.13323	10.2347	8.64552
TEX9	1.17719	1.59595	1.34516	1.47558
QTRTD1	25.5757	20.3031	21.4381	21.1203
MMAA	1.50218	1.66711	2.01566	1.69115
ZNF827	5.30206	6.32862	5.47234	4.83632
DPYSL4	3.12946	3.58814	3.83023	3.63633
KIN	8.74117	7.11698	6.03094	7.75657
PIGF	37.8118	35.6065	35.4838	37.4843
INPP1	13.3706	11.4567	12.777	13.6851
MFSD6	5.18354	5.84771	6.28964	5.82193
RNF144A	3.60576	3.7466	4.33239	4.416
ASAP2	4.22687	3.84817	4.65078	5.65223
ADAM17	12.222	9.47489	8.58341	10.4181
WWC2	8.63546	8.40799	8.2413	9.60838
MLF1IP	15.1631	12.0749	11.3842	12.1435
ACSL1	6.09374	4.3721	4.48256	4.84169
SLC25A4	40.5128	35.3074	37.161	38.8284
AMN1	11.8712	13.323	13.4103	14.733
BICD1	8.60108	8.25035	8.18172	8.12878
SAV1	41.2149	30.734	35.0075	34.462
CCDC122	1.32163	1.5442	1.38693	1.20027

NBAS	10.3329	10.5415	9.31833	9.01798
GUF1	18.3229	16.7949	16.9808	17.8645
SACS	8.28998	8.23674	7.24921	8.43869
CCDC175	1.86279	2.06392	0.904875	2.07281
CENPJ	6.62956	5.83862	6.4796	5.63785
FBXO4	5.26051	3.50029	3.80213	4.05207
C5orf28	12.8939	10.543	8.88959	10.4436
PARP8	4.96504	5.99897	5.24862	5.49098
CACUL1	23.7514	23.6232	20.359	23.4861
DST	35.055	38.5783	36.6843	36.6939
BEND6	1.26729	1.36936	1.3216	1.74671
TIAL1	113.199	97.4074	106.714	109.608
BAG3	55.5574	44.9792	48.5065	48.6206
SCHIP1	10.9988	11.4524	11.8955	8.231
LIX1L	5.4789	6.00871	3.07306	3.15823
NBPF11	2.04884	1.95653	1.91783	1.6493
AP1S3	65.9165	51.0915	54.6573	62.6341
RABGAP1L	6.30172	6.34743	7.00799	7.26059
CCDC74B	1.84567	1.87801	2.33377	1.93806
TMEM56	3.03623	3.18508	3.63023	3.72161
MZT2B	314.439	183.809	274.988	262.38
FAM168B	69.7253	63.1901	68.4532	67.7821
PTPN14	5.77048	6.49515	5.92321	6.21094
AC093838.4	14.2254	14.2989	14.225	14.0601
MGAT5	10.7955	9.06714	10.6737	11.4488
GPATCH11	4.95981	3.1335	3.30469	4.07129
HSPB8	2.49325	1.57397	2.04387	2.27609
GEMIN6	33.6834	26.1219	25.064	28.3926
RNF219	9.37714	7.24433	7.44792	8.46847

ARL14EP	19.3702	18.6299	18.5758	20.2216
EPG5	2.22667	2.24975	2.57479	2.31001
PSTPIP2	11.9107	13.3025	12.5591	13.14
ATP5A1	532.185	462.383	488.189	515.417
HAUS1	58.4501	47.4315	41.9583	50.8407
C18orf25	11.5643	8.45497	8.88515	10.879
SPC25	28.3243	16.0992	14.7835	23.532
PDK1	2.85011	3.41816	4.13299	4.29807
PDE3B	2.57599	2.38201	2.22069	2.51095
TCF7L1	4.97822	5.07966	5.53071	4.891
TGOLN2	21.4211	24.994	25.0916	25.3845
KCNK13	1.45304	1.17887	1.29126	1.22233
UHMK1	30.4334	18.2533	20.2838	25.1068
ATG10	7.62979	6.6581	6.61246	8.45019
POC5	11.6713	9.18508	10.7758	10.9241
TADA1	10.4657	9.75977	9.39709	9.81111
CWF19L2	2.56501	2.30459	2.39051	2.3444
JMY	2.15749	2.93398	2.90202	3.11168
HOMER1	9.70737	9.92605	9.78889	10.4225
XRCC4	19.4619	16.455	15.9703	19.3893
ZNF776	2.82092	2.90022	3.12861	3.14813
SUV39H2	17.1004	18.0024	15.7817	18.0275
DCLRE1C	5.40009	4.23896	6.60004	6.49317
RPP38	19.6486	16.746	16.8349	17.8272
NMT2	33.7842	29.8874	30.0822	32.182
ZNF837	0.592267	1.26489	1.21265	0.927354
USP12	18.311	9.69084	11.8686	14.9723
CCDC50	14.5351	14.9108	14.5388	16.6395
CAMK4	2.82411	2.14089	1.99572	2.49071

TRIM36	4.39342	3.64013	4.00687	4.29342
ZFP36L2	15.9395	14.4245	15.3516	15.7763
PAN3	7.84109	6.73027	7.89221	8.53128
PLEKHH2	0.896479	1.07643	0.953816	0.887159
PFKM	100.069	99.5411	95.0437	96.6207
TMEM123	138.27	126.175	134.132	145.381
SPEF2	0.788392	1.21775	1.20477	1.21583
MBNL1	67.0694	44.8034	50.3828	50.0424
NADK2	10.9913	10.1758	10.4387	10.9782
GPD1L	10.9513	7.89707	8.64441	9.96072
CCNO	7.41457	8.17015	9.25573	8.69724
SLC30A6	16.8817	9.43929	10.7483	12.763
PELO	18.5768	17.7483	17.1805	16.2849
SAR1B	37.4263	35.6018	34.579	39.1345
CATSPER3	1.61417	1.44771	1.32586	1.38946
FAM21B	4.44913	4.07956	3.44177	3.87306
GPR180	3.59958	2.81537	2.72416	3.10582
WDR78	1.15491	1.0974	1.14393	1.06974
FARP1	8.7559	10.3056	8.127	8.53403
IFIT5	10.2649	7.94947	8.10066	9.44216
PANK1	4.37363	4.75445	4.1162	3.73694
HNRNPDL	166.359	110.722	133.044	143.269
HHEX	4.14411	3.86104	3.89579	4.12148
UTRN	8.45397	8.61361	9.49751	8.82037
PTPRK	9.49893	9.33701	9.38639	9.82474
GGPS1	12.0588	15.0978	11.3321	14.6561
MARVELD2	5.63009	5.61567	5.65423	5.40482
RAD17	9.98414	10.5379	9.31582	10.0108
MED21	29.0244	25.0244	24.1362	28.0056

PLOD2	6.29548	6.50541	7.04901	7.34288
GPR125	27.6171	26.0949	25.4139	27.402
SREK1IP1	19.0202	15.3093	14.5542	16.0495
CWC27	17.2005	15.7103	15.176	17.3398
MR1	2.47311	2.88752	2.90202	2.96092
SRP19	91.0597	78.5636	77.4806	84.8047
CENPH	13.1179	12.0139	11.2736	12.0979
CDYL	13.9061	13.1882	13.06	13.6059
CARHSP1	53.7324	54.7671	49.7054	48.3988
BANK1	0.987883	1.20774	0.96535	0.964064
TXNDC11	15.7328	13.4534	14.4053	15.6022
DAB2	1.78277	2.23416	2.00361	2.31241
ACOXL	1.82891	1.67826	2.05563	2.0905
BCL2L11	5.28054	5.52658	5.57758	5.86672
ANAPC1	22.1454	20.4244	20.7306	20.819
CAST	38.7238	40.7584	41.1007	44.7095
SCOC	29.7046	32.1961	29.1002	36.0761
CETN3	34.5988	31.3439	31.9984	35.9336
SMARCA5	49.136	46.4952	43.2534	51.5781
SYCP2L	1.47306	1.61173	1.81315	0.900811
RASSF3	26.4747	24.2578	27.2118	28.3506
HNRNPU	254.296	230.691	222.669	241.445
RANBP2	13.9425	13.4628	13.0322	14.4427
AHCTF1	32.0112	34.0294	33.4605	36.1221
MERTK	2.02387	1.61011	1.86234	1.87022
TMEM87B	3.87181	4.15696	4.72067	4.38479
NR4A2	2.78788	2.8048	2.95284	2.99328
RBMS1	9.87209	7.54693	9.22149	9.76329
SLC25A27	2.46388	2.12077	1.83721	1.23387

GPR110	28.1929	22.2319	23.4824	25.3885
FAM49B	122.164	93.3978	97.4198	111.461
ASAP1	14.6695	11.1705	9.95269	11.0184
TRAPPC8	11.616	13.5537	13.0593	14.9893
LINC00467	13.2638	15.3902	13.0372	13.3493
INO80C	20.2002	19.405	21.2453	20.6332
LPCAT1	35.7065	33.8726	34.6722	35.4327
NMRAL1	61.9591	58.3152	57.4062	61.2858
UBALD1	20.0533	20.7862	23.043	18.9186
TMEM251	16.5439	14.1694	14.1122	14.4076
ING1	18.7823	14.6103	18.7017	15.9012
ADPRHL1	5.89589	6.81202	7.60935	7.38778
CMTM7	88.6926	95.572	89.9409	89.1665
FBXL2	5.06208	5.53859	5.33235	4.74341
UBP1	40.6602	37.9908	40.125	41.6333
RMND5A	17.9411	18.0204	16.4444	19.1773
RPIA	55.1054	51.872	53.6874	53.5074
TUBGCP5	7.89682	8.20046	7.60331	7.90293
LURAP1L	0.868646	1.24387	0.932257	0.692027
CNKSR3	6.66687	6.60564	6.22249	7.02892
GTF2E1	11.5295	10.3405	11.2645	13.0592
CFDP1	32.4812	32.5799	30.5307	32.8204
ZDHHC7	37.8098	33.421	34.6308	38.6799
JAZF1	1.42071	1.65661	1.66839	1.93674
CMIP	28.0479	30.4599	35.5228	30.8246
TRIP12	77.036	60.3146	59.2509	62.9056
FBXO36	1.61022	1.33363	1.53259	1.59806
CEBPG	20.3895	19.1869	16.6239	17.4419
KCTD15	18.6125	13.5759	16.1739	14.0425

MCOLN2	5.28702	4.64649	4.41478	4.55954
DDAH1	9.1155	7.58329	6.98016	7.71156
SREK1	21.312	20.9028	20.7324	21.5499
CHD1	24.0263	19.8624	20.028	23.4025
DGKE	1.18328	1.15359	1.13969	1.10883
HS2ST1	10.3865	11.0512	10.8042	11.2893
MSI2	45.7062	46.5421	48.3955	50.9052
ZUFSP	9.50224	7.6631	7.66425	8.49966
GDPD1	4.27439	3.23459	3.70351	3.80816
NUS1	34.6382	25.8327	26.3906	30.9145
PPP2R5E	16.5361	15.3788	15.1343	16.6371
C17orf103	0.795604	1.052	1.07635	0.931193
IMPACT	16.5521	21.3937	19.5733	20.5015
ANKRD29	5.05247	5.47248	4.70117	4.63534
C6orf57	2.22544	2.36234	1.86137	1.96204
C16orf74	19.5898	20.9409	24.8869	25.2512
TBCEL	5.0531	6.11522	6.13525	6.02663
ANKH	32.8746	30.7422	29.3341	30.8721
FAM105B	14.6322	11.1636	12.2904	13.5347
UBASH3B	13.8879	12.3948	13.1265	13.0459
ROBO3	11.2308	15.072	13.9926	12.712
TBRG1	15.3617	15.7414	15.6943	15.1612
NRGN	23.3302	21.7892	22.29	22.1533
FAM134B	1.59433	2.18864	2.03377	2.11147
GPR15	1.01135	0.855418	1.22613	0.888945
TOMM70A	33.5578	29.4811	29.7737	32.0013
PITPNC1	16.4484	14.5011	15.8077	16.6638
CC2D1B	18.1303	16.9646	16.5412	15.0561
PRKCA	4.02853	3.45877	3.83187	4.37142

LRRK1	4.28187	4.72793	4.95925	5.19808
CEP112	3.42652	4.10549	2.39269	3.31747
ABCA5	1.95535	1.87201	1.60199	1.74202
MIA3	23.5526	29.0597	25.4508	23.0128
DISP1	1.24619	1.27184	1.27544	1.42166
TNIK	5.61541	7.06417	7.4352	7.1013
FAM167A	1.16118	1.08989	0.862973	0.748053
NEIL2	38.1196	33.8586	35.5328	33.7519
WNT3A	1.99087	3.0131	3.08223	2.83386
OBSCN	1.87221	1.83895	2.02316	2.02999
LONRF1	8.4209	9.72635	7.18531	7.19221
TRIM11	20.5496	18.807	20.9214	20.1108
ENAH	33.3687	27.9731	28.131	30.7282
CCSAP	5.40297	5.78894	5.79324	5.40179
SH3RF1	25.75	24.2485	24.7031	25.3875
BUB3	260.735	221.034	231.626	235.629
FAM69A	2.76327	1.93582	2.1139	2.41283
ATP5G3	600.809	500.104	565.339	607.222
CNTNAP3B	3.50126	4.7108	4.28753	4.25866
PDLIM3	4.54183	3.7459	3.99388	4.4736
TCEB1	195.595	157.691	157.96	182.224
CEP170P1	2.24943	1.64883	1.87456	1.85748
CXADR	6.92512	7.86346	7.54888	7.72181
BTG3	69.7259	66.1865	63.2461	67.0853
C21orf91	5.57015	5.77775	4.80201	5.87549
RABGEF1	8.88431	7.23359	6.8717	8.04885
MRPL39	46.6414	41.5606	40.1974	42.5207
ATP5J	190.73	178.938	173.422	183.855
GABPA	7.86716	7.58118	10.0576	7.38023

TSEN2	16.8778	15.7204	14.8007	14.2097
XPC	11.6737	16.7201	16.5556	15.1176
CCDC174	7.04089	5.78519	5.48228	6.47229
FLCN	8.17383	7.44454	7.92515	7.02098
DPH3	14.4186	16.0592	14.5337	15.5315
OXNAD1	10.4641	8.29222	8.90045	9.31614
PLCL2	1.63295	1.78321	1.73955	1.77014
CXXC1	78.17	68.2004	75.7233	66.2447
SKA1	10.4425	8.42774	7.80843	8.37081
PPP4R1	36.9427	33.2336	33.7088	33.8581
MPPE1	13.7576	13.9836	14.6406	16.6672
USP43	2.57116	2.26402	2.74942	2.70207
RAB6B	8.32214	8.08928	8.93389	8.3546
EME1	20.1832	17.3174	17.0935	14.9695
ANKRD40	35.7273	34.9967	35.4126	37.1694
ZNF18	2.96884	3.41897	3.34623	3.51761
VOPP1	25.7205	22.2149	24.0065	24.8927
APOOL	4.48128	3.03471	3.24307	3.91896
CYP2U1	1.25541	1.68578	1.34164	1.5873
RSPH10B	2.36505	2.32744	2.36529	2.6057
FBXL18	8.85247	8.32974	8.91152	8.0933
PROM2	0.609535	2.17598	2.02909	1.21072
KLF10	37.8076	34.6572	34.832	37.7699
PTPRN2	1.0614	0.849334	0.949652	0.921754
AZIN1	104.967	92.0623	92.9368	100.697
ATP6V1C1	60.114	43.5988	44.1457	50.8172
TMEM55A	12.8249	10.1294	12.4135	12.3663
OTUD6B	20.4512	15.9967	16.1343	17.8287
CDK19	2.79706	3.17599	3.40934	3.10821

GTF3C6	34.7358	31.0079	29.466	33.0268
MARCKS	11.4616	14.7527	11.5721	12.885
TTC39B	1.3206	1.64616	1.43872	1.49414
AGPAT5	68.875	72.1119	62.1198	66.9155
MMS19	63.7658	56.0947	58.5353	58.3742
PI4K2A	10.7587	10.4599	10.6218	10.6537
MARVELD1	16.6039	23.0574	25.8112	22.6042
ZFYVE27	12.9835	10.8256	12.7933	12.6219
GOLGA7B	0.81382	1.73919	2.3226	1.794
TRMT44	9.39551	8.20788	9.41316	9.48423
SLC25A28	51.5347	42.5166	46.088	47.1146
HSPA13	13.7992	12.9737	10.587	12.2353
USP25	9.185	9.91819	9.54952	10.3244
GRAMD3	3.29354	4.38066	4.93046	3.78764
ZCCHC10	24.2306	21.2279	22.4707	23.7919
C16orf87	5.3423	5.2707	5.17296	5.78556
MOV10	53.2056	58.3447	51.1793	49.7697
RHOC	170.972	160.472	171.844	159.823
PPM1J	4.09103	3.51997	4.35529	4.07127
DBI	197.691	154.574	158.403	167.784
SLC16A1	61.4791	53.2014	49.0491	54.4463
HEATR3	12.0018	10.7002	10.158	11.1565
MKI67IP	97.4707	82.5196	69.8794	90.812
OXA1L	117.6	119.458	117.286	115.05
LARP1	59.707	51.3582	52.4892	53.4662
CNOT8	23.6289	22.2536	22.4256	22.4504
SETD9	5.84304	6.96203	6.22153	7.28438
MIER3	6.92069	6.37752	7.12101	8.12617
NUP205	37.5278	35.0637	32.0025	35.9875

ZKSCAN2	2.5612	2.20367	2.43011	2.56236
C9orf85	5.7837	5.88388	6.46024	6.54463
PIK3AP1	2.64874	2.60831	2.68781	2.65221
RBM45	8.20181	7.94373	7.82665	9.98997
C10orf12	2.25015	1.85233	1.76236	2.02063
PDIA4	139.658	138.725	143.761	146.23
KDM8	1.71315	2.05847	1.94341	1.83911
KCTD18	3.61507	4.5475	4.64049	4.67871
FAM126B	2.52768	1.85524	1.62958	2.18863
TMEM237	20.7771	19.0948	19.7129	20.83
FZD7	3.86116	4.52239	4.50302	4.25306
RNF20	13.5033	11.2159	12.0199	12.6412
PPARGC1B	2.73388	2.00587	2.32759	2.40011
SLC26A2	2.56844	3.11373	3.16196	3.26883
LSM11	3.1655	2.4406	2.60474	2.66938
MED7	7.15769	6.61243	6.91516	7.78391
RRAGA	37.4795	40.3674	43.6731	44.4225
ACPL2	10.4997	9.29818	9.98524	11.669
RASA2	3.12483	1.8663	2.35185	2.34889
RMND1	12.7328	12.9873	11.7224	13.7193
RAET1L	1.09057	1.64207	1.54076	1.31269
TMBIM4	31.9063	37.0261	38.2911	38.56
VBP1	26.5332	23.5646	22.1252	26.3834
VPS37A	26.547	22.6406	25.7805	25.392
KIF5A	1.17646	1.73079	2.02318	1.87408
TMEM185A	4.83594	4.36414	5.72642	4.59077
PSD3	3.80572	4.03123	3.73192	4.15707
C9orf41	15.1746	12.1957	14.127	15.8172
MCU	18.8163	16.94	17.4642	17.6116

ELMSAN1	7.34471	6.10752	6.85011	7.05265
FAM161B	13.8513	11.616	11.9211	12.1073
GNAQ	8.87467	8.6861	8.75474	9.49186
ADK	36.884	33.7334	33.1955	36.5721
DCK	32.8609	17.5602	17.0631	23.644
ALX3	1.98188	1.75956	1.60807	1.65265
DPY19L4	23.1521	23.3026	21.3784	22.7441
NDUFAF6	17.9619	16.5778	16.6941	16.3459
DRAM2	27.1957	24.7307	22.5758	25.5094
C8orf37	2.16966	2.05014	1.54339	2.04017
WHAMM	4.07579	2.91848	3.2268	3.26687
N6AMT1	4.92077	5.41931	5.35171	6.06475
RWDD2B	11.9307	16.0444	15.8195	16.0589
USP16	21.1634	16.9001	16.7744	19.8724
CCT8	381.811	299.239	311.192	352.777
BACH1	6.39238	4.72398	5.10242	7.11552
TSPAN7	1.78153	1.62843	2.02335	1.86337
TIAM1	7.80571	9.30709	8.45022	9.49371
SCAF4	13.0607	12.0496	12.1665	12.5098
RPGR	6.31287	5.56988	5.18813	6.42364
CDK20	2.96366	3.04932	3.12493	2.66189
PCGF6	21.2059	19.2563	17.8543	18.8291
ANKRD9	17.8951	20.7417	23.5819	20.2376
SFR1	18.4587	18.7303	17.0388	19.3502
SFXN2	9.72722	11.6443	10.5087	10.6964
C14orf2	334.573	320.242	269.876	318.547
FGF18	1.02941	0.747825	0.717755	0.874697
PCDH1	6.17801	7.15394	8.45734	7.43993
SH3RF2	7.38802	8.4767	8.55776	9.54986

UQCRB	275.166	279.961	264.891	298.453
MTERFD1	41.0095	32.6961	31.9245	36.9257
PTDSS1	114.929	102.847	103.283	106.234
RPL30	2328.53	2206.17	2037.55	2248.54
FAM122C	2.46912	2.42032	2.32519	2.8605
SUPV3L1	52.1392	48.2781	47.6914	48.3298
FAM122B	78.2611	67.0017	70.2868	75.9054
EEF1A1	5148.68	0	0	0
HKDC1	1.81088	0.991909	0.954049	1.20736
HK1	37.6966	27.2779	24.4591	29.0911
TYSND1	4.37917	5.04746	5.34211	5.37673
PHF6	12.918	13.6789	13.172	15.0335
CD109	8.55677	6.65202	7.44358	8.68355
UBE2L6	12.1614	13.583	12.5119	12.692
ZDHHC5	39.634	35.7428	41.5158	40.9887
MED19	14.7546	14.2626	14.3279	13.9275
ZFAND3	31.2363	35.4066	35.2259	35.0606
NPTN	69.4594	70.7903	71.6685	64.5906
KAT6B	2.2744	2.09906	2.15345	2.24694
SAMD8	7.37971	5.48166	6.0856	6.60594
RAB11FIP1	10.3394	9.845	9.92126	10.1197
UTP14A	15.678	15.7716	15.8029	17.1033
AIFM1	39.6055	34.7127	38.3367	38.4281
MAPK13	25.9752	28.427	28.8059	28.2263
BAG4	13.2154	13.5272	14.2226	13.0344
TBC1D31	25.3926	18.5554	20.7847	20.3319
WDYHV1	31.6151	21.8524	25.6721	27.2712
ATAD2	46.3454	36.4296	38.1701	43.7772
NSMCE2	25.7491	22.0726	22.2073	24.0207

ZNF689	7.76425	7.58681	8.09794	8.1666
PRR14	21.2713	19.0471	20.0596	18.2368
FBRS	23.9096	22.6225	21.7522	22.1586
FRRS1	7.66611	3.42242	4.16222	7.23126
PHKG2	18.2174	16.324	18.3135	17.6492
HIAT1	12.7259	10.6911	11.4666	11.9317
SASS6	7.13571	5.59958	5.59324	6.35892
MALSU1	117.963	108.298	108.591	115.342
VPS8	8.68997	8.78954	7.62354	8.39614
GALK2	11.7928	9.65045	9.85177	11.2499
B3GNT7	3.26716	1.6456	2.39734	2.32454
BUB1B	53.5541	36.2979	37.3013	41.6929
PDE6D	12.3669	14.2562	12.2058	12.796
EIF4A2	189.172	158.169	160.721	177.129
BRPF1	14.9517	12.9951	14.0691	12.2933
RPUSD3	39.6419	43.3031	44.8618	40.9945
TATDN2	21.3416	19.2636	20.2827	21.0651
SEC13	123.958	113.454	116.706	111.339
EXOG	3.41877	2.0826	2.63422	2.67235
NTAN1	35.334	33.9732	32.8041	33.6176
ZFYVE9	6.68989	7.65918	7.46326	7.8865
SMG1	10.1492	10.1435	9.22928	9.88338
FCHO2	3.51392	4.54325	4.61571	4.01674
RBPMS	10.2126	9.24108	11.1187	10.447
TMEM171	3.81647	3.68365	3.92633	3.6356
SYN2	3.69476	4.0421	4.61624	3.52148
NRG1	5.6147	5.6267	7.10277	7.74971
C1orf27	10.2772	9.73071	7.92852	10.208
CPT2	6.97877	8.67982	8.63393	8.5725

NECAP2	33.1955	28.6025	27.493	28.868
LRP8	27.6466	21.7178	26.4631	25.1013
PAXIP1	10.3429	9.30136	9.51697	10.3587
STEAP2	3.65964	5.17791	4.24063	4.14337
SSBP3	21.2476	23.1964	23.1426	22.8443
CLDN12	12.2098	12.3246	10.8925	10.675
MMP14	3.31404	4.67716	4.77767	4.29953
FZD1	3.65907	3.34866	3.63041	3.74771
GATAD1	8.41306	8.89091	8.67558	9.47369
SUSD3	5.20464	6.23499	6.37773	5.04925
DHRS4	29.5281	27.8696	27.3921	29.0809
DDX19B	18.0952	19.4727	18.7491	18.2785
ST3GAL2	8.1571	7.71677	7.45477	6.74538
FUK	4.94571	5.79388	6.79096	5.18156
DHRS1	12.6835	15.9638	16.3002	13.4848
AASDH	3.97137	3.79701	4.04696	4.3923
ZNF19	1.2076	1.56343	1.11309	1.08708
RNF111	21.1329	13.6485	15.6113	16.0418
CCNB2	60.2345	54.9429	45.8729	52.5664
FAM81A	5.01131	4.07049	4.45963	4.45494
MYO1E	16.0233	15.7466	15.7733	15.9368
APPL1	10.3308	8.85837	8.80217	9.64669
MUM1L1	1.35749	1.14899	1.27513	1.48106
AFAP1L1	2.66893	1.90346	2.15941	2.2241
TSC22D3	3.19509	4.14117	2.45642	1.95495
DSCR3	20.0084	18.9298	19.7468	21.7143
DYRK1A	20.4512	18.8297	21.3574	21.5283
ETS2	14.1271	13.8873	14.4318	15.0568
TSPAN18	3.59404	5.22531	5.58363	5.16019

SLC35B2	43.788	45.966	45.3896	45.694
TMEM164	8.02371	7.36073	7.2205	7.07843
C2CD2	9.2389	10.0917	10.6842	10.8701
TAB3	4.26756	4.46965	4.56168	4.68412
SLC38A10	37.4583	43.9315	47.4513	42.7142
ZNF618	3.984	4.56717	4.35071	4.17237
C9orf91	8.95599	8.30602	7.77325	7.87596
SNX22	2.93256	2.84876	2.75434	2.77636
UBN2	1.38386	1.49174	1.36121	1.46622
BRAF	4.91149	5.43497	4.97642	5.46077
PSMG3	93.865	83.7793	86.4032	82.5491
WDR19	3.56443	5.98152	4.14678	3.3334
SLC37A3	21.5307	22.9695	23.9184	23.5535
AP3S2	11.7912	13.0776	12.3325	10.9931
FMNL2	8.40217	8.2205	8.31246	9.05375
GAREML	11.4262	8.51843	9.09686	7.78912
SPPL3	27.9199	32.0017	33.5254	31.1443
DPYSL5	4.92877	5.59287	5.46879	5.16839
RAB28	13.8168	16.5398	12.758	15.2376
FAM213B	25.1083	22.073	24.9362	24.0253
PANK4	13.9034	12.6222	13.4468	13.5783
MEGF11	0.769361	1.2226	1.24372	1.28421
C12orf43	13.4558	11.9952	12.6321	12.6308
PEX10	19.652	22.6973	21.8693	20.936
RER1	178.69	158.323	152.923	161.009
RADIL	1.78172	1.69189	1.50507	1.74904
SKI	26.3869	26.7084	27.185	25.8928
WIPI2	32.1975	34.0133	35.1708	34.2556
LDLRAP1	14.8192	14.9931	15.6807	15.9729

AGAP1	14.4424	13.8571	15.9044	14.8392
KRTCAP3	65.4076	64.0498	63.2018	67.0739
PAFAH2	10.9322	7.06386	11.0398	8.64682
BRE	11.9896	13.2622	13.1912	13.7703
MRPL17	171.175	141.938	156.809	147.857
DUSP2	21.0799	18.8903	21.9239	19.6269
UBXN11	21.4253	22.2362	21.9288	19.2364
PTPDC1	2.01276	2.45618	1.94758	1.88181
GALNT14	14.1361	11.2521	12.5792	14.5303
NCK1	18.5014	14.8532	16.8589	19.3201
RHPN1	8.43704	10.3204	11.2796	9.50652
TPRG1L	20.8785	18.0344	22.1334	22.1213
AAED1	9.06081	7.17563	7.25726	7.88751
XKR8	7.71036	10.171	8.60531	8.65827
CNNM4	3.35151	3.38503	3.32958	3.45077
EYA3	14.2278	9.74119	12.3794	12.1317
DZIP1L	1.73129	2.09694	1.88227	2.76483
TMSB15A	4.18093	3.84359	3.64059	4.20599
FANCC	10.0031	8.05994	8.33828	7.20855
MRAS	11.4195	11.1457	10.7217	14.3009
WASF2	26.4882	28.8893	27.9545	27.6587
ABHD3	23.4273	23.7023	21.8059	22.2851
FAIM	18.7232	15.6496	13.2342	17.0155
FAM46B	5.81945	4.4159	4.9159	4.58398
RNF207	15.2128	17.7685	18.288	16.6999
CUL4B	22.4253	17.9462	19.5448	23.5143
GPR153	1.85029	2.5661	3.07505	2.58768
GPRASP2	1.17223	1.43322	1.56261	1.26408
HIST1H2BD	3.84564	5.08539	3.96404	3.99049

CDC25C	8.49327	7.31642	6.76336	7.20063
MITD1	22.8618	19.8548	19.1946	20.6397
EIF5B	77.0581	64.7748	69.0971	66.0918
CNOT11	47.2732	34.4441	37.4364	42.3787
TSPAN33	15.2891	11.9593	12.3635	13.074
AHCYL2	5.36189	6.25652	5.70048	5.69529
B4GALT5	60.5019	56.711	59.1223	66.8212
SPATA2	7.61959	8.04916	7.955	7.39124
FAM86C1	6.18542	6.6295	7.40109	7.51639
TSR2	29.3094	24.7526	27.1033	30.019
PPP1R9A	1.41513	1.38847	1.51339	1.48918
ZC3H18	26.609	22.0795	24.2411	23.9878
ZFAND2B	33.6317	27.8773	30.4893	28.2592
GDPD5	8.03879	8.2426	9.26257	9.72264
TMED4	29.1261	23.2439	25.8101	27.0482
PPP1R15B	23.1931	13.6935	13.8042	16.9422
COPG2	4.89651	5.42435	5.38967	5.17296
C11orf30	7.02069	6.72258	7.53413	7.97714
AGPAT6	69.3336	70.2117	73.3275	69.5552
TAGLN2	113.062	102.045	104.45	102.09
ELK4	6.86	6.78571	6.11434	6.91205
SLC45A3	5.87473	5.37114	6.79752	6.61051
DUSP23	42.656	43.6273	44.7662	43.8768
RNF166	29.7541	27.3173	30.7162	28.0434
NBL1	99.8041	74.5642	77.447	75.1775
F11R	53.0565	43.4824	45.5207	42.9884
USF1	35.3807	31.8722	33.9311	34.0034
SPATA2L	26.7857	26.6017	30.9382	27.8772
NIT1	24.2039	30.5845	31.072	29.2084

DEDD	26.7526	26.1851	27.9672	26.5349
ZNF276	15.5694	16.9978	17.6502	14.777
NPM2	14.1239	13.5997	16.6408	11.5635
EDA	1.22837	0.914265	1.02851	1.00113
CDA	12.8569	13.8186	15.6074	15.6615
PINK1	6.27603	6.85325	8.04718	7.70733
B4GALT3	49.9874	24.9683	36.8703	40.3894
DMTN	19.6018	26.392	29.3059	27.865
FAM160B2	18.1731	21.9269	23.0591	19.5844
NDUFS2	100.956	92.7871	108.823	103.063
TOMM40L	8.46077	6.53749	7.75786	7.89994
MPZ	1.79118	1.57712	1.71144	1.69198
KIAA1967	212.044	186.98	188.11	187.995
CACHD1	3.55826	3.33335	3.54101	3.66394
CDC42SE2	18.0777	17.8474	17.5956	19.309
RAPGEF6	3.032	2.63586	2.76717	2.8348
EPB41	20.7065	17.8181	17.5967	17.6915
MIS18A	32.0885	27.6169	26.8514	29.9276
ALG8	41.4937	38.8694	39.2462	47.7214
FBXW5	81.2095	79.4226	85.7162	75.8447
C21orf59	53.1626	51.2078	42.3715	53.8943
SYNJ1	3.69458	2.07364	3.61431	4.2704
PAXBP1	20.4072	19.4504	18.0525	19.0436
IFNAR2	8.48997	5.95988	5.99244	6.97221
MRPL10	112.452	103.493	102.454	99.6388
IFNGR2	21.0604	19.1374	17.5309	19.2611
GART	131.329	126.312	117.429	123.706
SON	62.6732	54.5866	52.0802	57.6653
DONSON	39.1493	32.0668	35.634	38.239

LAD1	50.6927	39.1724	45.1665	45.0448
CSRP1	52.7208	56.5057	59.1847	56.2295
HOXB13	3.53666	3.9592	3.82912	3.94276
ATP5G1	720.262	664.832	789.573	833.127
RCAN1	8.22665	7.08618	8.35989	8.52832
UBE2Z	86.8157	77.793	64.3494	67.6192
SNF8	222.966	217.236	216.955	222.763
CCDC24	7.41966	8.63715	8.74135	7.22834
RUNX1	9.87955	8.21174	9.7235	9.99832
CBR3	1.45899	1.23511	1.09427	1.13576
C2orf81	1.01831	1.76553	1.56149	1.47869
TUBBP5	2.54393	2.11902	2.31607	2.14183
MORC3	9.06397	6.2475	6.8386	7.32073
CHAF1B	25.5912	22.0506	21.1275	23.7186
SIM2	8.03562	7.75122	7.89277	7.52357
HLCS	4.97953	5.83949	5.6119	5.14544
SCUBE1	0.874002	0.968248	1.09016	0.931071
ARHGAP27	21.6836	25.4719	26.037	20.8772
ADPGK	38.0011	33.5984	31.3368	33.2457
PTMS	276.011	251.658	259.77	291.74
ADIPOR1	68.2928	56.3724	52.5691	54.234
CYB5R1	12.3044	12.5837	12.4245	12.9496
PSMD4	273.315	246.607	251.711	258.597
ATP13A2	43.3603	44.4545	49.0825	43.1242
M1AP	23.3262	22.3421	23.1056	23.1176
PSMB4	442.314	421.752	427.574	429.714
BTG2	11.3381	30.3936	24.9809	20.5888
HK2	16.8007	10.9608	12.6378	13.099
C1R	1.2518	0.982015	1.07087	1.09086

ALDH4A1	9.44885	10.474	10.7304	9.93053
THEM4	12.5616	12.8907	14.0533	14.3899
UBR1	6.09307	5.75738	6.0303	6.40052
AMFR	67.0153	50.0792	53.9645	52.4082
MED8	24.416	20.0996	20.8052	22.6191
RGL4	1.21519	1.38629	1.15904	1.07545
ISL2	2.77551	3.33051	2.89276	2.36935
RSPRY1	13.3307	12.5068	14.4288	14.7638
GPBP1L1	55.0434	47.0879	45.9571	49.2158
NAE1	81.0361	81.5888	72.7498	79.2377
TMEM69	30.2771	33.2498	30.7275	33.0689
GPR114	1.40066	1.92894	2.19618	2.00112
ACE	0.52443	1.13668	1.32259	1.11269
EFCAB14	19.1015	19.5246	21.5522	21.4632
CHCHD6	26.5142	28.0838	28.7137	28.8465
CTBP1	138.731	132.763	158.517	136.351
ZDHHC1	2.55018	3.92813	4.09174	3.42893
ATP6V0D1	45.6373	41.2859	46.6014	44.0868
ZFYVE28	7.56467	6.91102	7.85376	7.58524
RLTPR	27.4089	25.5358	27.3064	26.7329
RGS12	10.4538	15.4621	15.0581	9.65572
PSKH1	7.70516	13.11	10.5715	10.7533
ZYX	109.331	101.764	109.864	104.179
ABR	30.1504	23.1684	26.5377	25.3675
LYPD5	2.70344	2.30163	2.92337	2.5276
CCDC117	16.3061	15.2489	14.5325	16.1198
ZNF230	1.09098	1.01874	0.769649	1.25808
CCDC107	7.44201	7.67022	6.6359	7.43606
ZNF222	1.85218	1.88714	1.52899	2.13247

NPR2	7.23863	4.29808	5.58692	6.15164
ZNF235	5.11794	4.85333	4.64139	5.27424
GNE	4.18412	4.47438	4.47097	4.17081
TNFRSF13C	1.193	1.0713	1.06377	1.01859
ARHGAP35	12.734	13.8178	14.8902	13.8661
CALM3	290.093	239.822	271.451	263.944
DFFA	54.9331	49.169	47.641	46.8244
CCDC28B	2.25821	2.12797	2.21151	2.64668
IQCC	4.04209	3.0905	3.39017	3.24589
TMEM234	5.57381	5.83514	5.42251	5.14224
BSDC1	15.6509	22.9644	20.8272	20.1242
ZBTB8A	3.70148	3.45605	2.96288	3.24089
ATAD3B	56.1392	40.7756	44.7317	43.089
SSU72	75.1043	80.7788	75.9057	77.9252
UBE2J2	62.6372	60.2656	59.8581	60.6654
ZNF362	2.73525	3.80908	3.78397	3.82499
NR2F6	78.8813	60.9283	71.4176	65.4558
ANKLE1	8.6981	7.37684	7.18433	7.10371
CCDC58	91.4008	72.685	65.0085	78.8052
VMA21	16.8141	17.6057	18.6325	20.2546
KALRN	1.11314	1.48531	1.27366	1.39248
CILP2	5.82049	6.60362	7.08464	6.23809
FAM86C2P	4.59411	6.24362	6.5116	6.11434
SLC37A1	5.71192	6.65966	6.37248	6.42662
PDE9A	14.0128	14.6354	16.2095	15.5931
WDR4	22.248	15.5831	19.4221	19.0228
NDUFV3	20.8196	20.7349	20.672	21.2801
PKNOX1	8.17854	5.81932	6.53034	6.9624
U2AF1	196.049	172.509	171.099	191.106

RRP1B	38.8978	29.3177	25.7962	30.0125
PDXK	81.0863	70.3185	71.5736	73.183
G6PD	46.98	50.4342	50.0515	47.9732
CSTB	58.9229	53.6577	49.5894	52.1868
RRP1	55.6701	42.2044	42.854	44.9332
AGPAT3	25.0611	24.8485	27.5937	25.3044
TRAPPC10	15.3396	15.7669	16.4822	17.3495
C21orf33	45.2469	58.2464	60.1688	57.9998
ICOSLG	4.73736	4.19748	4.74464	4.46633
C21orf2	7.66336	6.4273	8.05191	6.63034
LRRC3	3.03573	2.90406	3.30175	3.466
FAM207A	50.7902	43.6039	43.3566	44.4112
RALGDS	14.21	17.9582	19.0247	16.8848
LSS	15.0606	15.5623	15.2262	13.4036
VAV2	18.0829	16.4238	17.9643	17.8481
MCM3AP	41.0678	37.9063	39.2184	36.3868
C21orf58	2.11451	2.09393	2.2958	2.00414
PCNT	13.499	12.2287	11.5801	12.1282
DIP2A	8.59128	8.66179	8.66317	8.59678
PRMT2	48.1898	43.0814	49.677	47.7127
CLDND2	1.85497	1.29514	1.48093	2.25325
CACFD1	1.69387	2.60015	2.53303	2.01293
SLC2A6	3.5028	3.63236	3.60536	3.20728
ZNF761	6.40848	5.80061	5.63638	5.48613
C9orf116	14.8941	15.7548	13.6442	14.5175
ZNF714	10.2804	9.47033	8.22402	8.99523
GPSM1	6.22158	7.95665	9.39293	7.8558
C19orf47	23.7981	17.4403	17.618	16.9081
C9orf117	5.44696	5.49839	6.26498	5.60253

TOR2A	9.82744	12.4065	12.1269	11.1685
ST6GALNAC6	21.1032	15.1087	17.1883	17.6396
SHKBP1	74.5822	81.3514	90.5512	81.1015
RDH13	6.04841	4.56501	4.7137	4.02994
ZER1	6.19332	6.49721	7.07254	5.85153
ZDHHC12	52.4994	58.7132	59.992	54.6729
PKN3	31.026	27.8997	28.8281	27.5561
SPTBN4	1.18311	1.21979	1.59508	1.03952
BRSK1	9.64636	8.65836	9.59522	9.64295
COX6B2	1.55961	0.471248	0.494696	0.490623
TAOK1	7.04091	6.82289	6.235	7.55909
MED27	40.1303	36.0685	35.0474	35.648
DEDD2	34.5224	29.5015	30.923	29.6171
SIK3	10.8447	9.23338	9.51053	9.39284
MPZL3	2.89266	2.92456	2.56393	2.76472
NEK8	1.8367	2.00264	2.21944	1.95251
TLCD1	39.8383	41.9248	36.9608	38.7253
PCSK7	18.7492	18.1331	20.2393	19.8811
SAFB	115.656	99.7187	118.262	92.2915
CHTOP	72.6304	67.2747	69.2406	72.7695
ZBTB7B	20.273	22.3105	23.8524	20.8256
FLAD1	50.5107	43.6932	46.248	46.8433
SHC1	100.476	90.1825	96.7445	96.8241
VPS11	5.54872	6.08574	5.40426	5.62685
NLRX1	6.44342	8.78596	8.93029	7.80237
ADAR	59.6608	55.4529	58.3774	64.2423
IL6R	2.22186	2.98137	4.22028	3.04465
UBE2Q1	130.363	123.481	114.056	110.22
CRTC2	24.2754	23.1119	24.0546	25.6558

ANO10	8.9484	7.80405	8.26368	9.10719
FDPS	134.436	122.294	124.971	133.932
RUSC1	56.5493	49.9628	53.4328	51.4209
GBAP1	3.06111	3.23361	3.44135	2.92094
FAM189B	22.2201	20.0745	20.4109	17.4321
PAQR6	1.44322	2.63827	2.42069	1.76034
PMF1	38.1846	34.49	34.5507	34.754
SLC25A44	13.9566	13.1604	12.296	10.68
LMNA	412.464	501.859	493.995	404.832
NBEAL2	30.824	30.4449	33.5561	27.2961
CCDC12	44.0341	38.2486	38.1486	38.3058
UBQLN4	47.932	47.7576	47.9936	48.6707
PPP1R35	37.5063	36.168	37.8047	39.1686
GPATCH4	65.6909	51.4786	51.4602	53.2842
STAG3L2	2.80759	2.60705	2.95649	2.27398
FGFR4	54.2291	53.522	55.3517	48.6612
NACC1	106.457	98.034	111.561	100.274
IER2	49.9435	44.7803	46.1612	48.8504
ZNF394	10.0573	8.90312	9.52704	8.77461
CPSF4	49.0932	47.5833	49.1903	45.094
LY6E	281.864	288.13	295.581	272.409
VPS28	60.0282	69.8107	66.1142	61.8201
TONSL	40.8043	33.3148	34.0069	31.6427
MUM1	33.0391	30.9114	29.8477	33.8424
RECQL4	222.764	180.313	191.48	182.383
LRRC14	36.2328	34.3586	36.8392	34.2838
ZNF333	2.64114	2.76882	2.40133	2.69982
PPP1R16A	84.6149	86.263	98.8649	86.6831
FOXH1	6.46415	6.80937	7.17144	6.29434

ORAI2	9.20432	7.0931	8.78759	8.27892
ALKBH4	15.2566	13.1962	14.2578	14.2876
SH2B2	3.18425	3.26903	3.26557	2.33951
C5orf45	119.7	116.712	109.721	107.129
SQSTM1	221.859	208.415	201.459	192.545
MGAT4B	139.588	143.147	150.738	136.16
RPL8	5149.34	4970.93	5017.09	4878.46
MAML1	19.1915	12.4861	15.6962	15.1294
LRWD1	35.8259	33.7627	35.9288	33.6052
NAPEPLD	3.81628	4.2752	3.43367	4.20254
PSMC2	103.346	86.9728	86.863	94.6772
MFSD12	93.2905	91.2933	94.6312	82.481
USP41	2.7507	2.65034	2.65959	2.86585
YDJC	83.0822	60.2466	69.5438	64.5111
DVL3	27.2021	26.5808	29.2132	25.8239
AP2M1	319.485	328.763	333.386	304.478
ABCF3	52.1904	55.474	57.1496	56.8501
PCYT1A	31.4041	24.8189	26.8153	27.4812
FBXO27	9.7507	10.5269	9.83129	8.92494
DMKN	186.687	182.875	174.202	187.88
U2AF1L4	9.4681	10.7254	7.6806	7.29027
BDH1	24.4758	22.9871	25.2543	24.6904
THAP8	1.72593	2.0972	2.24073	2.04528
DUSP14	36.7718	27.4815	29.0411	29.6048
LRRC56	1.28131	1.53741	1.69283	1.53187
PGAP3	3.71038	4.47144	4.58352	5.00072
FDXR	51.9921	93.0541	86.7836	72.4774
SAP30BP	118.138	109.725	122.894	123.674
ACOX1	16.2205	14.126	16.634	17.0601

PRPSAP1	71.3167	64.6352	70.3513	69.4204
SRSF2	220.269	195.329	185.908	210.691
ZNF577	2.8371	2.17288	1.89523	1.46979
TMEM143	3.63583	6.24935	5.98123	5.07891
TBC1D3G	1.26788	1.76754	1.81315	1.53283
ALDH16A1	17.5102	18.5286	17.4567	17.1894
ITGA5	21.1999	32.6247	32.7448	29.9
ZNF385A	24.0937	22.7203	13.5047	12.1154
MPP3	10.8986	11.2329	8.90582	10.0144
NAGS	7.48354	6.12893	6.16124	6.10245
LSM12	79.2036	62.5348	62.5945	73.1225
EMC10	103.228	108.801	127.423	110.573
JOSD2	39.2244	50.41	56.5816	44.5144
FAM171A2	12.1133	12.2334	13.9613	11.9901
DBF4B	10.6056	9.32945	9.22991	9.84768
PLCD3	150.535	147.045	164.596	136.257
FMNL3	1.08311	1.40464	1.43911	1.39065
RACGAP1	69.6175	48.2809	51.6083	54.3333
LARP4	30.2535	24.0206	24.517	28.3952
RAVER1	52.9604	48.9158	53.0012	46.7871
SPC24	26.6276	19.3137	21.0409	21.069
LEMD2	19.9957	20.0575	20.2005	19.1342
ZNF653	5.21202	5.31311	5.17693	4.74211
MED11	13.754	11.8279	13.7717	13.1185
CXCL16	40.8283	37.1216	40.7015	40.7219
RNASEK-C17orf49	14.4964	21.5451	25.0105	23.8315
TNFSF13	1.54213	0.989547	0.707584	0.883175
SENP3	90.1808	72.7682	74.3156	73.6952
FGF11	1.95891	2.17364	2.57828	2.1366

EIF4A1	1623.01	1308.09	1377.28	1387.29
RPL26	2073.53	2113.35	1884.27	2172.84
POLR3K	37.221	26.8241	30.2445	30.4317
SNRNP25	45.1527	40.1505	36.7785	43.0699
WDR90	32.7756	27.1131	31.2836	30.4595
JMJD8	133.761	130.708	141.939	129.505
CCDC78	3.73527	4.27696	5.07925	4.9633
SSTR5	6.473	6.07228	6.13443	5.93396
SPSB3	19.7	20.9381	20.8505	18.4317
C16orf59	30.7907	24.7831	25.0917	24.5658
CCNF	36.9067	31.0529	30.3214	29.5751
TBC1D24	10.3861	8.2087	7.65484	7.84044
AMDHD2	21.575	22.1057	23.9511	22.1494
CCDC64B	4.15333	4.84724	5.12781	4.66801
PAQR4	27.0389	33.6927	34.9296	32.1872
FLYWCH2	26.3588	29.3338	30.7587	28.7934
ZNF75A	4.8124	4.62255	4.22784	4.83804
ADCY9	3.46504	2.70287	2.96421	3.25186
SHANK2	1.56472	1.85411	1.60003	1.45329
CLPB	20.857	20.5177	20.2223	18.8822
NEU3	5.19705	4.24985	5.63746	5.71699
CYB561A3	33.4682	29.5805	25.7335	25.6559
PPP1R32	2.50286	2.77822	2.02666	1.82486
ASRGL1	29.4208	25.1847	25.6018	23.9621
GNG3	0.888685	0.820419	1.01001	0.955379
UBXN1	82.7885	80.2172	78.464	73.9813
C11orf48	149.34	123.926	100.85	115.021
TTC9C	21.8642	21.184	21.9656	24.0184
TAF6L	17.9654	17.3398	19.22	15.9848

NXF1	40.3726	37.2108	43.6672	37.8642
STX5	16.9833	17.019	16.2693	14.7129
SLC25A45	0.54973	1.50094	1.52993	1.35174
RPL29	1541.35	1626.64	1585.06	1529.75
DCP1A	11.5594	8.8325	8.99039	10.1339
SYVN1	20.4882	21.5192	23.0461	20.8814
ZFPL1	25.0793	22.3054	29.2081	24.6309
RPS6KA4	73.1037	65.506	76.7081	67.1998
LRP5	23.6498	28.801	34.1687	33.8023
TPCN2	4.85157	5.42224	5.43918	4.93299
FGF19	12.974	15.3962	13.1691	12.8171
CMPK1	48.5855	41.7617	39.1606	45.3693
SELRC1	43.9967	36.6654	37.6705	39.8388
ZYG11B	3.38331	3.88482	3.71975	3.9134
C1orf123	25.7334	27.8831	25.2116	25.9572
MAGOH	108.087	82.6969	79.2686	93.9334
ACOT11	6.24286	7.64024	8.67693	8.15933
FAM151A	3.50782	4.03555	4.79872	4.34142
PARS2	6.11881	6.07073	6.31174	6.12338
USP24	15.4377	13.0583	15.2049	13.9383
PPAP2B	1.17389	1.62967	2.40273	2.07344
NOL9	15.8407	13.9492	14.26	12.978
KLHL21	31.6016	26.7753	27.948	27.1996
GMEB1	10.9524	9.25159	8.88859	8.90664
SLC45A1	3.5453	4.22171	4.69149	4.50555
SEPN1	33.7966	39.0743	39.8159	37.6829
AK4	40.6048	40.0454	42.2946	42.9586
JAK1	19.1051	19.1929	20.9578	21.2637
RAVER2	15.2276	13.8559	11.7743	13.3949

LZIC	22.2635	19.0107	18.016	19.7815
RBP7	47.7354	39.7283	37.9893	38.7129
FBLIM1	0.792822	1.43708	1.01809	0.807503
DHRS3	21.3987	18.3304	21.055	19.9367
SDC3	11.988	13.9491	15.0616	14.1165
PEF1	59.606	53.8297	57.9155	56.5176
SYNC	10.8517	11.163	9.80948	12.005
RBBP4	148.759	146.358	147.745	162.409
KIAA1522	17.6288	15.5042	16.7462	16.7199
TMCO4	6.66848	8.19471	8.09972	6.57287
CAMK2N1	8.21737	8.31588	8.70412	8.82277
SCNN1D	0.773982	0.943715	2.45583	0.81087
MXRA8	1.83183	3.49812	2.91089	3.12467
C1orf86	71.9612	77.9576	78.8569	71.8088
MEGF6	6.34481	8.97812	13.9679	9.37006
NFIA	4.7515	4.97478	4.62291	5.24514
OMA1	20.164	19.2295	21.0551	20.9694
MYSM1	5.36601	4.8986	4.80249	4.77484
TM2D1	20.5042	18.1613	18.5636	20.1545
USP1	19.7679	16.1561	15.1605	17.6946
FUBP1	92.3955	67.3493	68.6776	68.821
DNAJB4	15.0408	13.2172	12.2717	14.5174
TYW3	30.9087	23.2883	22.9978	25.7141
SNX7	13.0575	12.6619	11.2784	12.1288
FAM102B	4.77517	3.93513	4.00163	4.61249
C1orf52	38.0349	31.9119	31.0051	30.9753
ATXN7L2	5.63167	4.87789	5.09849	4.60092
ZNF326	12.7268	11.3078	10.5741	12.132
GFI1	2.32086	2.60757	2.83718	2.58807

AGL	6.09491	5.79801	5.82569	6.37192
EXTL2	11.1379	9.70098	10.111	11.7691
SLC30A7	6.60883	6.24533	6.54062	6.96103
ZNF281	8.62116	8.3866	8.05474	8.69275
ARPC5	63.129	62.8291	65.9129	65.2419
ZNF496	23.7953	21.7131	21.6582	20.9466
IGSF8	16.1302	17.9939	19.2703	16.4817
PEA15	61.848	45.5309	47.1222	48.6481
PEX19	16.7277	13.5348	14.3135	15.4922
NCSTN	50.09	54.1136	57.3457	55.0532
OLFML2B	1.47961	1.25563	1.088	1.01439
C1orf74	5.40796	5.61701	6.18345	6.32877
FLVCR1	14.6631	12.1496	13.107	11.7554
ATF3	10.3935	18.0749	13.6648	13.8211
RBM15	8.101	6.36375	6.29999	6.84368
DENND2D	11.1657	14.7399	14.3106	14.2693
IER5	31.7201	33.2921	34.6879	32.1981
SNED1	1.40605	1.4043	1.35435	1.02922
BPNT1	29.731	24.6473	26.3467	27.5649
SPATA17	1.504	1.26007	1.11753	1.47929
C1orf115	9.53158	9.63211	10.0464	10.001
BROX	11.4681	12.0456	11.1572	12.2333
NBPF8	4.33194	4.36479	4.3022	4.26764
ACP6	3.85265	4.35013	3.98508	3.73695
MT2P1	3.60941	4.76889	0.351641	0.54775
TFB2M	27.2628	23.968	24.6415	25.458
CNST	4.01056	4.00998	4.11942	4.6105
PPP1R21	7.44865	8.21321	8.7468	9.70945
B3GALNT2	18.9849	15.3166	15.1589	15.6964

MAPKAPK2	17.5552	16.1974	17.7163	17.3798
CAPN2	106.592	103.164	100.09	101.4
MRPL55	126.019	99.4348	100.59	96.679
WDR26	33.3298	28.226	29.3616	30.5022
PUS10	1.96474	1.60364	1.45029	1.75258
PEX13	10.1413	9.20513	8.52062	10.8863
KIAA1841	0.927664	1.38438	0.977663	1.15445
MEMO1	45.0847	41.2954	41.9817	46.3475
DPY30	54.6431	49.7578	47.3615	61.9846
TYW5	4.77699	4.13416	3.59192	4.01986
C2orf47	52.7605	47.335	46.0827	47.4656
PQLC3	9.20865	11.8224	12.0695	10.6168
ARL5A	43.666	41.3233	38.5411	41.7797
CLHC1	3.21071	2.98948	3.4674	2.73759
CCDC104	23.8833	23.555	21.6833	23.2036
NUP35	29.7137	23.0311	23.95	25.9295
CCDC138	8.27771	7.21579	5.43106	7.17496
FBXO41	10.754	10.3468	11.7854	10.952
C2orf44	11.9509	8.06142	9.30082	9.13855
SMC6	13.4075	13.4545	12.1745	13.4392
VSNL1	7.30972	9.71896	8.89031	9.63622
CCDC74A	7.18645	8.03	6.40185	6.27642
H3F3A	299.05	295.687	283.279	293.821
ADCK3	11.9464	16.0657	14.417	12.7605
SLC16A14	8.02005	7.89482	7.12766	7.49553
ZNF2	2.55724	2.45638	2.5781	2.41645
SGCB	9.59292	8.56069	7.35218	7.47096
BBS5	3.16605	3.07318	3.14789	3.40875
SMARCAD1	13.7008	15.0727	14.312	14.8784

PDLIM5	31.9559	32.8943	37.3208	38.3279
OTUD7B	4.42879	3.90137	3.65952	3.62259
RPRD2	6.79166	6.90846	6.6515	6.82146
ANKRD23	3.80978	3.85435	3.91098	3.56768
MSX1	117.532	123.104	110.59	108.867
PACRGL	20.0871	17.4717	17.098	19.9347
LYSMD1	2.54251	2.15363	2.49391	2.44467
SCNM1	27.4966	30.255	25.8572	28.4847
VPS72	49.7211	46.9016	52.1298	53.8269
ERCC3	36.5825	32.4318	33.5492	34.9352
RNF149	26.1078	23.6633	24.3205	25.3385
IWS1	20.5009	17.9645	18.807	20.2633
BOLA3	68.5616	69.2128	66.4992	71.6997
CDC42EP3	12.8973	13.7652	13.3951	14.4936
S100A11	280.618	292.156	293.642	285.515
DHX57	12.5809	14.2928	14.0855	13.7824
TGFA	14.571	12.207	11.8947	12.5081
CCNYL1	6.95787	6.66449	6.83389	7.26004
FZD5	3.52085	2.88274	3.70519	3.64712
DCAF16	18.2105	13.388	12.84	15.4593
GNPDA2	11.9871	11.1537	8.62852	9.68195
PAQR3	30.9823	25.4022	26.4749	30.2318
NIPAL1	4.67481	4.14554	4.0103	3.97848
ANTXR2	2.9761	1.88499	1.87709	2.14879
HELQ	7.57153	6.80968	7.75172	8.34263
MRPS18C	132.366	102.687	89.635	110.603
CGGBP1	16.5912	14.3076	14.0562	15.3645
FAM175A	10.6596	9.87299	9.61183	10.0909
GPR155	1.75607	0.458435	0.968318	0.611578

PMVK	21.2473	20.1647	20.8251	21.0219
PBXIP1	3.06086	5.2211	4.90419	4.04733
PYGO2	19.5738	20.7855	20.3541	21.2005
HIPK1	11.7066	9.35369	11.2366	11.1204
C1orf106	3.45628	3.86674	4.56306	5.25522
YY1AP1	23.1425	19.5727	20.7922	22.0161
KBTBD8	1.96849	1.18866	1.21851	1.8909
EOGT	7.80434	7.34469	7.54097	8.19911
APOA1BP	114.179	120.138	121.622	119.86
NBPF10	7.70782	7.5833	6.99307	6.98469
POGLUT1	7.50833	8.10916	7.86932	7.62875
ATP1A1	161.829	132.007	136.476	150.154
EIF4E3	2.56931	2.48696	2.85221	2.93426
LRRC58	16.3321	14.2967	12.114	14.687
FSTL1	8.21728	8.68909	10.1144	9.56383
ELF3	20.4652	26.9361	28.1294	26.1979
TMEM183A	63.0076	60.0626	60.9546	61.2998
IGFBP7	8.32103	7.45357	7.23026	8.00647
TRIM46	0.880306	0.918338	1.09144	0.983782
KRTCAP2	290.661	233.66	248.078	239.073
ARPC2	195.62	197.982	204.414	209.137
TSACC	2.73978	2.0101	2.2182	2.89661
CCT3	634.339	572.523	575.504	560.288
TMEM79	3.57817	2.67214	2.96464	3.14508
SSR2	176.459	138.971	127.246	144.782
RNF25	43.4812	40.9794	44.4381	39.2275
STK36	14.616	19.1605	19.0576	17.0472
ADORA1	3.29011	3.21901	3.49622	2.95401
SRGAP2	8.32355	8.31585	7.41464	7.51334

KIAA1524	24.6711	19.0399	19.9241	22.8154
EOMES	1.65028	0.788609	0.800307	1.01697
CWC22	13.475	12.3353	11.8068	13.3034
AZI2	35.4435	38.3263	31.9794	35.0697
TGFBR2	9.81415	8.19291	7.04443	8.12436
ANKZF1	9.77912	13.4622	13.4647	12.5616
HDAC11	8.60666	12.8099	10.3054	7.81753
GLB1L	1.97335	2.21279	2.09323	1.94082
STT3B	80.744	67.7994	62.9069	76.3964
CHCHD4	20.0861	19.5561	19.439	22.2151
SGOL2	11.1752	8.35657	7.75456	9.73423
SERPINI1	3.92408	3.94168	3.12129	3.61951
CLASP2	13.0318	10.505	11.008	12.4337
SUCLG1	54.6248	47.7204	50.3378	55.8378
NUAK2	2.5469	1.6417	1.96293	1.99366
PRKCI	13.104	10.3515	9.61679	13.138
EIF5A2	15.3073	12.5569	11.1887	12.4362
RPL22L1	582.704	530.187	476.943	574.268
PPM1L	1.85919	1.98565	1.91405	1.757
SNHG16	144.946	140.123	128.759	139.406
RYBP	8.7227	8.58446	8.16592	9.06302
PPP4R2	34.5807	26.8683	24.5119	30.5615
GTPBP8	14.0874	8.75805	7.59039	9.36241
C3orf17	23.0644	23.7002	22.3153	23.9667
SPICE1	4.26728	4.05673	3.40315	3.79999
NKX6-1	2.98371	2.76536	3.18641	3.16514
CDS1	6.0223	6.15004	6.0098	6.4437
WDFY3	6.16395	6.25907	6.44749	6.70915
COX18	6.07674	6.55848	6.5884	6.77292

PTPN13	2.76926	2.74122	2.98122	3.1472
C4orf36	1.96681	2.672	2.58608	2.51761
THOC7	50.6384	47.0667	42.186	45.5102
ATXN7	6.2005	5.91084	5.40993	5.94465
PSMD6	94.621	81.7274	82.9335	89.4305
PPM1K	1.58498	1.3592	1.67512	1.62397
GMPS	71.232	58.1615	56.0079	66.4986
TIPARP	20.8977	18.6896	16.584	19.8495
CCNL1	29.0421	27.8867	24.6376	30.6745
HESX1	1.12012	0.814727	0.953949	0.881438
SLMAP	24.3559	26.4216	25.8199	22.7981
RPL9	1367.57	1493.23	1376.77	1491.35
SMIM14	2.62527	4.00148	4.13202	3.42434
RPP14	15.2665	11.9654	12.4647	12.2231
ABHD6	6.87924	5.43022	6.87662	6.88096
C3orf67	1.90959	2.08152	1.63572	2.04992
RBM47	4.79004	3.90104	5.31659	5.1575
APBB2	24.6926	22.9764	25.6424	24.8164
IL17RE	3.35942	3.35301	3.88905	4.02328
IL17RC	5.0389	7.43812	7.26142	6.57015
CRELD1	7.10634	7.47435	8.20192	6.77168
PRRT3	1.02438	0.980439	1.16923	0.964538
FANCD2OS	3.89937	2.32965	2.82737	3.29051
PCOLCE2	11.5878	11.1766	9.9333	11.5008
U2SURP	51.3751	38.9461	35.652	43.1928
MTMR14	16.1801	18.0526	18.3243	17.5298
TTC14	7.61486	7.54352	9.25067	9.09155
MTHFD2L	6.618	5.11327	5.77598	7.05658
RCHY1	39.8619	36.8567	36.4239	40.735

GYG1	27.1464	25.0366	26.2792	27.9798
HPS3	7.93167	7.51009	7.88621	7.95316
TM4SF18	1.66147	1.78683	1.93258	2.07304
TOPBP1	30.9649	24.9415	25.3783	28.3118
RYK	58.4999	49.3817	46.8284	48.4717
SNRK	4.27081	3.0423	3.62104	4.11648
UCN	2.10664	2.46798	2.84706	2.63748
ZNF513	8.18776	8.71672	10.0412	8.95281
SLC4A1AP	12.0039	9.41248	9.73446	10.1835
KIAA1143	17.2181	14.2072	13.438	14.4265
KIF15	6.68907	5.06284	5.35526	5.52187
WDR43	149.873	118.603	116.745	117.808
ZDHHC3	67.8504	56.7119	57.9819	58.0602
CDCP1	49.5482	45.1466	31.9913	34.1518
SLC6A20	1.04021	0.990714	1.66779	1.01359
LZTFL1	8.59994	6.47311	6.53397	6.74121
FYCO1	4.92759	5.88394	5.30094	5.01727
ELP6	46.4117	30.6075	36.0255	35.2176
DTX3L	1.78096	2.13846	1.86122	2.25327
ZNF148	7.01216	9.75468	8.29341	8.48495
NMNAT3	2.6835	3.12364	3.32581	3.13969
SMIM12	43.3073	38.692	41.7943	43.09
ZMYM6	7.45111	6.42504	6.23247	5.44698
TPRA1	35.3614	31.9849	35.4554	32.6231
YEATS2	16.8057	14.3989	15.0186	13.9731
ZC3H12A	3.52008	3.58374	4.05608	3.1479
MEAF6	34.8344	32.6203	30.0894	32.2837
SNIP1	6.12916	3.41081	3.7215	4.38348
POLR2H	161.457	133.34	138.304	145.639

CAMK2N2	2.3515	3.15287	2.96295	3.12318
LIPH	2.07671	2.75044	2.89909	2.67384
TMEM41A	15.4258	12.0172	15.1589	15.3006
RPN1	191.254	161.942	169.088	175.931
SENP2	14.7424	15.1231	14.2964	15.3776
HEYL	4.97652	5.52669	5.67589	5.52996
IFT122	7.80625	8.63233	7.9505	7.33495
RFC4	76.9649	65.0226	59.1096	63.6626
BAP1	44.4448	45.4198	45.7049	41.951
TKT	446.186	424.439	426.909	405.976
PRKCD	23.286	21.2047	23.4663	23.6231
RFT1	40.8076	39.2459	37.1413	38.0175
SFMBT1	7.8027	6.30787	6.93241	7.0896
GNL3	139.986	104.803	105.118	116.045
PBRM1	9.80799	9.12162	9.2341	9.76827
UVSSA	4.33408	4.70526	4.912	4.32071
FAM208A	16.5101	16.2725	14.9587	15.4506
ARHGEF3	6.23693	6.13938	5.46664	6.8511
SLBP	125.937	97.4717	99.5255	103.011
LRPAP1	84.6238	82.8973	85.0024	79.3455
SLC51A	1.29742	1.33679	1.17702	1.49755
UBXN7	5.59508	5.81918	5.13985	5.31522
RNF168	7.87249	7.61821	7.80629	8.63738
PIGX	15.8635	12.8511	12.2941	15.6236
MFI2	17.2292	21.2196	20.1935	18.7284
ABLIM2	1.81068	1.74472	2.54662	1.90003
C1orf50	7.32987	7.91115	7.8447	8.33461
ERMAP	1.11752	1.54928	1.42301	1.38568
ZNF691	5.90364	6.0424	6.43325	5.7499

AIMP1	38.2512	34.6646	32.6343	36.4136
SGMS2	12.3051	10.4013	11.3698	12.7632
METAP1	74.5236	68.1465	70.6271	72.2703
DNAJB14	10.4413	9.41782	9.61339	10.3366
H2AFZ	838.536	760.274	748.411	811.074
SLC9B2	15.5231	13.8515	13.6897	16.1298
BDH2	3.39829	3.35403	2.80512	3.20545
PGRMC2	24.8444	24.0152	22.0313	25.6563
CDC25A	27.0046	17.8313	22.1231	22.6112
ZNF589	4.00171	5.01504	4.06834	4.08914
PLXNB1	15.4611	18.782	21.0936	15.8345
CCDC51	37.1653	37.0903	36.9822	36.8219
ATRIP	13.1772	11.1245	12.0012	11.9662
SHISA5	68.8294	71.9505	79.8307	73.6028
SPRY1	0.979015	1.1027	1.12982	1.19945
APEH	223.669	216.422	228.634	218.503
INTU	3.43707	4.76675	4.04103	3.77822
RNF123	13.5743	15.3302	16.7051	14.4782
HSPA4L	9.16251	8.37066	8.49726	9.37397
MFSD8	4.39272	6.27445	6.09442	6.36915
C4orf29	2.7889	2.73969	3.20958	3.14243
MON1A	17.0882	11.3341	12.6475	11.6661
MST1R	23.0034	25.6964	34.426	26.3805
RAD54L2	4.39026	4.03246	4.34502	4.11553
TEX264	38.2583	38.3627	43.3576	37.2239
DUSP7	16.9917	13.1439	13.4189	12.9607
POC1A	20.374	19.4167	18.974	18.2012
PPM1M	5.7521	7.72314	7.55424	6.5823
WDR82	62.7908	55.2995	57.8361	60.7771

PITX2	4.29233	4.08555	4.29864	4.24779
C4orf3	26.5979	14.4961	14.2115	18.4028
PRSS12	5.51882	5.21022	6.62093	5.75022
HMGB2	269.457	189.577	164.995	198.454
SAP30	14.2267	9.19636	9.47492	10.6571
MAD2L1	112.449	88.1069	86.5721	107.614
ANXA5	198.479	184.803	161.981	168.722
FBXO8	5.43191	4.93499	5.04235	5.6923
CEP44	2.824	2.73555	2.29595	2.51387
TMEM144	3.96905	4.47292	3.96366	5.11232
NAA15	36.6565	30.3075	32.9371	37.6221
FAM160A1	2.3108	2.28745	1.97129	1.90286
ARFIP1	14.4147	12.2882	13.6306	15.3833
KIAA0947	10.8633	9.96747	10.0727	10.4688
ANAPC10	20.1124	20.3415	18.1161	20.0659
ABCE1	132.898	105.664	112.767	129.934
OTUD4	16.5273	11.9276	10.3104	11.6305
LSM6	65.2663	51.983	50.9348	57.4287
TMEM184C	14.6135	14.7687	15.2928	16.3043
PRMT10	3.52391	3.53984	4.07608	4.24074
ITGA2	5.96663	5.75511	4.31009	4.88377
MOCS2	31.0731	29.3107	28.9878	31.1637
TMEM161B	7.35952	7.6839	8.36176	8.371
ELOVL7	8.25189	7.46716	7.70043	8.26377
NDUFAF2	40.704	34.3771	33.9136	37.9646
NIPBL	8.60658	7.50485	5.91448	7.61386
SLC25A46	15.0574	11.9054	11.6025	13.7262
STARD4	8.58645	5.1497	4.65093	5.73274
PGGT1B	21.2944	16.4466	18.0936	18.0564

CCDC112	5.97106	4.65871	4.64598	5.54183
ANKRD33B	2.75417	2.06295	2.6263	2.37225
CMBL	36.8589	46.8411	43.1055	36.4028
C5orf63	2.94608	2.8628	2.89655	3.42171
PRRC1	22.4896	23.1533	25.8954	26.5775
F2RL1	82.7145	66.9445	71.1989	69.8945
AGGF1	7.07397	6.99527	7.84771	8.11743
WDR41	33.3076	33.5077	30.3179	35.921
NDUFS4	65.4689	59.1895	51.6666	61.5148
SCGB3A2	7.52279	7.35688	10.3935	10.303
GRPEL2	15.8519	12.313	11.9656	13.4042
ARSK	3.24586	2.88297	2.87647	3.01137
RHOBTB3	37.4969	40.4764	33.7312	36.1758
GPX8	24.98	24.7719	19.5139	23.8115
TIGD6	1.60102	1.9912	1.86349	2.01023
SERINC5	7.69354	7.5167	8.6087	7.56445
CASP3	9.68556	9.04289	8.40425	9.8594
CCDC111	5.69504	4.24189	4.46999	5.05413
ERAP1	1.54827	2.01755	2.57441	3.1842
KIAA1430	17.7273	19.7475	18.2324	20.4375
RICTOR	1.88591	2.18993	1.97961	2.36779
PAPD4	21.205	18.6738	20.5776	21.2965
ANKRA2	5.50148	8.55294	6.09446	6.15992
UBLCP1	23.3285	21.1158	16.6023	22.0135
UTP15	25.6401	20.676	21.2015	23.1662
NSA2	93.0664	84.3477	75.5873	84.7458
GFM2	19.8551	17.7132	16.2684	18.9303
TERT	4.39749	3.10821	3.52668	2.91758
CCDC127	2.91129	2.41463	2.37761	3.05238

8-Sep	13.7197	13.9369	17.3473	17.1885
SHROOM1	11.3688	7.41141	8.4665	8.09641
UQCRQ	409.028	418.227	376.482	419.662
SLC35A1	23.7186	18.6428	16.6576	18.2383
TLX3	3.81954	3.88688	4.54097	4.2845
CITED2	14.1775	14.4661	15.3057	15.3141
DCBLD1	10.982	10.0218	10.1837	10.4825
SFXN1	121.722	110.622	124.665	122.399
SAMD3	1.29284	1.88196	2.14151	2.07201
TMEM200A	7.813	7.57319	7.83938	8.48502
PDSS2	6.53159	6.02144	7.32496	8.10891
STXBP5	4.9243	4.38191	4.70973	5.49741
RAET1E	3.12458	2.3334	3.54353	3.16116
DAGLB	11.891	11.2347	12.3882	11.6637
KIAA0895	4.22187	4.28999	4.1109	4.23205
STK17A	11.9665	11.2213	10.1536	11.4412
TRA2A	101.367	79.2228	82.3842	85.7362
GALNT10	16.6533	12.7333	12.1221	17.0476
SAP30L	18.4418	15.7828	14.8201	13.2656
RPS14	1410.33	1398.99	1387.88	1487.75
COG5	9.14553	9.88623	10.4521	9.38067
C7orf60	1.41599	1.54345	1.6631	1.75649
SLU7	28.825	22.489	23.4207	26.9684
RP9	17.7898	14.183	14.6249	14.6237
PTTG1	155.502	136.697	110.829	129.618
CAMLG	15.2245	16.4387	13.9173	15.4641
RELL2	7.24852	9.92118	10.8531	9.76087
KCNK5	7.12903	5.6437	5.99183	5.93762
ZNF12	6.63813	6.90323	7.14504	6.84724

SLC29A4	7.95799	7.54218	11.1101	9.4551
STEAP1	17.2926	16.3079	15.0499	16.0922
CDCA7L	48.2552	45.4427	45.1758	50.1931
MIOS	10.7642	9.88514	9.95476	11.1603
KIAA1324L	5.43533	5.93162	6.76779	6.02516
USP49	1.08582	1.26734	1.15607	1.20394
SYTL3	59.6484	51.9567	57.5967	57.9643
ZNF704	3.99341	2.79672	2.89262	3.39066
FABP5	343.011	278.748	265.503	291.752
CHMP4C	5.658	6.60957	6.5714	6.98539
BRI3	65.9551	72.8278	65.507	70.3498
LMTK2	4.79179	4.66509	4.71561	4.79638
CTSB	166.772	183.173	177.848	176.447
SUN3	7.80782	6.63575	5.86097	5.65735
HNF4G	1.20719	1.03543	0.86095	1.16031
PEX2	43.2975	40.1368	35.4726	42.4377
RAD21	119.971	110.883	107.584	117.564
MED30	28.1061	26.0558	26.2513	24.4445
SPIDR	41.27	30.098	40.8592	36.0141
ORC5	24.6334	23.5866	22.7776	25.5349
HEATR2	63.2327	60.7734	56.0315	60.1987
OSGIN2	23.0142	18.7826	20.8373	24.4808
SUN1	79.0495	83.3372	89.0487	79.7992
OXR1	9.70761	9.87475	11.2154	10.569
TMEM184A	10.5805	8.7535	10.7245	9.54101
NOS3	27.8909	30.9889	35.7256	30.105
MICALL2	20.1497	19.7172	21.8316	18.1876
INTS1	78.4218	82.2266	89.1757	77.3114
CDK5	16.4109	16.5346	15.6331	15.4017

SLC4A2	119.879	99.4084	110.216	104.455
FASTK	57.5742	66.1035	67.3715	57.147
TMUB1	67.9946	63.4497	69.1507	63.6657
C7orf55	22.7065	20.4057	19.6331	23.9792
PHAX	19.6566	15.5771	16.0554	17.7301
ALDH7A1	61.1201	64.4029	64.2604	62.7163
FOXK1	12.5313	10.8907	11.1112	11.1837
COX6C	424.449	326.852	299.579	355.327
OSR2	4.04419	3.95421	3.86308	3.61212
YWHAZ	666.427	541.49	526.21	597.249
FZD6	19.7254	6.23745	8.80956	10.6789
CTHRC1	2.92951	4.12104	3.60008	3.76973
SLC25A32	42.4424	23.5431	23.1021	26.9722
DCAF13	131.298	117.97	120.52	135.951
TP53INP1	2.11069	4.11385	3.03936	2.86543
INTS8	63.7109	57.5469	60.3516	63.0051
KIAA1429	41.7301	35.9598	36.1131	37.2629
GEM	8.23285	5.31796	4.46538	5.51813
PDP1	345.656	359.871	357.772	365.659
TMEM67	13.575	15.5818	14.9364	13.0698
KIAA0196	26.1839	23.2178	26.4321	25.6216
RPP25L	53.3925	58.8348	57.8538	57.6174
FAM219A	6.521	6.58465	7.23105	7.35493
SNAPC3	14.7929	14.7087	14.9959	15.1225
KIAA1161	3.88954	3.4943	4.2466	4.67626
NUDT2	32.3442	33.8037	34.4156	35.3273
TMEM65	5.44498	4.73501	5.38125	5.91817
PSIP1	51.7194	46.1022	43.7978	50.0526
UBAP1	19.0887	13.5401	14.2028	15.8206

SYK	2.76332	2.28876	3.44022	2.78466
NFIL3	10.7106	9.70095	9.15839	9.92803
LETM2	21.046	16.1924	20.0195	21.992
METTL2B	32.3316	24.4805	25.2474	28.4784
FXN	36.2406	34.7963	32.7954	35.1176
KDM1B	12.0644	11.1609	12.1778	12.2131
HGSNAT	2.20796	3.79828	3.19118	3.13573
GKAP1	1.62622	1.54365	1.41959	1.57541
HNRNPK	481.819	404.165	395.994	443.873
ANKS6	20.8309	19.5338	21.479	19.5401
TMEM246	10.7819	11.217	11.6072	11.4395
ZHX1	8.31307	7.16264	8.33837	8.4167
DYNLT3	14.2103	14.1366	13.9084	14.6479
WBSCR27	5.47994	9.3896	8.11788	6.92247
MID1IP1	40.1692	39.5406	40.9269	39.2284
KIAA1958	1.98672	2.39411	2.27356	2.31335
PIGA	3.67086	3.62469	3.64794	3.79947
STRBP	15.247	13.961	13.9779	15.7991
CLDN3	3.52999	4.24218	4.38733	3.29405
GAPVD1	16.0957	14.016	15.3475	15.6824
C9orf89	46.8141	51.4569	54.6952	51.5694
WNK2	10.3055	8.63395	9.18451	8.97577
ZNF367	11.2763	9.39512	9.97932	10.6472
NDUFB6	78.5618	69.6836	65.4015	73.7814
NOL6	31.896	27.6273	29.6203	29.5899
AQP3	3.17758	3.46076	4.56253	4.47877
TRMT10B	2.7145	3.07619	3.29022	3.17369
VCP	165.471	148.165	160.798	157.352
PIGO	11.5547	12.0015	12.8247	12.5632

STOML2	244.502	219.541	231.837	226.097
BRWD3	1.81964	1.92372	2.13709	2.28547
MELK	47.8126	38.677	39.2181	42.94
OTUD1	1.27952	1.59294	1.4758	1.55399
ARHGAP12	24.6651	27.3821	26.1164	31.8489
HECTD2	2.98943	2.27921	2.23956	1.99503
FBXO33	7.33718	7.36747	6.65266	6.91578
ZNF488	1.86623	1.44919	1.78879	1.61813
SPTSSA	14.3968	12.026	13.0206	14.0046
WRN	5.3929	5.34785	5.36966	5.83894
8-Mar	7.39658	7.43262	6.36905	6.47742
CFL2	48.175	44.61	41.4263	47.6585
SUGT1	65.234	57.3202	53.5876	60.8939
GTF2A1	14.285	10.2898	11.6341	12.4667
ZCCHC24	2.14577	2.84784	3.53237	3.1293
SLC16A9	4.03879	4.77466	4.78884	5.26713
INPPL1	56.2362	59.5828	60.3188	53.8978
GJB2	14.4906	16.3076	15.871	15.9552
CRYL1	7.70108	9.85113	9.96544	9.33186
REEP3	8.01478	7.34265	7.3365	8.18582
SKA3	26.826	22.4559	18.1993	22.9743
MICU2	31.2061	29.5261	28.6069	30.4873
C11orf82	14.5694	12.1268	10.9513	12.5886
PCF11	13.5373	9.92522	11.2922	12.9421
LRR1	35.4456	26.0952	26.6129	28.5758
RPL36AL	317.761	253.772	252.12	273.66
DNAAF2	11.2904	9.98935	10.923	10.9228
ZNF22	4.742	4.3077	4.03165	5.0441
KLHDC2	11.1679	13.2886	11.4036	11.1257

EML5	1.45846	1.31989	1.26386	1.43404
NEMF	17.3493	18.1206	18.3717	20.6664
RPUSD4	46.3671	32.5907	36.0862	32.3944
ARF6	79.9461	72.754	75.6175	78.7492
TTC8	13.4945	19.2154	13.9685	16.4608
TMEM63C	2.83852	2.3988	2.94024	3.09117
CDX2	8.44965	9.6415	8.26641	9.36903
AKR1E2	1.69036	2.52034	2.54969	2.38321
KBTBD6	7.78671	5.0172	5.09319	5.5411
FAAH2	1.18001	1.55687	1.71182	1.77954
NUDT5	126.643	106.809	102.57	112.835
DACT1	1.87668	1.49433	1.4893	1.82104
BEND7	4.44752	5.15995	4.93343	6.40371
ATP5C1	260.634	256.406	250.19	262.68
PRPF18	11.348	10.0418	9.45974	11.0754
TAF3	3.57699	3.80553	3.62843	3.78731
VDAC2	215.687	205.366	199.788	212.089
COMTD1	32.3439	31.9382	34.7863	31.1788
SLC18A2	4.33452	4.78323	4.65078	4.959
PDZD8	20.2963	20.0543	19.0186	21.0675
ZNF503	18.8097	20.3368	22.1358	18.543
FAM175B	18.139	15.9756	15.1666	16.0351
QSOX2	21.0133	19.4551	20.7402	21.1797
FAM204A	44.0572	42.9406	35.7838	42.6307
NSD1	14.1749	12.7998	12.4586	12.8654
PRDX3	328.834	325.77	317.279	336.468
ENOX2	4.67222	4.73463	4.66352	4.33462
GHITM	160.277	164.452	160.316	162.188
SNAPC4	12.3586	11.2077	11.6701	10.7461

PMPCA	90.1503	87.0485	92.3995	89.7945
SDCCAG3	76.2297	74.9717	75.3942	74.3268
C9orf9	2.76878	3.5011	3.1548	3.13747
TSC1	6.1378	6.61488	5.59351	5.5505
HPRT1	65.0391	58.2879	56.9623	60.7119
LOH12CR1	4.88977	6.41155	5.83148	6.34047
FAM69B	8.6515	7.8076	8.94322	8.47367
ZMYND19	60.4144	49.6864	50.5878	50.0589
STOX1	1.17398	1.37484	1.28722	1.35106
RET	0.720929	1.15842	1.37009	1.05244
DDX21	97.7114	46.273	48.5887	62.3607
BMS1	48.6357	41.0578	40.549	44.8157
STK32C	16.1673	14.3254	14.4494	14.6354
KIAA1462	2.58874	3.38832	3.21516	3.26575
FUNDC2	18.5039	15.7904	15.8207	17.0746
TMEM55B	31.8036	39.0745	34.6092	33.7459
METTL17	56.0552	52.6736	54.3201	51.5718
NDRG2	10.7909	11.5179	12.4635	11.0439
ARHGEF40	2.20245	2.58652	2.45131	2.543
NSMF	76.3008	73.3564	74.4008	70.0479
ZNF219	8.99977	12.8324	11.9865	10.197
C12orf50	1.65785	1.7773	1.96028	1.95242
CASP7	24.1185	25.0043	26.3206	27.3467
BTNL9	2.37606	2.79797	2.50495	2.68643
C10orf118	2.69896	3.68054	2.5905	3.78123
METTL3	41.0623	34.5781	35.5451	36.9906
TRUB1	26.0818	24.0781	23.9772	25.6534
FAM194B	1.55427	1.20251	1.97397	1.50291
ZFYVE1	2.83092	2.7993	3.50084	3.02901

HSPA12A	3.29275	3.77358	3.43006	3.55766
UBTD1	11.7079	11.4556	12.5554	11.5333
E2F7	5.22316	4.75763	3.83061	4.16196
ARHGAP42	2.46094	2.5771	2.68493	2.95028
ISCA2	25.4047	26.1454	26.2154	25.8285
GYLTL1B	56.4013	65.9311	62.9172	57.4541
PACSIN3	44.1705	42.2422	41.01	39.0186
TTC7B	21.5056	23.0143	24.6351	23.0971
SLC39A13	13.0226	14.7879	15.5654	14.8547
PSMC3	200.209	173.352	178.816	181.521
TC2N	4.45076	3.77153	4.12452	4.44409
CPSF2	27.7634	25.0392	22.9757	25.3191
MOAP1	5.40309	5.97181	6.54042	6.57168
IFI27L1	16.7937	15.632	15.4274	15.2144
IFI27	1.53258	1.69233	1.26264	1.28192
CLMN	5.29637	5.08562	5.40353	5.29586
PTER	11.9596	8.24085	9.9693	11.425
PTPLA	9.12249	9.02012	8.4439	8.42409
ARL5B	7.28282	6.64484	5.46095	6.52738
SMCO4	28.2663	19.5616	19.5239	23.7675
KIAA1731	5.46843	4.31157	4.21857	4.67257
TAF1D	101.229	100.412	90.4204	101.863
ABTB2	3.47627	3.41261	3.58967	3.3689
R3HCC1L	5.32706	5.03577	4.90442	5.46846
AMOTL1	6.88731	4.79442	5.59345	5.84763
CEP57	10.202	14.1155	12.6194	13.8916
SPRED1	9.91706	8.01131	8.10323	8.34046
GPT2	22.1472	27.7897	24.1693	21.2817
AMN	0.717293	1.17587	1.34753	0.886401

RAB8B	5.71774	5.32355	3.30922	3.85822
IKBIP	9.08811	11	8.06893	8.32505
RPUSD2	18.215	15.4009	16.7971	15.749
HIF1AN	27.6979	26.4214	26.3273	27.3219
NDUFB8	377.038	325.576	316.865	356.52
ZFYVE19	13.9365	12.9214	13.408	13.6303
SPINT1	67.302	67.7842	78.0482	69.6961
DEPDC4	1.73127	2.0934	2.06533	1.86887
BRD7	68.383	61.3615	58.8171	64.7945
CKB	802.135	800.532	765.545	714.806
TRMT61A	22.9446	20.9416	21.6568	19.5764
BTRC	9.4918	7.82282	8.78963	9.44462
POLL	19.8611	22.9311	23.1539	21.3714
BAG5	13.563	12.0471	12.4496	14.222
DPCD	48.1891	43.7508	43.026	42.3447
LARP6	26.1027	17.7254	12.1008	12.6479
API5	70.7518	61.2278	58.124	70.1501
ZNF319	3.66829	3.64622	3.61279	3.85081
HPS6	28.0043	26.417	30.5229	27.582
SENP8	3.30796	3.30974	3.19853	3.2934
NOLC1	196.584	150.43	156.403	165.523
ALKBH3	28.7332	23.4092	25.6053	20.8632
COPS2	62.6445	55.5392	51.3649	60.5454
SGPL1	24.578	19.8215	24.2989	26.2619
FRS2	5.43329	4.68294	4.57344	5.00469
CCT2	359.509	254.012	268.143	295.171
PCBD1	139.408	153.348	140.064	151.535
ARIH1	29.0194	30.5197	29.8346	30.497
CLMP	1.31962	0.844872	1.08201	0.667008

COX11	38.1067	38.3344	34.653	35.9607
ZNF202	9.53703	9.24592	9.01566	9.79114
FAM227B	4.93211	5.63046	4.37141	5.88225
STXBP4	3.9637	3.34928	3.82876	3.79841
CUL5	11.6634	10.9172	12.7235	10.8253
WBP1L	10.5369	12.4599	13.3762	13.2692
C10orf32	10.7688	14.5873	12.6683	12.7141
PLEKHF1	4.02737	4.28779	5.11111	3.97377
ANAPC16	56.8709	61.2404	56.8552	59.0739
SMPD1	3.76675	3.99944	4.57397	4.34787
APBB1	6.44568	5.85549	6.6083	6.53679
NUDT13	1.06407	1.08388	1.48155	1.94696
C11orf65	1.25611	1.07153	0.974376	0.846907
TRIM44	11.1858	11.7492	11.781	11.8716
ILK	40.3964	40.9015	44.0377	42.2508
TAF10	209.584	197.714	203.97	208.368
TPP1	15.9387	14.1023	14.379	16.4424
CYB5A	19.5717	16.2292	16.4678	17.347
USP54	10.2107	9.02783	8.55427	8.80269
C11orf74	8.2833	10.5638	8.1272	9.66295
ATP9B	8.63942	9.28179	9.90247	9.24122
PPFIBP2	1.98804	2.05958	2.42417	1.58338
CYB5R2	11.3984	12.2692	13.5517	14.5598
KIAA0355	3.3839	4.09263	3.80743	3.96977
SERPINB8	2.79722	3.02022	2.332	2.38349
IDH3A	93.3133	76.5697	83.7153	93.9889
WDR72	2.13078	2.26402	2.44915	2.29327
XRRA1	5.89605	6.11483	5.90144	5.7634
TRIM66	1.9013	3.19526	2.91122	2.4102

RNF169	3.52144	4.30201	3.78257	3.51286
RPL27A	2667.01	2759.69	2682.06	2715.55
ST5	6.58259	6.37599	5.57551	7.19027
CENPN	98.5705	71.8227	65.4839	84.8955
AKIP1	35.2496	30.5723	32.0915	30.7318
ATMIN	36.0118	29.3236	27.8997	31.5686
C16orf46	2.93195	2.39205	2.47101	2.87926
TMEM41B	17.128	11.5552	11.6381	12.8598
PKD1L2	0.845399	1.04454	1.20163	1.04943
LEO1	18.8859	16.3294	16.1393	17.0763
ZNF143	27.8254	20.8587	22.3361	20.5063
TMX3	22.2656	12.8533	13.2841	14.913
WEE1	32.6394	32.257	33.6707	33.6719
MAPK7	7.453	6.51676	6.57667	5.88087
NDST2	11.193	11.5801	13.0854	10.6035
MCM7	347.212	285.47	302.043	294.434
CCDC68	1.73531	1.65076	1.9276	1.95532
ZNF3	17.5366	16.3187	15.8283	16.2631
ZSCAN21	5.1282	5.17956	4.17379	4.3012
TK2	5.99421	6.48848	7.12985	6.83861
TMED3	59.2109	38.6018	53.9143	53.4008
SEC11C	83.3314	80.4883	73.3954	84.6308
TMEM135	18.2841	16.2522	16.8918	16.1919
IQCD	1.1018	1.34125	1.20489	1.26388
NDEL1	20.7821	17.5977	19.2923	19.5022
CENPV	90.6271	71.6177	65.2929	72.5812
RRAD	1.56154	1.31542	1.59401	1.44186
FAM96B	191.397	175.613	182.517	170.592
HSP90B1	461.57	410.092	426.193	475.813

BLCAP	30.4481	37.3393	40.7429	37.4195
CHRFAM7A	2.07453	1.54243	1.322	1.68957
ATF7IP2	2.85719	3.45299	3.03732	3.38912
TVP23A	2.03147	2.00154	1.74416	1.94749
NGFRAP1	80.7933	88.6705	83.9177	87.6095
COG1	17.2683	15.7972	16.6014	15.8254
PLEKHA7	1.98777	1.88194	2.187	2.26592
B2M	481.891	472.663	469.878	496.823
ZNF592	9.61276	8.90364	9.48878	9.75079
CASC4	29.3803	28.1296	23.9469	29.9591
AP1G1	30.377	28.8684	30.2332	32.9611
SLFN5	1.32765	2.5346	2.12461	2.10242
CATSPER2	1.59844	2.68137	1.90183	1.89397
C16orf45	11.0255	11.115	13.8279	13.1855
KIAA0430	5.47178	6.7698	6.64834	5.87737
SAAL1	37.8512	30.7815	31.829	37.1891
PPIB	464.692	412.779	394.885	439.351
FAM96A	83.5602	80.1076	81.201	91.4772
FAM111A	9.59136	8.84681	8.47435	9.03182
KIAA0101	100.279	88.3309	87.6015	94.2551
KIF7	1.63994	1.25589	1.39488	1.03895
LDHD	0.593325	1.01671	1.00884	0.843121
PEX11A	4.94328	3.86051	4.26047	4.16762
TMEM170A	12.7545	11.7248	9.79089	13.7412
MESP1	10.4772	10.9034	11.1551	9.93928
RBPMS2	7.44062	8.51589	9.85287	9.47903
ANKDD1A	2.49643	2.70982	3.19726	3.85232
C18orf54	3.49191	3.44582	2.36766	2.96613
DCTN5	23.8516	17.1593	19.0116	20.4953

TERF2IP	27.6457	26.0941	26.8663	26.8283
PLK1	151.254	118.325	118.483	118.087
CLPX	32.9242	30.1829	30.71	32.0436
ZBTB39	5.77003	5.62602	5.26025	5.61053
TMEM194A	9.18586	7.68092	7.2988	7.87552
NAB2	31.0334	28.2301	30.7376	29.5878
VPS39	9.59072	9.88082	10.9717	11.3986
STAT6	26.1484	32.4472	33.5723	31.5535
PATL1	20.824	16.6912	15.4909	16.2856
XRCC6BP1	6.22944	6.14692	5.67419	5.89218
ELFN2	12.1117	12.968	13.8458	13.6812
STX3	22.1561	20.3022	21.3604	21.8064
MRPL16	76.7657	67.5673	69.5566	71.2291
PIP4K2C	28.5023	27.8391	28.2989	29.0209
MTMR10	6.33573	5.45467	5.64366	6.453
YWHAB	110.916	80.0634	79.4254	89.8396
NYAP1	2.01073	2.17223	2.30179	1.89991
TSC22D4	16.438	16.0845	17.5124	16.4251
DIS3L	17.2577	17.2562	18.7019	15.4626
CCNDBP1	15.3149	10.5655	14.4034	15.1842
SMAD3	25.7695	23.106	27.7113	28.9394
RCCD1	23.9458	21.8529	21.8604	22.0451
AKTIP	12.9366	17.6948	16.1774	14.9034
MAPRE2	14.221	14.8207	13.4175	11.3391
EVA1C	26.6456	26.4339	25.7243	27.6014
MARS	188.699	232.841	193.977	186.308
MBD6	9.19806	9.18887	9.12043	7.5563
CNPY4	2.25566	2.24809	2.33287	2.28293
PDIA3	423.052	395.105	412.359	393.024

NUDT21	94.5383	86.0362	89.5256	101.944
NKX3-1	3.6088	2.57967	2.53859	3.41147
SGSM1	1.24877	2.02511	1.65588	1.90524
DUSP18	4.08568	3.9385	4.50942	3.99538
TEF	5.27916	4.45914	4.41418	4.3145
PBX3	5.06294	3.80651	4.78879	4.63938
PHB	810.988	715.862	747.906	719.923
SNRPD1	301.829	226.466	225.5	264.586
SAMD14	2.83823	2.58778	2.9928	2.93608
PIP5KL1	6.23986	12.0468	8.59431	6.76235
TMEM92	3.75046	4.08685	4.82413	4.57046
FAM102A	14.0425	17.6182	16.0879	15.8765
ACSF2	11.9213	15.2367	15.3474	11.4977
GOLGA2	20.6606	19.5032	15.7009	13.8052
TRUB2	40.372	43.8704	42.45	42.8603
COQ4	25.9819	29.4854	28.568	27.1565
SLC27A4	33.2047	34.1861	35.1347	32.736
URM1	61.7981	51.5942	56.2965	57.9885
CERCAM	12.4701	16.5981	17.605	15.7884
DOLPP1	21.8209	15.7988	17.5973	16.6138
CCDC103	0.990547	1.29477	1.49157	1.35356
ENDOG	14.3339	14.4383	15.079	14.1045
PRRX2	18.3214	18.9663	17.2355	17.6759
C15orf39	15.3054	11.9635	13.7596	13.2333
SP2	8.87714	8.45844	9.01485	8.85447
COQ7	19.0011	17.4768	15.6047	18.2403
GPRC5B	5.42908	4.09602	4.77874	4.48916
CRK	42.6208	40.016	43.4133	44.3798
FBXO22	72.941	71.9	73.0828	76.8947

TBC1D2B	6.75896	6.59834	6.15292	6.09985
NOD2	1.78677	1.64908	1.64795	1.63697
HDHD2	13.924	15.3457	14.2699	15.8646
ZNF91	2.35224	3.09167	3.08425	3.0609
IGF2	3.153	3.36785	4.35563	4.06711
RNF214	13.392	12.9873	13.128	15.333
CDK12	18.4354	16.8603	16.2078	16.5029
DUS2L	18.6093	18.8886	17.2954	16.5798
POP5	67.9605	46.2913	35.0664	33.8503
ENGASE	17.0817	15.5144	16.6828	15.8296
ATP5L	256.271	225.372	204.877	213.893
TBC1D16	10.5349	10.1366	10.8279	10.5611
ENTHD2	13.01	14.3807	14.1327	11.8152
MYO5B	15.382	15.4408	16.6304	16.879
ART5	1.95866	1.88426	1.92283	1.70238
ACAA2	8.72305	10.6183	10.7762	11.2233
STIM1	11.6483	11.0112	12.9507	12.0403
RRM1	107.38	92.1989	89.8552	94.081
TRIM68	3.87819	3.66246	4.11877	4.09674
AC104389.28	86.2008	87.9009	83.7928	91.2146
OR51I1	1.37204	0.874145	0.868166	0.825657
FN3K	2.87393	3.02454	2.58124	2.32127
ZNF23	4.67123	4.57593	4.04621	4.45047
IRGQ	5.8586	6.46677	7.92622	7.30967
ZNF226	7.18887	8.98501	9.51419	9.64277
ZNF180	4.46983	3.4007	3.92098	4.25197
PPP2R3B	4.25531	2.78411	3.01484	2.69436
ZNF668	12.2655	10.8831	13.2486	12.0032
ZNF646	5.00376	4.84112	5.40565	4.80763

VKORC1	96.4864	99.8089	102.964	96.1831
SMG8	20.354	14.7862	15.1842	17.038
TPM4	169.008	160.792	152.383	163.068
RAB8A	83.4222	75.368	68.9122	97.2337
GPX4	340.545	348.115	344.322	339.236
MIDN	41.0194	39.2057	42.1366	39.6799
KLHL26	9.06767	8.37124	8.69651	7.7619
GATAD2A	73.3934	48.6738	63.7606	64.1322
MVD	74.6341	55.3052	62.315	55.0619
CDT1	95.3308	86.1914	95.9572	86.0798
TRAPPC2L	134.228	118.842	118.272	114.357
ANKRD11	60.6869	69.9917	68.6985	60.0129
SPATA33	15.8892	15.334	14.8155	14.5137
SGK494	5.41214	4.16703	4.98637	4.54402
RPL13	2951.95	3182.56	3258.28	2991.57
ZNF641	2.53441	3.22477	3.1216	3.2063
CACNB3	11.7021	11.0619	10.3914	10.8594
DHRS13	8.24878	7.68225	7.64569	7.80016
TP53I13	33.1911	36.1483	38.3555	34.6829
KMT2D	19.5873	19.1562	22.0087	18.5304
CORO6	2.90776	2.82537	3.47701	3.10695
RHEBL1	1.66981	2.0264	1.51924	1.55132
TUBA1A	151.411	101.383	120.326	124.895
TUBA1C	870.859	845.556	634.121	675.655
ZNF701	1.32657	1.4774	1.4959	1.50923
SERTAD3	15.2564	16.0225	15.5678	15.3578
NCKAP5L	3.13055	4.05466	4.40794	4.20386
RAB4B	11.2309	12.5034	15.5711	13.9243
GPD1	1.21404	1.85878	1.84353	1.60504

C19orf55	11.055	10.8752	10.7556	8.19347
CYP2S1	10.5373	11.1837	11.1111	11.2731
AXL	37.0342	33.7543	39.7671	38.3576
NFKBID	5.78957	4.62587	5.44443	5.36201
TMC4	2.34193	3.64677	3.01785	2.3748
LENG8	25.5335	18.5808	21.4671	24.1746
TMEM145	2.68607	4.01518	6.74356	4.21979
ZNF526	4.86759	4.98169	5.47048	4.94374
TRAPPC9	7.5554	9.17201	11.3663	8.30507
ZNF146	63.8622	54.2832	52.8375	59.5613
ZNF283	3.80373	1.87037	2.49192	2.18492
SPINT2	471.355	427.814	487.094	464.687
C19orf33	196.161	198.366	199.097	197.168
YIF1B	57.8158	60.813	63.3366	56.6981
DNAAF3	30.4652	24.7255	28.5136	29.4532
DAPK3	43.9485	36.5722	42.6302	40.0636
EEF2	2319.58	2482	2484.26	2257.66
CHAF1A	43.5455	35.2094	37.7079	39.658
UBXN6	48.4551	50.274	54.0877	45.711
HDGFRP2	51.8339	50.5418	49.2385	47.773
SEMA6B	34.1447	37.1575	43.5229	39.5086
ZNF444	20.7824	20.099	23.3809	18.7467
NXN	76.2744	76.943	76.838	75.7651
FAM57A	50.7876	42.3297	45.4202	45.9687
GLOD4	76.8747	67.4406	65.7869	79.2157
MFSD3	17.814	26.2334	26.5928	22.0383
KIFC2	27.6894	32.3367	36.5526	28.8292
SLC43A2	10.1933	12.3026	11.9782	10.9422
RILP	8.74182	8.59721	9.33415	8.37396

WDR81	9.43172	10.736	11.1128	9.54928
SRR	5.34348	4.95381	5.6855	6.05221
TSR1	76.8188	66.7911	67.2393	81.7206
HSD11B1L	2.31951	2.79747	2.78646	2.62756
CYB5D2	6.8102	8.36843	8.2132	7.22931
C19orf48	228.003	206.839	220.922	201.772
KLK1	0.854785	1.09422	1.18261	1.30894
KLK6	33.1352	37.5732	41.5513	39.674
ZNF83	11.9168	10.1587	8.46561	10.261
KRT80	5.56866	5.89424	5.37363	5.41248
OTUB1	84.4017	79.8079	86.7689	84.6468
RCOR2	4.17816	3.84408	3.60293	3.58643
ANGPTL4	3.71939	3.8014	3.85398	4.20122
NDUFA7	1.63572	0	1.28446	0
CD320	103.722	83.6533	89.1146	82.4912
SPRYD3	39.5266	40.7952	43.9037	43.8981
IGFBP6	63.1655	62.9358	69.005	65.7371
ZNF558	7.57162	6.3382	6.80822	7.83861
NDUFV1	141.295	138.586	148.196	136.342
CDK2AP2	50.7353	45.1965	50.8476	48.9095
NUDT8	7.22259	9.90696	10.3356	9.72058
CTD-2369P2.10	0.981133	0.885772	1.08483	1.04962
PRDX2	258.664	185.759	155.133	166.283
ZNF232	6.99698	5.85997	5.17331	5.2341
MIS12	10.7058	9.80501	8.9329	11.3614
HID1	10.5162	10.4548	11.432	12.2453
ICT1	70.7043	64.2153	62.2215	67.0048
ATP5H	292.489	315.711	292.318	317.293
EVPL	10.47	12.9451	14.6645	11.7497

SRP68	117.121	104.278	107.032	108.905
MGAT5B	1.46066	2.21036	2.46757	2.15448
TMC8	2.27997	3.2497	4.20035	4.13724
TK1	280.049	262.466	260.202	261.475
TMEM68	14.6954	12.3795	11.2164	13.4338
TMEM99	43.6095	42.9553	40.7494	40.561
GHDC	7.58946	7.56721	7.54308	7.11562
ITFG3	12.3849	16.475	16.8584	14.7582
ZNF598	47.7702	41.6297	43.6278	36.6062
RAB26	8.46571	6.83529	8.01843	7.96148
MLST8	56.86	57.5964	60.3826	54.278
E4F1	29.1811	25.891	28.6915	23.2902
ECI1	107.927	116.024	113.792	105.4
AC009065.1	161.145	154.256	159.462	163.705
CASKIN1	9.63423	7.85102	10.0538	9.66003
ABCA3	0.860514	1.18843	1.23283	1.13493
KCTD5	47.0106	36.8606	42.2556	40.8496
SRRM2	224.247	216.696	221.419	193.334
ZNF597	1.92104	1.82584	1.75435	1.81203
SDHAF2	38.1205	31.0249	35.4663	36.4232
DDB1	150.663	150.444	151.058	152.682
VPS37C	11.8481	12.4064	12.8875	12.831
RAB3IL1	16.4465	12.5214	14.4701	13.2652
BEST1	17.2656	16.3433	16.5152	16.4759
FTH1	3982.88	3935.21	4221.89	3892.32
BSCL2	41.8839	40.6893	35.866	33.8756
POLR2G	141.266	123.84	128.985	131.66
SLC3A2	342.312	291.713	277.212	276.161
C11orf84	42.7405	35.9061	35.9816	34.7947

ATG16L2	2.5452	3.49733	3.78497	2.59239
C2CD3	17.4042	14.4854	16.644	17.428
TTC21A	0.697156	1.14038	0.778189	0.823121
RPSA	2993.21	3054.03	3093.75	2996.79
CTNNB1	172.741	168.929	150.396	155.987
ULK4	1.6139	1.27455	1.36187	1.46387
FADD	10.4186	10.346	11.3194	10.4357
LTBP3	108.04	94.8377	111.915	98.3267
SAC3D1	41.1273	38.2654	40.4027	36.7729
SF1	204.023	180.25	191.062	189.932
MAP4K2	30.8933	33.4943	33.5977	33.3745
CCDC88B	9.33478	9.90494	9.70936	9.46863
SCARA3	33.6174	32.7844	33.8477	33.0432
PBK	85.9943	65.9563	61.3443	71.9618
COPS6	121.237	114.521	117.55	115.211
PAFAH1B2	55.5933	28.2575	37.524	44.0153
ANKS3	11.0257	10.0356	11.2811	9.79756
NUDT16L1	25.335	26.0406	25.2507	23.269
KIAA1586	5.59174	5.0609	5.66684	5.52813
RAB4A	49.7958	48.3108	49.5991	48.4277
SETD5	57.7718	62.2543	69.7973	62.2258
VASN	3.79636	3.6757	3.82451	3.34282
FAM83B	1.60369	1.53381	1.63355	1.6535
THAP9	2.03174	2.00873	1.71954	2.00969
RNF187	192.788	195.588	201.89	201.766
HOOK3	3.45762	3.36902	4.00918	3.63117
MAPK1IP1L	69.6149	57.9249	69.8384	68.9548
DDIT4	16.3526	46.911	23.012	19.3405
RBPJ	17.3203	15.4584	15.6227	16.3014

LMBRD1	4.20005	3.29272	3.61157	4.00091
ZCCHC4	7.16238	6.31801	5.86828	7.06742
TTC39C	9.0673	9.79572	9.75077	11.0487
GLYCTK	4.16598	4.94816	5.2154	4.47415
GNG4	4.48968	4.98936	5.21247	5.37396
UBTD2	9.28367	7.42225	8.25429	9.14232
POLR2J3	54.3181	53.1225	50.6502	55.0513
NKIRAS2	13.1389	14.3511	14.6	13.5779
DNAJC7	77.994	65.4812	57.7894	61.2434
IRF2BP2	13.4108	8.6949	8.90428	9.46081
NT5DC2	130.059	124.081	127.438	121.409
SMIM4	39.8885	32.0226	25.8714	32.1343
COA6	37.6286	30.2694	28.635	34.5586
KIF5C	0.973757	1.18322	0.991617	1.08205
MGAT2	1.90144	1.5051	2.24027	2.19163
BMI1	71.0629	58.4145	53.2907	56.0979
THAP11	22.3227	20.7539	22.2164	21.3564
MMADHC	111.143	105.284	100.436	108.004
PDHB	53.4998	49.0172	51.2559	53.3591
PXK	5.462	4.6889	4.3294	4.76298
PCMTD1	1.80506	2.70793	2.58751	2.83469
KCTD6	8.53533	7.48962	6.8828	7.67414
MPLKIP	32.6145	33.9268	34.9275	35.4131
ACOX2	7.05077	10.3308	9.93559	8.21468
IRF2	3.23031	2.67152	4.20262	3.21963
ARF4	160.395	141.847	147.278	159.39
2-Sep	181.49	184.307	167.472	191.905
MFSD2A	9.94079	7.77302	9.44506	8.8689
DTYMK	73.6191	64.342	62.1606	63.8544

TAP1	5.26142	7.10352	5.50565	4.59122
ING5	16.7274	15.9868	15.9353	17.877
ATG4B	74.0708	71.0805	75.6965	69.5213
MLKL	5.9133	4.37704	4.88043	5.182
RFWD3	34.6107	26.6695	31.088	34.3552
COG7	9.28358	8.98293	10.8736	10.7084
CDC40	17.9659	13.0966	16.9822	16.0613
STIP1	335.247	283.698	275.134	286.331
PPT2	1.7788	2.28067	3.71979	3.09668
HR	9.29056	10.3715	9.94084	9.64997
RAB31	16.8253	18.5047	17.1874	18.1479
ATF6B	4.36471	6.17767	6.11805	6.22218
REEP4	50.2437	49.1794	48.7371	46.1185
LGI3	0.969804	0.898462	1.16549	1.10254
BMP1	12.9446	16.3924	17.9093	16.171
ATXN2L	90.4309	87.0158	89.6837	83.3049
POLR3D	22.8346	20.5194	22.1717	21.6454
FEN1	115.796	80.2801	92.1047	96.445
SOGA2	9.00443	7.46641	8.34212	8.95611
GBX2	4.11992	3.16429	3.15715	3.34904
HEXIM2	4.06995	2.8859	2.75065	2.92351
FNTA	40.7164	41.1363	41.2028	41.2708
SERINC2	26.1678	27.3442	32.4533	27.7116
TRAPPC11	6.90905	5.43097	5.50767	6.48015
ING2	4.31154	3.37185	3.79076	3.66665
CDKN2AIP	5.1034	4.44865	4.25833	4.09813
SNRNP48	11.9142	9.40941	10.4017	11.4759
TMEM223	16.7531	18.1177	19.3636	17.7866
SLC20A2	24.3548	20.3025	19.3533	21.4312

TMUB2	11.6973	11.6363	11.2569	11.3981
STAT3	15.3721	17.7653	18.4513	18.1957
ZSWIM1	6.21158	5.36522	4.4344	4.25994
NBPF9	4.91537	5.75184	4.94988	4.08275
ADAM9	70.6607	58.5893	56.5571	61.0566
AXIN2	6.95761	10.9983	8.31831	8.45874
NDUFS5	430.648	410.507	400.401	444.609
ZNF30	1.7308	1.33637	1.15081	1.50898
FAM84B	6.54608	5.94765	5.86185	6.14823
TMEM208	66.2571	59.5527	61.8308	59.5804
AHCYL1	39.061	36.3889	33.5754	35.6367
DNAJC21	23.926	18.7442	19.6375	21.8131
PKIG	20.7528	15.3176	18.7577	18.106
NPNT	3.91374	3.62151	3.32211	3.5325
SEMA4C	11.2043	12.1129	13.0549	11.7087
CNNM3	20.358	22.3253	22.5754	23.8564
GSTM4	7.14625	7.92096	8.94129	9.085
TET2	1.60773	1.49698	1.36048	1.43618
TCTN2	5.48161	4.81131	4.37478	4.6034
SHOX2	2.42125	1.89154	2.14255	2.24392
PPIP5K1	11.2867	12.2006	11.7565	9.16847
TSPAN5	43.5321	39.8208	37.2656	42.3927
ABHD15	3.41399	4.38603	4.79549	4.60211
ZBTB5	11.3277	9.25303	10.5458	10.3588
CHTF8	108.352	88.0973	100.465	102.029
ADAL	1.64164	1.60452	1.65676	2.24354
LCMT2	4.54833	4.69736	4.34934	4.85323
SNTB2	9.1345	6.95781	8.16545	7.86503
IL12A	2.28926	1.88483	2.48141	2.13225

ZNF507	7.82329	6.9169	6.94859	7.36936
STX18	25.998	24.3028	22.4912	23.0393
ZBTB49	2.06813	2.29906	2.14639	2.04562
GFM1	25.2424	22.5133	25.754	28.0067
TPTE2P5	0.232513	2.06029	0.723657	1.0567
DDX19A	37.6865	35.1756	36.4575	38.6448
ANKRD49	4.91979	4.00644	4.49457	4.679
USP39	86.6548	77.8683	75.8943	79.5125
TNIP2	35.0606	31.1687	32.54	33.2495
C2orf68	7.58062	7.63127	8.67398	9.60606
TMEM150A	6.40905	7.27407	8.95345	7.76776
RNF181	87.2674	90.5574	91.9	94.805
LRRC28	11.6737	13.6723	14.4606	12.5098
MAT2A	152.282	130.258	134.492	144.082
ENHO	4.41169	3.6826	4.42684	4.60497
ZNF608	0.808186	1.53638	1.04876	0.97368
SLC35G2	2.46212	1.64112	1.96694	1.85083
INPP5D	0.313106	1.68007	0.787282	0.761851
LETM1	57.7856	44.8025	50.7704	50.6191
TMEM129	14.8043	19.4188	17.8618	15.5349
PPIC	13.6357	17.3958	18.7891	17.1023
CEP120	4.37149	5.58388	4.79141	5.20178
MFF	55.0287	57.4139	51.8129	58.1112
JMJD7-PLA2G4B	3.49724	2.65034	3.31815	2.29377
CPLX1	7.61476	10.4323	13.0943	10.7595
PXDC1	5.43724	4.53729	5.47493	5.43908
E2F6	22.8695	19.1785	17.6903	19.0599
FEM1B	13.6971	14.6905	14.2707	13.9087
COMMD8	21.3545	21.608	20.6674	24.8384

ATP5I	731.812	656.139	636.918	657.102
UQCRFS1	132.9	118.478	125.708	129.297
MFSD7	1.68419	2.79506	2.418	2.05219
MAP2K1	43.3301	35.8311	35.8055	42.2592
KLK7	9.10995	10.8583	10.2823	10.5634
HNRNPH1	666.299	564.91	558.989	548.875
IRS1	25.573	25.5361	29.2988	28.0229
MECP2	4.90902	3.98028	4.57905	5.01284
UPF3A	12.4425	13.5126	13.1313	13.4059
ROR2	7.36736	6.98748	7.73811	7.53377
DHRSX	4.75449	5.77917	6.02632	5.42272
HSPBAP1	3.8593	4.172	3.80263	3.59123
ASMTL	22.5166	25.0511	26.4638	24.0069
SLC25A6	251.792	276.529	267.442	256.639
CHST14	9.63364	10.1456	10.1821	9.54671
PARM1	4.97445	4.66706	4.78441	5.38553
CSNK1G1	5.7758	4.90543	4.60948	5.06293
ARMC4	4.46505	4.64509	4.61901	4.51926
ZNF354A	7.26908	7.34923	6.83437	7.31741
ATF5	25.4573	26.2332	29.7727	27.2651
UBE2V2	77.2776	65.7088	70.5258	73.257
ZBTB43	13.7771	7.95035	6.58613	9.22377
CPT1C	4.76656	5.42376	4.91014	3.64682
PCSK9	9.45978	8.18883	9.11864	9.1494
XPO6	168.983	139.781	156.558	156.098
APEX2	28.6917	26.0798	27.046	27.2379
NSMCE1	62.3089	59.9981	58.7055	60.3963
CCDC126	3.42614	2.85986	3.16598	3.2815
RP11-231C14.4	3.79821	4.42584	4.19685	6.54734

RAB3B	4.98459	4.27311	4.99174	5.19195
CD2BP2	37.4904	34.3697	36.6657	37.7845
RGS14	9.47988	10.8222	11.4465	11.9492
TBC1D10B	31.5964	27.5779	31.2861	28.7437
LMAN2	171.958	167.315	178.462	172.151
RAB24	8.35402	7.59081	7.91347	7.62024
PRELID1	362.559	304.081	320.305	333.928
THBS3	0.974291	2.30042	2.31984	2.28635
CA5B	1.02311	0.985381	0.995374	0.905497
SLC50A1	35.6864	31.1368	39.5108	38.5366
EFNA1	10.6395	10.418	10.5181	10.4906
NPIPB3	29.27	28.9116	31.0815	26.1183
SH3TC2	3.84456	5.37012	5.30467	5.26563
ZRSR2	3.35338	3.19644	3.76524	3.9451
NMD3	59.3051	48.8897	54.2683	57.5775
B3GALNT1	7.99086	6.67535	6.5991	7.68727
GPRIN1	12.9587	13.5467	13.9204	13.3032
MRPL1	63.45	64.0533	52.258	67.8132
PGM2	25.5341	20.3624	21.5181	24.8819
SLC33A1	9.91783	11.9179	9.81878	13.1141
SNUPN	16.7048	16.2763	16.8921	17.4935
CRADD	10.9595	9.73713	10.5297	10.5349
SIN3A	18.9589	19.3336	19.8731	19.2743
ARL13B	4.46989	3.24189	3.78762	3.96127
PTK2	86.8031	87.8061	95.3	89.0186
PTPN9	11.1838	8.28332	9.48572	10.2108
KCNK9	1.26166	1.36505	1.04414	1.13585
MMGT1	6.62101	6.07055	5.62616	6.63747
TM2D2	40.3291	37.5302	45.7469	38.8523

PLEKHA2	4.86139	3.85478	3.61559	3.74928
CLIC4	20.0405	29.569	22.4822	24.7659
METTL15	9.17494	9.24611	10.8198	11.0393
PCBP1	221.689	193.013	213.419	210.417
HINT1	308.474	255.044	243.392	288.452
DTWD2	2.23269	2.48745	2.23588	2.46049
CLIC3	3.17914	4.18866	5.13504	4.98271
INO80E	76.1219	71.4191	77.0133	79.3437
DFFB	9.87021	7.18038	7.11623	8.92399
NFU1	29.0247	28.1828	25.6503	34.9927
ANTXR1	8.34255	8.97202	8.27054	9.14381
CKAP2L	7.30806	5.3605	4.79475	6.79126
C15orf40	32.1657	31.0863	24.7739	27.3073
FAM103A1	18.6693	18.89	20.401	20.9399
BOLA2B	347.346	287.964	266.612	289.106
RGPD8	1.97389	2.16037	2.10683	2.36256
HIC2	2.84108	2.60609	2.82599	2.69108
LUZP1	7.75517	6.45472	6.70941	7.24929
HEXDC	13.8166	15.8385	16.7709	13.9082
BCRP2	0.998516	0.607568	1.11821	1.09286
BUB1	46.1581	37.8748	36.9558	41.2028
SPNS1	28.7645	27.5489	30.3141	26.3841
LRRC45	25.7309	21.7166	23.5607	22.8067
CHRNA5	17.124	13.2278	12.4214	14.2751
STRA13	261.837	202.617	210.143	211.003
AGPAT2	59.4525	57.4502	63.3768	57.1426
ASPSCR1	27.2569	29.0026	34.1803	28.4495
FASN	364.606	313.358	329.055	290.741
CNBP	287.951	252.455	237.425	262.207

MT1E	686.447	887.065	856.139	777.68
DUS1L	186.391	197.895	198.661	191.83
GPS1	136.174	132.541	138.126	127.368
RFNG	36.9834	40.4234	42.6869	41.9401
DCXR	306.039	295.98	309.057	297.731
ZNF32	7.52004	7.862	7.30419	7.57808
RAC3	254.798	189.858	206.837	197.762
NRG4	2.48831	0.961922	0.789274	0.77263
LIMS1	45.9004	41.76	37.7918	41.6787
TAPT1	4.90652	3.73323	4.71097	4.76883
UGP2	19.9465	19.3082	20.5224	21.6105
LINGO1	12.072	11.1055	12.806	12.0543
HNRNPF	239.62	201.55	216.545	221.998
BTD	4.68759	4.29588	3.86565	4.28399
CSGALNACT2	5.18795	4.69129	4.06652	4.72443
PCDH7	23.2527	23.9078	23.5293	27.0342
AVEN	10.3087	10.4773	8.96476	10.1628
TRIM56	4.47283	4.56505	4.95903	5.50927
WNT10B	10.9837	8.48787	10.1087	9.72822
MUC3A	9.74272	10.5049	13.1823	11.5932
SYAP1	15.5588	14.1517	15.0283	16.3786
PYDC1	1.35921	1.34582	1.39193	1.25906
TPST1	9.98886	6.33855	7.90745	9.32938
TOR1AIP2	35.942	24.5845	27.7862	30.0246
OTUD3	5.80214	5.96947	6.27597	6.59508
OTUD7A	1.0245	1.20576	0.998295	1.06918
GUSB	18.6343	18.284	18.5629	18.6725
BRD3	8.47777	11.3508	11.2502	9.15391
KLF13	15.6591	17.1889	16.717	19.1054

ZNF764	3.2288	3.08351	3.22767	3.157
ZNF747	0.859374	1.23565	1.27421	0.942336
ZNF768	29.5981	30.0727	31.7719	28.9862
TMEM42	8.39521	8.48214	8.70717	8.67281
MAP3K2	8.96273	5.29618	4.6662	5.44033
PUSL1	58.4432	49.5126	54.3924	50.2952
SF3B5	183.594	146.915	149.056	157.077
ZNF35	3.88329	2.5884	3.4311	3.42125
IFFO2	14.9759	10.6327	9.94641	11.1111
NLGN2	18.1518	23.4256	25.7288	23.28
CHD3	19.5779	19.5469	21.7032	21.1815
ALCAM	18.9266	16.5782	19.7655	20.8466
YWHAG	120.636	114.223	112.099	118.277
UBE2E3	66.5389	61.6356	66.9922	80.7543
CNTROB	34.5215	33.8723	33.7636	31.9422
TRAPPC1	171.067	190.875	211.371	187.756
KCNAB3	1.69183	1.49394	2.00426	1.85132
SIMC1	8.73392	6.96942	6.80623	7.08662
TMEM192	10.7403	7.0203	8.42152	9.70568
RP11-423H2.1	68.1259	47.7973	49.1834	48.5177
ZNF778	17.6015	11.9591	10.6884	12.0779
NIPA1	12.5543	10.8225	11.7009	12.9802
FOXD4	1.62496	1.32108	1.51504	1.55213
UBE2E1	77.186	72.8344	69.9334	74.0918
HNRNPA3	421.165	339.602	348.995	395.068
SIK2	4.23795	4.8385	4.82554	4.38552
RP11-262H14.4	3.53469	1.66342	2.22128	2.44341
CHRNB1	8.03479	7.71696	8.97718	9.09976
USP38	9.84484	8.07825	9.21899	9.45378

SLC16A5	21.2844	27.0114	31.5936	26.7029
NANP	6.09552	4.70564	5.31638	5.83748
ADPRM	4.74473	4.81382	4.50624	4.53276
FABP6	3.31544	5.20372	3.01624	3.25778
PWWP2A	7.22397	5.06385	4.74299	5.66776
USP47	11.3405	9.10238	10.5215	10.9497
PDCD6IP	149.797	127.466	139.212	147.192
ZNF212	6.25323	5.16512	5.29048	5.81786
FAM161A	2.76291	1.76438	1.79864	2.02704
ZNF282	14.8437	17.0818	15.415	14.416
GLB1	22.0914	23.4316	23.1204	20.1051
C14orf142	12.4554	10.1007	9.90666	11.8464
FAXDC2	1.75386	1.72878	1.96883	1.54348
CRTAP	70.6892	76.2823	65.5581	69.6671
ELP5	105.991	94.288	91.8461	93.1992
CMTM8	23.6275	21.6166	23.1553	23.2017
GABARAP	201.362	212.518	211.415	219.638
STX8	28.9595	26.8687	27.3355	31.5389
CDK1	105.276	84.5302	78.6331	106.628
UBB	1264.19	1222.99	1183.3	1183.7
NFRKB	9.84623	8.52936	9.00548	9.04424
PRDM10	2.32482	2.24797	2.28844	2.24606
B3GNT2	10.9805	10.2047	10.5118	11.2964
FOS	3.68153	2.72668	3.36979	2.92178
TMED10	179.501	161.486	164.966	176.297
OR2A20P	1.34556	1.65399	1.54847	1.54323
SETMAR	6.59215	6.39007	6.11838	7.41624
SMAD1	12.3593	13.1599	13.0649	14.2459
LRRN2	1.67199	2.48249	2.24503	2.01004

SLC30A1	5.91609	6.83393	5.8918	6.53095
GPRC5C	21.0581	30.0437	27.589	25.5099
TMEM182	4.83095	5.50113	5.12403	5.99648
KRT8	1463.25	1311.06	1421.68	1332.2
ADORA2B	50.4041	51.093	54.1983	52.1167
MGMT	7.37898	8.19073	8.25598	7.92103
METTL7B	10.0713	10.2215	12.2272	12.0954
HARS	85.3201	77.5066	80.2609	79.3531
NFXL1	11.1602	9.75994	8.92968	10.2179
DENND5B	6.29948	6.04502	6.57849	7.31642
DNAJC18	3.21569	4.15055	3.66006	4.38523
AC005280.1	1.72053	1.89641	1.34388	0.80421
SPATA24	3.51513	3.17872	2.85747	2.91902
RALGAPB	7.27365	7.54512	7.60779	7.77529
WIBG	22.3612	24.1268	22.232	22.7554
NPAS2	17.1375	12.6426	15.995	14.3683
NUDT9	13.7191	11.5855	11.4298	13.299
PA2G4	350.288	280.058	297.035	318.385
ELOVL6	20.0733	16.5918	18.6471	21.6373
PFKFB3	38.5723	28.6809	32.6578	39.6547
TMC7	1.92932	2.31563	2.06561	2.25902
ARL6IP1	164.179	151.715	133.532	148.803
SERPINB9	11.0101	8.55722	8.45831	9.27386
SMAGP	30.884	22.9327	25.1289	27.9737
EMB	5.68799	5.76723	5.51215	6.14275
STAT2	8.43105	8.83903	10.0175	8.93165
NUDCD2	38.3107	42.5727	37.3399	38.3867
IRF2BP1	18.4412	20.1826	20.7086	18.495
HSPA4	151.691	128.837	122.17	142.714

FOXA3	6.37789	5.15997	5.35266	5.00003
COMMD5	43.7687	39.1848	40.0081	39.0024
GTSF1	245.177	182.049	150.268	146.808
ZNF16	5.49413	5.15999	5.20788	5.30491
ARMC10	41.8397	42.6036	43.5072	48.0262
RNF34	17.6533	17.072	15.079	14.4339
ACYP2	6.29906	6.99024	6.12753	6.88169
TRABD	41.8473	42.8817	47.2793	40.4228
TMEM133	2.11891	1.53237	2.09111	2.14892
ATF7	6.32383	7.04025	6.14983	6.44891
RASA4B	1.18817	1.27169	1.35787	1.30152
SOCS6	21.6626	21.2844	21.4134	22.2108
ZNF296	9.10076	7.44159	8.25449	7.12104
HOXB9	12.6874	14.5179	12.3135	12.8416
BOP1	102.09	82.0623	83.7149	84.4756
POLH	11.2348	13.6431	14.0839	13.8425
KCNS3	4.2173	2.89348	3.51137	3.74426
KIF5B	59.3433	54.4763	45.2214	52.762
AKAP13	8.98017	9.2869	8.08922	8.78141
CDCA4	69.4574	57.7692	60.2266	62.6732
CHCHD7	18.1583	18.2662	17.9762	20.1187
FOXN2	8.34799	7.22748	7.42417	8.65906
USP32	15.8257	17.0293	18.5757	18.4341
PPM1D	17.5034	19.0971	19.2297	18.24
AC093323.3	16.7986	15.8731	16.0063	16.2481
KBTBD2	13.5984	12.1788	12.0699	13.0549
MINA	28.7609	26.0808	26.6047	29.4161
TRIAP1	58.4793	65.5584	56.947	61.8191
LSM3	19.0237	17.7452	15.5289	18.3089

KIAA0232	3.50618	4.52398	4.00454	4.35773
MTSS1	2.62951	2.49975	2.6777	2.50583
TMEM43	20.2556	17.1348	17.1766	17.0565
RNF139	29.2275	21.5008	24.6295	26.5131
RPS9	215.061	219.381	183.739	172.538
TSEN34	15.0787	13.112	12.3473	10.4267
MSANTD4	6.27237	5.39353	4.66757	5.58402
NDUFA3	46.6935	46.7284	39.0319	34.5798
PAQR8	0.378971	1.4129	1.58985	1.3351
NUDT6	5.14309	4.91568	4.33965	4.72754
TPT1-AS1	23.0085	25.672	30.0477	21.9078
TANC2	5.60704	4.81036	5.82028	6.28785
DNAJC24	9.52581	8.06948	8.63494	10.3839
ZNF160	3.27235	3.28441	3.72244	3.73556
PRKCDBP	10.7939	11.5725	10.639	10.1247
PLAC1	0.939324	0.99395	0.707244	1.03926
HS6ST2	18.8958	17.1362	17.1342	18.7134
LRRC8E	4.17405	3.42483	3.8533	3.43652
PKIA	1.07761	0.957243	0.842039	0.933856
TSNARE1	5.35895	7.0113	7.49041	6.32832
FEZ2	11.3831	10.4978	11.2792	10.8664
C11orf24	48.8101	44.6389	51.2239	50.0479
FAM86JP	2.28257	3.92317	3.88493	3.89962
CCBL1	4.93459	5.11372	5.23543	5.07144
MTM1	1.84961	1.79492	1.7833	2.28224
TRMT61B	7.90969	7.20317	7.28473	7.90028
INSR	4.22606	4.28459	4.5398	4.48539
MFN1	21.734	15.1731	19.8085	19.119
NRTN	8.9776	8.96016	9.5294	9.24794

ATP6V0E2	28.6298	24.4331	29.5605	28.7623
PRKCE	2.29992	2.09769	2.25038	2.52763
JAGN1	34.7529	33.5633	35.7566	35.4111
TADA3	39.4889	39.4666	39.9104	39.3179
SOCS5	8.55529	6.26939	7.44861	8.32808
C1GALT1C1	13.7949	12.2401	11.7692	14.3202
C9orf16	88.4763	92.8614	94.3019	87.3284
MORN4	4.329	4.49422	4.76168	4.59431
ZNF672	16.5097	13.3546	15.2919	14.3608
ZNF692	38.4009	38.4563	39.417	36.9962
NAIF1	8.24704	9.72574	10.1012	9.07432
RBKS	1.22336	1.56234	1.4265	1.66013
TMEM126A	44.1768	45.7157	41.465	45.7062
TMEM126B	51.7558	47.5607	42.9988	53.4803
TRIM8	34.0702	34.9396	37.1635	34.7246
NETO2	10.2942	7.3022	8.68996	8.84111
CDC42BPG	9.25018	10.6054	10.6391	10.9678
SCAND1	39.4501	41.1515	45.1368	40.6668
JUNB	40.8952	34.8963	43.6062	40.464
C10orf35	22.3397	18.5837	20.0519	20.4974
TMEM37	1.21165	1.49147	1.89849	1.47398
SHCBP1	37.081	31.9933	34.7956	35.363
FAM98B	47.4264	37.4135	36.6621	42.0028
RP11-1055B8.7	3.28802	3.13623	3.15406	2.76851
ZNF440	3.27207	3.51101	3.40518	3.7208
GAA	38.234	50.3088	51.911	46.8512
CANT1	56.6731	48.9366	60.3453	55.7406
ZDHHC16	37.5363	38.3264	40.9038	40.0119
CHST11	9.59401	7.92743	8.80261	9.67602

EXOSC1	52.9395	48.3726	45.6549	46.0533
PGAM1	557.093	525.232	523.928	529.646
CHD7	11.5327	10.2985	12.2477	9.60935
ESCO2	21.3655	14.5573	17.1237	17.7434
KRT19	715.629	809.349	866.215	813.824
KRT15	18.7741	22.177	20.974	22.7165
CLCN5	1.26017	1.07124	1.02836	1.17979
TPPP	1.36556	1.06563	1.40045	1.33313
MRPL36	106.278	86.0283	88.9212	88.5036
ZNF581	29.8543	24.6689	28.3648	25.7342
NAT1	3.40543	2.66377	3.02898	3.64529
KSR2	1.23715	1.25101	1.31203	1.36431
ZNF524	9.95488	11.9162	12.3934	10.7806
MCC	6.96931	6.54961	7.20353	7.45169
ZBTB26	1.84697	2.33092	2.30216	2.56006
POLR1C	92.684	73.7149	77.5488	89.6135
ASXL1	26.8221	24.24	23.7373	24.8609
DLK2	1.80618	1.15783	1.34632	1.33096
ZNF562	9.37729	8.53096	7.56621	8.42741
ZNF318	4.12844	3.67533	3.70428	3.96834
ZNF561	16.9076	17.0134	15.518	14.8524
MAP1LC3B2	8.11841	7.00753	6.43632	7.68507
WIPF2	9.14518	8.33593	8.6416	9.49745
LRRC8C	4.19629	3.5535	3.67743	4.11525
RSL1D1	173.585	157.237	136.606	162.888
LRRC8D	8.33049	8.5945	7.38642	8.37076
PPID	79.0554	66.7592	63.815	68.8131
ETFDH	11.603	12.0106	11.1785	12.8539
LPAR3	14.6453	12.1993	12.1996	13.2087

PTGER4	1.03472	1.05036	1.06284	2.03381
TBCA	233.942	230.234	179.486	226.307
BCL2L1	172.398	175.026	182.267	184.354
PLRG1	84.6359	82.6832	77.1495	80.3791
RAB4B-EGLN2	1.02912	1.23912	0.320179	1.0812
ZNF584	17.4159	13.6171	16.714	14.4605
CLSTN1	49.6624	49.6801	52.2915	51.1994
CXXC5	28.1425	26.6591	29.02	27.0935
ZNF274	3.4929	4.87909	4.26541	4.19794
PIK3CD	2.96	3.3868	3.64781	2.90726
SLC25A33	12.2769	10.8775	10.1441	11.8128
ENC1	15.9039	14.2848	16.4356	15.1112
SPSB1	8.94255	8.17931	8.58062	7.73623
BPTF	23.3846	20.4778	19.5564	19.5681
PLEKHG5	7.59033	7.87988	9.38533	7.90192
ATF7IP	6.56126	5.32463	6.33713	5.63426
RGS19	28.9334	25.9051	28.8291	26.8358
TCEA2	13.3624	15.8146	15.1293	15.6293
HDAC3	89.8491	83.5909	85.5934	80.9107
GPHN	24.2223	22.1294	26.4812	27.8597
TMEM51	12.6829	11.1005	12.4801	12.0023
CAMTA1	41.6983	41.4318	37.1628	44.6407
SPATA5L1	11.5583	11.3368	12.167	11.5777
GATM	0.81633	1.12831	0.994957	0.967779
BCL2	1.09583	0.936733	0.90293	0.927392
RHNO1	22.0736	20.0201	18.5432	20.0274
CTPS1	89.6472	68.281	61.0256	69.4276
KNDC1	1.23207	0.972011	1.48827	1.16901
METTL18	4.51578	3.84965	4.11264	4.57653

PWWP2B	8.77126	8.45695	9.49652	8.50234
FBXL14	12.6085	11.0981	12.307	11.6837
EXOSC10	40.3101	36.8041	36.474	38.2182
MLLT3	1.8224	1.7873	2.01877	1.38658
RRM2	374.19	273.289	263.49	304.733
TRAPPC12	26.5186	24.7928	25.7356	22.9143
RPS21	3373.79	3096.07	2713.92	2822.98
RNMTL1	29.4492	25.8422	26.2795	27.7333
PTEN	30.6862	29.3571	27.2891	31.7573
RPS7	1084.66	981.982	847.806	995.114
RNASEH1	27.9616	26.2748	25.6765	26.7492
PRNP	58.7453	51.9266	43.6952	45.5716
FRMD5	15.0779	14.0872	14.4592	14.9546
MIR31HG	1.06674	0.887801	0.922713	0.754923
CYP4F11	12.4146	12.0614	11.3632	11.7212
TLN2	2.17969	2.27847	2.70042	1.63327
TVP23B	45.651	37.8536	41.0324	43.6826
ZNF217	9.18069	6.94637	7.3904	8.12526
SRGAP2C	2.41403	2.05405	1.87017	2.00386
SCG2	4.74481	15.3986	20.2808	14.9333
ATPAF2	25.6764	27.9182	28.7797	25.3993
CYP4F22	2.19287	1.07528	1.73836	1.74303
PPIH	147.93	115.652	109.708	125.437
LRRC48	0.873138	0.898915	1.12998	0.704802
ZNF57	4.48183	3.9856	4.00409	4.18502
C20orf196	1.76156	2.44853	2.29968	2.2491
JMJD1C	11.0975	9.95731	9.74536	10.8015
SYNPO	0.39186	1.2692	1.38818	1.34498
RAB33B	2.16174	2.58395	2.84476	2.65273

THOP1	109.028	91.7969	100.598	91.9038
EPHX4	2.20121	2.17045	2.0893	2.41898
LAMB2	57.5914	68.809	82.6045	64.6751
USP19	45.0956	44.2238	49.7263	44.0675
QARS	164.798	183.928	183.792	181.63
ORMDL3	31.8264	28.5174	30.0116	30.8101
SERF1A	7.65631	7.8719	7.18661	7.94501
KLF11	2.76745	3.33435	3.08342	2.44108
SMN1	44.8113	19.022	19.5421	24.8662
EIF2AK3	3.94532	3.91547	4.59403	5.09922
MOB3A	7.7362	9.15224	9.53945	9.24294
KRCC1	1.24422	2.57029	1.84924	1.88539
NME6	17.0835	11.2887	14.3431	14.3501
CYCS	314.253	259.929	251.41	280.963
CALB2	2.76869	3.20794	4.61626	4.38416
SNTB1	0.830771	1.10532	0.95814	0.838423
MTBP	7.38265	6.71083	6.02224	7.522
TEFM	11.7462	10.6153	9.56654	10.7551
MRPL13	108.913	115.047	98.026	109.599
MALT1	25.3075	23.3984	22.4237	23.6514
ISG20	1.2366	2.81381	2.05143	1.84841
MBOAT1	4.45255	3.39514	3.2936	3.87337
CEBPB	30.7469	27.1143	25.7468	24.1231
PAIP1	67.5228	63.3599	64.776	68.4354
C5orf34	4.10899	4.8424	4.27832	4.78865
ZNF131	15.3599	16.8179	14.8815	15.7235
DPAGT1	27.2266	28.1972	31.6301	29.9897
BSG	450.454	478.963	444.932	416.191
HINFP	16.3354	15.8432	16.7479	15.3746

CERS6	9.25517	8.3604	8.20855	9.34901
COPRS	88.962	70.0654	67.93	70.4858
TP53RK	11.6104	10.9161	10.6265	11.6552
BPGM	8.65332	7.04495	7.17511	7.6584
POP7	82.8924	63.1782	68.8068	71.307
ALG14	17.0798	10.8131	11.2281	12.4078
SUCLG2	20.4032	22.2521	21.3994	23.0555
STARD5	1.90511	1.8207	2.02282	1.90291
ABCG4	1.02443	0.884028	1.25016	1.06288
GNB2	271.093	242.812	274.618	253.711
FAM195A	25.836	24.7321	26.4896	25.6058
C2CD2L	11.0173	8.04644	8.96827	8.71852
ARNT2	1.28183	1.43969	1.41392	1.55613
GNG12	23.9628	23.0799	24.4445	26.434
CLP1	19.7082	18.1649	19.4038	19.7771
MYEOV2	130.747	125.086	102.973	140.608
GTPBP2	21.4217	24.9823	21.8603	21.3574
FGGY	4.03458	4.73526	5.16901	5.39775
IL17D	3.69633	3.68779	4.11992	4.38569
PRSS30P	2.78294	3.60845	3.63551	3.00906
ZNF24	15.4157	13.6693	12.9643	14.4907
MANEA	3.3147	2.7076	3.09537	3.35054
AFF1	3.79466	4.1209	4.02762	4.52146
FIBP	81.0935	85.1926	82.2456	75.7358
BANP	13.2206	11.5235	12.0606	11.1465
PPP1CA	158.614	155.268	158.47	159.232
HCFC1	14.1998	11.8032	12.15	12.3217
CHCHD1	64.2683	58.6078	54.9684	62.1589
MRPL52	295.958	254.431	250.532	267.043

SMPDL3A	1.20298	1.07421	0.994931	1.14161
RAD9A	15.7031	12.7062	13.0404	12.1286
EFEMP2	0.815304	0.679716	1.15898	1.1264
AGAP5	1.75721	2.14087	1.68814	1.42858
TAF15	99.616	88.0487	89.0647	89.9267
FAM21C	4.88148	4.62774	4.00917	4.41564
TMEM134	29.858	33.53	32.5733	32.2547
ZMAT3	4.33184	6.38856	5.87982	5.41641
ZFAND4	7.40167	5.55839	6.15512	6.74814
ZNF738	3.3027	3.3711	3.12103	3.785
CORO1B	59.7966	67.8196	73.2028	68.5643
FUT10	9.97128	6.67037	6.01619	7.43395
LRRC20	17.2807	15.5195	15.837	15.703
MUS81	29.5973	32.6849	30.4263	29.7931
ZNF596	2.1552	2.62008	2.09432	1.84441
CFL1	1140.95	1092.01	1116.19	1104.96
TMCC1	11.6327	16.8532	13.2225	15.898
NAA16	6.38395	6.52613	5.71547	6.34006
FAM192A	53.1912	51.6131	53.1659	54.6895
RAB43	4.63707	5.25503	5.58488	4.92437
CBWD1	46.7605	43.7664	40.339	44.5686
HOXC5	1.46104	1.31438	1.56366	1.59497
RAB37	2.24009	2.50163	3.78088	3.36002
DCP2	11.3807	9.78758	9.51962	10.7349
SNX32	12.0402	9.23098	8.35249	9.75604
RPL38	2328.67	2160.54	1848.02	2337.23
OVOL1	4.66503	3.66064	4.29394	4.21146
RARG	27.0113	21.2172	23.9144	24.3023
CES3	1.48446	2.21189	1.51183	1.55139

SSH3	21.0883	23.7614	27.5326	23.5403
CES2	14.3497	17.948	17.8181	16.021
PDP2	6.13664	5.31302	5.17069	6.05793
SP3	40.3591	36.7566	38.3711	41.1833
LSM2	16.4032	13.5798	14.7033	15.8519
DMXL1	4.06696	4.99757	4.53632	4.62452
METAP1D	9.20785	8.82537	8.83395	8.37159
ZNF621	3.01333	4.30542	3.60146	3.3908
EGFL7	66.7466	68.8709	75.2856	64.6608
NADSYN1	19.982	24.5465	24.3288	22.3383
DHCR7	53.315	38.0865	43.7701	42.5467
RNASEH2C	30.3986	28.5164	27.4535	25.6076
MYEOV	125.223	96.6839	113.674	119.441
ANKRD13D	18.0198	17.893	19.2087	17.8237
MYD88	11.6898	12.6985	15.087	13.3083
OXSR1	14.9322	12.7668	12.6384	14.2132
PHF8	4.19415	3.76877	4.27516	3.76273
LCLAT1	10.6537	8.4446	7.96783	8.263
MIR4435-1HG	37.8941	31.5042	33.5192	34.5711
AC007318.5	6.93632	7.06619	6.40945	6.90542
KAT5	37.0636	34.2464	33.3985	33.8752
GXYLT2	1.37985	2.34606	1.34516	1.87063
DCAKD	16.8262	19.5618	21.821	19.2732
TADA2B	9.11253	7.55758	7.75934	8.34462
ADRBK1	87.6152	73.4047	82.7596	72.5219
RELA	45.3122	37.8596	43.3024	42.4505
ZNF680	4.88644	5.24083	4.16443	4.95901
HECTD4	4.29403	5.66649	5.46478	5.11908
FAM222B	10.5573	10.6434	9.99603	10.1396

HPSE	6.89244	6.57449	6.18475	6.44278
COQ2	23.2413	21.4693	21.4216	22.6856
TRMT112	436.487	412.795	400.08	430.111
KDM2A	24.1193	24.035	24.0012	23.1852
ADCK5	4.59507	5.612	5.52023	4.44997
MRP63	31.2984	30.2866	30.8782	31.5788
NOC3L	23.8348	21.1629	20.8707	23.7514
ESRRA	49.8216	50.4774	57.4503	50.6379
RHOD	46.3005	49.7715	55.4478	52.3406
COMMD1	17.1396	17.7908	16.2376	18.3697
RAPH1	3.03253	2.638	2.57836	2.83353
MTX1	53.0585	46.9226	51.4202	47.8005
PARP14	2.72024	2.8335	2.3849	2.67305
CKS1B	291.094	237.515	224.503	243.586
AHSA2	10.1934	9.71876	9.06941	7.94494
ABLIM3	4.48277	5.17298	5.23082	4.84672
KIAA1919	1.14783	0.863877	0.954658	0.943358
VANGL1	15.096	14.2013	13.727	14.0088
GLRX	22.0241	20.2695	21.4654	20.2156
IQCB1	11.4476	11.2565	9.26452	9.16839
SYT12	1.17465	1.18815	1.43187	1.22146
GOLGB1	4.55474	5.3345	4.67116	4.72817
PLAC8L1	0.983548	0.836233	1.30916	0.955125
GPR137	23.5316	24.2944	31.1808	29.3971
SNCG	24.7363	30.5205	30.1779	28.7298
MMRN2	6.68017	7.649	6.94268	7.0861
MZT2A	244.108	151.095	214.72	204.623
TNKS	6.43049	6.63994	6.3407	6.53507
ZBTB21	7.30709	6.53474	6.32786	6.9811

PPP1R3B	2.09508	2.31804	2.46925	2.45389
FAM86B3P	2.28417	3.13983	3.12607	2.60962
MAP3K11	24.9097	27.0924	29.8128	27.1844
TRIB1	85.9838	81.5731	98.8372	98.9426
SFT2D3	2.99178	3.87393	3.98962	3.68998
DAG1	47.2188	40.4828	33.6999	36.6476
ARV1	2.58045	2.22981	2.39263	2.18847
NAA20	108.999	87.676	87.1566	102.174
MINOS1	143.13	145.819	141.173	152.372
EHBP1L1	36.2484	32.1453	39.1236	37.7413
THAP2	2.07669	2.32547	2.41719	2.76915
RNF26	29.7943	26.7806	28.7787	27.8735
PPP1R14B	434.878	368.437	369.58	396.142
SSSCA1	90.9571	75.5809	73.6428	74.4442
SMARCC1	43.5802	41.5853	40.6869	44.3058
ZNF417	1.61665	1.67358	1.42891	1.54881
PTPRM	2.46897	3.23737	3.55045	3.1829
FKBP2	112.222	97.4593	97.0343	99.738
VEGFB	29.7564	19.7557	23.5015	22.0129
PEAK1	2.56291	1.91597	2.09457	2.13729
TNFRSF10D	23.1332	28.4878	23.0346	23.2741
MST1	4.80154	6.1674	6.1654	6.2283
TNFRSF10C	1.79803	4.21068	3.19748	2.56825
GMPPB	23.3625	19.6961	20.8692	20.5313
MOB1B	6.27355	3.82108	4.01344	4.5246
ZNF622	23.9595	21.1963	22.8409	22.6847
CSPG4	3.93917	5.76672	5.56669	4.79909
SNX33	3.26866	3.96954	3.93741	3.53683
C2orf70	5.66534	6.21438	5.26702	5.04327

NABP1	9.00051	10.416	9.96514	10.8735
NUDT18	5.9259	7.69604	8.77837	7.67187
CHD2	42.1318	42.0199	36.4758	43.6961
CCDC41	6.32883	6.3433	6.89718	7.11925
NUDT4	25.7206	21.4789	20.6661	23.1128
PC	6.76279	7.26375	9.42755	8.91
SCAI	3.51988	4.00717	3.98959	3.9484
NMNAT1	4.41606	5.63774	5.29515	5.32214
LRFN4	45.9621	44.5209	51.6013	47.7327
SLC19A1	80.5058	71.6091	76.7137	70.9917
RCE1	41.2553	38.947	42.4529	39.7225
UQCRH	680.805	618.017	564.95	576.182
EIF1AX	84.9044	61.2646	60.6253	71.2502
CXorf23	0.989608	1.14031	1.24557	1.28447
PSMD1	154.049	133.628	152.385	154.403
HEG1	1.25824	0.911268	0.986797	1.17474
C11orf80	8.60318	9.40215	7.27538	6.93015
TOMM20	230.997	179.188	190.028	184.428
AP000769.1	1.97306	1.63467	1.2401	1.7968
AGFG1	63.3678	54.3222	55.8499	61.7554
STAT5B	1.37392	1.24799	1.60822	1.21799
CNP	24.7976	27.2881	28.8032	25.8308
JUP	26.0393	43.9607	46.2465	24.6336
EIF1	497.426	517.952	450.757	476.05
ENDOV	15.9979	18.6496	17.7503	16.8772
RNF213	8.06113	11.8472	10.7951	9.22901
PLK3	30.8042	30.6746	32.2738	29.8989
NET1	23.3842	19.1015	19.6654	20.1473
DPY19L1	34.9143	33.1347	33.272	38.2494

PHOSPHO1	2.74275	2.24217	2.19842	2.04015
ZNF791	6.76755	5.10622	6.37837	5.57329
PHC3	8.03275	7.23828	7.597	6.88477
GPR160	6.24252	4.34495	4.49926	4.95646
CBX2	10.0901	8.04864	8.7217	7.68598
SPTBN2	10.0914	11.2909	11.6387	11.6671
GOLIM4	5.21042	5.22192	5.54274	5.6678
RBM4B	16.7696	16.7763	16.9904	18.0737
USMG5	611.723	614.667	531.396	600.312
HOXB2	1.09975	2.00982	2.15329	1.60343
C1QTNF1	1.41715	1.55381	1.9328	1.6818
3-Mar	1.78983	1.87085	1.9263	2.06719
SWSAP1	2.03092	1.64866	1.82981	2.16899
RBM4	86.7554	84.5021	86.829	86.2871
XXYLT1	6.36708	7.88224	7.60643	6.93499
UBXN2A	7.81755	7.94172	7.45316	9.24391
CCS	15.6142	20.1786	18.3716	17.3931
CEP19	1.69894	1.64136	1.63118	1.77938
KLHL15	3.46032	3.07124	3.03159	3.41746
FBXO45	21.1122	10.5535	12.9247	16.3951
GNG5	141.068	136.442	128.266	137.148
FAM3C2	10.7988	9.68446	11.8178	11.2729
SLC25A30	12.6397	10.0784	11.4791	13.1036
RP11-1407O15.2	6.57889	6.32381	6.93157	7.35995
MSRB3	5.53793	4.07512	4.54611	4.38753
MRPL45	43.2549	38.9725	35.7248	35.0728
LEMD3	8.49719	8.0774	7.62296	8.0493
C16orf91	34.5242	29.1929	28.975	28.8279
SOCS7	6.1803	6.69354	6.63687	5.87597

FAM174A	9.43172	7.99703	9.88086	8.97769
RGMB	6.19964	5.94129	6.04053	5.49797
CYB561D1	2.20597	2.6607	3.09577	2.54193
ZDHHC24	5.7112	6.48776	7.33504	6.61436
RP11-23P13.6	2.02039	1.76012	1.90454	1.97389
TRMT10C	37.9139	29.4035	28.7141	37.2785
CTU2	104.496	81.4877	83.2706	80.3661
AGAP8	2.59065	2.31528	2.36757	1.97869
FAM21D	3.54002	3.33327	3.22404	3.05774
MGA	4.53343	4.01397	4.0587	4.56922
C12orf66	5.11316	5.7053	5.4861	6.14139
PIGG	22.0219	23.7406	23.2363	21.7825
PRPF8	192.471	166.608	180.347	164.376
ADCY6	8.03672	9.02067	9.11537	9.73505
REP15	1.11676	0.953732	1.05402	0.984804
PITPNA	39.4865	38.3576	40.7176	44.0261
DDX23	98.0345	72.2909	77.5873	75.4684
ZNHIT2	21.2792	21.3335	23.272	21.1931
ZBTB4	5.5555	4.66719	5.58915	5.45484
TNK1	16.6215	15.3017	14.6099	11.9165
ZHX3	3.48491	2.94646	3.81688	4.2043
PHLDA3	30.0213	43.3078	37.201	31.5346
SLC16A13	2.33592	2.90224	3.1745	2.47768
STAG3L3	3.51027	3.56702	3.40318	2.96976
SNHG11	8.74071	10.0795	8.68521	8.74357
PMS2P2	1.0778	1.08214	1.0498	1.18342
C11orf45	1.01833	1.32755	1.65963	1.35273
EXO1	21.6698	14.5317	18.2363	19.1325
RALGAPA1	5.19931	6.82106	5.92453	6.40856

WBSCR16	21.3364	19.2166	20.1813	18.9761
RP11-313P13.4	0.80372	1.39337	1.3334	1.51647
LIG4	4.53857	3.63434	3.44879	4.12528
GTF2IRD2B	5.77423	6.45505	3.36556	6.11872
ATP2A2	121.983	111.516	115.254	124.589
ZWILCH	27.9273	19.7813	19.6592	21.897
RPL4	3377.53	3394.37	3249.8	3387.52
SNAPC5	38.0793	31.392	32.1098	35.0018
C12orf76	5.00877	6.15465	6.19098	5.37893
BBS1	9.04072	8.74167	8.04092	8.05065
DENND4A	2.10083	1.7306	2.13067	1.98809
MFSD4	0.630168	1.06613	0.70802	0.84609
PELI3	7.71548	7.97965	7.5906	7.37911
TTC9B	0.937849	0.599142	0.760337	1.05377
TMEM81	3.53858	2.75329	3.24875	3.25952
MRPL11	169.929	161.029	148.507	145.627
IL20RB	1.07093	1.3351	0.815863	0.759128
GOLT1A	4.05512	6.05064	7.02381	7.88954
AKIRIN1	49.9245	45.698	45.187	47.3109
MSL2	11.8316	9.95626	8.77392	10.5316
ZNF497	0.906684	1.59803	1.18269	1.00467
ANGEL2	8.7466	8.11982	9.42061	9.34098
UGT8	8.65896	7.95854	8.16213	8.77774
IQCK	3.98724	4.42016	4.04358	4.44348
ZNF266	4.43225	4.17732	3.65075	3.67
SLC29A2	16.4009	17.9576	18.609	16.4012
BRSK2	8.25304	7.57826	10.065	9.22278
B3GNT1	5.77492	5.73762	5.6227	5.66988
TMEM167A	143.306	113.324	109.771	119.003

SH3PXD2B	9.82625	9.55077	10.385	10.5476
KIAA1551	10.4919	8.88152	9.07861	11.5398
LARP7	30.4175	26.1946	27.3194	29.3078
NR1D2	18.338	17.427	15.7294	16.8783
BRMS1	66.6603	55.3334	56.5329	56.2843
RPL15	1387.88	1399.28	1341.64	1343.78
C4orf32	6.45552	4.83245	4.50665	5.09195
HRAS	137.883	124.118	132.737	121.533
SRP72	117.264	96.9835	99.0327	108.816
RIN1	19.1056	24.2927	25.466	22.9632
THAP6	8.55897	7.05845	7.70714	7.24659
CEP135	3.3969	2.79489	2.62533	3.17354
FZD4	1.37212	1.35729	1.44596	1.44922
BTC	0.598247	1.04595	0.986131	0.830343
DENND6A	5.45239	5.35246	3.86811	4.56925
PDE12	21.6015	16.3206	16.7259	17.5876
GLMN	14.0945	11.5043	10.8739	14.1834
YIF1A	57.1137	54.1583	61.9816	55.8553
NDUFA11	327.642	334.507	365.277	328.873
RSRC1	15.7534	14.3388	15.4053	15.3256
RAB1B	101.671	85.5216	95.5632	95.6843
PTDSS2	32.3443	28.2071	32.0332	27.9661
C19orf70	80.7119	81.4168	86.8143	80.2354
C3orf33	2.96898	3.13116	2.88844	3.28354
SEZ6L2	12.6112	16.7732	18.6723	15.1459
ASPHD1	6.66366	8.28414	8.66852	7.78946
KCTD13	15.8009	16.8855	17.1911	14.0219
CD164L2	2.38334	2.69311	2.74771	2.29138
FUT1	3.54852	3.64149	3.27772	3.18339

DHX36	27.6631	28.5365	30.095	31.7355
FBXW8	4.17841	5.10529	5.13093	5.01031
KLC2	43.1277	44.4667	45.6258	42.2951
CTBP2	90.1328	69.5193	79.3177	76.7872
CHST2	1.67138	1.44091	1.77711	1.69914
ZDHHC14	4.06354	4.04314	4.65114	4.57879
ATR	14.5613	15.4192	14.0174	14.3615
C17orf76-AS1	584.985	649.131	594.89	631.268
UBE2C	237.231	191.075	186.326	197.755
GK5	2.14682	3.0922	2.91863	3.55462
VCPIP1	4.0028	3.83245	3.79044	4.22176
DES	1.19338	1.44354	1.73003	1.39302
PDIK1L	3.88762	3.10898	2.99748	3.43671
TRAF6	4.43989	3.59956	3.74394	3.61355
ZNF654	1.16212	1.03373	1.13213	1.34383
TVP23C	9.54816	12	10.543	11.8972
MRPS22	90.2732	79.7846	76.0015	83.9794
PACS1	16.5583	17.2462	18.7902	18.1437
MARCKSL1	94.2599	103.966	99.6468	99.3299
SH3BP5L	15.0872	12.7351	14.7123	13.5475
PSMD2	494.478	430.225	471.297	478.589
FAM131A	9.44846	8.64862	8.79044	9.09994
CSRP2	18.9137	19.35	18.5757	17.8143
PARL	66.6344	56.8756	54.7945	62.3024
DDIT3	11.9427	17.6416	11.8875	12.5563
PCCA	7.79421	10.6297	9.81035	12.8747
DCTN2	138.683	133.11	168.169	187.724
ZNF408	8.48829	8.43213	8.14057	7.71183
CTDSP2	25.1349	21.3574	15.0611	16.2043

CKAP5	64.6928	58.3827	55.3435	58.1893
ARHGAP1	18.8276	19.7693	19.8534	18.869
MED16	46.6585	47.4012	48.7134	45.2239
ATG13	31.2029	37.979	39.2482	37.0266
GOLGA8A	7.39414	7.9502	8.31994	7.76591
TP53I11	20.5568	20.9696	22.1447	21.7246
APITD1	21.2525	18.1911	16.1198	19.7682
DOLK	17.4366	17.0444	16.8329	16.5268
CATSPER1	1.82386	2.10282	1.72603	1.58823
CCNE2	14.2473	8.70726	9.54473	11.7792
PHYKPL	18.8608	18.4931	17.192	16.7735
CST6	94.2585	73.4913	91.02	81.3089
GRAMD2	3.78844	4.64842	4.65085	4.5704
ZNF519	2.48746	2.38638	2.19735	2.28507
LSM1	53.3394	46.788	45.2156	51.0765
BANF1	304.21	288.743	294.903	321.691
CHRNA7	2.82069	2.5818	2.33446	2.44731
TMEM9B	31.5815	26.3268	27.5461	30.8177
PTPN2	40.6438	31.861	32.6318	35.9973
EIF1AD	47.4622	33.316	35.9367	37.9075
SMAD2	50.8234	47.106	46.951	50.5505
EIF3F	217.316	223.084	226.099	217.005
ZNF25	1.16333	1.23145	1.05552	1.16334
CLTB	88.6167	73.7214	82.8106	79.561
RFESD	2.07772	2.32212	2.68617	2.68034
CCDC14	19.391	19.0248	21.0762	19.906
SART1	63.4293	55.3758	56.0829	62.5564
PPP2R2D	8.60913	8.19044	8.22585	7.95203
POLD4	11.1432	14.6095	16.9115	15.2841

CLCF1	8.26622	5.44532	6.62177	5.56026
LIPT2	1.04373	1.17619	1.05208	0.99289
ALG10B	2.00717	2.74379	2.4564	2.80001
DRAP1	174.991	161.56	158.29	161.575
LONRF3	1.1814	1.06865	1.10409	1.19119
UCP2	10.5674	12.6788	12.5752	11.2762
C11orf68	44.0026	39.6767	43.2811	43.0134
PAAF1	12.1172	13.3954	14.654	15.1666
MRPL48	58.782	52.6558	48.5066	54.3006
RAB6A	47.813	38.2952	41.8924	44.2436
P2RY2	1.96924	1.99309	2.19698	2.14868
FOSL1	155.176	116.973	106.39	111.983
ERCC4	2.60432	3.28473	3.38036	3.15712
C7orf10	1.58502	1.84435	1.96028	1.76336
CCDC85B	111.139	107.844	116.057	105.016
TMEM70	86.8835	64.9358	71.8256	77.5402
LINC00476	1.87906	2.39038	2.65955	2.49028
RPS6KB2	39.4414	39.7311	37.8052	40.6139
RMI2	8.68389	10.6107	10.749	11.3568
TOM1L2	5.94693	7.12077	8.56254	7.82475
ZNF77	4.55757	3.82765	3.8841	3.82146
LINC00116	41.023	39.6548	41.4857	37.3998
C1orf172	14.1043	13.545	15.4338	14.0757
B3GNTL1	36.7696	33.3646	33.2288	33.7386
MLXIP	22.3365	18.9541	19.5625	20.4801
BAK1P1	2.13166	1.45194	1.9098	2.29048
NR2F1	37.3229	31.0497	39.8475	33.0108
AURKAIP1	221.542	202.46	203.807	208.51
TTLL11	1.0326	1.15758	1.26818	1.18226

TOMM5	227.656	187.39	182.929	207.586
AC112229.7	3.8139	4.07417	5.53175	5.38891
SLC35E3	3.83224	3.251	3.73414	4.07103
ZNF169	1.48308	1.699	1.56868	1.56706
RUVBL1	138.923	131.705	130.718	133.204
SFN	211.218	215.358	222.097	224.269
MSRA	5.39859	4.45801	4.75593	4.52718
CTDNEP1	166.246	140.811	156.197	147.874
AP001266.1	9.73689	11.4966	11.1437	10.1845
ETV4	28.1946	29.5003	28.2958	28.3542
SWI5	17.95	18.8281	18.8269	18.3261
BAIAP2	67.7928	62.0175	68.4434	61.9152
HOXD8	2.73921	3.70446	3.13407	3.81397
RPL7AP66	5.33677	5.36255	4.70895	4.43899
ZDHHC21	2.0021	2.29948	2.13427	2.40593
PLEKHF2	9.14522	6.7216	7.86327	9.67164
CTD-2369P2.2	3.62455	3.37953	3.74355	4.13352
ARL4D	7.08173	5.94592	5.90531	5.95454
UBE2O	35.1682	34.4565	38.8651	36.6087
ORAI3	0.958188	2.6712	2.49879	2.41939
UNC119B	17.7487	16.9888	18.0463	18.3466
DENND2C	1.19911	0.983728	1.08152	0.994789
TUBB6	124.782	118.258	116.299	121.379
LYSMD3	6.00121	5.70553	6.69976	6.71348
B3GALT6	11.0623	13.9541	15.2123	13.8219
ZNF613	1.33221	1.78242	1.63723	1.53682
MBLAC2	1.53698	2.21409	2.54504	2.28671
TPRN	11.0956	10.6115	14.3224	9.24705
SLC35A4	30.5239	30.4577	27.4732	27.1996

AIM1L	4.62135	5.27477	5.11313	5.53579
IP6K1	17.2871	16.9546	13.5013	14.5932
SSNA1	109.196	98.0402	98.7834	99.9785
CSTF3	24.7241	24.9707	21.0562	24.5521
YES1	34.8528	33.3319	31.1736	35.5413
CHMP6	14.5463	20.0456	18.9961	18.0188
DLEU1	21.3621	17.5794	16.7912	18.2757
UFSP1	2.24871	3.34302	3.1946	2.45981
TMEM39A	29.0119	23.3491	23.1389	26.3947
TCP11L1	4.06799	3.8997	3.69634	3.7107
CCDC57	91.3374	94.5367	96.4532	89.8577
SPHK1	3.70998	3.24046	3.74971	3.30058
BNIP3	25.2999	24.6882	24.1254	25.305
MYPOP	5.71387	6.8326	6.9126	6.63022
ATAD5	1.94121	1.56305	1.38953	1.71209
SMIM19	19.5826	18.3727	14.8514	17.9534
RTTN	14.665	13.7166	15.1398	14.7271
ACBD7	1.24276	1.03619	0.834945	1.09307
ANAPC2	36.0203	35.7165	43.4387	35.8378
ZBTB8OS	72.1207	52.5159	55.2938	57.5424
SLC35G1	6.8575	5.76562	5.83429	6.77069
SLC25A53	1.35485	1.30544	1.38085	1.33095
IDSP1	1.72138	1.1199	1.36789	1.99543
COX8A	597.69	607.434	648.191	614.52
ZSCAN2	4.09029	6.17575	7.04756	6.62537
B3GNT4	5.35111	5.64174	5.62881	5.66386
CDC26	35.0219	28.699	27.1785	29.5149
HSD11B2	8.33197	8.26873	9.28427	7.82512
CRLF3	20.6067	18.6109	19.5312	20.4386

RNPEP	50.4435	55.0452	56.3731	55.5753
EID2	12.5033	10.5719	11.6344	12.2083
KCMF1	64.4613	58.0005	56.7977	63.0901
DNAJC30	2.54021	3.2629	3.16685	2.85333
SPRYD4	6.60379	6.64462	8.89904	8.10664
VPS37D	3.02581	2.88358	3.12556	2.77115
CLK2	17.3749	17.9456	19.0919	16.5317
LPCAT4	32.3414	28.5932	34.8726	33.2992
SLCO3A1	3.48797	2.22479	3.03362	2.27809
ZNF575	1.11907	1.08191	1.22477	0.993433
WDR25	3.919	5.016	3.87799	4.19133
CCDC101	14.1439	10.4075	12.3563	14.2753
PLA2G16	23.8294	22.4439	23.6489	22.8571
DIRAS1	15.882	16.9163	19.0691	17.9751
PHLDB3	8.2294	8.52691	8.87636	7.66806
GNG7	2.99035	3.95921	3.93496	3.59443
KIAA2018	2.43866	2.25477	2.18874	2.58003
KBTBD11	1.57194	1.72042	1.56694	1.63092
B3GNT5	7.90115	8.00133	7.98153	9.03765
LMNB2	204.609	169.815	168.693	178.846
RMDN1	46.5418	55.5426	52.0305	54.3165
MEX3C	19.9412	20.1564	18.9059	19.9175
MYO1D	10.1282	11.9147	11.422	10.2893
FOXL1	0.731827	1.0979	1.49234	1.00935
BDNF	18.2604	14.6855	16.6818	17.9382
SCAND2P	3.32953	2.75912	2.88367	3.14236
CCDC121	1.05637	1.05658	0.664118	0.769679
ACSF3	41.5277	41.9652	42.4879	43.6302
BOK	23.8959	26.0561	27.5389	24.0619

C8orf59	240.931	199.291	165.382	192.735
CDK5R1	5.02189	3.99487	5.13978	4.44124
RUFY1	16.2755	16.6473	13.8618	14.12
LRRC37A3	1.15844	2.16062	1.85569	1.7221
FKBP9L	2.05164	2.17352	2.15117	1.83045
VSIG10	13.9137	17.3189	16.6232	16.8487
METRNL	54.8479	51.5981	58.2663	53.4369
FAM91A1	37.5497	39.2888	38.1422	37.2577
AL358781.1	17.8797	16.2471	15.5468	17.3785
WSB2	59.8368	59.3206	53.2652	53.7399
GRIN1	5.28637	8.20974	5.76476	7.07566
TYMS	242.168	210.364	224.981	220.018
PXMP2	33.6636	31.409	32.3818	33.9555
PNMA1	15.0062	15.4874	14.5605	14.9959
MAMSTR	0.699334	0.937903	1.14326	1.14954
C18orf56	2.09509	2.01879	1.93082	1.98259
ANKLE2	42.3054	36.4439	35.8241	42.9108
C8G	0.742573	0.875473	1.14036	0.655052
FUT2	3.94622	4.74815	5.58672	5.32703
THAP4	60.3744	46.6558	47.3313	47.8154
NFATC2IP	32.1713	25.8765	27.5799	27.1837
FAM89B	40.6483	40.8122	39.6746	42.8888
SHMT1	31.8323	31.3854	30.517	32.4055
DPP7	204.639	205.926	215.794	198.881
SEC24C	37.7736	30.9413	39.666	38.9859
SMCR8	3.75478	3.27145	3.38582	3.09082
MTHFR	2.54597	4.1728	4.0809	3.37635
DEAF1	22.483	21.1009	22.8419	20.786
MTX3	6.84503	6.54756	6.36234	7.41279

TMEM80	6.25756	7.44806	7.32595	6.99344
SIX5	5.71258	7.64821	7.67616	6.0099
FBXO46	11.1956	10.017	12.1361	10.3283
ZDHHC13	12.1433	11.4324	10.492	12.2994
SLC38A9	8.68651	7.03532	8.21643	7.57765
ACER2	2.35562	2.49894	2.6888	2.26602
WDR73	22.0395	24.0568	22.0686	22.4012
POLE	50.6265	48.9447	46.8185	44.835
RHOG	59.8397	46.0094	52.0886	49.5799
EPS8L2	57.8219	71.2102	73.5592	58.9297
MRVI1-AS1	0.869409	1.64514	1.69078	1.89803
ANO6	12.936	12.4579	11.5508	12.4529
ZBTB34	2.23569	1.86685	2.1234	2.20962
LINC00982	0.853796	0.915727	1.13684	1.08151
FAM210A	25.5693	21.4464	21.3474	23.7306
TALDO1	153.539	145.403	138.367	158.34
ULK1	5.23814	7.40154	7.96971	7.16903
RIMKLA	2.11917	1.66857	1.26572	1.79649
RPS6KA3	22.3664	20.0007	20.8433	21.4555
PUS1	64.7834	56.321	59.6473	56.3074
CHD9	4.1396	3.6904	3.71952	4.14035
PDDC1	36.723	40.4422	41.2494	37.8959
AP006621.1	2.17272	2.29494	2.65486	2.55142
TRIM72	18.3436	17.7998	21.8981	18.8998
MAN1B1	32.5902	30.9456	38.1665	35.9295
TOP3A	24.2802	20.4845	22.4446	23.4414
CASKIN2	13.8869	12.8898	13.3288	13.3243
ZBTB38	11.8095	13.2893	11.4323	13.6352
DLGAP1-AS1	8.35926	10.2779	7.67305	8.48482

CCDC71	17.1625	17.5723	18.651	18.2748
RP11-551L14.1	8.48035	8.40432	7.38084	9.71492
TIMM22	31.0655	27.4536	29.2927	29.0542
PPFIA3	16.6748	17.2095	20.1714	17.8711
MAGEF1	29.5615	31.4606	32.9857	32.6941
ZFAS1	236.814	238.446	197.541	204.617
PAWR	27.2029	24.025	22.0195	22.0653
TGIF1	121.191	136.331	122.862	129.723
SMCR7	5.27878	6.09408	6.56786	5.87239
NAP1L5	1.10813	1.00915	0.681965	0.85449
C8orf47	3.49264	3.32199	3.6125	3.50608
NR2C2	5.33785	4.75204	5.0836	5.45029
PTRF	78.3438	57.9858	65.1213	66.9288
ARIH2	57.2915	49.6772	51.8295	50.0714
ZBTB33	2.68694	2.67568	2.43064	2.93293
ZBED2	19.3231	21.3704	20.8173	22.5536
SLC25A22	80.7139	69.624	79.6914	72.2875
RABEP2	8.33911	8.02478	8.05754	9.34107
ATOX1	85.288	85.449	78.8818	80.171
TBL1XR1	30.6256	22.9389	24.6232	26.2897
SAMD12	2.22718	2.79508	1.95903	2.44986
C18orf32	21.7194	24.8176	24.5488	26.7594
PIDD	18.6997	19.0434	18.9103	16.1856
RPLP2	2969.13	2883.1	2831.66	2941.26
GSG2	12.7656	9.26596	9.71494	10.4565
JUN	7.05525	8.11792	8.70322	7.69466
CSTF2T	12.8437	12.206	12.5326	12.9766
GBA	14.2652	16.8993	15.5007	13.8543
CASC2	2.1235	2.46697	2.26596	2.7836

ACAD9	24.108	21.9036	20.8969	21.6425
IL17RA	8.36851	8.69332	9.58476	8.27104
PNPLA2	55.1786	48.7197	53.9314	48.0529
AGTRAP	45.9652	50.5591	50.7764	46.8012
CD163L1	21.7359	21.0568	20.9988	20.9997
SRRM3	9.11228	9.85521	10.4587	9.96198
THAP5	13.2975	9.82893	10.2572	12.814
EFCAB4A	2.78751	3.02879	3.88016	2.76138
CD151	253.902	224.038	236.357	227.288
POLR2L	379.599	333.625	349.886	352.003
FAM20C	15.583	12.9681	12.1625	12.54
PVRL3	31.3613	32.8193	37.0346	39.5702
KIAA0195	31.2586	36.2486	40.652	36.5511
FLII	196.851	192.408	195.319	177.529
SOX12	19.8049	26.4911	26.4258	24.4802
HNRNPA0	131.968	112.936	101.972	112.285
ZCCHC3	6.76879	8.39816	8.51468	7.95036
RP5-1061H20.4	1.36519	1.18049	0.69861	1.50615
AC004917.1	6.97855	8.71817	9.40261	6.72145
CHID1	76.6235	77.3965	84.6029	83.2274
ZNF620	3.50127	3.17414	3.21011	3.42119
ZNF518A	4.72295	5.27937	4.44898	4.98175
TMEM187	3.26242	3.89933	3.49506	3.76754
CCDC23	13.6886	14.789	12.6684	13.8803
ZNF619	1.41358	1.53314	1.43106	1.48001
AP3S1	437.541	467.963	451.426	522.479
GRB2	132.595	110.196	104.346	106.493
ZBTB41	2.78967	2.53025	2.06592	2.51141
UBE2N	160.786	142.819	140.061	150.649

ARL6IP6	42.1544	34.7803	31.3458	31.8913
MAMDC4	3.20394	3.27629	4.00331	3.06828
CENPBD1	3.52928	3.5967	3.17285	3.61817
BET1L	30.2873	24.2081	25.3826	25.9192
RPS27	3110.08	3313.87	2935.47	3169.34
RIC8A	67.0023	63.0892	62.6991	60.2326
IMP3	100.172	87.8876	93.7332	90.4592
ASB8	9.16157	7.73984	9.22038	8.66921
ODF3B	1.72237	2.40537	2.38805	2.44366
FAM211B	1.88653	2.61968	2.87741	2.40059
DMAP1	17.8992	19.907	21.4225	17.5569
IMPDH2	379.082	352.376	354.222	356.606
ALS2CL	5.67527	6.86318	9.02716	8.61039
MLF1	27.3972	24.4517	19.9879	24.0877
NDUFAF3	105.227	113.33	110.847	102.338
C2orf69	11.4964	12.0571	11.3851	12.3059
GRAMD1C	1.57656	1.20363	1.00479	1.09872
STAP2	1.29199	1.65609	1.17913	1.26347
TSSK6	1.90998	1.89703	2.26866	2.11182
BOLA1	11.9657	11.0626	10.3063	9.85962
PDE4DIP	6.02076	4.87728	5.02286	5.61572
DDX10	36.0612	31.5878	32.1833	33.2733
NDUFV2	138.161	129.451	120.825	132.957
DALRD3	50.8022	51.3607	54.0719	53.2605
ZNF114	2.68977	2.73763	2.31749	2.23518
ZNF518B	9.15277	10.3424	9.14965	8.95645
LCORL	7.57968	6.73733	6.57037	7.50289
PARD6G	7.29169	8.23307	7.57667	7.67582
SH2B1	28.3249	25.7631	28.4011	27.0021

KDELC2	11.487	11.8849	11.1236	11.9393
PLEC	36.5549	40.4829	44.9446	37.5172
ZNF543	1.46658	1.87026	1.65136	1.66602
GALNT11	16.3553	16.5127	16.2881	17.5236
WDR6	78.0089	74.6281	86.7376	75.3927
GEN1	5.64343	4.89418	4.86878	5.25409
TMEM11	34.8144	30.3614	31.9144	31.1621
ZNF354B	2.87553	2.82397	2.49471	2.59774
ZFAND2A	32.4085	24.8773	24.3099	25.5682
FAM220A	13.7555	17.5669	15.3845	13.835
DNAJC22	10.6164	13.1483	12.7608	12.8555
BEND3	2.13951	1.84591	1.87371	1.78641
NT5DC1	33.4798	35.3465	33.6849	34.6862
RPS3AP5	4.86197	4.88635	3.5512	4.38271
AC006111.1	2.01596	1.9482	1.96262	1.93906
LINC00843	0.523537	0.933522	1.31723	0.443145
GLDC	1.28185	1.80662	1.57035	1.47096
COX14	37.9644	37.9416	41.5193	43.108
H3F3AP6	3.95104	3.75634	4.52082	4.76628
CTD-2192J16.15	31.3561	36.4087	18.8371	14.0605
P4HTM	22.0107	25.303	25.886	21.1123
KLHL11	2.59272	1.97866	1.83378	1.92693
CTXN1	45.5406	47.7239	52.0395	49.949
SLC25A20	12.8343	7.71497	10.7109	11.1689
CA8	1.14656	0.929288	1.00577	0.807748
EPM2AIP1	2.26034	2.88286	2.86442	2.90323
CTNNBIP1	12.3705	15.8164	15.9476	16.744
GTPBP6	29.8286	28.0137	30.9595	27.0862
ERN1	3.10077	3.03177	3.10925	3.06264

ARMC10P1	0.926871	1.11833	1.13182	1.16456
SUZ12	26.8312	22.7758	22.9878	22.9339
NSUN3	5.93009	4.17797	4.6077	4.5707
DHFRL1	0.840829	1.03884	1.15891	1.25899
RPP25	7.97172	7.25026	8.84594	8.19045
GRINA	95.1369	96.5824	108.747	90.3304
COX5A	379.298	361.787	358.877	368.259
FAM132B	9.03363	9.87581	8.46606	7.84694
FAM219B	18.1324	18.1573	17.237	14.353
ZHX2	1.596	1.97527	2.04146	2.04384
CPNE7	71.1329	71.22	82.5078	71.1852
MPI	34.6657	40.6299	40.4493	36.1491
TRIM73	2.0102	1.26136	1.35089	0.646514
OPLAH	3.11242	4.66724	4.63835	3.88068
TMEM52	32.1646	32.4809	31.9268	27.6294
TMEM139	2.73707	3.89866	4.45175	5.02017
EXOSC4	59.2003	52.3983	58.1737	52.0316
DPY19L3	3.86352	2.85578	2.62245	2.5959
TAF7	33.8576	28.2376	27.2071	29.5705
ZNF852	1.1422	1.08573	1.18225	1.24736
PFAS	31.689	33.7734	29.9291	30.4755
HYI	14.5428	14.7255	15.2946	13.7487
C17orf62	40.8117	43.5226	48.047	47.3413
ZNF552	3.48187	3.82115	3.52956	4.11585
GAK	56.1813	55.2345	55.7286	47.2204
ZBTB7A	15.5168	16.9759	18.8903	16.4149
TUFM	469.575	450.03	481.824	446.196
RMI1	13.3967	12.873	12.4814	13.7966
CTC1	25.908	26.2672	33.6319	29.6477

FBXO34	8.41262	8.57321	7.88752	8.98507
SEPW1	199.084	191.91	200.489	201.291
EIF3K	285.685	274.534	269.416	295.656
MRFAP1L1	39.2943	37.036	36.7287	38.2267
SNX18	6.80759	6.73887	6.47201	7.05969
AURKB	110.532	95.0232	87.9205	89.9709
MRFAP1	334.203	297.575	313.301	304.898
C3orf38	11.6685	9.33344	9.38787	11.5407
TMEM107	12.5115	12.3369	13.1591	12.7791
RRS1	55.2337	50.3	53.753	52.7351
TRIML2	7.29487	6.82467	5.79288	6.56503
RCC2	97.8927	100.918	101.79	104.93
DPM3	81.4316	76.5427	75.4006	69.4033
CYC1	393.48	384.762	408.434	400.216
PER1	24.1257	13.904	16.4437	14.9204
RP11-349N19.2	6.07522	6.63551	7.70588	6.79325
TMTC2	2.52699	2.11739	2.34329	2.43719
FARSA	131.628	114.856	125.983	121.233
SPTY2D1	8.08906	6.84706	7.05283	7.83092
SAMD4B	95.1117	80.4779	92.3853	84.1096
ALOXE3	2.05557	1.60284	1.86361	1.99627
EDC3	40.5649	31.1639	29.9382	35.917
TCAIM	7.2938	8.5243	7.91571	9.851
FUCA1	10.9419	14.6732	15.2761	14.4613
TMEM125	3.41346	2.87047	5.33467	2.95265
ZNF664	70.4472	62.5208	52.8268	60.5886
CALR	634.809	610.614	644.309	632.081
MAGED1	16.4309	21.7141	19.6362	21.2656
LDLRAD3	12.2287	12.1299	12.3765	12.4517

RAD23A	107.852	96.9084	105.012	105.304
GADD45GIP1	208.617	176.156	177.501	166.839
TMEM151A	3.8364	5.62915	6.1022	4.97395
C17orf96	25.6057	22.2149	23.2454	24.3576
PTPN11	52.8729	47.5183	44.8953	51.2526
FAM156B	4.81754	4.21441	4.82975	4.15482
CLK3	39.7617	32.7677	39.8716	37.8229
GATA2	9.88075	8.70118	9.19906	8.79426
ARID3B	3.16793	2.35513	3.04031	3.13867
PACS2	14.1882	15.2024	16.0866	14.6495
ELMOD2	11.302	9.86246	8.15479	10.2643
VWA1	52.6719	44.9643	49.9333	45.6657
LINC00174	1.3118	2.40963	2.31835	2.37462
GEMIN4	48.1125	37.453	40.8477	40.0137
FJX1	14.9518	13.1615	14.1147	13.8957
KLHL28	2.39527	1.70356	2.16289	2.28786
MKRN3	5.4724	5.03629	4.73793	4.85585
ZBTB18	0.847176	1.02764	1.20272	1.13092
ALOX12B	0.224652	1.01582	0.771706	0.763081
EIF3J-AS1	3.0844	3.79784	3.46791	3.55985
SHARPIN	36.6413	38.5321	37.9024	35.3808
LBX2	1.81377	2.4997	2.84667	2.32299
DNHD1	1.54617	1.90936	1.57237	1.4922
HTR1D	3.64143	2.6587	3.302	3.13042
GCC1	9.21558	9.77028	8.40234	8.79736
ZFPM1	2.94986	4.63868	4.10124	3.71982
PLD6	13.3569	10.8303	10.6128	11.1578
CDC42EP4	29.1515	25.5359	27.5015	26.8045
ZBTB42	1.91603	2.23032	2.47559	2.15135

LACC1	0.922382	1.1417	1.00726	1.09741
MAF1	93.0575	90.4707	78.2053	74.3255
APOBEC3B	7.12228	7.6621	7.44258	7.57918
PCBP1-AS1	10.5465	9.07876	9.25531	10.2673
MYADM	54.328	45.3954	51.9898	45.7187
MROH1	12.9424	12.7824	14.9257	13.121
SERTAD2	22.978	10.5903	13.1549	15.1741
RBM15B	8.24821	8.71831	8.59814	8.14756
CITED4	2.40516	3.03216	4.03322	3.24373
TIGD5	11.7959	13.2769	14.4029	12.0562
PDXDC1	40.6948	37.8912	40.2776	40.9133
PHC1P1	2.48403	2.16223	2.26905	2.56117
R3HDM2	8.80372	11.4147	12.6557	14.0244
B3GNT3	7.47107	6.58631	8.02842	7.40855
SEPHS2	36.3409	39.3386	39.3895	38.3485
ZNF784	2.68109	2.91469	2.71416	2.3384
C14orf119	66.6642	58.9992	65.2626	62.1733
BBS10	5.58384	8.31732	8.51479	8.49713
FIZ1	9.83148	10.2189	9.83482	10.0181
PUF60	158.304	138.791	146.514	136.833
DCTPP1	166.255	134.37	130.017	142.726
ZNF771	9.85235	10.7137	11.4976	9.10734
CRIPAK	2.60677	2.64685	3.20551	2.95049
TSHZ1	2.23272	2.16119	2.23831	2.20396
PSTK	4.23626	3.61026	3.62016	3.6581
SOCS4	8.16881	8.26817	8.2127	10.2963
ZADH2	13.1243	10.885	11.9196	12.9252
RP11-756P10.3	1.80705	1.99385	1.97807	1.97073
ZNF48	12.2801	10.17	11.0526	10.528

C3orf80	1.67292	1.76593	1.85275	1.86501
ANKRD18A	12.2937	13.8295	15.5794	12.0211
TMEM86B	3.46606	3.31651	3.45688	2.97494
1-Sep	2.49936	1.73003	2.01586	1.40707
TRNAU1AP	23.1113	21.1114	22.8896	24.9491
EXOC3	21.3661	20.3525	22.8555	21.4551
LYNX1	1.03022	1.86317	1.56607	1.26676
AC018865.8	1.53221	1.15421	1.03928	1.01001
MED14	21.476	15.4569	17.5899	19.4875
FAHD1	16.393	15.4277	16.5326	17.6283
RCC1	166.916	126.567	131.184	133.479
PRKRA	28.4085	24.6031	23.8122	25.7255
HERC2P3	10.8249	10.6503	11.5792	10.0942
ZNRF2	6.20458	4.70605	4.84223	5.56728
ZNF816	3.36321	2.91393	3.13434	3.4399
FGD6	2.93956	2.98111	3.05873	3.27619
OAZ2	64.4335	56.7508	66.2542	62.9705
CCDC43	20.9687	18.9778	18.1326	18.6206
FZD2	7.99707	7.73076	8.03581	6.8974
TIGD2	4.44543	4.43109	4.06381	4.37868
C7orf41	3.22262	4.57506	3.97528	3.9248
ZNF609	7.89637	7.69853	7.24453	7.41399
PAK2	40.441	35.0762	37.1225	39.9984
CCDC66	4.79286	4.02848	4.46491	4.33133
AC034193.5	2.90914	3.27941	2.80493	3.0005
MCFD2	41.3901	35.2044	33.7776	37.4175
HARBI1	10.9173	11.5229	12.3056	11.8661
HMHA1	25.2481	19.9147	23.2875	21.5225
FAM73A	3.47464	1.95716	2.06688	2.48782

NRIP1	3.84245	3.37221	3.60598	4.23598
BHLHA15	5.96299	5.17026	5.5385	4.89413
RNF182	3.16469	3.70897	3.53088	3.46505
HIST1H2AC	3.70914	3.15002	3.62955	3.77939
EIF2S3L	2.25788	2.02335	1.21599	0.814475
SRP9P1	10.6135	9.86909	8.67565	10.3981
HIST1H2BC	1.06928	0.844304	0.941458	0.973833
MB21D2	3.43219	2.53352	3.01927	3.26335
ZNF594	1.39417	1.32002	1.37518	1.55371
PCGF5	13.7798	10.9864	11.0059	13.2392
PRF1	2.68735	4.3376	5.16718	4.19511
YOD1	6.55348	7.28382	5.29278	6.12809
TMEM64	28.385	27.5031	25.7346	26.676
S1PR5	3.0769	2.74872	3.16495	3.22028
CTD-2547E10.2	10.2279	8.38081	7.9192	7.50062
GPR157	11.247	6.25379	10.9021	8.28622
SRSF8	5.54437	3.06193	4.0905	4.22806
SLC36A4	10.9765	9.89391	10.5013	9.69058
ZDHHC20	38.576	35.2858	28.296	33.0381
HOXC9	5.44649	5.98857	5.46841	5.29794
MAP3K15	2.18589	2.42154	2.53968	2.29755
PPA1	219.363	191.039	185.696	207.948
HOXC10	1.18347	1.88629	1.58413	1.75942
PSMG4	30.6932	20.5884	23.0114	22.0723
MAP6D1	8.63622	7.89037	8.70097	8.1025
ZNF443	1.13245	1.06134	1.08186	1.09049
PDIA3P	3.44627	3.24957	2.89966	3.20573
SSR4	79.7555	75.8776	74.6869	73.8715
CAPS2	1.95871	3.45312	3.32717	3.20855

CUEDC1	48.6423	50.5735	50.6133	47.5653
SCRIB	60.7075	60.9451	69.7031	60.1482
KCTD2	18.3536	15.4315	18.009	17.7557
D2HGDH	12.1847	13.0529	15.7929	13.0971
FTSJD1	11.3335	7.5554	8.78585	9.53944
FAM83H	25.5009	28.043	29.9313	26.0785
ST20	1.91475	2.39626	1.7467	2.09238
PITPNB	38.2565	32.154	30.6739	35.9691
TCEAL8	25.425	25.1859	24.9907	25.9603
LRRC57	10.6643	8.09239	8.82355	9.47409
MRPL14	96.4218	81.4444	85.4196	87.8298
BBS12	1.18469	1.12817	1.0991	1.16453
NQO1	483.122	464.314	357.801	390.901
AEN	43.7757	42.857	43.7398	41.1929
FKRP	8.50089	9.81811	10.8132	9.76979
TRAPPC5	107.303	108.013	114.081	98.8869
RPH3AL	3.17394	2.59159	3.18226	2.56337
SLC25A42	5.48186	5.3112	6.02455	6.56515
METTL23	41.4729	42.1941	37.4339	38.246
SLC26A11	6.34168	8.23554	8.72432	7.1925
HIGD1A	107.459	107.076	105.521	114.564
MAPK15	4.34053	5.08271	5.15857	4.65639
EHMT1	27.6288	28.3394	31.386	29.0371
F2R	6.55202	7.80341	8.55493	8.65922
ZNF707	3.87085	4.455	4.1781	3.80272
NPM1	3704.06	3142.45	2967.93	3502.85
PJA1	9.12274	9.19942	9.15744	9.90689
DHTKD1	4.1912	3.61999	5.96703	3.56888
HIST3H2A	10.653	13.2944	13.7458	10.8905

ZNF746	10.3812	9.53404	9.73637	9.63254
POLR2A	60.9515	66.5746	64.6239	63.2624
TMEM136	10.0296	10.2199	9.913	9.83121
FRAT2	2.68108	2.79062	2.64737	2.63878
TMEM102	7.85923	7.90068	9.75923	8.47962
ZNF322	2.8416	2.3303	2.37867	2.37277
FAM211A	65.9821	80.9804	80.0208	80.4657
DDX60L	22.7296	21.3036	24.203	26.5931
SYNE4	18.8028	19.5523	20.2793	19.6897
OGFOD3	32.6301	35.7287	36.3494	35.595
XXyac-YRM2039.2	11.6191	11.6505	12.1061	11.317
AATK	1.18039	1.30348	1.38108	1.02832
DDN	8.08905	5.86012	6.65364	6.20597
ZNF467	1.49958	1.55357	2.17213	1.65307
ZNF678	3.16047	2.52158	2.75744	3.08001
TMEM45A	6.91043	5.99376	5.29107	6.60344
RAP2B	15.5791	16.6165	15.3052	16.1108
ZBTB2	11.4774	9.1042	8.73414	10.0957
RNF135	5.77626	3.39426	4.75578	4.2489
ACBD4	1.58278	2.11019	1.97344	1.92696
SGSH	13.4672	12.4887	13.5501	11.4723
FANCB	3.52984	2.38311	2.75316	2.56347
SETD2	8.64734	7.49633	7.81037	7.84888
C6orf223	1.12092	1.7255	1.70166	1.75999
MEX3D	54.5808	52.982	54.3422	49.7168
OR52D1	2.12765	0.9379	1.30609	1.01588
MRPS23	203.021	171.819	161.634	177.984
SLX1B	24.8128	28.5628	30.1166	26.8111
ZFP41	5.6908	6.73623	6.49174	5.96709

PHLDA2	238.099	192.812	231.748	218.959
ATG9B	11.8128	13.3608	14.3516	11.9378
HKR1	5.02846	4.79252	4.52859	4.8032
YIPF6	21.1484	19.4087	18.3007	20.4156
ZBTB20	1.10744	1.07997	0.668836	0.957144
C3orf58	5.36303	4.358	4.70431	4.9646
C5orf30	3.95541	3.14402	3.5025	3.37079
GPR3	3.52192	4.32623	5.67538	4.91248
SIAH2	36.8236	18.4781	21.598	23.2763
COPG1	100.528	104.833	107.659	103.765
BAI1	0.695281	1.17197	1.35622	1.0589
LSM10	70.3421	61.4469	61.7877	65.209
RELL1	5.11732	5.29637	5.44273	5.25257
RFX7	3.49801	3.51298	3.24786	3.61084
SLC35C1	16.3381	17.0416	20.8835	18.8953
RNF41	26.186	15.7891	18.5338	18.3296
IBA57	7.52762	6.82183	8.04217	7.1048
CLDN7	101.487	100.092	104.883	104.783
ZNF329	1.172	1.15923	1.08627	1.2614
ZNF101	7.82079	8.35244	8.8441	7.46281
C5orf24	19.0075	18.7885	26.6623	25.1276
ADO	15.4273	16.0952	16.4747	16.6792
COA4	97.7157	87.4927	86.8014	90.5149
PRKAG1	56.8033	53.6735	49.2261	53.6153
GINS3	15.3759	11.1673	11.7947	13.1409
CCDC149	1.32014	1.06963	1.17204	1.44389
MRPS11	49.397	46.1584	48.9689	47.1653
SNRPE	494.106	439.79	425.073	474.015
RTKN2	2.34393	2.67139	2.25911	2.32352

CHST15	17.1128	15.0052	16.9834	18.2704
USH1G	0.817411	1.22942	1.26941	1.2692
IDH2	15.9395	21.0821	20.5036	19.2272
Z83851.3	2.12059	3.78536	3.52533	3.01252
TMEM259	164.9	149.665	173.214	150.006
WRB	20.8403	21.8211	19.2516	21.7258
TNRC18	25.8261	35.0274	35.4065	30.4867
DEXI	32.2024	34.2417	34.3312	35.5967
RP11-69E11.4	3.43708	3.6513	3.85961	3.46005
NOP10	300.572	274.769	271.909	284.172
FAM89A	19.646	17.5202	17.5293	18.3766
TDRKH	3.39093	3.29554	3.32071	3.39142
ZNF708	1.79793	1.71408	1.71179	1.89456
IST1	105.27	92.5555	101.432	97.7315
ERCC6L2	11.3302	11.0745	10.6084	11.1094
MRPL41	87.7082	77.8858	81.3407	76.8537
CREB3L2	5.61693	5.90472	5.69404	6.11335
TSEN54	95.413	78.7774	89.5344	82.4194
RGMA	2.58689	2.30806	2.36007	2.29582
MRPS16	108.631	97.3668	102.53	106.093
RAD51B	8.40626	6.49745	7.03107	5.07157
ARL6IP4	121.869	125.103	118.795	121.897
EXT1	16.9979	12.6935	16.7795	16.3276
SHMT2	205.18	215.06	201.478	199.211
MOB2	14.648	15.3544	14.3701	14.7486
ATP6AP2	55.7458	53.4542	53.5815	55.1419
CYB5D1	14.6769	12.0817	12.5772	13.4455
BACE2	13.9103	16.4488	15.5074	14.5712
UBE2E2	12.974	13.5806	14.3232	15.0525

B4GALNT4	24.353	19.4283	22.1796	19.0359
AP1S2	12.7478	9.95297	9.96835	11.6428
C8orf33	77.5558	45.4896	55.2157	62.4787
LINC00085	2.94665	3.75873	3.46452	3.04884
ZSCAN22	1.63807	1.41032	1.622	1.51482
SGK223	8.42307	8.93412	9.62648	9.14387
FBXL6	43.792	43.3441	42.1109	37.506
KBTBD3	5.28748	4.91352	3.14457	3.95209
YBEY	20.4796	19.3009	21.3653	21.1402
FAM27A	1.5239	1.02671	1.01846	0.924164
CLN8	16.2301	16.9118	16.3911	16.8574
PLCXD1	8.77301	7.28778	7.60711	6.52581
NXPH4	10.3558	10.1517	10.9227	10.1546
TRAPPC6B	8.67731	7.29544	7.89388	7.96126
NPLOC4	156.302	136.055	157.884	131.93
TEX19	1.18159	1.0605	1.04171	1.30503
CAPN12	37.7473	33.5166	26.7092	29.082
EXOC7	49.5382	49.9412	53.1819	50.389
KPNA2	222.387	173.811	169.746	178.523
WASH6P	22.7365	23.2397	24.6665	21.9127
ORAI1	9.12879	10.7483	10.7974	9.90047
CEP97	2.57574	2.53436	2.44314	2.39012
FES	3.89723	4.41861	4.40747	4.01963
GLRX5	82.7241	77.5758	73.0867	76.348
FAM104B	16.2374	15.4233	13.5497	15.0088
MXRA7	42.3316	48.7407	42.9342	44.3438
LIMK2	14.6664	12.2122	12.82	12.3307
MFSD5	27.7573	22.2197	24.3886	23.0359
ADI1	34.2251	26.1656	24.7705	26.1996

RWDD4	15.0331	14.6607	14.0876	14.0178
EPHB3	1.36769	1.63033	1.74903	1.68056
C2orf82	9.18586	8.9429	9.49988	7.76753
TRAK1	7.76807	8.23683	8.39458	8.15885
HIST1H2AJ	1.69138	1.39265	1.15871	1.57298
TSPAN10	0.88236	1.16499	1.15915	0.924622
PLCB1	0.880176	2.51693	1.85254	2.7061
SKA2	91.3773	69.4425	72.9758	73.2555
CCNYL2	1.38444	1.53116	1.53877	1.56955
FAM156A	4.65018	5.06398	5.20666	5.16104
AC073871.2	0.538935	1.25472	1.06075	1.06739
TTC3	16.3287	15.1788	16.5488	17.2715
BRICD5	6.72458	4.76734	5.04074	4.97466
IGIP	1.07021	1.54128	1.66824	1.69824
TSKU	15.5466	12.8009	13.9313	14.266
CMC4	2.06683	1.81702	1.83187	2.23297
ANXA2	1472.88	1481.22	1403.67	1462.85
HOXB4	6.87807	5.78898	6.70119	6.96947
PAQR7	8.72283	9.36773	9.88937	9.74312
MAFA	0.970938	0.946065	1.01006	0.935566
NGRN	87.7188	84.819	82.3035	85.6283
GRID1	1.80513	1.70976	1.9635	1.86841
RPS17L	1084.73	1053.2	965.551	995.236
C1orf116	2.7387	2.83151	4.14234	3.88304
TMEM198B	1.34274	1.88751	2.1775	1.73033
MAGEB17	2.28929	1.79526	2.01597	2.01903
CRIP2	13.4433	15.3359	15.8893	13.1929
DDX28	11.8428	11.1371	11.3205	10.7272
ACBD3	13.819	13.0347	14.2097	15.3924

C16orf72	12.1361	10.1973	9.44997	10.3781
RRP7B	12.1041	7.20685	11.3434	9.18565
ALG12	13.954	12.4377	14.7431	13.1991
COL18A1	41.0134	46.278	49.0187	43.237
RBM10	19.9973	18.1829	19.4332	18.3607
RP11-181G12.2	0.983628	0.96114	1.25308	1.0235
RPL35A	1225	1266.71	1122.85	1304.87
ZNF721	20.0155	19.3287	19.5667	19.1734
LENG9	0.222655	2.02336	1.87516	0.115134
C11orf54	12.985	12.1865	12.5667	14.2816
CEP63	7.88335	10.4867	8.76825	8.72078
SRPR	51.3862	45.1055	50.6906	50.1329
EWSR1	315.031	265.335	293.541	268.215
HMGN4	45.2723	44.4619	39.8666	49.4961
SPATA13	4.63885	4.73354	4.21799	4.45313
GJC1	18.3095	15.3901	17.2614	17.2959
CNOT10	33.5479	29.7655	26.4256	26.1909
MTA1	48.9508	59.7072	62.4018	50.0296
ZNF320	5.09563	5.45006	5.92172	5.74425
C12orf60	2.61732	2.37628	2.63172	3.08835
PYCR1	292.478	325.563	301.375	275.564
LSMD1	228.813	193.004	188.088	186.806
SPNS2	2.29082	2.48363	2.42846	2.08391
AP2A2	65.6836	64.2928	73.8332	65.6411
SLC25A21	1.41444	2.93663	2.14082	4.3834
ABAT	1.06478	1.38545	1.19372	1.24737
SLC25A10	70.3147	79.0213	82.0895	76.506
CAMK1D	1.89764	1.78386	2.0739	2.39269
RGPD6	4.36392	4.42943	4.48491	4.56734

LYSMD4	3.91288	4.18653	4.0359	3.66007
AFMID	58.2032	52.2709	53.6097	57.6763
GATSL1	1.26437	1.01985	1.19486	1.10462
GAS6	31.5415	32.026	37.3808	34.9429
BEGAIN	3.18631	3.30616	3.89866	3.47262
ARHGEF37	0.98642	1.39248	1.22101	1.0852
CEP57L1	11.5439	9.93307	11.6316	11.7452
GJD3	3.4928	5.39305	4.72613	3.99959
RABIF	5.65819	4.37339	5.28421	5.10537
FANCF	1.93783	2.13582	1.85833	1.94523
SMDT1	18.6123	18.3786	14.9407	13.9533
C2CD4C	2.58858	2.87828	3.00757	2.84107
CHST6	3.63889	3.989	4.06059	4.01141
RUVBL2	270.302	244.744	243.642	239.141
GDPGP1	3.01512	3.20179	3.25858	3.05635
MICA	22.2653	17.5946	16.8313	18.3282
CTD-3193O13.9	0.957078	1.13815	1.29981	1.33671
C21orf67	1.1504	0.949024	0.736084	0.730056
OR51B4	12.4131	13.156	16.9152	15.5291
PTTG1IP	48.6363	47.1079	45.8588	47.9645
DDX41	95.3318	87.5031	89.5881	82.9812
ABHD16B	1.71125	2.97116	2.85775	2.03186
DAZAP2	209.971	203.535	235.952	219.692
RP4-604K5.1	153.526	144.395	114.367	137.644
MAGEA2B	32.535	30.2243	32.3849	31.3478
AC005037.3	2.18454	1.78876	1.40706	1.80182
ZNF623	5.12019	5.78593	6.62364	6.70675
TUBB	143.952	117.22	134.689	135.807
EPHA10	1.59534	2.35126	3.15703	2.81289

CCDC125	3.6176	1.86787	2.76473	3.3066
BOLA2	16.2555	13.0714	12.0896	12.7129
BCOR	7.86345	7.14072	6.12434	5.94038
JRKL	2.44929	2.48524	2.89939	3.00742
KIAA2026	3.29823	3.37075	3.59633	3.77646
FHL3	17.0248	16.908	17.592	17.3775
PMCH	6.58827	5.01507	4.87864	5.95655
C19orf71	3.13708	3.17669	3.89465	3.48521
CCDC159	5.91866	5.72844	5.77058	5.55216
RPS7P1	1.84375	1.79174	1.25942	1.6274
RIPK4	4.2453	4.13579	4.74508	4.28616
NPIPA1	15.956	18.1531	17.4929	14.9394
SF3A3	142.675	106.927	112.673	128.71
AC004967.8	11.0675	13.1535	17.0865	15.1306
RP11-958N24.1	8.76689	9.68132	9.38117	7.49607
GTF2H2C	25.1617	24.6704	21.9197	25.9137
ASB7	4.36278	4.37076	4.45321	4.47953
EP400	6.78856	7.10939	7.23381	7.19078
MEX3B	0.860022	1.14585	1.21081	1.06351
PI4KAP2	8.5278	8.8749	9.03294	8.66041
COA5	12.9019	14.3636	13.0568	14.2646
UTP11L	57.9606	45.9788	45.678	50.7902
PSMG1	137.78	111.146	110.84	133.339
PRR14L	10.0502	6.3731	9.86358	10.3699
Z98256.1	1.09512	0.655608	0.743411	1.02419
HIST2H2AA3	0.79594	1.04931	1.00549	0.883074
SERHL2	2.14606	1.80616	2.10332	1.87556
PCBP3	0.740006	1.15641	1.14623	0.928609
RNF5	2.60532	2.89108	2.86084	2.97358

SETD3	24.6696	21.9647	23.3488	25.0674
ZNRF3	6.13496	6.59777	6.5604	6.22214
TANGO2	12.9309	10.7014	17.4207	12.4788
RP11-347C12.2	1.3933	1.54493	1.5674	1.41095
SFXN4	68.0708	67.2244	63.6996	74.8677
MRPL54	49.9932	46.3297	44.8317	52.5248
C3orf37	63.6076	49.4123	53.9857	53.2093
DGCR6	10.4974	8.5666	10.4696	10.2815
ZNF530	2.79297	1.68986	2.00566	1.68373
NDUFB1	196.244	179.231	178.288	184.162
KLHL25	2.90151	2.82093	3.13344	2.68576
TRMT12	12.2107	11.0405	11.0437	11.3726
BMP8A	1.6823	1.58699	1.72265	1.7819
ALYREF	442.436	346.826	336.066	378.979
FAM101B	10.6048	9.82827	9.70955	9.95094
UPP1	25.333	23.991	21.0931	21.2719
TRIM52	3.43354	2.3572	2.98757	2.44356
CMTM4	20.6754	22.9447	24.8243	26.8675
TMEM50A	53.0161	50.8837	46.4048	52.2905
TBK1	16.4973	13.5519	13.9674	16.8135
CBX6	43.284	40.7771	44.2188	42.5041
MACC1	1.22912	1.67724	1.11999	1.70099
TBL3	94.3939	82.0818	87.5049	82.2079
PAPL	1.36467	1.36301	1.23217	1.37488
KREMEN1	6.53508	5.99841	7.67615	7.19515
TRAIP	17.8687	15.2585	17.4748	17.105
CHEK2	23.1747	21.3637	21.5136	23.5363
AIFM3	1.53287	2.3489	2.28856	1.9352
ZNF703	3.35591	2.96294	3.15743	3.05892

SLC35F3	5.25082	4.1488	4.60754	4.91259
TUBA8	1.10623	1.11648	1.06234	0.638928
NPIPA5	7.28057	8.66292	7.62506	8.09646
RBM12B	15.8217	13.2737	11.2666	12.5481
LIN9	5.10615	4.31069	4.2187	4.72229
BTBD9	2.77196	2.32328	2.78695	2.67178
NUDT14	19.7554	21.9873	25.9625	24.8635
KIRREL	10.2595	9.53348	10.3809	10.6658
IQGAP3	8.90166	9.74822	9.08075	8.48772
TOB2	18.9551	18.0766	19.1399	18.2635
SCN5A	6.13074	5.75721	6.15242	4.93745
AC138969.4	30.2073	29.3556	27.0025	27.6464
TTC32	2.9342	2.93892	2.68273	3.07834
AC099522.1	11.0823	9.29327	9.99136	10.7145
PRKX	14.5101	15.1675	13.5265	15.9053
SETD8	39.368	24.4114	29.6068	35.4154
SMTN	54.0025	45.7465	46.1156	47.3977
NPW	170.7	159.692	165.137	151.187
COA3	47.9287	48.4931	47.9373	51.9587
PTP4A2	212.047	180.133	176.354	203.768
ACTG1	4142.94	3443.24	3357.16	3163.67
DENND5A	31.0703	31.7836	33.0846	32.1866
DIABLO	83.8395	85.1434	86.2424	86.5687
VPS33B	19.9523	18.3444	21.5188	20.9494
ADAP2	7.00593	6.38856	6.24588	6.60247
RP5-821D11.7	1.45414	1.37681	1.55972	1.5979
UQCR10	170.107	155.674	152.497	152.512
EIF3C	308.353	280.173	275.304	276.346
NIPSNAP1	40.1672	54.1562	54.0224	52.6724

LRTOMT	12.57	12.0979	10.9309	10.5423
ADRA2C	19.1667	17.8557	19.0238	19.3128
NR2C2AP	47.652	43.7266	42.1893	45.9714
FAM132A	1.50748	1.68619	1.66173	1.5679
CRELD2	41.176	38.6112	45.6918	41.2322
SCFD2	8.1332	6.58825	7.10738	7.87904
UBE2F	34.9211	33.2282	30.5933	33.2
KCNJ12	3.54913	3.38564	3.71342	3.61083
PPP1R2	34.8086	28.975	25.8698	26.8097
TSPYL2	4.77881	4.21091	4.35585	4.08094
PGP	43.2731	35.4615	38.8595	37.0443
C22orf46	3.35293	4.96473	4.87615	4.97831
SNRNP35	11.6925	9.15898	9.04399	9.5821
IRAK1	53.4061	41.9986	40.1951	38.1698
CMSS1	47.302	39.1551	36.7288	42.9251
C11orf72	1.20618	1.26129	1.41012	1.31387
OAF	13.1186	17.0912	16.7528	15.3244
ALDH1A3	193.945	145.339	207.605	205.317
HIST2H2AC	2.58256	1.76133	1.59402	1.28899
POU6F1	1.5395	2.47379	1.98785	1.21203
TM2D3	26.0155	22.7611	23.7767	25.5641
TSSC4	62.5052	51.504	50.5141	47.4039
TACSTD2	2.77157	2.29718	3.02466	3.10073
CCSER1	0.918953	1.19716	1.06222	1.17257
ZDHHC23	6.03776	5.33731	5.4888	6.18716
AC002055.4	17.3773	18.8655	16.3557	17.6045
CSAG2	6.21439	5.52764	6.10629	6.32622
PKP3	54.3378	50.0378	60.1542	52.3187
CSF1	1.13732	1.63689	1.45097	1.50179

ACTRT3	1.34766	1.43009	1.50507	1.59521
PLA2G6	5.14501	6.95564	6.67581	6.15898
SS18L1	12.1478	10.098	12.1427	11.0347
TOP1MT	26.1687	23.7952	26.2271	24.7308
COPB2	87.6488	78.365	84.0818	85.435
THAP7	33.1367	33.4765	33.1616	31.448
KNTC1	27.0361	23.9714	25.0403	28.5752
WDR27	10.8285	13.3993	13.9887	11.1874
TXNRD2	13.2917	13.6875	14.3727	13.73
FOXO4	4.19325	4.60529	4.13377	2.98476
PTP4A3	3.968	7.63512	8.10134	6.72066
NEU1	1.71734	1.13789	1.506	1.73678
TMEM255B	1.54239	1.51417	2.10552	2.04599
PROS1	2.54009	3.33111	3.05735	3.41903
HDDC3	14.4109	18.9479	17.3459	18.2911
ZFP1	8.75034	5.24713	7.05065	6.78146
DUSP8	5.76345	5.77471	6.25231	5.93786
AC132872.1	4.37021	3.53536	4.10132	3.69217
SOCS3	10.9749	8.89686	10.1291	11.2466
LPAR5	2.28454	2.6922	3.32072	2.8788
XPOT	51.1764	45.9226	43.5839	45.2621
TMEM173	8.38803	11.558	11.7204	10.8081
PDE4B	24.4421	28.0514	29.5378	28.8681
SNN	8.02074	9.11162	9.58956	9.63174
MED12	11.6708	12.5979	14.5952	14.7772
9-Sep	158.95	158.827	176.608	172.563
CDCA2	19.0022	15.4129	15.2432	17.3181
OR7E14P	0.420125	1.08668	0.803791	0.842246
AMER1	1.1016	1.15144	1.11625	1.17383

ZBTB40	5.69306	5.0186	5.11978	5.38792
5-Sep	6.661	5.75758	6.09891	7.10453
EIF4ENIF1	12.5401	13.4847	11.9367	13.2781
LRRC26	1.51032	1.57302	1.74338	1.61673
SERINC4	2.48531	2.3982	2.39057	2.66772
FAM110C	4.61552	5.47165	5.78255	5.51052
ATL3	10.5806	11.5543	10.977	12.2253
MAGEA2	22.7473	19.9015	21.9999	21.1212
NDUFA12	76.0338	73.9989	69.5285	81.7315
RPS17	1068.86	1037.52	950.826	980.893
UBE2G2	50.9418	39.294	44.2282	44.7413
OSBP2	8.07928	7.03891	8.62858	8.12137
APOO	27.9516	21.917	19.689	24.0214
TMED9	177.064	163.56	169.979	163.682
TMEM186	11.4376	11.3552	12.068	11.995
SDR42E1	14.3968	14.4958	14.5515	15.0004
RBM33	10.311	8.98426	9.67535	10.0591
OR51B2	3.34139	3.669	3.66831	4.44513
PIGW	11.3977	11.7828	11.6937	13.2219
BTBD6	38.7546	33.0652	34.3726	33.4288
H1FX	74.3401	77.8825	70.5939	63.2651
RBM43	0.614994	1.09185	0.837348	0.88442
SUMO3	29.3851	27.9675	27.9914	28.5127
IMMP2L	7.09012	9.66531	8.5297	8.91926
JAG2	66.0932	51.0741	60.8687	58.2848
FMNL1	1.971	2.00037	2.17923	1.72918
PTRHD1	30.6852	31.0163	31.479	31.9182
ZFP90	5.87851	6.68968	6.75233	5.98505
NOC4L	48.3441	43.5416	46.5524	41.8874

USP18	3.1773	2.97583	3.45086	3.46979
NDUFA6	39.5019	41.7106	35.4201	36.9167
TMEM121	3.75879	3.06121	3.39964	2.82856
SIVA1	197.364	185.991	182.882	170.624
BRI3BP	20.8999	18.135	18.7839	20.4752
DGAT1	41.5313	45.8014	46.7923	40.5381
AP3M1	22.406	22.4566	21.7922	24.0982
CA13	3.1538	2.83908	2.9049	3.23195
UBOX5	3.95831	4.27855	4.4089	4.1649
MAFF	8.29951	8.00012	8.24569	8.0204
BRF1	18.9815	17.3903	19.1208	18.071
SEMA4B	17.6511	18.1951	19.3031	15.7836
CIB1	61.7575	59.1652	60.8849	56.8339
ANKS1B	1.13717	1.20079	1.06389	1.02101
NELFA	42.543	37.1939	39.952	37.5901
EFCAB10	5.64812	5.24258	5.41503	6.60409
INTS5	21.5554	21.1814	22.3653	21.6554
RPS27L	107.463	150.695	128.146	149.617
MANEAL	16.2131	15.2328	16.128	15.3188
ADSSL1	1.44267	1.8499	2.45413	1.65421
ANO9	9.29167	12.3021	13.2521	11.5219
FAF1	30.2165	27.6104	27.4849	32.3586
FAM43A	3.65927	4.17221	3.94696	3.51929
NDNL2	14.1126	14.1694	13.5987	13.2695
HSF1	55.8479	49.799	51.5332	45.8288
C6orf120	13.2724	12.9093	15.6358	14.245
TBC1D3F	1.70208	1.9848	1.96141	1.53529
PURA	8.02538	7.85232	8.97758	7.97947
INPP5J	1.98046	2.87121	2.5879	2.1393

LRRC37B	3.8044	3.30804	2.85391	3.4564
DDX51	29.8681	27.9616	29.4136	28.7782
NOMO2	38.9525	38.315	42.2807	40.7468
LINC00482	0.925516	2.03067	1.25678	1.38888
SIGIRR	26.9292	26.8517	31.481	27.8252
NRBP2	2.88882	3.90758	3.59725	3.1044
IFITM2	117.649	110.713	132.606	131.591
TNFAIP2	29.7573	32.1342	36.0967	37.683
ZNF445	3.36766	3.3382	3.33806	3.41762
PGBD2	3.20674	3.39991	3.11669	3.12006
WBP5	18.6384	18.0144	17.6119	22.1703
RAB11B	73.5496	78.9502	86.3443	77.5316
PRMT3	15.4957	13.3479	12.2324	14.229
PRPF39	16.5691	15.5735	14.0012	16.4014
ZNF74	7.0796	6.39454	6.78399	6.25659
UBALD2	35.5977	38.8503	29.0415	29.1461
NOTUM	7.91896	9.56149	7.78527	7.87584
CCDC137	62.3762	50.3579	56.0742	54.8027
ARL15	6.5531	4.49218	3.92392	4.51557
CDK10	58.1268	59.0072	63.7483	59.4293
TMEM105	1.46259	1.05925	1.51759	1.81418
SOCS1	2.51418	3.38067	4.02412	3.59011
TCN2	1.06007	1.3608	1.53864	1.42209
GAS2L1	25.3284	24.983	26.9589	24.8722
ATP6V0A2	12.0823	11.1926	11.141	10.159
C14orf80	23.9433	19.6686	21.6858	18.3483
HGS	219.825	207.682	210.981	195.293
TNFAIP8L1	8.46818	8.37344	8.05889	8.30902
RAD51D	6.88052	6.26161	9.11309	7.75861

MAPK11	9.47399	14.0876	16.1884	13.476
SP140L	3.68451	5.11889	4.94127	4.7708
MRPL30	13.5234	8.4196	7.03332	9.55064
TARSL2	4.45684	4.43956	4.42053	4.93547
SMYD3	13.8778	16.7716	15.6371	17.6425
IFNLR1	3.98521	3.12349	2.98652	2.86686
SH3BGR	2.84534	3.66654	3.84713	4.16228
C19orf68	3.18419	3.46132	3.92546	3.42658
TMEM179B	56.6954	71.273	71.118	64.8558
PARPBP	14.7211	11.8224	10.7921	13.12
STAC3	1.14873	1.58426	1.14294	1.44703
SDHAP1	13.4872	11.246	13.5165	13.1775
MUC1	1.01899	2.77187	1.38765	1.136
C17orf70	25.4524	29.9785	31.8505	28.6949
IRF7	3.63582	3.8956	3.91138	3.65701
L3MBTL1	3.73312	3.61733	4.16512	2.93011
BRCC3	6.50513	5.30053	6.07375	6.33721
FAM131C	6.56727	7.25516	8.23233	7.61565
C11orf35	1.16788	1.71061	2.09968	1.47372
NR2F2	66.592	51.9273	56.9386	54.818
AHNAK2	2.58708	5.18567	4.87189	4.5092
OLFML2A	0.880024	1.23586	1.14548	1.16455
SP1	18.4315	17.2831	16.2913	17.5377
WASH3P	29.1066	30.4898	29.9197	27.014
MRPL40	56.4306	49.5163	49.1163	50.2588
FAM212A	2.07596	2.379	2.40084	2.01034
PCGF3	24.5536	28.0614	27.9818	26.2833
LMLN	1.7383	1.95028	2.09052	2.21671
P4HB	1394.18	1279.44	1428.42	1347.68

PSMD13	230.629	209.953	221.441	248.528
PBX1	1.95699	3.11106	3.48145	2.95413
CTD-2287O16.1	1.12448	0.675712	0.289894	0.217856
ZFP36L1	104.534	112.087	134.864	117.506
UBE2L3	96.618	82.0152	78.5397	94.4309
BRWD1	9.00398	7.56155	8.06816	8.74364
PMEL	2.33184	1.61655	2.25463	2.22023
SNAI3	0.756312	1.02375	1.08844	0.969498
ZBTB3	2.52776	2.36782	2.23664	2.21863
EP400NL	4.67127	4.61654	5.88636	5.11369
PRAME	22.2345	19.0739	21.2062	21.3768
MYBL1	4.03409	3.70307	3.40837	4.12698
RP11-645C24.2	4.94738	5.10925	4.5922	4.68469
C16orf52	12.1571	11.1438	10.7464	12.6521
DRG1	94.3673	83.4588	80.4035	89.7945
ANKFY1	10.9775	9.97459	10.5596	10.4869
YTHDF3	25.8681	23.4843	21.5769	23.2432
ZNF696	11.7566	11.545	12.0246	11.126
CXorf38	6.74065	4.86028	6.43538	6.24525
ADAMTSL5	12.0437	10.9722	13.8692	11.9508
MORF4L1	279.952	200.415	208.658	243.13
WDR53	13.5893	10.1481	9.90342	12.6221
DMWD	19.6194	19.2303	21.0303	19.2812
SLC52A2	73.3134	69.5836	70.5223	63.3602
PIGP	16.2204	18.8777	16.6748	19.1753
PCYT2	42.9419	35.2251	42.4921	39.6035
NAT8L	5.44703	5.52995	6.23993	5.60392
BCAP31	78.2368	71.576	72.8256	72.6155
ARL17A	3.29026	4.25184	4.13238	4.60912

GNB1L	9.67176	8.12527	8.57197	8.00691
DNAH14	11.8601	11.5686	13.1975	13.0084
RP1-46F2.2	13.7473	18.2543	14.7612	15.291
NPIPB4	31.4353	36.5194	36.69	30.3789
THNSL1	3.44824	3.79059	3.58576	3.89422
TRIM69	5.27793	7.79112	6.73081	7.34356
ATP6V0C	186.081	183.154	195.939	174.183
IFITM1	2.36709	1.51309	2.23404	1.66214
LAMP1	143.294	148.289	140.895	135.242
SGK196	5.86469	3.83314	5.82014	6.16044
LINC00839	6.29175	5.35553	5.39297	5.38437
KLHDC8B	13.3969	11.4897	10.2529	10.7191
SETD4	15.4383	13.6364	13.0291	12.9924
PTCH1	1.48841	1.79015	2.60741	1.80465
RTN4RL1	3.01643	2.47507	3.31925	3.00279
PAGR1	29.2709	24.3316	27.2537	26.2551
RNPC3	4.68495	4.17004	3.87797	4.40509
ZNF267	6.68456	5.5361	6.2699	6.06731
IRS2	2.53161	2.60503	2.52645	2.41541
BICD2	14.807	13.6641	14.5041	14.0393
TMLHE	3.52036	2.66562	2.88816	3.44
SDHAP3	3.06904	3.06139	3.1983	3.03339
RASA3	7.17701	5.90144	6.91051	6.87686
F8A3	2.83762	2.79936	3.01304	2.98069
LRCH3	19.3719	18.0267	17.8098	14.1023
NDUFA13	120.705	144.677	148.09	133.02
ZNF566	2.36994	1.53511	1.77899	1.58618
AC084219.4	1.4513	1.74103	1.61122	1.55547
AIDA	34.938	33.9626	33.7903	36.2699

C15orf41	7.15954	3.88912	5.07396	5.47429
RP11-887P2.3	75.6842	74.0545	76.7752	68.8917
CYP2R1	4.3958	4.54753	4.5889	4.38261
ANKRD46	9.89932	7.99457	8.99678	9.09383
PIP5K1C	10.1902	11.391	11.9653	11.213
ZBTB6	6.05891	5.4598	5.70011	6.31998
C2orf76	6.2408	6.02591	5.43059	7.30934
POLR3C	10.8388	9.49201	9.8191	10.1727
WWOX	7.27104	6.4147	6.54969	6.86886
CIDECP	5.79751	4.82909	4.75545	4.39156
CCDC84	9.01738	8.55501	8.09933	7.30149
BCL9L	49.9338	39.4327	47.8928	49.55
POLR1D	126.963	108.915	115.941	120.829
KIF18B	32.5272	22.7441	23.1099	22.9891
ZNRF1	30.091	29.0466	29.6231	29.0142
SAPCD2	66.0511	53.3843	54.4259	53.1603
CYP4F12	0.580473	0.809476	1.03217	0.840137
1-Mar	13.1599	12.1036	11.5281	13.1176
BLOC1S4	10.6981	12.3055	11.9572	11.2022
ZNF749	6.37907	7.34035	7.99197	6.20493
MKL2	6.25415	5.87218	6.60965	6.54147
ZNF17	2.0272	1.70897	1.74878	1.80358
NBPF12	17.8539	24.7583	22.3381	17.8383
GPAT2	26.0405	27.6371	25.8113	25.6582
TOR3A	44.4804	37.5397	45.0603	39.0457
PPP1CC	186.245	148.522	147.238	168.899
ZNF555	1.08442	1.58954	1.1016	0.952138
MST1P2	3.48765	4.12833	4.61011	3.68418
CA5BP1	11.6017	10.2877	10.7299	11.1079

PRELID2	6.22623	5.66184	5.04619	6.40642
BACE1	3.19	3.71259	3.6001	3.30268
RXRA	22.4552	20.9117	24.6389	23.2789
ANKRD37	2.80468	2.63224	2.52779	2.07933
NUDT17	0.832986	1.03967	0.859134	0.753338
KRT10	13.0678	11.7723	10.5788	14.3885
NKRF	13.6891	11.575	11.8861	13.418
KPNA4	35.4571	21.672	21.1277	23.1147
ZNF197	7.49958	7.47226	7.41415	8.02227
RPS23	1900.01	2009.53	1840.96	2051.89
BTN3A2	3.57358	3.75461	4.015	3.76722
INSIG1	17.8111	12.7561	13.1252	13.8951
ANKRD20A5P	0.838436	1.18396	0.667477	1.05592
TMEM222	28.666	28.7809	29.2507	28.7915
10-Sep	62.8172	58.397	56.4955	52.2487
FAM86B1	4.90772	5.92673	5.9087	5.28251
CYP4F3	1.29743	0.921322	0.718843	0.850942
SMYD4	4.93106	4.27258	4.52519	4.75153
FOXD2	1.01506	1.08144	1.1451	1.1016
GPATCH8	7.41203	7.12671	7.22046	7.32103
CEACAM19	1.1754	1.14203	1.19705	0.895215
NF2	28.3522	22.277	24.7722	26.6529
C6orf1	16.5387	15.5974	16.4282	15.8049
UBE2H	45.4044	51.3927	52.2272	51.702
MIR22HG	1.89693	2.13033	2.05097	1.8185
KTN1-AS1	3.41516	3.31727	3.09192	2.77675
KATNA1	13.8303	12.3137	12.3085	13.9282
ARAP1	21.5515	22.7736	26.3751	22.33
KIF24	1.53696	1.33445	1.3911	1.50621

PDE2A	2.55549	2.47866	2.7092	2.00765
PRR5	24.8637	22.236	24.5778	22.7923
ZFP91	28.6532	20.42	20.3279	23.0911
C17orf58	15.8079	15.7586	15.0024	14.4974
BCDIN3D	4.51703	4.59921	5.30329	5.16267
LYRM7	4.09681	5.15493	4.77035	5.17729
DTX2P1	1.54961	2.13353	1.86586	1.56826
CCDC73	3.48266	3.33443	2.62239	2.95071
MST1L	4.71149	5.3736	5.56895	4.86939
BCR	34.8221	34.3441	37.0466	34.3527
FSCN2	0.724397	1.08862	1.20948	1.09836
SPIN4	1.74579	1.69002	1.85621	2.03275
SPIN2B	3.32803	2.36568	2.8533	3.19158
HYAL3	2.47096	2.5133	2.67105	2.96377
VSIG10L	2.53252	2.97492	3.3555	3.07873
ZNF397	6.40064	7.12954	6.74434	7.79034
ZSCAN30	2.12236	2.17405	2.33888	2.24454
TPCN1	7.43127	8.25008	8.00972	7.03649
HEXIM1	19.7891	17.2075	18.2344	18.8248
TRABD2A	2.09187	2.34957	1.95189	2.19642
PDZD7	2.45745	2.59807	2.9523	2.81404
POFUT2	11.9243	11.5512	12.7727	13.4204
MAPT	7.1136	7.66895	7.54649	7.57009
ERCC6L	7.41384	5.79266	5.72144	6.29407
TMEM17	1.61612	1.8755	1.80414	1.87461
TNFRSF18	33.7017	30.8301	32.8059	30.0916
C1QL4	2.64224	3.42363	3.93988	3.24428
RTN4RL2	19.5803	22.5669	25.0638	22.1572
ZDHHC17	8.66318	8.25289	8.17394	9.12836

ZNF395	15.3753	13.6925	17.8466	15.0876
CHCHD2P9	5.23582	4.51631	6.18457	4.03508
PPARA	3.17213	2.30914	2.73456	2.68474
KANK3	7.14898	10.1758	8.76301	7.33862
EMID1	3.69609	4.74255	5.64374	3.87908
ESPN	34.2945	39.6946	40.9127	34.2546
PTRH1	12.1959	15.5426	16.4841	15.2376
TMEM216	27.7762	25.0113	25.2068	25.3821
RPS19BP1	136.395	106.647	110.793	112.721
AP003068.6	2.57613	2.46513	2.88368	2.70912
TEAD1	19.7757	17.5287	17.2288	18.975
PLCD1	3.94299	3.50468	3.53748	2.97051
ENTPD5	7.04671	7.47249	8.353	8.34901
NAP1L1	256.805	251.763	222.542	245.143
CMC1	35.9687	36.2318	33.1266	40.3586
LYPD6	1.37322	1.02421	1.00525	1.22845
SPATA21	13.3487	12.1455	11.7965	12.0013
MRPS21	79.6798	73.2893	68.7629	67.8342
RNF220	53.7403	52.264	55.5392	51.5922
KIAA1598	19.0182	16.4029	15.1537	17.0732
RP11-195F19.5	4.03807	4.8537	5.23219	5.08545
TSPYL4	2.44095	2.74678	2.95367	3.07805
MT1X	104.025	138.007	132.464	124.606
GCNT1	2.99588	2.55652	2.89699	2.77715
SESTD1	6.38222	7.82471	7.67363	7.21073
FNBP1	8.69554	8.05355	8.26253	9.20081
BCAM	20.9948	22.4243	24.7119	20.8761
RSBN1L	9.15211	8.59211	9.22916	8.63137
WDR86	1.37776	1.13116	1.13102	1.33979

EPOR	6.17945	6.40778	7.49815	6.53508
TAF9B	8.97069	6.93317	7.16099	8.51751
CHP1	35.9396	34.223	37.1907	39.0179
RDM1	4.06617	3.4972	2.84425	2.93376
KCNJ11	2.17883	2.1926	2.64891	2.14269
PTMA	2113.4	1669.96	1675.22	1833.75
HSPA14	43.0561	34.6971	34.1601	37.4702
SIRT7	45.8372	44.3264	54.2081	47.3063
CTC-471F3.4	43.6939	42.8228	48.0883	50.0445
IFT140	4.97099	5.33694	5.51643	5.32042
USP7	118.528	95.0242	90.0641	106.404
PLEKHN1	3.47915	3.64785	4.15672	3.61174
TET3	4.87579	3.65567	3.37908	3.75383
ZNF286A	18.4054	18.3389	18.2802	18.2231
ISG15	121.902	111.868	125.354	111.77
EXD3	2.65618	4.62238	4.68896	4.15476
C17orf97	0.946345	1.28195	0.99303	0.945981
ZKSCAN4	2.12046	2.15219	2.43679	2.00244
DHRS4L2	19.1124	19.3345	20.2016	18.2252
SAMD11	0.787373	1.38826	0.736326	1.21204
C1orf170	1.83633	2.03334	1.98942	1.88664
TMSB4XP8	7.99366	10.4982	7.98517	7.71836
B3GALTL	3.07991	2.32565	2.61385	3.09807
SPRY4	22.4994	20.8326	20.5458	21.413
TRPV2	0.954367	1.85678	1.77516	1.19687
C2orf88	1.84385	1.37959	1.84315	1.85953
TMEM203	59.2256	55.5045	57.1595	55.8781
THSD4	0.98917	1.42466	1.72418	1.64783
GABRD	7.48455	9.80021	11.3807	9.15987

TCEA1	60.6367	57.3194	54.4491	58.4776
NHEJ1	11.6555	11.0958	10.4122	8.69397
FANCA	93.4989	86.4001	83.7685	83.2495
SECISBP2	19.9977	24.2676	25.4286	24.7272
SEMA4D	7.06128	7.55512	8.763	8.44356
MCRS1	90.5384	81.5982	85.8969	85.9289
FANCM	4.84161	4.33511	4.42806	5.32194
ZNF70	1.06043	1.06267	1.24454	1.1094
PEAR1	3.91978	4.15875	4.55872	3.85753
HIST1H1C	8.83997	9.10893	8.58267	8.98498
PLSCR3	36.133	36.1975	38.0975	35.0812
EIF4EBP1	103.825	127.412	115.267	108.522
CCDC157	2.95631	2.63211	3.07381	2.93869
FAM122A	3.14362	3.24525	3.43664	3.44056
PALM3	3.27765	2.66679	2.45469	2.57089
DOC2B	1.48961	1.4716	1.77805	1.85279
ARHGAP11B	11.2136	8.69039	8.72725	9.88277
HS6ST1P1	1.34014	1.48486	1.82335	1.57636
PMS2CL	9.34853	9.41022	10.2684	10.0469
CYHR1	21.7067	25.441	27.4185	23.2927
KLHL17	45.59	42.4288	45.9711	39.3893
ANKRD19P	7.62025	9.0218	7.9024	7.97949
RINL	8.61179	16.6871	16.7107	16.275
RP11-43F13.1	19.5885	32.0161	27.14	27.8278
MORN2	11.1976	11.2509	9.26106	11.354
S100A3	8.13628	5.14322	7.88698	6.5671
UBQLN2	13.6569	11.0288	12.3146	12.6428
RILPL1	4.19889	3.91216	4.38332	4.36694
ARL4C	2.55731	3.24979	2.795	3.0646

TMEM221	1.31177	2.33942	2.25946	1.80027
WNT7B	4.55133	2.90457	3.90147	4.11741
C11orf95	5.50464	4.87133	4.48547	4.45122
GPR89B	3.86673	3.05737	3.14278	3.69609
FAM25A	2.49927	1.94268	2.02969	2.91717
C6orf132	7.34233	7.74534	7.94723	8.51446
MAPK12	45.3181	51.2175	52.9392	47.3624
COL4A5	3.16097	4.81655	4.67992	4.29959
AGRN	35.1995	37.856	42.2908	36.4021
NHS	1.20778	0.76004	0.983355	1.08359
FAM166A	6.22497	4.75753	4.83165	5.56721
TMPPE	0.822742	0.937847	1.01441	1.08452
SMTNL2	2.00931	1.7811	1.53779	1.23922
LINC00265	2.45656	2.5035	2.86631	2.51867
LAMTOR4	151.022	139.561	145.794	141.405
PRKAR1B	12.4983	11.8284	12.8481	12.0146
HNRNPU-AS1	11.6316	12.5217	11.0907	12.1777
DCUN1D3	4.70507	6.26949	6.49812	5.9931
AC002398.9	4.33879	4.31128	3.95889	4.535
TUBB4B	619.844	487.36	498.366	493.395
AGAP4	2.26142	2.16116	2.16943	2.22129
CTD-2228K2.5	22.1067	21.8102	21.762	21.3461
COMMD6	124.924	127.654	111.238	119.953
HYKK	2.4757	2.02316	1.66861	2.06884
ZNF383	2.76616	2.47984	2.10204	2.24687
HES4	76.7185	72.5531	76.6651	70.1049
ZNF669	4.30661	3.83315	3.24401	3.58822
PLSCR1	20.1626	18.7534	18.2023	19.0254
C3orf62	3.5398	3.37384	3.79379	3.53255

ZNF559	6.0377	4.858	4.43954	5.45636
SBK1	6.3611	8.08359	8.03381	7.20644
SLC38A3	1.17547	1.41224	1.11176	1.00632
GTF2F2	60.4385	51.6989	51.1888	55.4129
FAM92A1	105.423	91.8244	78.3354	87.3123
FOCAD	18.5457	19.1282	20.7453	18.5981
PRR19	5.6976	6.01399	5.48228	5.10196
ZP3	8.41753	8.74882	8.82712	7.94202
SELL	1.16573	1.15351	0.753107	0.729067
CHM	6.9261	6.0925	5.68629	6.35627
BLOC1S5	5.77738	5.1848	4.16132	4.88975
IER5L	49.28	45.3911	42.7417	38.3746
H2AFX	245.934	230.045	228.97	220.293
SERPINA5	0.612259	1.05671	0.919034	0.717762
C19orf54	5.36958	6.06579	5.90157	5.44222
LCTL	3.32906	2.55158	2.59912	2.62599
C22orf34	3.46766	3.74728	3.84463	3.71548
FAM83G	20.9461	20.8334	21.4791	21.5092
SRSF10	58.7484	46.1574	45.8893	48.7666
HBA2	2.13172	3.11178	4.48631	3.66235
DUSP28	4.3903	4.39086	5.49323	4.67847
C15orf52	2.95182	4.61624	4.48215	4.07114
NBR1	14.2551	16.7927	16.0982	16.176
RALGAPA2	2.62699	3.29594	3.03668	3.16467
NDOR1	10.7674	10.7098	10.9844	9.64547
NPIPP1	7.74416	8.6235	8.66137	7.35462
CLN3	33.2778	27.3435	28.3314	29.2134
FAM72B	5.09767	3.11137	3.24481	3.78893
ASAH2	6.38612	4.37141	3.89096	4.94596

SUMO2	484.449	413.803	381.815	422.75
NANOS1	2.31671	2.11379	2.30854	2.35508
ZNF177	1.32051	0.929223	0.91303	1.0429
LDOC1L	10.1899	11.5176	11.7952	11.8862
S100A16	177.915	190.606	197.023	194.391
PTAR1	6.31045	4.78613	4.90763	5.88347
FAM154B	2.491	1.71256	1.43711	1.48691
PARVB	7.56207	10.0225	9.44318	9.5153
TEKT4P2	2.98571	3.27287	3.46426	3.24637
SLC4A5	3.00855	2.1345	1.60013	2.00621
UROS	30.3503	41.8544	38.4776	36.8082
ZDHHC9	12.0314	10.4227	11.2122	11.5299
ZBED6CL	2.68072	2.73295	2.90781	2.74352
SMIM15	71.8144	50.4575	52.0407	55.7663
FAM221A	1.51893	1.79266	1.4896	1.19754
TMEM120B	14.6767	13.7873	13.7124	14.2416
RBM34	47.9035	43.8994	43.9366	51.4071
NOXA1	1.04358	2.33275	2.25781	1.64018
TMEM198	3.99992	7.07349	6.56076	4.32511
BCL2L15	1.7216	0.930087	1.22627	1.12132
ZNF548	2.2026	2.28478	2.57051	1.91367
MTF1	5.34663	5.17304	5.23118	5.70515
ZNF322P1	1.20188	1.35661	1.2134	1.30819
TMEM201	23.3381	19.5626	21.162	19.8441
NHLRC3	2.91762	2.42583	2.2203	2.30761
ZDHHC11	3.40985	4.52825	3.63285	3.4778
SLX4	2.35393	2.15129	2.27577	2.11506
RPL14	3563.62	3222.09	3380.89	3438.36
RPSAP47	1.52675	0.99766	0.50006	0.908439

FAM78B	0.742959	0.786434	1.0909	0.788968
FBF1	4.64083	5.91331	5.61883	5.0369
MSL1	36.7481	34.3556	34.0853	35.9348
GJB3	24.4781	19.4845	23.1886	23.0915
TRMT2B	4.24812	4.19442	4.90401	4.70483
PTPLAD2	2.84872	2.89253	2.5633	2.58735
C1orf192	1.37746	1.16631	0.882901	1.20068
FAM120AOS	23.4859	24.6533	23.246	25.0085
RP11-427H3.2	4.31995	3.61279	3.84288	4.02515
NOC2L	192.031	169.23	178.697	171.789
NELFB	69.9347	63.719	65.7692	62.4241
ZNF292	3.12048	2.70463	2.7514	3.00253
KCTD21	1.92876	2.00068	2.3701	2.819
ADAT2	7.01694	6.67992	6.62141	7.25388
AC138783.12	2.38602	2.62164	2.60999	2.41679
ZNF567	4.31883	3.65802	3.5241	3.51041
NDUFA4	222.461	211.048	192.609	217.212
ALKBH2	31.2435	28.3071	27.602	30.2763
RNFT1	9.88046	10.0444	11.3275	10.2305
FAM111B	6.26187	4.8173	5.56463	6.40391
H1F0	110.149	137.529	128.203	139.355
LITAF	37.0288	35.3744	35.2957	36.2517
TMEM120A	15.1392	14.2264	13.6348	12.4631
ARID2	7.86623	7.79309	7.03414	7.44696
SF3B3	232.42	209.207	211.5	218.115
BLOC1S3	5.7891	5.77477	7.13154	6.76105
SP6	7.45156	7.28793	8.35128	8.54676
UBE2Q2P1	1.06343	1.84007	1.33444	1.34366
CLDN4	38.1256	37.1793	38.9832	34.7119

ZNF573	2.08968	0.88987	1.23997	1.22308
HN1	812.747	736.089	700.66	734.53
ZNF527	0.774817	0.942605	0.939059	1.06261
S100A13	131.748	127.896	132.356	134.659
ZNF33A	5.94371	6.52308	5.49053	6.57774
ZNF600	5.93486	5.79958	4.73315	6.7582
MAOA	3.9927	3.88099	4.13045	4.76058
AC016683.6	34.5233	36.5846	40.0561	37.8933
C15orf61	13.3088	12.8818	12.8299	12.9562
TSPYL1	33.0529	32.8106	27.6773	32.475
PNRC2	122.447	56.5845	64.7789	70.6605
GJB5	6.404	6.54717	6.46799	6.64457
FHIT	2.05113	2.35296	1.96906	3.11519
ZKSCAN3	3.42794	3.28574	3.64805	3.73556
RRP7A	52.9637	45.9781	48.9655	52.6151
LIN54	8.98413	7.66043	7.01021	8.05649
FAM53B	9.43146	8.44675	9.40936	9.18273
S100A14	68.0784	61.7762	66.0722	67.4277
KAZN	7.49624	7.00684	7.72807	7.3594
SLC35E2B	7.07567	11.1841	11.3053	9.69807
RPS2P46	571.696	592.151	520.688	585.521
TMEM194B	4.66641	4.33979	4.73212	4.69058
ALG1L	0.660062	1.24205	1.07398	1.31968
C8orf76	39.1945	35.4503	31.3938	36.4216
HMGB1	618.034	538.802	493.525	539.351
MMP23B	3.39681	1.86978	2.52065	1.86551
SH2D5	10.8781	8.72148	9.70379	10.3767
BLOC1S2	60.2852	59.5631	59.0193	60.246
ZNF724P	2.03934	1.65549	1.47633	1.39212

IL1RAP	1.36466	0.97355	0.778652	1.02736
ZNF699	1.47668	1.05828	1.56915	1.57169
TDRD7	3.47846	4.26704	4.35452	4.26349
C16orf93	5.39272	5.75234	5.8183	6.02731
KIAA0895L	9.89652	10.7022	12.5111	11.4041
MYT1	1.26153	0.807284	1.01287	1.15697
SPATS2L	51.2539	45.354	45.1332	53.0902
ZNF250	3.4275	3.42976	3.44921	3.3834
WDSUB1	4.56421	4.28955	3.97072	4.45651
ZNF79	2.8886	3.88464	3.596	3.08399
S100A4	289.713	314.338	306.398	308.487
PLEKHG4	19.656	23.1739	24.2256	21.6227
ACADSB	10.1956	12.3113	10.4105	11.0417
STK40	13.9644	17.5818	15.6202	15.045
TMEM63A	3.8518	5.87514	5.52392	4.51654
MPHOSPH8	9.75543	10.4036	9.96392	11.5401
RNF216P1	9.95147	10.1727	10.5164	10.7793
EEF1A1P5	479.313	2274.28	2111.55	2247.46
GREB1	1.343	1.41973	1.37659	1.33418
ZNF766	6.93987	6.0153	5.91643	5.99412
RYR1	2.093	2.00724	1.62733	1.82796
SRGAP3	1.59666	2.41429	1.72742	1.56897
FAM217B	5.31604	2.5648	3.07627	3.65131
TUBB	281.831	232.894	204.698	177.656
LCOR	8.18551	10.3457	8.15528	9.39177
SUPT5H	57.9369	51.9846	56.6894	54.0017
XPNPEP3	124.75	114.566	105.947	113.045
ZNF107	2.22805	2.42413	2.252	2.62842
PPIA	3222.19	2822.85	1756.8	2099.26

GTF2IRD2	0.462378	1.12728	3.05432	0.851298
SUPT3H	4.60708	5.59548	5.16688	6.06709
NIF3L1	40.5763	34.1658	35.1024	38.4117
AC005154.6	3.57928	3.10889	3.36502	3.32408
ATP2A1	2.55085	3.15907	2.21306	2.29983
RP11-497H16.5	1.03917	1.15179	1.16447	1.06149
IARS	149.321	160.226	134.055	138.399
HIATL2	9.34338	13.8888	10.806	12.6528
POM121	13.0797	11.5507	11.2521	11.0559
ZBTB44	10.1187	9.38909	8.16894	8.9788
CGB7	1.4663	1.49098	1.74525	1.3385
CD55	26.7737	24.9731	22.7643	23.964
ZNF565	1.75308	1.5136	1.46059	1.66805
WDR5	55.285	47.8609	49.5327	50.5823
LONP1	113.023	111.581	106.535	97.6502
TRRAP	14.2076	12.8485	11.5898	11.784
SRGAP2B	3.84725	4.41054	4.05034	3.39379
FUT4	2.79552	2.93858	3.25295	3.47539
ASB13	10.859	12.7504	13.656	13.0626
ZNF34	2.4974	2.25829	2.62594	2.94315
ZNF774	1.16613	1.31652	1.9703	1.35423
PTPN1	39.3501	36.8638	39.7509	41.4283
EVL	8.15857	5.55066	5.49023	7.00431
NOXO1	5.76262	5.54825	5.72532	5.80546
EPHB4	35.7358	36.3171	39.8064	37.5687
ZNF765	3.64611	2.81082	3.31271	3.02058
ZNF124	5.86259	5.92947	5.547	6.44949
XRCC6	457.053	441.561	434.492	447.533
S100A5	3.21312	3.10795	2.60732	2.4959

PPP1R26	12.3145	9.91503	11.7251	12.2236
TSC22D2	8.80596	8.93419	9.08795	9.17028
YRDC	59.8223	40.9625	50.5538	50.8512
ZNF777	10.9572	10.7262	10.8835	10.0182
PIK3R4	30.2118	25.2863	26.7531	30.3973
ZNF775	6.02159	6.95388	8.56814	7.19097
TRAPPC2	6.9426	7.1256	6.88324	7.59319
MYL6B	74.6278	68.4212	62.328	61.5115
ZNF799	1.6503	1.39382	1.34681	1.64163
SIAH1	13.8697	13.4574	12.6023	14.3384
C20orf96	7.44561	8.06224	8.40039	8.11157
IPO4	64.6434	58.4151	61.3532	59.549
NCOR2	33.6297	41.3441	46.9572	39.2154
SULT1A1	10.4534	14.7739	13.9977	13.7733
PRPF40A	231.372	198.772	213.394	227.141
GDAP2	5.83545	6.7479	5.82872	6.90064
ANAPC7	52.0303	43.2309	46.3627	42.9481
TPK1	4.22034	4.90531	4.6847	4.57725
SLC6A9	2.56773	4.93223	3.58445	3.53459
AFAP1	4.19871	4.55423	4.59259	4.48156
NACA	1142.41	1000.11	1014.22	1045.58
MYO18A	27.0637	21.563	32.8002	28.3288
C17orf59	6.69864	6.03065	6.55964	5.88357
MAN2A2	26.73	26.8996	29.5107	25.6358
FAM72A	8.89347	6.83298	6.27419	7.02842
CACNA1H	7.21608	9.67783	11.2342	9.20345
SULF2	6.80159	13.7604	9.65651	9.06911
HBG2	4.10712	4.97591	3.68504	4.87426
LAMA2	3.13995	3.3161	3.20166	3.39082

PLXNB2	75.352	92.3664	91.651	79.3057
XRCC2	5.34155	4.49363	4.38456	4.61781
MYO6	10.8016	10.5722	11.6408	11.9799
MKL1	10.5975	9.81234	10.3337	10.8677
HDAC2	192.584	136.461	152.947	166.403
ZNF782	1.64178	1.24582	3.08544	2.20599
ACN9	34.8406	28.3956	25.6576	34.3075
HRH1	2.95644	2.70177	2.77892	2.92222
RABL6	50.1113	51.5548	55.9131	52.6654
GPR89C	2.2378	1.87897	1.98737	2.02114
ZNF136	1.45231	1.44106	1.20546	0.9253
ZKSCAN5	4.73964	4.60279	4.7202	4.89107
TRAPPC4	55.2644	47.7694	46.3376	47.3813
AC004057.1	153.637	130.873	109.719	123.851
TECPR2	5.19813	6.03413	7.4312	5.68096
LINC00173	4.04151	7.31159	6.86938	5.61581
ZFP62	8.28061	10.2643	9.32219	10.1381
ERI2	16.3033	21.2027	18.5121	18.6461
TOMM7	256.299	228.03	223.425	247.232
HSH2D	3.86863	4.86941	5.75123	5.99605
TRPV1	1.37062	0.975872	1.3077	0.723423
ZNF33B	1.76899	2.68037	2.25731	2.43818
PDXDC2P	2.69219	3.1882	3.5783	3.43716
ZNF512B	5.29761	5.19926	6.50489	5.9338
AMZ2	65.4689	64.227	60.7276	61.2201
ZNF431	5.50463	5.17752	4.99876	6.06396
NF1	4.72239	3.95505	3.80421	3.34337
VKORC1L1	17.6812	15.0567	15.1677	16.2723
DAPK1	1.14687	1.38293	1.72114	1.74133

COL27A1	2.85934	2.76903	2.94375	2.56771
GM2A	9.97448	11.75	10.8663	10.3874
S100A2	34.4956	33.9042	28.0092	31.326
SNHG17	54.1773	48.4278	43.5934	47.7746
ZNF700	4.8751	4.2249	4.21936	4.08607
CD47	22.8919	20.6894	22.4021	21.2916
TLE1	10.4825	9.47521	10.2989	10.6406
STRN3	22.2387	20.5709	22.214	22.5369
ZNF239	3.93833	3.78294	3.2986	3.3582
CTBP1-AS2	6.98596	7.48011	7.72929	7.27434
ZSCAN16	2.53994	2.46127	2.3402	2.38979
MVB12B	4.35039	3.66984	4.66711	4.36327
C6orf106	25.3436	19.1227	22.894	23.1405
ADA	17.5602	20.221	21.5125	20.0608
ARID5A	6.49512	5.08939	5.95673	5.28426
PPTC7	10.3999	8.72172	8.92247	9.76435
NHLRC2	9.25775	5.98654	7.32232	6.39658
HIST1H2AD	1.12769	0.378472	0.997869	0.180188
CBWD3	24.1996	20.3118	20.2064	22.5397
LAMB3	21.7672	22.0043	25.7735	23.4448
KPNA5	3.28474	2.12992	2.40827	2.51163
ANKRD36B	1.06439	1.40237	1.40927	1.40697
ARHGEF12	16.4772	16.6392	16.801	18.6717
ZNF252P	7.97453	8.55967	7.89278	8.89143
PDLIM7	70.8475	72.0798	76.7676	67.4411
FLNA	177.355	176.59	187.954	169.442
RPS26P11	1.05567	1.01365	0.0800627	0.559367
SRGAP1	4.426	3.92082	3.87515	4.23252
FAM3C	150.358	162.46	161.669	188.021

NOP9	20.9332	19.3778	22.1073	19.9723
SLC39A10	10.9359	8.9222	8.36527	9.25576
CASP4	8.04454	8.32814	8.49595	9.02132
AP2A1	47.7093	54.9153	62.1712	54.8866
ZNF585A	2.12121	2.25306	1.92087	1.52907
FUT11	9.88987	9.10936	9.51719	10.2049
ANXA4	37.6817	46.2899	45.7562	41.3347
LAGE3	31.2504	28.6784	29.1561	29.2743
WDR5B	1.16776	1.55044	1.29003	1.45721
WDR45	9.24049	13.4908	11.8281	10.5803
METTL9	32.5942	30.8671	31.7345	33.2833
ZNF138	4.80284	4.42108	3.64183	4.8369
SERTAD1	19.5578	18.3574	18.0834	17.4678
ZNF100	4.41599	3.31577	2.50802	3.42051
CXorf40B	10.695	8.54604	10.0841	9.60865
ZNF398	4.89929	4.50909	4.51658	4.93577
ZSCAN25	7.17524	6.37078	6.27467	5.75264
ANXA6	43.3582	46.405	46.3709	44.1696
GMFB	34.2637	37.2401	31.2476	37.2111
ZMYM1	9.47292	8.53183	8.24828	10.1619
RP5-874C20.3	4.10203	4.35603	3.65238	3.41116
MAFG	26.951	23.9958	25.669	25.1267
ARRDC1	29.7294	37.4024	38.1119	33.1779
KIAA1671	7.55165	7.02621	7.35031	7.93516
IGF2R	12.8548	15.3226	18.3973	13.1752
DYNC1H1	102.183	121.864	110.608	101.992
SLC6A17	4.71555	5.84259	5.63615	4.96271
PCBP2	492.273	458.944	493.378	477.768
ZGPAT	23.517	20.4509	22.8634	20.7549

SLC25A29	20.9953	20.4891	23.3907	19.0469
PGAP1	1.33304	1.44161	1.33773	1.342
SRC	24.0553	28.0857	26.8969	25.179
PCNXL3	30.3487	29.1279	35.208	31.5378
ACSL5	37.9706	52.9617	53.2725	48.1512
LRRC8B	7.18577	6.29576	5.45141	6.77264
ABCB8	29.5433	30.4302	32.1795	29.2998
SND1	52.7643	56.7815	55.2896	60.5523
PSMD12	112.514	106.245	101.737	113.31
MAGEA6	7.55379	6.88987	7.09954	5.68607
FLJ27365	8.58077	6.8838	7.11924	6.20893
C20orf112	2.2808	2.35224	2.34262	2.32893
C9orf169	3.05955	3.99728	3.63803	2.89063
ENTPD4	16.1979	17.0982	17.1096	19.0764
C1D	24.161	18.9718	19.4176	24.5916
TBC1D9B	71.266	69.343	64.4827	59.6134
HIST1H4J	3.04926	2.88953	2.42752	2.40335
SERPINA1	0.689587	0.828918	0.956171	1.11554
KANK2	18.9058	18.0004	19.7565	18.8278
C6orf141	5.56478	5.00669	6.48603	5.97616
GTF2E2	41.3591	35.6003	37.8106	40.2247
RAD54B	13.4308	9.23723	9.19163	11.1265
ZNF165	3.34032	2.79519	2.92972	3.307
SYNGAP1	3.62449	2.81161	2.14731	2.86447
FITM2	2.42294	1.96512	2.77938	2.84739
BLM	13.9638	11.3744	10.7149	12.9363
RP11-366L20.2	1.03236	1.33083	1.31717	1.18354
ZNF720	7.324	7.26907	8.02351	7.79765
DDI2	7.25087	6.82629	6.01899	6.75364

SVIL	6.19916	6.41757	6.08327	8.64535
TRIM33	14.1299	12.2285	12.1989	13.502
LRP10	19.8931	23.1332	24.2598	20.6455
PELI1	2.52598	1.84751	1.79971	2.03729
ZNF655	27.4756	27.6192	33.8369	32.7905
MRPL21	115.804	99.8141	106.071	116.591
UAP1L1	8.96668	8.05461	8.4267	8.01941
BNIP3P1	1.69557	1.9048	2.0426	2.16416
ZNF786	4.41224	4.35693	4.91474	4.69033
ZNF517	3.06519	3.71392	3.78703	3.72855
ZNF675	2.16342	1.98698	2.06237	2.22353
SLC22A5	9.80009	10.9067	12.7818	12.4845
DACT3	2.00095	1.28463	1.58109	1.52007
ADARB1	4.23325	3.92133	4.80419	4.43425
ZNF860	1.27744	1.27309	1.30926	1.42424
HTT	17.9253	16.7778	19.2086	15.7663
C5AR1	0.979268	1.11252	1.00879	0.830266
VEPH1	2.03869	1.33113	1.81017	2.36797
SHPK	17.4911	14.7588	15.9115	15.4687
IPP	6.95014	6.58849	7.27405	7.92411
MAP3K5	3.63039	3.45256	4.28201	4.88569
OGDHL	4.00284	5.9481	6.38596	5.31726
GSTK1	45.2574	52.0973	49.5094	51.2304
HNRNPAB	269.452	197.449	212.177	229.428
STMN3	122.875	139.912	160.25	138.516
PDGFA	11.8983	11.7904	9.5397	9.05754
CSAG3	6.21439	5.51304	6.10629	6.32622
COL13A1	2.64605	2.33489	3.51658	2.95342
ZNF695	9.40668	7.14909	6.9759	8.39142

ZNF628	4.49417	4.25138	4.67455	4.58292
RPF2	57.6954	44.5037	43.7686	48.6097
MIB2	121.891	119.757	135.166	113.736
MYO5A	5.86293	5.96667	5.89887	6.10608
ATG7	15.6603	18.2649	18.9377	18.6939
SIPA1L1	10.8312	10.8288	10.9654	11.9186
RAB40C	7.79986	7.68405	7.97909	7.74765
PIGN	3.65384	5.89182	4.51276	5.09707
ZNF624	0.751904	0.724921	1.02258	0.704403
HHLA3	7.59783	5.6667	6.42212	6.5836
HOXA4	1.30391	0.880583	0.954242	1.20932
TOPORS	6.86744	6.45958	6.17193	6.4315
BCO2	1.04759	0.902796	1.23044	1.13989
ENTPD6	44.8158	50.0536	50.7348	48.8633
DMBX1	1.99988	1.56623	1.83898	1.7074
ENPP1	1.84467	1.94664	1.77124	1.9172
FAR1	35.1715	30.3881	33.9209	34.2884
C5orf42	1.65851	1.18227	1.46528	1.55035
CXorf40A	1.64199	1.58297	1.69079	1.94347
CDC42SE1	24.9645	26.0333	25.7331	26.8458
DPP4	0.891692	1.37053	0.858407	1.02731
TBC1D3	1.83171	2.45576	2.40167	1.99011
SPTAN1	17.1668	18.4697	16.4276	16.8178
NMB	12.5222	12.3435	12.0153	11.5216
ZNF595	0.969321	0.730456	0.652004	1.13673
PARVA	14.9691	16.4887	16.2275	17.9587
FAM114A1	8.4683	10.9942	10.8401	10.6742
RPE	46.0142	39.1612	37.1469	41.4458
PHF2	6.98598	6.95801	7.32914	7.76023

RPS26	959.37	829.508	831.39	887.743
PTMAP2	3.31093	2.6603	2.32577	1.37132
PSAP	180.645	217.156	221.137	214.809
S100A10	352.613	349.012	306.11	359.684
RPL37A	2330.55	2346.06	2247.65	2524.25
HOXC6	1.44514	2.01754	2.01359	2.19737
TXNRD3	5.55073	7.91809	6.53187	6.0968
CFD	85.7925	92.7093	94.2792	78.8044
MCMBP	138.402	105.946	108.259	124.16
EME2	6.77395	8.16167	8.46081	6.92268
DHRS4-AS1	7.30961	7.89232	8.03879	9.01043
TAF13	34.316	31.0106	30.9152	35.5513
ZNF780A	9.26307	7.09889	7.88588	9.52393
ATAD3A	100.442	85.2988	90.6898	85.6949
FAM118B	13.7833	11.3386	11.1433	12.9784
CTC-301O7.4	1.61459	2.55093	2.1249	2.23296
CCDC180	1.9956	2.21954	2.8675	1.91784
SLC9A8	3.4479	3.29115	3.30967	3.25706
OCLN	6.90918	6.44665	6.18991	6.17934
AC061975.1	0	1.92238	0	0
ZNF181	2.77589	3.10646	3.00867	3.14674
FAM212B	1.70135	3.3764	2.5085	2.43659
ZNF44	1.33501	1.60009	1.31166	1.46682
GPAA1	43.8483	47.3495	47.3151	44.3254
SGTB	4.73841	2.58936	2.52793	3.27396
MYO1C	80.429	87.1625	99.0938	88.3051
NKIRAS1	12.3645	10.6255	9.81172	11.5507
MEIG1	0.493879	0.522661	1.09475	0.709125
KIF13B	1.85364	2.40871	2.25401	2.24587

ADH5	153.321	141.428	145.63	152.26
HIST1H2BK	26.1584	22.8497	16.7718	16.1697
TEAD4	42.2243	34.6228	36.3738	35.0085
SPG7	40.4342	42.5902	44.6699	37.9943
HIST1H4K	2.27238	1.79894	1.60482	1.7755
C2orf27A	1.5028	0.75537	1.23849	1.27369
ERO1L	33.2999	31.4044	28.9002	32.3817
F8A1	6.90765	6.88972	7.74304	7.57768
ZNF823	2.54973	2.39119	2.17295	2.32237
PLCG2	9.21533	10.0417	13.1814	11.5859
FCHSD1	8.95598	11.8194	11.7957	9.78492
S100A6	2019.73	2110.04	2174.44	2028.34
RPL12	2105.29	2185.02	2100.92	2086.96
DNM3	1.82457	1.38274	1.58377	1.21818
ZNF121	16.9206	14.697	15.0379	16.3011
MPZL1	55.8524	41.1719	41.2239	45.015
VPS13A	7.51576	8.25823	7.68264	8.20467
MBP	8.8214	8.24379	9.13291	10.034
AKAP17A	14.2063	13.2047	13.7377	13.3074
LEKR1	1.1977	1.28852	0.912427	1.00842
C1orf122	80.0163	81.7803	103.907	95.4958
SNHG12	123.772	86.0315	74.7019	92.2579
NOL8	15.3959	16.3638	16.8819	16.0661
IRAK4	4.0801	3.37766	3.81113	3.95606
MRPL42	111.413	96.4814	95.7752	112.74
ENTPD7	5.85963	4.79035	4.46571	5.08094
ZNF335	9.2326	8.29568	8.86792	8.03969
RPS4X	1004.35	976.721	1033.21	1072.24
ZNF273	4.54336	4.05908	3.96818	4.51864

ZNF84	3.45169	3.53598	2.56422	2.96208
MAK16	32.7689	26.4063	27.0185	31.294
SIRPA	10.5867	10.6308	10.9922	10.9991
GRK6	68.9753	58.4477	64.1453	57.3686
PRIM1	37.3188	35.3777	30.5434	36.5348
5-Mar	36.7283	33.0073	34.7738	37.2293
RP11-347C12.1	10.0458	10.0232	10.4416	8.411
AKR1B10	0.903104	1.18633	0.678607	1.09895
ZBTB14	4.95593	3.78508	5.11286	4.90764
CD2AP	14.2897	12.618	12.0242	14.2714
NUP62CL	6.58882	7.85646	7.48367	8.06036
SFI1	15.5372	15.8252	13.4219	14.3727
ZNF649	1.82761	2.08062	1.84171	1.96509
ZNF248	2.56693	2.72972	2.37461	2.44078
SNX29P2	1.02863	1.22808	1.0722	0.996615
CHSY3	1.04786	0.811191	0.773489	0.757134
TOR4A	24.4567	21.1713	24.6627	22.2498
LPAR1	4.56119	4.01283	4.99493	4.87362
HIBCH	19.4342	19.7555	22.7666	23.3561
ZNF544	56.24	45.2661	40.2987	52.0858
SOWAHC	6.82033	6.6804	6.99405	6.95239
ZNF770	12.0713	13.7479	12.1437	13.4887
HMGN5	4.40023	3.35789	3.74994	3.84654
MIER1	8.84234	8.16351	8.01789	8.78741
MAN1A2	10.0559	11.1177	11.4876	10.6937
SVIP	13.2427	11.1548	11.7321	14.4389
ZNF251	6.87695	8.03329	8.17633	7.83324
DDRGK1	29.3678	25.261	25.8422	24.3629
TFDP1	220.923	179.281	186.677	192.544

ZNF607	2.00268	2.20783	2.18159	2.1268
HSD17B11	21.6733	21.8731	20.2642	21.6976
SZT2	3.08676	3.51546	3.71181	3.82811
SULT1C2	0.543024	1.90015	0.953135	1.537
ZXDA	1.00668	1.09439	0.874069	0.894821
RPS6KL1	5.55784	6.91806	6.73876	6.43749
QRICH1	99.1975	104.55	104.815	98.4172
CSF2RA	0.724449	1.42932	0.911366	0.506503
FKBP1C	8.26761	9.62023	8.1974	8.21217
DDX42	63.8327	62.7551	60.522	62.462
RP11-98J23.2	1.37904	1.7605	1.94967	1.82265
RPL23A	1139.1	1112.16	1127.36	1239.02
SLC29A3	3.51428	4.38891	4.96516	4.55588
STYX	10.222	8.2878	9.24987	9.49226
UBL5	351.055	371.903	361.351	377.026
HELZ	5.99264	6.03019	5.97024	6.55657
TMEM116	2.79005	3.88361	3.29955	2.99791
UCKL1	32.3679	31.1527	33.5503	30.3722
ZNF485	2.38868	2.36151	2.51919	2.6161
SDAD1	29.1072	26.8201	25.0419	27.6936
SPDYE8P	1.50407	1.68759	1.55066	1.63468
ZKSCAN8	3.92567	4.18569	3.72532	3.9899
FAM109A	6.24084	5.4893	5.96449	5.71567
HYLS1	12.2714	10.3284	7.25199	8.61244
ZNF813	2.45044	1.46478	1.55808	1.32966
PIM3	102.836	79.0681	88.2953	89.5836
ASNA1	93.1655	93.2073	86.5508	87.8665
ASPH	165.916	148.948	152.61	155.065
SPRED2	17.674	16.4706	15.7811	15.5753

WWP2	15.8381	15.772	15.4769	15.5759
GFPT1	13.0073	15.5359	14.9216	14.9655
UVRAG	3.34851	3.19113	2.84756	3.05085
ZNF26	1.35796	1.16393	1.19587	1.10639
ITSN2	7.75153	7.94882	7.673	8.38269
MGEA5	44.1603	45.1435	41.5378	43.3778
MT1F	5.77601	6.68121	7.26177	6.5662
FAM115A	12.0527	13.5986	12.3092	11.9298
TXNRD1	335.223	238.628	253.222	267.881
NRARP	7.69149	6.9248	6.89689	7.1822
F8A2	2.83762	2.80317	3.01304	2.98069
ZXDB	2.6923	2.92793	3.02557	2.87148
ZNF480	11.8193	11.4108	13.2074	15.0925
ZNF587	6.64324	6.68988	6.76206	6.31562
TPM2	286.367	243.671	241.461	240.51
FLVCR1-AS1	5.72351	6.0895	6.04987	5.5092
ZNF280B	2.06782	1.70459	1.79354	1.99803
SH3BGRL2	1.95281	1.97011	2.10537	2.16527
ZNF808	1.25	1.27178	1.5139	1.34444
YTHDF2	69.0523	60.0938	70.3523	66.0543
NBR2	1.1177	2.03717	2.26568	2.15502
TMA16	37.3627	36.843	33.8613	39.8526
ATL1	2.06099	2.22693	1.90445	2.70812
MAFK	28.048	23.1726	26.0838	23.8995
GPN1	65.4974	56.0684	54.0815	56.165
ZNF28	8.77036	13.9713	7.33854	11.3382
ZNF511	44.7713	37.7783	38.7885	37.6589
ZNF627	5.14433	5.95042	5.41425	5.732
KCNRG	0.917442	1.31049	1.2297	1.27905

WDHD1	15.9381	11.6191	12.1879	15.5033
ZNF789	3.11118	3.52381	2.74383	2.4549
CTNND1	44.277	41.3712	49.2835	47.0689
DDX39B	127.458	115.017	89.9259	71.5854
ARC	6.13219	8.10507	8.30094	8.72481
NUDT16	5.86783	6.54389	6.54635	6.40272
TLK1	13.8984	12.139	11.105	12.8383
LRBA	21.9249	20.1343	19.9266	21.5931
MMP17	6.29748	8.6025	9.40951	9.16426
BAZ1A	18.2274	14.4738	15.3203	18.2405
COPS8	45.5456	41.0358	40.3907	45.9835
PPIAP22	76.2957	68.5994	44.6552	48.1146
CCDC69	3.00294	3.09055	2.57738	2.79174
MDM4	8.58802	7.24085	7.46195	6.14886
KLHL9	9.7164	9.05709	9.32547	10.038
NCOA6	7.36123	7.34166	7.26551	6.9103
STK39	48.3856	49.0134	47.6365	53.5557
C6orf89	18.6206	17.4984	18.5877	19.3975
CALM1	206.025	162.162	178.003	181.491
TTC37	7.71606	8.26178	7.80873	8.624
PAPSS2	7.68565	8.53833	8.5576	8.62376
AC012615.1	7.91869	6.82383	7.15911	9.2258
SLC9A6	3.60767	3.16175	3.44269	3.72555
FAN1	11.3624	11.564	13.0519	14.0487
EIF1AY	3.61803	3.76211	3.02494	3.48266
MT-ND6	1126.56	1230.77	1470.35	1448.41
IPO9	54.6639	52.2901	50.0127	56.3984
CEP290	3.30251	3.28994	2.83694	3.02419
MT-CO2	7042.79	7953.43	8405.8	8271.29

C1orf85	26.8606	27.8097	28.5978	28.5995
DLL1	1.12716	1.26358	1.4227	1.2979
ANKRD13B	17.2267	21.5702	20.493	18.6958
ECI2	40.4733	34.1441	39.1735	42.3745
UNC13B	7.81863	7.22788	7.9194	7.35809
MT-CYB	1062.5	1087.66	1226.15	1137.9
LDB1	39.4157	38.1129	38.9949	38.5272
PPP1R14C	8.17554	7.30531	7.49153	8.46499
CTR9	50.3052	48.3106	48.8867	50.5929
MSRB1	29.6546	28.4415	29.7117	29.5565
ZNF652	6.17928	7.47343	7.81438	8.0828
SMURF1	8.60437	8.29676	8.87285	9.30383
SLC5A3	2.87116	2.79806	2.88225	3.18366
RP5-857K21.11	53.187	62.5477	49.1221	39.7726
GPATCH3	13.7339	12.2535	12.3656	13.7865
GATSL2	1.35013	1.16501	1.19953	1.05132
CDC42BPB	29.3351	31.1732	30.1664	29.6658
PLXNB3	1.23646	1.42141	1.29658	0.632905
RPL10A	1220.41	1256.76	1198.39	1214.28
MT-ND2	2921.81	2847.78	3406.2	3382.7
FAM169A	5.83904	5.84285	5.98886	6.52863
ZNF830	9.22442	8.76126	8.32046	9.30831
MT-ND5	691.425	748.949	873.62	746.394
CNOT7	87.0908	84.0827	81.7949	86.976
TMEM184B	33.5083	32.9115	36.3761	32.8443
MTOR	19.154	21.769	20.6991	20.7264
SCAMP5	3.19772	2.41785	2.75468	2.68945
LRIG2	2.76224	3.03488	2.97357	2.87693
MT-CO1	3016	3671.65	3783	3233.41

PNP	83.4287	70.631	71.7121	70.9427
PAX9	1.63842	1.75389	2.02901	2.07701
GK	1.5067	1.50166	1.37154	1.74594
FOXJ3	19.0887	16.7481	16.8788	18.4098
ZNF358	8.51104	12.1659	11.4926	10.4074
SFT2D1	59.9548	57.9882	62.0724	66.778
CHAMP1	8.1166	8.4271	8.1365	8.99204
INPP5F	11.0446	8.67286	9.56302	10.392
ARHGAP11A	50.1314	34.0982	36.0201	40.3114
HMGN2	602.573	576.997	526.527	574.685
RP3-412A9.11	75.2244	75.4723	77.5708	70.1858
UBE2J1	15.3076	13.9981	12.3999	13.7648
OPA1	51.045	41.6699	46.8476	56.8771
DENND4B	22.6191	23.8496	27.5619	25.3139
ZNF277	7.57754	7.83899	7.1381	8.61437
MT-ND3	2937.52	3638.31	3795.84	4781.37
KTI12	11.8298	9.90781	11.0691	10.4661
SELT	112.454	99.7102	98.167	102.14
RUSC2	3.72311	3.10883	3.51929	3.2745
FICD	1.83496	1.29845	1.58786	1.39408
OSTC	101.288	85.2442	83.9493	94.9053
R3HDM4	57.7973	62.8385	67.3131	61.5361
TSEN15	25.7857	23.5903	23.3328	27.5918
LTN1	1.46207	1.84459	1.49061	1.83807
RUNDC1	8.72397	6.67142	8.1009	8.04142
GRK5	3.00765	2.93979	3.42466	3.56973
TYW1	29.5568	24.9908	25.8749	27.3809
DCAF12	22.9069	23.4305	24.0424	22.4334
ITPRIPL1	11.7717	8.88127	9.01185	9.15748

MT-ND4	3210.72	3566.97	3882.58	3568.27
SMC5	10.0559	7.04196	6.62849	8.24312
MT-ND1	1071.34	1153	1327.01	1206.36
PRMT6	7.21579	7.88911	8.7255	8.96685
SHISA4	11.6031	11.464	12.5719	12.6241
KIAA1737	10.4601	9.38145	8.86786	10.1029
CAPZA2	86.3329	71.0532	67.7613	82.8791
MT-ATP6	4215.29	4500.45	5052.64	4709.75
TOP1	75.2677	65.9747	73.6442	78.622
PRC1	107.55	87.3614	88.2128	96.7197
MAP3K3	7.1022	7.63834	8.03307	7.25447
L1CAM	1.72423	2.14246	2.38953	2.42187
SREBF2	33.0363	32.3445	35.5473	32.7939
C1orf174	28.3134	23.8366	25.155	26.6693
RASGEF1A	15.5672	14.8973	16.5273	15.3436
C9orf114	85.8579	80.0394	84.133	84.814
RPL39	1834.51	1842.27	1662.22	2244.61
DZIP3	4.62553	4.90115	4.87939	4.976
KIAA0753	5.81889	5.26305	4.9945	5.28018
DCLRE1A	10.4779	8.9442	8.90113	10.1145
ATG9A	39.7681	41.0445	43.4065	43.5481
NOS1AP	1.28971	1.41194	1.59704	1.33498
APRT	293.355	298.826	305.836	286.044
TBKBP1	6.26809	6.01547	6.35437	5.59494
CCDC167	32.2636	21.4871	21.7189	24.1581
MT-CO3	5785.92	6231.21	6460.27	5731.32
L3MBTL3	6.85535	6.89957	6.94993	8.12142
MFAP3L	5.36054	3.50625	4.1151	4.06759
NAGA	13.198	13.173	12.9091	12.9407

SMG5	46.3903	39.2496	42.6879	42.1995
KIAA1279	17.554	14.8325	14.9871	17.041
TGM2	4.17163	2.98001	3.08478	3.42327
ARMCX6	8.66174	8.30443	8.00822	9.21889
PJA2	11.5019	11.1789	12.4359	12.177
SGMS1	13.2555	12.2556	12.8161	12.4421
SNORA60	2.61309	0	1.08766	1.61122
RNA5S12	0	0.148145	0.716106	1.20688
RNU4-82P	0	1.65354	0	0
RNA5S11	0	0.148145	0.716106	1.20688
RNA5S3	0	0.148145	0.716106	1.20688
RNA5S1	0	0.148145	0.716106	1.20688
Y_RNA	0	4.03221	0	0
RNA5S5	0	0.148145	0.716106	1.20688
SNORD9	0	0	3.59984	0
SNORA31	14.6098	8.66607	10.8501	6.8345
Y_RNA	0	0	2.62185	0
RNU6-315P	0	0	3.0796	0
RNA5SP319	3.74511	0	0	0
RNU6-1330P	8.24019	0	3.54861	5.24559
Y_RNA	0	0	0	3.72298
RN7SKP173	1.05412	1.36345	1.31918	2.26479
RNU4-23P	2.72262	0	0	0
SNORA52	20.0291	9.32598	22.2524	22.9107
SNORA42	3.19649	0	0	0
RNA5SP383	4.68048	8.3922	9.84756	2.91305
RNU1-72P	0	0	0	2.38839
RNU5B-2P	0	7.74868	0	0
RNA5S10	0	0.148145	0.716106	1.20688

Y_RNA	0	7.40642	7.81301	0
SNORA73B	17.3737	20.2771	17.9276	19.5041
RNA5SP123	0	2.42524	0	0
RNU4-32P	0	0	1.02254	0
RNA5SP352	2.72441	0	0	3.3146
SNORA63	0.950504	2.05475	0	0
RNA5S8	0	0.148145	0.716106	1.20688
RNU6-485P	0	0	0	4.63037
RNU6-1285P	0	0	3.15579	0
SNORA71D	0	0	1.14049	0
SNORA72	0	0	0	2.33349
RNA5S17	0	0.148145	0.716106	1.20688
RNA5S4	0	0.148145	0.716106	1.20688
SNORA38B	3.36176	2.00363	0	0
SNORA63	4.05901	0	3.74199	7.23536
Y_RNA	8.09214	0	0	0
RNU6-1214P	0	0	3.31038	0
SNORA33	37.7797	17.7918	14.3369	26.1096
RNA5S6	0	0.148145	0.716106	1.20688
Y_RNA	0	0	0	3.62725
U3	2.25785	2.67636	3.78605	4.23614
RNU1-124P	0	1.15828	0	0
RNU6-105P	0	0	0	5.95722
SNORD8	0	4.18151	18.048	0
SNORA80	3.16429	0	0	1.94408
RNU4-1	2.82574	0	0	0
RNA5SP48	0	0	0	2.16922
SNORD46	0	11.4566	0	0
RNU6-1048P	0	0	2.73925	0

Y_RNA	7.12949	0	0	0
SNORA74A	0.671474	3.21913	0.851917	1.25815
SNORA3	19.5352	3.01346	12.3883	22.2104
RNU1-85P	0	0	2.13581	1.0568
SNORA58	2.39139	4.2862	0.997184	0
RNY1	0	3.3574	0	0
RNVU1-12	1.01818	0	0.32364	0
Y_RNA	0	3.76463	0	0
SNORA63	0	0	1.47101	0
SNORA65	22.3539	7.55716	13.4463	23.657
RNA5S14	0	0.148145	0.716106	1.20688
RN7SKP71	0.579098	1.21379	0.542843	0.574238
SNORA55	0	5.59945	5.25207	1.93479
RNA5SP78	4.17314	0	0	2.58416
SNORA71C	28.5285	23.6072	22.6722	19.4362
SNORA16B	2.09528	0	0.876112	0
RNA5S2	0	0.148145	0.716106	1.20688
SNORA14A	0	0	1.40701	0
RNA5SP160	0	0	1.39666	0
SNORA5C	17.9457	5.89069	3.31473	4.85047
RNU1-140P	1.36502	0	0	0
RNA5S15	0	0.148145	0.716106	1.20688
SNORA72	4.23235	0	0	2.64091
SNORA23	2.19249	6.48746	6.44838	8.86888
RNU6-1240P	0	0	0	5.00664
RNU6-234P	0	0	3.76609	0
RN7SK	7.50536	9.42224	10.5466	10.7442
RNU1-51P	1.104	0	0	0
Y_RNA	0	0	2.84456	0

RNA5S16	0	0.148145	0.716106	1.20688
RNA5SP37	2.79747	1.66046	1.15298	0
Y_RNA	0	3.70534	0	0
SNORA2	0	3.14045	0	1.6624
RNVU1-20	1.45414	1.05242	2.48548	1.22644
RNA5S7	0	0.148145	0.716106	1.20688
RNA5S13	0	0.148145	0.716106	1.20688
RNU6-395P	54.4277	69.2207	76.3427	44.0191
RP11-498P14.5	1.14433	0.747797	0.804441	0.742686
AP001007.1	2.71899	3.35517	3.98382	3.64395
ZNF525	5.92618	3.56264	3.35851	3.66343
RP5-930J4.4	26.7119	27.021	23.7049	29.0837
RP5-1113E3.3	14.713	13.6514	14.7987	14.0066
RP11-77K12.4	1.63741	1.76443	1.28771	1.492
INF2	47.5913	39.2754	41.2084	38.0379
FAM83H-AS1	7.73213	8.47667	9.49811	9.06148
AC004771.1	1.29481	1.11241	1.27251	1.36291
AC090519.4	1.76931	0	0	0
AL021707.1	4.4386	6.58951	5.70522	3.94558
AC069200.1	0	1.84516	0	0
GP1BB	3.08021	3.56693	3.63434	3.93042
MRPS18B	13.4852	12.1971	13.9001	14.9935
AC083799.1	5.8318	5.35661	5.64613	5.92703
AL353671.3	2.61256	3.36637	2.30414	2.84203
EFCAB2	4.266	4.85181	4.48616	4.09573
COX20	57.9248	52.799	53.0031	52.8449
CHML	3.61791	3.14311	2.9695	3.2543
TATDN3	11.2475	12.2845	13.5277	13.6143
C6orf99	6.25287	4.09311	5.14741	3.88433

C1orf53	4.28078	5.42264	5.22812	5.223
SAMD5	0.822731	1.42294	1.21211	1.2277
CENPW	58.9199	47.3678	44.0284	51.6548
MSTO2P	14.5886	12.5898	12.594	12.4396
SPRN	1.04555	1.0391	1.09728	1.05589
FAM229B	4.52254	4.7464	4.41985	4.45935
FANK1	1.89129	2.15544	2.04883	2.40809
METTL10	32.0099	29.2069	27.3291	31.8804
HIST2H2AA4	2.28957	2.33455	2.49645	1.99705
HIST1H3H	0.943544	1.21857	1.0034	1.17952
FAM72C	0.182591	0.035184	3.03276	0.0185933
HIST2H2BC	1.26464	1.34057	1.54994	1.11752
NBPF16	1.85663	1.91952	1.84771	1.69248
NBPF20	1.43983	1.70935	1.64423	1.52873
NBPF24	1.13799	1.25977	1.04073	1.15691
ATP1A1OS	5.53867	6.03395	6.47258	6.29805
SNHG5	134.507	129.997	116.625	138.428
GDI1	36.5784	25.7347	30.8677	31.6305
PCMTD2	6.59327	7.7279	7.69574	7.97054
SOX18	3.55267	3.53535	3.33844	3.29099
LIME1	15.8496	14.5481	17.9389	14.0543
SAMD13	4.32806	5.23127	4.73605	5.92547
FAM127B	77.9326	72.6653	64.0438	66.8908
EFCAB7	3.52588	4.10451	2.71048	2.99445
C9orf37	4.04277	4.20749	3.84864	4.17374
RP11-290F20.1	1.4995	2.36613	2.19931	2.1234
LINC00963	10.7316	15.7174	14.1218	12.521
RP11-247A12.2	21.9573	20.3157	20.4909	18.867
FOXO6	1.84146	1.49151	1.76466	1.75466

SYS1	16.7569	13.2192	17.0475	17.4885
RP4-545K15.3	5.67611	6.60528	6.36073	6.65807
INPP5B	5.57922	6.04448	5.10514	5.31861
TRAF3IP1	9.83339	8.64171	8.6533	8.23641
GIGYF2	17.6693	17.2917	19.8459	19.0468
C2orf72	2.02432	2.17475	2.40763	1.95126
RUFY2	13.8992	10.7599	12.1334	12.659
PHACTR4	7.85512	7.72698	7.88433	7.7402
ASAH2B	8.25528	7.49789	6.1347	7.62185
AGAP6	2.79484	2.37127	2.46183	2.29937
TIMM23B	44.2981	36.1817	38.4501	41.3102
ZDHHC18	26.3696	24.8285	27.351	24.6032
BMS1P5	1.333	1.22346	1.18736	1.20943
AGAP10	1.05983	1.19922	1.03323	0.994624
TMEM57	2.51618	2.50679	2.47859	2.6326
KIFC1	14.8429	11.8006	13.4539	14.2709
DAXX	14.4115	13.6039	12.9957	12.4154
BMPR2	1.86251	1.9652	2.00737	2.0904
TCEA3	2.80524	3.25764	2.78539	2.59268
PFDN6	42.798	40.5998	33.0126	34.2605
WDR46	15.2664	14.9793	17.0458	15.7754
RING1	11.6336	10.9288	9.82597	8.44017
HSD17B8	2.02535	3.03206	2.51609	2.00768
RXRB	5.85656	7.03027	5.85861	4.85432
OXLD1	40.4536	48.3861	51.6688	46.4171
HNRNPCP2	3.47695	2.7347	2.39411	2.0744
BRD2	48.7136	42.1366	35.0745	32.473
HLA-DMA	0.546081	1.23512	0.903184	0.622153
TAP2	16.8016	12.0495	10.959	11.624

SPIN3	2.29628	2.11275	2.46137	2.23544
RP11-622K12.1	45.067	43.838	42.268	44.4707
TNRC6C-AS1	1.14843	1.3799	1.50636	1.58586
PBX2	8.48852	9.44274	8.65717	7.55684
AGER	0.979541	1.10744	1.0353	0.823071
RNF5	5.82379	6.7359	4.93348	4.63045
AGPAT1	10.5191	11.1048	9.46281	7.71823
DFNB59	1.04816	1.13608	0.807202	0.787043
PRRT1	1.26688	1.05808	0.952484	0.741494
FKBPL	4.03346	3.72278	3.11851	3.16961
MRPL38	161.308	149.631	160.372	155.473
SP5	0.823619	3.94953	1.98635	1.43867
STK19	2.18654	2.64059	1.97255	2.15128
DOM3Z	3.27733	3.48867	2.53685	2.46766
SKIV2L	9.24721	9.12236	9.13485	7.7285
NELFE	21.6202	22.0835	17.168	15.53
ZBTB12	1.40795	1.84127	1.38816	1.32149
SDHD	154.773	114.893	154.177	153.665
EHMT2	12.603	11.794	9.83771	8.23771
C1orf134	1.85179	3.52711	3.69576	6.32751
NEU1	3.76192	2.57846	2.42695	2.38919
C6orf48	42.7062	43.4425	37.7145	34.5526
HSPA1B	62.2072	40.8413	38.0433	34.6004
HSPA1A	33.9513	22.3957	21.4056	18.923
LSM2	40.5421	36.9159	30.3112	33.5792
VARS	43.075	46.5359	37.2595	31.3675
MBD5	6.98107	6.10482	6.87236	5.42314
MSH5	7.71554	8.73814	7.02912	5.99439
ABHD16A	4.6185	5.80187	5.13936	4.01316

CSNK2B	59.2699	60.3487	49.2548	42.0126
GPANK1	3.22485	3.01132	2.31017	1.96005
C6orf47	1.74528	1.87677	1.6775	1.40769
BAG6	44.6964	47.0758	39.0895	36.1581
PRRC2A	16.8345	16.4837	13.9163	10.9283
NFKBIL1	2.91347	3.40365	2.32914	1.76398
ZNF814	1.57012	2.58544	2.02064	1.9717
MICB	4.50093	6.88146	4.4563	3.811
ZNF551	5.33723	3.74747	3.90707	4.33968
MICA	2.88165	2.52035	1.96766	1.58388
ZNF805	1.36527	1.42453	1.26082	1.24552
HLA-C	68.9567	72.7763	64.5513	59.4864
CCHCR1	5.16602	4.67205	3.55444	4.17957
PSORS1C1	0.577727	1.24069	0.638112	0.547561
DHX16	10.0342	9.01935	7.3596	6.62642
C6orf136	11.4943	12.5855	9.38934	8.78646
MRPS18B	33.398	26.6819	24.3603	22.7573
PPP1R10	5.78045	6.26673	5.18557	4.39582
ABCF1	15.0888	11.483	10.9264	10.8509
PRR3	4.35526	4.19672	3.32777	3.05617
DDR1	38.1925	37.9638	29.1848	26.8033
AC013268.5	11.452	9.90349	11.6994	11.7911
GNL1	24.482	22.3442	17.5877	15.1698
HLA-E	12.6716	12.8313	10.8312	9.01606
TRIM39	2.09816	2.23062	1.53474	1.36663
ZNF468	6.20889	5.96883	6.1914	6.38927
TRIM15	1.4681	1.26006	0.575757	0.825798
ZNF616	3.31023	2.51559	2.51613	2.84424
PPP1R11	5.25167	4.15048	3.75163	3.69494

GNB2L1	3179.61	3117.53	3105.28	2949.08
TBC1D8	8.9689	12.1863	12.2711	11.6765
HLA-F	1.77422	1.92151	1.33326	1.59971
CRHR1-IT1	26.2968	25.1669	25.2496	29.0777
RPS26P8	34.7454	30.7412	28.183	29.1786
AKT1S1	74.8171	71.1826	74.5555	73.7911
GABBR1	2.3681	2.89156	2.42895	2.03384
CASC10	2.0591	3.00964	2.68496	2.26533
AC012307.3	15.6599	14.8813	16.6867	16.1045
TRIM27	11.684	9.63918	7.31771	7.09126
AC083899.3	5.98506	5.23611	5.10475	5.25093
RANBP17	4.91889	3.66785	4.41008	4.94747
CTD-3232M19.2	3.43058	4.34469	4.58977	3.89992
KM-PA-2	68.945	58.0828	60.6889	56.2057
RP11-15J10.1	7.71997	8.15466	8.26815	8.81768
ZNF204P	1.84576	2.16856	2.03464	2.47592
CBWD6	34.8533	29.1782	29.3237	32.1078
RP11-111F5.4	2.57532	2.02105	2.15146	0.847467
MRPL53	92.1123	88.7795	93.7467	89.2444
MROH6	33.4344	32.8671	36.639	31.6132
ATXN2	21.7939	21.7618	22.8869	21.6719
DCTN1	61.5684	63.1884	65.8096	62.6899
TCTN1	10.301	12.3202	8.94222	11.8496
FAM216A	10.8057	9.64849	10.06	10.2437
ZBTB48	24.0944	22.2773	24.0571	19.4002
FAM201A	2.96069	3.21473	3.60005	3.49022
MZT1	19.5613	13.6246	15.1332	18.8323
C11orf83	32.4491	27.6468	27.2881	27.5166
ZNF783	2.77353	3.17924	3.29764	3.20865

C12orf73	24.2515	21.5812	22.8708	22.3264
ARHGEF34P	7.36186	6.34434	6.88053	6.76301
TRIM13	14.584	15.2168	13.7396	16.6494
SPIRE2	5.31491	8.81542	10.1365	7.67909
FLJ00104	2.07137	1.90414	2.04993	1.90191
SLC35B4	7.20658	6.23961	6.88709	7.31034
SYCE1L	13.376	17.4773	15.3134	14.6845
TMEM231	4.20604	4.45726	4.20573	4.06879
TRIQK	4.04627	5.68206	3.75249	4.61536
SDHAF1	17.0983	15.858	18.0515	17.4758
PSENEN	60.8094	68.253	58.4944	56.7728
ZBTB10	3.58096	3.7414	3.56437	3.90354
C4orf46	9.80625	8.99409	9.67226	10.2106
LGR4	1.29153	1.01527	1.24737	1.11583
PSMB10	109.203	88.9975	90.1825	90.58
RPSAP58	2.5893	2.31356	1.31604	1.07939
E2F4	106.393	93.9117	96.9972	95.0041
PDE7A	9.15205	8.71886	8.39041	9.53572
SNX2	51.1811	43.939	46.969	44.0971
NT5M	3.00044	3.78129	3.84624	3.67525
SARNP	77.531	70.6804	63.0305	70.8797
GPR56	10.6761	13.5711	11.4684	11.9094
IPO7	62.3446	53.3936	51.9142	56.4537
PRR13	252.017	241.073	253.286	262.82
TECPR1	5.24974	5.20222	7.72672	6.69543
MT1DP	3.74297	1.76525	4.31408	2.26125
C15orf59	1.70336	1.48473	1.67113	2.20233
SAMD9	0.851884	0.996121	1.0119	1.09608
CNEP1R1	9.06936	10.697	9.56346	10.5803

KRT81	0.772506	1.08371	0.742417	0.854385
ATP6AP1L	3.49976	4.85955	4.56923	4.39583
CCDC85C	26.0351	20.3823	21.4796	20.7462
AC004980.7	3.85065	3.41345	2.76271	3.06919
RGL3	15.1528	17.4077	18.7469	16.2223
NAP1L4	141.403	134.433	126.202	134.094
RP11-345J4.8	4.88842	4.07777	4.07469	4.57414
TMSB4X	202.146	240.764	208.763	235.674
TMEM256	99.3049	113.209	106.874	111.537
CHKB-AS1	3.82763	4.65201	4.60417	3.80842
CPT1B	8.25947	10.1328	10.5993	8.52067
SMN2	212.163	77.7867	96.5754	108.097
SERF1B	12.4166	12.3187	11.0386	12.1761
POM121B	5.51735	4.80965	4.8148	4.31562
HMGN1	265.906	185.574	186.629	223.178
STAG3L1	5.39108	5.90918	5.46733	4.98031
DENND6B	1.07211	2.55633	2.52914	1.76212
AREGB	62.6544	71.2695	63.8694	66.6826
EIF3CL	124.703	118.822	117.078	113.213
LCMT1	39.045	36.4199	38.1899	35.104
MFSD2B	2.12753	1.56555	1.91522	1.35032
CDPF1	13.1158	12.6686	12.4166	12.5735
LIN52	11.165	8.07825	7.96188	8.99508
RP11-706O15.7	1.08201	1.13187	1.12408	1.10767
RP11-706O15.5	5.05558	6.09107	5.704	5.56272
RP11-706O15.1	19.8199	20.3269	19.7619	19.5989
SMIM11	19.8862	17.2951	16.8217	19.0115
LINC00634	0.749841	1.05293	0.258725	0.966378
LYRM5	3.98105	4.58977	4.01044	3.79253

ITSN1	16.7663	12.3508	16.4592	16.9948
ITPRIPL2	16.0705	21.52	20.3813	15.1121
DENND1C	4.69417	5.73116	6.02356	6.44338
RP11-1212A22.1	35.8787	33.9746	37.6139	27.4918
CRYZL1	27.1107	26.8681	28.6872	27.3665
RP9P	9.74328	8.81541	8.51271	9.13166
C5orf51	9.04313	6.99817	6.88237	7.87971
AC005594.3	1.61183	1.62207	2.03619	1.89439
CYS1	0.474332	1.09193	0.933066	0.997012
PPAPDC2	1.81885	2.63464	2.69107	2.69316
KLRC2	2.42354	3.22848	2.62635	2.95719
RPS3AP47	1.81248	1.74661	0.885984	1.54951
C1RL-AS1	2.87132	3.30456	2.64685	2.14905
ZNF316	14.3679	13.0335	14.5727	13.2465
PDPK2	1.97404	1.85068	1.54176	1.88475
ONECUT3	3.0842	5.32542	5.58033	5.0064
CEMP1	10.2182	9.45772	8.51031	7.99138
RNPS1	188.653	172.48	171.428	173.377
DNAJC19	42.5839	39.3214	34.9126	39.9054
HN1L	75.1606	63.3234	52.2786	62.2791
SERPINB5	11.8517	17.5912	15.4034	15.6401
AC010507.5	6.87856	7.87327	7.63371	7.0629
TMEM191C	3.5921	2.77468	3.32717	3.03979
HERC2P9	8.8557	8.68076	8.48394	8.02488
HBA1	1.93837	3.17539	3.62818	2.98911
AC023490.1	5.85737	5.13195	5.57142	6.75641
AP000525.9	3.06681	3.36223	3.46573	3.33972
TSSK2	1.27331	0.72255	0.963152	0.837122
DAXX	6.49031	5.68144	6.57613	6.99837

TAPBP	5.95512	5.51905	7.01536	6.80569
TAP2	5.84454	5.10493	6.20259	5.89781
PPT2	2.12555	2.84415	4.70036	3.80765
TNXB	0.49307	1.79811	1.68702	2.45408
NELFE	9.49325	9.2205	9.64753	10.1228
DAXX	8.56933	7.59962	8.76942	9.22866
ZBTB22	0.895625	1.14843	1.24088	1.05928
TAPBP	5.02913	4.99423	6.15643	5.92691
RGL2	2.34539	2.58886	2.86489	2.81683
PFDN6	22.4427	21.932	23.103	27.2649
WDR46	11.2838	11.4233	12.0637	12.0216
VPS52	6.18254	5.55538	6.14648	6.06468
RING1	4.57873	4.63756	5.16991	5.36993
SLC39A7	13.7911	12.4049	14.891	15.1576
RXRB	2.63068	3.85892	3.95303	3.85542
TAP1	1.63101	2.10633	2.39861	2.2203
TAP2	5.83671	5.10011	6.19392	5.89345
PBX2	4.54345	4.72094	5.55721	5.62852
AGPAT1	6.61832	6.84869	7.83162	7.49857
PPT2	1.97475	2.47568	4.00708	3.23424
HLA-H	2.26505	1.98725	1.92793	1.68068
STK19	1.0493	1.3927	1.54931	1.32878
DOM3Z	1.31378	1.49703	1.75228	1.62886
SKIV2L	3.27665	3.58731	4.26555	4.13162
CCHCR1	3.47546	2.95744	3.42702	3.32451
NELFE	9.48955	9.21702	9.64916	10.1207
ZBTB12	0.969311	0.997605	1.17734	1.07321
EHMT2	5.55337	5.50912	6.16598	5.79973
FLOT1	6.6541	6.08468	6.38878	6.27039

C6orf48	21.2236	20.5373	22.2706	22.4847
CLIC1	52.4115	48.0831	49.5037	53.1856
DDAH2	2.8587	2.43304	3.24235	3.00445
ABHD16A	3.27709	3.39596	4.56743	4.20688
CSNK2B	29.0256	29.4642	32.9433	33.1048
GPANK1	2.902	2.36328	2.68734	2.77478
GNL1	8.3006	48.8057	58.3906	9.57669
H1FX-AS1	17.6768	17.171	17.4333	16.2849
RAB12	24.5653	11.2112	9.83383	12.9872
PRRC2A	13.0472	12.2404	14.9175	13.3254
HLA-C	28.3703	30.6212	35.1679	30.9249
NFKBIL1	1.69535	1.54301	1.77636	1.90203
MICB	3.92415	4.06146	3.92401	4.13352
HLA-B	47.2614	54.1142	55.6307	49.5671
HLA-C	28.6777	30.808	35.2945	31.181
TCF19	2.10363	1.65378	1.78074	1.91849
CCHCR1	3.70852	3.15873	3.35365	3.47587
PSORS1C1	2.53689	2.00342	2.44005	2.22194
GABBR1	0.657161	1.00031	1.09707	0.791833
VARS2	1.70844	2.17329	2.39229	2.35138
IER3	39.8246	38.6205	43.7979	47.9091
FLOT1	6.64336	6.08991	6.38067	6.26459
MDC1	2.28067	2.66823	3.30399	2.97341
NRM	1.33981	1.21169	1.33517	1.41562
PPP1R18	2.33526	1.81685	1.9749	1.96192
DHX16	4.20937	3.60026	4.08812	4.21277
C6orf136	2.39019	2.21112	2.51426	2.46932
ATAT1	1.10142	1.29496	1.40293	1.35361
PPP1R10	2.61282	2.59808	2.87065	2.97058

ABCF1	6.0562	5.3334	5.45968	5.99397
PRR3	2.35612	2.07138	2.04601	2.26776
GNL1	9.53273	8.72251	9.91136	10.4573
HLA-E	6.06875	5.95527	6.57118	6.20401
PPP1R11	1.5801	1.34977	1.59397	1.80439
ZNRD1	1.62095	1.40949	1.44432	1.60041
HLA-A	21.9105	21.3246	19.4407	16.07
HLA-F	1.07639	1.15158	1.09726	1.16998
PTPLB	29.8551	24.1909	24.629	26.8073
WDR52	2.51469	1.32671	1.21958	1.92144
ANKRD28	17.5762	15.6511	16.49	16.8231
COLQ	1.16865	1.1743	0.951575	1.266
METTL6	13.3301	12.4061	11.6277	13.066
SETD5-AS1	9.59838	9.8882	5.92546	8.38554
RNU6-354P	3.55392	0	0	0
SNORA57	5.68429	0.851162	13.8068	0.600946
SNORA22	0	0	0.988564	2.8799
SNORA2A	0	1.42313	3.00755	0
SNORA69	3.35396	11.819	2.81948	4.16742
RNU1-39P	0.201748	1.63702	0	0
SNORA22	2.55506	0	1.05808	3.07824
SNORA2	2.82181	1.67379	1.18796	0
SNORA70G	0	0	1.04472	0
Y_RNA	5.51571	0	0	0
SNORA56	3.04321	0	0	0
RNVU1-3	1.01819	0	0.32364	0
Y_RNA	2.46656	1.46193	0	3.11527
RNU6-26P	0	0	3.05278	4.55111
Y_RNA	5.71252	0	2.40659	3.57766

Y_RNA	0	5.25457	0	0
SNORA6	16.8298	15.6822	13.0651	2.45883
Y_RNA	0	0	2.14188	0
SNORA15	1.49443	0	1.29814	1.92001
SNORA32	17.0595	0	0	2.96301
Y_RNA	0	0	0	4.00076
RNU1-138P	1.49292	0	1.26128	1.88549
RNVU1-5	1.43978	0	0	1.21272
SNORA5A	6.83089	4.09248	3.82553	5.63074
RNU6-398P	6.33558	3.79939	2.73311	24.5982
SNORA51	0	2.32126	0	2.41155
SNORA75	6.55006	5.82333	5.47935	0
RNU6-48P	0	8.45347	0	4.51819
SNORA9	0	1.81087	0	3.71158
SNORA70	0	1.73245	0	0
SNORA7	0	1.48354	0	1.55956
RNY1P6	0	0	3.18329	0
Y_RNA	2.46832	1.46775	0	3.11481
SNORD15A	1.85372	0	0	0
SNORA50	0	1.53481	2.15308	4.73017
RNU6-1316P	0	0	3.25986	0
U8	0	0	0	2.31087
RNU6-610P	3.4984	0	0	0
SNORA54	0	0	2.10735	0
SNORA27	20.7313	0	4.10849	0
SNORA72	10.9949	10.7953	10.7468	2.26517
SNORA7B	0	2.56249	0	0
RNVU1-4	1.43978	2.39937	2.4464	1.21272
RNU1-106P	2.87327	0	3.63099	6.28627

SNORA25	16.9641	7.22827	7.06936	9.16583
SNORA18	27.2802	22.0273	23.7911	20.6608
SNORA70	60.2385	48.6043	42.063	55.7486
SNORA68	3.34175	0	0	0
SNORA15	1.73123	3.93375	4.26868	2.0908
SNORA14B	12.7013	7.61075	9.22736	9.60546
SNORA64	5.40423	2.07967	2.08445	1.84629
SNORA42	0	0	1.19297	0
SNORA37	3.63715	0	0	2.19337
SNORA8	0.493229	0	0	12.0681
SNORA2B	7.08116	1.39076	1.98291	0
Y_RNA	0	0	2.50939	0
Y_RNA	0	0	0	5.03535
Y_RNA	0	4.6123	0	0
SNORA20	4.27603	5.02721	3.58243	5.30304
Y_RNA	22.3645	11.2411	7.54851	8.06506
RNU6-1011P	0	0	0	4.8997
SNORA64	12.7208	0	0	0
Y_RNA	2.46656	1.46193	0	3.11484
SNORD15B	8.09575	9.68563	8.09994	22.0956
SNORA19	4.799	5.66026	0	0
SNORA42	0	0	1.30028	0
Y_RNA	0	0	0	5.85743
SNORA7A	1.78447	0	7.77036	4.01977
RNVU1-14	0.647901	2.23055	0.813435	3.57586
SNORA66	0	3.48541	2.47875	3.69277
MIR7-1	4.80948	0	0	0
AC009065.2	0	0	3.30715	0
MIR222	0	2.20716	0	0

AL132780.1	140.153	35.4083	102.156	61.2163
MIR221	2.84511	6.74309	2.40102	5.35925
MIR181B1	0	0	2.90139	0
MIR182	7.26515	0	0	0
SNORA40	0	2.38165	0	0
SNORD94	2.33873	5.52338	7.80371	2.85511
SNORA49	0	1.81138	1.27488	1.86165
SNORA48	5.67138	10.2614	7.3621	13.715
MT-RNR2	2456.42	2973.2	3291.72	3163.62
MT-TA	35.2498	0	0	0
MT-TC	0	0	18.9887	0
MT-TS1	0	16.5959	0	35.4701
MT-TH	24.6401	0	0	0
MT-TE	35.8515	0	0	0
SNORA40	9.13308	1.84381	3.29334	0
C11orf31	129.43	139.14	148.327	133.086
GNRHR2	7.2044	6.69336	5.85621	6.20607
STK38L	12.4633	7.51595	9.20897	8.92141
SACM1L	15.7592	13.9323	11.9009	12.3654
MT-RNR1	1394.28	1410.35	1763.53	1648.16
TSN	76.7397	70.4601	64.685	72.2051
AC092574.1	0	0	2.436	0
MIR671	7.41217	0	7.84375	6.15256
AC010525.2	0	1.60048	0	0
AC090673.1	0	2.51553	0	0
MIR765	0	0	2.06175	0
SLC48A1	9.73807	13.4709	13.1333	13.5301
MIR762	0	8.71071	0	0
IGLV5-52	4.74112	3.76312	3.61923	4.46597

PRR22	8.49415	8.10204	8.06103	7.36859
SNORD67	0	3.1868	0	0
U3	1.025	0	0	0
SNORD17	33.7935	22.7616	24.6497	23.0088
RNA5SP18	3.55442	0	0	2.13388
RNA5SP219	0	0	0	3.2262
SNORD89	7.581	2.24783	3.20556	0
SNORA46	0	1.15983	2.46863	2.44956
SNORA48	0	0	0	1.46577
RNA5SP323	0	1.61682	2.26012	1.66633
RNU11-3P	0	1.47171	0	0
SNORA75	0	0	1.03902	1.52802
SNORA75	0	2.79278	1.483	0
SNORA53	1.67449	1.01405	1.39895	0.847395
RNVU1-13	2.46608	0.841651	0	1.74952
RNU6-460P	0	3.98365	0	0
SNORA12	7.53642	8.73808	7.30036	7.59363
SNORA75	0	0	1.48341	1.4525
SNORA26	9.21719	5.45836	3.88087	5.762
SNORA45	12.0152	7.23312	9.11149	13.3902
ZRSR1	1.14296	0.25575	0.565096	1.08762
RP11-592N21.1	1.51208	5.39099	0.654647	5.60098
AC084018.1	3.77258	4.18848	3.44255	2.89307
C17orf51	2.55259	1.95939	2.19676	2.3298
KRTAP2-3	9.46872	11.218	13.6538	13.6642
KRTAP2-1	1.32853	2.15811	3.07469	2.52434
AC114546.1	18.0311	18.4022	18.1018	20.5867
RPL15P3	111.009	110.584	103.209	102.525
RPS26P3	1.34105	1.1327	0.451111	0.294339

RNF208	13.0547	13.0554	15.6399	14.3664
HSPA1B	39.7578	25.6796	29.3077	31.1874
AC011841.1	1.20397	1.95891	1.87771	1.82937
KRTAP3-1	1.53132	1.41832	1.37052	1.36721
MT-ND4L	6243.39	6755.28	7180.51	5816.15
AL391152.1	5.63031	4.22962	5.45892	6.18133
RPL23AP4	2.90778	2.94815	3.06046	3.0929
RPS26P6	76.9198	67.1442	59.869	62.2348
RBM12B-AS1	1.45068	1.06126	1.10307	0.654934
AC117834.1	1.82399	2.2341	2.12601	2.27212
AC120194.1	59.1167	46.2822	50.9306	57.3845
ZNF580	28.2438	30.4087	30.0876	26.2888
ZNF611	3.68588	3.02828	3.17603	2.5704
SYT3	2.42919	1.6138	2.4777	2.13486
NUP62	58.5	46.8301	50.305	49.0351
COX20P1	4.05341	3.08893	1.88882	2.71351
DENND1B	1.93535	2.04317	2.0272	2.08964
SFT2D2	8.10788	6.96126	7.61908	8.17958
FGFR1OP	13.6446	12.08	12.3461	13.1429
SCAF8	9.70441	10.8279	10.1684	10.8996
RP11-312J18.5	1.47619	1.39027	0.879925	0.555565
AL590867.1	3.52273	3.48047	3.10165	3.5526
TCTEX1D2	7.30705	6.68498	5.60021	6.92649
CRYGS	1.12689	1.13292	0.985866	0.963153
CRIP1	98.2458	94.145	79.765	75.962
KLHL23	25.4514	21.9553	18.0978	22.5389
RP11-641D5.1	0	0.10775	0.0377976	89.3615
FAM24B	3.21788	3.1938	2.55578	3.05596
TRIM59	23.3247	21.5927	20.4084	23.4175

BTF3L4P2	24.2898	24.9369	21.8525	21.3359
ASIC3	0.929989	1.36564	1.28984	1.50335
KIAA1984	1.15949	1.00338	1.19001	0.839164
ARHGEF35	9.62404	8.93708	8.52539	9.2411
DNLZ	26.7044	25.2476	27.2273	26.3933
AC093724.2	1.43849	1.02654	1.25021	1.28242
NOTCH2NL	6.05677	4.39838	4.6307	4.83292
SUPT4H1	153.639	151.364	135.706	142.369
YWHAZP5	1.03343	0.991439	0.827422	0.964021
AC093106.7	1.19585	1.13768	0.513645	0.601504
NRAS	30.4274	27.9153	25.4244	29.9419
AC009237.8	1.10898	1.34204	1.76943	1.45049
HNRNPA3P6	84.573	79.234	72.4744	111.094
LTC4S	0.470263	1.27846	1.06224	0.753611
RPS7P11	2.9875	2.80401	3.04619	3.71908
ANKRD39	27.8744	23.4749	24.8275	23.7767
QTRT1	30.5506	32.7467	34.0224	31.3291
CHUK	31.4795	25.8887	25.9376	28.9962
MXD3	14.3906	15.5195	14.563	12.4981
COG8	33.1891	34.1512	31.9014	34.3347
RP11-577H5.1	1.37914	1.1796	1.05185	0.883038
ARHGAP19	13.79	12.8842	11.9449	13.7545
HAUS7	22.9755	17.6376	20.6623	18.6946
LCAT	12.0557	11.3647	11.6536	9.91034
AC022210.2	0.962684	1.37044	0.327783	0.288396
PTPRCAP	0.766176	1.03728	1.02989	0.835003
KRTAP2-4	0.689024	0.919614	1.07999	0.781017
GPC2	3.1616	4.67567	5.37856	3.99341
KRT222	2.43674	2.06948	1.02746	1.71429

RPL18AP3	96.8763	101.037	88.8746	90.3103
SIPA1	45.1133	43.7772	48.0065	47.398
ERV3-1	1.50657	1.43013	1.09934	1.87127
SYNJ2BP	8.37232	8.95513	8.03784	9.76213
ARL2	45.2032	52.1537	48.2927	46.2577
RBMXL1	6.60896	6.01842	6.09473	6.99485
SRA1	51.9227	53.095	51.4705	49.2532
TMEM110	11.9565	8.06073	8.98998	8.46221
DNAJC9	38.4973	31.1915	29.5108	33.8909
RPLP0P6	1.30571	1.68916	0.708981	0.53563
C8orf82	32.6258	40.9671	42.6616	38.5554
VDAC1	245.155	213.732	210.167	227.719
ZBTB9	7.46496	6.8699	6.8854	6.84796
TMX2	86.3786	72.9946	73.7567	79.1042
RP11-112J1.1	1.80576	1.1515	0.606655	0.825984
SLX1A-SULT1A3	2.4765	3.41835	2.65572	2.43429
HEXA	28.7213	34.3132	38.0817	33.9513
NDUFS3	176.602	141.01	147.308	162.636
RPSAP54	4.85902	3.9806	2.52156	3.09519
LEPROT	14.7701	15.8662	17.1356	16.5965
ADAT3	11.1388	10.6424	11.8093	9.84873
PPP1CB	62.1935	60.0918	59.3802	64.7792
SULT1A4	10.6692	10.3176	11.8635	10.7836
LAT	1.68717	1.42608	1.25506	0.699766
NCKIPSD	21.5841	21.6357	22.7966	22.0336
ATF6B	8.72174	11.3328	10.0204	8.89713
TREX1	18.0093	14.8445	14.9758	15.1739
SLC35F6	27.6884	24.9753	27.3547	25.8881
AC008865.1	3.13714	3.39722	3.45597	3.39425

PIGCP1	2.71851	2.98515	2.78729	2.42867
CLIC1	107.797	101.345	78.1108	72.0087
DDAH2	7.26724	8.31668	6.78476	4.52972
RPS29	3903.57	3788.13	3280.47	3980.4
RP4-694B14.5	0.915693	1.39849	1.67212	1.02749
RPL23AP79	19.716	20.4484	22.2625	22.2298
MT1P1	0.798764	0.945056	0.998975	1.46967
ZNF134	4.17933	3.71044	4.33557	3.89257
GTF2H4	10.6937	11.3406	8.49875	8.21383
DDX47	87.2768	69.4206	74.0455	82.4947
ZNF888	54.7543	50.0738	44.3771	37.1287
ZNF845	2.76551	2.43002	2.37287	2.35711
RP11-3J10.4	14.6214	14.4775	15.904	14.8811
EMP2	11.7337	10.7372	10.3258	9.49263
KCTD11	2.46204	2.6265	2.53298	2.25519
RPL21P75	5.27445	24.6476	25.5923	5.86359
C8orf44	1.97602	2.15117	2.20473	1.93516
RPL13AP7	2.86289	3.41586	2.41802	1.69932
PPM1N	1.21446	1.8684	1.46539	1.63769
SLC23A3	1.24863	1.48694	0.853653	0.831507
LTB4R	9.55033	8.58863	9.87458	7.95774
LIPE-AS1	13.0837	11.2567	8.54022	11.4368
LTB4R2	3.13186	4.96661	4.97396	4.18099
DNASE1	6.18703	8.79259	8.18088	7.97631
MDP1	16.4695	17.0064	15.1582	17.2034
CSNK1E	98.6975	92.1546	100.982	96.6841
IRF9	4.93841	4.77446	4.26865	5.16035
GALT	10.6844	13.7798	14.1914	12.3879
HBE1	61.1309	76.8162	61.5305	61.0593

NUDT19	15.037	15.3077	15.8599	15.6065
RP11-15H20.6	1.00928	1.15178	1.05701	1.15938
CTD-2561J22.2	2.45764	1.31973	0.0765385	0.176168
TAX1BP3	52.0291	56.2657	59.5072	54.8252
AP1G2	42.7383	49.1681	46.6143	46.5889
CARKD	17.9761	17.4375	18.333	19.1187
MEF2B	2.99318	1.24916	2.02039	1.34345
GANC	4.89227	5.88314	6.11658	5.47107
TTLL3	3.92852	5.10589	5.3058	4.01284
REPIN1	58.2549	61.1403	67.5029	64.1289
MRPL23	158.235	138.932	159.863	148.38
SMIM7	51.6951	45.8181	45.3628	51.1958
UCA1	44.2343	40.0561	45.2476	51.5063
FBXO16	7.45995	6.16348	6.51756	8.22287
TSPAN4	47.4001	47.2832	53.8244	47.3917
CPSF1P1	1.9642	2.27602	2.73444	2.46815
CPNE1	177.933	197.416	193.243	192.484
ARL16	52.8085	48.396	50.8415	50.1994
MAGEB1	1.4496	1.36465	1.56154	1.44476
LDHAP4	22.3208	17.1891	17.3681	21.164
LYRM4	27.7888	24.0204	22.7496	24.488
MYCBP	40.8118	43.8661	39.6865	47.8563
AC024560.3	6.15892	4.65072	5.27426	5.94436
ALG3	71.8057	69.8624	74.3072	67.6865
AMZ2P1	6.01019	6.61191	7.09888	6.62446
PLEKHM1P	2.20827	2.47603	2.59809	2.75746
PTMAP5	164.55	134.524	137.126	158.04
SH3D21	1.02549	1.32546	1.07085	0.935032
AC073346.2	58.2293	55.4643	52.0491	58.6671

EEF1A1P12	1.95683	3.54486	1.85911	1.91789
HNRNPA1P10	7.95154	6.94516	6.38495	6.486
AC010336.1	5.40989	4.35818	4.86925	4.83871
FIS1	101.9	107.043	112.669	112.928
ANKRD36BP1	3.96842	2.73024	3.29463	3.51684
ANG	2.36939	1.70707	1.68961	1.83286
RSBN1L-AS1	13.6167	11.4401	11.5673	10.852
SPDYE3	2.96649	3.28207	3.08542	3.19893
MBLAC1	1.45134	1.23035	1.33767	1.25163
RP11-252A24.2	2.53089	2.25207	2.53013	2.37608
NEURL1B	2.37029	2.7501	2.51187	2.4525
HAUS3	8.96798	7.89395	7.97162	8.89048
RPS3AP26	17.1749	16.2522	13.272	15.2669
DKFZP779J2370	1.7122	1.43557	1.40155	1.61037
BBIP1	28.7534	26.9474	22.8024	26.3931
LRRC37A4P	4.29996	5.1455	4.50504	4.37736
RCN1P2	22.5613	26.0269	25.0635	24.4837
PLIN5	0.78353	1.47291	1.3024	1.1755
CTB-13H5.1	0.657993	16.7552	0.411174	19.9956
PPME1	56.5266	53.9283	52.5775	52.4814
KRTAP2-2	1.52852	2.18911	3.16365	2.50448
STARD10	46.7558	45.6208	53.9703	44.6393
HMGN2P15	1.18361	0.429936	0.299684	0.15196
EML6	1.65861	1.43474	1.9235	2.08356
TMEM249	1.24084	2.14923	2.37133	1.6425
AL162389.1	3.1464	3.09588	3.63754	3.01559
HNRNPA3P3	3.09489	2.98347	2.82773	2.75778
RP11-27I1.4	6.7798	8.47546	7.89813	8.48241
ZSWIM8	26.1185	27.057	29.5701	24.1241

AC007347.1	6.74151	6.23229	6.84989	6.85818
IFRD2	81.9351	72.6005	65.63	64.0426
ZBED1	6.13889	6.43504	6.20696	6.29184
AC005562.1	7.38566	6.40191	6.2132	7.03266
TOMM6	363.092	319.953	301.757	364.404
HNRNPUL2	22.4168	19.1128	17.582	19.5776
METTL12	17.3059	16.494	16.0745	15.7923
SEPT7P2	4.0972	3.13672	3.61868	3.56778
POLR2J4	93.3488	80.1144	72.6318	80.9429
AC010468.1	2.1419	2.05977	1.21729	1.29827
RP11-480I12.5	4.83575	4.09465	5.43364	4.38091
DDX12P	5.88903	5.46154	5.8563	5.64551
MTCP1	3.94606	2.66056	3.30008	2.83771
RP11-261C10.3	3.26094	4.52834	4.06549	3.8896
NPIPA8	4.6097	5.23549	4.26042	4.76177
ZSWIM7	26.4878	28.7025	28.2381	29.1452
ARHGEF28	1.8388	1.35466	1.41206	1.51048
LRRC69	0.967201	1.21219	0.823735	1.13038
NPIPA7	12.9747	16.1064	14.9377	14.2425
PARGP1	3.96412	3.21238	3.91526	4.08179
AC129492.6	1.35821	1.64948	1.59666	1.44813
C22orf29	10.1733	9.69776	11.1639	10.2246
PHB2	241.703	221.298	210.85	206.735
RPL13P12	5.4692	6.34945	5.77997	6.06487
CD27-AS1	3.55811	2.80798	2.51452	3.04162
NEURL4	29.7814	29.7484	33.2339	29.7261
AC027763.2	2.09637	1.64233	1.90512	2.01502
BRD2	24.9878	24.3855	24.3343	24.685
UBXN2B	8.93825	9.06438	8.12435	8.74425

CBWD7	16.4735	17.8708	16.9129	18.0322
RP11-292B8.1	1.0446	1.37595	0.957234	1.10733
AC141586.5	2.72278	1.75989	2.23883	2.43259
RP11-1023L17.1	2.59032	2.99264	3.9642	3.88859
MSMP	8.95471	10.8558	8.9461	8.75909
FAM166B	0.589825	0.829308	0.789971	1.18714
LINC00680	1.69485	1.99787	1.86143	1.93348
PEX26	12.5	13.0878	13.5505	13.5445
RP11-266K4.9	2.51289	2.37713	2.63237	2.44495
FASTKD5	15.9676	12.9259	13.7844	15.1908
GOLGA8B	10.0197	10.6198	10.4239	9.06113
DHRS4-AS1	6.7957	7.58049	8.17322	7.83184
HOMEZ	9.94998	11.4012	10.8191	9.41767
HMGB3P24	0.875863	1.18765	0.982982	0.868207
FP15737	1.26668	1.33254	1.61265	1.23097
DDX3X	113.613	84.2814	89.8656	98.9906
CTD-3092A11.1	1.27126	1.0974	1.334	1.4265
VPS16	20.7587	20.5162	22.7414	21.4009
HSPA1A	13.2953	8.7735	10.8657	10.9203
MYL5	2.52346	3.86873	3.56153	3.52163
MIR17HG	2.2128	2.66996	2.32953	1.87685
ZNF407	1.58859	1.75913	1.9231	1.68103
MCM3AP-AS1	2.50257	2.36011	2.12628	2.2032
DDX39B	76.6738	68.2684	72.4261	70.0864
NPEPL1	5.5546	6.55378	7.30458	6.36828
BX322557.10	3.32685	3.59422	3.43508	2.87252
AL136218.1	1.19377	2.22938	0.132815	4.93666
RPL17-C18orf32	22.123	23.4943	21.2761	25.4299
PI4KAP1	3.93695	4.58731	4.60306	4.43874

DDR1	18.8978	19.6791	18.6504	18.2256
AP005482.1	1.35306	0.829417	1.47731	1.09815
AL354822.1	4.62314	5.18306	4.58234	4.95386
GUSBP9	1.96734	2.14869	2.15497	2.0922
TRIM27	5.79827	4.78717	4.73859	5.00977
AC114730.8	5.67018	6.80085	9.42936	7.70491
RSC1A1	1.81612	1.98813	1.91082	2.23409
SRSF10	26.5001	20.5107	21.7749	25.6932
PNRC2	12.5249	6.30616	8.01089	9.44832
TMEM242	6.1074	5.81326	6.5923	6.62698
TMEM167B	11.3116	10.7805	11.4959	12.2673
AC145212.1	1.36234	1.07375	1.46428	1.33419
hsa-mir-6080	1.5536	1.36206	1.38206	1.61555
AC011043.1	14.7683	15.2175	13.5525	14.8469
FAM72D	5.02758	3.8489	3.16927	4.30451
TNFRSF25	11.3639	12.2916	11.9403	11.1947
SLC35E2	2.62785	5.53214	5.5573	4.79203
TSTD1	94.0283	109.802	101.933	109.135
CYB5RL	5.30061	4.92127	5.05294	4.90463
CROCCP2	10.3055	10.4973	11.266	9.90947
MIR941-1	0	2.22188	5.51447	2.33461
MIR941-2	3.79132	2.23341	4.14231	5.82399
MIR941-3	0	2.23341	1.45875	5.82399
RP11-490H24.5	27.5811	26.9467	28.7721	24.037
IFI30	76.5755	77.4296	76.3417	72.4128
RPS2P55	6.00203	4.92082	4.06128	2.65838
AC009403.2	5.8136	6.1803	6.08772	6.37165
PPIAP31	1.27593	1.13079	0.299357	0.240362
FNIP1	3.08511	3.23609	2.90658	3.45613

SYCE3	0.761765	0.314973	1.04466	0.815064
AC091801.1	1.06802	0.152174	0.111625	0.101348
CKLF	37.9915	34.3232	31.4189	32.8009
RP11-204C16.4	1.41141	1.051	0.966395	1.17119
RPS10P3	4.66677	3.45649	2.11561	2.95634
RP11-465B22.3	12.3679	12.7596	12.5897	11.9596
PAM16	105.865	95.9797	91.0621	95.5969
AC016739.2	3.01823	1.80233	0.92405	0.878329
RAET1K	1.04458	0.875108	0.817444	0.717759
AC016773.1	0.905584	0.912128	1.05488	0.807837
RP11-475C16.1	186.008	207.72	222.786	212.379
LINC00339	8.31639	8.49919	8.48049	8.69208
AP000350.4	214.644	220.445	233.262	201.569
AC007390.5	24.9678	26.0081	27.3815	28.5712
ZNF579	22.872	23.6316	25.6987	21.5435
RP11-203F10.6	0.147357	0.440594	3.18151	2.95273
RNASEK	122.792	121.063	121.55	124.594
RP3-337H4.6	1.09217	0.961758	0.5293	0.574533
NBPF1	21.5967	19.0429	17.186	19.2252
FTH1P8	0.864108	1.13473	0.576761	0.504843
AP000580.1	6.56633	4.41499	4.3401	4.60523
AC006465.3	9.77492	9.75419	9.81595	10.6574
FAM228B	1.38569	0.758093	1.12839	1.0383
CTD-2006C1.2	2.93013	2.59632	2.29974	2.57095
RP1-292B18.1	0.654233	0.88598	1.34217	0.82859
ZSCAN12P1	1.02209	1.42202	0.595729	0.735579
AL592183.1	1.59281	1.62747	1.30106	1.60737
ZGLP1	2.20569	2.5678	1.86462	1.83992
VAMP2	12.7353	13.7546	17.1995	17.5123

HIST2H2BD	1.98719	1.89295	2.13182	1.55676
RPL21P28	2.3062	2.2944	1.84349	1.78534
MTMR9LP	1.07071	1.16123	1.05396	0.98515
RPL21P119	21.7867	24.5078	26.2167	22.5821
AC093642.5	1.93427	1.45328	1.57159	2.03257
RP11-572P18.1	36.918	41.766	41.9613	40.911
RPS18P9	1.09009	1.21207	1.07412	1.14456
SNORA11	12.995	7.75794	4.06613	12.0995
MIR1184-2	0	1.41697	0.502793	0
SNORA79	0	1.8446	0	0
RNU6ATAC18P	5.86059	0	0	0
SNORA34	0	24.3868	3.84446	2.86938
U3	0	1.59394	1.87553	1.67629
RNU6ATAC16P	0	0	1.04804	0
AL390071.1	0	0	2.19127	0
MIR1184-1	0	1.41697	0.502793	0
MIR1184-3	0	1.41697	0.502793	0
SNORA77	7.55315	4.39635	1.56117	2.28105
RNU6ATAC	0	2.02125	1.43251	3.21019
SNORA11	0	1.70567	0	1.77027
MIR1307	5.14443	3.55927	0.724184	12.759
MIR1282	31.764	42.3565	17.3261	6.35003
RP11-137L10.6	0.980128	1.41508	1.39721	1.39134
C6orf226	5.94292	6.6087	7.22176	6.3326
PPP3R1	34.1516	28.6609	29.7125	33.3714
FANCG	56.2025	40.601	45.1805	46.195
AP4M1	15.2647	12.6082	13.6356	13.1884
MAGEA3	52.1519	46.4496	47.3258	47.7109
CEBPD	6.07719	6.79381	7.97887	6.93729

ARIH2OS	1.111	0.886578	1.05887	0.963952
C5orf54	2.16439	1.87246	1.85982	1.99444
HMSD	2.26616	4.29741	0.583567	2.33249
NPTXR	3.28767	5.71454	5.34197	5.02937
FAM200A	3.17324	3.5427	3.11497	3.50653
PPP2R2A	54.3459	46.0811	44.1485	50.3205
C19orf73	0.817155	0.914126	1.04023	0.800816
TRIM16	35.3416	32.8055	34.3014	36.3025
TIGD1	1.68147	1.65897	1.47636	1.70161
SLC12A8	12.3646	11.6147	10.7593	11.0237
FADS3	28.0648	31.6982	30.7169	29.8396
GTF2H4	5.67702	5.94772	6.09215	6.37262
CCNL2	49.2335	60.2888	59.8878	55.1532
UBA52	1907.53	1917.87	1838.01	1902.92
PPT2	6.20939	4.49694	6.48615	3.19246
C5orf55	1.21631	1.75883	2.05555	1.64509
BTBD19	0.992324	2.02648	1.3584	1.35151
FAM185A	3.58392	3.24576	3.70335	3.44788
URAHP	6.11566	6.9109	7.31237	6.9041
AC062017.1	0.765891	1.81813	1.41205	1.4173
LINC00152	65.6399	47.1071	54.4596	61.5941
AC079305.10	11.7033	11.3119	10.0783	11.3991
RNA5SP317	0	1.02705	0	0
RNU2-2P	3.02429	2.60528	0.781233	3.8522
RNU6-613P	0	4.36338	0	0
RNA5SP203	0	0	3.18093	0
RNA5SP314	0	1.02705	0.452082	0
RNU2-59P	0	1.72308	1.60526	0.5927
SNORA79	6.47725	6.39409	4.50816	1.31393

SNORA19	0	0	1.80506	0
RNU2-63P	1.12616	0.67163	0	0.700584
RNU2-7P	0	0	0.499485	2.23456
AC008671.1	0	3.59406	0	0
RNU4-46P	3.47222	0	1.4715	0.822798
RNU4-53P	0	0	1.20142	0
RNU4-78P	0	2.8334	0	0
Y_RNA	0	4.27152	3.03757	0
RNA5SP265	0	2.58874	1.79849	0
RNU6-981P	0	4.64473	0	0
RNU6-722P	0	0	0	2.76084
RNU6-516P	8.59028	5.09288	0	0
RNU2-6P	1.9482	0.588501	1.2353	1.21545
RP13-131K19.2	12.0377	8.81066	11.6045	10.8065
FTH1P10	84.1195	72.0418	74.2112	86.9513
RPS18	580.848	582.83	634.886	672.956
SEC22B	3.18653	3.11658	2.72454	2.40939
RP11-91A18.4	6.45785	5.18488	6.66429	6.17761
RP11-393I23.2	1.30126	0.210681	0.393486	0.291842
AC004041.2	4.82293	4.17361	4.61823	5.34862
FAM115B	5.32637	6.38068	6.09409	6.58162
VN1R42P	1.33663	0.33555	0.479639	0.816251
RP11-545E17.3	11.6887	11.654	9.6613	9.8571
TAP2	4.07221	3.3846	4.21145	4.08553
NUTM2A-AS1	24.6676	19.1204	19.6519	22.3323
SKIV2L	2.83512	2.94693	3.56678	3.27367
VARS2	2.81153	3.2894	3.10564	3.39885
EXOSC6	32.8772	28.56	31.9605	30.71
VPS52	8.67334	8.29616	7.4071	6.4381

RPL23AP53	3.03835	3.04221	3.11986	3.88459
RP11-632K20.7	4.32373	3.83628	3.74464	6.33124
HLA-B	30.0848	29.0309	29.2195	28.1435
CCHCR1	2.34048	2.01946	2.25327	2.17033
AC005838.2	14.1772	13.11	10.8336	11.7412
LINC00630	1.33156	1.51595	1.16001	1.3633
CKMT1A	9.13675	12.5203	12.4772	11.791
TINCR	1.24517	1.02652	1.25837	1.50633
RP11-289I10.2	1.15049	0.639663	0.840897	0.862033
VPS52	3.45021	3.40972	3.89286	4.0896
CLIC1	52.4118	48.0831	49.5036	53.1856
FLOT1	10.8945	9.91833	10.6329	9.40769
FKBPL	2.25533	1.97593	1.94262	2.11107
DDR1	19.9888	20.1144	22.3973	22.3265
NSUN5P1	8.92942	11.8311	12.8136	10.8726
RP11-467L13.5	0.918863	1.32477	1.91275	1.74532
RP4-717I23.3	4.65282	7.21681	7.45007	5.86857
MIR503HG	0.378324	1.06948	0.6696	0.436977
ATAT1	1.1103	1.35313	1.4362	1.38592
PRR3	2.32442	2.11681	2.07018	2.19417
RNF5	2.60532	2.89108	2.86084	2.97358
LINC00205	2.6714	3.31961	3.27756	2.9431
SUMO2P1	6.27504	5.57659	5.82932	6.38224
CD99P1	4.09341	3.14139	3.38069	2.99514
MRPS18B	13.4852	12.1971	13.9001	14.9933
RP11-554I8.2	2.70023	3.64998	4.15172	5.27293
ENTPD3-AS1	1.17076	1.19092	1.11469	1.38742
IL10RB-AS1	2.37344	1.71592	2.23619	2.40311
RPS20P14	1.20335	1.37262	0.137419	0.340328

RP6-206I17.1	9.63432	1.78981	9.90113	2.03328
CFL1P1	1.80967	0.759245	1.04021	0.868297
FAM95B1	1.98699	1.8162	2.04636	1.64946
PRR3	2.44249	2.07138	2.04601	2.38344
OSER1-AS1	1.33594	1.7441	1.14766	1.22137
GPANK1	1.11463	1.18345	1.16005	1.33675
NEU1	1.71768	1.13789	1.50624	1.73677
AFG3L1P	25.7737	20.1425	21.2169	19.5246
AC009948.5	4.26699	4.69794	4.3405	4.41885
RP11-84A14.5	1.07585	2.22629	1.91262	1.44748
AC022400.1	0	1.0619	0.898211	0.881865
RP11-728K20.1	3.33145	3.38008	3.20415	3.53042
RP11-383C5.4	1.22558	1.57195	1.56778	1.90093
EPB41L4A-AS1	48.5615	52.3328	48.7009	45.1421
AC016725.4	1.36878	1.26158	1.07094	0.928977
AC005076.5	3.44639	3.10888	2.62106	3.00771
RP1-90G24.6	1.28526	0.93633	0.704829	0.899751
GLTPD1	13.6954	15.885	17.4632	15.1545
AC007319.1	1.38277	0.107582	0.743631	0.344046
RP4-622L5.7	0.957716	1.09522	0.696583	0.871254
UBE2FP1	1.46339	0.981346	1.24176	1.14017
RP11-86H7.1	35.2295	28.3306	37.6812	47.4478
RP5-1033H22.2	1.29804	0.609688	1.31253	0.7945
RP11-578F5.1	5.94141	5.85462	5.92736	6.18495
HCG25	6.66592	7.29406	7.00075	7.45786
RP11-343H5.4	1.87911	1.82351	0.939569	1.43113
C6orf136	2.4106	2.23918	2.52975	2.48798
UBE2SP2	0.11968	0.359068	0.347978	8.36991
EHMT2	5.542	5.01413	5.66977	5.85506

TUBB	135.31	111.042	128.549	130.144
RP11-309L24.6	6.90347	6.40131	6.839	6.7686
DNAJC27-AS1	2.89517	2.41907	2.61437	2.82076
CCHCR1	2.34048	2.01946	2.25327	2.17033
RP11-574K11.5	2.75572	2.40838	2.24947	3.43307
FKBPL	2.13813	1.67752	1.72324	1.87552
TAP1	1.63158	2.12596	2.4734	2.31667
TBC1D3B	0.767851	1.18401	1.81365	1.94572
VARS	19.4096	19.4952	23.0169	22.0664
MSL3P1	12.6062	11.602	11.3342	12.0912
DOM3Z	1.21524	1.50122	1.63814	1.56524
HLA-A	41.3488	41.6413	47.2504	43.0977
RP11-169K16.6	1.70914	2.16358	1.21074	1.06619
MDC1-AS1	1.74929	1.89122	1.38154	1.32964
RP3-486L4.4	2.24623	1.91082	1.51509	1.6986
RP11-252P18.1	17.5397	13.8452	11.0662	15.7944
MICB	3.46055	3.40511	3.6615	3.50765
TCF19	1.91269	1.60756	1.73651	1.93866
CSNK2B	22.6985	22.3681	25.421	24.2222
SLC39A7	14.2103	12.6832	15.198	15.4566
RP5-956O18.3	19.8359	23.9638	27.1818	24.0821
RP11-1033A18.1	2.97708	2.5299	2.53212	2.84283
RP11-351O1.3	1.07868	0	0	0
ADM5	4.63866	3.21403	3.39883	3.3628
PRKAR2A-AS1	3.13776	2.99712	3.39113	2.79616
VPS52	5.01998	4.7101	5.30901	5.54031
AC079354.6	1.89382	0	0	0
ATXN1L	7.54582	4.88263	5.05973	5.40778
TCF19	1.8687	1.61448	1.67608	1.83677

AL355490.1	0.790726	1.38943	0.160102	0.70742
HSPA1B	39.1116	25.5679	29.1629	31.0025
AC002117.1	9.88661	9.75618	10.0744	9.93934
SMIM13	11.6247	8.38042	10.0511	10.6338
RP11-134G8.7	1.07054	1.08795	1.242	1.17321
AC012318.3	6.80924	6.05174	4.73659	5.81779
ABHD16A	2.00667	2.13184	2.73491	2.75353
RANP1	1.03809	0.876902	0.822142	0.972662
HNRNPA1P48	5.12913	3.92299	3.0797	3.58627
MDC1	3.26174	3.58564	4.29976	3.85349
PTCHD3P1	19.6526	11.875	15.0391	15.7159
HLA-B	8.69291	9.44553	9.77835	8.4241
RP11-247I13.8	0.947014	0.565106	0.781668	1.16729
RP5-1142J19.2	0.590629	0.355147	1.00382	0
RP11-51O6.1	1.30914	1.145	0.698782	1.19115
RP11-126K1.8	11.7306	10.4156	9.47978	8.32188
SH3BP5-AS1	0.69088	0.519553	1.02099	0.888908
AC011330.6	1.65102	0.143631	3.51698	0.91259
LAMTOR5-AS1	0.978919	1.61768	1.23945	1.52463
NPIPA3	2.41772	2.77852	2.46534	2.38967
AC099850.1	2.08534	1.82559	2.19528	2.20767
FLOT1	33.2987	30.3966	31.5553	30.893
TAP1	1.93141	2.49096	2.84589	2.69488
CSNK2B	29.3074	29.6107	33.1789	33.4601
PFDN6	22.4394	21.9304	23.1004	27.2626
AC012363.4	2.07044	2.3307	2.40468	2.64626
RP11-134G8.8	1.57175	2.36021	1.73696	1.76804
C6orf47-AS1	1.07315	1.05498	1.20262	1.33446
FKSG52	2.43297	2.28058	2.44684	1.91115

RGL2	2.42467	2.61444	2.91198	2.84225
RP11-123K19.1	1.26459	1.32683	1.07682	0.920032
RPL29P11	12.5871	14.2212	12.3993	12.2712
ZNRD1	13.0506	9.61376	9.70991	10.9278
YBX1P1	28.6031	25.1468	28.3668	25.2068
RP4-758J18.2	18.3906	20.1794	18.0792	17.9973
C17orf89	424.712	396.799	429.954	415.676
IKBKGP1	4.17539	4.52513	5.24749	4.52156
BEND3P3	2.75948	3.39896	2.88922	2.70186
RP5-1142A6.2	2.7254	3.01734	3.61071	2.49691
LINC00863	1.26884	1.49171	1.52744	1.41198
PRRT4	2.30919	1.90854	2.36697	2.23569
TCF19	1.8687	1.61448	1.67608	1.83677
PBX2	4.29911	4.6043	5.35725	5.24187
RP11-206L10.1	4.33707	5.72597	5.1856	5.64295
LSM2	16.4032	13.5798	14.7033	15.8519
RP11-297K8.2	13.1826	11.8817	11.7114	12.1922
EIF4BP7	2.07506	1.75472	2.03251	2.13793
RP11-228B15.4	0.779397	1.06695	0.686944	0.755209
PPP1R18	2.68367	2.10688	2.33835	2.2583
AC021016.6	0.752058	1.12756	1.00671	1.19553
RPL23AP2	19.1396	17.1963	18.7533	18.6575
GS1-184P14.2	12.669	11.6419	9.79536	11.0573
DDX39B	77.0798	68.8685	72.9503	70.6619
RP11-426L16.3	2.02363	2.55917	1.97146	2.42403
SNORA71A	10.2405	4.98839	11.8032	12.0919
HCG4P5	4.55124	2.91937	5.93674	5.33189
AL133216.1	2.02362	2.39037	2.0312	2.03487
PSME2P2	1.24445	0.375469	0	0.389119

RP11-361F15.2	0.952726	0.850591	1.14797	0.957501
CTD-2228K2.7	9.07273	11.2498	12.769	11.1245
AC103965.1	16.9863	15.733	16.3908	15.0822
LINC00618	0.799869	1.0829	0.655395	0.850945
PRRC2A	13.08	12.2928	14.9505	13.3735
RP11-390P2.4	0.596015	1.1449	1.01317	1.0554
AC073283.7	0.965164	0.719944	1.09751	0.733044
PLEKHM1	6.40722	6.7807	6.69132	6.5563
HLA-E	5.96165	6.24159	6.54414	6.54591
AL589743.1	4.89614	5.18136	5.64445	5.24158
RP11-640M9.2	12.4558	12.392	13.8219	13.6489
AC007255.7	1.37265	2.32559	2.18498	2.47923
UBE2Q2P2	1.55727	1.50586	1.20617	1.35017
RP4-758J18.10	0.627841	1.28846	0.859208	1.01297
RP11-57H14.3	0.817285	2.53084	1.87553	1.68987
RP11-539I5.1	1.95295	1.83442	1.98929	2.23675
HSD17B8	1.13393	1.50853	1.52585	1.4469
RP11-415J8.3	1.03739	0.770246	0.822414	1.09767
RP11-513I15.6	8.10219	8.02867	7.7341	8.53482
SNX18P3	1.82114	2.27955	2.26811	2.30033
PPP1R26-AS1	1.15596	1.43002	1.54948	1.45765
WASH5P	54.3893	63.0836	61.5165	53.4883
RP5-1103G7.4	1.73334	2.79895	2.42381	2.35816
C6orf47-AS1	1.07315	1.08123	1.20262	1.33446
AC107016.1	570.776	386.002	357.724	433.52
RNF5	2.60532	2.89108	2.86084	2.97358
JPX	17.528	17.2999	17.9904	20.5023
RP11-773D16.1	27.6803	26.8834	23.29	29.5311
ARHGAP23	19.2589	14.7318	15.2595	16.7383

AC069282.6	3.5746	2.74248	2.4741	2.85371
RP11-396C23.2	0.787111	1.23615	1.49147	0.844948
KB-1027C11.4	1.01091	0.86785	1.0059	0.788778
PHF1	12.2041	10.6609	12.4119	13.3569
RPL35P5	3.60642	2.19505	1.67706	1.15069
NCBP2-AS1	1.32795	1.29653	1.18541	1.18937
MDC1	2.94087	3.19578	3.90472	3.52389
VPS52	6.34104	5.77893	6.25251	6.24734
ZNF469	0.621725	1.36828	1.19286	1.01355
MTND2P28	431.876	429.961	432.085	414.844
DDAH2	2.65476	2.36341	2.98744	2.91297
SBDSP1	31.618	24.7791	24.1075	24.3062
FAM195B	41.7085	46.6373	48.1201	43.1853
LINC00505	0.809292	1.60827	1.61254	1.45262
DOM3Z	1.25611	1.45908	1.63254	1.55407
HLA-C	1.10573	1.07641	1.22219	1.13727
SLC26A6	25.3974	26.134	26.4281	23.3306
RP11-269F19.2	5.53897	4.31964	4.88045	4.58041
FGD5-AS1	26.5565	25.1651	25.7037	29.1126
SKIV2L	3.71062	4.05508	4.85132	4.5747
PRRC2A	6.83369	6.31879	6.99015	7.01546
RP11-365P13.3	4.11913	2.72144	2.8449	2.73535
RP11-592B15.4	1.20245	1.30771	1.30308	1.56392
TRAM2-AS1	2.37518	2.45123	2.42063	2.33191
DNAJC19P5	0.979281	1.06736	0.761054	0.204274
FAM229A	1.5608	2.05432	2.19034	1.96127
ERCC6	2.09094	2.61857	2.98984	2.85599
RUSC1-AS1	22.5614	20.4581	19.3902	20.2425
DDX39B	73.4969	66.3427	70.5719	66.6962

RP11-384K6.2	3.53589	4.63935	3.89447	3.7073
XXbac-BPG154L12.4	1.99652	2.01895	2.00782	2.19552
NOL7	46.2137	39.2776	36.4856	39.1259
NTF4	1.52189	1.3183	1.34857	1.58517
SATB2-AS1	0.616823	0.733604	1.00344	1.23132
RP11-508N22.6	0	0	1.01148	0
TAP2	4.07221	3.3846	4.21145	4.08553
ELFN1	2.12742	2.12273	2.9918	2.5123
MTND1P23	118.731	140.283	141.171	105.866
RP11-139H15.1	8.92296	9.65681	8.72013	9.81883
HAR1A	0.252721	0.323275	1.2821	0.276532
PBX2	4.29907	4.60426	5.35721	5.2415
ABCF1	6.05691	5.33419	5.46045	5.99476
LSM2	0	0	1.43311	0
RP11-211N8.2	0.722264	1.41875	1.15756	0.679041
STK19	0.995961	1.30798	1.16323	1.1602
ZNF503-AS1	1.54266	2.09329	1.63277	1.56849
MEMO1P1	1.38616	1.37321	1.34202	1.53957
TRIM26	1.62366	1.54432	1.7201	1.80153
AC108938.2	13.8259	12.1091	12.8041	12.6841
RP4-706A16.3	33.3942	27.546	20.2742	23.8251
UQCRFS1P1	0.658402	0.815085	1.0033	1.68908
LINC00937	1.35045	1.82373	1.31254	1.75131
SEPT7P3	1.8223	1.74805	1.75388	2.28866
AC004383.3	1.05336	0.381736	0.358014	0.26866
MRPS18B	13.4852	12.1971	13.9001	14.9934
BAIAP2-AS1	3.43375	3.19728	4.57192	4.36764
DHX16	4.61513	3.95511	4.58453	4.72146
TAP1	1.62461	2.04888	2.31941	2.17208

AC010536.1	1.75422	1.60846	1.68997	1.46262
ABC7-42389800N19.1	4.86057	4.80896	4.31607	4.06599
AC022431.1	17.8836	19.5645	19.7596	19.2743
RPS18	580.847	582.833	634.886	672.956
ZNF32-AS1	3.41127	3.98636	2.71185	2.6878
CLIC1	52.4126	48.0841	49.5045	53.1865
STK19	1.01753	1.3652	1.19893	1.1363
GTF2H2B	20.0212	18.7032	16.7575	18.5784
AC009961.3	0.606523	1.07079	0.408223	0.87379
RP11-61N20.3	1.35059	1.49702	1.323	1.44089
PSPHP1	10.4405	10.8903	11.5325	13.0208
TMEM191A	9.98608	10.3667	11.9947	10.0093
CFLAR-AS1	0.700927	0.28874	1.17609	0.535624
CTA-217C2.1	3.18536	3.22666	3.36747	3.54564
RP11-157P1.4	10.9599	9.41889	10.22	9.98356
RP11-119F19.2	4.08469	4.91559	4.65492	5.60874
GTF2H4	3.67628	3.75671	3.9296	3.76253
RP11-31F19.1	2.4717	1.73747	2.89117	2.81058
CLIC1	52.4118	48.0831	49.5037	53.1857
RP11-31F15.1	2.64894	2.62788	2.28403	2.30107
TRIM39	1.18297	1.33082	1.53745	1.41654
AGPAT1	6.96708	7.16899	8.35369	7.95688
CTA-292E10.6	0.998697	1.59615	2.42098	1.78559
TMEM185B	17.2229	17.2575	17.5414	17.1069
CUTA	57.5073	51.3746	57.1281	59.1409
RP5-882O7.1	0.001575	1.68699	0.585443	0.867374
UPK1A-AS1	1.60516	1.25474	1.07697	1.1882
VARS	19.3905	20.8831	21.5263	21.8811
AC007098.1	5.51841	4.99919	7.29511	7.11451

FTLP3	2.72839	4.83709	1.38	3.90526
SLC39A7	14.0822	12.5695	15.102	15.3241
PRRC2A	6.84583	6.44035	7.41974	7.09609
DDAH2	2.85868	2.43304	3.24236	3.01554
RP6-206I17.3	0.377364	0.848999	1.11749	1.13235
CLIC1	52.4113	17.9101	49.5036	53.1856
RP11-137H2.4	0.944865	1.37783	0.913183	1.20715
ENTPD1-AS1	9.14259	7.51769	7.41658	8.2362
RP11-426A6.5	9.83102	8.36811	9.06305	8.12784
C6orf48	21.2236	20.543	22.2706	22.4898
EEF1DP2	5.46839	7.9304	7.86687	6.94862
HSBP1L1	31.6542	24.9637	25.6908	27.896
AC079781.5	0	0	3.45	0
PSMD5-AS1	3.40663	4.18084	4.66192	4.34637
PGAM4	0	2.85082	2.9307	3.21576
RING1	4.77211	4.79056	5.37636	5.56262
RP11-203B9.4	3.27684	3.31803	3.27474	3.6621
RP4-756H11.3	1.32016	0.680227	0.603006	0.775325
RP11-148B18.3	1.10844	1.40979	1.25462	1.03741
AC010894.3	1.10229	1.09093	1.40064	1.65385
XX-2136C48.7	1.13813	0.319678	1.74532	1.31104
GNL1	9.51602	8.71338	9.90089	10.4276
ERVMER34-1	20.9146	17.3242	17.9086	18.9158
LA16c-395F10.1	2.57221	2.24653	2.3104	2.097
WDR46	15.2664	14.9793	17.0458	15.7754
CEP164P1	4.68311	3.73055	3.60178	3.94814
DANCR	135.673	139.011	124.318	133.536
AC078883.4	5.2503	4.79859	5.54351	4.85545
COX6A1P2	10.236	9.36245	9.92708	10.1391

NBPF2P	0.96815	1.17294	0.933837	0.920587
RP3-417G15.1	2.95233	2.88875	2.90891	3.76006
LINC00511	18.2983	25.0015	25.0745	24.4237
DAXX	6.49031	5.68144	6.57613	6.99837
C14orf132	1.67142	1.17413	1.32776	1.56383
WDR46	27.7321	25.5995	23.2938	19.2636
RPL41P1	2206.37	2008.05	1882.47	1899.71
AC107983.4	55.1834	62.4125	66.169	68.5412
CTD-2021A8.3	1.68282	1.7236	1.82893	1.73867
RP11-543P15.1	8.48114	11.39	7.7223	7.76844
RP5-1028L10.1	3.60698	6.23662	4.26534	3.64313
RP11-742N3.1	386.491	443.216	468.854	420.438
NEU1	1.71745	1.1377	1.50614	1.73677
EPPK1	0.924743	1.24338	1.29317	1.16691
C6orf47-AS1	2.9546	2.78765	2.4426	1.93413
DHX16	3.74359	3.67544	4.37651	4.36477
AC017101.10	2.41386	1.89765	1.31105	2.08512
RP11-261C10.5	1.33796	1.29131	1.50018	1.90833
IER3	47.9911	46.055	52.1778	57.9323
WASH7P	17.7196	17.7433	17.626	16.9881
AC009963.4	5.49387	5.9722	4.34752	3.84482
RNF5	2.60532	2.89108	2.86084	2.97358
AC009502.4	1.62184	2.06454	3.36137	2.71943
MSH5	2.49118	2.61595	2.69887	2.69092
NEU1	1.75707	1.07661	1.43775	1.68134
DDAH2	2.65464	2.36336	2.98506	2.91258
RXRB	3.47776	4.86318	4.86586	4.65549
EHMT2	5.56673	5.26907	6.06877	5.80718
PARG	9.76338	8.31247	9.28128	9.32057

RBM26-AS1	1.99543	2.17988	1.9892	1.82915
RP11-112J3.16	1.79375	1.34531	1.96974	1.49662
SLC39A7	13.7907	12.4049	14.8909	15.1576
AC009299.3	7.13568	10.1277	11.0761	11.0563
ZMYM4-AS1	3.28929	4.43621	3.55604	3.93842
MRPS18B	13.4852	12.1971	13.9001	14.9933
ATP5G1P4	1.93794	1.20769	1.31565	1.98062
SYNGAP1	2.14596	2.20972	2.04037	2.53278
AC073415.2	5.58714	2.19867	3.13384	8.16577
TSSK5P1	1.55863	1.3138	1.36212	1.12923
SCAMP4	43.4297	41.4477	41.9264	40.6092
RP11-152N13.5	1.52013	1.47368	1.15436	1.79724
SPAG5-AS1	1.82189	1.59956	1.42587	1.07579
NFKBIL1	1.69535	1.54301	1.77636	1.90203
PPT2	2.11007	2.84039	4.12296	3.61069
RP11-864N7.2	2.16941	2.19406	1.74288	1.39752
CERS6-AS1	15.756	15.3726	16.1662	17.7708
AGPAT1	3.36405	3.40793	4.08271	3.90752
RPL3P2	11.3578	10.4256	9.30322	10.1422
C6orf47-AS1	0.898864	0.911837	1.05469	1.15093
MIR3916	10.6826	10.187	10.9207	10.9197
RP11-73O6.3	3.58855	2.35372	2.8613	2.76157
VARS	14.8289	15.7941	17.712	17.966
GSTM3P1	2.10299	2.34642	1.75381	1.41147
DNMBP-AS1	2.1267	1.59282	1.65639	1.95776
HLA-A	8.21579	42.134	9.04483	7.89324
PPP1R11	2.8045	2.41108	2.81803	3.13626
TUBB	139.191	114.232	130.782	132.979
RP1-20B21.4	85.8039	99.3836	81.4741	97.3563

BAG6	17.4866	18.0149	21.2011	21.8126
HCG4P5	1.25606	1.12472	0.620132	0.531388
MICB	2.24934	2.50969	2.3701	2.43913
RP1-283E3.4	1.23643	2.05261	2.2315	2.28745
ZBTB22	0.884783	1.15542	1.24122	1.13917
RPS18	6.44741	2.80227	4.72653	4.51454
PPP1R10	2.61282	2.59808	2.87033	3.00554
TAP1	1.56657	2.02676	2.28442	2.11559
RP11-958N24.2	2.04274	2.22931	2.31886	1.9824
RP11-156G14.6	3.66202	2.87388	1.52089	2.22896
C6orf47-AS1	1.08342	1.09112	1.214	1.34641
AC007276.5	1.17994	1.10078	1.12788	1.10345
SAPCD1-AS1	0	1.52052	1.33217	1.42319
SAPCD1	1.04397	0.732861	0.692554	0.727857
PSPC1P1	4.03485	3.25617	3.78463	3.93488
AC007383.3	1.22737	1.22875	1.122	1.17289
RP3-323P13.2	0.267097	1.0179	0.659846	0.598493
AC108463.2	2.14685	1.3834	1.32425	2.18223
AC011933.2	2.02926	2.59788	1.20664	1.4324
CTD-2330K9.3	0.717973	0.66277	1.14046	0.691724
POLR2J2	3.97162	1.86693	2.66294	0.683151
RP11-69E11.8	17.009	13.9905	10.7246	11.2777
RP11-222A11.1	4.15343	3.56794	3.48944	4.02945
RP11-452F19.3	5.17827	5.67796	5.83847	6.41554
MFI2-AS1	7.03148	6.24362	4.61338	4.71925
PPT2	8.75586	12.3818	23.04	21.6637
DDAH2	2.65467	2.35819	2.98831	2.9126
SUMO2P1	6.27504	5.57659	5.82932	6.38224
TMBIM4	11.0747	5.36105	8.71387	6.39954

RP1-40E16.11	5.77936	8.68595	8.59161	8.90925
RP1-317E23.3	8.34265	11.4274	11.5337	10.6227
GEMIN8P4	1.42172	1.76086	1.52521	1.55868
PRR3	2.35612	2.07138	2.04601	2.26776
RP11-342M1.3	0.509639	0.959227	1.00168	0.772845
MT-ATP8	3903.27	4487.11	4784.51	4693.69
RP5-1125A11.1	2.43592	1.99485	2.76351	2.53718
RP3-508I15.9	2.36756	2.58581	2.31495	2.39953
HLA-C	6.64716	6.75764	6.53166	6.69908
C19orf24	105.1	93.0438	97.4177	92.233
RP11-186N15.3	3.22325	3.18072	2.89797	3.46058
GUSBP11	6.77314	5.30302	5.52925	5.80126
RP11-206L10.2	1.34811	1.77458	1.50553	1.50773
RXRB	3.21687	4.51172	4.64984	4.56335
RP11-1148L6.5	4.8172	3.99169	4.22338	4.51851
HSD17B8	1.13393	1.50853	1.52585	1.4469
RNF5	7.43729	8.82305	8.64655	9.34143
RP4-800M22.1	1.32773	0.719095	0.561536	0.999925
CCT6P1	2.04893	2.24942	2.03889	2.27486
VPS52	4.6773	4.25187	4.79025	4.72411
TSTD3	1.52199	1.84893	1.65965	1.81839
AP006222.2	0.630286	0.943116	0.865735	1.0576
OST4	211.809	211.366	195.658	196.93
EEF1A1P11	2.67871	5.8882	5.92092	4.84479
AC006460.2	2.92579	2.41853	2.70881	2.16973
RP11-298C3.2	2.04132	1.70995	1.88758	2.17432
RING1	4.7639	4.78219	5.36747	5.55299
CTA-313A17.3	1.43788	1.5198	2.56555	3.13146
MDC1	3.17161	3.16008	3.96434	3.58484

GNL1	9.40947	8.63972	9.66097	10.0914
TAP2	6.15921	5.38795	6.54288	6.21575
SPCS2P4	10.4505	9.4105	6.43858	6.84285
C1orf233	4.13221	5.66213	6.42094	5.76059
RPL39P	2.04182	0	0	0
GPANK1	2.90305	2.36221	2.68892	2.77593
ATF6B	4.21743	6.03022	5.8782	5.64591
FCF1P2	4.14803	3.2466	2.98853	3.01095
AC005682.5	3.1183	4.19549	3.87329	3.97075
PROB1	1.42939	1.78388	1.99296	1.88278
NEU1	1.71745	1.1377	1.50614	1.73677
ARL17B	4.59725	4.7814	4.90515	4.74743
TNKS2-AS1	1.73428	1.75181	1.84898	1.7673
RP5-1160K1.6	4.244	3.98187	4.9658	4.72547
LINC00659	2.03174	3.01309	3.49145	2.59656
HSD17B8	1.13393	1.50853	1.52585	1.4469
DHFR	57.4605	64.0691	53.0465	67.2655
SAPCD1	2.81923	2.44786	1.55454	1.77525
RGL2	2.33507	2.57863	2.86111	2.79283
BAG6	17.4107	18.1711	21.3552	21.8918
MRPL45P2	11.7392	12.7791	12.9727	15.2202
RP11-206L10.11	9.79855	8.22442	9.41705	8.25858
RP11-110G21.1	1.28177	1.30133	1.39128	1.67404
AC073641.2	2.40188	2.87198	2.98704	4.48933
RP4-781K5.2	33.0148	23.993	23.638	26.8247
RP11-112J3.15	4.61869	5.11861	4.7199	5.80143
NRM	1.33981	1.20812	1.33517	1.41891
NRM	1.33981	1.21251	1.33435	1.41245
CSNK2B	27.462	27.0901	30.9158	30.573

TRIM26	1.62366	1.54432	1.7201	1.80153
UBAC2-AS1	1.59687	1.41231	1.82827	1.85458
AGPAT1	5.31692	5.66848	6.31955	6.14508
SKIV2L	3.27314	3.56871	4.25168	4.12821
RNF5	2.60532	2.89108	2.86084	2.97358
HCG4P5	21.1286	20.2699	20.332	16.5627
RP11-567G11.1	0	1.17887	0.688801	0.620493
OR2A9P	1.45075	1.8374	1.73299	1.63654
HLA-B	39.6327	41.2182	45.2999	37.1977
AC133528.2	5.52266	4.38943	5.20872	4.28435
RP11-697E2.7	4.56427	5.65137	5.08893	4.68482
TRIM27	6.10133	5.22643	5.23106	5.64779
EXOSC3P1	2.57292	2.20292	2.46573	2.40406
RP11-313P13.3	0.821604	1.19241	0.968381	1.07308
RP5-931E15.2	0.633962	0.738619	0.788132	1.03094
AC091729.9	3.62568	4.61077	4.85065	5.10432
AF127577.10	6.28909	6.29193	6.25167	6.9283
ATAT1	1.10142	1.29496	1.40293	1.35379
CALM2P2	1.40443	1.39704	1.17054	0.534857
MED4-AS1	11.2354	10.5982	11.3472	11.2141
RPL41	4503.36	4126.31	3874.99	3877.25
CTB-63M22.1	1234.93	1234.11	1208.48	1187.9
ANKRD10-IT1	3.58504	3.22618	4.31486	3.91661
SAPCD1	1.74139	1.10253	1.08185	1.13891
ADAM1A	0.810142	1.05529	0.962681	0.855537
PRR3	2.35612	2.07138	2.04601	2.26737
SUMO2P1	6.27504	5.57659	5.82932	6.38224
RP11-561C5.4	8.20334	7.123	7.42113	6.40836
HLA-A	42.895	43.5664	48.8386	45.1731

RP11-38L15.2	2.05943	1.36459	2.10534	1.74823
MT1P3	1.9663	1.74056	0	0
KRT18P53	0	2.02512	0	0
HLA-E	6.06875	5.95527	6.57118	6.20414
ZBTB22	0.884783	1.15542	1.24122	1.13917
RP11-229P13.22	6.01544	4.60455	5.38637	2.94908
RP11-104G3.2	2.26957	1.79183	2.06961	1.89803
RP11-483F11.7	2.86634	2.74411	2.91781	4.60062
LA16c-4G1.4	0	0	4.99149	2.47664
TUBB8P1	3.46788	3.25749	3.50251	3.49082
RP4-583P15.10	6.14133	5.70496	7.80499	4.81021
C6orf48	21.203	20.5372	22.26	22.4713
KRT8P12	1.73457	1.51098	1.84596	1.39939
RP5-857K21.7	28.7863	32.6785	23.2532	18.7481
NELFE	9.48908	9.22007	9.64937	10.1224
SZT2-AS1	1.68313	2.02507	2.30765	1.99393
AC103801.2	3.76917	5.46386	5.32913	6.14366
RP11-494O16.3	0	1.35834	0.478904	1.41852
AC018638.1	0.859523	0.509776	0.589779	1.04237
RP1-92O14.6	1.91057	1.93289	1.72921	1.99379
GNL1	9.53614	8.72548	9.91001	10.4533
RP1-64K7.4	1.55199	2.75643	2.16078	1.26672
DDX39B	77.9952	69.2714	73.4673	71.1055
RP11-52J3.2	1.30108	1.54229	1.46447	1.74142
RP11-421E17.4	16.5705	17.9116	17.7506	10.1014
AC068580.5	0.692698	1.40784	0.989701	0.813953
BAG6	24.615	26.7771	28.969	30.4367
RP11-119B16.2	1.03071	1.47253	1.69272	1.56522
NKX1-2	2.27111	2.16979	2.33049	2.64707

RP5-981O7.2	2.54461	2.39008	2.43074	2.77928
RP11-492M23.2	1.65826	2.12989	1.46573	0.846173
AC106722.1	2.29937	2.20536	2.33341	2.41378
RPL4P4	4.83018	5.45765	4.85294	4.23483
NAMPTL	2.13342	1.78834	1.93905	2.13995
TUBB	135.33	111.057	128.547	130.143
RP11-21N7.2	74.8433	63.8478	70.9334	72.3499
HLA-F	1.52954	1.34715	1.62325	1.42837
RP11-159G9.5	1.83098	1.85802	1.81114	1.71848
AC019349.5	0.77074	0.717018	1.02858	0.522839
DDR1	30.9773	33.1527	31.8712	31.6783
SLC39A7	13.8763	12.4855	14.9528	15.2338
ZNF688	3.62642	4.11829	5.64915	4.31183
PET100	156.649	159.576	166.666	149.927
ARSD-AS1	0.828629	1.6751	1.92273	1.4699
MRPS18B	13.4852	12.1971	13.9046	14.9934
STEAP3-AS1	2.89796	3.05791	3.159	3.08362
RP11-312O7.2	2.65404	2.78192	2.5243	2.90364
Z83851.1	7.18519	7.95197	8.54619	6.83523
YWHAZP3	4.94762	3.96305	3.32199	3.9589
RP11-111F16.2	3.22654	3.22587	3.07952	5.2197
RP11-284F21.7	12.0126	17.2922	17.0774	13.9343
PPP1R18	2.68367	2.10688	2.33835	2.2583
RP5-857K21.4	0.424442	0.975147	0.724791	1.04054
TMSB4XP6	75.0321	71.141	31.5087	12.6249
CISD3	57.7636	54.0817	58.3697	55.7785
RP11-195F19.9	1.56018	2.04086	2.08029	1.83112
AC016708.2	19.8013	20.8565	13.2272	9.81192
PRRT3-AS1	11.3651	11.0577	13.3067	12.4807

AC091177.1	1.31103	1.77693	1.771	1.46706
RP5-1180C10.2	1.14457	1.3056	1.36278	1.22077
IER3	47.9911	46.055	52.1778	57.9323
FLOT1	6.64846	6.08822	6.38296	6.26825
RP3-508I15.19	7.43395	7.17124	7.08752	6.96794
RP3-477O4.14	0.994762	0.935307	1.04074	0.573506
C9orf147	0.587545	1.00648	0.807023	0.667974
GS1-124K5.2	0.747196	1.46365	0.964128	1.07539
AC118138.2	8.12471	7.49726	7.91852	8.54145
RP11-632C17__A.1	30.4097	35.1708	37.1108	33.8583
TRIM26	5.38593	4.81817	5.54719	5.41891
HLA-E	6.06875	5.95527	6.57118	6.20401
AC008738.1	1.35524	0.651721	0.886013	1.29377
MSH5	2.49705	2.62618	2.71761	2.69571
FEZF1-AS1	1.77072	1.99299	1.99048	1.7705
RP11-385F5.4	1.03771	0.765071	0.918857	0.904626
RP4-635E18.6	4.21192	4.29453	3.89904	3.2496
PPP1R18	2.28583	1.6943	1.93715	1.82853
GOLGA6L5	0.979821	1.11962	1.0712	0.881879
AC092651.1	5.63164	4.47605	4.79625	4.59076
TCEA1P2	1.49446	1.38307	1.23715	1.38434
ANKRD18B	0.949603	1.25606	1.0961	0.814253
DDR1	17.0923	18.3305	17.4455	16.8901
ABHD16A	2.76059	1.98547	2.71048	2.63111
PSMG3-AS1	0.809054	1.05707	1.27164	0.753852
AC108463.1	1.0262	0.618118	0.539107	0.747425
THAP7-AS1	1.60276	2.2274	2.04229	1.90975
LIMD1-AS1	11.4395	11.0019	10.065	10.9339
CTB-89H12.4	18.7874	14.5831	12.5184	15.244

FAM203B	24.8371	19.7143	22.948	18.5454
RP13-228J13.5	1.50786	3.05498	3.62495	3.85617
GTF2IRD1P1	2.81712	2.97183	2.68056	2.92086
FTX	0.75143	1.10747	0.676601	0.717052
AC090804.1	0.841844	1.21818	0.381971	0.391458
AC159540.1	1.7536	1.87452	1.99566	1.75109
DDX39B	73.5151	66.2976	70.5636	66.6643
RPS23P8	361.063	400.346	333.07	425.419
HCG18	2.6936	2.28327	2.50662	2.66031
BRD2	15.2024	13.0532	14.3498	15.5366
ENO1-AS1	0.652118	0.991818	1.03141	0.936598
CLIC1	52.3863	48.0642	49.4766	53.1716
CTD-2330K9.2	4.33402	5.10036	7.10987	5.79188
CSNK2B	27.3828	27.1839	30.6359	30.3322
TAP1	1.66686	2.14827	2.50073	2.34417
RP11-274B21.4	11.7289	10.0429	11.2284	9.96516
RP11-304M2.2	8.2162	10.0505	9.35793	9.14949
RP11-296L22.8	2.08046	1.93634	2.35591	2.06021
AC092171.4	7.17482	6.89578	7.85284	7.46075
RPL10P3	1.16555	0.778585	0.487971	0.766007
YY2	1.44799	1.44336	1.38163	1.43291
RP4-792G4.2	1.04809	0.879132	1.09803	1.05044
ZNF674-AS1	1.27109	1.12006	1.37331	1.27525
RP11-195E2.1	2.08347	1.88057	1.50058	1.84495
AC138649.1	13.2036	15.0699	18.5445	15.7888
SUMO2P1	6.27504	5.57659	5.82932	6.38224
RP11-767N6.7	2.4514	1.7565	1.82468	2.0538
RPL9P8	83.2115	87.8529	81.0831	89.7869
FKBPL	2.67089	2.24465	2.25633	2.49272

RP11-395C3.1	1.07782	0.639772	0.533944	0.91136
RP4-631H13.6	0.786932	1.42698	1.43964	0.00954446
AC079250.1	0.80177	1.23422	0.686751	0.716758
VARS2	2.93073	3.31171	3.55088	3.618
HSBP1	141.833	116.955	97.3265	123.788
PPP1R10	2.61282	2.59808	2.87033	3.00554
TRIM26	3.29125	2.86165	3.15636	3.31238
CTB-54D4.1	2.75293	2.7228	1.77133	3.2184
CDC20P1	4.53346	3.5362	3.40117	3.41407
RP11-175O19.4	5.61219	4.98056	4.67379	4.68205
HCG25	2.23903	2.4422	2.68543	2.70981
NELFE	9.48944	9.22016	9.64969	10.1207
RP11-263K19.4	0.876896	2.71289	1.9747	1.61893
HCG18	3.78512	5.13169	4.26745	3.7
RP11-354M20.3	3.01349	2.60074	1.60996	3.08981
RP3-475N16.1	1.00303	0.786877	0.81624	0.813644
RING1	4.7639	4.78219	5.36747	5.55299
VARS	19.5472	21.0467	21.8081	22.1224
ABCF1	4.85321	4.08791	4.52928	4.65456
RP11-346D6.6	1.66351	1.02763	0.960973	1.20481
MDC1	3.67129	3.99371	4.68659	4.11275
MICB	1.89733	2.16719	1.98633	1.97246
AC005592.2	1.05119	1.05998	1.54111	1.91525
RP11-38L15.3	1.11379	1.05162	1.41534	1.16038
FARP1-AS1	1.10964	0.469653	0.529988	0.394219
MICA	2.24082	1.77975	2.38962	1.8513
SKP1P1	0.578756	3.18717	0.381274	0.745365
PPP1R18	2.21327	1.63176	1.79427	1.7873
ATAT1	1.10532	1.29978	1.40255	1.35155

AC125232.1	8.27695	7.17791	7.24158	6.79164
HLA-DQB1	1.22402	1.72609	2.77445	1.07528
AC007246.3	2.57117	2.27537	3.02217	3.18385
RXRB	2.63907	3.90367	3.97759	3.86196
HCG25	2.1681	2.37812	2.58081	2.55625
RP11-418J17.1	3.26592	4.09075	3.24241	3.22518
PRRC2A	7.2651	6.82189	7.59714	7.53076
MICB	2.48922	2.57939	2.56011	2.70914
DHX16	4.75739	4.04195	4.68845	4.82918
AC016700.6	260.246	247.794	87.9884	94.7042
TIMM8AP1	1.58931	0.704916	0.496708	0.365212
ABHD16A	2.922	3.04856	4.14868	3.66195
RPS18	1013.44	1035.23	895.072	835.09
LSM2	16.4032	13.5798	14.7033	15.8519
HSPA1B	27.956	17.9374	20.9737	22.2123
RP5-1158E12.3	2.55041	1.75544	1.81586	1.64459
FAHD2CP	3.80582	4.74304	4.57581	4.04288
DLEU2	22.9212	16.9358	15.0979	18.0178
TNXB	0.143828	1.45868	1.14259	1.94827
AP006216.11	1.01965	0.638226	0.626079	1.06288
DAXX	8.68731	7.76774	8.8529	9.33991
PPT2	1.87287	2.33697	3.78316	3.10034
TRIM26	1.57186	1.48597	1.66173	1.76958
RP5-827C21.4	1.24606	0.764261	0.672571	1.08608
RP1-203P18.1	1.44742	1.88247	1.83317	1.6041
AC058791.2	1.76409	1.62837	1.91037	1.71996
AC010976.2	2.81899	2.9101	3.01355	3.28624
PPP1R10	2.61282	2.59808	2.87032	3.03673
RP11-112L6.4	1.8291	0.795928	1.00662	1.12404

AC079922.2	0.889396	1.29659	0.68095	0.985409
MIR4426	1.14301	1.13694	0.305735	0.505111
TMEM44-AS1	1.48148	2.47812	2.25578	2.05463
DBIL5P	0.613404	0.396824	0.29203	1.13676
RP11-558F24.4	6.3242	7.29107	7.79484	7.14117
PCAT7	1.19896	1.30936	1.07734	1.26062
AC019097.7	0.869181	0.872158	0.757021	1.00343
PRRC2A	13.6564	12.8986	15.8661	14.2211
AC104655.2	20.6087	19.3643	20.2905	19.9795
RP11-229P13.23	93.0252	74.0035	72.7506	73.2848
IPO9-AS1	1.35811	1.47117	1.84354	2.08214
KBTBD4	5.8258	4.44159	4.33113	4.68506
TRAF3IP2-AS1	2.63103	3.12762	2.30757	2.4792
AC093391.2	1.84015	2.09754	2.47131	2.3266
TAPBP	8.52602	8.15358	7.32876	6.84455
VARS	18.1178	19.3343	21.3729	20.3878
AC018648.5	1.24993	1.38923	1.45219	1.39705
AC007383.4	1.82954	1.64457	2.12978	2.25001
RP11-452K12.7	8.32714	6.71179	5.55908	6.1009
ANXA2P2	1.69294	1.85909	1.29875	1.28185
RP1-85F18.5	0.672241	1.0083	0.584538	0.811703
RP5-1007M22.2	1.25872	1.39735	1.20526	1.30635
PBX2	4.53	4.70459	5.5115	5.39731
AC079781.7	0	0	0.48742	1.40974
EHMT2	5.63482	5.38128	6.19995	5.87626
AC023797.1	0.552852	1.46998	0.805013	1.68501
RP11-307C12.11	1.34442	1.63113	1.60869	1.41846
CTD-2619J13.14	8.34976	5.59031	8.1562	6.80925
TMA7	437.611	439.329	354.132	463.21

MCTS1	69.6704	60.0253	61.7861	68.7521
HLA-B	40.0315	41.6098	43.1427	36.6348
IMPDH1P10	2.36811	2.01926	2.20701	2.06613
GNL1	9.40947	8.63972	9.66097	10.0914
FERP1	7.75697	5.90447	5.98908	6.70612
AC092669.3	1.33109	1.36891	1.49136	1.53181
ABCF1	6.0562	5.3334	5.45968	5.99397
FTH1P7	0.91223	1.25675	0.428046	0.242853
AC008440.5	29.9544	22.2902	24.183	23.6218
RP3-434O14.8	1.03601	1.30784	0.651114	0.218997
RP11-573D15.2	0.42036	0.791756	0.846997	1.02329
FLOT1	7.61828	7.09999	7.52568	7.3016
FAM215B	0.758132	1.17654	1.1486	0.986943
GPANK1	1.11533	1.18371	1.16043	1.33431
AC009299.5	1.02103	1.08291	0.706563	1.14257
AC008440.10	0.916804	1.09359	0.64374	0.839091
TAP2	4.07273	3.38506	4.21197	4.08619
RP11-435D7.3	2.05706	1.26231	1.24943	1.49545
HSD17B8	1.13393	1.50853	1.52585	1.4469
TAP1	1.73062	2.21244	2.48317	2.31894
RP11-66B24.2	2.23001	1.35812	1.96742	2.02183
LINC00493	60.3699	55.9876	57.9664	62.3365
RP1-315G1.3	1.45593	2.14477	0.982382	2.12082
TUBB	135.327	111.066	128.558	130.141
C9orf172	1.00171	1.26142	1.33711	1.07572
CTD-3184A7.4	3.00871	3.47182	3.72841	3.15189
RP11-132A1.4	12.9634	12.0721	11.04	12.2361
RP11-5P18.10	1.21524	1.31067	1.77382	1.47869
AC098820.3	1.15339	1.19619	1.29254	1.56666

RP11-14N7.2	23.4461	18.7887	16.4045	23.3698
AC093673.5	4.33496	4.27387	4.94842	5.23027
GS1-124K5.12	6.7843	6.64254	6.63179	6.50976
GTF2IP1	5.50629	5.78195	5.32011	5.69363
GLUD1P8	0.765472	0.374052	1.15561	0.4739
RPL3P4	10.5129	11.5037	10.0163	9.26004
TUBB	135.314	111.059	128.559	130.133
AC079742.4	44.6677	39.0788	44.5482	44.8855
RP11-46A10.4	1.32463	1.50317	1.27409	1.2763
AC084125.4	5.92841	5.66047	6.42297	5.44383
SKIV2L	3.27314	3.56871	4.25168	4.12821
PRPS1P2	1.25726	0.848161	0.933762	0.881311
GABBR1	0.658826	1.00237	1.09859	0.942202
WI2-3658N16.1	2.19343	2.35441	2.234	2.13014
RP11-126K1.2	0.631559	1.63199	1.65345	1.3572
RP3-437C15.1	50.8598	43.4374	45.1469	51.3946
ZNF668	15.2122	13.4922	16.8498	14.2635
RP11-201O14.2	0	1.35432	0.477336	0
RP11-47I22.3	1.32783	0.946952	0.918212	1.3698
AC078883.3	1.27311	1.12238	1.50709	1.12495
RP11-93B14.5	6.33897	5.73988	6.19768	6.01487
HSPA1B	28.884	18.4051	21.5103	22.8142
RP11-536K7.3	16.131	16.1665	17.1225	16.6084
AF196970.3	4.33261	3.47494	4.19971	4.10305
PET117	11.4335	10.607	9.98809	10.0087
LYRM9	1.59885	3.38404	2.99824	2.72138
RP11-544M22.3	1.318	1.57438	2.22535	0
RPS11P5	1.06682	1.32289	0.568836	0.59467
RP5-977B1.7	3.07386	4.90134	4.54978	3.87393

AC090186.1	1.8061	2.31071	2.25323	1.97289
SNHG15	67.708	58.806	62.0524	64.2667
CSNK2B	27.127	26.8711	30.2997	30.0743
RP11-267N12.3	1.75544	0.72638	1.0457	0.934064
AC034220.3	1.21637	1.09185	1.53775	1.22239
SNHG7	149.851	109.555	114.835	101.129
RP11-1212A22.4	13.5856	13.2897	12.9391	12.5659
CASK-AS1	4.54287	3.56815	4.61457	5.07601
AC097523.1	1.62993	1.74428	1.33587	1.19973
DHX16	4.89342	4.32845	5.2555	5.46291
MICA	2.24082	1.77975	2.38962	1.8513
DDAH2	2.65484	2.36341	2.98702	2.91263
RP11-290F20.2	1.29197	1.85105	1.91784	1.7143
AL672183.2	1.72065	1.38899	1.39296	1.57968
HOXB-AS3	5.07189	4.35556	4.76281	3.58506
AC104451.2	3.21266	1.44985	1.19531	1.45024
RP11-220I1.1	17.3464	19.5004	20.1438	21.6994
RP11-537A6.9	3.25088	3.2204	4.39683	4.68333
GTF2H4	5.65644	5.92927	6.0724	6.35785
HMGA1P8	0.411766	0.811396	0.381351	1.09235
C6orf136	2.41254	2.25656	2.4967	2.45735
C6orf47-AS1	1.07315	1.08123	1.20262	1.33446
RP11-12A20.6	1.385	0.382146	0.635084	0.314011
CTD-2020K17.3	0.501214	1.41155	0.637329	0.6784
RP11-421L21.3	3.56667	5.6851	5.16602	5.2496
RP11-67L3.4	0.946245	0.947297	0.784869	1.22175
GPANK1	0.956853	1.00023	0.981565	1.13929
AC113189.5	9.78276	6.98354	7.67218	7.51542
HIST1H2AM	1.29223	1.28804	1.11558	1.11613

AC079807.2	0.983564	1.31576	0.926034	0.823539
SNRPEP4	0.966311	0.719983	1.52523	1.51498
RP11-12C17.2	0.718524	1.18025	1.23875	0.829635
GPX1	259.344	296.538	300.134	287.657
HMGA1P5	0.897219	2.12654	1.77913	1.92684
PPP1R11	2.80442	2.41107	2.81776	3.13658
TNXB	0.067242	1.30493	1.00045	1.5898
MIPEPP3	2.9336	1.66499	1.74465	2.3089
RP1-12G14.7	1.32878	0.499789	0.491356	0.20767
UBE2FP3	0.832657	1.20232	1.35686	0.703415
MSH5	3.43114	6.49432	5.65188	8.03711
BAG6	24.6614	26.7055	28.8916	30.5622
Z83844.1	2.14132	1.7797	2.24333	1.93492
RP11-396K3.1	6.04643	6.47859	6.08174	6.46553
AK4P3	3.26891	3.19445	3.2021	3.02259
NKAPP1	1.00706	0.614923	0.408335	0.402026
RP11-458D21.1	1.03306	1.06224	0.973937	1.21196
RP11-296A18.5	1.97306	1.27408	1.10395	0.940227
DHX16	4.70833	4.09126	4.66972	4.84228
EIF3FP3	17.6618	20.1243	20.6522	19.9233
HOTAIRM1	6.1694	4.55081	5.3922	4.43069
KIFC1	15.3927	12.7317	14.3357	15.5061
RP11-295G20.2	25.4774	20.5237	19.586	21.8996
EEF1A1P6	260.977	1128.96	1125.47	1183.94
TMEM238	6.12354	8.60605	6.08302	7.56374
AC009963.3	2.25301	2.70935	0.945738	0
LINC00460	5.19881	4.19594	5.38178	6.26483
RP11-57H14.2	5.43985	5.9711	6.47037	8.0522
RP3-486I3.4	1.55032	1.80753	1.31438	1.47416

AC016831.7	1.45694	1.48744	1.47886	1.53259
DHX16	4.70833	4.09126	4.66972	4.84228
PRR3	2.49885	2.19937	2.14332	2.38949
RP4-694A7.2	8.66675	10.0949	8.43082	9.67318
RP4-665J23.1	2.19854	1.75561	1.84391	2.09098
AC079135.1	20.0317	16.4516	15.541	15.8834
RP11-457M11.2	2.60556	2.44445	2.33066	2.6272
C6orf136	2.4106	2.23918	2.52424	2.48798
AC093388.3	0.714965	0.888177	1.19461	1.12798
GAS6-AS1	1.01015	2.08128	2.22694	1.5577
RP5-1087E8.3	9.26331	7.74376	7.76559	7.41995
RP11-109P14.9	24.57	23.6423	24.6643	23.5919
AC007969.5	6.60143	5.62936	4.88629	4.50977
HCG25	1.27812	1.31907	1.1116	1.38878
ZNRD1	5.61511	5.05934	5.41102	5.89569
AC089999.1	47.9187	36.3274	42.6932	37.7012
NELFE	9.48607	9.2171	9.64974	10.1192
MRPS18B	13.4852	12.1971	13.9001	14.9933
HIST1H2BN	1.00925	1.36569	0.857683	1.46451
RP11-135A24.4	6.23402	4.95334	5.41721	4.81843
AC005083.1	1.45782	1.77336	1.67205	1.68054
DPH3P1	6.15654	5.58758	4.62383	4.01072
HLA-C	108.57	115.568	118.713	114.687
RP11-307L3.2	1.01484	0.926064	0.694517	0.719384
AC022201.5	2.466	2.44763	2.18241	1.95331
TTTY15	0.409924	0.94601	0.729083	1.11737
AC007881.4	19.6587	15.7019	20.2174	17.1642
YEATS2-AS1	1.23868	0.860065	1.14286	1.05524
TCF19	1.8685	1.61477	1.67544	1.83633

RP11-65J3.1	1.05082	1.78128	1.44656	1.85451
Z83851.4	1.44506	2.31614	2.63571	2.33841
HLA-E	6.06683	5.95527	6.57118	6.20401
CTC-575D19.1	13.2308	12.5254	5.42716	4.87916
RPS28	1160.67	1271.07	1242.63	1189.96
MT1XP1	3.55634	1.04282	2.19059	1.6096
CTC-338M12.4	2.47082	2.67935	2.83809	2.47676
TRIM27	6.61808	5.16661	5.02482	5.76951
RP11-169K16.7	1.82962	0.493509	0.750963	0.406338
RP11-87H9.2	1.31092	1.2328	1.03126	1.61702
UBE2SP1	25.644	18.4868	17.6043	17.3781
RPSAP14	9.32783	11.9039	12.5844	11.1642
PPP1R18	2.28583	1.6943	1.93715	1.82853
MDC1	2.97155	3.25229	3.95026	3.54976
VARS2	1.70904	2.1741	2.39292	2.35224
TRIM26	1.56452	1.51176	1.60706	1.73473
RP11-247A12.1	1.0466	0	0.293528	0
PPP1R11	2.8045	2.41116	2.81782	3.13656
AC074117.10	5.29869	4.49678	5.39517	4.36021
DDR1	18.9419	19.6721	18.6588	18.2332
AL391994.1	0.766324	0.233619	1.43106	1.10291
CCHCR1	3.61252	3.05082	3.47252	3.36457
TRIM26	4.03607	3.81952	3.16635	2.54649
RP3-460G2.2	14.8047	15.0796	13.7388	11.9549
RP11-613M10.6	28.6957	17.9942	19.1972	20.7726
AC108488.3	6.02792	6.4126	5.99044	6.26207
RP5-1050D4.2	0.999172	1.49972	1.49599	1.64396
MICB	1.54656	1.67385	1.62844	1.58201
AC093616.4	10.7314	8.47651	8.7471	8.70903

AP000936.1	3.66454	3.55251	1.63964	2.26661
RP11-761N21.2	8.96913	9.34846	10.4092	9.65862
H2BFS	1.06015	1.14464	0.934612	0.924708
AC012146.7	19.2283	18.7755	20.0417	20.7593
NEU1	1.71768	1.13789	1.50624	1.73677
AP000347.4	0.784446	1.09166	1.45133	0.225366
RPS26P47	1.2637	0.654971	5.73896	6.1665
GTF2H4	4.84425	5.18694	5.36599	5.53828
ZNF37BP	1.64203	1.95425	1.56503	1.33825
AC007283.5	2.19986	2.19281	2.5412	2.69123
RP11-706O15.3	7.02324	8.37922	8.37988	7.76122
AC006960.5	0	0	0	1.1141
HSPA1A	15.4191	10.1491	12.5099	12.4706
RP11-275I14.4	5.66024	5.49169	5.73645	4.86825
HLA-F	9.05813	5.7038	3.57212	5.45945
AC003665.1	1.51461	2.56641	2.37345	2.44966
TRIM27	5.79785	4.80032	4.73791	5.03532
RPL13AP20	3.62814	2.8827	3.50349	2.93963
BRD2	15.9112	14.4656	15.6338	15.0834
SUMO2P1	6.27504	5.57659	5.82932	6.38224
NFKBIL1	1.69535	1.54301	1.77636	1.90203
RP11-288G11.3	5.54481	6.25104	5.26047	6.5961
ATF6B	5.4705	7.07554	6.54339	6.41722
FAM133B	20.622	21.3884	18.3566	19.9156
ATAT1	1.10087	1.29496	1.40293	1.35379
CCT6P3	1.62301	1.7126	1.77144	1.8209
MCIDAS	3.93406	3.06139	3.39079	3.60607
AL356740.1	23.1767	21.7558	32.9716	26.4903
MAPKAPK5-AS1	18.5613	16.4778	16.8099	19.7493

JRK	3.88076	4.88203	4.89488	3.93558
SNRK-AS1	1.34988	1.66242	1.39198	1.49995
RPSAP9	1.39002	1.43487	0.76577	0.878222
AL162407.1	1.42239	1.46472	1.66058	0.832643
MED14-AS1	0.932652	0.60129	1.24625	0.96548
AL162151.3	3805.52	4223.87	4660.47	4552.85
BAG6	17.6499	18.1155	21.266	21.8763
RP11-262H14.3	1.85568	1.54558	2.30225	1.90792
TCF19	2.10363	1.65378	1.78074	1.91849
RP11-465N4.4	1.44914	1.55278	1.42577	1.10064
RP1-92O14.3	1.92507	2.0798	2.06413	2.15002
BRD2	20.5458	18.6303	19.6636	19.3677
HMGA1P4	1.44278	1.55815	1.26106	1.32564
RP11-166B2.1	6.93444	7.54745	6.66544	7.074
C6orf48	21.2236	20.5373	22.2706	22.4847
GAS5	371.343	420.14	363.904	362.311
HLA-B	44.1675	43.6636	37.0856	33.3242
RP11-328M4.2	5.54584	3.11293	3.35305	5.15142
WASH4P	18.1756	17.7752	20.0524	18.1109
RP11-395P17.3	1.58591	1.0974	1.24346	1.03752
RP11-483H20.4	5.02512	3.77249	3.86315	3.52593
AC114730.7	1.72235	2.11479	1.65671	1.49471
RPS3AP6	17.1212	16.8539	13.4842	17.749
DOM3Z	1.2198	1.46718	1.61194	1.57313
NRM	1.33981	1.21153	1.33517	1.41957
AC092687.5	3.24624	2.67985	1.80071	3.62533
NEU1	0.463319	1.12272	1.47707	0.225028
RP11-3P17.3	216.54	202.54	195.858	238.511
RP11-831H9.16	27.0283	24.8219	25.3667	25.7576

LINC00338	11.8893	8.20562	9.8083	5.82472
RP5-994D16.3	1.1246	0.701701	0.584142	0.897073
AC010883.5	0.753174	1.18238	0.713169	0.471784
AC109828.1	14.0228	11.6639	13.012	14.4892
STK19	1.03945	1.32625	1.26948	1.29124
AC104667.3	1.36675	1.5151	1.39477	1.51379
SAPCD1	1.08995	0.908428	0.783461	0.864154
SHISA8	8.40818	9.18034	9.22533	8.63567
TBC1D3C	1.39723	2.27213	1.96949	1.74356
FTH1P2	142.358	155.126	177.946	151.24
RP11-493K19.3	1.45713	1.99038	1.88595	1.79293
AC068580.6	1.71983	2.31494	2.40501	2.41316
IER3	47.9911	46.055	52.1778	57.9323
C19orf81	6.40731	8.76718	10.7082	8.00513
RP5-1099D15.1	1.82095	1.59066	1.59583	1.44291
RPL24P2	1.18368	1.45477	0.995907	2.32136
TUBB	135.31	111.057	128.541	130.146
AC068138.1	0.764866	0.852514	1.07533	0.913023
AC142528.1	3.63215	2.80208	3.46744	2.97428
LINC00094	11.0295	11.8609	11.7474	11.249
RING1	4.57873	4.63756	5.16991	5.36993
ZSCAN31	4.55416	5.57002	4.25513	4.3569
RP11-9M16.2	0.951001	0.559677	0.38806	2.83737
NFKBIL1	1.14346	0.969526	1.07963	1.57721
C12orf75	85.5976	78.2457	71.305	78.707
SMIM1	1.68762	2.60763	2.67903	2.2039
FAM203A	22.4964	19.7831	20.6388	20.1877
RPL39P3	169.861	162.816	76.3192	62.102
ZNRD1	4.53594	4.23861	4.31327	4.51226

PPP1R3E	1.08812	1.56152	1.09753	1.41285
HLA-F	1.31498	1.27052	1.12743	1.53758
MSH5	2.42681	2.67056	2.77737	2.47764
MICA	9.80216	8.80443	8.4517	8.91016
RP13-131K19.1	2.74417	3.07243	3.3036	2.68447
SUMO2P1	10.0225	9.07955	7.92258	7.80297
AL162759.1	5.15188	5.68289	4.13693	5.10358
FAM103A2P	14.4592	15.6726	13.4521	14.8714
PPP1R10	2.61282	2.59808	2.87032	3.03673
RP11-575L7.8	1.99749	2.91766	2.33681	3.53809
BRD2	16.5794	14.7133	15.9675	15.4748
PVRIG2P	7.07119	6.25614	7.46229	7.18781
AC114730.11	2.5583	2.99293	3.47697	3.22787
CTA-276O3.4	1.92679	1.69832	0	0.585936
SSR4P1	0.316087	0.710352	0.664083	1.07602
MRPS10P1	9.39785	10.8348	7.55736	9.98078
LINC00623	11.7521	12.2693	11.0461	11.1233
SNORA71B	8.28458	3.08303	9.21095	1.59498
RP11-282O18.3	3.00639	2.46612	2.73961	2.77316
RP11-342M3.5	0.95864	1.2558	1.33433	1.65171
WI2-2998D17.2	5.56733	5.87868	6.06239	4.79535
RP11-357C3.3	2.04368	1.63591	1.51178	1.60498
DDX39B	62.5128	56.4436	59.9702	58.6434
ZNRD1	5.51933	5.10059	5.35314	5.76077
TOPORS-AS1	7.0134	6.2347	5.5471	5.37843
RP11-678B3.2	1.63201	1.14225	1.12991	1.37407
RPS26P31	0.930729	1.45677	0.339735	0.504467
RP11-122G18.5	1.8603	2.11883	1.89184	2.00233
AC073046.25	3.2505	2.81941	2.93595	3.04192

RPS2P7	3.00678	3.05853	2.76954	1.46843
TAB3-AS2	6.16488	6.41737	7.52112	7.76062
RP4-756G23.5	4.649	3.39299	3.57609	3.18162
C6orf47-AS1	1.08338	1.09108	1.21391	1.34636
AC009505.2	10.5804	8.27119	10.3216	9.31469
AGAP1-IT1	1.78568	1.05412	1.09444	1.21397
RPL6P27	46.2168	43.5829	40.2955	45.8973
NOL5BP	1.282	1.26732	0.734343	0.782522
AC002310.12	1.43905	1.10215	1.47407	1.56752
MSH5	5.47786	6.28352	7.93887	7.34354
XXbac-B476C20.10	0.677314	0.677004	0.380489	1.41154
NUS1P1	2.14015	1.54673	2.56552	2.208
RPS18	580.847	582.83	634.884	672.956
RP11-545I5.3	3.19408	4.07295	3.98808	3.91001
H3F3AP4	2.94151	3.82766	3.26888	4.51372
HLA-A	70.9824	43.3878	73.6843	69.9838
ATAT1	1.10587	1.29978	1.40255	1.35155
SAPCD1-AS1	0.564649	2.87357	1.9515	1.97082
SUMO2P1	6.27504	5.57659	5.82932	6.38224
ABHD16A	2.92685	3.04988	4.13544	3.65421
PFDN6	22.4802	21.9068	23.3287	27.3268
DICER1-AS1	0.824786	1.41977	1.18438	1.26235
RXRB	2.71659	4.0527	4.0957	4.02701
HCG18	3.66605	3.24938	3.03674	2.93604
KIAA0040	4.36254	5.30582	6.38153	6.2
AGPAT1	3.80114	3.91649	4.60623	4.43173
NRM	1.33981	1.21159	1.33517	1.42002
LINC00263	2.73858	3.31588	2.80083	3.15803
AC073333.8	5.57971	3.9158	5.71817	5.90522

AC006978.6	0.789935	0.722511	1.03445	1.03622
RP11-335O4.3	3.18131	4.10896	3.40103	3.79169
AP001468.1	2.02694	1.7355	1.99581	1.15456
AC012379.1	36.4846	32.5476	32.8126	28.6557
LINC00941	6.13379	6.18503	5.61262	4.67723
TM4SF19-AS1	2.58003	2.48595	2.47705	2.42127
RP1-159A19.3	3.97802	5.20292	1.962	3.74412
ASH1L-AS1	1.22621	1.05273	1.24346	1.15542
AC007405.8	1.21794	1.01566	0.856494	1.14846
HSPA1A	14.6858	9.65964	11.9503	11.8831
TTC28-AS1	10.9238	9.00913	8.55835	9.75493
COX7CP1	0	1.74422	0	0.454776
GNL1	19.401	17.9929	19.9246	21.3866
AC011747.4	0.792212	0.690435	0.465968	1.1259
GPANK1	2.78532	2.37385	2.67007	2.74041
VPS52	4.88065	4.64962	5.29674	5.45045
ASMTL-AS1	1.30437	1.28242	0.843937	0.946606
AL953854.2	0.786489	1.19623	0.980291	0.842247
MYCBP2-AS1	6.07966	5.16754	5.23507	4.73112
HSPB1P1	73.7013	60.3755	53.6312	59.6784
ABHD16A	2.93413	3.07417	4.14441	3.81917
AC074389.9	36.8999	40.984	41.6251	40.6529
COX10-AS1	2.4125	2.48563	2.55611	2.4876
RP11-148B18.4	1.31451	1.08175	0.985159	0.942499
ZBTB22	1.38858	2.14734	1.90404	1.5372
AD000090.2	11.1022	11.6029	11.7892	11.369
ABCF1	6.0562	5.3334	5.45968	5.99397
VARS2	2.99521	3.79649	3.60306	3.85709
NFKBIL1	1.69535	1.54301	1.77636	1.90203

KDM4A-AS1	13.5655	16.7613	16.1439	13.6032
WDR46	14.7309	14.974	17.0774	16.0004
BRD2	12.8268	2.18883	1.21204	1.58011
STK19	1.03346	1.3819	1.27271	1.11
AC009404.2	2.06855	1.62111	1.65628	1.28508
RP11-263K19.6	22.2156	21.7493	22.5513	22.49
RPL26P30	9.97882	9.59161	8.26927	9.90673
FLOT1	21.2925	24.7833	29.546	29.3699
AC114737.3	1.31182	4.32014	1.64591	5.36616
ZBED5	13.8181	13.6723	13.5841	14.4644
RP11-126L15.4	1.44654	1.4419	0.685453	0.803015
RP3-486I3.5	3.53591	4.78729	4.05155	4.87729
AC015987.2	79.4084	66.3657	67.2219	79.9919
ABCF1	4.85321	4.08791	4.52928	4.65456
NFKBIL1	1.69535	1.54301	1.77636	1.90203
PBX2	4.26536	4.52805	5.29697	5.13745
DDX11L2	2.48477	2.40997	2.62339	2.16753
PPP1R18	2.23887	1.79093	1.9591	2.09241
AC097662.2	0.558236	0.82033	0.57839	1.28174
RP11-175B9.3	6.47682	8.44548	4.59148	4.45017
AC002401.1	1.91734	2.3718	2.12516	2.07489
TAPBP	4.85115	4.84901	5.99302	5.71225
AC107081.5	49.9102	51.8212	46.2506	55.2201
EM:AC006547.7	2.22942	2.83777	2.06879	1.37934
RP1-63P18.2	0	0	1.34522	0
ZBTB9	6.22938	5.97915	6.40316	6.97164
RP11-732M18.3	2.33542	2.26433	1.88249	1.53793
AC006547.13	1.98383	2.29928	2.58751	2.62472
RPL13AP5	10.4331	10.9655	8.63098	8.67984

PPP1R11	2.86544	2.45157	2.88037	3.22877
HNRNPA3P5	0.874647	0.044335	0.735853	1.63727
RP11-162J8.3	0	1.48374	0	0
PITPNA-AS1	7.31644	6.00963	7.14821	7.21307
CCDC163P	2.35982	1.73746	1.51631	1.70668
HLA-E	5.96288	6.24159	6.54414	6.54553
PPT2	2.06325	2.55921	4.03738	3.37714
RP11-108L7.4	1.75881	2.58149	1.88417	3.07072
PRKG1-AS1	1.55618	1.37899	1.22667	1.52145
RP11-69I8.2	9.15919	7.85775	9.64112	10.6012
MTX1P1	12.0347	11.279	11.3442	10.1593
EIF1AXP1	6.80073	6.83143	3.65504	2.93432
RP5-1024G6.2	9.15377	11.6748	11.905	10.8826
AC018642.1	1.75466	1.72175	1.47117	1.94139
DNAJC9-AS1	31.4031	33.2605	35.1215	39.4623
EHMT2	5.55905	5.26322	6.06109	5.79947
DOM3Z	1.04566	1.17433	1.33813	1.26842
INTS6-AS1	0.839903	0.912603	0.670896	1.12325
RP11-96L14.7	88.4285	73.2631	70.6559	71.2214
ZNRD1	4.95046	4.69215	4.89745	5.1068
RP5-886K2.3	13.9292	12.1171	12.8646	14.1453
BCYRN1	1.34708	0.803194	0.627889	1.17339
LSM2	16.1509	13.3119	14.6981	15.7245
NRM	1.33981	1.21153	1.33517	1.41447
LL22NC03-80A10.6	12.5499	11.5353	11.9189	14.0511
AC018737.1	1.42922	1.49842	1.29472	1.34068
LINC00106	0.502346	0.988263	0.584287	1.04539
AGPAT1	3.21209	3.40849	3.81691	3.6997
AP001615.9	6.06259	5.55396	6.49322	5.66891

GTF2H4	5.89795	6.18048	6.23354	6.83673
MIR600HG	1.65128	1.4663	1.47627	1.41559
RP11-1008C21.2	0.633934	1.03567	0.842285	1.01483
RP11-390F4.6	0.996393	1.79614	1.78634	1.57576
RP11-439A17.7	1.54569	1.599	1.76679	1.59233
RP11-640M9.1	10.5345	9.49887	9.12278	9.55937
ZNRD1	5.01205	4.63005	4.86712	5.00432
SUMO2P1	6.27504	5.57659	5.82932	6.38224
RP11-383C5.7	1.88652	1.64172	1.80097	1.43732
ZNRF2P1	2.27688	3.33027	3.14057	2.84696
C6orf136	2.4106	2.23918	2.52975	2.48798
CTA-984G1.5	1.31495	1.22515	0.974305	0.803017
HLA-C	2.23417	2.49928	2.33778	2.32445
ZEB1-AS1	1.43572	1.65125	1.63822	1.79333
NDUFA6-AS1	1.35012	1.53661	1.65567	1.66369
AC018738.2	83.7382	90.69	94.0628	93.5819
PRMT5-AS1	4.62045	3.71328	3.70496	3.60093
ZBTB22	0.878658	1.15683	1.24488	1.16396
TRIM27	5.61386	4.55955	4.37872	4.88319
COX5BP6	1.73052	0.690536	1.69962	1.0771
RP4-669L17.10	13.6189	16.7495	16.7254	14.6985
MDC1	3.25777	3.59032	4.29774	3.85316
C6orf136	2.4106	2.23918	2.52975	2.48798
GABBR1	0.657159	1.00031	1.09707	0.941018
AC073254.1	3.72911	3.25741	3.58193	3.98984
ZNF503-AS2	22.3594	22.6793	23.7536	24.6097
IER3	47.9911	46.055	52.1778	57.9323
B3GNT9	3.26522	3.39274	3.37987	2.85997
NR2F1-AS1	10.5722	10.6257	10.5634	10.1241

CDKN2AIPNL	45.4984	41.9548	40.5549	43.8231
FTH1P11	2.39046	2.27078	0.93323	0.845548
ANO7P1	1.65775	3.4603	2.62348	3.03096
CKMT1B	13.976	17.4264	19.8469	15.5546
SMG1P1	7.28711	7.46309	7.15205	7.40128
TTN-AS1	3.15479	2.62355	3.23396	3.30728
GS1-124K5.4	3.40092	4.74206	4.85539	3.47095
RNF223	2.11441	1.92244	2.20408	2.04244
MSH5	1.41875	2.00489	1.97316	1.49301
PFDN6	22.4394	21.9304	23.1004	27.2626
PBX2	4.54417	4.71852	5.52204	5.4222
RP11-475I24.3	1.07697	1.63472	1.48399	0.945659
PITRM1-AS1	1.11248	1.10864	1.11551	1.1586
PPP1R11	2.80446	2.41107	2.81776	3.13658
AC069257.6	1.73429	0	1.451	0
FOXD2-AS1	1.95256	1.89987	2.21823	2.11407
CECR7	2.55194	3.31785	3.16023	3.02136
RGL2	6.30127	6.1732	5.89	5.14454
TRIM27	12.2835	8.36896	8.80588	10.6623
XXbac-B135H6.15	0.926319	1.43496	1.36275	1.13371
SGOL1-AS1	4.50475	4.05759	3.65148	4.58598
RP11-206L10.9	0.752283	1.23028	1.17036	1.16542
RPSAP15	10.2855	9.17087	7.90522	8.7287
HLA-F	1.42764	1.19097	1.18871	1.35109
AC008268.2	4.63655	4.99214	4.70561	4.59795
UNC5B-AS1	0.843263	2.51085	2.51514	2.21704
SHISA9	1.0347	0.895431	0.894485	1.01888
LINC00857	6.92773	7.39552	6.96671	7.39964
RPL9P9	83.2139	87.853	81.0837	89.7877

GCSHP3	5.12368	4.39837	4.4161	5.64997
AP000251.2	1.91836	2.62246	2.76626	2.55858
TAP2	35.0854	30.4221	32.3476	33.4372
AL353898.3	0.544273	1.12057	1.90124	1.80253
OXCT2P1	1.83896	1.58103	1.84481	1.46249
KIFC1	24.754	19.7903	18.1746	17.7809
AL627309.1	4.92534	5.84316	6.23846	5.019
RP5-1120P11.1	1.67828	1.03634	0.925248	1.25188
HSPA1A	14.6775	9.64675	11.9397	11.8748
AC002075.4	1.70407	2.74215	0.354719	0.70755
RP3-423B22.5	4.51507	4.11023	1.3602	1.23787
BANF1P3	23.9832	16.2178	12.5655	17.2825
FAM200B	14.0342	13.9248	11.7681	12.7603
AC002066.1	1.90895	2.63219	1.51029	2.28376
AC007000.11	1.91792	1.14634	4.64654	0
AC002454.1	1.35826	0.675974	0.617122	0.457787
RGL2	2.42467	2.61444	2.91198	2.84225
PPP1R11	2.28746	1.93425	2.26406	2.55922
RP11-216M21.1	1.80052	1.4501	1.07047	1.5734
LINC00674	2.97927	3.26695	8.81319	7.36744
RP11-435O5.2	2.22141	2.24844	2.54492	2.68593
DGUOK-AS1	11.1503	9.72538	8.98091	10.2247
RP11-611D20.2	1.52485	2.30425	2.35582	2.25612
DDX39B	47.749	45.0516	48.2442	44.1957
RP11-288I21.1	2.8282	3.31382	3.14709	3.57262
PRKCQ-AS1	5.85852	5.27134	4.26178	4.55343
hsa-mir-6723	213.276	268.612	244.752	193.034
RP11-385J1.2	4.8307	6.4029	5.55425	5.26962
AC093110.3	0.91673	1.16594	1.02199	1.76689

AC138035.2	8.61079	10.6379	10.2813	8.9944
AC009133.14	70.7299	62.6404	63.0183	62.6821
RP11-432J22.2	1.05635	0.866157	1.13171	1.14591
HCG25	0.815313	0.650913	0.754681	1.05823
RP11-305M3.2	14.3138	14.0382	12.8731	12.487
LRRC37A2	2.76844	2.92282	2.90241	2.64112
RPL9P7	2.44181	2.36878	2.02663	2.8111
PPP1R10	2.61282	2.59808	2.87033	3.00554
GOLGA2B	2.20789	2.96086	2.74425	1.9878
RP11-262H14.1	2.98824	3.14194	2.39973	2.3324
CTA-941F9.9	1.36338	0.639066	0.619296	0.80896
MID1IP1-AS1	1.32926	2.98995	3.23816	3.44643
EHMT2	5.63482	5.38128	6.19995	5.87626
RP11-431J24.2	6.2699	5.03373	5.51852	6.57383
RP11-472B18.2	0.825123	0.62175	1.01683	0.780621
C9orf69	16.9209	24.1731	23.8441	21.709
BX470102.3	1.6019	1.81346	2.13183	1.53683
MICB	1.54427	1.76196	1.68076	1.63945
snoU13	7.61555	0	0	0
SNORA13	3.7034	6.56737	1.57052	0
snoU13	6.82112	0	0	0
SNORA81	1.21723	2.92317	0.524396	3.90504
RNA5SP311	0	1.02705	0.452082	0
U1	1.65249	0	0	1.02496
snoU13	0	0	4.39323	0
TRNAI2	6.72279	0	0	8.08185
SNORD97	6.22665	4.56008	6.08693	0
Y_RNA	0	0	2.31092	0
SCARNA12	5.24889	5.74284	7.04898	5.88654

snoU109	0	0	0.973689	1.44276
snoU13	0	5.1061	0	0
SNORD10	13.8211	11.8752	14.3052	8.69183
SNORA47	0	1.31205	1.85681	1.36949
SCARNA10	0.350542	0.624068	1.31093	0.741774
Y_RNA	0	4.48967	0	0
snoU13	8.3381	0	0	0
RNU2-11P	1.2276	0	0.517983	0
SNORA59A	5.99812	6.55603	6.91137	11.1823
SNORA84	5.36264	3.16598	3.37215	1.67575
RNU1-123P	1.20042	0	0	0
RP11-85F14.5	1.68196	1.55496	1.54598	1.97965
RP11-464D20.2	4.99903	3.91288	2.6094	2.44684
TXNDC5	123.051	107.43	98.2386	107.547
GATSL3	1.96814	1.95325	1.76417	1.29759
LY6G5B	1.08232	1.04226	0.771401	0.806272
RNF103	4.79027	4.53349	3.95423	4.44305
RBM14	75.4841	75.3709	85.4555	74.873
ALKBH6	21.0366	20.4456	23.8917	20.6733
ST3GAL6-AS1	1.46506	1.11956	1.16044	1.05138
AC007405.6	1.25605	1.94637	1.61233	1.64532
LY6G5B	1.08469	1.04324	0.775083	0.811197
GATS	2.4234	2.83165	3.76763	3.0316
MYLK-AS1	1.12036	1.14052	0.941774	1.45061
RP11-325F22.4	2.1322	1.85355	1.86481	2.09365
RN7SL573P	0.70874	0.702579	1.18296	0.433808
GUSBP4	1.41589	0.495314	0.373685	2.5728
RP11-295P9.3	2.32155	2.28416	3.31873	2.49729
NME1	788.126	723.719	730.178	812.519

TNFSF12	7.74528	7.85026	9.03449	9.42169
HLTF-AS1	2.35901	1.62209	1.77692	1.83791
PHF1	3.2302	0.396685	0.551538	0.364467
WBP1	16.5762	20.572	16.7084	17.9096
MRPS17	36.6966	32.1306	29.176	36.1516
RPL21P39	0.561094	1.11494	0.606718	0.593138
GET4	43.3741	39.7471	44.0068	40.4812
RPP21	22.1575	20.3789	22.5257	21.2444
RN7SL608P	2.37547	1.71148	1.08494	0.64207
C1orf226	2.42409	3.14181	3.10821	2.9853
ADSL	110.667	107.422	107.602	109.443
RP11-34P13.14	4.37394	6.19505	5.99084	4.63411
RN7SL75P	1.17245	1.81913	1.07824	1.16538
PRKAG2-AS1	1.09757	1.47371	1.47299	1.34216
RPL39P36	0	2.13141	0.760767	2.2681
RPP21	8.04689	9.45488	10.878	7.76935
RP11-163E9.2	4.6855	4.42016	3.75678	3.77888
RP11-293A21.1	0.801978	0.955605	0.327459	1.98514
LINC00888	2.83629	2.94475	2.89706	3.31231
RP11-587D21.1	1.6818	1.36911	0.776832	0.479499
LY6G5B	2.55264	2.92324	2.46874	2.02353
RP11-254B13.1	43.9302	39.6922	42.8484	39.2277
RN7SL146P	1.29364	0.995885	0.388165	0.570765
RP11-103G8.2	0.485508	0.764049	1.11911	0.794708
RP11-745A24.1	0.850238	0.307928	1.16184	0.754889
SMKR1	5.03637	6.48049	7.4692	6.34278
RP11-758P17.3	31.2578	29.3955	24.566	25.8475
COX19	6.80352	6.05489	6.35943	7.0104
ACAD11	4.92857	5.5573	5.03506	5.12338

RPS2P5	224.958	225.638	180.431	220.1
PPIL3	43.0653	40.7753	37.2255	44.0818
RPL23AP7	7.08403	6.41489	5.47671	6.48084
AP000304.2	2.85992	3.052	2.03456	3.03758
RPL13P5	4.74547	3.58914	3.51599	2.99376
RP1-228P16.1	1.15738	1.2987	1.90228	1.50304
FOXO3B	0.696152	1.94125	1.83198	1.86777
LINC00973	2.71226	2.29723	2.52813	2.66986
SETP14	1.52677	1.05139	0.797087	0.963985
PSMB9	0.784547	0.78064	1.03817	0.702774
RP11-429G19.3	2.72971	2.40797	2.56917	2.27909
RP11-247I13.7	1.20855	1.27506	0.933761	1.3492
RP11-479I1.4	2.30812	2.804	2.41925	2.30058
RP11-247I13.11	3.03368	3.24978	3.57458	3.62391
RP11-64D22.2	3.3256	1.80534	3.04544	1.94362
RP11-206L10.5	4.40177	6.64843	5.09263	4.43919
RP11-366M4.1	1.34313	0.988172	0.685496	0.326335
ISY1	41.3926	36.9836	36.3904	40.9903
RP5-890O3.9	13.7435	10.5061	11.2129	11.0684
RP11-155G14.5	2.39372	2.2607	1.69028	1.76257
RP11-25I15.1	0.29766	1.07638	0.124037	0.370778
ARHGEF25	1.36413	1.47287	1.36641	0.993866
AC132217.4	5.62939	6.4315	5.59352	5.24456
TMEM189	31.1672	30.1377	27.7314	28.7507
RDH14	15.2126	14.6273	13.1868	13.5533
NDUFB2-AS1	1.85397	1.91874	2.15486	2.18586
PLCXD2	4.75511	3.73864	4.55165	5.56625
AP000356.1	2.65424	2.96738	2.95034	2.46549
RPL15P2	2.63981	2.03229	1.96356	1.8354

MIF	636.909	620.024	630.418	556.249
HOXA11-AS	2.93486	2.9918	2.67187	2.593
TPM3P9	2.11497	2.08932	1.90445	1.98313
RP11-727A23.1	3.34851	2.52708	3.14869	4.72043
NSUN6	6.29294	4.84922	6.83313	5.97281
CTD-3141N22.1	10.3494	5.27855	8.51929	12.2768
AC008280.3	0.167214	0.201485	1.06876	0.320857
YAE1D1	14.6333	13.8729	13.2068	15.1772
RN7SL809P	1.35316	0.811634	0.762929	0.568871
RP1-89D4.1	2.21021	2.28752	1.97808	2.05514
CRCP	29.2556	22.9803	22.5924	24.4229
WWTR1-AS1	5.81152	4.32773	6.90022	7.70202
CDRT1	0.287268	1.01567	0.896406	0.762008
RP11-807G9.2	2.91792	1.72754	2.12375	2.17357
RNA5-8S5	207.16	218.244	268.518	176.466
RPL36A	634.276	560.142	473.135	621.284
RP11-758P17.2	2.11497	1.45896	1.42795	1.99531
PDXP	38.3494	32.0151	32.2273	32.408
RPP21	30.3918	28.854	25.2055	22.3742
EGFL8	1.73708	1.17176	1.20004	1.4204
RP11-512H23.2	0.973833	1.8342	1.7681	1.41466
RP11-461O14.1	1.67472	1.76953	1.44828	1.45384
RPL39P38	1.80528	0	0	0
ATP5J2	690.065	693.229	627.437	705.206
PTPRG-AS1	2.59136	2.96095	2.64873	2.99383
ARHGAP8	30.7215	29.9897	31.8588	28.4447
AF011889.5	1.50361	2.12779	2.08685	2.11985
PSMC1P1	9.23946	6.79071	6.6105	7.42312
RP1-130H16.16	0.769594	1.40129	0.995385	1.09474

ARPC4	190.852	124.144	167.291	176.106
RP11-490G8.1	3.27767	3.12285	1.89559	1.74703
RP13-585F24.1	2.51623	1.69127	1.2569	0.77164
RP4-697K14.12	1.11697	1.66877	1.25028	0.726487
RP11-381E24.1	3.53877	1.88413	2.43636	2.07862
AP006222.1	2.50491	2.076	2.39239	2.16798
ARPC1A	106.546	118.409	114.669	118.162
TMEFF1	1.1459	0.896408	2.09236	1.92466
AC002467.7	2.30731	2.66632	2.40197	2.83481
AC092620.2	0.745137	0.729009	0	1.04654
RPP21	4.989	4.49921	4.57668	5.05859
ATP5O	305.166	289.404	273.276	293.489
PLEKHO2	4.67685	3.7206	3.71049	4.15563
C8orf58	8.51013	7.96614	9.11736	8.84714
RP11-34P13.13	2.33004	3.16736	3.15374	2.75514
RPP21	14.0539	13.4708	15.0472	17.0915
PISD	35.7401	38.9858	45.7642	40.404
PWP2	62.212	57.8165	57.877	56.9954
CTC-340A15.2	0.584769	0.741968	0.637235	1.0244
C2orf15	20.6866	22.1114	22.1513	24.4509
PI4KA	23.9531	22.1454	25.8544	22.8539
TCEB1P19	1.08151	1.31442	1.31187	1.15653
AKAP2	17.4145	15.5204	16.1039	17.3383
RP6-109B7.3	23.4285	18.7458	14.2548	18.4804
USP27X	1.9888	2.01831	1.90127	1.98252
HYPK	12.5318	8.9327	12.7066	9.60933
EGFL8	2.22331	1.41656	1.59271	1.80199
RP1-90G24.10	1.32537	1.83532	1.81225	1.52057
LINC00969	15.9504	15.4903	15.4675	15.1969

AMACR	2.10797	3.04032	2.83672	2.97986
MTFP1	39.3092	30.6922	34.6118	36.4776
SNHG3	38.0037	29.0775	37.4752	35.4378
RP11-134G8.6	0.568221	1.08398	0.783534	0.592615
AC000041.8	9.12822	7.91152	10.3225	8.86573
OR51B5	6.55329	7.15305	8.17782	7.67604
RP11-568K15.1	1.90723	1.91871	1.72032	1.79461
HOXB-AS4	1.47076	1.88326	0.824587	1.83021
ARFGAP3	6.26408	6.04581	6.45355	6.56958
RPL39P34	6.83906	3.09283	5.27425	4.20374
C22orf39	19.9548	18.1251	19.1231	17.6981
RP11-62J1.3	4.03926	3.79714	3.45971	4.02778
PEG10	3.56225	2.95329	2.75047	2.90994
AC108488.4	4.05713	3.10428	4.07328	4.3622
STAG3L5P	2.70325	1.44642	1.57259	1.7043
RP11-234A1.1	2.25874	3.06682	1.56602	1.7538
EIF6	320.02	280.74	303.465	284.201
RP11-498P14.3	1.46553	1.43136	1.04667	1.24273
TBC1D3H	0.955744	1.2793	1.13473	0.922041
RP11-67L3.5	22.4015	16.1425	17.607	17.7438
RN7SL800P	1.86771	0.744766	0.778503	0.954071
AC093627.9	1.5988	0.978333	1.00082	1.03164
MRPL20	186.001	166.147	155.573	173.4
C15orf38	3.11077	3.79771	3.70001	3.49731
RP11-299J3.6	0	1.83304	1.2885	0
RPL21P11	1.05807	0.747854	0.374504	0.459346
RP11-274B21.1	1.40061	1.41811	1.48441	1.12373
RP11-54O7.14	0.755352	0.82275	1.24846	0.976706
DECR2	8.62255	8.47136	9.52304	8.53981

GNG10	37.6	39.6641	37.1065	41.0389
AC009228.1	1.76553	1.05393	1.35095	3.04227
RP11-508N12.4	0.421259	1.88634	0.333878	0.808204
PSMB9	0.784539	0.780641	1.03793	0.702772
RN7SL529P	0.704903	0.805971	1.27436	0.957505
RNA5-8S5	207.16	218.244	268.518	176.466
RPP21	5.6172	5.1956	5.61825	5.70811
RGAG4	0.968702	1.28368	1.2063	1.30506
AC018462.3	0.810965	1.19631	0.387499	0.244406
GLYCTK-AS1	2.29842	2.83368	2.26101	2.85273
AP5Z1	10.8054	11.5106	11.2783	10.065
RP11-285F7.2	0.754226	1.22181	1.11857	1.02492
FTH1P23	5.11154	5.32928	4.55164	3.8355
AC068522.4	1.68407	1.24873	0.321918	0.240785
RPP21	4.98886	4.80542	4.91454	5.05857
RN7SL737P	6.07054	5.20572	4.25379	4.86341
RP11-613M5.2	1.85342	1.59256	0.920263	0.75635
MRPL33	88.9998	84.8076	73.8868	89.6034
RP11-538P18.2	0.960683	1.00709	1.19749	1.04883
AL592188.1	38.4305	51.8163	80.3942	59.3896
MICAL3	9.2138	7.85072	7.13577	6.1475
RP11-408P14.1	2.23264	2.43211	2.06271	2.32162
RP11-286H14.8	1.30209	1.3213	1.4043	1.301
PCDHAC2	0.854047	1.02139	1.01052	1.00479
RP11-384F7.1	0.832279	1.44422	1.47388	1.76237
PRAF2	12.1699	8.21008	11.6005	11.7981
RP11-274B21.2	3.66626	4.18631	3.93131	4.1789
C7orf73	58.3023	60.0138	57.0656	69.9827
KCTD7	4.74987	4.81721	5.30532	5.14268

RN7SL738P	1.09125	0.65941	1.19416	0.684399
EFNA4	9.05237	9.90161	9.23428	8.78417
AC012442.5	0.649606	0.629714	1.01082	0.648882
C4orf48	194.555	193.77	221.903	183.127
NBPF15	4.09465	4.66231	4.53185	3.98214
NAT6	4.01537	3.96574	3.65726	3.31971
RP5-1121A15.1	4.77319	5.41927	5.54681	5.25351
TNFRSF6B	47.2807	69.0949	96.724	73.2955
RP11-740N7.3	2.81259	1.02944	1.86272	2.43319
AC004967.7	3.78526	4.49008	3.57458	4.59044
UPK3B	3.21887	2.93364	3.73209	3.54035
RP11-281P11.1	1.0927	1.35548	1.34934	1.14365
IL10RB	9.99769	9.17998	10.4199	9.94633
ZNF487	1.70627	1.63614	1.73929	1.81256
WDR92	6.8534	7.0487	6.81546	7.7789
NME1-NME2	531.196	525.016	509.081	488.053
RP11-274B21.3	4.70531	5.89611	5.34686	4.77702
PLA2G4B	7.16451	5.26888	6.98873	3.75705
NPIPB5	16.92	20.3505	18.5149	17.1286
TTC4	33.8901	27.74	24.7516	27.7413
ZMYM6NB	13.2573	16.9537	16.6236	17.0919
AC006547.8	19.8855	16.159	17.5864	17.5896
JMJD7	3.89301	7.22272	5.4603	5.17715
RN7SL56P	1.05797	0.75394	1.49459	1.29989
OR2A7	1.85645	0.79211	2.39273	2.10813
RP1-63G5.5	0.555005	0.492432	1.01861	0.768143
TIPARP-AS1	1.10743	1.9113	1.07055	1.12907
MRPS6	48.1868	41.4164	42.9301	47.503
ZNF512	14.5937	14.0827	16.1166	16.462

PSMB9	0.784542	0.780661	1.03818	0.70277
RP11-552M11.4	44.8918	37.9187	43.8869	40.6365
ACY1	25.5014	33.4955	32.6361	29.9703
NFS1	33.5413	31.5917	32.2455	31.6724
RP11-50D9.1	20.3906	21.6553	23.4945	19.6052
FAM86DP	4.84921	6.25703	6.35177	5.91347
DDOST	302.497	308.281	312.767	314.892
TMEM199	20.814	19.5666	19.5333	19.8871
DNAJC25-GNG10	0.325148	1.32691	2.35032	1.26239
RP11-148K1.12	37.6478	30.8607	32.5321	30.1631
P2RY11	16.386	13.6088	15.787	13.8305
AL592188.2	38.4305	51.8163	80.3942	59.3896
RP11-314A20.2	0.876724	0.94696	1.02029	1.01738
TMEM141	54.9041	48.7976	54.2443	53.2213
RP4-545C24.1	3.91308	3.85286	3.68709	3.91367
GS1-259H13.2	1.29631	1.02167	1.10776	1.09045
RN7SL151P	0.55517	0.831524	0.806415	1.02016
AC007009.1	3.85901	4.5564	4.2393	4.01444
RP11-120B7.1	1.06344	0.64039	0.350563	0.371462
PKD1P1	3.55248	3.53314	3.48046	2.78687
SIAH2-AS1	1.00452	0.840084	1.2113	0.855506
RPL32P29	12.0043	20.1224	14.8519	14.64
DBNDD2	44.3409	40.7849	42.2937	41.8475
RP11-475N22.4	7.66221	8.57694	9.26021	8.10519
CTD-2314B22.3	3.39333	3.48335	3.68203	3.12105
RP11-425L10.1	11.3843	10.082	7.90586	8.90338
RPL7P23p	3.37354	3.50814	2.18734	1.66524
RP11-466H18.1	161.616	155.78	76.273	67.0899
ETV5	17.3138	19.9184	16.7372	16.5972

RN7SL268P	0.597028	1.92278	1.25998	0.995824
EGFL8	1.88833	1.79277	1.97324	1.3762
RP11-1398P2.1	1.2961	1.12055	1.36962	1.62499
RBM12	60.1595	43.3036	49.642	53.7495
RP4-798C17.6	6.68798	6.78383	6.30906	6.76558
SCARF2	0.735949	0.839132	1.1601	0.946831
RP3-508I15.18	16.0495	17.4883	16.8713	16.7188
APOBEC3C	24.3309	30.4753	31.7669	30.1758
RP4-800G7.2	3.06698	3.53662	3.50315	3.01859
AC006011.4	52.8141	45.12	43.8884	45.2565
AC096772.6	0.967121	0.92896	0.902371	1.20732
CTA-211A9.5	1.55596	1.35061	1.49547	1.52262
RP3-449O17.1	0.768888	1.16411	0.817844	0.675101
CTD-2377D24.6	0.749196	1.45047	1.10068	1.15719
AC108676.1	0.852645	0.788759	0.906265	1.05491
UBE2V1	103.853	104.671	94.8068	107.093
CTAGE8	2.32078	2.30294	2.23653	2.55962
RP5-894A10.2	1.25203	1.36562	1.25983	1.62998
CD46P1	1.42902	1.23656	1.64205	1.4029
RP11-20O24.4	106.93	92.4405	74.4368	87.2483
RP11-506M13.3	1.50175	1.17502	0.944005	1.22106
N4BP2L2	19.4911	19.9631	19.9716	18.6098
GABPB1-AS1	4.21192	4.6721	4.17331	3.21825
LINC00847	5.62554	6.28367	6.30206	5.42686
SMARCA5-AS1	27.0294	24.7024	28.4177	25.2006
RP11-383J24.5	1.13789	0.785127	0.662726	0.817886
EEF1A1P4	1.05451	1.03385	0.457259	0.569568
RP11-10K16.1	0.863762	0.997593	1.05752	0.875977
USP2-AS1	4.02454	3.32863	2.68926	3.34481

RP3-330M21.5	0.605571	1.24112	1.10014	1.07522
RP11-286E11.1	0.885134	1.01014	1.1935	1.35871
RP11-334E6.10	38.6478	35.0875	37.8366	39.577
NEAT1	21.7764	41.0367	35.7116	31.4557
CTD-2037K23.2	1.03212	1.09963	1.30551	1.42007
AP001258.4	0.574113	0.927178	1.13475	0.975563
BDNF-AS	7.66792	5.99511	7.44475	6.92214
ZNF585B	1.72296	2.15213	1.75344	1.82655
CRNDE	6.89548	7.12609	6.72123	7.68752
RP11-367J11.2	20.7755	20.69	21.7038	21.6373
RP11-410D17.2	10.9412	9.5587	12.7289	10.8738
CEBPA	3.30802	2.33065	3.09133	2.97954
RAD51-AS1	0.872093	1.00004	0.782915	1.25207
SNHG6	384.702	362.63	326.745	374.041
CTC-228N24.3	15.9623	17.158	16.7252	17.3425
RP11-33B1.1	5.92407	5.37818	5.67319	4.44603
KB-1208A12.3	53.3311	49.708	51.0027	48.687
RAB30-AS1	6.48486	6.32446	5.7649	7.84315
NUDT16P	3.07267	4.90136	5.23513	4.93766
RP11-115C21.2	3.54171	4.05119	4.33679	4.52277
CASC8	14.038	12.1452	13.1095	12.5544
KB-431C1.4	1.78702	1.97668	2.20598	2.0545
SBF2-AS1	3.48319	3.68803	3.29415	3.28565
CTD-2036P10.3	1.3947	1.34561	1.14448	1.71393
PRR7-AS1	4.03627	3.06469	2.53882	2.47819
RP11-382J12.1	1.71634	1.68196	1.96237	1.94654
RP11-10L12.4	1.39697	1.49669	1.20579	1.43203
RP11-1149O23.3	13.0574	13.7871	15.28	17.2803
H2AFJ	12.5363	11.7664	11.2427	11.4145

AP000487.5	6.50155	5.07515	5.11136	5.45603
RP11-252E2.1	1.72472	2.34259	2.24894	2.07482
CTC-338M12.7	7.50824	7.07252	9.04123	8.61902
PGAM5	161.986	139.102	142.742	141.784
RP11-318M2.2	1.42601	1.89531	1.88079	2.02859
SNHG10	13.4033	9.61162	9.43745	8.41196
MIR210HG	0.909029	1.21713	1.25732	1.41107
RP11-11N9.4	7.68507	10.2961	10.2105	10.727
RP11-727A23.5	7.34556	6.49596	6.71207	8.39298
RP11-296I10.3	7.02631	4.67427	4.50108	4.75568
UBL7-AS1	2.12227	3.33449	2.77128	3.01015
ZBED5-AS1	5.40045	4.92167	5.59364	5.74134
AL034548.1	8.73791	11.5344	8.48205	9.69281
RP11-392P7.6	4.69347	5.35667	4.54149	4.06853
OIP5-AS1	27.6184	23.5368	25.4249	24.2684
CTC-281B15.1	3.80285	3.49375	3.38729	3.54034
SPTY2D1-AS1	1.2409	0.809726	0.711866	0.382947
TWF2	48.3976	55.5706	54.3114	50.3948
MARS2	12.547	10.1428	11.2637	11.8955
MTND4P12	1.26068	1.21985	0.836462	0.528569
STX18-AS1	1.25676	1.51899	1.23763	1.57884
CTD-2366F13.1	2.459	2.82018	2.9707	3.16836
NR2F2-AS1	2.49589	2.40021	2.19098	2.57876
TMEM161B-AS1	8.98295	7.6083	6.71805	8.30019
CCAT1	1.9301	2.07467	2.52844	2.77386
RP5-940J5.6	6.14068	5.20718	5.43514	6.06033
RP11-421F16.3	3.24138	3.29051	3.70657	3.65029
RP11-79P5.2	21.7757	14.075	19.2712	20.1565
DYNLL1-AS1	6.70588	6.53748	6.12571	6.07224

AC005329.7	3.27267	2.90436	2.98833	2.75475
RP11-453E17.1	3.35254	3.571	3.55125	3.86184
RP11-159F24.1	2.77503	2.86464	3.04644	2.95836
BCKDHA	14.5069	18.4803	18.3253	15.7985
AC002116.8	0.634007	1.33322	1.52794	0.74228
RP11-78C3.1	5.08452	4.38977	4.89807	4.6944
RRN3P1	2.09965	2.26851	1.98711	2.43221
CTD-2012J19.2	23.0557	20.3564	16.686	15.4679
RP11-499E18.1	2.43823	2.77694	2.45201	2.25482
AC037459.4	2.88218	3.04743	1.56139	2.24033
AC092597.3	1.23072	0.915791	0.64756	0.733101
OCIAD1-AS1	4.53172	5.29176	4.41522	3.93598
CTC-338M12.3	13.6304	10.7251	11.5685	12.4379
CDK11B	50.1234	44.1612	45.4772	48.5839
LINC00504	1.56154	1.71876	1.625	1.29167
RP11-177B4.1	1.33308	1.56559	1.36005	1.81781
PRR5-ARHGAP8	1.9514	1.65733	1.83829	1.90629
OR51K1P	1.56312	1.10494	1.54369	1.25516
CTB-118N6.3	1.91775	1.4074	1.52784	1.70523
AC004510.2	5.24011	4.39202	5.65367	4.66182
ABHD14A	19.1791	22.457	22.2279	19.4933
SRP14-AS1	2.50318	2.71558	2.78204	3.06679
MTATP6P1	723.687	824.557	892.179	703.95
RP11-10A14.5	1.20891	0.749516	0.905842	1.45011
RP11-159F24.6	0.541271	0.577739	1.01542	0.657876
TMEM110-MUSTN1	1.53391	1.82065	1.39739	1.07248
DSTNP2	2.5533	2.31366	1.89289	2.2179
AC003029.1	5.63968	6.09348	4.08822	2.68951
RBM14-RBM4	3.30151	2.29597	2.05975	2.44854

CTC-504A5.1	1.75222	1.44851	1.72886	1.42675
CTC-498J12.3	2.64443	2.33477	2.18976	2.44945
OXCT1-AS1	4.79361	4.87998	5.07252	5.07226
RP11-798M19.3	11.4352	13.0458	13.5061	12.571
AC009238.6	8.76674	6.1706	5.82303	5.34536
AL357673.1	3.94158	3.49227	3.60822	3.53348
RP11-227H4.5	1.04177	1.52103	1.49489	1.3559
USP46-AS1	0.821489	1.29053	1.13887	1.03456
CTC-366B18.2	1.54397	2.14822	1.74973	1.97172
RP11-481K16.2	9.68029	9.53252	8.04132	9.08985
RP11-319G6.1	0.61348	0.946244	1.24689	0.785234
CTD-2015H6.3	1.30599	1.57647	1.34189	1.59672
C1orf213	1.52436	1.34584	2.26613	1.97974
HAUS5	16.915	14.7336	15.792	14.6121
CTD-2194D22.3	1.88969	3.1512	3.62931	2.69339
CTB-36O1.3	2.99024	0	0	0
RP11-360F5.1	2.10214	1.18034	1.68834	1.91319
AP000304.12	6.82863	8.05212	5.33022	6.85743
EEF1A1P9	0.6305	1.26589	0.369045	0.341947
AC068533.7	2.91238	1.65479	3.19705	4.75133
UGDH-AS1	2.23626	2.60386	2.48056	2.54836
NPM1P27	1.59219	1.13924	1.12028	0.543193
NAIP	1.17567	1.15748	1.38688	1.16142
RP11-298J20.4	6.8287	5.03506	4.47534	4.65307
ZNF286B	2.22185	2.32527	2.68991	2.87846
ZNF324B	4.38084	3.29295	4.6318	4.63163
RP11-49I4.3	1.25164	0.755715	0.534081	0.484748
MIR302B	13.3993	14.3162	11.7957	12.6472
RP11-438N16.1	21.5592	12.9873	15.3682	17.7059

CTD-2203K17.1	0.719044	1.12959	1.01305	1.0817
RP4-539M6.19	5.9372	6.25883	6.5954	6.2883
RP11-440L14.1	9.97865	12.6604	11.8486	12.9427
RP11-703G6.1	2.58834	3.05711	2.56852	2.54152
AP000295.9	0.244895	1.07624	0.205378	0.0452164
NOP14-AS1	9.39139	11.0615	11.9633	9.98076
RP11-619J20.1	1.9466	0	1.65932	2.46719
RP11-423H2.3	1.60424	1.25549	2.28067	1.89346
SRD5A3-AS1	0.683796	0.965615	0.938004	1.11473
ZNF564	1.0945	1.43619	1.03691	1.09106
RP11-259O2.3	3.88789	3.61316	3.8685	3.29125
RP11-673E1.3	1.40389	0.793835	1.02431	1.13287
SCARNA22	10.2851	14.1491	23.8796	18.3035
EAF1-AS1	26.4256	28.8049	27.7937	33.0023
VCAN-AS1	19.2268	17.7378	15.3394	18.5986
AC011330.5	2.16813	2.75549	2.41661	2.37826
EEF1A1P19	8.63567	186.974	157.495	189.11
PVT1	119.889	119.236	115.166	119.073
RP11-63E5.6	3.33225	2.77229	3.57716	3.2038
CTD-2541M15.1	2.8376	2.31035	2.51184	2.9114
CTD-2589H19.4	6.71924	6.94649	7.07922	7.47617
PDCD6	121.796	116.172	126.294	122.614
RAC1P2	1.39508	1.1117	0.878367	1.09629
RP11-80H5.5	2.97302	2.33483	4.00225	2.84966
RP11-256P1.1	33.6023	22.9467	24.3638	21.5487
TMEM158	11.5477	7.62385	8.7741	8.84769
RP11-359P5.1	1.3049	1.36987	1.25023	1.71591
C15orf38-AP3S2	8.5071	8.73459	9.03809	8.96505
YJEFN3	3.25662	3.79011	4.15108	2.48233

CTD-2116N20.1	1.33874	0.580015	0.506312	0.443593
RP11-359B12.2	1.65422	1.72156	1.81778	1.97672
RP4-622L5.2	3.34176	3.2996	2.33921	1.87956
ARPC4-TTLL3	5.69093	4.44866	3.96399	5.36407
EEF1A1P13	6.78302	15.6475	11.696	12.1328
RP11-1079K10.4	275.039	240.68	247.011	239.307
CTC-338M12.5	1.84573	1.77929	1.24363	2.18207
PKD1P6	14.2044	13.8403	14.8157	12.87
TAP2	1.07332	0.431931	0.695916	0.52677
PSMC1P5	32.4808	20.4218	20.5792	23.9422
SMIM20	31.5444	34.0319	32.0915	33.832
CTA-963H5.5	0.832668	1.06728	1.22327	1.04069
RP11-478C6.4	2.34159	4.23057	0	4.41324
RP11-23P13.4	5.07538	5.76642	5.6553	5.90436
AACSP1	1.62292	1.74067	1.62657	1.27956
HOXC-AS1	4.04168	3.62048	2.36533	3.10167
LRRC37BP1	2.21326	2.35	2.96431	2.54857
CHCHD10	426.147	381.675	381.88	360.675
CDK3	2.6189	3.19253	2.94473	2.85352
GPR162	0.956211	0.929964	0.946928	1.06048
KRT8P33	0.484711	1.0705	0.931179	0.827518
RP11-47I22.2	0.913245	0.927028	1.06032	0.955817
RPL38P4	4.97671	2.69433	4.23266	3.83395
ATP6V1E2	8.20457	8.6656	8.24415	8.94064
GLI4	17.9537	22.7886	22.2192	19.2792
RP4-669L17.8	1.86609	2.83578	2.82651	2.30342
IQCJ-SCHIP1	9.21291	8.72697	9.3459	9.16967
CTC-564N23.2	8.18742	11.7753	9.05309	9.14681
LINC00491	1.21479	1.57962	1.66787	1.34477

RP11-1149O23.4	2.31773	3.01399	3.39659	3.42529
SEPP1	1.03891	1.83185	1.22788	1.67504
RP11-230G5.2	2.22785	3.61405	1.86253	2.09925
ALG1L12P	0.514925	1.0223	0.681843	0.688513
ZBED3-AS1	1.14738	2.07828	1.6467	1.67322
RP11-253E3.3	4.82522	3.43915	3.5185	3.91095
CTC-338M12.6	1.20031	1.79134	1.6674	1.36462
RP11-301H24.4	7.48575	6.60748	6.7973	7.37714
RP11-297P16.4	37.0739	24.3556	27.0834	30.3178
GLUD1P3	0.542383	1.97118	0.415596	0.417613
RP11-752G15.6	4.84691	4.8632	4.12012	3.86829
RP11-392E22.5	4.65105	4.74896	5.56688	3.86667
RP11-1379J22.5	0.689211	1.14446	0.839882	1.03826
RP11-152P17.2	17.0369	18.1591	18.1413	18.5088
GAPDHP62	1.27912	1.16911	0.824994	0.810582
THAP9-AS1	26.8965	30.4088	25.9785	32.1384
RP11-138J23.1	0.891796	1.15071	1.07515	1.27247
RP11-168A11.4	1.34958	1.22275	1.1519	1.07517
CTD-2218G20.1	3.82744	0	0	1.20132
RP11-115D19.1	1.13136	2.11197	1.0997	1.29754
RP11-37B2.1	1.57475	1.67404	1.67431	1.46165
RP11-53O19.1	1.89509	1.59457	1.58088	1.62721
RP11-849H4.4	14.172	14.2676	13.8287	12.884
RP11-384K6.4	1.56737	0	0	0
CU459201.1	1.27464	1.19299	1.36069	1.41292
ZNF674	1.49551	2.21129	1.89893	1.65449
RP11-54F2.1	3.39488	3.26004	3.30628	3.90755
TMED7-TICAM2	5.19788	4.40449	3.42755	3.5715
AC004069.2	10.5684	10.5426	9.85431	10.5803

WDFY3-AS1	1.05915	0.59122	0.639911	0.651246
RP1-5O6.6	1.1674	1.35278	1.46439	1.07631
CTC-436P18.1	9.08811	6.10212	6.77965	7.02164
RP11-380P13.2	15.6145	16.4924	23.4688	24.8822
SHANK3	6.25762	5.47185	6.49573	5.93999
MSANTD3-TMEFF1	2.51017	1.82502	1.21452	1.86708
AP000350.10	7.94549	7.31163	9.35824	7.98999
CTD-2091N23.1	1.17761	0.981777	1.00835	0.990803
RPL32P3	1.83098	2.25712	2.15131	1.88826
AC068134.10	1.50416	1.16972	1.14117	1.67752
FOXD1	4.41095	4.1164	2.79963	3.84303
PPIAP11	1.32702	1.8885	0.400263	0.639766
APITD1-CORT	1.99402	1.78403	1.97397	2.20813
MALAT1	8.37976	9.76164	8.45734	7.18943
RP11-539L10.3	2.06469	2.48955	2.27909	1.6957
ABCA11P	2.98867	2.77857	1.96491	2.49263
RP11-164P12.4	3.15003	2.01816	2.26584	3.19433
FAM86EP	6.3382	6.36692	6.507	8.44084
CTC-458I2.2	2.40603	2.33207	2.74677	2.62158
RNA5SP419	0	1.64292	0	0
SCARNA8	2.90258	0	0	1.80013
RNU6-322P	0	0	3.75036	0
RN7SKP215	1.19396	0	0	0
SCARNA21	0	0	1.05707	0
SCARNA23	0	0	1.77427	0
SNORA70	11.1995	11.4085	16.21	23.0097
SCARNA11	0	0	1.28174	1.86983
AC104169.1	0	0	0	2.1993
RNA5SP216	0	0	3.09683	0

SCARNA17	5.51001	4.82876	7.94393	4.96157
RNU2-27P	21.8423	9.69325	6.90945	5.08587
AC092574.2	0	0	0	7.47744
SNORA9	0	0	1.46086	0
SNORA74	5.15546	6.10839	2.16916	1.58468
SCARNA24	0	0	0	1.88623
RNU1-103P	0	1.83937	2.58233	0
RNU5A-4P	0	0	3.50588	0
RNU6-885P	0	0	0	4.05527
Y_RNA	0.004303	2.76968	0	0
SCARNA13	1.96394	3.13555	3.9546	1.76375
SNORA73	36.6958	22.9963	16.5943	17.6677
RNU6-914P	6.40241	0	0	2.53863
SCARNA20	3.27379	0	0	0
RNU6-826P	0	0	0	5.15371
RNA5SP268	0	1.79698	0	0
RNA5SP392	0.475451	5.31704	4.73361	0
RNU6-850P	0	0.656968	1.41687	0.816269
SCARNA24	0	0	0	2.72267
RNU6-388P	8.25848	0	0	0
SCARNA4	0	2.11323	0	0
SCARNA21	2.56276	0	0	0
Z98044.1	0	0	1.36849	0
SCARNA3	0	1.85855	0	0
Y_RNA	7.17887	4.19232	2.98913	4.37453
SNORA31	10.1441	4.53963	3.22422	8.02174
RP1-117B12.4	80.1001	65.7318	74.1007	79.2091
RP11-946L20.4	1.02422	1.39249	1.15648	1.06076
RP11-158K1.3	1.64787	1.8464	1.34297	1.71045

KIAA0196-AS1	2.6325	2.8678	2.96478	3.72785
RP11-360L9.7	21.2109	18.2899	15.8423	18.4416
HOXA-AS4	8.74157	6.48314	6.52007	7.01793
RP11-582J16.5	15.4898	13.9267	14.4015	14.3587
GUSBP3	2.79341	2.80626	2.69502	2.61293
RP11-809O17.1	0.587736	1.03007	1.13808	0.954355
CTC-534A2.2	2.13701	1.80798	1.91716	2.2558
CCDC71L	3.22764	4.45329	3.98254	4.23734
HOXA10	30.8153	18.3399	21.5664	24.3573
TMEM200B	7.61457	7.41024	7.27949	7.64633
C1orf210	2.5738	2.41052	3.16231	3.6778
KB-1507C5.2	1.63226	2.6182	2.23865	2.33415
TUG1	16.3084	16.1463	12.9815	14.897
RP11-589F5.4	1.12122	1.64121	1.80902	1.61262
TRNP1	29.9798	29.9282	30.4273	30.3111
CTC-436K13.3	1.83362	2.50487	1.68081	2.43685
RP11-10A14.4	1.44332	1.31785	1.69382	1.39506
CTB-79E8.2	3.77681	4.7271	3.46486	3.67185
CTD-2501M5.1	1.02129	0.779561	0.653791	1.0449
FAM86HP	0.487672	1.41579	1.43017	1.08292
OSGEPL1-AS1	1.26583	0.925547	1.0654	0.760345
RP11-394O4.4	3.95743	2.96547	2.65175	2.30874
AC144568.4	0	1.62351	1.15898	0
EIF5AL1	4.76008	4.66539	4.63719	6.73701
CTD-2544N14.3	1.51071	2.46714	2.00825	2.83111
SOD1P3	0.721349	1.5601	1.57757	1.2189
KB-1732A1.1	1.45594	1.99095	1.67318	1.89806
RP11-59O2.1	3.36328	5.13242	4.28194	2.65969
CTB-79E8.3	2.49644	0.665089	0.259758	0.836357

RP11-1081K18.1	0.065577	1.27015	0	0
ALG11	3.39519	3.23234	2.80163	3.09713
RP13-582O9.5	36.1768	36.4956	35.7564	35.1503
ATXN7L3B	22.5196	22.1541	17.411	18.6084
RP11-10N23.4	9.73556	10.1923	8.90083	10.0542
PRKDC	109.78	112.095	109.541	99.7082
GS1-251I9.4	9.55337	9.91665	9.7326	10.7467
AC025442.3	3.23132	2.8241	3.58744	3.79058
UTP14C	3.83526	3.77894	3.43586	3.6299
CTD-2292P10.2	7.64063	10.0895	8.50327	8.18505
RP11-1415C14.3	2.39629	2.20128	2.55106	2.66385
PCDHGA10	1.34218	1.82127	1.84783	1.53075
RP11-10N23.5	1.06505	2.18673	2.18678	2.05439
SLC2A3P1	3.03398	2.03678	3.29918	1.74666
RP11-16P20.3	2.26211	2.27432	2.13216	2.41432
RP11-382A18.2	0.812214	0.860469	1.20742	0.643204
RP11-1149O23.2	2.73397	2.95961	2.22097	2.69718
RP11-410L14.2	4.2867	3.85733	3.69586	4.12617
CLDN23	1.74601	1.85816	2.03815	1.74589
RP11-363L24.3	2.274	0.955611	1.93269	1.41006
RP11-4K16.2	15.1492	13.7649	12.5015	13.0737
ALG1L13P	0.755233	1.93144	1.52249	1.34344
CTD-2336O2.1	11.4597	9.42833	9.80954	10.1065
RP11-103H7.5	5.80107	6.27518	6.29598	6.22685
RP11-681L8.1	0.551562	0.939309	1.02546	0.963294
RP3-388N13.3	1.18611	1.1251	2.32767	1.61941
CTD-2530N21.4	1.3105	0.789246	1.24658	0.653027
LYN	20.8482	17.9557	19.1448	21.3963
PINX1	20.4971	16.1523	17.0479	18.7388

AC005740.5	1.41484	1.08208	2.04324	1.73237
7SK	0.397091	1.0498	0.985426	1.08174
RP11-255B23.4	1.80716	1.86649	1.47521	1.70258
TYW1B	1.96475	1.4266	1.75721	1.92415
RP11-585F1.8	6.52654	9.00049	7.15966	4.71065
RP11-598P20.3	16.3292	8.16615	12.8292	14.7159
RP11-582J16.3	7.99541	4.97299	5.4182	5.6098
RP11-398H6.1	1.76953	2.10757	2.45296	1.94631
KRT8P3	5.37246	4.13201	4.34457	3.67111
KB-1608C10.2	7.63096	9.6623	10.3237	10.6474
RP11-909N17.3	0.88431	1.10796	0.913406	0.753423
RP11-10A14.3	0.694941	0.801168	0.833297	1.17661
RP11-195E2.4	3.17325	2.27794	2.4365	2.15229
RP11-34P1.2	1.00338	1.55479	0.855683	1.01201
RHPN1-AS1	0.494143	1.65353	1.53371	1.10745
LRRC24	13.8975	16.7747	19.4574	15.1327
MSH5-SAPCD1	1.30986	1.00066	1.41335	1.27888
CHKB-CPT1B	2.82968	2.77364	3.32411	2.86191
RP11-864G5.3	22.3629	19.931	18.0163	22.0491
RP11-110I1.11	0.81007	1.34378	1.20417	0.805907
CTD-2562J17.7	3.45366	3.27436	3.72474	4.11344
RP11-677I18.3	4.09013	3.18361	3.11809	3.74519
RP11-867G23.4	140.58	118.737	129.089	133.353
RP11-755F10.3	1.04664	1.25125	0.779825	0.642922
RP11-849H4.2	1.10683	1.49183	1.56309	1.52004
AP5B1	5.62624	5.81059	6.77975	6.4167
RP11-522I20.3	6.88061	4.44872	5.01913	5.26058
RP11-23F23.2	1.05869	1.17292	0.948653	0.933101
AP003068.9	36.7218	39.6982	39.9865	34.7252

CHMP4A	29.0763	33.8621	31.3689	30.5371
AP001816.1	11.0886	16.2105	15.678	13.3095
RP11-304M2.5	38.7967	28.6307	31.9613	32.3808
RP11-60I3.4	1.31545	0.496442	1.20568	1.80309
CTD-2517M22.16	5.66767	6.48522	5.87747	6.03233
CSNK2A3	1.00087	0.884973	0.861581	1.0794
AP003068.23	2.48633	1.59526	2.5351	3.16865
RP11-395G23.3	8.65703	6.98529	7.32959	7.61192
TMED10P1	1.00967	1.04778	1.21673	1.05744
RP11-231C14.3	8.71663	8.49402	9.78163	12.4066
WAC-AS1	1.7642	2.27881	2.22732	2.4012
RP11-872D17.4	3.94508	3.11408	3.36481	4.6235
RP11-152H18.3	2.034	1.98611	1.64408	2.02646
RP11-598P20.5	0.702358	5.422	1.40016	6.289
RP11-265D17.2	9.85241	10.2912	9.67772	10.3899
PKD1P5	15.7686	17.0226	17.6136	15.5671
RP11-660L16.2	13.8119	8.69463	10.205	11.5242
FPGT	6.72103	5.64751	5.03111	5.53257
GS1-393G12.12	14.0144	12.853	12.5073	11.3096
TM9SF1	7.01634	7.12521	8.01753	8.91549
RP11-50B3.4	0.173708	1.34931	0.663302	0.658776
RP11-1415C14.4	2.38083	2.16588	2.5233	2.24338
RP11-565P22.6	2.0908	2.02783	2.19594	1.71866
RP11-805J14.5	34.3963	32.4663	35.3867	35.6433
MEX3A	2.68602	2.96217	2.46165	2.40742
RP11-334E6.3	2.05076	0.993136	1.35999	1.55335
RP11-661A12.7	185.758	174.229	172.148	166.593
RP11-867G23.12	5.38389	7.37712	7.17869	6.0141
RP11-867G23.2	9.31901	7.20374	7.46768	8.85099

EEF1G	1450.94	1519.23	1432.25	1481.99
CKLF-CMTM1	0.00116	0.96772	1.04102	1.92309
SYS1-DBNDD2	1.8675	0.646266	1.23553	1.03575
RP11-535A19.1	0.980811	0.908508	1.01427	0.932323
RP11-496I9.1	1.24646	1.07186	1.43868	1.44481
SLC22A18AS	1.59992	2.32071	1.98342	2.47409
RNF185-AS1	27.0326	21.7648	26.7163	27.3711
AP001372.2	1.18941	1.41897	1.45017	1.48385
RP11-109L13.1	2.59244	2.21894	2.42834	2.49167
NPIPA2	2.5182	2.76345	2.51601	2.38172
CTD-2523D13.2	0.742991	0.92159	0.950162	1.05073
MPV17L2	32.9	32.6111	31.5408	30.684
TMEM9B-AS1	2.43317	1.61952	2.34692	2.58018
RP11-770J1.5	9.84676	5.67023	5.34339	5.97673
RP11-466P24.2	0.424634	9.5905	0.287399	0.556955
RP11-513D5.2	0.594231	0.941424	1.16334	1.03205
MEF2BNB	19.0291	16.7034	18.0846	17.4947
ANO1-AS1	14.5845	16.1142	14.0984	17.5295
RP11-712L6.7	3.14402	2.22779	2.25631	2.80574
RP11-484D2.2	4.37284	3.90996	3.74066	3.88989
RP11-110I1.5	3.87513	5.951	5.09614	5.37663
CTD-2562J17.9	2.45866	2.92263	5.46706	2.32027
ALG1L9P	1.98746	1.88586	2.20147	2.40122
RP11-872D17.8	1.36401	0.795992	1.17408	1.06711
DPP3	38.7469	42.049	45.2129	40.3048
ANKHD1-EIF4EBP3	4.89638	4.92482	4.68013	4.49779
BRK1	118.138	118.31	123.444	134.661
RP11-802E16.3	3.71422	1.8925	2.6465	1.72666
RP11-712L6.5	4.97491	4.00554	3.33161	4.42374

PIGY	208.207	233.712	212.406	216.024
RP11-820L6.1	15.5275	19.9868	7.91762	14.0401
RP11-481A20.11	1.00857	1.16603	1.27228	1.46392
KIAA0754	2.85956	3.07763	3.33917	3.38256
AP006621.8	13.3625	12.0904	13.3169	10.918
CHMP1B	8.61903	11.6584	9.66898	11.1208
RP11-110I1.12	0.615357	0.825645	1.21809	0.886905
CTD-2531D15.5	13.4047	12.5202	12.0698	12.0402
RP11-111M22.3	13.0818	13.1691	13.0118	13.7401
AP006621.6	0.651625	0.90787	1.27774	1.07082
RPP14	14.0785	13.0854	13.9928	15.7973
RP5-1047A19.4	1.22417	1.60067	1.62111	1.64883
CTD-2517M22.14	7.99603	7.80033	9.29232	7.86362
RP11-106J23.2	4.29427	3.57116	4.1351	3.97464
SNHG9	139.82	99.8561	78.6708	96.0652
RP11-350N15.4	2.33484	2.80212	2.46737	2.20376
C14orf169	1.94441	2.02174	2.52825	3.18543
RP11-166D19.1	15.0954	14.2441	12.9266	13.8888
AP006621.5	4.66004	5.98593	4.54801	4.84291
RP11-624G17.3	16.6896	18.4436	20.0892	16.5597
EID1	67.6764	68.9636	68.8937	77.8757
RP11-755F10.1	0.857754	1.53606	0.814578	0.879621
NDUFB8	1.87804	1.82502	1.99059	1.68206
RP11-770G2.5	4.91461	4.09324	3.84606	4.79797
MSH5-SAPCD1	1.26465	0.93342	0.901713	1.06842
RP11-196G11.1	1.73124	1.83108	1.56782	1.04689
RP11-890B15.3	0.837383	0.939874	0.909318	1.0177
RP11-144G7.2	1.7352	1.18023	1.2526	1.42454
RP11-867G23.8	1.31356	1.45674	1.50493	1.51938

RP11-618K13.2	7.62385	8.74161	10.0606	8.45171
MIR3654	1.8388	1.85275	2.75217	2.28747
AC005363.9	5.57594	4.09633	4.54864	3.78812
CTD-3074O7.5	2.63978	3.6481	3.21823	3.25498
POLR2M	21.8713	22.1463	20.9204	22.1482
CTA-797E19.2	2.36828	2.0581	1.3674	2.13854
FDXACB1	3.10229	2.79719	3.03295	2.97615
AL590762.1	143.98	176.402	148.23	183.419
Z82188.1	5.8499	3.80077	1.95547	5.05167
RP11-564D11.3	0.881549	1.29331	1.31081	1.26838
RP11-196H14.3	5.19051	4.67783	4.73628	5.89999
MTRNR2L9	1.48657	1.2675	1.80101	1.52546
RP11-234B24.6	13.397	11.8244	12.3128	13.0161
NKG2-E	1.4695	2.00666	1.39466	1.5585
FAM222A-AS1	5.58649	4.55022	4.20147	5.27264
RP11-867G2.6	0.934207	1.31913	0.993878	1.46544
RP11-227B21.2	1.94458	1.7589	1.92923	2.01202
SNHG1	268.619	248.51	212.173	198.88
AC005618.1	2.48347	1.09708	1.48392	1.27719
AGAP2-AS1	16.1887	15.7931	18.9011	16.3234
RP11-152F13.3	3.83109	3.45961	3.46261	3.06311
RP1-34M23.5	0.75088	0.767951	1.30838	0.821594
AL139385.1	18.164	18.1916	18.1967	17.7012
RP11-338K17.8	4.08507	4.70612	4.23891	4.63756
PXN-AS1	8.7225	7.72778	7.82807	7.47691
LINC00346	1.03835	1.90566	1.89729	1.43886
RP11-685N10.1	2.32671	2.20856	1.83892	2.03084
U47924.19	1.08753	1.5603	1.2547	0.887369
AP001062.1	1.51344	1.7162	1.91043	2.12899

AC005000.1	30.4293	31.7648	27.1867	30.3604
RP11-867G2.8	3.63023	4.0572	4.7951	4.02905
AP003419.16	16.165	18.3617	21.9431	23.4675
RP11-804A23.2	0.970174	1.14885	1.17114	0.950021
AC034102.1	4.13939	4.20069	3.67556	3.9415
RP11-575F12.2	1.16015	1.04841	1.30771	1.50689
AC093734.1	14.961	12.2739	15.5378	14.0094
ARAP1-AS1	16.3559	21.266	19.3338	18.5524
RP11-728G15.1	2.41419	2.061	1.78045	2.13114
RP11-27M24.1	13.9926	10.9967	11.1502	11.6574
RP11-707G14.1	28.4364	21.6395	25.3361	23.4357
APOPT1	26.3169	23.8861	19.8543	23.7354
TRAPPC2P1	5.42411	4.75004	4.15396	4.63857
DYX1C1	2.09557	2.46024	2.65576	2.03424
C21orf119	8.31673	9.07649	9.31119	8.92784
hsa-mir-8072	1.12593	1.03958	1.15652	1.36254
RP11-783K16.14	3.13477	2.79808	2.83697	2.84788
RP11-809N8.5	0	3.72419	0	0
AC004889.1	2.36154	2.48533	2.48381	2.63478
RP11-612B6.2	1.25887	0.760745	0.539869	0.81702
AC005255.3	2.93153	0.00023	0.302529	5.10E-05
AC010620.1	2.62218	4.95751	9.35684	7.00788
RP11-221N13.3	2.84519	3.06735	3.01038	2.95154
HMBS	52.9712	50.119	50.2239	51.7399
ZNF225	1.10227	0.993918	0.814245	1.00114
RPL41P2	11.0288	8.41839	3.92326	3.52238
RP11-173P15.3	70.4409	47.994	50.5528	62.4824
RP11-667M19.10	0	0	0	1.32345
DND1	8.88947	7.56966	8.36434	7.82206

RP11-856F16.2	8.4273	5.68151	6.57229	8.09394
RP11-73M18.2	2.37807	2.87268	4.27993	3.79397
RP11-860B13.3	20.0447	21.086	20.7815	18.6185
AP003419.11	9.466	10.3693	12.007	10.2888
POLG2	12.5176	11.5806	11.2682	11.832
RP11-785H5.1	9.23301	9.58998	5.55381	2.28015
AL139819.1	47.6646	31.5397	29.3758	26.2395
AC156455.1	10.4718	8.61194	8.58493	8.99383
RP11-173P15.5	32.1029	30.6057	33.0127	30.6331
RP13-977J11.2	1.08822	1.35356	1.44347	1.20545
AC091171.1	8.7861	10.3431	11.7177	10.5015
RP11-286N22.8	39.3959	26.7412	37.3067	42.9405
AC020663.1	7.1314	6.44319	5.3884	7.27942
ZBTB11-AS1	6.92632	7.09602	7.89656	6.77085
PSMA2	8.093	7.10456	7.07175	7.38452
RP11-424C20.2	5.47986	4.48319	5.21829	5.14292
RP11-611O2.3	23.1792	17.2504	15.5166	17.4509
KLRAP1	1.10526	1.23967	1.13832	0.840308
RP11-599J14.2	0.506401	1.06615	0.704882	0.447164
ZNF350	1.10331	1.19503	0.924321	1.17406
AP001029.1	3.29425	3.00504	3.57275	2.81394
RP11-697H9.2	1.50616	0.842695	0.634003	1.18486
RP13-977J11.5	1.48939	1.19081	0.695436	0.614871
RP11-804A23.4	19.8757	15.8689	15.3438	15.3779
RP11-467L13.4	172.906	171.251	171.376	88.3322
RP11-512M8.5	1.89776	0.639156	1.67232	0.431093
NOL12	26.0889	21.4529	23.8514	22.1534
AL021977.1	4.71274	4.65577	3.2479	4.80749
AC090571.1	0	0	1.12717	0

RP11-221N13.4	1.40692	1.41676	1.1955	1.53393
RP11-809N8.2	8.20552	6.42402	7.23077	7.29087
AC067852.1	10.634	10.8006	18.5057	11.5097
RP11-783K16.5	5.99133	5.92026	5.69753	6.30991
RP11-87C12.2	9.47334	7.58388	5.80772	5.51905
AC055876.1	12.1734	14.0926	17.8374	14.6908
RP11-613M10.8	3.34278	4.35144	3.53643	3.62265
RP11-273B20.1	1.28087	1.5617	1.69862	1.6249
LIMS3	0	1.86269	1.86369	0
KHDC1L	5.94708	4.60415	4.67816	5.9401
RP13-820C6.2	11.2424	8.31275	10.5899	10.0685
AC040173.1	4.22114	3.83273	4.1648	4.91555
C15ORF37	0.94242	1.03877	1.18555	1.08995
AL391357.1	13.0859	14.9768	19.6039	17.511
AL021707.2	0.689265	2.31869	1.2015	1.57532
AC011933.1	69.3536	57.0804	52.8825	48.6744
RPL29P33	1.99436	2.09814	1.1021	1.36463
U47924.27	5.57602	6.49778	6.54041	5.19573
RP11-783K16.13	4.37784	4.37111	4.10865	4.81077
KIAA1147	6.53346	6.69428	7.1385	7.6803
LSM14A	54.1126	49.8704	49.7516	49.0555
RRN3P3	4.69778	4.76109	4.97601	4.54977
BX927168.1	2.57297	2.35707	3.34352	3.9096
RP11-320M2.1	0.740097	1.22929	1.29444	0.936835
TMPO-AS1	1.60692	1.37648	1.25198	1.39073
RP11-611O2.5	29.2556	25.4815	20.9121	21.7542
GATC	20.5591	14.4384	14.8001	16.1806
RP11-96H19.1	5.90376	12.9297	10.5226	9.9853
ZNF271	5.90191	5.75288	5.26889	6.1088

RP11-521B24.5	20.397	24.5821	21.7193	20.1449
RP11-298I3.1	1.60086	1.02458	1.01309	1.43748
RP11-545P7.4	9.01483	10.2612	10.1542	10.1014
RP11-977G19.11	0.787338	1.13254	1.4787	1.2127
RP11-603J24.18	1.27513	0	0	0
ZBED6	2.05705	3.04422	2.85359	2.86875
RP11-983P16.4	49.5533	44.548	43.3156	49.3686
PLEKHM1	1.49043	1.47887	1.72608	1.63985
CRIP1	6.73673	8.90884	9.60976	9.54604
RP11-571M6.7	33.6945	38.0677	38.9822	38.7112
RP11-780K2.1	1.34188	0.832807	0.944413	0.763206
FNTB	15.4989	10.3576	12.5487	15.62
MIR3179-2	1.25941	0.811185	1.13315	0.923903
AC217772.1	2.9344	2.98214	3.24455	3.46403
RP11-56G10.2	25.3522	21.5238	19.2752	18.2447
RP11-762I7.5	6.24392	5.12446	6.0236	6.21111
RP11-603J24.9	43.3663	56.9322	38.6574	56.7784
YTHDC1	4.00599	3.29722	3.61075	4.14648
RP1-197B17.3	0.930768	1.60201	1.23622	1.20232
AC217779.2	1.6984	1.53681	1.65147	1.74735
RP11-290L1.3	192.822	200.162	218.428	200.965
RP11-175P13.3	0.908923	1.07382	1.16274	0.90773
RP11-585P4.5	1.98627	1.40252	1.19647	1.70229
RP11-571M6.8	1.15819	1.03535	1.34083	1.36916
RP11-203J24.9	2.69152	3.54953	3.66957	3.04056
MIR3179-3	1.16046	1.08236	0.932529	0.85501
RPL36A-HNRNPH2	12.4422	20.5283	26.7801	33.5111
RP11-793H13.3	1.76415	2.21293	2.09614	1.95868
RP11-603J24.17	225.086	156.385	151.01	165.636

RP11-44N21.1	1.74579	2.88028	2.49389	2.51691
RP11-84G21.1	0.853303	0.868593	0.664539	1.09896
RP3-473L9.4	2.22848	2.74432	1.45619	2.33924
RP11-680A11.5	1.03194	1.31361	1.11973	1.07062
RP11-349A22.5	7.1185	8.32483	7.44518	7.90559
RP11-44N21.4	0	1.46887	0.888487	0.527873
RP11-804F13.2	1.17507	7.60095	3.12636	2.2626
RP11-579D7.2	6.40356	6.16417	6.26993	6.69394
RP11-1100L3.7	58.7652	48.3776	52.3592	50.0792
RP3-416H24.1	3.76034	4.16353	3.47537	3.59679
RP11-341G23.4	1.332	0.996925	0.528222	0.961152
RP11-620J15.3	3.91562	4.87253	3.38281	3.62581
LBX2-AS1	0.935027	1.2128	1.57852	1.25433
PRR24	12.1456	12.0057	13.8057	12.2117
RP11-256L6.2	1.61709	2.03874	1.90016	1.93193
AC225613.4	30.9538	26.483	25.5231	28.5119
CNPY2	143.166	127.304	123.624	125.375
RP11-818F20.5	3.64848	2.84134	1.93449	1.82543
RP11-977G19.12	1.29534	0.595882	0.754716	0.61903
RP11-350F4.2	1.75951	1.68297	1.74784	1.99485
RP11-162P23.2	1.00519	1.28454	1.96867	0.369284
ARL17	2.00835	2.0621	2.27504	2.3773
RP11-611E13.2	0.491805	1.13068	0.983245	0.841179
BX511262.3	1.16685	1.02368	1.26506	1.26656
RP11-454K7.1	1.31864	1.84602	2.62342	2.3533
RP11-386G11.5	4.47483	5.10187	4.75845	4.08538
RP11-571M6.15	5.77387	0.679938	2.49818	3.03983
CUX1	15.6285	16.1979	18.1858	17.2345
TEN1	25.5452	21.8489	19.1989	24.7092

AC003070.1	5.50774	6.04296	7.14311	6.67969
RP11-386G11.10	75.7951	62.451	60.2106	68.0281
RP11-493P1.2	11.0414	11.6933	10.5049	11.7466
RP11-530C5.1	17.7281	13.0936	14.6133	12.2668
RP11-644F5.11	2.68588	2.58355	2.75578	2.59835
RP11-1079J22.1	0	0	1.89435	0.954567
RP11-114H23.1	2.35625	3.87463	4.05094	3.78324
RP11-114H23.2	2.31568	2.97511	2.29142	2.65165
RP4-816N1.7	298.518	232.791	255.626	263.614
RP11-864J10.4	1.03537	0.694931	0.733857	0.720441
AC225613.7	3.55272	3.45285	4.43966	4.32138
RP11-44N21.2	4.74111	9.73107	6.76579	5.2123
RP11-588H23.3	3.05896	3.35312	3.82723	3.60757
RP11-394J1.2	8.10913	8.51037	7.81953	7.91849
SLC7A5P2	2.57526	1.95791	2.96397	1.69921
RP11-977G19.5	6.09032	4.59267	5.45902	6.2451
RP11-136F16.1	0.824218	0.926579	1.09183	1.43208
AL805934.2	1.16703	1.02392	1.26532	1.26677
RP11-161H23.5	972.648	795.719	762.245	861.754
RP11-114H23.3	5.13273	8.63419	6.2827	6.34574
CR759761.2	1.16703	1.02392	1.26532	1.26677
RP11-977G19.14	1.22416	0.73416	1.07465	0.98301
RP11-386G11.3	9.94082	10.919	7.68933	14.4635
POLR2KP1	1.14719	0	0	0.733471
CHURC1	62.9464	62.2743	55.4893	65.8198
RP11-658F2.8	5.30684	7.33634	6.32107	4.37996
RP11-488C13.5	8.91727	8.04456	9.57199	10.0483
RP11-644F5.10	1.67411	3.01625	1.80622	1.75893
C17orf49	17.4469	12.8249	8.64853	9.11858

RP11-603J24.5	5.16166	5.05002	4.58843	5.20604
RP4-816N1.6	4.76329	4.04272	4.05994	3.31128
CR753842.2	1.25749	1.1359	1.36519	1.36164
RP11-1105G2.3	1.06262	0.855735	1.12692	1.22016
RTEL1	20.1153	20.114	21.6048	18.3204
ARL17A	1.74602	1.93217	1.62443	1.65277
RP11-649E7.5	20.3974	13.5841	16.623	18.7881
AC068831.6	1.03772	0.887367	0.749643	0.934482
PDF	48.4126	51.2591	52.5675	50.516
LINC00641	5.40358	3.14083	3.2105	3.65092
RP11-903H12.3	1.00192	0.611621	0.72756	0.63372
RP11-1012A1.4	2.21301	0.729782	0.218921	1.28537
RP11-84C10.4	2.90702	2.69193	2.67937	2.6065
RP11-192H23.4	24.588	18.1957	20.0189	17.0134
LINC00640	1.75013	1.30838	1.13397	1.27515
RN7SL1	340.762	388.274	332.478	167.679
DIO3OS	0.289322	0.283979	0.288973	1.50687
RP11-638I2.6	24.1903	20.2204	22.8508	20.8893
RP11-796G6.2	0.456217	1.08225	0.99755	0.910079
RP11-829H16.3	1.76065	1.65901	1.80456	1.74333
CSNK2B-LY6G5B-1181	1.03837	0.570901	0.900098	0.732691
RP11-973D8.4	35.8134	31.0098	30.8728	34.544
RP11-72M17.1	1.4842	1.27947	1.08515	1.08243
RHOQP1	1.7875	1.23489	1.3974	1.06801
CSNK2B-LY6G5B-1181	1.13927	0.721572	0.939839	0.870949
RP4-773N10.4	2.59244	2.51134	2.15704	2.28899
BCL2L2-PABPN1	12.4016	11.3561	11.2149	8.01808
RP11-718G2.3	33.5927	33.656	34.9434	41.0567
CTD-2302E22.3	1.56354	2.06116	0.567269	0.240995

RP11-950C14.3	15.8711	11.7569	12.5876	12.994
RP5-1021I20.4	0.816709	0.887909	1.28947	1.84573
ARHGAP5-AS1	0.847478	1.0978	0.965222	1.08127
RP11-193F5.4	3.31105	2.80891	2.78008	3.67319
RP4-693M11.3	1.86081	1.37452	1.9656	2.0062
IFRG15	27.405	15.3632	18.316	23.4615
RP11-638I2.8	4.95803	3.41231	4.36533	3.01971
CTC-260F20.3	147.751	120.854	107.545	139.471
RP11-998D10.7	7.19837	8.38873	10.9632	9.15143
PRC1-AS1	61.9267	51.0909	50.8573	52.0277
RP11-66N24.3	1.91261	2.36479	2.3601	2.12785
RP11-73E17.2	1.54669	1.3815	1.36421	1.62946
RP11-688G15.3	23.3947	20.323	20.6692	21.7425
RP11-841O20.2	12.4183	12.0564	9.75674	11.2876
KIAA0391	0.522349	0.813625	1.23227	1.02992
CTD-2302E22.2	20.1793	14.9893	14.5348	16.2064
RNASE4	1.6923	1.17573	1.70949	1.06834
AC005041.17	0.993265	1.5428	0.928114	0.768073
CTD-2555O16.2	1.07972	0.901473	0.748538	0.674514
MC1R	1.2376	2.14517	1.97414	1.53529
CTD-3035D6.2	0.410799	0.488603	1.10282	0.995508
CEP95	25.3698	24.1309	18.7043	18.0035
RP5-1021I20.5	6.49306	4.69622	4.05402	5.55137
RP11-193F5.1	4.58004	5.92329	5.90318	6.51828
HNRNPCP1	1.24517	0.824743	1.00003	0.666364
RP11-203M5.8	42.4901	33.9886	38.4119	41.6866
FOXN3-AS1	1.30975	1.87424	2.11486	1.99316
TUBB3	126.48	136.586	125.448	115.543
RP11-1017G21.4	21.3845	27.7448	28.535	23.9396

RP11-618G20.1	5.94366	3.86517	3.66978	4.3728
RP11-638I2.4	0.235702	0.964961	1.05397	0.841186
RP11-368P15.3	12.2756	4.69508	4.68827	7.62612
RPPH1	1.97064	1.62114	1.8097	0.82247
RP11-566K11.4	3.18873	3.85276	3.40858	3.83305
TVP23C-CDRT4	3.35451	3.28917	3.28293	3.09803
CTD-2062F14.3	1.60572	1.68345	1.84795	1.67908
BLOC1S5-TXNDC5	61.1877	65.1865	71.5718	65.0624
CTD-2058B24.2	5.42387	4.59232	5.7676	8.44367
RP11-589M4.1	49.4467	48.4951	48.6926	51.5695
AL157871.2	4.24474	2.25322	3.37297	3.34529
RP11-247L20.4	3.14197	2.21274	2.11379	2.07639
RP11-488C13.6	2.23442	3.423	3.43354	2.11349
RP11-134E15.2	1.11144	0.887179	0.538417	0.809715
CTD-2017C7.2	0.945001	1.18079	1.28665	1.23059
RP11-603B24.2	1.31996	1.17189	1.58937	1.15192
NDUFC2-KCTD14	9.07871	11.1445	11.4531	10.6733
RP11-298I3.5	14.3429	12.4751	11.394	9.06942
RP1-261D10.2	0.746129	0.642188	0.516487	1.06995
RP6-65G23.3	2.25708	2.60312	2.09695	2.25106
GNRHR2P1	7.96588	7.47461	6.8261	6.89268
AL163636.6	1.14941	1.544	0.776632	1.19948
RP11-56B16.4	17.505	15.6295	11.4944	14.3161
CTD-2008A1.1	36.799	39.1059	35.7081	41.2222
RP11-56B16.5	1.60855	1.14275	1.15999	1.39022
RP11-64K12.8	3.79537	2.366	2.32444	2.31166
CTD-2555C10.3	0.628946	1.38731	1.60455	1.19373
RP11-361D15.2	6.04419	6.04011	7.20807	6.27522
RP11-182J1.12	0.985106	0.630646	1.0902	0.701812

USP3-AS1	21.6031	15.1976	14.8699	17.005
RP11-50C13.1	0.801108	0.820812	0.923111	1.05176
RP11-617F23.1	2.71498	3.23892	3.39323	3.08897
ZHX1-C8ORF76	2.13903	1.42185	1.98987	2.00304
CTD-2116N17.1	1.01657	0.891184	1.21315	1.03385
RP13-608F4.5	2.54647	0.669943	2.95624	0.801475
RP11-152F13.7	3.61537	3.23382	3.38765	3.18446
LINC00984	9.38604	5.35393	6.79922	7.8252
RP11-761I4.3	1.71218	1.54446	1.24453	1.69194
RP11-109D20.2	32.7802	30.2415	30.9202	36.0951
RP11-316M1.12	107.603	94.6858	73.5686	81.9988
CTD-2647L4.4	1.65506	1.17359	1.23244	1.58335
RP11-64K12.4	2.17565	3.12247	4.59757	3.45103
RP11-815J21.2	1.41339	1.70491	1.53023	1.63564
RP11-346D14.1	1.11121	1.20092	1.25881	1.35168
TGIF2-C20orf24	0.640703	0.470725	0.68762	1.30354
ISCA1P4	0	1.3365	0	1.40163
RP11-158M2.3	1.48398	1.71251	1.91694	1.76682
RP11-253M7.1	4.36003	5.36794	3.50529	5.50696
UBE2Q2P3	1.61167	1.54986	1.19927	1.33682
THTPA	2.71111	3.70137	4.60273	4.51231
RP11-89K21.1	1.06947	1.70851	1.46881	0.883833
RP11-128A17.1	0.594473	0.792023	1.02929	1.25407
RP13-996F3.3	3.86957	3.50481	3.47438	3.06988
CTD-2008A1.2	25.5042	22.4376	22.7794	24.3611
RP11-26F2.1	3.19674	2.44536	2.39807	2.83759
MRPL46	42.2144	30.7636	36.1971	35.8651
RP11-244F12.3	2.48266	2.06837	2.27023	2.09424
RP11-685G9.2	0.733436	1.19946	1.04796	1.00849

RP11-365N19.2	8.81283	7.27957	8.4097	7.5843
RP11-540O11.4	2.20005	1.61836	1.66745	1.52136
RP11-66B24.4	50.2548	37.9495	49.4472	54.9019
RP11-516C1.1	7.8211	6.31673	6.61948	6.50171
RP11-156E6.1	7.4756	5.44203	5.03867	5.78423
RP11-244F12.2	5.488	4.35643	4.45514	5.7133
AC068831.15	13.2792	12.3954	13.1614	14.0474
RP11-485O10.2	0.407823	1.30194	0.830385	0.710055
IQCH-AS1	0.869495	1.264	1.18019	1.34767
RP11-120K9.2	1.30036	0.928694	0.958202	0.678031
CTD-3110H11.1	1.32737	1.37973	0.950045	0.680238
RP5-977B1.11	71.8404	70.1861	66.0813	64.1542
RP5-977B1.12	1.56251	1.7823	2.02071	1.80121
FKSG62	1.84221	1.91107	2.04039	2.0021
RP11-505E24.2	1.71879	1.63046	1.76318	1.96533
CASC7	18.0664	11.6312	13.6156	15.0837
RP11-299G20.3	0.791015	1.23524	0.903489	0.8782
RP5-991G20.1	1.3183	0.686648	1.342	1.16495
RP11-673C5.1	152.176	119.001	115.749	147.593
RP11-114H24.6	3.20078	2.66433	4.02245	3.89311
CTD-2256P15.2	1.10816	1.51259	1.20319	1.00473
SLC22A31	13.152	17.7892	19.6589	16.9421
CTD-2196E14.4	3.42574	2.65278	3.27805	2.98375
RP5-1142A6.5	6.09408	5.16212	8.61884	8.51059
RAB43P1	1.66795	1.47371	1.60143	1.68274
C17orf51	1.73151	1.39286	1.83447	1.9456
RP11-488L18.10	1.84376	1.74407	2.17311	2.39737
MANF	64.4267	54.7208	61.2213	64.9887
RP11-830F9.5	2.65664	2.59537	3.3894	2.25088

RP11-1100L3.8	20.8732	15.1932	16.6707	17.4152
SH2B2	4.67124	5.00198	6.17534	5.76145
KLHL11	1.80825	1.42038	1.47305	1.62606
RP11-392E22.8	5.9065	6.0522	5.80854	5.11029
RP5-991G20.4	1.94388	1.69639	2.1352	1.92238
RP11-304L19.1	6.65057	5.52442	4.84596	4.49463
CUX1	9.13199	9.71714	12.3316	11.5256
RP11-77H9.5	10.5431	10.1419	9.20924	8.67445
RP1-39G22.7	1.54018	1.50007	1.59503	1.79702
CTD-2012K14.3	30.7484	20.815	19.1949	19.8153
RP11-326A19.5	9.93853	11.0835	10.0816	11.0814
AC009133.15	126.068	112.741	123.421	114.632
CTD-2547G23.2	2.44341	2.32226	2.07944	2.85581
RBM15B	5.20936	5.53218	6.19779	6.31449
KAT2A	11.2749	10.4584	11.8164	11.8933
AC099668.5	0.47028	1.88048	1.86817	1.66067
AC009120.6	87.1821	73.3359	78.3694	78.4412
RP11-553L6.5	6.31686	7.70493	7.4782	7.53006
MRPL45	27.361	23.8828	23.9721	25.9203
RP11-549B18.1	0.610685	1.0144	0.83043	1.02187
RP11-305E6.4	1.01307	0.68955	0.770098	0.806835
TGFBR3L	17.3255	18.8373	20.2508	17.2948
AC027601.1	1.85106	2.10456	2.28335	2.02168
RP11-469M7.1	1.02359	0.705202	0.770672	0.922488
RP11-315D16.2	4.323	2.02857	1.78399	2.40336
RP11-505K9.1	3.13246	2.78564	1.96221	2.11502
RP11-49C24.1	9.12074	7.78101	7.83577	7.05392
RP11-490M8.1	1.13321	1.09737	1.18677	1.14025
HOXB7	51.9137	47.2357	49.8897	51.9797

LINC00657	51.8326	62.0679	71.9828	74.3628
STAM	6.05384	5.84393	6.22689	6.71359
LA16c-390E6.4	0.640984	0.857724	1.00526	0.573461
RP11-611L7.1	16.5389	14.8195	14.3656	15.1039
NSFP1	8.40871	7.94085	7.44097	8.48419
MIR4519	0.869026	1.16846	1.29332	1.30308
RP11-33B1.4	2.43386	3.02219	2.66616	2.86305
RP11-715J22.3	0.733577	0.442817	0.982588	1.05832
AC005606.15	1.65302	1.14074	1.06092	0.766753
RP11-529K1.4	4.46352	3.57917	3.57214	3.65089
CTD-2574D22.4	0.767668	1.16998	1.2288	1.10998
CHAMP1	5.7876	5.83524	6.52667	7.66787
RP5-1142A6.9	13.0878	13.4825	14.7873	12.8589
ULK4P2	0.446777	0.506492	1.3832	0.539824
MYOM2	0.835163	0.168006	1.48686	1.25542
CTD-2270L9.4	7.5714	7.1519	7.52274	7.69451
RP11-114H24.1	0.98263	1.50049	1.13579	0.622827
MYO19	23.2529	20.0365	21.749	22.718
SURF4	64.685	51.9579	56.815	57.1799
RP11-432I5.6	2.04242	1.32659	1.26631	1.68733
EXOSC4	59.2003	52.3983	58.1737	52.0316
EPHB6	1.28496	1.94741	1.66996	1.3904
RP11-347C12.10	1.27753	1.42806	1.34994	1.15866
GPAA1	43.8483	47.3495	47.3151	44.3254
PMF1-BGLAP	0.48155	1.06136	0.782093	0.820017
ACLY	40.5182	37.8038	42.4349	43.5703
RP11-384M15.3	11.674	9.40789	8.13317	8.62421
RP5-1085F17.3	2.52601	2.62329	2.21259	2.53949
RP11-304L19.5	115.547	129.346	109.48	114.392

RP11-44F21.5	1.18831	0.871084	1.05138	1.32653
CTD-2323K18.1	4.82535	4.11441	5.42744	3.65374
RP11-817O13.8	1.00679	1.24121	1.30737	1.22458
RP11-77H9.2	2.22781	2.65042	2.86264	2.32699
RP11-101E7.2	5.17315	4.03291	3.87	4.11736
AC137932.5	1.38777	1.69785	0.493451	1.29623
CNP	17.1717	15.6862	18.5642	18.6707
CTD-2033A16.2	3.3051	3.19604	2.86189	3.13688
RP11-388M20.6	49.7916	52.7362	56.7113	53.1225
VPS28	26.0232	30.664	33.3784	32.5467
COX6CP1	1.29284	0.771096	1.25483	0.529397
RP11-416I2.1	1.22005	1.13526	1.62225	1.57233
FAM83H-AS1	9.00235	9.6995	11.0629	10.3037
RP11-345J4.6	4.69464	4.97039	7.91418	6.92928
RP11-395B7.7	1.10605	1.5448	1.78844	1.91506
RP11-1035H13.3	6.80571	3.25036	1.37133	4.38784
RP11-473I1.5	4.60684	7.24654	6.69815	6.14854
RP11-152P23.2	18.9105	11.8558	17.5716	19.5563
RP11-264B17.4	2.23641	1.61263	1.59255	1.44548
RP11-343C2.9	2.77412	2.42631	3.15267	2.21318
ARHGAP23	10.3502	7.0586	10.2222	10.89
FRG1	9.09057	8.21014	7.39348	8.70853
MROH1	0.836411	0.828918	1.03862	0.861987
SERINC2	17.0865	17.4947	23.8709	22.2014
RP11-384K6.6	2.61364	3.02973	2.64098	2.5566
RP11-923I11.1	0.891954	1.17336	1.19051	1.09809
RP3-406A7.7	2.23131	1.8758	1.47398	1.74088
LA16c-316G12.2	0.846068	1.06048	1.10531	1.20803
RP11-22P6.3	6.99651	6.44596	4.88361	6.98901

RP11-449H11.1	1.23628	1.51305	1.17862	2.00247
C16orf95	7.41462	5.92843	6.16179	7.72973
RP11-541N10.3	0.947017	1.12411	1.16286	1.33129
MAPK15	4.34053	5.08271	5.15857	4.65639
RP11-63M22.2	14.0994	15.4342	14.3155	16.1189
RP4-536B24.2	8.0356	5.72434	5.88699	5.69746
CTD-2196E14.9	2.28119	2.11045	2.28229	2.43299
PUF60	158.304	138.791	146.514	136.833
CTD-3193O13.1	4.81257	5.11163	5.89426	5.0524
RPL7A	800.767	804.804	847.924	888.873
CACFD1	0.988356	1.51123	1.99793	1.62589
GGNBP2	8.96727	8.25595	8.79183	9.94706
FAM157C	2.25561	4.42011	3.62964	2.61831
NRBP2	2.89698	3.90758	3.59729	3.10434
RP11-529K1.3	4.33245	3.57267	3.52166	2.98523
RP11-252A24.7	14.3391	16.5154	16.8055	16.5385
RP11-49I11.1	1.56363	1.93907	1.77754	1.96724
RP13-516M14.1	1.31044	1.38238	1.83635	1.62156
ERVK13-1	1.56807	1.66408	1.93533	1.8173
RP11-24N18.1	108.66	100.829	96.6897	93.6418
RP11-413H22.2	3.18475	3.83022	3.44469	3.19649
VPRBP	14.9728	12.5616	14.8176	15.3949
RP11-1166P10.1	2.52729	1.88785	2.46087	1.83342
SNAI3-AS1	1.22924	1.34113	1.31496	1.0224
RP11-303E16.8	1.82959	2.3389	1.87287	2.703
RP11-46C24.6	7.55259	6.67486	5.13807	5.78536
7SK	2.04061	0.871858	1.70359	1.31506
SURF1	22.7981	26.7169	27.2302	26.6745
CTA-29F11.1	4.99609	3.7108	3.65902	3.87411

TONSL	3.36721	2.68023	2.87479	2.98177
SLC7A5P1	5.09822	4.28787	3.61095	3.23114
RP11-72I8.1	0.961983	1.13082	1.01792	0.76212
RP11-156E6.1	3.52071	2.7878	2.95007	3.68804
NT5C3B	45.0594	41.4387	47.8682	48.9833
RP11-403P17.3	3.34877	3.33549	3.48662	3.31002
RP11-226L15.5	0.873675	0.946289	1.19845	1.10275
NKIRAS2	11.0591	11.8682	12.1463	13.1281
RP11-311C24.1	16.7899	15.4706	17.5175	16.4591
GPSM1	2.92014	3.61137	4.30387	3.98114
RP11-566E18.3	5.12435	4.76658	3.62612	4.15814
RP11-161M6.2	3.43935	4.95129	5.13081	5.25442
CTD-2007L18.5	10.1762	5.95084	5.95711	6.38896
RP11-107F6.3	0.755319	0.273146	1.11434	0.471009
GS1-358P8.4	1.4568	1.48046	1.47326	1.58162
CTD-2235H24.2	12.6819	15.1636	12.2824	13.3018
AC124789.1	2.16337	1.37623	1.6341	1.32576
RP11-256I9.2	9.15135	7.98775	10.535	11.6708
RP11-264B17.2	4.45629	4.35864	5.28107	5.07679
RP11-715J22.4	5.76723	4.31147	4.53267	4.3501
RP11-211G23.2	1.56535	2.51921	1.6178	1.42344
RP11-483I13.5	3.83222	3.54965	4.16915	3.86358
CTD-2026K11.1	5.09298	7.18641	8.31159	6.76858
CTD-2012K14.4	45.9224	27.9726	28.3559	32.8448
ADPGK-AS1	1.916	1.77084	1.73727	1.46559
RP11-105C19.1	0.465137	0.896027	1.20704	1.89607
RP11-363E7.4	1.2909	2.09779	1.92913	2.11378
CCPG1	2.62661	2.41197	2.47178	2.51194
RP11-57H14.4	3.71541	4.33395	4.69165	4.4729

RP11-407G23.3	38.9571	40.5364	41.7184	42.6122
ZNHIT3	14.4434	13.67	12.4525	15.5654
LA16c-313D11.11	197.391	187.036	194.645	190.478
SCRIB	60.7075	60.9451	69.7031	60.1482
RP11-521L9.1	12.58	12.6377	13.309	13.9476
LA16c-313D11.10	12.5073	8.35904	7.74163	10.1705
CTD-2319I12.1	0.912916	1.50069	0.849209	0.777466
SLC52A2	53.4353	52.5621	58.8019	56.7945
SULT1A3	10.7881	9.8897	11.4461	10.769
RP11-303E16.2	15.0103	7.69752	8.01916	10.4797
RP11-538I12.2	4.45711	5.85629	6.28752	6.02566
RP11-264B17.3	1.96464	1.51515	1.45209	1.412
RP11-163E9.2	2.80254	2.23153	2.88996	3.00276
RP11-61A14.3	3.21523	3.58783	3.14761	3.74281
CTD-3126B10.1	11.7272	6.94056	8.5593	8.62264
CH17-260O16.3	1.06033	0.983869	0.89988	1.06292
PLEC	36.5551	40.4884	44.9444	37.5175
RP11-141O15.1	2.15675	2.14935	2.67214	2.93065
RP3-523K23.2	2.31162	2.55491	2.12815	2.67372
RP11-304L19.3	4.82942	3.67556	4.167	3.88878
HSF1	39.3992	35.76	40.2369	39.6746
RP4-798A10.7	0.874658	1.24449	0.976881	1.16098
CTD-2517M14.5	6.56096	6.857	7.17343	6.17512
RP11-20I23.6	1.59952	1.54141	1.29241	0.967329
RP11-296I10.5	0.080975	0.828811	1.80809	0
EPPK1	2.10458	2.91863	3.16645	2.63928
RP11-723O4.9	0.659711	1.00973	0.597455	0.698899
LEPREL4	6.3659	8.06833	8.75411	8.67538
RP11-517B11.7	1.13445	0.780367	0.854957	0.926771

RP11-532F12.5	15.8089	12.5597	12.9026	13.3028
CTA-445C9.14	1.62145	1.42053	1.46071	1.72677
ZNF865	8.55909	8.60428	9.91572	8.1652
CPSF1	48.8726	44.9267	52.4582	48.3819
BOP1	52.9677	45.5845	54.3648	52.3158
RP11-304L19.4	1.2426	0.74539	1.22496	1.03321
RP11-318L16.6	6.29421	7.21995	6.78253	5.01098
AC137932.6	0.880365	0.851372	1.1968	1.43544
FKBP10	12.2592	13.4042	14.9914	14.4585
PRKRIP1	4.26974	4.66246	4.66916	5.26671
SLC39A4	46.3643	47.488	53.7487	50.1609
PIGW	13.8696	10.0745	13.1415	10.8683
SURF6	13.6442	11.4504	13.1682	14.1538
KCNJ12	0.952705	1.06683	1.2017	1.3508
AC002550.5	11.8188	10.585	11.5224	12.4817
RP11-297D21.2	0.671486	1.16981	0.927778	1.38447
FAM101B	5.97295	5.72922	6.50292	7.09422
CTD-2049O4.1	0.696351	1.14197	0.506891	0.840829
AC006538.1	1.62579	1.6818	2.0034	1.77283
CTD-3185P2.1	2.15931	2.2457	1.97426	2.23589
SURF2	22.2074	17.9475	19.2587	20.3401
VPS9D1-AS1	38.6222	39.8162	45.519	39.3828
RP11-578F21.12	2.34592	1.49525	2.13745	3.00302
RAB5C	13.117	12.0823	14.4572	16.0458
FAM83H	25.5009	28.043	29.9313	26.0785
RP11-388M20.2	1.12139	1.39772	0.715565	0.96866
RP11-473I1.6	0.401635	0.802095	1.00852	0.954435
CTD-2012K14.2	1.3983	1.0883	0.833772	0.707511
TEN1-CDK3	1.1795	0.97224	1.23237	1.01635

SOCS7	10.7717	11.9096	12.5852	12.4447
RP4-616B8.4	4.00274	1.43053	0.331652	0.480108
RP5-1129J21.3	0.67478	1.14361	1.04806	1.17826
OPLAH	2.99737	4.43355	4.37076	3.73179
REXO4	10.9237	8.55446	9.98244	10.2224
CH17-260O16.2	2.20668	2.29996	2.76712	2.94918
LA16c-358B7.3	1.8169	1.82217	1.90006	1.90658
DHRS11	4.41147	3.6002	4.41777	4.46342
RP11-46D6.1	2.44324	2.40908	2.6148	2.64526
ZNF707	3.83516	4.49975	4.1168	3.7008
RP11-408H20.1	1.29938	1.17984	1.32436	0.930086
CTB-31O20.2	1.99893	1.80601	1.57702	1.86883
FBXL6	29.3848	28.6215	33.1088	31.0919
RP11-264B17.5	1.31368	0.878686	0.583569	1.08109
RP11-29G8.3	0.420743	1.13815	0.422576	0.441334
RP11-296I10.6	1.62797	1.27391	1.5059	1.34208
GS1-393G12.12	9.67984	8.29532	10.2455	9.59941
RP11-680G10.1	3.6253	3.18133	2.87192	2.11505
RP11-259G18.1	1.08514	0.965407	0.556501	0.541824
JUP	38.0474	29.7553	34.3196	46.6371
MED22	4.98138	5.1798	6.20458	6.16576
TMEM249	1.34063	2.20777	2.28191	1.96723
HERC2P8	0	2.2684	2.37784	2.66437
CTD-2033A16.1	58.4724	52.976	25.8316	33.2735
ABC7-42389800N19.1	2.84095	2.71842	2.91633	2.97855
MIR4720	6.5097	4.02098	5.06241	4.80578
RP11-20I23.13	1.07242	1.13654	0.979306	0.980252
LA16c-390E6.5	20.951	23.6371	25.1245	21.0793
RP11-529E10.6	1.57939	1.3235	1.663	1.46723

LA16c-313D11.12	3.79803	3.12038	3.27973	3.03456
RP11-304L19.11	1.31284	1.7254	1.45587	1.44667
PTPLA	5.42094	4.97518	4.7418	4.78618
RP11-265N6.1	9.75974	10.343	9.02751	9.93117
RP11-401P9.4	1.09098	1.2805	1.0768	1.31572
RP11-473C18.3	1.41909	1.76481	1.26361	1.4637
AC009133.12	2.93304	3.06432	2.06121	2.91204
LA16c-366D1.3	10.4507	10.569	12.3366	10.8586
ADCK5	3.15373	3.79101	4.30197	3.81698
DGAT1	19.3123	21.7251	25.3224	23.4693
RP11-61A14.2	4.19784	4.69598	3.76116	4.44171
RP11-264M12.2	1.72754	2.78789	1.91165	2.03186
SSTR5-AS1	0.705493	2.1712	1.69022	0.780897
RP11-77K12.1	4.92036	0.982204	1.08829	0.706813
GRINA	99.0061	102.81	114.865	96.2852
CTD-2358C21.4	1.22612	1.52515	1.5077	2.70888
RP11-345J4.5	59.5692	51.9126	49.8868	55.2079
RP11-626G11.3	13.658	18.6686	16.2028	16.7744
RP11-1223D19.1	31.6423	37.7096	39.3536	40.0858
RP11-650L12.2	7.94614	6.69547	6.48425	6.61136
SLC2A6	2.4702	2.53005	2.95975	2.74015
RP11-529K1.2	1.4863	1.41168	1.23285	1.59167
RP11-69H7.3	32.7694	19.8109	19.7672	19.9291
CTD-2354A18.1	11.5392	9.17944	11.9685	12.7785
RP11-252K23.2	1.03592	1.57568	1.48852	1.1953
AC005606.14	23.4215	18.1687	20.5991	19.0083
RP11-350O14.18	0.323939	0.143463	1.16512	1.07221
ISY1-RAB43	1.04196	1.26001	1.40533	1.13866
RP11-283I3.6	1.22226	1.48785	1.39672	1.3912

LOXL1-AS1	5.07444	6.3614	6.44315	7.10771
RP11-680G24.4	1.53706	1.1839	1.66003	1.48887
RP11-265N6.2	17.3486	11.7135	12.4912	13.1117
LINC00662	7.39082	10.6895	9.0058	9.33002
RP11-146F11.1	0.395171	1.27072	0.694184	1.13268
RP13-638C3.4	19.287	9.63057	13.0158	14.3475
HYAL3	1.81657	1.76269	2.294	2.50979
RP11-110I1.5	3.87431	4.19083	5.0961	5.37654
NAT6	2.57737	2.90539	3.05349	2.84588
RP5-867C24.5	2.79139	2.49	2.22339	2.99453
PMS2P3	1.32984	1.24915	1.40399	1.25986
HMBS	2.42295	1.60532	1.93217	2.04031
CTC-479C5.12	13.3185	8.19968	9.40063	9.03843
C5orf30	3.95541	3.14402	3.5025	3.37079
CLK2	13.2669	13.5403	15.7565	15.2353
RP11-314A20.5	1.19095	0.953138	0.952816	0.968441
ZNF518A	3.45351	3.81303	3.81112	4.52912
ABC13-48559800H2.1	8.58551	8.10651	9.58061	9.04835
LENG9	0	1.08493	1.35538	0
RFC2	35.4288	29.1905	30.7338	33.4601
MBOAT7	6.5869	7.75411	8.02451	7.43122
GTF2IRD1	6.32787	6.88314	7.0896	7.14297
HYAL2	22.1755	20.5518	23.5103	22.4728
RP11-388M20.9	14.5082	12.5855	14.2518	15.1982
BDP1	1.58458	1.96873	1.62621	2.43346
RP11-461A8.1	3.40555	3.81289	4.17339	3.84825
DNAJC30	1.87868	2.1233	2.32663	2.32228
RP11-396K3.1	4.74924	5.03127	5.30736	5.77779
CTD-3148I10.1	0.885339	1.06396	0.961455	1.05346

POM121B	4.11634	3.54023	4.01694	3.97372
RP11-473M20.7	4.68393	6.04111	7.78151	6.60499
CWC15	27.6552	24.5711	22.5703	26.9787
RDH13	2.37442	1.71119	2.23811	2.20521
GATAD2B	2.34044	2.62934	2.50962	2.88805
DLGAP1-AS2	2.41286	3.49708	2.90588	2.83534
RP11-676J12.7	1.4965	1.20943	1.37857	1.53057
SEMA3B	1.83762	2.92963	3.86543	3.33886
VPS37D	1.83322	2.04027	2.15542	2.04198
ZNF100	1.04924	0.925517	0.961928	1.11606
JRK	2.08108	2.40152	2.58849	2.36141
TCF20	6.13433	5.10802	5.94037	6.62813
FDXACB1	1.8496	1.73207	2.02553	2.18466
WBSCR22	92.4319	96.2164	108.869	115.675
TMEM97	20.3833	12.4352	15.2101	16.2121
RP13-1032I1.7	38.4891	36.961	40.51	35.702
MBOAT7	6.56411	7.76884	8.02564	7.43961
MCCC2	2.83869	3.23626	3.4652	3.89828
LIMK1	13.3415	11.2994	16.4876	16.4326
KRTAP3-1	0.893395	0.967457	0.836358	1.06767
CLDN3	2.04607	2.68878	3.39237	2.82404
MLXIPL	3.00852	3.57517	4.25824	3.98722
RPS25	481.691	413.405	408.199	476.899
RP11-589P10.5	0.648717	1.3404	1.21354	1.48431
EPS8L1	0.711044	0.844668	1.09004	0.915752
RDH13	2.37597	1.71038	2.23861	2.20281
RP11-670E13.5	24.4064	19.3924	20.0796	21.537
POM121	8.24996	7.40374	8.24935	8.65816
ZNF558	2.07229	2.16049	2.48064	2.52345

LAT2	1.59725	1.48594	1.52827	1.46718
AC012314.1	1.47217	1.3873	1.70761	1.65644
CTC-479C5.11	1.51661	0	0	0
XRCC6BP1	4.19817	4.52062	4.61358	5.35386
RP13-638C3.3	1.95805	2.33996	2.31386	1.90096
RP11-96D1.11	9.91261	6.97077	7.85015	8.71613
RDH13	2.37096	1.70887	2.23765	2.20279
GTF2H2	14.8122	14.283	13.6914	16.5977
SMN2	55.3324	23.258	25.2726	33.9508
NLRP2	3.18106	3.04641	3.24289	3.46154
C11orf1	20.9371	22.088	22.2643	24.9522
SLC46A1	3.80272	3.58244	4.31802	3.82922
CTD-3195I5.5	2.53009	2.15865	1.77277	1.77385
SRCIN1	0.780009	0.860627	1.04854	1.20515
LINC00674	1.48553	0.489796	0.512518	1.24671
RDH13	2.37345	1.71184	2.2356	2.20297
CTD-2031P19.5	4.86519	4.33544	3.49122	3.937
RP11-876N24.4	10.0753	10.5777	11.2401	10.6357
EPS8L1	0.711044	0.844668	1.09039	0.915752
CTD-2292P10.2	5.53603	6.35684	7.51443	7.22703
PPIP5K2	9.26963	8.13682	7.70224	10.3581
RDH13	2.33222	1.68298	2.20357	2.17055
CORO7	5.6311	5.71162	6.27496	5.55749
RP11-199F11.2	1.06445	0.923275	0.812702	0.911666
SMDT1	2.28489	2.33443	2.16883	2.2611
NLRP2	3.18104	3.04601	3.24575	3.4639
GTF2H2D	14.9951	14.8456	14.435	17.1141
STYXL1	11.6394	13.2944	14.1703	16.7007
SLC16A6	0.756835	0.986643	1.06183	1.32544

DIXDC1	1.16484	1.71318	1.40852	1.34619
EPS8L1	0.711044	0.844668	1.09027	0.917284
SBDSP1	10.453	7.85036	7.97013	9.13981
SHPK	1.15986	1.30048	0.985676	1.43663
SERF1B	13.5234	13.4563	13.1912	15.8292
TMEM120A	10.678	9.70672	10.8902	10.8334
AGK	5.20303	4.06147	5.68962	5.46879
NLRP2	3.18087	3.04687	3.24767	3.46218
VPS11	3.33038	3.66042	4.09806	3.97834
RDH13	2.3685	1.71178	2.23501	2.20272
DOC2B	3.90219	3.85207	3.42269	3.40516
RP11-1275J23.2	2.98598	2.48802	2.7402	3.04761
CNOT3	2.91083	2.53249	3.15804	3.06733
KRTAP2-5P	0.298304	0.798535	1.23772	0.710993
TTC12	6.31875	6.32206	5.49882	6.60181
CCDC125	1.88625	1.12967	1.79349	2.16677
RP11-670E13.3	2.3586	0	0	0
RP11-498C9.3	9.56501	7.292	8.97061	7.681
RP11-490O6.2	1.04447	0.92975	1.0123	1.00964
RP5-1050D4.3	8.13693	9.37826	10.7501	10.4649
EPS8L1	0.711044	0.844668	1.09007	0.915752
ABC7-42389800N19.1	2.84095	2.71842	2.91633	2.97855
GBA	11.1816	12.9652	13.9297	14.2966
TNFAIP1	7.23951	6.38739	9.29149	8.9853
8-Mar	2.23354	2.19139	2.11956	2.30998
SPC25	0	9.76463	8.62436	0
RP11-65J21.3	1.43779	0.801368	0.738577	0.818576
RP1-59D14.1	4.51702	4.21708	4.90321	4.46035
POLDIP2	36.3982	33.0158	38.045	39.3764

EPS8L1	0.711044	0.844668	1.09023	0.915752
KPNA2	167.737	129.849	142.868	160.456
GART	7.93114	7.64718	6.94293	8.73401
RP11-1275J23.3	3.42734	2.83431	2.95628	3.63743
TUSC2	20.96	18.8617	22.4331	20.9899
RRAS2	18.9329	17.3535	17.9051	21.2732
RP11-846E15.2	0.984452	1.04504	1.33177	1.14551
WBSCR27	3.99144	6.04448	6.16831	5.66542
RAD17	5.8515	5.31698	6.31027	7.35683
CTD-2545G14.7	0.013827	0.03472	0.0483301	1.02917
LA16c-349E10.1	1.29391	1.26896	1.24738	1.13579
RP11-667K14.4	95.0585	107.122	108.612	105.809
LA16c-360H6.2	4.73589	5.66808	5.73024	4.91777
POR	23.2078	24.2808	28.2811	24.9465
ALG9	5.97857	6.17265	5.969	5.87363
RP11-334C17.5	1.27447	2.46479	2.18164	1.73902
NSUN5	26.4739	24.7271	28.018	28.7631
MRM1	5.9662	4.59901	5.54634	6.26101
C11orf52	0	0	1.00314	0
CTC-786C10.1	3.58843	2.81909	3.20874	3.23533
LENG8	14.8056	10.5374	12.1881	15.6415
MRPS36	6.71519	5.58742	6.22813	7.23058
NLRP2	3.18104	3.04642	3.24062	3.46187
RP11-104H15.9	4.1067	3.88692	4.35765	4.71001
SKA1	7.25207	5.51846	5.84551	6.80444
OVCA2	27.4375	27.3822	27.5854	29.4199
FAM189B	16.3018	14.5152	16.9251	15.437
OCLN	1.75119	1.35224	1.53123	1.93508
SLC37A4	23.5329	22.8311	23.1117	22.0543

RP5-1050D4.4	0.985968	1.08248	1.58726	0.960005
DNAJC7	34.2325	29.3909	29.5883	31.3846
BAZ1B	16.3267	13.1056	13.9347	15.7459
NSUN5P2	7.72203	7.62917	8.00962	8.85512
RP11-295D4.1	5.22197	4.07118	4.89253	4.582
TSEN34	8.8223	7.35144	9.25387	9.27085
KRTAP2-1	0.388189	0.593272	1.09087	0.939225
CTD-2135D7.2	2.23462	2.09274	1.78646	2.2811
PIH1D2	1.16799	0.808336	0.993963	1.24504
RHBDD2	16.2646	17.5564	15.7953	18.0672
AMZ2	46.813	46.9028	47.843	53.8968
CTD-3195I5.1	6.89616	5.8161	7.11386	7.29087
AC006014.7	1.67283	2.00061	2.08065	2.30944
CBX2	7.08567	6.00058	6.97208	6.81976
PPP2R1B	3.52948	3.68846	4.15188	4.50513
SSBP1	82.867	68.5359	75.5807	86.4067
CIRH1A	28.6039	22.4073	26.4325	28.5135
RP11-961A15.1	6.57307	8.02458	7.27194	6.73846
IFNGR2	13.403	10.7047	10.5553	13.0812
NLRP2	3.18278	3.04599	3.24237	3.46154
HYOU1	33.9634	27.0371	35.6682	36.0086
MRPL12	299.528	276.327	294.409	275.73
INTS3	9.34395	10.6901	11.6938	11.2578
RP11-498C9.2	478.813	497.849	499.857	514.674
EPS8L1	0.711044	0.844668	1.09009	0.915752
CDK7	25.6295	22.2405	24.6259	28.9417
MDH2	168.107	163.438	193.439	188.57
BPTF	13.4823	12.614	14.132	14.0497
LSM14A	33.2846	26.4178	29.7561	34.3956

KRTAP2-3	7.68776	8.0015	12.065	11.8517
IFT20	5.2663	5.65253	5.3439	6.28997
TSEN34	8.82317	7.35201	9.24778	9.26999
CAPN5	1.59679	1.52858	2.11273	1.993
EPS8L1	0.711044	0.844668	1.09023	0.915752
RP11-156P1.3	7.52032	9.27952	8.64231	8.86514
NLRP2	3.18104	3.04668	3.24137	3.45193
CU467002.1	1.63279	0.623138	0.708379	2.6744
SMURF2	25.5959	23.5552	24.2757	27.9875
POLG2	10.8243	10.8003	9.19721	11.6525
FAM58A	19.4444	15.0672	16.3041	14.7802
NLRP2	3.18293	3.04599	3.24822	3.46297
ALOX12P2	4.94288	5.05655	4.86334	4.60831
RP11-473I1.9	6.46561	10.1318	8.12117	7.49109
ABHD11	19.6526	17.9772	20.6863	20.4471
RP11-490M8.1	0.858802	0.931799	0.844179	1.14025
RP11-294J22.6	46.5772	46.2586	46.7387	49.1963
CYP2R1	3.80638	3.49289	3.48847	4.0606
ACER3	2.10772	1.77872	1.72614	2.02192
RP11-1151M9.1	2.87794	2.35244	2.48341	2.87752
RDH13	2.3299	1.68346	2.20254	2.16933
CLDN4	23.2698	22.5277	27.9558	26.0148
RP11-846E15.3	0.630061	1.05307	0.587194	0.78175
RP11-110I1.14	16.3244	15.7244	18.5247	18.7831
GTF2I	12.64	11.9541	13.0153	14.5728
RP11-166P13.3	0.653331	1.37105	1.40819	1.18197
RASSF1	4.313	3.16636	4.24796	3.97804
ROCK1P1	1.67324	1.78251	1.5184	2.03969
WBSCR16	14.232	12.9801	15.3996	15.4871

STX1A	5.39089	4.60038	5.30379	5.15311
GIN1	3.88455	3.16382	3.02156	2.81587
DLAT	24.3004	19.4673	23.0338	25.5426
RP11-124L9.1	1.61553	1.60833	1.86018	1.82976
MTX1P1	8.56947	8.09142	9.56317	9.03186
PRR4	4.24282	3.32728	3.26779	3.68735
MARVELD2	3.70551	3.3135	4.06667	4.05037
NSUN5P1	10.1616	11.0393	11.854	11.4132
RP11-28B23.1	1.10287	0	0.462994	0
CEP95	8.57317	8.03576	7.58906	8.7694
TBL2	14.0535	12.6361	13.85	13.1105
RP11-667K14.3	0.663744	0.732287	0.835467	1.12044
HSPA12A	2.49081	2.66448	2.85812	3.20153
RP11-1055B8.6	3.4168	2.70623	2.90669	2.55822
CU467002.2	0	2.86053	3.02702	0
STAG3L3	3.52159	3.94499	3.55422	3.7775
RP11-473M20.14	2.8481	3.72854	3.08049	3.70314
ARSG	1.23652	1.5667	1.5503	1.80186
RPS9	72.1881	75.6985	80.0186	86.2932
DDX5	141.953	132.994	140.035	150.307
C17orf58	10.8154	11.04	12.0222	12.3185
IFRD2	59.4056	53.1553	54.7781	59.1134
RP11-95P2.3	0.544639	1.54061	1.24338	0.753799
RP11-124L9.2	3.67398	4.34777	3.48312	2.80616
CTD-2574D22.6	5.69162	10.3262	8.88215	14.4104
MEGF11	0.575204	0.594683	0.927137	1.26638
LRRC37A17P	0.939696	1.52269	1.41227	1.73742
BCL7B	21.2063	17.8756	21.4289	21.1528
MYZAP	1.21368	1.22164	0.139512	0.254935

GNAI2	46.7507	28.1084	42.8906	43.8383
TMEM50B	7.74274	6.77116	8.51128	9.6862
SLC27A3	2.96604	2.21866	2.42149	2.77744
RP11-810M2.2	16.6217	15.4567	18.3483	16.9762
RP11-542C16.1	2.06107	2.5803	2.07572	1.92776
CCDC84	6.46404	6.06256	6.15173	6.16145
RP11-517A5.4	3.81904	3.71957	4.02468	3.96525
CHTF8	13.0061	10.5605	13.5577	10.9306
CTD-2561B21.7	33.4019	39.9213	41.3026	36.9088
CLIP2	4.72573	4.27093	5.06555	5.00377
RP11-461A8.4	1.50215	1.44204	1.41395	1.30326
RP11-473I1.10	6.34694	8.94385	7.57843	7.63183
PRR4	1.09583	0.676427	1.04182	1.16425
ZNF430	1.27843	1.52135	1.13773	1.32669
RDH13	2.36845	1.70929	2.24067	2.20152
CTC-524C5.2	5.282	5.57288	5.03842	5.81784
SCAMP3	31.9422	24.5788	28.4944	29.2854
EPS8L1	0.707968	0.844668	1.09021	0.915752
RP11-104H15.8	1.78493	0.77427	0.743577	1.6755
TAF9	55.366	51.4297	55.3932	62.6535
KRT23	2.26667	2.74539	3.5004	3.37319
HCN3	1.1829	1.19607	1.31965	1.22979
RP11-1264N5.1	1.38963	1.35184	1.75983	1.57279
RPS9	114.915	116.634	125.366	136.474
HIP1	2.30078	2.70585	2.84159	2.93838
AF001548.5	0.8497	1.05626	0.805856	0.935077
EIF4H	121.107	100.259	110.42	123.065
RP1-59D14.5	1.26911	1.89296	1.62533	1.67002
RP11-13N13.2	2.36933	2.31218	1.79011	1.97514

CTC-297N7.10	29.4814	35.452	40.3184	35.0745
AC005702.1	0	0	5.10709	7.58423
MIR5189	0	0	2.12335	0
PIAS3	7.14071	4.81899	6.92884	6.88871
RP11-735A19.4	14.8755	15.3988	18.3222	15.8323
RP11-160O5.1	2.82728	2.29967	2.38652	2.41761
FAM72C	3.64915	2.67502	2.49486	3.61438
HIST2H2AC	2.25878	1.12884	1.17458	1.17372
IKBKE	1.33194	1.50696	2.12059	2.15506
AK4P1	1.57786	1.75344	1.22067	1.36933
UBBP4	6.06992	6.27451	5.85689	4.69295
RP11-498C9.13	228.8	264.243	230.704	231.571
MIR3936	5.49457	0	2.28474	3.35772
RP11-599B13.6	0.680447	0	1.34693	0
U6	0	0.656968	1.41767	0.816269
AF235103.1	5.29188	0	2.17891	3.21212
RP11-927P21.5	0.851796	2.03346	1.33557	1.26466
FAM72A	5.02334	3.73094	3.51481	4.55902
RP11-597M12.1	1.69804	3.2011	3.67698	2.48589
RP11-498C9.15	4.80101	3.18173	3.36505	3.52375
RN7SL4P	1.44319	1.58811	113.599	0.250429
XXbac-BPG181M17.6	15.1097	13.2835	12.0565	11.0908
RP11-580I16.2	45.1689	45.7126	54.22	52.5994
AC005702.2	0	0	6.32213	4.52768
RP11-326K13.4	1.35422	1.23402	1.22812	1.5324
RP11-649A18.12	1.18483	1.29347	1.61438	1.08329
RP11-498C9.16	5.97726	4.13697	4.08681	4.67416
AL353629.1	13.03	11.6812	2.75551	8.13152
RP11-51L5.5	1.65462	1.54955	1.60534	1.76625

U6	0	0.656968	1.41687	0.816269
RP11-214C8.5	3.52109	3.20116	2.92156	3.24071
RP11-53I6.2	1.93501	2.29807	2.13452	1.51427
SNORD3A	21.8937	17.0729	17.7591	19.2087
MIR4461	0	0	14.2873	0
RP11-789C17.5	0	0	0.394654	1.7776
CTB-58E17.9	5.10532	3.96125	4.96765	4.67566
SF3B4	45.2556	36.4553	41.4628	45.4299
RP11-746M1.1	74.2906	70.2288	69.9443	70.0473
RP11-6N17.6	41.8504	48.1282	40.5823	42.5914
RP11-68I3.2	1.98412	9.54311	6.31962	4.41284
RP11-192H23.7	24.8559	23.2571	24.2067	24.6545
MIR4737	0	0	0	4.35814
KRT222	1.51101	1.57312	1.82289	1.68408
DND1P1	0.779725	1.29411	0.943215	0.775659
VPS45	4.8276	4.62383	4.70498	5.38711
RP11-159D12.2	8.27681	10.8095	10.9627	9.51976
RNU4ATAC	0	2.26323	4.79033	0
LINC00909	5.33455	5.06972	4.75461	5.33153
AL603831.1	40.6872	64.1197	59.4274	62.1367
RNVU1-12	1.01818	0	0	0
CTD-2514K5.4	10.8331	12.3036	12.1289	11.2456
CTD-2031P19.4	531.055	486.286	464.201	454.62
RN7SL657P	1.20712	0.425394	0.379218	0.723271
RP11-680F20.12	2.74835	2.59932	2.59009	2.69987
GPR89A	4.72248	3.70357	3.57763	4.20944
SNORA77	8.1381	2.44863	0	0
RP11-159D12.9	8.28598	5.92271	5.33444	6.10217
TAMM41	3.00522	2.273	2.8588	3.13074

RN7SL208P	0.720254	0.440033	1.15137	1.01849
MIR3180-5	1.57521	0.925602	0	0
FAM72D	3.59795	2.81537	2.61196	3.7103
RN7SL718P	1.22813	1.26395	1.04214	1.19934
FAM229B	3.57941	3.70877	3.75978	3.80646
RP4-594I10.3	0.945203	1.56541	1.40192	1.4334
MIR3648	30.699	31.3708	14.2201	32.3325
RN7SL731P	8.00613	5.91945	4.27471	4.82841
OTUD7B	2.61979	2.22818	2.24918	2.51639
MTMR11	1.63474	1.70848	1.49643	1.97649
TUBE1	6.72756	7.90772	7.36277	7.11158
RP11-343H5.4	1.26548	1.23414	0.635065	0.970013
SUZ12P	10.7037	9.37064	9.25955	10.4144
U6	0	0.65715	1.41691	0.81713
RP11-138C9.1	550.777	453.267	493.228	529.494
AC010761.8	63.1321	56.975	59.2994	61.2964
RP11-360L9.8	26.4931	25.8785	30.5268	27.6533
AL158069.1	5.19906	0	2.14305	3.11842
RP11-192H23.8	1.16728	1.24655	1.10908	1.81766
MIR3615	0	7.15992	5.66877	0
RNA5SP314	0	1.02705	0.452082	0
RP11-524F11.2	4.70994	3.27262	3.76425	4.12427
RP11-498C9.12	3.89946	2.49027	2.68932	3.25331
SNORA67	26.59	13.6557	9.52695	19.5786
GPR89B	3.13887	2.55197	2.69553	3.24387
POLR3C	8.34763	7.12222	8.10349	9.00961
FAM72B	3.49387	2.4713	2.66377	3.34419
AL592188.4	0.538079	0.888073	1.04864	1.17318
AGAP4	1.78036	1.66547	1.80395	1.84804

RP11-75N4.2	2.16001	2.12343	1.96026	2.16359
MEST	21.6468	22.7191	24.1543	24.9337
RP11-815I9.4	1.51827	1.55953	1.38763	1.34273
RP11-6N17.4	3.11418	5.1477	6.39517	5.67577
AC100830.5	1.9451	0.872585	0.204197	0.907183
U6	0	0.656968	1.4354	0
CHD1L	7.91713	7.2362	7.39076	9.83367
RP11-344B2.2	2.0786	2.33116	2.33811	2.56088
MIR3652	180.693	183.365	368.44	385.117
AC010761.6	1.33829	2.52573	2.40695	2.17295
MIR3942	0	0	2.41532	0
RP11-227G15.2	1.3301	2.59971	1.85142	2.3412
RN7SL2	494.112	560.398	439.686	233.547
RP11-192H23.5	14.6938	12.1198	11.7874	9.52171
DYRK3	1.37605	1.49434	1.46202	1.29248
RP11-70L8.4	1.65376	1.59121	1.38668	1.67331
PDIA3P	2.76849	2.42461	2.42638	2.93632
PRPF3	11.2011	9.66526	10.3451	10.5443
RBM8A	61.2991	53.8654	58.1754	64.7517
RP11-649A18.7	2.24786	2.19581	1.83265	2.39961
AC073343.2	0	9.5768	0	0
RP11-21J18.1	12.2957	14.2961	16.9545	18.6319
RP11-135L13.4	9.40725	9.13958	8.90501	10.0741
ACP6	2.89718	4.09213	3.75023	4.02671
RP13-104F24.3	2.09503	2.73077	2.47017	2.67147
CTD-2510F5.6	1.82651	0.073783	0.0281981	0.686624
ANP32E	24.6185	18.0111	18.4173	21.7323
TIMM23B	31.621	26.4864	30.4048	35.3579
CTD-2517M22.17	34.9138	24.001	31.7994	28.9204

RP11-138I1.4	1008.98	968.014	978.628	921.307
RNU6-1030P	0	0.658177	1.41687	0.816487
CTD-2510F5.4	37.2393	34.9796	25.6771	31.1055
HLA-ABC	38.4628	39.3049	44.3382	40.3427
RP11-421E17.4	11.0503	12.7106	15.2815	10.1015
RP13-20L14.6	2.24371	1.78664	2.86267	2.71596
AC010761.9	4.67835	4.28225	4.15353	3.55166
DTX2P1-UPK3BP1-PMS2P11	1.11514	0.874418	0.859105	1.06534
RNF115	8.80413	8.63986	9.4263	9.91676
SENP3-EIF4A1	1.62127	1.70767	2.12106	1.8683
AL031721.1	2.91416	0	0	0
FAM21C	4.28164	3.89614	3.83619	4.23777
MIR3918	0	10.2571	0	0
BOLA1	8.92238	7.89999	8.67213	8.96877
C21ORF59	15.3272	3.87234	14.5698	7.76186
CEP41	2.31146	2.50611	2.25971	2.50559
MIR5010	0	2.22311	0	4.72656
CTB-96E2.7	5.58156	5.66867	5.3927	6.09941
AC005702.3	0	8.37697	5.96826	0
RP11-1376P16.2	1.89008	2.11211	2.95511	1.96817
RPL17	1497.17	1452.01	1335.61	1518.38
MAFG-AS1	9.2604	11.6113	10.9363	9.84748
RP11-815I9.5	3.70867	0	0	0
RP11-848P1.3	0.583901	0.518134	1.16618	0.81014
RP11-849F2.5	0.892223	0.452622	0.899318	1.22728
CTB-96E2.7	3.70337	3.92834	4.0794	4.84254
ZNF488	1.23577	1.02171	1.46111	1.44091
RP1-56K13.3	57.8855	59.5112	62.2202	73.0374
SRGAP2B	2.45275	2.64421	2.42187	2.18735

RP11-649A18.5	1.44079	1.30962	1.06088	1.01337
AL592188.6	0.538079	0.888073	1.04864	1.17318
SEC22B	25.1084	24.0414	21.3578	24.3125
EEF1E1-MUTED	0	1.20016	0.758497	1.11433
EIF2D	21.9294	21.6283	21.6993	23.348
PEX11B	6.8258	6.94458	8.58341	8.33752
AC010761.10	33.6074	40.928	44.1732	50.6344
CBWD3	11.5929	9.46086	10.5256	12.4788
ESRG	4.3697	3.9617	5.26201	5.75803
SRGAP2	6.74679	6.69491	6.9058	6.85926
RN7SL3	21.2846	21.9307	19.7534	11.9079
BAHCC1	4.32929	3.64104	4.52548	4.56716
RP11-159D12.5	23.8094	19.1865	19.6587	18.7076
RP5-1028K7.2	1.06802	1.25621	1.33348	1.1834
RASSF5	1.02829	1.10941	1.38844	1.46441
BCL9	3.76003	4.16586	4.22732	4.49726
RP11-769O8.1	1.1675	1.07832	1.08969	1.3301
STRADA	17.6618	19.9914	20.4298	19.1013
PRKAB2	2.17702	3.46268	2.92165	3.20497
CTD-2267D19.3	1.35233	2.29297	2.5668	2.16857
HIST2H2AA4	1.53629	1.50833	1.83329	1.77998
U6	0	0.657372	1.41687	0.817432
RP11-640I15.1	3.56717	2.93706	3.34911	2.96911
RN7SL138P	3.71376	1.53594	1.17989	1.88602
RP11-627G18.1	0.732348	1.0104	1.15453	1.10329
APH1A	45.1855	37.4342	42.64	46.498
NBPF15	2.77483	3.18531	3.41405	3.30033
RP11-848P1.4	0.79317	3.75016	4.57374	3.77557
RP5-890E16.4	70.4242	66.6125	86.373	68.2464

RP11-710M11.1	1.3084	1.69705	1.42076	1.2438
RP11-874J12.4	1.39299	2.09844	2.23464	1.9155
SNORA76	108.739	126.272	130.02	99.865
NCOA4	17.8831	17.0084	18.4606	19.9116
CTB-131K11.1	13.831	14.1248	12.684	14.7965
AC242988.1	30.7321	25.3712	28.9137	30.0108
CTD-2349P21.9	7.96462	6.37657	8.46016	8.26998
RP11-17J14.2	3.68683	3.54593	3.12895	2.75407
MIR3934	0	0	0	9.15271
MAPKAPK2	11.9211	11.2871	13.4815	14.6244
RP11-138I1.3	4.92075	2.2204	3.52623	2.31255
RNA28S5	1323.97	1413.77	1577.19	1420.63
SRGAP2C	1.62231	1.3104	1.46636	1.6555
MYO15B	3.19977	5.17909	5.44896	4.31147
POLR3GL	5.03457	5.54586	6.28764	6.28938
MIR3661	0	0	0	5.07988
RP11-690G19.3	1.1671	1.68047	1.74409	2.00846
RN7SL524P	1.51166	0.596989	0.576123	0.326729
NOTCH2	4.24239	4.08694	5.27868	3.77058
COPG2	13.357	16.2346	17.7575	16.6474
RP13-104F24.1	2.2708	2.53093	2.45174	1.87793
RP6-114E22.1	1.55559	1.9619	1.94585	1.58996
RP11-19P22.8	2.75496	3.74944	2.10335	2.54907
LINC00663	0.690851	1.44699	1.17977	1.17778
CTC-448F2.6	0.820835	0.929576	1.09412	0.964019
CTD-2085J24.4	1.67288	2.41127	2.01349	2.26792
RP11-1252D15.1	5.99453	0	0	1.64335
CTC-479C5.16	1.29477	1.69744	1.53122	1.21042
AC005559.2	1.43046	1.98342	2.29673	2.10056

AC104532.3	1.23487	2.02914	1.03299	1.60083
RP11-618P17.4	10.9429	7.30059	7.66699	6.13805
AC005786.3	0	1.02184	0	0
RP11-400F19.6	9.14384	10.3473	10.4457	9.6719
FXYD1	2.91747	3.42973	2.76399	3.32936
AARSD1	67.6799	56.7895	57.8813	58.4393
RP11-773H22.4	8.9549	6.59273	6.29829	7.26102
CTD-2369P2.5	12.5894	11.9287	12.3861	11.5529
DHX40P1	2.56295	2.75159	2.1679	2.35354
RP4-657D16.3	13.8668	13.353	14.5351	14.478
RP11-886H22.1	0.806522	1.09636	0.733231	0.485605
CTB-31O20.3	20.9853	18.9766	22.7923	20.6532
RP11-108P20.1	2.39118	2.45195	2.32525	2.19131
AC011558.5	261.846	186.056	264.652	291.864
LRRC37A16P	1.49178	1.18854	1.11008	1.2372
RP11-92C4.3	7.03474	6.9363	8.8412	9.11781
AC092299.7	24.6252	31.4446	32.1628	30.4193
CTB-50L17.7	0.58897	1.00476	0.766642	0.638541
RP11-35G9.3	3.22548	2.82642	3.05623	3.23
RP11-589P10.7	5.42403	4.89139	3.88253	4.5834
RP11-566K11.7	1.81424	1.10941	1.02526	1.05687
AC002398.13	3.53857	3.70589	2.72889	3.60391
AC005336.4	2.5278	2.49884	2.10008	2.27407
UQCR11	24.5948	24.2338	23.3818	23.3023
CTD-2659N19.10	420.484	287.264	230.757	256.659
RP1-212G6.7	3.58146	4.24811	3.51062	3.4983
AC005256.1	13.4439	17.4647	16.8172	13.7439
ASB16-AS1	9.49906	10.4837	9.2967	8.55242
CTB-25B13.9	1.49979	1.27901	1.51193	1.21085

ILF3-AS1	6.51996	6.60313	7.03469	7.62813
CTD-2369P2.4	112.742	98.1947	96.344	91.6012
C19orf82	3.0972	4.53449	3.92276	4.43672
CTD-2587H24.4	2.82632	1.726	2.7267	3.61515
RPL10P15	1.48452	0.871754	0.339859	0.3025
CTD-2357A8.3	1.91993	2.20123	1.66601	2.6506
AC005262.3	7.14027	5.40403	5.28892	6.35014
RP11-322E11.6	1.22007	0.142352	0.418112	1.06404
RP11-677O4.6	2.23038	2.1999	2.02538	2.54805
CTC-548K16.1	1.12579	2.03634	1.56097	1.38889
CTB-54O9.9	1.07433	1.80748	1.4699	1.61812
AC005943.5	1.56742	1.50469	1.67146	1.6956
RP11-78A19.3	16.6939	19.493	17.9094	20.1509
CTB-55O6.12	1.25185	0.95786	1.15127	0.976781
CTB-179K24.3	12.544	8.83097	11.5546	11.5995
RP11-798G7.6	2.59585	2.58554	1.91544	1.43907
MIR132	0	0	0	5.46272
AC005624.2	1.74578	1.41293	0.988224	0.590647
IER3IP1	2.01517	1.15676	1.37493	1.02231
CTB-31O20.9	3.4493	2.13626	2.42026	2.39444
CTB-31O20.4	1.25914	1.74396	1.9505	1.5228
CTD-2319I12.4	1.88437	2.08982	1.91182	2.2177
CTD-2132N18.3	8.52402	3.97449	2.23432	3.3195
CTC-550B14.7	1.07915	0.914914	0.75915	0.718459
MAP3K14-AS1	1.49867	1.38333	1.75021	2.00975
RP11-879F14.2	2.22428	1.82811	1.58256	1.56403
CTB-129P6.4	2.17965	1.73492	1.92902	1.38474
AC005306.3	16.3465	16.4855	18.8096	15.1488
RP13-890H12.2	3.24528	3.48075	3.06995	2.8776

CEBPA-AS1	1.14891	1.04	1.367	1.45026
LRRC37A16P	1.11867	0.922204	0.970899	1.0867
CTD-2369P2.12	4.84645	1.92412	1.64496	5.29507
RP11-333J10.2	0.415635	0.249759	0.177404	1.04152
CTB-25B13.12	4.88852	5.77257	6.15286	6.17706
RP11-178C3.1	1.24114	1.13234	0.747747	0.95992
RP11-1094M14.11	4.67157	3.99731	3.8485	4.12644
SLC25A1P5	3.42126	3.19134	3.73074	3.64265
RP11-242D8.3	0	3.29003	1.63394	2.38668
CTB-39G8.3	1.22477	2.21494	1.5353	0.591824
CTB-179K24.4	0	1.7203	1.06999	0
RP1-178F10.3	5.87056	5.62157	5.56808	5.9833
RP11-411B10.3	0.95262	1.74307	1.16494	0.8601
LINC00669	1.0391	0.900774	0.732076	0.698376
CTC-548K16.5	33.5726	37.4065	40.3644	39.8356
CTC-260E6.6	0.990263	0.916936	1.19756	1.24148
RP11-635N19.1	0.526357	1.01316	0.781533	0.605438
RP11-845C23.3	1.35591	1.37928	1.59222	1.42406
RP11-973H7.2	30.5124	32.0528	29.5337	33.6566
CTD-2265O21.3	1.76046	2.10606	2.12906	2.01551
CTC-503J8.6	40.3905	35.1097	37.3562	36.2463
RP11-559N14.5	2.87929	4.0776	4.92444	4.04099
AC005786.7	31.2319	29.3721	29.0632	26.7969
AC002398.11	1.31528	1.19501	1.38136	1.18195
AC004791.2	16.0639	11.0156	13.9678	16.7969
CTC-425F1.4	513.544	506.011	544.024	544.005
AC005944.2	5.67172	5.3844	4.5375	4.40569
CTD-2538C1.2	1.0112	0.927892	0.983816	1.04748
CIRBP-AS1	1.48318	2.31932	2.91397	1.98814

CTC-250I14.3	64.2791	58.3575	67.6022	73.4659
MIR24-2	2.03625	2.2767	2.1169	2.08874
RP11-373L24.1	1.64798	1.69442	1.62355	1.9038
CTD-2537I9.12	1.88771	1.86131	2.44669	1.78429
MIR497HG	3.05984	3.17846	3.36148	3.326
S1PR2	3.15077	2.7343	3.26333	2.95904
RP11-380M21.2	1.51155	1.38953	0.612177	0.660876
CTC-459F4.3	7.23753	8.1412	7.40293	7.57823
CTC-510F12.6	5.84506	5.97295	6.03901	6.75434
CTD-2587H24.5	11.6234	8.76362	9.96372	10.597
CTC-250I14.6	161.352	140.25	153.495	137.3
RP11-323N12.5	59.7668	63.2852	66.1648	69.9211
CTD-3220F14.1	0.676095	0.58446	1.15281	0.651557
RP11-552F3.13	3.24146	3.21671	4.33824	4.00689
RAD51L3-RFFL	0.008742	0.469701	1.208	0.495098
TRAPPC4	43.1616	38.7685	41.5081	45.3996
AC004152.5	1.43346	1.71566	1.79694	1.67606
CGB1	1.25385	0.833627	1.0024	0.867113
CTB-5E10.3	9.99113	7.34313	8.1585	8.36985
POLR2J2	3.01269	5.60917	4.74049	6.9733
CTD-2286N8.2	1.32506	0.691721	0.948787	0.935125
CTC-479C5.17	2.99959	2.09557	2.25849	1.77041
FDX1L	31.026	29.2268	30.3015	29.0484
ZNF224	1.65994	2.26588	1.95746	2.10021
AC005262.2	37.3578	28.3349	32.2083	29.2392
AC010525.5	1.23939	1.12451	0.262069	0.397814
TAF9P3	8.586	1.70132	0	0
CTB-151G24.1	1.00772	0.983751	1.75468	1.02513
LUZP6	424.439	395.479	408.89	430.09

AC002116.7	1.25113	1.06817	1.23053	1.39502
RP11-108P20.3	3.72088	3.55139	2.80298	3.23885
TMEM199	16.366	14.6556	16.6953	17.9233
CTD-2540B15.7	5.88756	3.9492	4.55612	5.88581
CTD-2265O21.7	6.03387	8.18103	7.73328	5.10488
AC024592.12	2.30383	3.02547	2.51225	2.565
CTB-102L5.4	7.75061	6.78448	9.38953	6.78988
AC009005.2	4.06958	3.66306	4.49501	4.10625
AC004623.2	14.2779	12.3642	13.5204	13.875
AC006132.1	2.35651	2.64456	3.21861	2.73306
CTB-25B13.13	0.47696	1.42389	0.600565	0.871891
CTD-3193K9.3	1.11697	0.885578	1.0829	1.08577
RP11-35G9.5	1.0002	0.89644	0.808159	0.741602
CTD-2659N19.2	9.2301	10.8532	8.5414	8.66249
SMIM22	1.03663	1.02411	1.30842	0.425523
LIN37	5.00476	5.95206	6.70205	6.47205
RP11-552F3.9	6.21465	5.7616	6.84914	5.81826
ACTG1	3068.65	2517.29	2821.91	2835.64
NDUFV2P1	3.76647	3.10206	2.59092	3.02203
CTB-191K22.5	14.772	18.9562	19.0285	17.9008
IDS	9.95928	10.2651	11.7178	11.2053
SCARNA12	5.26893	5.61297	6.54664	5.37705
TIMM17B	10.0864	10.8583	10.9554	10.1138
ZNF185	5.08445	5.61092	6.4858	5.49266
RP11-167N5.5	0.491201	0.835321	1.02269	0.889861
WDR13	16.5328	16.1705	19.2052	16.3298
NDUFA7	107.432	105.36	101.136	98.2937
PHLDB1	3.22272	3.88537	3.9351	3.91176
RPL13P5	2.37456	2.05827	2.22537	1.80533

PIM2	5.85545	6.17681	6.18302	6.02366
CTD-2126E3.4	0.970142	0.915816	1.11804	1.09984
ZNF322	2.1643	2.04999	2.20242	2.18809
AC018766.4	22.6462	22.398	23.1645	23.2652
AC073333.1	3.79041	3.3967	4.01228	5.32459
KMT2A	3.77381	4.28069	4.13202	4.25898
SLC35A2	17.3334	14.2568	16.3836	16.4699
RPS26P8	28.4657	25.2054	25.4819	27.6615
AC090673.2	0.509278	0.901919	1.01033	0.308438
TMLHE	3.52036	2.66562	2.88816	3.44
AC136297.1	26.4324	19.0945	29.3561	23.2435
L1CAM	1.72423	2.14246	2.38953	2.42187
AP000349.1	1.71218	3.21805	2.72756	2.77735
RP11-178G16.4	3.01385	2.81883	3.14891	3.89143
AC002055.4	14.2431	12.8048	13.4541	15.401
BCAP31	78.2368	71.576	72.8256	72.6155
AC007292.6	1.12595	0.939045	1.17535	0.870951
OTUD5	11.692	11.2141	12.2307	12.1828
CTD-2616J11.9	14.159	10.8173	10.5967	14.302
AL161784.1	67.5019	55.2181	60.1454	17.5122
IDH3G	39.1669	40.3202	38.2336	38.8168
DNASE1L1	3.75172	4.18824	4.52291	4.06494
PTOV1-AS1	6.61518	7.00411	6.89921	7.03539
RP1-203P18.1	1.44742	1.88247	1.83317	1.6041
TMEM185AP1	0.905045	0.783232	1.03348	0.924516
MAGED1	16.4309	21.7141	19.6362	21.2656
AC025262.1	58.5077	82.045	57.0053	83.2522
AC005253.2	1.36286	1.4467	1.26819	1.44845
RNU6-722P	0	0	0	2.76084

RP11-357C3.3	2.04368	1.63591	1.51178	1.60498
RP11-247A12.8	2.1747	2.20272	2.26566	2.29027
AL441883.1	86.8996	76.1167	75.6794	76.8604
NAPA-AS1	1.20233	2.04998	2.25688	2.04464
RNVU1-14	0.647901	0.872822	0.813435	2.0763
AC004466.1	1.27674	1.47482	1.53757	1.51227
AC104534.3	27.4668	29.1868	29.3427	33.9718
CTC-429P9.2	1.80499	1.76256	2.24523	2.99345
FMR1	5.75595	6.45137	5.78643	6.56022
FUNDC2	18.5039	15.7904	15.8207	17.0746
UBE4A	5.8974	3.57433	3.8772	4.70702
CTD-3149D2.4	30.0057	25.9637	33.9357	22.3451
CTD-3193O13.11	1.01212	1.11971	1.30899	0.801757
C12orf57	97.3263	82.953	88.5415	90.3932
RP11-91G21.1	0.86059	0.762641	0.822613	1.07325
FAM156A	2.55113	2.37792	2.46422	2.32246
LRRC23	1.94897	1.8391	1.93766	1.95178
CDCA3	25.759	21.5306	21.7837	23.1876
PORCN	4.33866	4.26871	4.4637	4.81775
PRICKLE3	2.52749	2.78877	3.61774	3.28088
PQBP1	36.6028	29.0825	33.1455	30.8247
CTD-3193O13.13	22.3723	20.7359	22.7488	20.9222
FKSG61	4.76842	2.89417	4.04331	2.9245
FAM3A	1.40582	1.61135	1.76184	1.52905
PIK3R2	12.5742	11.0192	14.2255	13.0719
HSD17B10	62.7888	63.4984	65.1469	60.5763
SPSB2	3.25665	4.32512	5.25059	4.55111
CTD-2396E7.10	7.7119	9.8289	10.6266	11.9765
GNB3	1.41575	1.25664	1.41758	1.55276

MAGEA3	51.1704	45.4005	46.1921	47.1154
CTD-2396E7.9	9.67856	11.6265	8.24656	6.26102
CTC-444N24.11	3.83254	3.65934	3.98843	3.97958
ARMCX6	8.66174	8.30443	8.00822	9.21889
MIR1184-1	0	1.41697	0.502793	0
AC137932.1	5.2901	6.3293	6.08167	5.38871
RP11-379K17.12	3.54785	3.5862	3.09975	3.95347
ARL14EPL	5.18744	5.26912	5.6422	6.28353
DKC1	74.364	56.3737	71.1323	68.6425
ARHGEF9	1.24938	0.992403	1.44459	1.42521
SNORA70	47.6373	35.9375	30.2069	41.4578
TIMM8A	8.01371	7.13921	7.40596	8.8196
CTC-246B18.8	5.79959	4.6191	4.5824	3.46708
TSPYL2	4.77881	4.21091	4.35585	4.08094
MIR1184-3	0	1.41697	0.502793	0
NAA10	72.9411	63.1496	64.5451	62.2129
AC006547.15	10.1549	8.96362	11.5921	11.5758
AL035252.1	105.518	114.506	137.412	108.141
CTD-3222D19.11	15.5046	17.9858	16.3731	18.9503
AC087645.1	40.63	29.7038	30.8384	32.9633
PHF8	4.19415	3.76877	4.27516	3.76273
AC119673.1	52.531	52.8605	47.1292	56.7011
CXorf40A	4.77335	3.89919	4.65183	4.78867
KATNB1	6.35127	6.11022	7.14609	6.98936
MIR1184-3	0	1.41697	0.502793	0
FAM156A	2.55113	2.37303	2.46422	2.32246
SYP	0.283626	0.911426	1.01019	0.699609
VN1R81P	3.22754	2.15607	2.09219	2.17008
TMSB15A	4.18093	3.84359	3.64059	4.20599

IQSEC2	2.07611	2.85378	3.21067	2.92292
TMEM187	3.26242	3.89933	3.49506	3.76754
EBP	61.2778	45.8569	48.188	48.2572
AC008443.1	1.99329	1.7792	1.6016	1.66426
CTD-3214H19.4	18.246	14.873	14.2788	9.19166
AC132192.1	2.39807	2.28512	2.07546	2.12952
AL022328.1	117.113	187.29	179.571	162.266
DOC2B	2.33149	2.3706	2.54693	2.71593
PRRG1	2.64037	2.55062	2.69247	3.06747
AC026407.1	0.023878	1.18451	0.548845	0
EMG1	66.1943	57.422	63.2221	71.0977
VBP1	55.9365	50.9139	47.2971	52.5034
USP5	31.6058	31.357	35.2222	35.7837
F8A3	2.83762	2.79936	3.01304	2.98069
AC104841.2	25.2478	23.1461	24.3286	23.2089
RBM3	148.17	135.532	130.183	139.038
HDAC8	5.48998	5.76375	5.78726	6.45899
CCDC22	3.28817	4.1154	3.8387	3.58759
CETN2	7.68785	7.71561	5.83562	7.02652
CTB-59C6.3	16.2895	16.7753	18.2039	17.5334
CTD-2619J13.16	2.61742	2.15413	2.79935	2.31873
TPI1	512.63	460.433	504.533	489.801
ATP5L	205.355	174.552	174.704	190.597
AL109927.1	156.913	160.259	139.793	168.589
EMD	56.2266	53.3112	57.0153	59.5874
MECP2	4.90902	3.98028	4.57905	5.01172
AC005339.2	18.5473	19.5498	16.5419	15.3417
AC007228.9	1.08309	0.899801	0.946055	1.13681
HIRA	3.34598	2.91574	3.46526	3.32052

FAM199X	6.36608	6.22869	6.16298	6.8611
AC239811.1	3.82087	3.00322	4.15681	3.50455
PHKA1	3.53134	3.38192	3.53121	3.77201
PLXNB3	1.23646	1.42141	1.29658	0.632905
RP11-244K5.8	4.84108	6.40774	7.47822	5.70319
CTD-2611O12.6	0.226942	0.709743	0.944321	1.1893
SLC10A3	9.4716	8.30276	8.71564	8.31486
MAGEA2	16.9115	15.3308	16.8014	16.6386
IDSP1	1.72141	1.11985	1.36784	1.99546
ABT1	16.0847	13.8383	13.8685	14.6962
RP11-1148L6.5	4.8172	3.99169	4.22338	4.51851
CTB-33G10.11	1.32544	1.35376	1.53043	3.01093
HNRNPH2	21.6034	20.1149	21.4901	22.054
AC006486.9	1.05902	0.210947	0.0350338	0.226564
MIR222	0	2.20716	0	0
HAUS7	22.9755	17.6376	20.6623	18.6946
GPKOW	7.80394	6.92464	6.93872	7.18069
CTB-33G10.6	4.94004	4.07522	3.71119	3.30237
AC233294.1	1.39232	1.52356	1.46647	1.29747
AC007382.1	2.49783	0.993956	2.44397	1.54734
GDI1	36.578	25.7345	30.8672	31.6303
RPH3AL	1.84209	1.93436	2.77698	2.6586
MTCP1	3.94606	2.66056	3.30008	2.83771
AL049829.1	173.23	161.851	145.553	153.172
CTC-444N24.8	1.59336	1.44219	1.40731	1.5983
CTD-2287O16.3	5.14474	6.85114	5.64658	4.70272
AL671561.1	3.33719	3.79919	4.72313	4.09096
F8A2	2.83762	2.80317	3.01304	2.98069
CTD-2105E13.14	2.31133	2.54583	2.78189	1.95032

NCS1	13.2798	9.17608	11.4157	12.253
CTC-518B2.12	13.035	12.6247	16.6219	13.3699
SUV39H1	7.94741	6.15791	6.55508	6.61816
CTD-2583A14.10	4.96643	3.62417	3.61763	3.75038
AC104534.2	2.1126	1.70111	2.08974	1.5092
ABCD1	1.2504	1.52165	1.66179	1.6431
SPIN4	1.74579	1.69002	1.85621	2.03275
AC018630.2	1.34208	0	0.286975	0
PLS3	23.7614	21.981	22.5195	23.5502
HCFC1	14.1998	11.8032	12.15	12.3217
AC110615.1	27.0353	30.812	21.4523	26.1863
CTC-429P9.4	13.2649	17.129	14.5604	16.3935
AC027307.3	1.57702	1.23945	1.16029	1.38876
MGC20647	1.70537	1.9165	2.31238	1.61924
GLA	21.6641	17.1327	16.6471	17.1796
LEPREL2	7.22335	7.42029	7.86371	7.89354
LPCAT3	8.93835	8.16475	10.6515	11.5844
F8A1	6.90765	6.88972	7.74304	7.57768
AC018867.2	0	1.38224	0.978457	2.91344
CTD-2545M3.2	82.3981	86.1715	79.7156	82.6501
AP001579.1	0	2.76717	0	0
C20ORF135	3.33066	3.85993	4.84081	3.27493
PLXNA3	9.39095	11.6324	11.6171	11.1083
AC135048.1	0.804624	0.868106	1.22784	0.893667
ESPNP	1.59784	1.65388	2.1228	1.94334
CXorf40B	10.695	8.54604	10.0841	9.60865
ATN1	22.1364	20.8755	23.775	21.9673
SNORA56	3.04321	0	0	0
ARHGAP4	18.5802	20.2999	21.0428	16.6295

RP11-256I23.1	0.798197	1.08268	1.25051	1.15035
CSAG3	4.07187	3.84438	4.19488	4.28138
RP11-34P13.15	3.89071	5.04781	4.71633	4.52761
TAZ	3.84485	4.15711	4.84967	4.34357
AC026806.2	8.2979	8.97497	8.21812	8.95485
CSAG2	7.16035	6.95245	7.22471	7.61834
FLJ00418	14.2999	13.5407	15.0569	13.75
SLC25A1	23.7817	21.6093	26.5501	27.5179
RP4-545K15.3	5.67611	6.60528	6.36073	6.65807
CKS1B	12.0759	11.2064	8.71697	10.3624
IRAK1	53.4061	41.9986	40.1951	38.1698
ATRX	4.87061	4.44095	3.8753	5.02096
AC114494.1	6.73581	6.97433	8.47874	8.48646
PTPN6	7.0378	4.1965	6.10811	6.22735
RP11-313P13.3	0.557814	1.02875	0.826091	0.99634
RPL10	1410.02	1351.65	1250.35	1365.08
RNU6ATAC	0	2.02125	1.43251	3.21019
HUWE1	12.4978	13.7837	13.3371	13.3712
AC005253.4	112.091	99.5022	106.267	105.516
VN1R82P	1.36783	1.50329	0.861141	1.68237
AL807752.1	0.645049	1.79312	3.09593	1.82433
LAGE3	8.90508	8.05724	8.06237	8.10852
AP001362.1	0.33215	0.690325	2.91346	0.508335
MTRNR2L12	2.90933	2.3696	1.69191	1.21844
MTM1	1.84961	1.79492	1.7833	2.28224
AC016629.7	4.0072	3.38193	5.36726	6.25916
PLP2	118.383	103.614	104.994	111.618
SLC9A6	3.60767	3.16175	3.44269	3.72555
AF011889.5	2.14463	2.75997	2.75205	2.88498

CTC-429P9.3	2.97099	1.99265	2.41545	2.63356
CTC-360G5.6	1.04659	2.03974	1.58692	1.36952
PBDC1	9.2814	8.03304	8.41075	9.23488
PHB2	179.019	162.977	178.562	186.358
MAGEA2	16.9001	15.3308	16.8013	16.6386
COX7B	142.5	132.028	126.875	132.66
CTD-3222D19.10	0.898586	2.14454	0.763126	0
G6PD	45.6894	49.75	48.541	47.5651
AC006449.1	30.6428	29.707	29.6184	26.8379
AC114494.2	2.72775	3.28203	4.278	4.61772
HDAC6	13.9475	14.0731	15.6991	14.1246
CTD-2619J13.23	0.452997	0.861267	1.04156	0.704269
MAGEA6	5.15082	4.52761	5.15536	3.82682
RP11-98F14.11	2.90672	1.15933	0.409953	1.21962
AC004447.2	7.82494	7.81082	8.00185	7.69978
PGAM4	0	2.85082	2.9307	3.21576
CTD-3193O13.8	2.6301	1.40115	2.55646	2.68444
AC007192.6	5.89877	8.1273	8.43652	6.83745
AC007193.8	160.733	151.674	143.806	126.568
ARMCX3	3.84508	3.87866	3.43698	3.64282
AC093157.1	6.13369	5.59918	5.39656	5.76555
RP11-727F15.12	5.78192	6.63136	6.24805	5.49921
KIFC3	9.65716	8.99137	10.4066	10.0907
FBXO17	8.95694	9.13588	9.21516	8.9108
MMGT1	6.62101	6.07055	5.62616	6.63747
CLCN5	1.2308	1.03595	1.00424	1.17491
CTD-2192J16.22	11.778	14.247	10.0965	7.49809
CTD-2231E14.8	76.3744	65.0892	65.5796	71.5707
CTC-246B18.10	22.216	8.72035	6.9967	7.45834

TAF9B	8.97069	6.93317	7.16099	8.51751
RP11-457M11.2	2.46738	2.2601	1.89289	2.29743
PPP2R2D	6.21062	6.18589	6.84538	7.41771
IFT46	4.34431	4.08463	4.29238	4.41342
AL136376.1	23.3308	23.4592	22.6442	22.8493
RP11-869B15.1	1.00978	1.16925	1.02834	1.00705
CTB-12A17.3	130.181	113.263	115.909	120.116
RP1-265C24.9	1.40441	1.46999	1.29165	1.76318
AL358113.1	18.4811	18.9664	21.7208	20.6772
RPL36A	634.276	560.142	473.135	621.284
MIR221	2.84511	6.74309	2.40102	5.35925
TMEM25	8.49041	6.74352	8.28067	7.2598
MPP1	3.04175	2.80242	2.98608	3.12237
FLNA	177.355	176.59	187.954	169.442
IKBKG	4.18504	4.00206	4.71787	4.34294
NSDHL	11.0098	9.39188	9.94454	9.83614
ZNF587B	4.07329	3.41082	3.53704	3.88131
AC018766.5	7.05524	7.83543	8.48659	7.06345
AL138743.1	14.4433	12.0469	12.9108	13.4761
DSTNP2	1.82876	1.25284	1.49633	1.90626
CTD-2207O23.11	6.87183	9.20677	12.9421	7.78807
CTB-50E14.5	1.71173	1.72249	1.80965	1.88459
ITGB1P1	21.2535	23.0108	24.503	24.4624
RP11-770J1.5	7.555	4.40256	4.53165	5.45116
ARCN1	37.5597	34.9621	37.602	38.9746
SMC1A	25.3846	17.075	21.0888	21.5558
RAB11B-AS1	1.59213	2.52745	2.45864	2.73116
RP11-298J23.8	1.46571	0	0	0
AC018755.16	1.77009	1.88873	1.58402	1.41834

AC116407.2	0.954956	1.03599	0.727861	0.571517
CTD-2616J11.11	1.52629	1.3506	1.22587	1.03208
HMGB3	44.3855	41.2774	37.1818	43.3204
CTC-273B12.6	1.89181	1.37408	1.93808	1.71806
LRRC3DN	1.87827	2.41392	2.66913	2.64208
CTD-3131K8.2	2.9513	4.58198	2.98289	3.03909
CD99L2	1.53143	1.92249	1.95185	1.84946
CTB-174O21.2	10.3822	9.28668	8.34447	9.66212
HIST2H2BC	0.98469	0.928123	1.40947	1.01329
TBC1D25	2.99545	2.69338	2.93502	2.63219
RP11-353N4.6	24.3188	12.8996	17.9335	29.8992
SSR4	79.7555	75.8776	74.6869	73.8715
ENO2	8.69134	7.71723	8.99525	8.80583
CTC-360G5.8	0.603472	1.12301	0.138929	0.776317
AL590822.2	2.8876	2.70889	3.17743	2.6345
TMEM185A	3.74922	3.60693	3.7096	3.80547
AC093677.1	1.33595	1.43874	1.04406	1.13242
AC005000.4	30.4293	31.7648	27.1867	30.3604
L34079.4	1.56094	1.84398	2.3629	1.59701
SLC38A5	15.8381	13.1023	13.3131	13.142
AC006128.2	2.0546	2.6703	2.60859	1.99757
AP000758.1	53.4338	51.2164	56.2135	62.4769
ZBTB33	2.68694	2.67568	2.43064	2.93293
AC005523.2	38.895	36.274	37.3634	36.2381
RP11-18I14.10	12.6866	12.9264	13.104	14.1974
DDX6	11.2574	10.902	10.595	12.3696
CMC4	2.06683	1.81702	1.83187	2.23297
AF196970.3	4.28835	3.44472	4.18095	4.09277
AC017081.1	6.17332	6.07137	7.23231	7.51537

AC010441.1	17.1058	19.4548	22.2714	20.7812
MAGT1	6.68791	5.79309	6.10611	6.74516
FAM50A	19.0913	16.5288	17.5629	17.5296
CTD-2571L23.6	1.21525	0.646644	0.807489	0.671955
PGK1	300.324	284.479	277.367	282.702
ATP6AP1	28.1115	26.8215	28.8263	27.5923
RABL2B	6.12049	6.33247	7.0318	6.6127
CTD-3128G10.6	1.85202	2.19057	2.49811	2.04533
AC008982.2	1.00322	0.85819	0.987897	0.446002
WDR45	9.90862	13.88	12.2664	11.4366
AC244163.2	16.5714	14.1361	13.456	15.2025
NBPF10	1.95014	1.9391	2.02946	2.14371
UBL4A	17.0508	13.4674	13.9938	13.7806
RPL36A-HNRNPH2	12.4422	20.5283	26.7801	33.5111
AC009060.1	6.42399	8.13426	7.53394	8.48508
TFE3	3.21275	3.11105	3.73216	3.45309
CTC-273B12.8	2.81591	3.04618	3.48369	3.15128
MTMR1	6.8596	5.63509	6.10063	6.33736
RP11-178G16.5	7.10181	5.16829	4.48261	4.61927
FTH1P8	0.864108	1.13473	0.576761	0.504843
FLJ20306	4.44375	4.05304	3.86989	4.2403
GPR107	3.02427	3.24616	3.46478	3.75545
DUSP9	5.48086	5.06401	5.17954	4.51687
AL353354.2	287.615	263.353	224.662	237.607
CTD-3193O13.14	5.34491	3.51759	3.71555	4.31042
KDM5C	11.5227	11.6694	10.9797	11.1244
CTD-3099C6.9	1.07876	0.933301	0.94257	1.1175
RP11-15H20.5	0.992816	1.04009	1.25485	1.16073
MAMLD1	0.925591	1.08537	1.30863	1.25199

U47924.27	3.90399	4.9529	3.9851	4.6045
EGLN2	49.0287	45.7515	48.7148	46.6497
HIST2H2BD	1.54809	1.27855	1.92514	1.40206
FKSG63	3.09615	2.77967	4.65087	4.14284
CTD-2583A14.8	0.963181	1.22229	1.33903	1.32496
AC040977.1	0.755337	1.05311	1.36611	1.30302
FTSJ1	29.2895	24.9008	26.6092	25.7483
BRCC3	6.50513	5.30053	6.07375	6.33721
RP11-266J6.2	0.320368	0.763846	1.00243	0.593522
RP11-3P17.5	3.7168	4.4414	3.86696	3.25801
SNHG8	180.881	165.258	149.776	165.698
COMMD3-BMI1	28.0737	27.1023	27.3988	26.3818
RP11-589N15.2	1.82928	1.89333	1.53752	0.84618
RMRP	13.2334	8.27237	8.70338	8.76691
RP11-248J18.2	3.54846	2.85002	3.02769	2.82625
RP11-489N22.3	0.8018	1.47971	0.868626	0.510721
AP006621.9	1.03901	1.13181	0.998839	0.858391
RP3-467L1.6	1.03191	1.39631	1.67573	2.00403
RP11-442H21.2	23.7012	64.4351	30.5701	27.4343
RP11-73M18.7	6.9459	7.46269	6.58965	6.93716
RP11-2B6.3	1.27434	0.246809	0.86536	0.752694
RP11-849F2.9	1.42591	1.10557	0.865413	0.908042
RP11-738E22.3	1.48812	1.40069	1.25076	1.36572
RP11-797A18.6	1.43346	1.33647	1.8463	1.77659
LUC7L2	23.0279	18.1486	19.8922	20.9325
RP11-73M18.8	10.1172	9.25251	7.86037	8.53339
RP13-238F13.5	5.78403	5.22126	5.34845	5.77699
RP11-73M18.9	3.32119	3.57304	2.91825	2.98757
RP5-940J5.9	7112.04	6674.97	6624.49	6676.01

RP11-498E2.9	0	1.14373	0.197997	0
RP3-430N8.10	22.2057	30.8876	24.3121	28.7601
RP11-95D17.1	1.19511	1.09183	1.00836	1.13316
RP11-932O9.10	1.56303	0.610355	0.793987	0.685854
RP11-338N10.3	0.321316	0.574088	1.35874	0.606658
RP11-498E2.7	3.82333	2.54394	2.2075	2.63141
RP11-34P13.16	2.38532	5.86485	2.03837	2.07905
RP11-497H16.9	6.8767	5.87118	5.35545	6.1733
RP11-362K14.5	2.93931	2.71799	2.70011	2.78608
RP3-430N8.11	14.6109	13.8519	12.6854	13.6039
RP11-395P17.11	6.34759	5.98343	6.1063	6.24183
CTD-3074O7.12	0.785196	0.94063	1.16595	1.30184
RP11-343N15.5	3.00298	3.06572	2.9468	3.22431
RP11-178L8.7	1.93907	2.81629	2.50892	2.10482
RP11-540B6.6	2.17331	2.06912	2.41361	2.30263
RP3-462E2.5	0.970884	1.07787	0.967417	0.935488
RP11-343N15.5	1.1404	1.22639	1.18208	1.27605
RP11-571M6.17	1.02843	0.922786	1.35049	1.5686
LL22NC03-N27C7.1	1.03163	1.06453	1.28972	0.726594
RP4-769N13.7	1.61922	1.42363	1.47577	1.37191
CTD-3092A11.2	1.63054	1.52349	1.73892	1.69116
CTC-487M23.5	2.87952	3.14472	2.57678	2.35732
RP6-99M1.2	1.47292	1.41002	1.54469	1.52912
RP11-127L20.5	3.23308	4.5926	3.60893	3.3063
RP6-99M1.2	1.79367	1.71355	1.98982	2.03926
RP5-935K16.1	12.2691	11.8255	11.3033	13.077
RP11-245P10.8	5.97829	5.3115	5.11544	5.84893
RP11-24B19.3	4.23441	4.28648	3.74777	4.44829
RP11-498E2.8	2.32115	0.987556	1.14378	1.2471

RNU11	2.36441	1.51961	5.28565	3.09124
RP11-393M11.2	1.66751	1.00057	1.62936	1.60394
RP11-282O18.6	3.9761	2.94906	2.3487	2.76754
RP11-57A19.5	1.40434	1.39564	1.25501	1.19787
TERC	1.14259	2.36216	2.68391	1.78142
AC004257.3	2.11373	1.50798	2.55741	2.50784
CTD-2267D19.6	1.92647	1.45983	1.52545	1.50605
RP11-646I6.6	4.12115	3.88984	3.38273	3.90026
F11R	2.18446	2.37265	3.32946	2.2913
NCBP2-AS2	36.1228	32.2143	38.7461	34.7952
RP5-1024G6.8	3.5224	3.28475	3.39955	3.39069
RP11-382B18.4	1.04676	0.533982	0.636403	0.997607
RP11-793H13.11	0.901244	1.67296	1.39102	1.22751
RP11-159N11.4	1.02402	1.06249	0.596237	0.859921
BIVM-ERCC5	1.92807	1.64979	1.67508	2.01244
RP3-365I19.2	13.0896	17.7141	19.8808	12.9834
RP11-453E17.3	1.07872	1.24448	1.63039	1.53305
NBPF10	3.24143	3.35635	3.50336	3.70193
RP11-17P16.2	0	0	0.356434	1.61511
CNOT3	6.06491	5.84374	5.97319	5.36969
NDUFB11	33.7562	35.4347	34.9993	34.1209
C14orf80	16.2823	13.3766	17.6947	16.1658
RP11-734K2.4	0.520669	0.850874	1.07532	0.866655
NLRP2	6.29249	5.70084	4.99914	4.62256
MTA1	35.4467	41.5996	50.4262	44.242
RP11-796E10.1	0.931972	1.66778	2.01781	2.51654
AP2B1	10.2893	12.6688	11.7022	11.3021
RNVU1-5	7.11864	10.2184	4.84918	7.22731
TSEN34	16.2866	13.8902	13.8928	11.9268

RP11-420L9.5	0.714641	0.837054	1.0021	0.912867
CTD-2124B8.2	1.56631	1.98251	1.47439	1.37675
RP11-379K17.11	1.07683	1.24619	1.20906	0.901083
RP11-1186N24.5	6.50567	6.55733	6.84887	6.40484
RP11-51F16.9	2.91419	1.90096	1.68455	1.40385
CTC-550B14.7	1.07915	0.914913	0.75915	0.718459
RP5-1165K10.1	1.47349	1.8566	2.01312	0.946009
AC239811.2	1.67052	1.55588	1.45692	1.5208
CDK16	2.32474	3.13145	3.47991	2.75813
TAF15	78.0247	67.0018	76.4758	80.817
AC245389.1	2.41633	1.87905	2.02484	2.48891
YTHDF3-AS1	1.99453	0.651471	1.00192	1.23816
RP11-216F19.2	2.88177	3.67857	2.7865	3.28726
RP11-342K6.1	4.87754	4.01252	4.48894	4.57191
RNVU1-7	0	0	0	1.68479
RP11-461L13.2	1.39221	1.00562	0.649477	1.05151
MOB4	31.4016	24.0906	22.7367	27.4174
RP11-385F7.1	0.892771	0.697846	1.09728	1.15683
RP11-501C14.9	0.819984	0.993532	1.11015	0.51549
CRIP1	58.824	56.5857	53.8223	58.527
RP11-632K20.8	0	0.383004	1.60548	0
RP1-187N21.4	75.6264	71.7638	72.3869	68.91
CTD-2583A14.11	4.90957	4.62298	5.23021	4.96475
RP11-730B22.1	0.931324	1.12894	0.779319	0
EPS8L1	1.44775	1.50349	2.13211	1.35233
RP11-2E11.9	2.73126	2.53986	3.35051	2.51199
SRGAP2D	3.56214	2.94358	3.31497	3.63686
CRIP2	9.83295	10.6771	13.1234	11.3261
RP11-65L3.4	0.731585	0.873721	0.475782	1.06179

AC245884.3	0	4.39604	4.08195	0
RP11-1112G13.3	5.39744	1.56379	0	0
RDH13	6.04841	4.56681	4.71078	4.02798
NDUFA3	46.6825	46.7226	39.0221	34.5722
MBOAT7	12.2378	13.9681	12.5753	10.4233
RP11-10C24.1	0.736622	1.08517	1.02702	1.10947
RP2	1.67345	1.67371	1.71028	1.86674
AC243756.2	2.41633	1.87905	2.02484	2.48891
RP11-379H18.1	1.34669	1.96489	1.90316	1.96699
RP4-769N13.6	2.90087	3.54685	3.20443	3.49276
RP11-708J19.2	1.13926	1.21526	0.685697	0.721183
RP3-523E19.2	2.94132	2.51003	3.30048	3.24512
NBPF10	1.38152	1.46233	1.49497	1.43998
SRXN1	108.159	76.4386	78.863	85.1664
UBA1	65.9442	67.7202	69.2254	67.0603
CRIP1	4.12503	5.14269	6.82262	7.32142
RP11-324I22.4	0.659146	1.10087	0.877713	0.701383
RP11-474P12.5	2.21365	1.78077	1.83721	2.16155
RBM10	19.9973	18.1829	19.4332	18.3607
RP11-84C13.1	4.17817	4.85319	4.71145	4.74132
RP11-138I18.1	1.07259	1.13338	1.04917	1.39153
PHF16	2.28026	2.09869	2.1634	2.44884
PACS2	9.56958	11.0741	13.5841	11.3
RP11-307C12.12	0.60847	0.839108	0.715235	1.01826
AC242842.2	2.81515	2.70025	2.62544	2.62966
AC239799.1	1.05188	1.04658	1.0197	1.092
RP13-238F13.3	3.77419	1.89019	2.92431	1.95844
PRPF31	13.1017	12.0777	10.7451	9.06763
RP11-521B24.5	14.6646	17.009	17.7065	17.7935

RP11-342K6.2	2.47503	3.20384	2.71253	1.75058
RP11-382A18.3	0	0	1.07363	0
CICP14	2.15529	2.32674	2.41839	1.96482
RPS9	214.62	219.49	183.574	172.36
RP11-486G15.2	1.68853	1.31407	1.45736	1.49755
AC243547.1	0.227489	0.213902	2.68169	2.7727
RP3-365I19.2	20.4574	24.9673	23.0622	12.9834
RP11-95I16.4	0.747117	1.3017	0.881153	1.09909
TMEM121	2.69396	2.10247	2.90818	2.20507
RP11-1012A1.10	0.954524	1.96999	1.30306	0.328238
RP11-787I22.3	2.98578	4.40731	3.90727	2.6574
RPS9	107.296	110.567	118.765	126.748
MBOAT7	6.5844	8.05581	8.54017	7.68146
CTB-113I20.2	0.7727	2.10013	1.65519	1.54078
SMDT1	11.8865	12.7158	12.0409	12.6762
ZMYM6	0.913728	1.00141	0.925615	0.948272
MBOAT7	6.55094	8.05575	8.55113	7.66668
RP1-202O8.3	1.30242	1.26524	0.940056	0.876515
BEND3	1.43599	1.30077	1.53641	1.60248
RP3-337H4.9	3.64315	3.01406	2.35923	2.797
RPS9	107.296	110.567	118.765	126.748
RP11-216P16.2	1.1977	1.04484	1.36206	1.10003
CNOT3	3.28003	2.71515	3.35896	3.25227
CTD-2589H19.6	1.20532	1.33338	0.960843	0.935152
RP11-254F7.3	3.15281	3.97938	2.92808	3.1289
MBOAT7	6.5844	8.0507	8.54705	7.68707
RP11-321N4.5	10.3757	8.45477	8.74403	9.75756
CTC-428G20.6	1.18176	1.12548	0.824312	1.12251
SNORA51	24.1608	20.4154	12.4941	16.906

NDUFA3	20.0011	20.6051	21.1812	22.4972
TSEN34	7.90213	6.62841	7.9198	7.89424
CNOT3	2.99337	2.60261	3.23786	3.20237
TMC4	0.926465	1.36083	1.55154	1.57274
RNF34	14.2975	12.5462	12.6601	13.2522
TSEN34	7.87935	6.6677	7.91953	7.89616
TSEN34	7.88574	6.64839	7.93768	7.88055
NDUFA3	20.0506	20.623	21.2327	22.5334
RP1-178F15.5	1.14824	1.25546	1.80068	1.76796
XXcos-LUCA11.4	1.14074	1.57162	1.55238	1.50944
RPS9	107.296	110.567	118.765	126.748
C6orf203	2.76013	3.25784	3.26417	4.0248
RP11-51J9.5	1.26988	1.44163	1.66642	1.71921
RP11-97C16.1	3.80426	2.58195	3.11986	2.23657
SRSF8	9.27721	13.9377	12.4249	13.0344
RP4-635E18.8	1.56549	1.60836	1.51332	1.22805
MBOAT7	6.56225	8.05135	8.55031	7.71034
NDUFA3	20.0506	20.621	21.2313	22.5334
PARG	1.35127	1.50828	1.44193	1.46686
CTD-2287O16.5	1.18821	1.2794	1.34377	1.36367
CNOT3	3.28003	2.71515	3.35896	3.25227
TMC4	0.925847	1.35694	1.55154	1.57274
RP11-443B20.1	3.7551	3.29386	3.6968	3.31747
RP11-188P20.3	2.47164	1.37341	1.80808	2.04322
RP11-242F4.2	1.4451	1.81827	2.2256	2.46752
CNOT3	3.2464	2.69473	3.31782	3.21998
PRPF31	7.58604	6.42974	7.55013	7.31846
RP11-427H3.1	5.88052	5.14988	5.8718	5.88306
AGAP6	2.79484	2.37127	2.46183	2.29937

PRPF31	6.42381	5.63856	6.46198	6.32449
RP11-927P21.12	0	1.47086	0	0
RP11-884K10.7	1.96978	1.70534	1.74242	1.83502
TMC4	0.925847	1.35694	1.55154	1.57274
CTD-2256P15.4	47.2874	41.5515	39.157	40.832
RP3-337O18.9	5.61946	6.17063	6.65824	5.86634
RP11-23P13.7	2.01484	1.67941	2.29525	2.17339
RP11-583F2.6	0	5.39939	0	0
RP1-313I6.12	2.035	2.62213	2.00647	2.20884
ABBA01057584.1	2.10044	2.5743	2.61941	3.01673
RP11-215G15.5	5.49636	4.97664	5.32605	5.28908
RP1-199J3.7	2.96641	2.19903	2.1193	1.86011
RP1-178F15.4	1.0343	1.24455	0.958804	1.41515
TMC4	0.926465	1.35694	1.55154	1.57274
GTF2H5	3.45231	3.56464	3.49593	3.79755
RP11-458N5.1	1.52968	3.90565	2.47609	2.072
NDUFA6	28.1722	27.6515	27.0252	31.657
RP11-423P10.2	0.996521	0.841262	0.972152	1.04496
RP11-503P10.1	2.55383	2.2238	2.753	2.73056
RNA18S5	429.875	478.697	568.216	659.099
PRPF31	7.15574	6.16528	7.16024	6.94703
U47924.28	40.5388	36.3849	36.9142	42.354
FAM21A	9.51873	9.39306	8.75919	9.34655
RNA5SP317	0	1.02705	0.452082	0
RP11-348P10.2	1.24022	1.17196	1.39795	1.25097
CTD-2186M15.3	1.26566	1.17166	1.03897	0.979423
TSEN34	7.88477	6.65922	7.91384	7.89854
CTC-365E16.1	2.77922	2.85821	2.63031	2.54353
AC092299.8	8.7784	11.6593	11.6147	11.3947

RP11-155O18.6	2.12536	1.05784	2.36204	2.39282
XXcos-LUCA11.5	0	0.431016	0.437912	1.63565
RPS9	107.296	110.567	118.765	126.748
CTB-113P19.5	1.06657	0.784591	0.670985	0.658603
RP1-261G23.7	21.1227	17.6248	16.3712	13.7285
GS1-393G12.14	1.29676	0.864103	0.896075	0.909926
MBOAT7	6.57356	8.05516	8.54715	7.69678
ZNF275	2.25435	2.06809	1.66695	1.75113
TEX261	18.698	14.1841	16.1086	17.8018
RPS9	107.296	110.567	118.765	126.748
TSEN34	7.87289	6.65474	7.9287	7.90552
RP11-574K11.29	0.134463	1.05708	1.15626	0.437091
RP11-465B22.8	1.47364	2.82805	2.67387	1.68409
RP11-755B10.4	1.60966	1.74116	1.84353	1.79593
RP11-627J17.1	3.15246	3.27247	2.44027	3.73979
RP1-286D6.5	2.22026	2.14148	2.18299	2.72639
RP11-350N15.6	19.8225	15.5713	18.1811	19.5069
U4	0	0	0	2.28463
ORAI1	6.19504	7.54626	8.39012	8.59493
RP13-582O9.7	3.33705	2.42802	4.65125	3.66227
U47924.31	10.2017	9.08568	9.64018	9.89655
RP11-802O23.3	11.1172	10.8273	11.7148	9.98222
U47924.30	3.62972	3.74337	3.8661	4.0464
PRPF31	7.387	6.27977	7.34932	7.10321
HIST2H2AA3	1.43615	1.42181	1.87086	1.6764
PRPF31	7.38478	6.29378	7.36966	7.16314
U47924.30	4.7481	4.94761	4.39169	4.35101
ANAPC5	48.619	46.5756	50.583	53.2018
PLEKHO1	1.97427	1.81342	1.82868	1.76035

RP11-927P21.9	2.05796	0.856167	0.181878	0
RP1-20C7.6	0.757526	1.27864	0.932325	1.07987
TMC4	0.926465	1.35189	1.55154	1.57274
AC242426.1	4.12411	4.21862	4.02663	4.49846
5S_rRNA	0	3.36693	0	0
RP11-489E7.4	10.4887	11.0853	11.3382	9.36828
RP11-500C11.3	9.19225	6.97959	8.42716	8.50894
XXbac-BPG252P9.10	0.923612	0.866283	1.00472	1.1998
RP11-791G15.2	1.80176	1.31663	1.36458	1.24186
RP1-40E16.12	1.61708	1.3124	1.56987	1.60709
RP11-140K17.3	6.00353	5.49811	5.79657	5.48387
PDSS2	0.908128	0.850991	1.11318	1.21202
U47924.31	7.36965	6.4343	7.5326	7.99907
NUDT3	4.45968	4.17128	4.41624	4.89663
NDUFA3	20.0011	20.6051	21.1593	22.4969
RP4-594A5.1	0.988923	0.471443	0.820341	1.26641
LINC00843	1.79114	0.933544	1.31723	1.61193
NDUFA6	28.1722	27.6515	27.0252	31.657
WBP7	6.35787	5.72789	6.05904	5.60869
RP11-53O19.3	1.94815	2.20795	2.26095	2.67101
RP11-722E23.2	4.14658	2.53233	2.03397	2.67167
CNOT3	3.27336	2.71648	3.35951	3.25515
RP1-30M3.5	17.3983	18.2786	17.2999	21.2073
KDM2B	12.0792	8.09259	9.31054	8.5591
U4	0	3.87688	2.72616	2.023
CTC-428H11.2	2.80142	2.71431	2.54834	3.05726
RP4-605O3.4	1.19182	0.788134	0.801021	0.755916
RP11-446N19.1	4.19438	4.30907	4.79332	4.21193
ZNF33B	4.43306	3.81659	4.53178	4.9371

AC211429.1	8.08601	6.94796	7.84756	7.89047
CD24	1.87328	2.51118	2.27055	2.69313
RP11-284F21.10	14.9019	20	20.5853	18.301
NDUFA3	20.0011	20.6051	21.1509	22.4969
RP11-438J1.1	2.60276	2.17906	1.83244	1.33917
FAM47E	3.69236	3.47773	3.40868	3.24867
PRPF31	7.00971	6.07565	7.10696	6.83861
RP11-762H8.4	17.7422	16.8469	16.925	19.4723
RPS9	107.296	110.567	118.765	126.748
TMC4	0.926465	1.35581	1.55154	1.57274
RP13-131K19.6	1.47748	1.16981	1.58966	1.06891
RP11-182L21.6	2.52397	2.97215	2.64317	2.91766
RP11-391M1.4	4.33977	3.38674	3.11352	3.2507
RP4-758J18.13	3.2279	2.57861	2.97331	2.89874
CNOT3	3.24732	2.69612	3.32076	3.22356
PRPF31	6.38534	5.59829	6.40019	6.32087
NDUFA3	20.0506	20.6233	21.2255	22.5338
RP1-317E23.7	35.0489	44.3645	38.3816	38.6869
PRPF31	7.07737	6.12004	7.02251	6.91258
TSEN34	7.90116	6.6534	7.90222	7.88211
RP11-532M24.1	1.06279	0.69263	0.660075	0.999277
NDUFA3	20.0506	20.6213	21.2226	22.5334
RP11-54O7.17	2.15866	1.50525	1.62931	1.90607
MBOAT7	6.57356	8.05137	8.54662	7.68571
TGFA	9.6552	8.14075	9.45007	10.4173
CTD-2044J15.2	1.57413	1.26715	1.18438	1.54463
RP1-63M2.6	29.5447	31.1513	32.6902	27.5523
CTC-329H14.4	1.68032	2.10976	1.5813	2.2399
RP11-254F7.4	0.876118	0.953784	0.79463	1.33787

RP11-79P5.9	1.10471	1.09957	0.873201	1.04466
SNORA28	5.76755	3.46556	1.22278	1.83103
NDUFA3	20.0011	20.6051	21.1668	22.4972
XXbac-BPG252P9.9	234.232	185.486	152.699	122.959

Table S6: The expression change of genes related to cell-death after the transfection of siRNAs with or without N

	FZD6-0N	FZD6-sN	FZD6-dN
BAK1	**		
TRIB3	**		
BBC3	**	**	
UNC5A	**	**	
RRAGC	**		
F3	**		
GAN	**	**	
RMDN3	**	**	**
TNFRSF21	**		
DDIT4	**		
INPP5D	**	**	
SMN1	**	**	**
TNFRSF10C	**	*	
PRF1	**	**	**
SIAH2	**	*	*
SMN2	**	*	*

**, $P < 0.01$; *, $P < 0.05$

Table S7: The number of essential genes in previous RNAi screenings using cancer cell lines

NO. of essential genes	NO. of cancer cell line(s) used	NO. of shRNAs	NO. of target genes	References
25	3	8203	2924	[11]
169	1	6K	NA	[7]
268 *	12	45,000	9500	[10]
134 $	102	54,020	11,194	[12]
131 $	72	78,432	16,056	[6]

***:** in more than 8 of the 12 cancer cell lines

$: in >50% of the cancer cell lines
